# Blood Peptidome-Degradome Profile of Breast Cancer

**DOI:** 10.1371/journal.pone.0013133

**Published:** 2010-10-18

**Authors:** Yufeng Shen, Nikola Tolić, Tao Liu, Rui Zhao, Brianne O. Petritis, Marina A. Gritsenko, David G. Camp, Ronald J. Moore, Samuel O. Purvine, Francisco J. Esteva, Richard D. Smith

**Affiliations:** 1 Biological Science Division, Pacific Northwest National Laboratory, Richland, Washington, United States of America; 2 Environmental Molecular Sciences Laboratory, Pacific Northwest National Laboratory, Richland, Washington, United States of America; 3 Biological Design Interdisciplinary Graduate Degree Program, Arizona State University, Tempe, Arizona, United States of America; 4 Departments of Breast Medical Oncology and Molecular and Cellular Oncology, The University of Texas M. D. Anderson Cancer Center, Houston, Texas, United States of America; George Mason University, United States of America

## Abstract

**Background:**

Cancer invasion and metastasis are closely associated with activities within the degradome; however, little is known about whether these activities can be detected in the blood of cancer patients.

**Methodology and Principal Findings:**

The peptidome-degradome profiles of pooled blood plasma sampled from 15 breast cancer patients (BCP) and age, race, and menopausal status matched control healthy persons (HP) were globally characterized using advanced comprehensive separations combined with tandem Fourier transform mass spectrometry and new data analysis approaches that facilitated top-down peptidomic analysis. The BCP pool displayed 71 degradome protein substrates that encompassed 839 distinct peptidome peptides. In contrast, the HP 50 degradome substrates found encompassed 425 peptides. We find that the ratios of the peptidome peptide relative abundances can vary as much as >4000 fold between BCP and HP. The experimental results also show differential degradation of substrates in the BCP sample in their functional domains, including the proteolytic and inhibitory sites of the plasmin-antiplasmin and thrombin-antithrombin systems, the main chains of the extracellular matrix protection proteins, the excessive degradation of innate immune system key convertases and membrane attack complex components, as well as several other cancer suppressor proteins.

**Conclusions:**

Degradomics-peptidomics profiling of blood plasma is highly sensitive to changes not evidenced by conventional bottom-up proteomics and potentially provides unique signatures of possible diagnostic utility.

## Introduction

Breast cancer is the most common malignancy in Western women; in 2009, more than 192,000 women were estimated to be diagnosed and ∼40,000 died of this disease in the United States alone [1]. Breast cancer is a heterogeneous disease, and prognosis is determined largely by tumor size, shape, location, and metastasis in addition to molecular characteristics, such as whether the tumor is hormone receptor-positive or -negative, genetic factors, and the rate of cell division [2]. Tumor markers, such as the estrogen receptor, progesterone receptor, and the human epidermal growth factor receptor 2, are routinely employed to assess invasive breast cancers, and in cases of advanced disease, circulating tumor markers CA15-3/BR27-29 or carcinoembryonic antigen may be used to monitor response to therapies [3]. However, there are presently no tumor markers for early detection of the disease in otherwise healthy women [4]. Impeding the development of such markers using proteomics approaches is the expectation that protein targets in blood reflective of cancers, if present, are most likely present at extremely low levels [5].

Cancer progression, invasion, and metastasis require and/or bring about changes to tumor microenvironments that involve protein degradation by cancer degradome proteases [6]. In addition to proteases, the degradome includes protease activators and inhibitors, and degradation substrates [7], [8]. Protease inhibitors account for 5–10% of all cancer-related drugs [9], while protein substrate degradation products afford a potentially rich pool for cancer biomarker discovery [10], [11]. As protein degradation products, intracellular and/or intercellular peptides that constitute the peptidome have been explored for their potential as biomarkers [12]; however, the small pieces of peptide sequences detected and identified are difficult to correlate to available cancer degradomic information, possibly because such small sequences are likely the terminal products of the multi-stage degradation of protein substrates.

Herein, we report on degradomic behaviors based upon the analysis of the peptidomes of pooled breast cancer patients and control healthy persons. Our strategy involved comprehensive separations combined with tandem Fourier transform mass spectrometry and new data analysis approaches that facilitated top-down, global peptidomic analysis [13]. The results obtained herein demonstrate remarkable variations between the pooled samples that far exceed those evident using conventional bottom-up proteomics approaches. The present results obtained for the samples carefully selected suggest that the breast cancer patients have increased degradation of functional domains of cancer-relevant proteins, including proteases and inhibitors, extracellular matrix (ECM)-relevant proteins, innate immune system key components, and other protein molecules functioning to suppress cancers. Overall, the present results support the view that the peptidome/degradome is a potentially rich source of makers, support the use of the present top-down proteomic analytical strategies for obtain of the relevant unique information, and highlight the need for more extensive degradomic-peptidomic studies of individual samples, as is enabled by the present work.

## Results

Blood plasma samples for this work were collected under the identical lab protocol from15 breast cancer patients (BCP) (ER positive, Her2 negative; invasive ductal carcinoma; 5 are stage I, 7 are stage II and 3 are stage III) and 15 control healthy persons (HP) of matched age, race, and menopausal status. The blood plasma peptidomes of these samples were isolated without observable discrimination and analyzed in parallel under well-controlled conditions (see Methods below).

**Figure 1:**
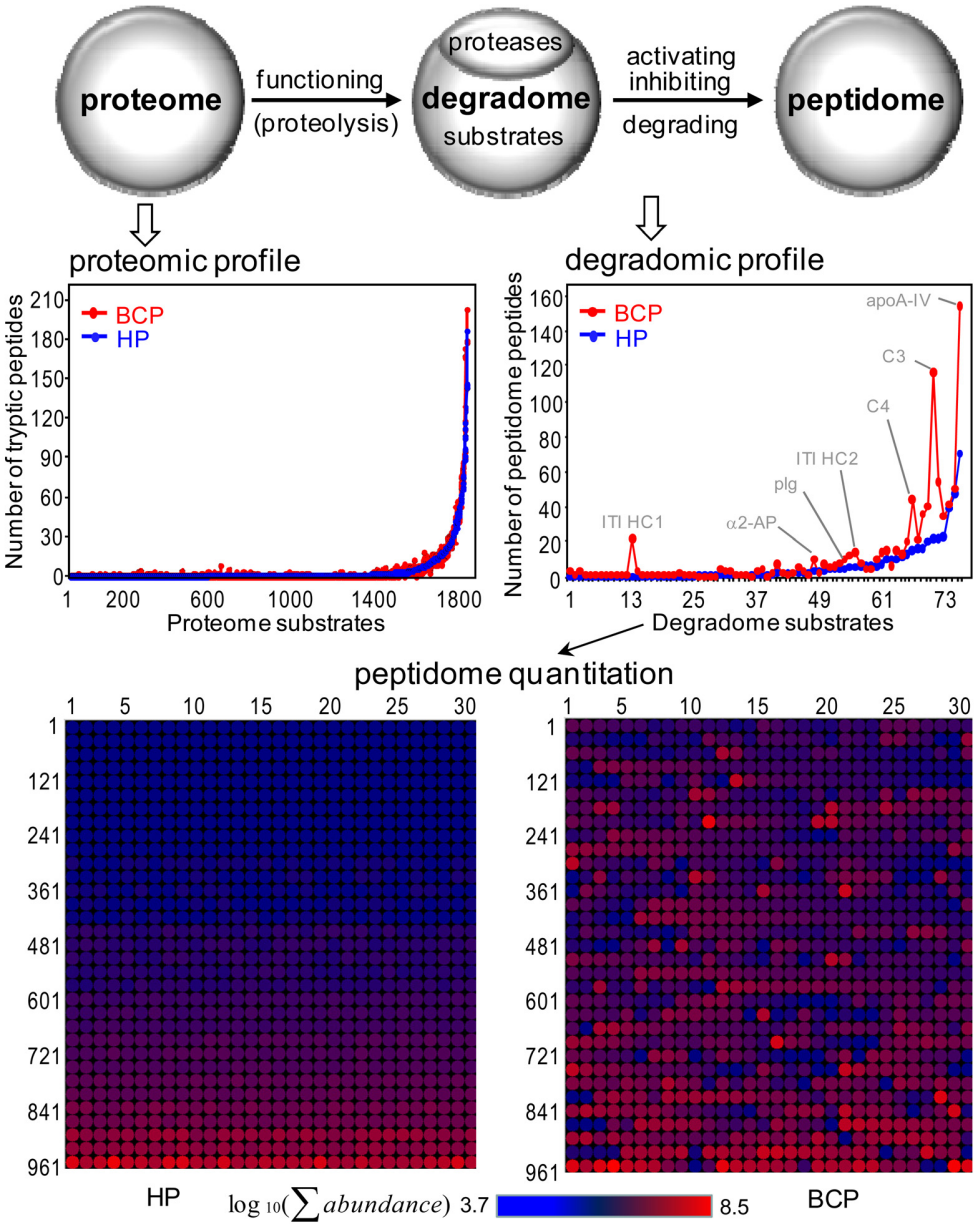
The proteome and degradome profiles for the 15 BCP and control HP samples. The degradome is a sub-proteome that participates in the proteolytic activities and produces peptidome (top). The proteomic and degradomic measurements are represented by tryptic peptides and peptidome peptides, respectively (middle). The BCP degradome is compared to the HP degradome using the peptidome peptide abundances (bottom). Details for each degradome substrate, peptidome peptide and its quantification, proteome protein, and tryptic peptide are given in Tables S1 and S2.

**Figure 2:**
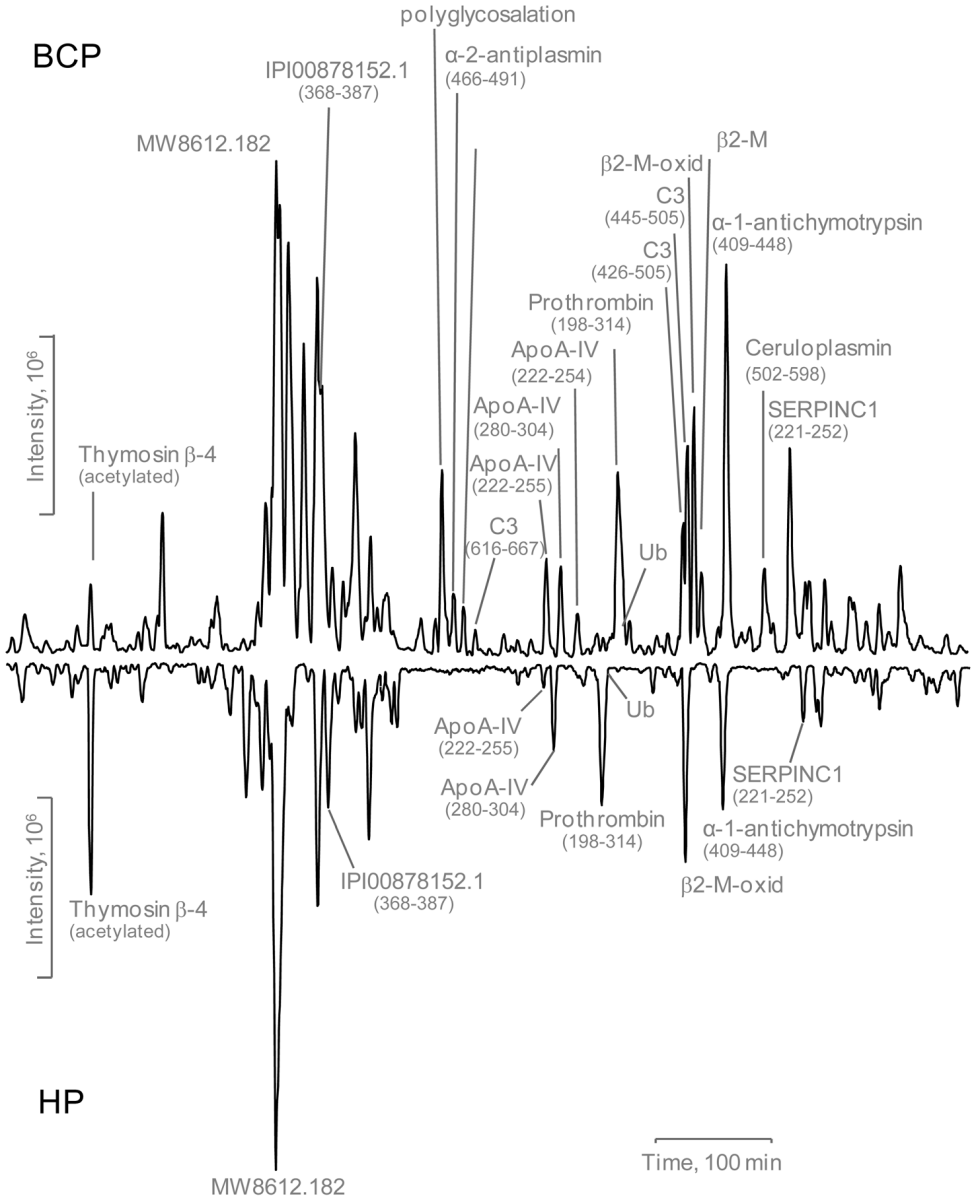
The comparative base peak chromatograms of the BCP and HP peptidome components. Differences exist between the base peaks of the BCP and HP peptidome major components.

The BCP and the control HP have very similar sets of plasma proteins (Table S1) identified using a conventional bottom-up proteomics method (i.e., analysis of peptides originating from tryptic digestions of proteome proteins), but show strikingly different degradation patterns (Figure 1) of these proteins that are revealed from analysis of the peptidome peptides. The BCP degradome encompasses 71 protein substrates that generate 839 distinct peptidome peptides, while the HP degradome encompasses 50 substrates that generate 425 peptides (Table S2), suggesting that the BCP have a more active degradome than the HP. The ratios of the peptidome peptide relative abundances can vary by >4000 fold between the BCP and the HP samples tested (Table S2). The striking degradome differences observed for the BCP also includes selective changes of major high abundance peptidome peptides (Figure 2). Moreover, the degradation observed for the BCP is selective in the degradome substrates. For example, inter-α trypsin inhibitor heavy chain 1 (ITI HC1) produces 22 peptidome peptides in the BCP, but none in the HP; whereas ITI HC4 displays similar selectivity (53 peptides in the BCP, and 48 in the HP).

**Figure 3:**
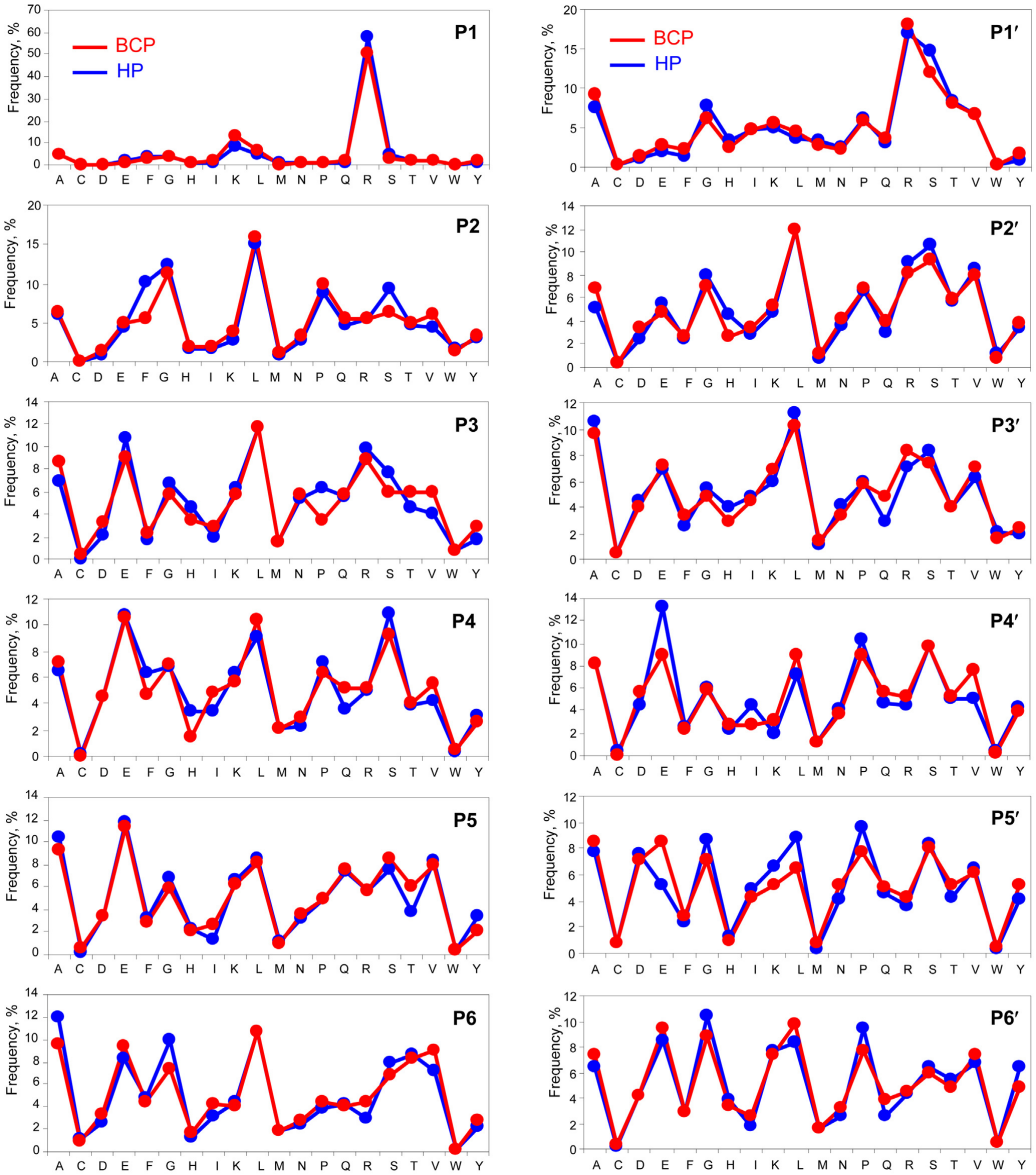
The net cleavage specificity of BCP and HP proteases for the degradome-wide substrates. The cleavage positions P and P′ defined by Schechter I. and Berger A. (*Papain Biochem Biophys Res Commun* 1967, 27: 157–162) are adopted herein to present the specificity measured from ∼1000 peptidome peptides listed in Table S2.

**Figure 4:**
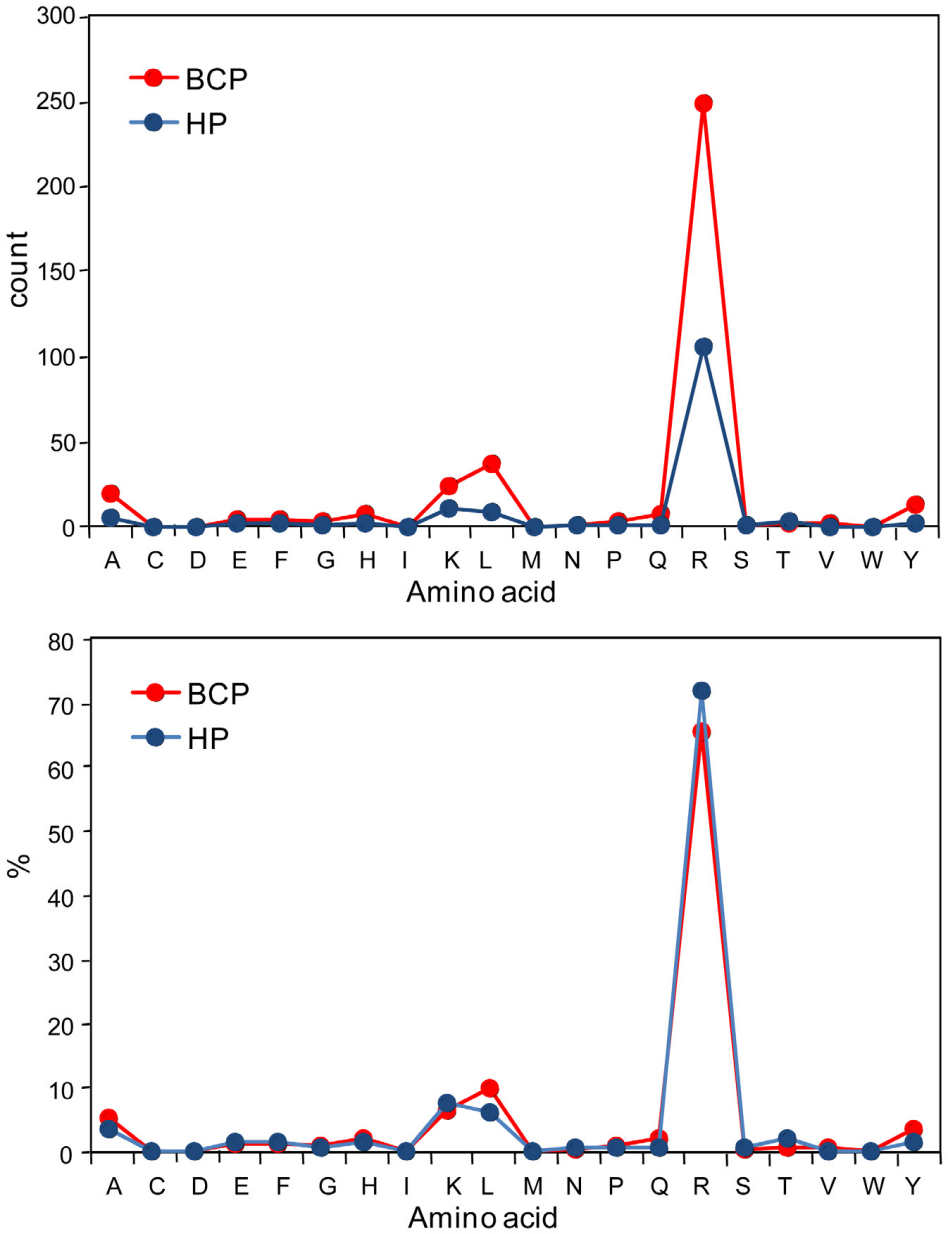
The net cleavage specificity of BCP and HP proteases for the degradome individual substrate. Protein substrate apoA-IV is used for this examination. The counts and frequencies (percentages) for amino acids at P1 in the BCP and HP are used to present the specificity. The BCP degradome proteases are more active than the HP ones to cleave more sites at P1-Ala, Lys, Leu, Arg, and Tyr. For the BCP, the P1-Leu, Tyr, and Ala are relatively preferred for cleavage in comparison with other P1-amino acids.

Figure 3 shows that the overall cleavage specificities of degradome proteases active in the BCP and HP are similar, which suggests that the BCP degradome has no proteases that aberrantly cleave protein substrates. However, the cleavage specificity for individual substrates, as illustrated in Figure 4 for ApoA-IV, can vary significantly, which suggests differences in the substrate proteolytic activity between the BCP and the HP examined.

**Figure 5:**
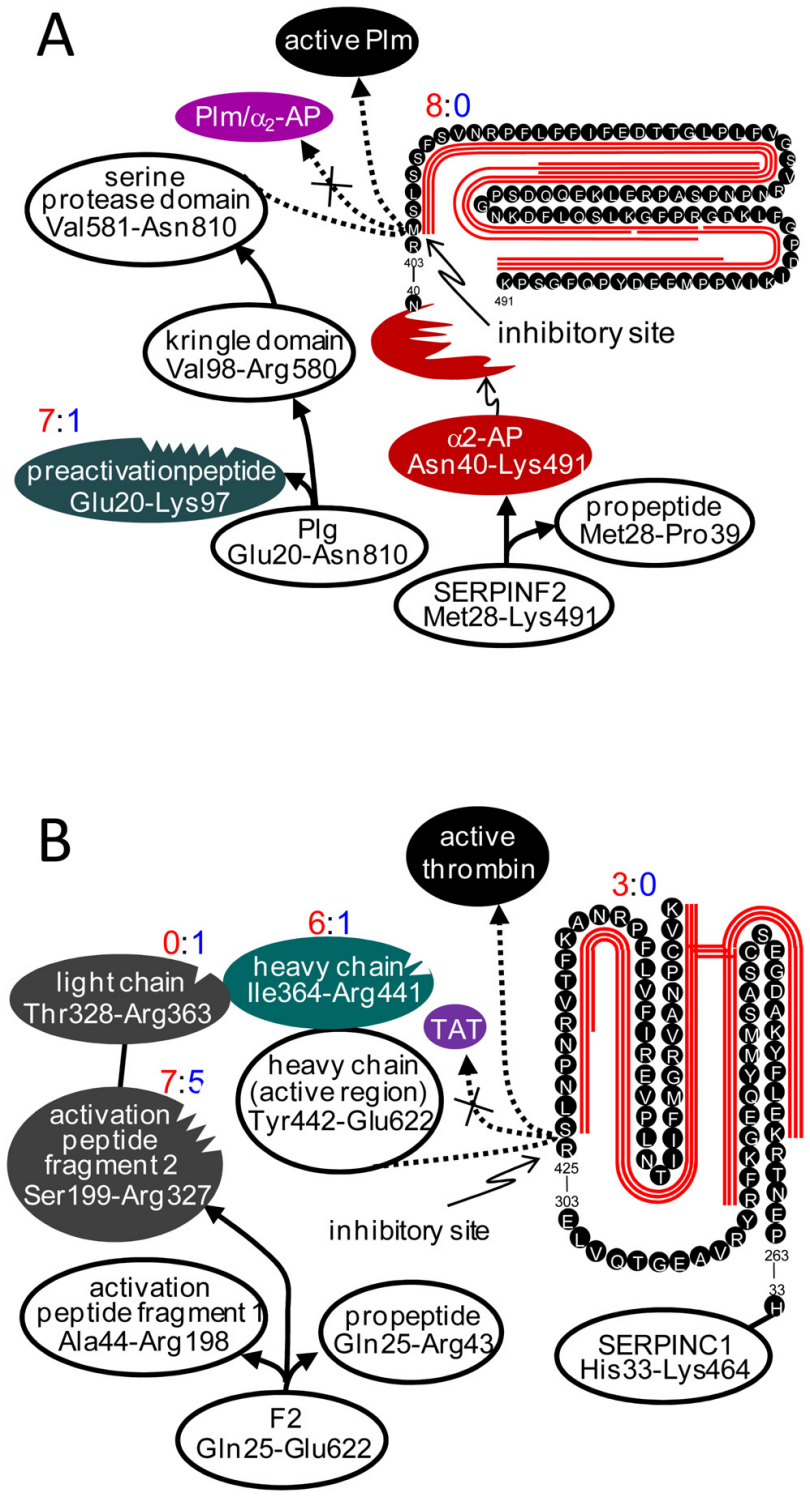
Selective degradation of function domains of protease systems observed for the tested BCP. (A) For Plm-α_2_-AP system, Plg degrades on its preactivation peptide; while α_2_-AP on the inhibitory function bonds. (B) For FII-AT system, FII degrades on its heavy chain, not on its proteolytic function domain; while AT degrades on its inhibitory function bonds to prevent formation of thrombin-antithrombin (TAT) complex. The red lines along the sequences represent the peptidome peptides solely observed from the BCP; the differential degradation is represented by the numbers (red for the BCP and blue for the HP) of different peptides observed.

Plasmin (Plm) is an extracellular protease that physiologically and pathologically participates in the remolding of tissues and female reproductive organs [14]. The Plm proenzyme plasminogen (Plg) is secreted to the extracellular space where it is converted to Plm by removal of the preactivation peptide domain (Glu20-Lys97) through cleavage of Lys97-Val98 (catalyzed by Plm) [15], while the Plm proteolytic activity is controlled by α_2_-antiplasmin (α_2_-AP) where Arg403-Met404 and Met404-Ser405 inhibit Plm (and other serine proteases) through formation of stable complexes (e.g., Plm/α_2_-AP) [16]. The activated Plm has been found to degrade the ECM and its adhesive proteins [17], [18], a required step for cancer invasion and metastasis. Figure 5A shows observations of the differential degradation for Plm-α_2_-AP system of the BCP tested. The differential degradation (7∶1) for the BCP and control HP occurs for the Plg preactivation peptide (Table S2), which would accelerate conversion of Plg to Plm; while the proteolysis function domain Plm light chain remains unchanged. Degradation of α_2_-AP is observed at its inhibitory bonds only for the BCP (8∶0 for BCP:HP). The implication of Plm in cancer invasion and metastasis has focused on the Plm activation by urokinase-type Plg activator (uPA) through cleaving Lys580-Val581 and releasing the serine protease, i.e., Plm light chain [19]. Our degradomic study demonstrates that the Plm-α_2_-AP system proteolytic activity for the BCP could also be enhanced by selective degradation of Plg and α_2_-AP sequence functional domains.

The prothrombin (FII)-antithrombin (AT) system is involved in angiogenesis, as well as in cancer invasion and metastasis [20], [21]. This system displays selective and differential degradations (Figure 5B) similar to the Plm-α_2_-AP system in the BCP (Figure 5A). The increased proteolytic activity of the FII-AT system is in agreement with observations of >100 fold more fibrinopeptide A in the BCP than in the control HP (Table S2).

The matrix metalloproteinases (MMPs) [22], a major type of proteases that degrade ECM, are found to have no differences between the BCP and the control HP samples examined from either degradomic or proteomic measurements (Table S3). This is not in contradiction with the hypothesis that the MMPs signature is a poor marker for prognosis of patients with primary breast cancer [23].

**Figure 6:**
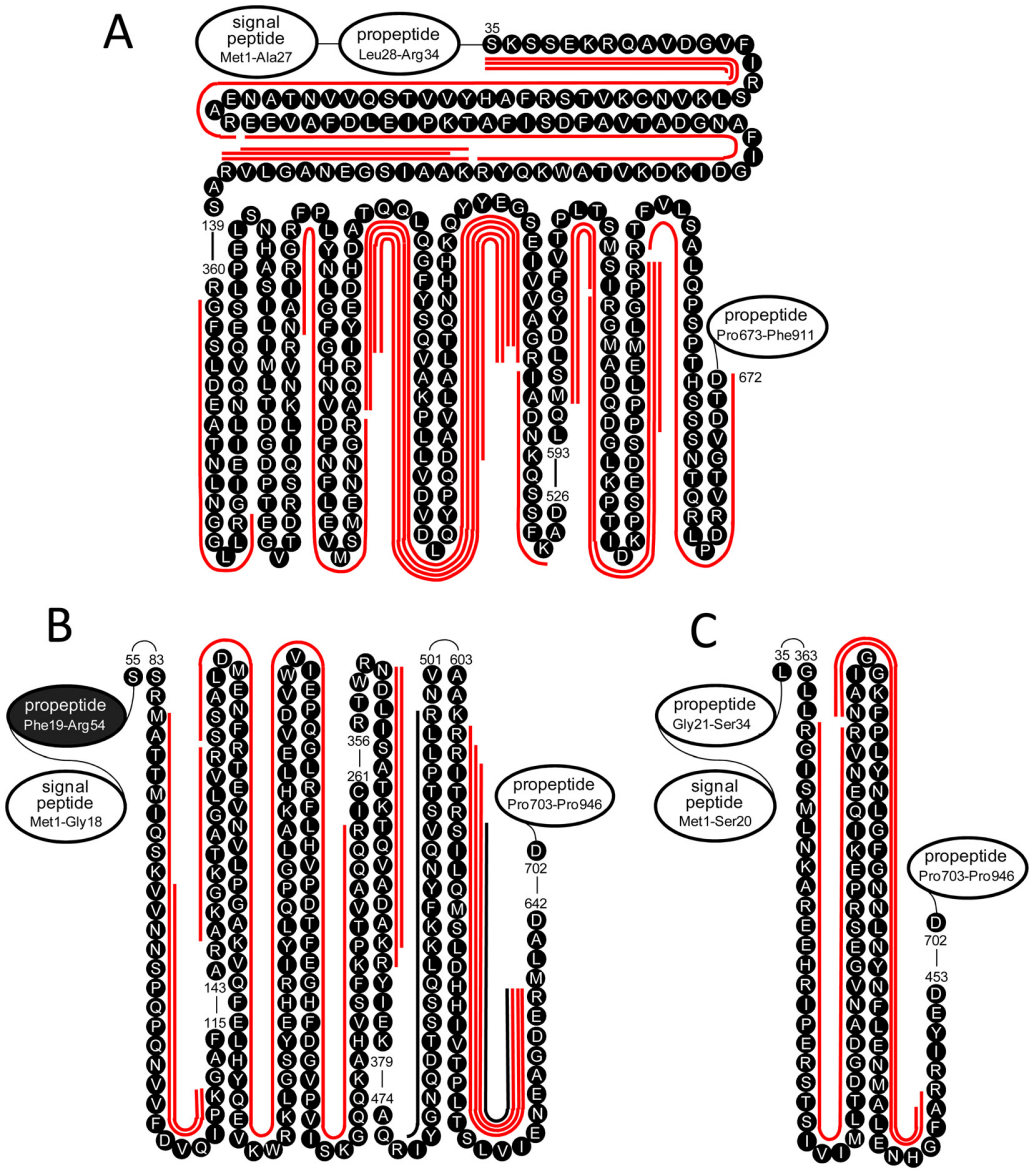
Selective degradation of ITI HC1-3 observed for the tested BCP. (A) The peptidome peptides were observed solely from the HC1 main chain, not from the two propeptides (blank-filled ovals). (B) The peptidome peptides were observed dominantly from the HC2 main chain, except for one (not labeled) from the propeptide (black color-filled oval). (C) The peptidome peptides were observed solely from the HC3 main chain, not from the two propeptides (blank-filled ovals).

Tissue remodeling for cancer invasion and metastasis requires degradation of the ECM [24] that binds surface proximal proteins (Figure S1). It has been found that mediated by TSG-6 [25], the ECM hyaluronan (HA) binds ITI heavy chains (HCs), which make up the complexes IαI [HC1/HC3/LC (LC: ITI light chain)], PαI (HC3/LC), and IαIL (HC2/LC) [26], [27] to inhibit plasmin and other proteases and then protect and stabilize the ECM. Figures 6A–C shows that HC1, HC2, and HC3 are differentially degraded in the BCP tested. The differential degradation occurs to the HC1-3 main chains, preventing them from functioning to form the protein complexes. It is noted that the differential degradation occurs only in HC1-3 that function to protect and stabilize the ECM, but not HC4-5 (Table S2) that have other functions. In addition, only a few peptidome peptides are observed in the BCP for the ECM components, suggesting that these proteins themselves have not undergone broad degradation yet in the ECP examined (Table S2).

**Figure 7:**
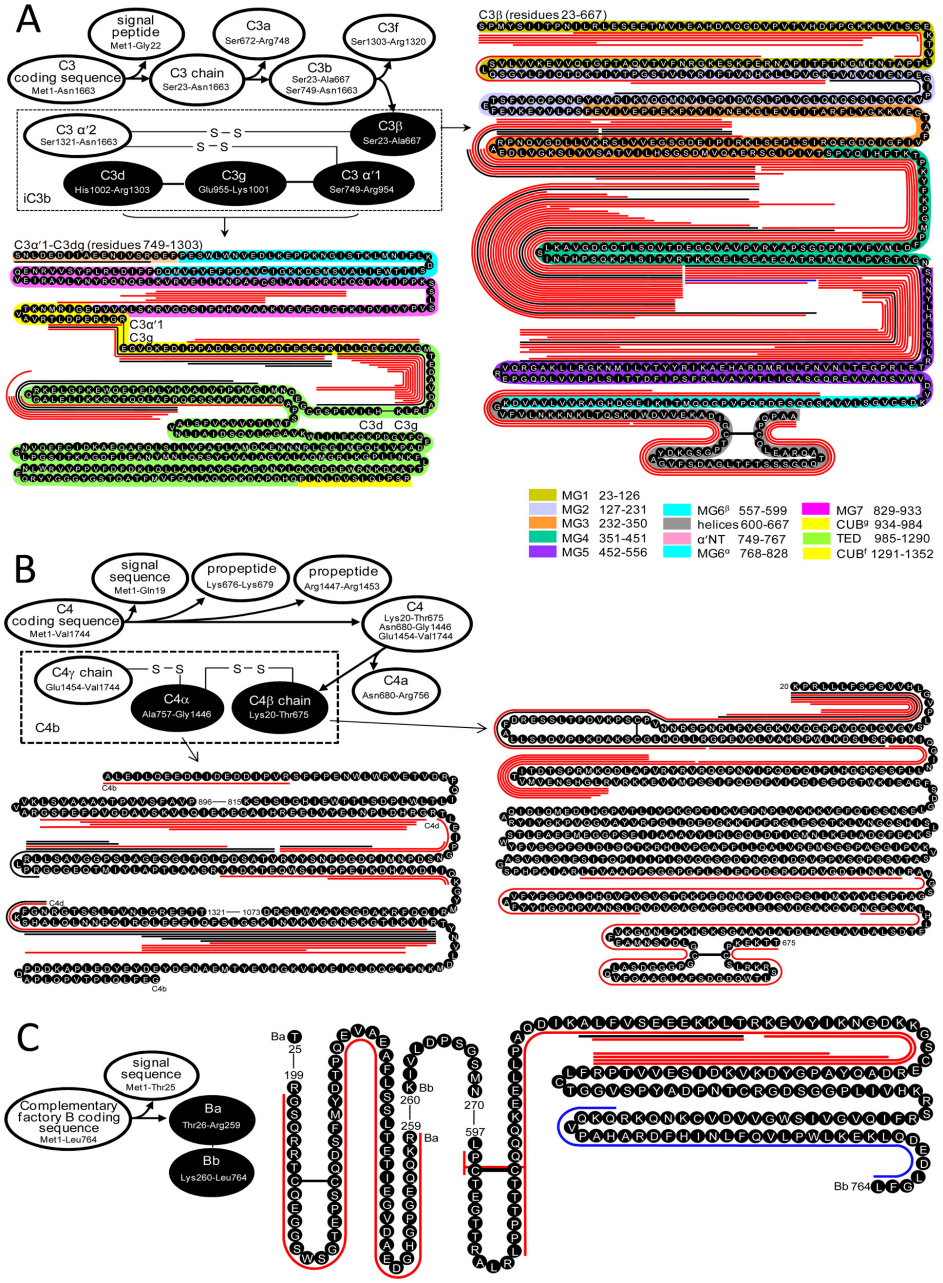
Degradation of complement system convertases observed for the tested BCP. (A) For C3, the degradation was solely on the C3d, C3g, C3α′1 and C3β (black color-filled ovals), not on the other fragments (blank-filled ovals); the C3 sequence domains are colored for comparison of the domains observed with degradation. (B) For C4, the degradation was solely on the C4b and C4β (black color-filled ovals), not on the other fragments (blank-filled ovals); the degradation of the front portion of the C4b fragment was observed solely for the tested BCP. (C) For complement factor B, the degradation was solely on the complement factor Ba and Bb (black color-filled ovals), not on the other fragments (blank-filled ovals). The red, blue, and black lines along the amino acid sequences represent the peptidome peptides identified solely from the tested BCP, HP, and both the BCP and HP, respectively.

**Figure 8:**
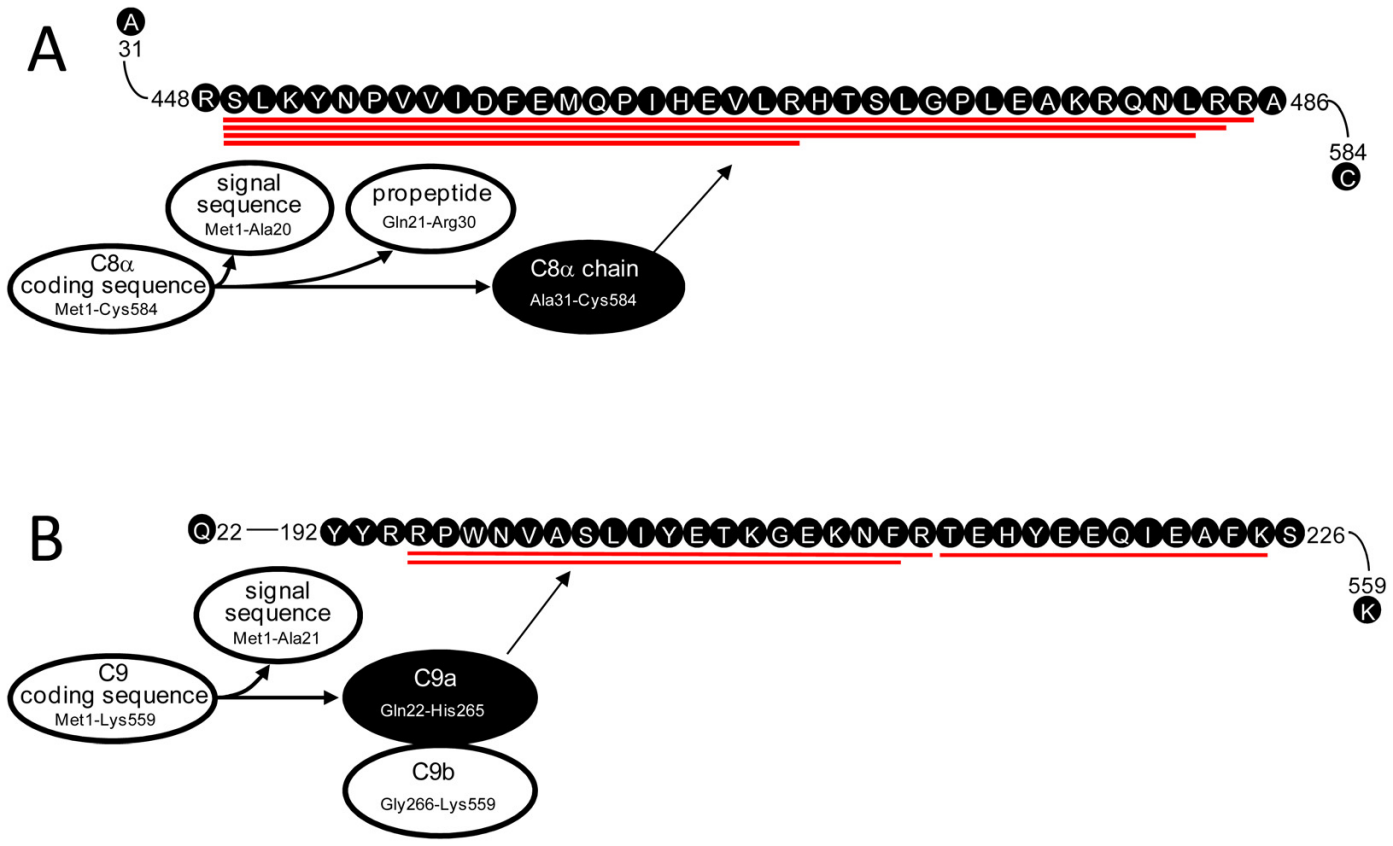
Degradation of complement system MAC key components observed for the tested BCP. (A) For C8, the degradation was solely on the C8α (black color-filled ovals), not on the other fragments (blank-filled ovals). (B) For C9, the degradation was solely on the C9α (black color-filled ovals), not on the other fragments (blank-filled ovals). The red, blue, and black lines along the amino acid sequences have the same significances as for Figure 7.

The complement system is an essential part of the innate immune system [28] and has multiple roles in inhibiting or promoting tumor growth [29]. Figures 7A–C show the differential degradations for complement system key convertases in the BCP. Convertases C3, which occupies a central position in the system, displays differential degradation in the BCP (5-fold more peptides than in the HP, Table S2). The differential degradation occurs to C3b (Figure 7A), but not to the anaphylatoxin C3a released, which could reduce the activation of C5 to C5b that initiates assembly of MAC. The degradation of C3b further occurs to iC3b (C3α, C3β, and C3dg) that contains the TED domain for C3b to attach the target (e.g., tumor cell) surface and enhances antibody-dependent cellular cytotoxicity rather than C3f released during C3b conformation changes [30]. The peptidome peptides observed show that C3dg links to C3α'1, C3c (C3α+C3β) is disrupted, and C3β exists separately during iC3b degradation. These results suggest that iC3b degradation occurs prior to the conformational changes from iC3b to C3dg [30]. Except for MG6^α^ and CUB^f^, all iC3b domains [30], [31] contribute peptidome peptides, and the peptides generated encompass sequences across C3b multiple (≥2) domains. MG4 and MG5 release the greatest numbers of peptides, while MG7 and helix domains release peptides only in the BCP. Convertases C4b and factor B (fB) that activate C3 to C3b in the lectin, classical, and alternative pathways are differentially degraded in the BCP (Figures 7B and 7C) with releasing ∼3-fold more peptidome peptides than in HP (Table S2). The differential degradation of C4b and fB occur to C4α, C4β, fBa, and fBb domains. In addition to the complement system convertases, the complement system membrane attack complex (MAC) key components C8 and C9 are also differentially degraded in the BCP (Figures 8A and 8B). The differential degradation occurs to the α chains of C8 and C9. These peptidomic-degradomic evidences show that the BCP examined have excessive proteolysis destruction of the convertases and MAC components, which could hinder initiating and/or result in dysfunction of the immune complement system (Figure S2). Consistent with previous report that C3, C4, C8, and C9 are deposited on the breast cancer cell surface [32], the selective and differential degradation observed for the tested BCP are at the surface attachment domains of these convertases and components.

**Figure 9:**
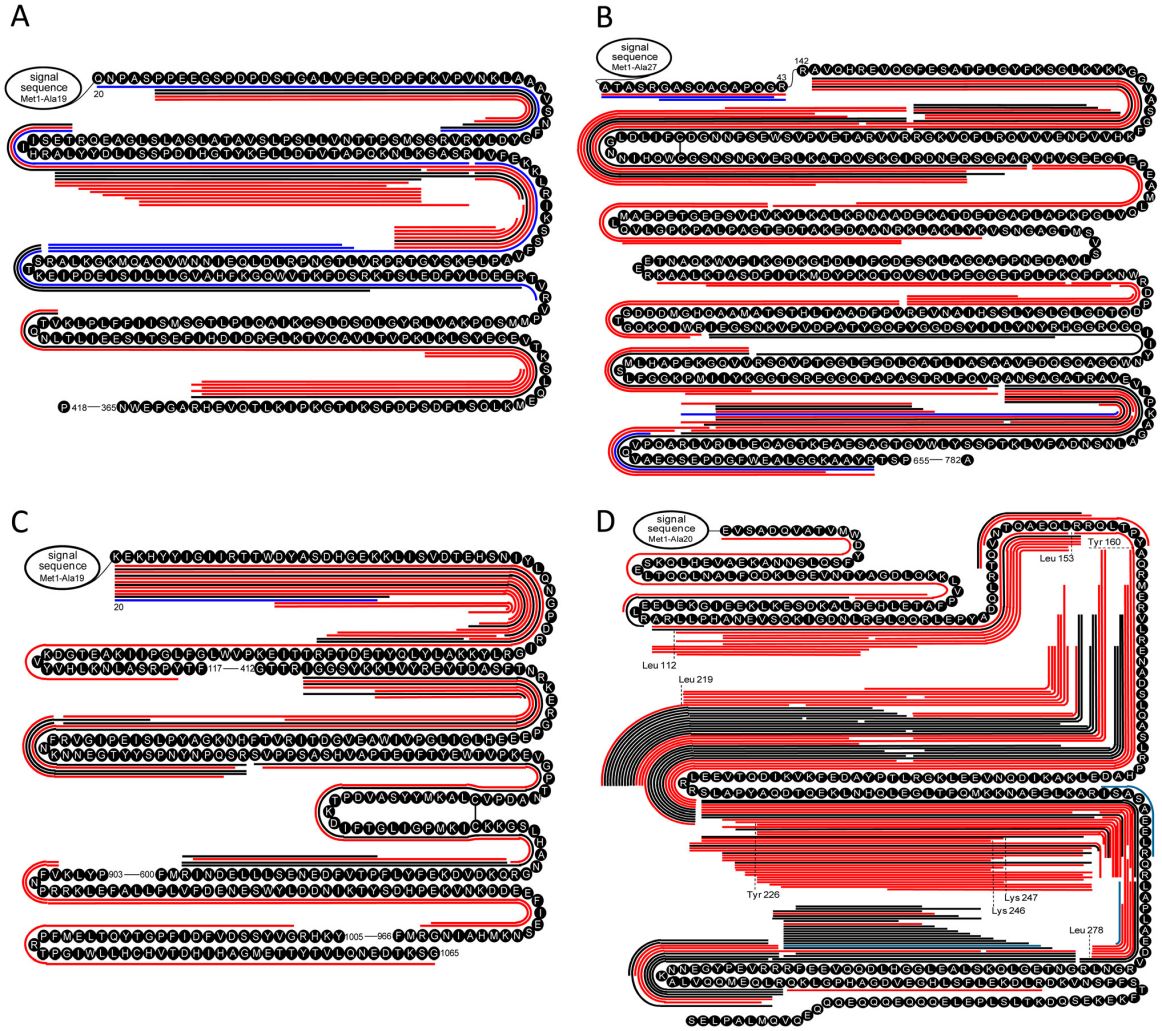
The differential degradation of other protein substrates observed for the tested BCP. (A) Pigment epithelium-derived factor; specific truncates from the N-terminal direction exist solely to produce the multiple BCP peptidome peptides. (B) Gelsolin; the sequence zone in the middle of the substrate sequences specifically produce the multiple BCP peptidome peptides. (C) Ceruloplasmin; the sequence zone toward the substrate C terminus specifically generated the multiple BCP peptidome peptides. (D) ApoA-IV; the cleavage P1-Leu, P1-Tyr, and Lys (labeled in the figure) specifically for the BCP exist to produce the multiple BCP peptidome peptides. The red, blue, and black lines along the amino acid sequences have the same significances as for Figure 7.

The BCP degradome active substrates also encompass proteins that have been previously investigated individually for cancer therapy, although the roles of some of these proteins in tumor biology have not been well elucidated. Figures 9A–C shows examples of the differential degradation in the BCP for tumor suppressors, e.g., ceruloplasmin [33], pigment epithelium-derived factor [34], and gelsolin [35], involved in angiogenesis, apoptosis, differentiation, Rac signaling, and mobility, etc. ApoA-IV, which has been reported having decreased blood concentration in cancer patients [36], is also high differentially degraded in the BCP examined (Figure 9D).

## Discussion

The bottom-up proteomics results obtained in this study, generally with a detection limit of ∼ng/mL [37], show that the blood proteome is not sensitive to the differences between the BCP and HP (Figure 1), and perhaps much more sensitive analytical technologies (e.g., with the detection limits of low level pg/mL) need to be explored for direct comparative blood proteomic studies, which is consistent with earlier insights [5]. In contrast, major changes in blood plasma degradome-peptidome are readily apparent for the BCP (see results shown in Figures 1, 2, 3, 4, 5, 6, 7, 8, 9). The degradomic information obtained in this work is also in agreement with characteristics known for breast cancer, suggesting that the broad top-down degradomic-peptidomic analysis has the potential to serve as an alternative strategy for discovery of the diagnostic and therapeutic targets for early detection of breast cancer.

However, it is recognized that this work was limited to 15 BCP and the matched control HP samples. The observations at the present time does not allow statistical evaluation of the differential degradomic-peptidomic signatures, and extensive investigations of the degradomes-peptidomes of a much larger BCP population need to be explored to validate whether every BCP displays all or part of the degradomic features described in this work. The BCP tested in this work are for stages I, II and III (maybe spread to nearby lymph nodes but not distant parts of the body), but not for late (advanced) stage IV breast cancer; the differences between the different stages of breast cancer were not studied in this work. Analysis of degradomic profiles for different stages of breast cancer, breast benign tumors and different molecular subgroups of breast cancers, and other types of cancers (e.g., uterine, liver, lung cancers, etc) and diseases (e.g., inflammation) are needed for statistical validation of which aberrances of the degradomic profile and/or which substrate sequence domains generating the peptidome peptides are specific to or unique for BCP before the BCP degradomic profile and/or individual peptidomic peptides reported in this work can be directly applied for the diagnostic purpose. We are now developing high-through measurement capabilities based on the accurate mass and time tags approach [38], [39] to support such extensive degradomic-peptidomic investigations, and the work reported herein is the impetus driving these efforts.

We also note that degradomic-peptidomic analysis of blood plasma is highly sensitive to a variety of conditions such as specimen collection and storage, peptidomic sample processing, sample analysis processes etc. For extensive comparative studies, standard operation procedures need to be established and executed to minimize the unnecessary variation that could affect the analytical results. The present study, albeit with the relatively small cohort size, used carefully selected patient and healthy control samples that were collected using the same laboratory protocol and processed in parallel. The data generated with our global peptidomic strategy are highly suggestive and provide a foundation for future efforts in this area.

## Methods

### Human blood plasma samples and collection procedure

Human blood plasma samples were collected from the BCP and HP at the University of Texas M. D. Anderson Cancer Center (Houston, TX), following the same strict laboratory protocol for each sample. Approval for conducting this study was obtained from the Institutional Review Boards of the University of Texas M. D. Anderson Cancer Center and the Pacific Northwest National Laboratory in accordance with federal regulations.

The 15 breast cancer subjects selected for this study are patients with ER/PR positive and Her2 negative invasive ductal carcinoma; 5 are stages I, 7 are stage II and 3 are stage III; 11 Caucasians, 3 Hispanics and 1 Asian/Pacific Islander; 8 premenopausal and 7 postmenopausal; aged from 30–78 years old (median age is 48 years old). The 15 control healthy subjects were carefully selected to have matched age, race, and menopausal status as the BCP. Blood samples collected in EDTA tubes were first centrifuged at 1,500–1,600 g for 15 min at 4°C, and plasma (upper phase) was transferred to another EDTA tube and centrifuged at 14,000 g for 15 min at 4°C to remove any remaining cellular material. The top 90% of plasma was then transferred into cryovials with approximately 0.5 ml plasma in each and stored frozen at −80°C until further use.

### Degradomic-peptidomic analysis

The method reported [13] was used for peptidomic analysis. Briefly, prior to analysis, 10 µL of Halt Protease Inhibitor Cocktail (Pierce Biotechnology, Inc., Rockford, IL) was added into every 1 mL of the plasma samples. The two pooled blood plasma samples of the BCP and the control HP from the matched BCP and HP were depleted of its 12 high-abundance proteins using a 12.7×79.0 mm IgY12 LC10 affinity chromatography (AC) column (Beckman Coulter, Fullerton, CA). The AC-depleted plasma samples were separated using a size exclusion chromatography (SEC) column (Superdex 200 10/300 GL, GE Healthcare, Piscataway, NJ), and species <20 kDa were collected (based on the calibration with various sizes of standard proteins) and denatured (1 h at 37°C with 8 M urea). The resulting solution was buffer exchanged to 50 mM NH_4_HCO_3_ (pH 8.0) in Amicon Ultra-15 filter (3 kDa nominal MW limit, Millipore, Billerica, MA). The plasma peptidomes of these samples were isolated without observable discrimination (Figure S3). The AC/SEC-isolated peptidome components (50 µg of each sample) were separated using the high-resolution liquid chromatography (HRLC). The HRLC was performed on a 1000 mm×0.100 mm i.d. fused silica capillary column containing 3-µm porous (300 Å size) C4-bonded silica particles (Sepax Technologies, Inc. Newark, DE) with the mobile phase gradient from A (acetonitrile/H_2_O/acetic acid, 10∶90∶0.2, v/v/v) to B (acetonitrile/isopropyl alcohol/H_2_O/acetic acid/trifluoroacetic acid, 60∶30∶10∶0.2∶0.1, v/v/v/v/v). Two HRLC separation runs were for each sample with gradients of 14,000 and 16000 min, respectively. The HRLC-separated components were detected using an LTQ-Orbitrap mass spectrometer (Thermo Fisher Scientific, San Jose, CA) under the conditions as follows: AGC targets of 1×10^6^ and 3×10^5^, respectively, for the Fourier transform mass spectrometry (FT MS) and FT tandem MS (MS/MS), 60K resolution for acquirement of the spectra, 400≤*m/z*≤2000 for a survey scan followed by FT MS/MS of the 5 most intense ions from the survey scan, 35% normalized collision energy employed for CID-FT MS/MS, a duration cycle of 30 s for the dynamic exclusion, 3 micro scans selected for FT MS and MS/MS measurements of the two HRLC-FT MS/MS analyses of each sample. The unique sequence tags (UStags) method [13] was used to process the FT MS/MS data using the ICR2LS developed in-house (http://ncrr.pnl.gov/software/), and only unique sequences from the IPI human sequence database (ipi.HUMAN.v3.39 downloaded from ftp://ftp.ebi.ac.uk/pub/databases/IPI/) were used for identification of the peptidome peptides. The identification false rate was estimated using a decoy dataset that was constructed by reversing each protein sequence in the human sequence database; no random hits were obtained using the UStags as the identification criteria. The identification of disulfide bond-containing peptides was completed with the method reported elsewhere [40]. The protein annotations and information from EBI (http://www.ebi.ac.uk/) and Swiss-Prot (http://ca.expasy.org) were used to describe the proteins (substrates) identified in this work.

Quantitation of peptidome peptides was achieved through extraction of the FT- measured precursors of the FT-MS/MS Spectra. ICR2LS was used to deisotope high-resolution spectra and produce lists of neutral masses for both precursor MS and MS/MS spectra. Functions were built specifically for this study to isolate MS spectra only and merge re-enumerated spectra to form a single continuous dataset. ICR2LS was also used to assign absolute intensity (abundance) values for the deisotoped masses using the area under the curve (peak area) as a measurement. To compile quantitative measurement of the identified peptides, the datasets for the two samples were processed for finding features (unique mass classes) based on the algorithm described previously [41]. Abundance of a feature is defined as sum of the individual spectrum abundances and log10 of this value is used as the abundance value without any further processing or normalization. Several heuristics principles were used for reducing quantitation information to unique peptide forms for presentation. In many reported cases multiple charge states for the detected features were observed, and the abundant, identical charge states were reported for those having the same abundance ratio between the various charge state distributions. In a few cases where the abundance ratio was not preserved for the different charge state distributions, manual inspection and correction of abundance value were applied and indicated in the data list (Table S2). The quantitative data include following two categories: 1) peptides were identified by the FT MS/MS-UStags for both the BCP and HP samples and 2) peptides were identified for either the BCP or HP sample. For situation 2, the unique mass classes between the two samples were matched according to the FT MS measured masses (5 ppm error tolerance) and the HRLC elution time (10% variance tolerance). When the peptides identified from one sample were not matched to species from the other sample, the species were declared not found in the other sample. The not-found species were assigned to have the abundance values of the background intensity scaled for summing over the features based on the same condition as the identified peptides correspondingly.

### Proteomic analysis

Conventional bottom-up approach was used for proteomic analysis of the BCP and HP plasma samples. The samples were depleted using the same procedure as used for preparation of the peptidomic samples. The IgY12 flow-through proteins were denatured and reduced in 50 mM ammonium bicarbonate buffer (pH 8.2), 8 M urea, 10 mM dithiothreitol for 1 h at 37°C, followed by alkylation in 40 mM iodoacetamide for 1 h at room temperature in the dark. The resultant protein mixtures were diluted 10 fold with 50 mM ammonium bicarbonate buffer (pH 8.2), and then sequencing grade modified porcine trypsin (Promega, Madison, WI) was added at a trypsin:protein ratio of 1∶50 (w/w). The samples were incubated overnight at 37°C for digestion. The tryptic digest samples were loaded on a 1-mL SPE C18 column (Sigma, St. Louis, MO) and cleaned with 4 mL of 0.1% trifluoroacetic acid/5% acetonitrile. The peptides were eluted from the SPE column with 1 mL of 0.1% trifluoroacetic acid/80% acetonitrile and lyophilized. The peptide samples were stored at −80°C for analysis.

The 300 µg of the prepared two tryptic digestion samples were individually reconstituted with 300 µL of 10 mM ammonium formate (pH 3.0)/25% acetonitrile and fractionated by strong cation exchange (SCX) chromatography on a Polysulfoethyl A 200 mm×2.1 mm column (PolyLC, Columbia, MD) that was preceded by a 10 mm×2.1 mm guard column. The separations were performed on an Agilent 1100 series HPLC system (Agilent, Palo Alto, CA) at a flow rate of 200 µL/min, and with mobile phases that consisted of 10 mM ammonium formate (pH 3.0)/25% acetonitrile (A) and 500 mM ammonium formate (pH 6.8)/25% acetonitrile (B). After loading 300 µL of each sample separately onto the column, the gradient was maintained at 100% A for 10 min. The peptides were separated using a gradient from 0 to 50% B over 40 min, 50–100% B over 10 min, and then held at 100% B for 10 min. A total of 30 fractions were collected for each sample, and each fraction was dried under vacuum. The fractions for each sample were dissolved in 30 µL of 25 mM NH_4_HCO_3_, and 5 µL was used for the capillary LC-MS/MS analyses.

Peptides LC-MS/MS analyses were carried out using a custom-built automated capillary LC system coupled online to an LTQ mass spectrometer (Thermo Scientific) *via* a nanoelectrospray ionization interface manufactured in-house. The LC separations were performed on 650 mm×0.075 mm capillary columns containing 3-*µ*m Jupiter C18 bonded particles (Phenomenex, Terrence, CA). The 5 *µ*L of each SCX fraction was loaded onto the column, and the mobile phase was held at 100% A (0.1% formic acid) for 20 min, followed by a gradient from 0 to 70% buffer B (0.1% formic acid in 90% acetonitrile) over 85 min with a flow rate ∼500 nL/min. Each full MS scan (*m*/*z* 400–2000) was followed by collision-induced MS/MS scans (normalized collision energy setting of 35%) for the 10 most abundant ions. The dynamic exclusion duration was set to 1 min, the heated capillary was maintained at 200°C, and the ESI voltage was held at 2.2 kV.

The LC-MS/MS raw data were converted into .dta files using Extract_MSn (version 3.0) in Bioworks Cluster 3.2 (Thermo Scientific), and the SEQUEST algorithm (version 27, revision 12) was used to independently search all the MS/MS spectra against the IPI database (version 3.39, released February 2008) with dynamic oxidation on methionine residues and static alkylation on cysteine residues. The false discovery rate (FDR) was estimated based on the reported decoy-database searching methodology [42], and filtering criteria were applied to limit the FDR at the peptide level to <1% as follows: for charge state +1 peptides, Xcorr >1.5 and ΔCn>0.05 for fully tryptic peptides and Xcorr >2.5 and ΔCn >0.16, or Xcorr >3.0 and ΔCn >0.10, or Xcorr >3.3 and ΔCn >0.05 for partially tryptic peptides; for charge state +2 peptides, Xcorr >1.5 and ΔCn >0.16, or Xcorr >1.7 and ΔCn >0.10, or Xcorr >2.2 and ΔCn >0.05 for fully tryptic peptides and Xcorr >4.1 and ΔCn >0.16 or Xcorr >4.3 and ΔCn >0.05 for partially tryptic peptides; for charge state +3 peptides, Xcorr >1.5 and ΔCn >0.16, or Xcorr >1.8 and ΔCn >0.10, or Xcorr >2.9 and ΔCn >0.05 for fully tryptic peptides and Xcorr >4.8 and ΔCn >0.16 or Xcorr >5.1 and ΔCn >0.05 for partially tryptic peptides. Identified proteins were grouped to a nonredundant protein list using ProteinProphet [43] software, after which one protein IPI number was randomly selected to represent each corresponding protein group that consisted of a number of database entries.

The identification details for each proteomic and peptidomic peptides were given in Data S1, and all spectra collected for the degradomic and proteomic analyses are accessible at http://www.ebi.ac.uk/pride/init.do.

Supplementary materials are included with this manuscript.

### Ethics Statement

We have obtained the written informed consent from all patients involved in this study.

## Supporting Information

Table S1 The proteome tryptic peptides identified from the pooled breast cancer patients (BCP) and control healthy persons (HP) blood plasma samples.
(9.08 MB DOC)

Table S2 The peptidome peptides identified from the pooled breast cancer patients (BCP) and control healthy persons (HP) blood plasma samples.
(0.95 MB DOC)

Table S3 The MMPs identified from the pooled breast cancer patients (BCP) and control healthy persons (HP) blood plasma proteomic samples.
(0.06 MB DOC)

Figure S1 The HC1-3 composed of protein complexes IαI, PαI and IαIL that function to stabilize the ECM. The red and blue numbers represent the different peptidome peptides observed for the BCP and the control HP, respectively.
(0.06 MB DOC)

Figure S2 The selective degradation of the BCP complement system convertases and MAC components would lead to the system dysfunction. The key convertases C3b, C4b, and factor B and MAC components C8 and C9 in the complement system that limits tumor growth are differentially degraded on their function domains for the BCP; the grey arrows represent the activation routes to be limited due to the excessive degradation [1]. The red and blue numbers represent the different peptides observed for the BCP and the control HP, respectively. 1 Markiewski MM, Lambris JD (2009) Is complement good or bad for cancer patients? A new perspective on an old dilemma. Trends Immun 30: 286–292.
(0.23 MB DOC)

Figure S3 The BCP and the control HP peptidome peptide molecular weight distributions. The BCP and HP peptidomes were isolated with use of size exclusion chromatography.
(0.08 MB DOC)

Data S1 (1.36 MB XLSX)
