# Supplementary material for: Blood Peptidome-Degradome Profile of Breast Cancer
Source: PLoS One. 2010 Oct 18;5(10):e13133. doi: 10.1371/journal.pone.0013133 (PMC2956627; doi:10.1371/journal.pone.0013133)
Supplement: Table S1 — The proteome tryptic peptides identified from the pooled breast cancer patients (BCP) and control healthy persons (HP) blood plasma samples. (9.08 MB DOC) [file pone.0013133.s001.doc]

Table S1. The proteome tryptic peptides identified from the pooled breast cancer patients (BCP) and control healthy persons (HP) blood plasma samples.*

| Tryptic peptide | Protein | Source |
| --- | --- | --- |
| K.WEFLQDGEKIHR.Q | [Protein ADP-ribosylarginine] hydrolase | BCP |
| R.DYLM*SKHFWGPVANWGLPIAAINDMK.K | 12 kDa protein | BCP |
| -.M*SSAAHLLCR.Q | 12 kDa protein | BCP |
| R.DQIAIAILILR.K | 12 kDa protein | BCP |
| K.YYHAQCNFFQDGLK.T | 126 kDa protein | BCP |
| R.IVTTASMVFR.G | 14 kDa protein | BCP |
| K.AAFDDAIAELDTLSEESYKDSTLIM*QLLR.D | 14-3-3 protein epsilon | BCP |
| K.ARAEAEDARQKAKDDDDSDIPTAQRKR.F | 145 kDa protein | BCP |
| K.SSTVGEIVNLM*SVDAQR.F | 166 kDa protein | BCP |
| R.KSDLFQDDLYPDTAGPEAALEAEEWFEGK.N | 18 kDa protein | BCP |
| R.RNSSESLR.D | 19 kDa protein | BCP |
| R.LAGGAGGGSRAGRASQAAAARRPR.A | 19 kDa protein | BCP |
| R.CLQKPNLTTDTK.D | 190 kDa protein | BCP |
| K.RAAPTGCPRLRGPLGVMPR.W | 20 kDa protein | BCP |
| K.RLSRAEVCSGCCEVCR.S | 20 kDa protein | BCP |
| R.VKAGKLSQLPGK.L | 21 kDa protein | BCP |
| R.DLDTWYKEQSAAM*SQEAASPATVQSR.Q | 24 kDa protein | BCP |
| K.IAELLCKRGASTDCGDLVM*TAR.R | 2-5A-dependent ribonuclease | BCP |
| R.TLTLPQNSKNRSPVASSRRRYAKR.R | 27 kDa protein | BCP |
| K.VEGEASALPTFWGPR.G | 28 kDa protein | BCP |
| K.NARGLAHVLIEVDETLDKFHISGNLK.F | 4 kDa protein | BCP |
| R.KSGELAKSGSRAPAGARR.E | 47 kDa protein | BCP |
| R.APRQRRNPVTVLSSSAPSDLIRRAR.K | 55 kDa protein | BCP |
| R.DQMRANVINEIM*STERHYIK.H | 56 kDa protein | BCP |
| K.DVLEVYGTGVASTR.H | 59 kDa protein | BCP |
| -.MGAGFSRNSR.Q | 6 kDa protein | BCP |
| R.SIYSLLMASSSNGIFDLFFSNCR.S | 60 kDa protein | BCP |
| K.VLPVLSAPQSVTDM*SASETAHGLRK.V | 7 kDa protein | BCP |
| R.KTIFCM*WPTVM*RVSMGNEWLEAQQMGAPGLGGRGGVCVR.D | 7 kDa protein | BCP |
| R.DLSLSSWQYIGEVR.K | 7 kDa protein | BCP |
| R.QAPTRPRAR.S | 7h3 protein (Fragment) | BCP |
| -.IKVECSNK.D | 8 kDa protein | BCP |
| R.VFEERRALLGKWM*GCCSGWFQIPGLKRFSCLCLPK.C | 8 kDa protein | BCP |
| R.RPPVLAKGDDPLPPR.A | A.T hook DNA-binding motif-containing protein 1 | BCP |
| K.ATGLFM*STNGNLEDLK.L | Absent in melanoma 1 protein | BCP |
| K.FKIRIEDPPRRKHMVFLGGAVLADIM*K.D | Actin-related protein 2 | BCP |
| R.KAKPAPSKTQARPVGVKIPTCK.I | Activator of 90 kDa heat shock protein ATPase homolog 1 | BCP |
| R.EPDLEKKYSK.Q | Adenylate cyclase type 5 | BCP |
| R.VVNLPLVSSTYDLMSSAYLSTK.D | Adipophilin | BCP |
| R.QAQVAEKLREQQAADAKKQAEESMVASMR.L | ADP-ribosylation factor GTPase-activating protein 2 | BCP |
| K.KLQNLEGKKREQAERLGM*GLVSR.S | ADP-ribosylation factor GTPase-activating protein 2 | BCP |
| R.QELHKLLDKDPDLPVVVVANK.Q | ADP-ribosylation factor-like protein 10 | BCP |
| K.LARVDSEGDFSENDDAAGDFR.S | A-kinase anchor protein 8 | BCP |
| K.FTVTAYDCGKK.R | Alcadein beta | BCP |
| -.M*VAEAGSM*PAASSVKKPFGLR.S | ANKRD26-like family C member 3 | BCP |
| K.EKM*EQLSRALKETEGGCPDTTFIEDAVHVLLKTR.R | Ankyrin repeat and IBR domain-containing protein 1 | BCP |
| K.STKLCGKTSCPRSNIFCNLLDKIVK.R | Ankyrin repeat and SOCS box protein 17 | BCP |
| R.DRSVPNLTEGSLHEPGRQSVTLRQNNLPAQSGSHAAEK.G | Ankyrin repeat domain-containing protein 27 | BCP |
| K.QVSQSEKTKALTTSSCVDVKSR.I | Ankyrin-3 | BCP |
| K.LSCAASGFTFSDAWMDWVR.Q | Anti-colorectal carcinoma heavy chain | BCP |
| R.ASILWLIGEYCEHVPRIAPDVLRK.M | AP-3 complex subunit beta-2 | BCP |
| R.HLVQPRGSRNGPGPWQGGRR.K | Apelin precursor | BCP |
| R.FKELFHPYAESLVSGIGR.H | Apolipoprotein A-V precursor | BCP |
| K.KLYIAELLLK.L | armadillo repeat containing 2 | BCP |
| R.AEGPVPMPQGSSLRTPAKPLPGPPPK.Q | Arrestin domain containing 1 variant (Fragment) | BCP |
| R.GSQAWQLRLCCPEPLM*TM*QFQGKLK.F | arylhydrocarbon receptor repressor | BCP |
| K.ICKLRSFFRKLNTR.L | Arylsulfatase I precursor | BCP |
| R.EAGVEM*GDEDDLSTPNEKLLGHLVKEK.Y | Aspartyl-tRNA synthetase, cytoplasmic | BCP |
| R.RSRIRSSVGPVR.S | ATPase, aminophospholipid transporter-like, Class I, type 8A, member 2 | BCP |
| K.MLTGDTTVTR.G | ATP-binding cassette sub-family A member 1 | BCP |
| R.RKLSIGIALIAGSKVLILDEPTSGM*DAISRRAIWDLLQR.Q | ATP-binding cassette sub-family A member 3 | BCP |
| K.KM*EKKNSGNFQKKAANMLQQSGSKNTGAK.K | ATP-dependent DNA helicase Q1 | BCP |
| -.M*ALFYVARYPGPDAAAAAGPEGAEAGAHGRARALLERLQSR.A | ATP-dependent RNA helicase DDX51 | BCP |
| R.GISRFIPPWLK.K | Band 4.1-like protein 2 | BCP |
| R.GDQEAVLQAILNNLEVK.K | Beta-1,3-galactosyl-O-glycosyl-glycoprotein beta-1,6-N- acetylglucosaminyltransferase 3 | BCP |
| K.SKATPAARASK.K | Beta-adrenergic receptor kinase 1 | BCP |
| K.HVSPAGAAVGIPLSEDEAK.V | Bifunctional purine biosynthesis protein PURH | BCP |
| K.QQLVLGTKVSLVENIHVLGK.E | Breast cancer type 2 susceptibility protein | BCP |
| K.LLRKLQKQEQARVAKEAK.K | Bromodomain adjacent to zinc finger domain protein 2B (hWALp4). Isoform 3 | BCP |
| K.DQDESDSDTEGEK.T | Bromodomain adjacent to zinc finger domain protein 2B (hWALp4). Isoform 3 | BCP |
| R.KGDNEELLLLCDGCDKGCHTYCHRPK.I | Bromodomain adjacent to zinc finger domain protein 2B (hWALp4). Isoform 3 | BCP |
| R.RQQAVLLKHQERERRR.Q | Bromodomain adjacent to zinc finger domain protein 2B (hWALp4). Isoform 3 | BCP |
| R.KVDEAPSEDLLVAMALSR.S | BTB (POZ) domain containing 12 | BCP |
| K.VIEKDISVQAIACRSAPVSKTLSSSDTELLVLNGSDPVAEVAIR.Q | C2CD2 protein | BCP |
| K.TEPEKKSQSTKPKSLPKQASDTGSNDAHNK.K | calpastatin isoform l | BCP |
| K.TPIKGPPVSSLSLASLNTGDNESVHSGK.R | CAMSAP1L1 protein | BCP |
| R.ISWSGM*TPPVEPPLDLSWLLGWGCGCWI.- | CDNA FLJ10915 fis, clone OVARC1000288, weakly similar to VACUOLAR AMINOPEPTIDASE I | BCP |
| K.ALYAGFQAAIGENNK.F | CDNA FLJ11142 fis, clone PLACE1006552 | BCP |
| -.MCVCFCRGGGM*GPCR.A | CDNA FLJ13544 fis, clone PLACE1006815 | BCP |
| R.TQSSPSVLSGPSET.- | CDNA FLJ16024 fis, clone BRAWH2018729, weakly similar to GLYCEROL-3- PHOSPHATE ACYLTRANSFERASE, MITOCHONDRIAL | BCP |
| R.ALLLSCVRVFQHGTDGSSGHSLSQGLARLRHDGPSPALTTTK.S | CDNA FLJ16283 fis, clone NT2RI3005928 | BCP |
| -.M*ECHLKTHYKMEYKCR.I | CDNA FLJ16555 fis, clone SPLEN2016781 | BCP |
| R.SSSGSVLVLAM*RVCSDKNK.N | CDNA FLJ25336 fis, clone TST00709 | BCP |
| K.WEDGLSSGGRGCSELR.G | CDNA FLJ25625 fis, clone STM02974 | BCP |
| K.ESPVKCDPLIMPRNR.E | CDNA FLJ26213 fis, clone ADG07906 | BCP |
| R.GRGGPGGMRGGRGGLM*DRGGPGGM*FR.G | CDNA FLJ31747 fis, clone NT2RI2007377, highly similar to RNA-BINDING PROTEIN EWS | BCP |
| R.SKIDTLMSQLKELEKQEQINSK.A | CDNA FLJ33807 fis, clone CTONG2001524 | BCP |
| R.KPERGGGGR.S | CDNA FLJ34873 fis, clone NT2NE2014950 | BCP |
| K.ENGQMAVSDGSVKGLLSVVRGWSR.G | CDNA FLJ35552 fis, clone SPLEN2004346 | BCP |
| R.RVNKMVISEEQMKL.Q | CDNA FLJ37098 fis, clone BRACE2019004 | BCP |
| -.M*SLACLRTFLGVDVLQDLAAVPPVMDLM*DR.L | CDNA FLJ37940 fis, clone CTONG2007779 | BCP |
| R.RKEAREGGKKEEGTKM*NRWIDGRKEKSR.R | CDNA FLJ38343 fis, clone FCBBF3028472 | BCP |
| -.MRGRARGVGRAGWTSGPAAGAVVRPLCR.G | CDNA FLJ40277 fis, clone TESTI2027239 | BCP |
| R.ASSPARSGR.A | CDNA FLJ41761 fis, clone IMR322003675 | BCP |
| K.KKKNWLGAVALASNPDTLR.G | CDNA FLJ42214 fis, clone THYMU2037208 | BCP |
| R.ELVPAFPTNAGLGR.R | CDNA FLJ42289 fis, clone TLIVE2006529 | BCP |
| K.LGTQKGPRNGQCHTRLGLVLWVMWPPLTAHVRWSQFR.A | CDNA FLJ43158 fis, clone ERLTF2000324 | BCP |
| R.KLNARRRQDKFWYCR.L | CDNA FLJ44602 fis, clone BLADE2008809 | BCP |
| -.M*LPKPGAGPGPGRGRVVGDRERR.G | CDNA FLJ45052 fis, clone BRAWH3022542 | BCP |
| R.GGFGEWEM*TEALRRRKR.S | CDNA FLJ45289 fis, clone BRHIP3002363 | BCP |
| R.KYMTVPARRSIPNVTKSTGVQTSPDLKK.C | CDNA FLJ45557 fis, clone BRTHA3002411 | BCP |
| K.VTQDELKEVFEDAAEIR.L | CDNA FLJ45706 fis, clone FEBRA2028457, highly similar to Nucleolin | BCP |
| K.FFKM*NPNTGELINWVALDR.E | CDNA FLJ45804 fis, clone NT2RI3005923, weakly similar to Cadherin- related tumor suppressor | BCP |
| K.RSQVKPVNYNKLATIDQGPHENPTAFLER.L | CDNA FLJ45949 fis, clone PLACE7007973 | BCP |
| K.GLCPVAFPPGRDKGPR.D | CDNA FLJ46230 fis, clone TESTI4014977 | BCP |
| K.DQVYLNSQVVSAAIGPKR.N | cDNA FLJ75360, highly similar to Homo sapiens egf-like module containing, mucin-like, hormone receptor-like 3 (EMR3), transcript variant 1, mRNA | BCP |
| R.ANKARDDYKTLVGSESPPLTVIRK.F | cDNA FLJ76812, highly similar to Homo sapiens IQ motif containing GTPase activating protein 2 (IQGAP2), mRNA | BCP |
| R.FEGCTPRKCGRGVTDVVITREEAER.I | CDNA: FLJ22222 fis, clone HRC01658 | BCP |
| R.WFSIQNNQLVYQK.K | Centaurin-beta-2 | BCP |
| K.WPSLATSACTPISSSKSNGLSK.D | Centaurin-gamma-like family member 5 | BCP |
| R.RVKNPNNLDEIKSFIASEVLKLFSLK.K | Centrosome-associated protein 350 | BCP |
| K.RQAISAEPTAFDIQDLSHVTLPFYPK.S | cGMP-dependent protein kinase 1, beta isozyme | BCP |
| R.RGAKAGVSAEPTTRTYDLNKPPEFSFEKARVR.K | cGMP-dependent protein kinase 2 | BCP |
| K.LIDAVGFSPLRILR.K | Chloride anion exchanger | BCP |
| R.ILGRNIGSHR.V | Chloride channel protein ClC-Kb | BCP |
| R.TQKNVKVIQQTM*RGWARCVLPPRREHR.R | Ciliary dynein heavy chain 11 | BCP |
| R.KLSKIATLLLAKNYILM*QAQALDEMR.R | Class B basic helix-loop-helix protein 4 | BCP |
| R.HVPARPRGAPLHLSGLRGRHAVREVR.Q | Class I MHC antigen HLA-B heavy chain (Fragment) | BCP |
| R.GDASKPLSDAQGK.E | Coiled-coil domain-containing protein 16 | BCP |
| K.LQRERDKLAKQERKEKEKKRTQRGER.K | Coiled-coil domain-containing protein 43 | BCP |
| R.TVTIENGM*QRK.A | Coiled-coil domain-containing protein 60 | BCP |
| R.DIITSIVNDLNIR.E | Collagen XXIX alpha 1 | BCP |
| R.KQSEAHTPASFLPCS.- | Conserved hypothetical protein | BCP |
| K.KNLPLPSFSPSLPLPAPVYFLYL.- | Conserved hypothetical protein | BCP |
| R.GSGATPVLM*TLPGSGCGCFAGSGFCGDGRATR.A | Conserved hypothetical protein | BCP |
| R.VPQNAMLESALGGYRSEEQSSLGRQPR.R | Conserved hypothetical protein | BCP |
| K.TDKYTEVLKTHGLLVCTQKSCSFLK.N | Conserved hypothetical protein | BCP |
| R.LQGQLQGGSR.E | Conserved oligomeric Golgi complex component 5 | BCP |
| K.SSAGCEGIGDFVELLGGTGLDPSK.M | Corticotropin-releasing factor-binding protein precursor | BCP |
| K.MTPLADLCYPFHGPAQMK.V | Corticotropin-releasing factor-binding protein precursor | BCP |
| K.M*TPLADLCYPFHGPAQMK.V | Corticotropin-releasing factor-binding protein precursor | BCP |
| K.EIEKESCGDPGTPLYGIREGDGFSNRDVLR.F | CUB and Sushi multiple domains 3 isoform 1 | BCP |
| K.GPKTIAEMRKQDLAKDTDPLK.L | Cyclin G-associated kinase | BCP |
| R.APSFAQKPK.V | Cyclin G-associated kinase | BCP |
| R.YGFLLLMK.H | Cytochrome P450 2A13 | BCP |
| K.ESLRLHPPVPVISRHVTQDIVLPDGR.V | Cytochrome P450 4F2 | BCP |
| R.RNIALRNDSESSGVLYSR.A | Cytokine receptor-like factor 3 | BCP |
| K.HKKRKPSSAKAHLDCDVSLGTVADESQR.A | DBF4-type zinc finger-containing protein 2 | BCP |
| R.VGEAAHALGNTGHEIGR.Q | Dermokine gamma-1 | BCP |
| K.YEEIDCLINDEHTIKGRR.E | D-glucuronyl C5-epimerase | BCP |
| R.VLVCGGDGTVGWVLDAVDDMKIK.G | Diacylglycerol kinase epsilon | BCP |
| R.FQM*PDQGM*TSADDFFQGTK.A | Dihydropyrimidinase-like 2 | BCP |
| R.VLEDNSALDK.M | Dipeptidyl peptidase 4 | BCP |
| R.YKSRDIWAK.I | DKFZP434B0335 protein | BCP |
| K.VESDLLDQEEM*VDKPDIGSYERHQIER.R | DmX-like protein 2 | BCP |
| K.LLVDSNNPKIKDILAKSK.G | DNA-directed RNA polymerase II subunit RPB1 | BCP |
| K.LVQLYGVCTKQRPIFIITEYMANGCLPCTVAGMRK.Q | Dominant-negative kinase-deficient Brutons tyrosine kinase isoform 3 | BCP |
| K.LQKYINNPDFVPEKVEKVSKACK.S | Dynein heavy chain domain containing protein | BCP |
| K.EVIMKESVAPTEHLR.L | dynein, axonemal, heavy chain 7 | BCP |
| R.TRAAESAEIEPR.N | E3 ubiquitin-protein ligase RAD18 | BCP |
| K.EMEEQVEIKRLRTENRLLKQRIETLEKHK.C | Ecotropic viral integration site 5 protein homolog | BCP |
| R.RNKRSKSNEGADGPVKNKK.K | Ectodysplasin-A | BCP |
| K.QKEM*DEAATAEERGR.L | EIF4G1 variant protein | BCP |
| R.CLKDTDCPGIKK.C | Elafin precursor | BCP |
| R.LLVSRSLGGAVGSVASGAR.A | Ells2 (Fragment) | BCP |
| R.VCVEKWNLLNSSRLHLPRASAVALEVQRLNALDLEKKIGK.S | Elongation factor 2 kinase | BCP |
| K.GKDGVGAVM*DSMELERQR.G | Elongation factor G 1, mitochondrial precursor | BCP |
| R.HVAGLWAGLR.E | EMILIN-1 precursor | BCP |
| K.KGRRKGRQAASGHCRPRKVKADIPSLEPEGTSAS.- | Endonuclease VIII-like 1 (EC 3.2.2.-) (EC 4.2.99.18) (Nei-like 1) (DNA glycosylase/AP lyase Neil1) (DNA-(apurinic or apyrimidinic site) lyase Neil1) (NEH1) (FPG1). Isoform 2 | BCP |
| K.EVIKEKGLEHVTVDDLVAEITPKGR.A | Enhancer of yellow 2 transcription factor homolog | BCP |
| K.KHGEAEK.K | erythropoietin 4 immediate early response | BCP |
| K.SKGRLVNVSSMGGGAPM*ER.L | Estradiol 17-beta-dehydrogenase 2 | BCP |
| R.YIVNGSHEANKLQDMLDGFIKK.F | Eukaryotic translation initiation factor 5 | BCP |
| K.M*PRLIAKVEGKGNGIKTVIVNMVDVAK.A | Eukaryotic translation initiation factor 5 | BCP |
| R.HADILAAVETR.L | F-box only protein 28 | BCP |
| R.WKVESLPGAHGTDFPDPK.V | F-box only protein 6 | BCP |
| R.GERRLEASMGRPEVSM*M*SSSASKNLKFK.I | FERM and PDZ domain-containing protein 3 | BCP |
| -.M*EDGGQREEETR.R | FKSG88 | BCP |
| R.QQQDLM*PQCR.D | fms-related tyrosine kinase 4 isoform 1 | BCP |
| K.NVHLFATPLAASLEEVAPGAR.H | fms-related tyrosine kinase 4 isoform 1 | BCP |
| K.LPDNFAFDVSHECAWGCRGSFIPARYNSSR.A | Functional smad suppressing element 18 | BCP |
| R.EFDGIGAVSGGGATSR.L | galactosylceramidase isoform a precursor | BCP |
| K.NPFLSLAACVMPSRLK.A | GCN1-like protein 1 | BCP |
| R.IHRIFEQGKK.S | Glutamate receptor, metabotropic 8 | BCP |
| K.IRVDILENQTM*DNHM*QLGMICYNPEFEK.L | glutathione S-transferase M1 isoform 2 | BCP |
| K.GQFPNLCRLCAGTGENK.C | Growth-inhibiting protein 12 | BCP |
| R.RAVMVSNILLINKVNEK.S | Guanine deaminase | BCP |
| R.LSSGFQSSTLLTSVRLQFLAGCFGLGTVGHTGGK.G | guanine nucleotide exchange factor p532 | BCP |
| K.FFAATSIVLFLNKKDLFEEK.I | Guanine nucleotide-binding protein G(t) subunit alpha-2 | BCP |
| R.RPEYLEERRIK.E | Heat shock protein 90Ae | BCP |
| R.TQLYKAERDRADRLNFMLLPRLVVKSLK.E | Heat-stable enterotoxin receptor precursor | BCP |
| K.IEDAQVQDTGR.Y | Hemicentin 1 | BCP |
| K.WRMLTDLRAVNAIIQPMGPLQSGLPSPAM*IPK.D | HERV-K_5q13.3 provirus ancestral Pol protein | BCP |
| R.ITKLCGNDPDK.I | HERV-K_5q33.3 provirus ancestral Pol protein | BCP |
| K.KLGEMWSEQSAKDK.Q | High mobility group protein B2 | BCP |
| R.FRSLLDSDHHPSEIAESHR.F | Histidine ammonia-lyase | BCP |
| K.EPRDPDQLYSTLKSILQQVK.S | Histone acetyltransferase PCAF | BCP |
| R.GLAGGRVLALLEER.T | Histone deacetylase 10 variant (Fragment) | BCP |
| R.RPAETLGDSSPSSSSTPASGCSSPNDSEHGPNPILGSEALLGQR.L | histone deacetylase 7A isoform c | BCP |
| R.RRGSSGAGGRGRTCSR.T | Histone H2A-Bbd type 2/3 | BCP |
| K.TPSSDVLVFDYTKHPSKPDPSGECNPDLR.L | Histone-binding protein RBBP4 | BCP |
| R.QIADIILPMLAK.Q | Huntingtin | BCP |
| K.LLYQVQASKQEITAVLEMKSRLQMR.R | hypothetical protein | BCP |
| R.ATQELQCPRGPSR.L | Hypothetical protein | BCP |
| K.ISGGGGGGK.G | hypothetical protein | BCP |
| R.LLPSANHSDSAELDGAEVAFAK.G | hypothetical protein | BCP |
| R.VRM*WPPLGGHYSACHHSQALGM*RNTLKISRNK.S | Hypothetical protein | BCP |
| R.ERIHNFLSELLK.E | hypothetical protein LOC129881 | BCP |
| R.ICSQRVNSKSFK.G | hypothetical protein LOC254268 | BCP |
| K.SM*WDDIRLHLR.R | hypothetical protein LOC285600 | BCP |
| R.LKKTRKYDIITTPAER.V | hypothetical protein LOC400451 | BCP |
| R.GSRPGPGWAGSPKTEKEKGSSWRNWPGEAK.A | hypothetical protein LOC54540 | BCP |
| K.SRSGASGLRRAFSWLRGKR.R | hypothetical protein LOC57648 | BCP |
| R.RDSDSFLNIFPEKQVTK.A | hypothetical protein LOC643314 | BCP |
| R.QGSQPAPVRK.N | hypothetical protein LOC84182 | BCP |
| -.MKRKVVNTHKLRLSPNEEAFILK.E | hypothetical protein LOC85459 | BCP |
| R.AVHEAASPSQTVQR.A | Ig epsilon chain C region | BCP |
| -.DIQM*TQSPSTLSVSVGDR.V | Ig kappa chain V-I region Ka | BCP |
| -.DFMLTQPHSVSESPGK.T | Ig lambda chain V-VI region AR | BCP |
| R.FLTNFPFSTAVK.L | Inactive serine protease 35 precursor | BCP |
| K.M*M*GNMTSPR.S | Influenza virus NS1A-binding protein | BCP |
| K.QRMHFALKSLLQEAQNNLKIFK.N | Inositol-pentakisphosphate 2-kinase | BCP |
| R.AEIM*RSFSLSTNLQESLRSK.E | Interferon alpha-2 precursor | BCP |
| K.TSLVSLLLLLSLEAIVK.A | Interleukin-17A precursor | BCP |
| K.SFICGQNETPM*ETISLPPPIIQPMEVQM*K.V | Isoform 1 of AF4/FMR2 family member 2 | BCP |
| K.RGSWRTGKTEARNEDEMYK.I | Isoform 1 of Afadin- and alpha-actinin-binding protein | BCP |
| R.TGVSVVMGIPSVR.R | Isoform 1 of Alpha-1,3-mannosyl-glycoprotein 4-beta-N-acetylglucosaminyltransferase B | BCP |
| K.TKEGVVHGVATVAEK.T | Isoform 1 of Alpha-synuclein | BCP |
| R.TCHALQEDEKLVK.K | Isoform 1 of AMY-1-associating protein expressed in testis 1 | BCP |
| R.DTPLHVAAQRGLDEHARLYLGR.G | Isoform 1 of Ankyrin repeat and SOCS box-containing protein 18 | BCP |
| R.NFKEERDKIKKESEKSFR.E | Isoform 1 of Ankyrin repeat domain-containing protein 12 | BCP |
| K.RDALAASHEPPPEPEEITGPVDEETFLK.A | Isoform 1 of Ankyrin repeat domain-containing protein 2 | BCP |
| K.TVALLLEAGADPSLR.N | Isoform 1 of Ankyrin repeat domain-containing protein 29 | BCP |
| K.KDKKDFAREMTKMKM*FKSQLTLM*EHNYLIEYQK.E | Isoform 1 of Ankyrin repeat domain-containing protein 53 | BCP |
| R.FIVVVGGGSAGVEM*AAEIK.T | Isoform 1 of Apoptosis-inducing factor 2 | BCP |
| K.NPHKPTGKVLGLAHSTTGPQESK.G | Isoform 1 of AT-rich interactive domain-containing protein 5B | BCP |
| R.ASLGTGTASPR.T | Isoform 1 of Bridging integrator 2 | BCP |
| R.ASGGLKKGDFLKK.G | Isoform 1 of Bullous pemphigoid antigen 1, isoforms 1/2/3/4/5/8 (Fragment) | BCP |
| K.SEGLAVLAVLIEM*GSFNPSYDK.I | Isoform 1 of Carbonic anhydrase 12 precursor | BCP |
| K.EPARVFSM*IIDASGESGLTQLLMTEVMK.L | Isoform 1 of Caspase recruitment domain-containing protein 9 | BCP |
| R.SLLKNLGHWLGM*ITLAKNKPILHTDLDVK.S | Isoform 1 of CCR4-NOT transcription complex subunit 1 | BCP |
| K.AFLNAVMRWGMPPQDAFTTQWLVR.D | Isoform 1 of Chromodomain-helicase-DNA-binding protein 3 | BCP |
| K.CCNHPYLFPVAAMEAPKM*PNGM*YDGSALIR.A | Isoform 1 of Chromodomain-helicase-DNA-binding protein 4 | BCP |
| R.QNLSQFEAQARK.R | Isoform 1 of Cleavage and polyadenylation specificity factor subunit 7 | BCP |
| -.MEPRMESCLAQVLQKDVGK.R | Isoform 1 of CLIP-associating protein 1 | BCP |
| K.MKLLNLYIKRAQTTNSNSSSSSDVSTHS.- | Isoform 1 of CLIP-associating protein 1 | BCP |
| R.ASVHASFYSADLVCKLYSQQLM*RQYHK.E | Isoform 1 of Coiled-coil domain-containing protein 108 | BCP |
| K.DMMLTQAPSSVVRSRNSR.N | Isoform 1 of Coiled-coil domain-containing protein 108 | BCP |
| K.YLLYEDEKDFKDYVNLGPLGVKLMSVESK.K | Isoform 1 of Coiled-coil domain-containing protein 83 | BCP |
| K.GHRGFTGLQGLPGPPGPSGDQGASGPAGPSGPR.G | Isoform 1 of Collagen alpha-1(II) chain precursor | BCP |
| K.SKDCKYPQKPHKSRSRSSSR.S | Isoform 1 of Cyclin-L2 | BCP |
| R.SLAMSRLDEVISKYAMLQDKSEEGERKKR.R | Isoform 1 of Death domain-associated protein 6 | BCP |
| R.SHCKNIKQKISQWEGRANGISNPEK.W | Isoform 1 of DENN domain-containing protein 2C | BCP |
| R.ARATGPAAPPPR.L | Isoform 1 of Desmuslin | BCP |
| R.MLEEILKTRFMVKQSMKAYK.Q | Isoform 1 of DNA polymerase zeta catalytic subunit | BCP |
| K.RAAGDGGSGPPEK.K | Isoform 1 of E3 ubiquitin-protein ligase BRE1B | BCP |
| K.LSVISVEDPPQR.T | Isoform 1 of Electron transfer flavoprotein subunit beta | BCP |
| K.AIASNSKIYFGKDIPNM*FMDSAGSVSKQFEGLADK.L | Isoform 1 of Erlin-2 precursor | BCP |
| K.AKADAECYTAMKIAEANKLKLTPEYLQLM*KYK.A | Isoform 1 of Erlin-2 precursor | BCP |
| R.AGASPATSKTLAFSIERIMAK.T | Isoform 1 of Fez family zinc finger protein 2 | BCP |
| K.M*LKDDATEKDLSDLVSEMEM*MKM*IGK.H | Isoform 1 of Fibroblast growth factor receptor 2 precursor | BCP |
| K.NKVLRAYNILIGELDCSKEK.G | Isoform 1 of Gametogenetin-binding protein 2 | BCP |
| R.LTTGTPASPR.R | Isoform 1 of GAS2-like protein 1 | BCP |
| R.DLHTTAFKVDAGKLHYHR.K | Isoform 1 of General transcription factor 3C polypeptide 1 | BCP |
| K.GVYVKNIRPAGPGDLGGLKPYDR.L | Isoform 1 of Glutamate receptor-interacting protein 1 | BCP |
| K.CTHSSSIEDLALGR.C | Isoform 1 of Glycoprotein-N-acetylgalactosamine 3-beta-galactosyltransferase 1 | BCP |
| K.CPDSSNSGVKGCLLSGFR.G | Isoform 1 of Grainyhead-like protein 3 homolog | BCP |
| R.VSELFGGCCRPGGGPAVGGTLKARGAGSSSGCGGPKGKKKNGRNR.G | Isoform 1 of GTP-binding protein 2 | BCP |
| K.SVSNSILECYTPAQTISTEFAVK.L | Isoform 1 of Hepatocyte growth factor receptor precursor | BCP |
| R.GPPGGNRGGFQNRGGGSGGGGNYRGGFNR.S | Isoform 1 of Heterogeneous nuclear ribonucleoprotein U-like protein 1 | BCP |
| R.RRLNSSSGSGSGSSGSSVSSPSWAGRLR.G | Isoform 1 of Inositol-trisphosphate 3-kinase B | BCP |
| K.QRTIPWRSKSPEILSSTKAGCTGEEQWR.G | Isoform 1 of IQ domain-containing protein C | BCP |
| -.M*AAAGAGRLRR.V | Isoform 1 of Iron-sulfur cluster assembly enzyme ISCU, mitochondrial precursor | BCP |
| R.ESLVRAGKKIAYQGR.V | Isoform 1 of JmjC domain-containing protein 3 | BCP |
| K.RQLRQELESLMKEQDLLETKLRSYEREK.T | Isoform 1 of Leucine zipper putative tumor suppressor 1 | BCP |
| K.RKKLEEATAAR.A | Isoform 1 of Macoilin | BCP |
| K.FHLVPPVSVPRYQR.F | Isoform 1 of Membrane-associated phosphatidylinositol transfer protein 3 | BCP |
| -.MPEEMDKPLISLHLVDSDSSLAKVPDEAPK.V | Isoform 1 of Metalloreductase STEAP3 | BCP |
| R.NYAHALDGLYRVAR.E | Isoform 1 of Mitochondrial dicarboxylate carrier | BCP |
| R.KLSM*GSDDAAYTQALLVHQKAR.M | Isoform 1 of Mitogen-activated protein kinase kinase kinase 7-interacting protein 2 | BCP |
| R.CEDRCEQGTYGNDCHQR.C | Isoform 1 of Multiple epidermal growth factor-like domains 10 precursor | BCP |
| K.CYHVSGACLCEAGFAGER.C | Isoform 1 of Multiple epidermal growth factor-like domains 10 precursor | BCP |
| K.HQRQLSLPLTQSKSSPKR.G | Isoform 1 of Myotubularin-related protein 12 | BCP |
| K.LQHYTDSYLGFLPWEKKK.Y | Isoform 1 of N-acetylglucosamine-1-phosphotransferase subunits alpha/beta precursor | BCP |
| K.RNENFPK.D | Isoform 1 of NEDD4-binding protein 2 | BCP |
| K.RWRKLVSKTQLEMNLPLMIK.K | Isoform 1 of Nesprin-2 | BCP |
| R.TRTCTNPAPLNGGAFCEGQAFQK.T | Isoform 1 of Netrin receptor UNC5B precursor | BCP |
| R.SNSIPTHEAAFELYSGSQMGSTLSLAERPK.G | Isoform 1 of Neuron navigator 1 | BCP |
| R.NSSPGEASLLEKETTR.F | Isoform 1 of Nucleolar protein 8 | BCP |
| K.GSKEESGASASTSPSEK.T | Isoform 1 of OTU domain-containing protein 7A | BCP |
| R.KHNKIPRSELLNELMDSAK.V | Isoform 1 of Pentatricopeptide repeat-containing protein 3, mitochondrial precursor | BCP |
| R.LEPTERPLQIVYDYLSRLGFDDPVR.I | Isoform 1 of PH domain leucine-rich repeat protein phosphatase-like | BCP |
| R.M*YPFQIHSIALSTFASLIGPFGGFFASGFK.R | Isoform 1 of Phosphatidate cytidylyltransferase 2 | BCP |
| K.FNVANGGPAPDVVSDK.I | Isoform 1 of Phosphoglucomutase-like protein 5 | BCP |
| R.LMKRIAEM*TDIKPILR.K | Isoform 1 of Presequence protease, mitochondrial precursor | BCP |
| K.NYTDEAIETDDLTIK.L | Isoform 1 of Proteasome subunit alpha type-7 | BCP |
| K.CLIIGAGPCGLRTAIDLSLLGAK.V | Isoform 1 of Protein MICAL-3 | BCP |
| R.VSYSLIASDLESRTLSSYVSVSAQSGVVFAQR.A | Isoform 1 of Protocadherin gamma B7 precursor | BCP |
| K.EVRM*AEM*VSMGVGNKAFR.D | Isoform 1 of Puratrophin-1 | BCP |
| R.KKSTRGFYFAKLYYEAKEYDLAKK.Y | Isoform 1 of RANBP2-like and GRIP domain-containing protein 4 | BCP |
| K.TTEKENLGPRMDPPLGEPEGSLGWVLPNTAM*K.K | Isoform 1 of Ras-related GTP-binding protein B | BCP |
| -.MSTGGDFGNPLRKFKLVFLGEQSVGKTSLITR.F | Isoform 1 of Ras-related protein Rab-6A | BCP |
| R.EQPSEGATELRQVAGDAPVEQATAETASPVHR.E | Isoform 1 of RAS-responsive element-binding protein 1 | BCP |
| R.RHRSRASAMSSPSSPGLGRVAPR.G | Isoform 1 of Retinitis pigmentosa 1-like 1 protein | BCP |
| R.DIFAQQRESKETAPGGTESQSLRTNENK.Y | Isoform 1 of Rho GTPase-activating protein 18 | BCP |
| K.GATIKRIQQQTHTYIVTPSR.D | Isoform 1 of RNA-binding protein MEX3B | BCP |
| R.GGCPRGASCTFAHSQEELEKFRKMNK.R | Isoform 1 of Roquin | BCP |
| R.GQQRQGGPPAADAAGPDDMEPKKGTGAPK.E | Isoform 1 of Sarcospan | BCP |
| R.ELRSIIETMKDQQKREIK.Q | Isoform 1 of SH3 domain-containing kinase-binding protein 1 | BCP |
| R.MHTAVKLNGVVLNKSQDAQLVLLNMPGPPK.N | Isoform 1 of Solute carrier family 12 member 7 | BCP |
| R.GQPRVVVIGAGLAGLAAAK.A | Isoform 1 of Spermine oxidase | BCP |
| P.YLVVVIGLENVLVLTK.S | Isoform 1 of Sterol regulatory element-binding protein cleavage-activating protein | BCP |
| K.LSPSDRQVPHSSR.E | Isoform 1 of Storkhead-box protein 2 | BCP |
| R.NPPTTIGMMTLK.A | Isoform 1 of Telomeric repeat-binding factor 2 | BCP |
| R.LSM*WDRPDDLIGR.A | Isoform 1 of Transcription elongation regulator 1 | BCP |
| R.SLLGLGGLDAECGRPLFATYSGLWRK.C | Isoform 1 of Transmembrane protein 178 precursor | BCP |
| K.EVDPAKCVLQGEDLHRAR.E | Isoform 1 of Tripartite motif-containing protein 45 | BCP |
| K.LAEQCGGLQGFLIFR.S | Isoform 1 of Tubulin alpha chain-like 3 | BCP |
| R.ATHYPPYAGGGGYVM*SRATVRR.L | Isoform 1 of UDP-GlcNAc:betaGal beta-1,3-N-acetylglucosaminyltransferase 4 | BCP |
| R.M*TESSGLVSTPVPR.A | Isoform 1 of Uncharacterized protein C1orf170 | BCP |
| R.GRGALARPVLTKEQLDNQLDAYM*SK.T | Isoform 1 of Uncharacterized protein C1orf77 | BCP |
| K.KM*VIMESGESAASHEAKK.T | Isoform 1 of Uncharacterized protein C4orf21 | BCP |
| K.DTEAHISEPEDLGKIRSPPPDHVEVETAR.E | Isoform 1 of Uncharacterized protein C4orf21 | BCP |
| R.ILKNAGVGFSITSSTFSSSTK.K | Isoform 1 of Uncharacterized protein KIAA1958 | BCP |
| K.MNIKHLVDPIDDLFLAAKKIPGISSTGVGDGGNELGM*GK.V | Isoform 1 of UPF0317 protein C14orf159, mitochondrial precursor | BCP |
| -.MDNSAQKNERTGKHPR.R | Isoform 1 of UPF0490 protein C1orf201 | BCP |
| R.M*LDNRKM*GDMPEINGKLVKSIIR.V | Isoform 1 of Upstream-binding protein 1 | BCP |
| R.TM*ATAALAASPAPVSNLQGPYLASGDQPLER.A | Isoform 1 of Voltage-dependent L-type calcium channel subunit beta-1 | BCP |
| K.KSLENHLAKTHRSLLLGKK.H | Isoform 1 of Zinc finger and BTB domain-containing protein 41 | BCP |
| K.RHM*LIHEPFKK.Y | Isoform 1 of Zinc finger protein 341 | BCP |
| K.QTFVGHQQR.I | Isoform 1 of Zinc finger protein 549 | BCP |
| K.TYCDASGLSR.H | Isoform 1 of Zinc finger protein 57 homolog | BCP |
| K.FHGDMVKTEALERKDTGRQSSRQVAK.L | Isoform 1 of Zinc finger protein 64, isoforms 1 and 2 | BCP |
| K.EELLDDNNFISDK.E | Isoform 1 of Zinc finger protein 644 | BCP |
| R.M*ITTSGPTSEKPTR.S | Isoform 1A of Mitogen-activated protein kinase kinase kinase 7 | BCP |
| R.RERNYSWM*DIITICKDK.L | Isoform 2 of 1,2-dihydroxy-3-keto-5-methylthiopentene dioxygenase | BCP |
| R.QVVEVTVGLQLIQLINVDEVNQIVTTNVR.L | Isoform 2 of Acetylcholine receptor subunit alpha precursor | BCP |
| R.EEEVQQGQQDLEK.E | Isoform 2 of A-kinase anchor protein 13 | BCP |
| R.QLQQELADTLKKQSM*SEASLEVTSR.Y | Isoform 2 of Ankyrin repeat domain-containing protein 26 | BCP |
| K.KDRRALHWAAYMGHLDVVALLINHGAEVTCKDK.K | Isoform 2 of Ankyrin repeat domain-containing protein 44 | BCP |
| R.VHAKDPDAANSPIR.Y | Isoform 2 of Cadherin-11 precursor | BCP |
| K.IKAIARKTKEVADRVR.K | Isoform 2 of DC-STAMP domain-containing protein 2 | BCP |
| R.YPPWLLLLK.R | Isoform 2 of DENN domain-containing protein 2A | BCP |
| K.RLLASTASSSSPASVM*VAK.E | Isoform 2 of Dual 3',5'-cyclic-AMP and -GMP phosphodiesterase 11A | BCP |
| R.FITTICAIIGGTFTVAGILDSCIFT.A | Isoform 2 of Endoplasmic reticulum-Golgi intermediate compartment protein 1 | BCP |
| K.KTLNPVFDQSFDFSVSLPEVQRR.T | Isoform 2 of Extended synaptotagmin-2 | BCP |
| K.CMSECPGGYYADATGR.C | Isoform 2 of Extracellular matrix protein FRAS1 precursor | BCP |
| R.GCVGNTNIWKTGPLFKR.V | Isoform 2 of Glutamate [NMDA] receptor subunit zeta-1 precursor | BCP |
| R.KVSDVKVPLLPSHK.R | Isoform 2 of Grainyhead-like protein 1 homolog | BCP |
| R.SVLTAFDLSVFPLM*CDLGGDFFK.D | Isoform 2 of Hydroxyindole O-methyltransferase | BCP |
| R.KVVFAVPK.T | Isoform 2 of Membrane-associated guanylate kinase, WW and PDZ domain-containing protein 1 | BCP |
| K.KCKDPKPR.V | Isoform 2 of MKL/myocardin-like protein 2 | BCP |
| R.ELAAFRTK.V | Isoform 2 of Nuclear mitotic apparatus protein 1 | BCP |
| K.SRGPRGEGGAM*SENIWSTLR.L | Isoform 2 of Peroxisomal targeting signal 1 receptor | BCP |
| R.AM*LGM*KLSLPYGLK.G | Isoform 2 of PHD finger protein 1 | BCP |
| K.KQITDHTVNLRSRNGWPGAVAHACNPSTLGGQGGR.I | Isoform 2 of Plakophilin-2 | BCP |
| R.ALDLFSDNAPPPELLEIINEDIAKR.T | Isoform 2 of Protein disulfide-isomerase A6 precursor | BCP |
| R.DEIEFMWRFAR.A | Isoform 2 of Protein FAM82A | BCP |
| R.DSGQNAFTTCSLPEDLPFKLEK.S | Isoform 2 of Protocadherin gamma A2 precursor | BCP |
| K.YPQVRAYNFPPKKAIGNK.D | Isoform 2 of PX domain-containing protein SNX29 | BCP |
| K.YGLLNVTKIAENGKKVR.K | Isoform 2 of Rho GTPase-activating protein 12 | BCP |
| R.GQRWHQGSGYFGR.T | Isoform 2 of Ryanodine receptor 3 | BCP |
| K.ANKTLLPLHCGSK.R | Isoform 2 of Secretory phospholipase A2 receptor precursor | BCP |
| K.MSFEILRGSDGEDSASGGKTPAPGPEAASGEWELLRLDSSKK.K | Isoform 2 of Separin | BCP |
| R.SWTAFSTSSSSPGTPLSPGNPFSPGTPISPGPGIM*RR.N | Isoform 2 of Serine/threonine-protein kinase WNK4 | BCP |
| R.CDQLQAEQKGLTEVTQSLKM*ENEEFK.K | Isoform 2 of Tax1-binding protein 1 | BCP |
| R.LM*ALALAER.A | Isoform 2 of TC10/CDC42 GTPase-activating protein | BCP |
| R.LM*RPNGVQAVVRGILEGAGGKK.- | Isoform 2 of Transmembrane and coiled-coil domain-containing protein 7 | BCP |
| R.AREQAEAEVASLNRR.I | Isoform 2 of Tropomyosin alpha-3 chain | BCP |
| K.LEEAEKAADESER.G | Isoform 2 of Tropomyosin alpha-3 chain | BCP |
| K.HIAEEADRKYEEVAR.K | Isoform 2 of Tropomyosin alpha-3 chain | BCP |
| R.RGGADVNIRHSGRDDTSRYDER.D | Isoform 2 of U1 small nuclear ribonucleoprotein 70 kDa | BCP |
| R.KPQEM*IDIWSQLQSAHLK.C | Isoform 2 of UDP-GlcNAc:betaGal beta-1,3-N-acetylglucosaminyltransferase 2 | BCP |
| R.KPQEMIDIWSQLQSAHLK.C | Isoform 2 of UDP-GlcNAc:betaGal beta-1,3-N-acetylglucosaminyltransferase 2 | BCP |
| K.QSLQEKM*TEIGIRSGWK.Y | Isoform 2 of Uncharacterized protein C8orf80 | BCP |
| K.WSPYMADVKTFLGYLVKRLIDLEMTCLAQDPTASRK.T | Isoform 2 of UPF0493 protein KIAA1632 | BCP |
| -.M*IGQKTLYSFFSPSPAR.K | Isoform 2 of Uracil-DNA glycosylase | BCP |
| K.AKENKFTVREFYYDEKEIEREREEMAR.L | Isoform 2 of Vacuolar proton pump subunit C 2 | BCP |
| K.QLVQVKNKLGMFNK.I | Isoform 2 of WD repeat-containing protein 87 | BCP |
| R.EGRDTHRKEDTYPEESR.S | Isoform 2 of Zinc finger CCCH domain-containing protein 13 | BCP |
| R.DLQKLSISTQPGVQWR.N | Isoform 3 of 5-azacytidine-induced protein 2 | BCP |
| K.RHGCSVSNDEGHQTLVMYNAESK.G | Isoform 3 of Cadherin-like protein 26 precursor | BCP |
| K.M*KM*KPEKPSLPKCGAQAGIKISSVHKRPAPEKK.E | Isoform 3 of Death-inducer obliterator 1 | BCP |
| R.NEQELRKVVGEAQTASPLPR.F | Isoform 3 of Dehydrogenase/reductase SDR family member 12 | BCP |
| R.TQMEMLTKDKADK.D | Isoform 3 of DNA repair protein RAD50 | BCP |
| R.GRFYLVGGLLAGGAR.E | Isoform 3 of Kelch domain-containing protein 9 | BCP |
| K.VDEQM*VVLMDPMEDPDDILRAHR.S | Isoform 3 of Kinesin-like protein KIF19 | BCP |
| K.LRWNTHRPAPWHALSR.L | Isoform 3 of Methyl-CpG-binding domain protein 2 | BCP |
| K.CLSLVM*DDEIESEAKIFKGYRPGKLK.L | Isoform 3 of Nexilin | BCP |
| K.LQKLNEVR.K | Isoform 3 of Pericentriolar material 1 protein | BCP |
| -.M*DLWQLLLTLALAGSSDAFSGSEDSSKEPK.F | Isoform 4 of Growth hormone receptor precursor | BCP |
| R.M*PSDSDDSLTKKM*ESHCVAQAGVQWHDLR.S | Isoform 4 of GTPase-activating Rap/Ran-GAP domain-like 1 | BCP |
| K.SQREELDAILFIFEKILQLLPERIHQRWQFHSIGLILKK.L | Isoform 4 of GTPase-activating Rap/Ran-GAP domain-like 1 | BCP |
| R.HCFDRTDSSNSMETLYHK.I | Isoform 4 of Mediator of RNA polymerase II transcription subunit 12-like protein | BCP |
| R.LHQQFESYKEQVRKIGEEARRYQGEHK.D | Isoform 4 of Regulating synaptic membrane exocytosis protein 1 | BCP |
| R.RGPGLLRAGTPLCISAEAASAQQ.- | Isoform 5 of Leukocyte-specific transcript 1 protein | BCP |
| R.QKLQLEKVTTEAKMK.K | Isoform 5 of Myosin-14 | BCP |
| R.QRKDSFQENEDGYRWQDTRGCR.T | Isoform 6 of Zinc finger CCHC domain-containing protein 6 | BCP |
| R.LTPKISFPWR.- | Isoform 7 of Merlin | BCP |
| K.SPM*VQIYELEEHK.I | Isoform A of 5'-AMP-activated protein kinase subunit gamma-2 | BCP |
| K.LEEKASASPGENDSGTGGEEPQRDK.R | Isoform A of Zinc finger homeobox protein 3 | BCP |
| R.AQFSYKWQNSPDNPQR.F | Isoform Alpha of Tripartite motif-containing protein 10 | BCP |
| K.SSVTIWQPLK.L | Isoform Alpha-1 of N-chimaerin | BCP |
| K.SFIKDYPVVSIEDPFDQDDWGAWQK.F | Isoform alpha-enolase of Alpha-enolase | BCP |
| K.LAMQEFMILPVGAANFR.E | Isoform alpha-enolase of Alpha-enolase | BCP |
| R.IGAEVYHNLK.N | Isoform alpha-enolase of Alpha-enolase | BCP |
| R.HIADLAGNSEVILPVPAFNVINGGSHAGNK.L | Isoform alpha-enolase of Alpha-enolase | BCP |
| K.SSANWMRTKARDPSM*SLM*LSGLFKSK.I | Isoform B of PR domain zinc finger protein 7 | BCP |
| K.GFQASHATECGGQVR.A | Isoform BMP1-3 of Bone morphogenetic protein 1 precursor | BCP |
| R.IGIAGSKKKSTRARLVFRVNIMRK.D | Isoform C of Nuclear factor of activated T-cells 5 | BCP |
| K.RINYTINIM*ELK.T | Isoform Flop of Glutamate receptor 2 precursor | BCP |
| R.GHSVCTNPSDKWVQDYIK.D | Isoform HCC-1 of C-C motif chemokine 14 precursor | BCP |
| K.LNECLQEVYEPDWPGRDEANK.I | Isoform IIA of Myc box-dependent-interacting protein 1 | BCP |
| K.YLDFVFAVK.N | Isoform LAMP-2A of Lysosome-associated membrane glycoprotein 2 precursor | BCP |
| R.VLGSILNASTVAAAMCM*VVTR.T | Isoform Long of 60 kDa SS-A/Ro ribonucleoprotein | BCP |
| K.KQARCQGVVCAMKEAFGFIERGDVVK.E | Isoform Long of Cold shock domain-containing protein E1 | BCP |
| R.RSVASSQPAKPTKVTLVKSR.K | Isoform Long of Tight junction protein ZO-1 | BCP |
| K.TVAPMPPAQDHKR.L | Isoform Long of Trifunctional purine biosynthetic protein adenosine-3 | BCP |
| K.SDVWSFGILLMEIVTYGR.I | Isoform p59-HCK of Tyrosine-protein kinase HCK | BCP |
| K.NYSMSVYLVRQLTSAMLLQRLKMKGIRNPDHSRALIK.E | Isoform PIAS2-beta of E3 SUMO-protein ligase PIAS2 | BCP |
| K.RFQAQSALQQHM*EVHAGVR.S | Isoform PLZFA of Zinc finger and BTB domain-containing protein 16 | BCP |
| K.LLPLPSAITSQLDKASIIRLTTSYLK.M | Isoform SIM2 of Single-minded homolog 2 | BCP |
| K.SMKELTEEQQNLQKELESLQNEHAQRMEEFYVEQK.D | Isoform SNON of Ski-like protein | BCP |
| K.SGAEDQTPKDVPNKSGAEK.Q | Isoform TGN51 of Trans-Golgi network integral membrane protein 2 precursor | BCP |
| R.RQVFGHNVIPPK.K | Isoform XD of Plasma membrane calcium-transporting ATPase 4 | BCP |
| R.EMTADVIELKGKFLINLEGGDIREESSYK.V | Isoleucyl-tRNA synthetase, mitochondrial precursor | BCP |
| K.VKFIPGSALNGMVEMM*DR.R | Isoleucyl-tRNA synthetase, mitochondrial precursor | BCP |
| R.SQYEQLAEQNRK.D | Keratin, type I cytoskeletal 10 | BCP |
| K.EKALEKSASRDVSPFVMSMQKNK.W | KIAA1841 protein | BCP |
| K.CVVEMEGNQTVLHPPPSNTKQGERLVTVAHISNSSTLGGQGKR.I | KIF13A protein | BCP |
| R.ATGPVRRRSTGLRLGAPEAR.R | Kinesin-like protein KIF13B | BCP |
| K.SVLEKTSVSEKSLAPGMALGSGRRLVSEK.A | ladinin 1 | BCP |
| R.DVMQQQLAEYQELLDVK.L | Lamin-B2 | BCP |
| R.INDISHTQSVSSK.Q | Leptin precursor | BCP |
| R.GVPTGPSHSDSGRSSSSKSTGSLGGRVAGGLLGSGTR.A | Leucine zipper putative tumor suppressor 2 | BCP |
| K.HYLLLLVGCQAWGAGLAYHGCPSECTCSR.A | Leucine-rich repeat-containing protein 15 precursor | BCP |
| R.M*GVM*TLPSDLR.K | Liprin-alpha-2 | BCP |
| R.HLTTKFNALIGPEAEQEKREKMASPAYTPLTTTAKVRPR.K | Liprin-beta-2 | BCP |
| R.FLSVGDVDDTQCVR.L | LOC554223 protein | BCP |
| -.AHWKQDLSTNATEDVRAQEQGLGR.L | LOC642032 protein (Fragment) | BCP |
| R.RKSDALSMDNCKELER.L | LOC646358 protein | BCP |
| K.CINDPNSMFLTGDTAQSIMK.G | lupus brain antigen 1 | BCP |
| R.TLPEGSAVPDSWETLPGTQVTRK.E | lupus brain antigen 1 | BCP |
| D.EVHLEELSLSK.E | Lymphocyte-specific protein 1 | BCP |
| R.YLVADKYWK.K | Lysosomal Pro-X carboxypeptidase precursor | BCP |
| R.GNEPVYTSTQEDCINSCCSTK.N | MANSC domain-containing protein 1 precursor | BCP |
| R.NANTFISPQQR.W | Matrix Gla protein precursor | BCP |
| R.GSRPQGPFLIADKWPALPR.K | Matrix metalloproteinase-9 precursor | BCP |
| R.RKIEVPSEIEVPR.E | Meiotic recombination protein REC8 homolog | BCP |
| K.IEGSKAPSNAEAKLLEGKSRR.I | Membrane associated guanylate kinase, WW and PDZ domain containing 3 | BCP |
| R.MRPHEDLSEDNSSGEVVM*R.V | membrane-associated ring finger (C3HC4) 11 | BCP |
| R.KAAEEVTLQTGIK.R | Methionine synthase | BCP |
| R.LCM*M*SSHLRKTEK.A | Microfibril-associated glycoprotein 3 precursor | BCP |
| R.ADGEYWLGLQNMHLLTLK.Q | Microfibril-associated glycoprotein 4 precursor | BCP |
| R.KSLAIRQEEVAGIRAKFPNKIPVVVER.Y | Microtubule-associated proteins 1A/1B light chain 3C precursor | BCP |
| R.AKFYPEDVSEELIQDITQR.L | Moesin | BCP |
| K.FYPEDVSEELIQDITQR.L | Moesin | BCP |
| K.YGDFNKEVHK.S | Moesin | BCP |
| K.ERQEAEEAKEALLQASR.D | Moesin | BCP |
| R.RGASLQQLLDIISEFILLGLNPEPVCVVLK.K | mTERF domain-containing protein 2 | BCP |
| R.TPVCTTGQGSGSTATVFAMAELQK.G | Multimerin-2 precursor | BCP |
| K.ISFQDFTRCRM*QLVREIRKEEVDLSAKSDNSCTK.K | mutated in colorectal cancers isoform 1 | BCP |
| K.RNM*GYSRRRLWN.- | My002 protein | BCP |
| R.ALSVVSTVVRASKDLLHR.A | MYC binding protein 2 | BCP |
| R.KSTVTDESEMQDM*MTRGNLGLLEQAIALK.A | Myelin transcription factor 1 | BCP |
| R.ARGDPTSLQASSEK.T | Myocyte-specific enhancer factor 2B | BCP |
| K.VLQLISHEKIQYGVPVIK.Y | myosin 1H | BCP |
| R.SIYQNANSM*EK.E | Myosin IIIA | BCP |
| R.VGDGKILYSQCGDVMRALGQNPTNAEVLK.V | Myosin light polypeptide 6B | BCP |
| K.QM*ARDEKNQSIIVSGESGAGKTVSAKYAM*R.Y | Myosin-Vb | BCP |
| R.EDAIDWQRTFSVGAVDFELLR.S | N-acetyl-beta-glucosaminyl-glycoprotein 4-beta-N- acetylgalactosaminyltransferase 1 | BCP |
| R.SCQEVFDKLAR.V | N-acetyllactosaminide beta-1,3-N-acetylglucosaminyltransferase | BCP |
| R.RPLTVVLQGPAGIGKTMAAKK.I | NACHT, LRR and PYD domains-containing protein 6 | BCP |
| -.M*AAAAASRGVGAKLGLREIR.I | NADH dehydrogenase [ubiquinone] 1 alpha subcomplex subunit 2 | BCP |
| -.M*AEVSSVYSVGNVYITQLASWQISVGM*M*SSLK.N | NAG20 (Fragment) | BCP |
| R.QREEAEAAARALARFAQEAEAAR.V | Neurofilament heavy polypeptide | BCP |
| K.HKRCIPASLDAYYSSQDPNSR.S | Neuron-specific protein family member 2 | BCP |
| R.EQSGDYECSASNDVAAPVVR.R | Neurotrimin variant 3 | BCP |
| R.LSTNSADKGSK.V | Neutral and basic amino acid transport protein rBAT | BCP |
| R.NQIHLFGVLLAILGNLVISISLNIQK.Y | NIPA-like protein 2 | BCP |
| R.WEHGVLYMKYPVWPRYSASLQPVVDSR.H | N-methyl-D-aspartate receptor 2C subunit precursor | BCP |
| K.IQSTVTQPGGK.F | Nodal modulator 1 precursor | BCP |
| R.SSRPPPAK.R | NOL1 protein | BCP |
| K.APAAHPEGQLK.F | Nucleobindin-1 precursor | BCP |
| R.GPRRGPAGGGEKALKRLK.L | Nucleolar MIF4G domain-containing protein 1 | BCP |
| K.GRLNELMSQIRMQNHFGAVRSEER.Y | Nucleoporin 54kDa variant (Fragment) | BCP |
| R.EPPKTRGSRGHLHTHPPGPGPPLQGLAPRGLK.T | Occludin/ELL domain-containing protein 1 | BCP |
| K.WMMETK.A | Oligodendrocyte-myelin glycoprotein precursor | BCP |
| R.QNSPVNLQLRCLQQVSVPTSVESMSPPQAR.G | OTTHUMP00000029985 | BCP |
| R.QRGWGNATGGPGEGAR.R | OTTHUMP00000031495 | BCP |
| K.TFKTGSILKAHMVTHSSR.K | p120E4F | BCP |
| K.FVTKAEEK.K | P25 | BCP |
| R.EHEPPYEISVQEEITAR.L | Pantothenate kinase 4 | BCP |
| K.VLDVNDNIPSIHVTWASQPSLVSEALPK.D | PCDH12 protein | BCP |
| K.VAASIGNAQK.L | PDZ and LIM domain protein 1 | BCP |
| R.DLVAWSRRVVVPELSAGVASR.Q | PH-interacting protein | BCP |
| K.QDYDLMVFPESDSQK.R | Phosphatidylinositol-4-phosphate 3-kinase C2 domain-containing alpha polypeptide | BCP |
| K.FIIDNTKGQMLGLGNPSFSDPFTGGGR.Y | Phospholipase A-2-activating protein | BCP |
| R.GPSEVLPPHPEVELLRSQLILKLR.Q | Phosphorylated CTD-interacting factor 1 | BCP |
| R.RSISSSRSENVLSR.L | Pleckstrin and SEC7 domain-containing protein 2 | BCP |
| K.MHDSNTGIRSSPNMEQGSTYKK.T | Pleckstrin-2 | BCP |
| -.MAAAAMAAAAGGGAGAAR.S | Poly(ADP-ribose) glycohydrolase ARH3 | BCP |
| K.RYLRCPAAMTVMHLRKFLR.S | Polycomb complex protein BMI-1 | BCP |
| R.GRTDGQVGECTEGAAPHK.H | PP13187 | BCP |
| R.LLELAGQSLLRDQALAISVLDELPRELFPR.L | PRAME family member 19 | BCP |
| K.VKHRLVENM*SSGTADALGLSR.A | PRKCA-binding protein | BCP |
| R.LLISKLYPGESIGQTSDISSPELMGVGSLLK.K | Probable E3 ubiquitin-protein ligase HERC2 | BCP |
| R.CPLQGLYLCGSGAHPGGGVMGAAGRNAAHVAFR.D | Probable oxidoreductase C10orf33 | BCP |
| R.DMYEDELVPVFER.A | Probable RNA-binding protein 46 | BCP |
| R.KLIPSPKAR.N | Proline-rich protein 11 | BCP |
| K.RDYDSSRVQSTYFNDPK.W | Proprotein convertase subtilisin/kexin type 5 precursor | BCP |
| R.GSLSVGDKDHM*CSVK.S | Prostacyclin synthase | BCP |
| R.IFFHKIFIRPLRYRSR.C | Prostaglandin D2 receptor | BCP |
| R.YLLQYQEPIPCEQLVTALCDIK.Q | Proteasome subunit alpha type-4 | BCP |
| K.ICCPAAVSRAVAGDSGS.- | Protein | BCP |
| R.LEHIMRRAAREEQPEKDFFGRVVVR.S | Protein CTF18 homolog | BCP |
| R.SPWHADDRHYNSR.A | Protein FAM116B | BCP |
| K.IIKGSSGTPKLSYTGRDDR.H | Protein KIAA0082 | BCP |
| K.ESQQGCRGLQAPLASGGPVLGR.E | Protein naked cuticle homolog 1 | BCP |
| R.NPSPPPPPGR.D | Protein SAAL1 | BCP |
| R.NGGQLEPGPAGAPSPAPGAPGPRR.R | Protein Wnt-10a precursor | BCP |
| R.ETAFTHAVSAAGVVNAISR.A | Protein Wnt-5b precursor | BCP |
| R.M*LRSSAM*DENSEK.K | Proteinase-activated receptor 2 precursor | BCP |
| R.IGQLLVCNCIFKNTLAIPLTDVK.F | Protein-glutamine gamma-glutamyltransferase 4 | BCP |
| -.M*SDAAVDTSSEITTK.D | Prothymosin alpha | BCP |
| K.SYTISPNSHFHIK.M | Protocadherin beta 11 precursor | BCP |
| K.NRPGVYTK.V | PRSS1 protein | BCP |
| -.MNPLLILTFVAAALAAPFDDDDK.I | PRSS1 protein | BCP |
| R.LVKGQSVHQLLPPTYR.D | Putative ATP-dependent RNA helicase DHX29 | BCP |
| R.LDPQAKGAPLQGRSAGSRRLLDR.D | Putative cat eye syndrome critical region protein 4 (Fragment) | BCP |
| K.ESRAAALR.A | Putative DNA helicase INO80 complex homolog 1 | BCP |
| K.ESLITDSGKLYALDVLLTRLKSQGHR.V | Putative DNA helicase INO80 complex homolog 1 | BCP |
| K.RPAVTSQPCPSAATHSLGLGSNLSFGSGAK.R | Putative protein FAM90A20 | BCP |
| R.STEHIQEDQGRRVGGPSLHGTEAPGQQR.K | Putative uncharacterized protein | BCP |
| R.KGILKSGTRSLQKVR.R | Putative uncharacterized protein CXorf58 | BCP |
| -.MANRDSASPHSPPRRRRCLGGPTVLPLR.K | Putative uncharacterized protein FLJ43491 (Fragment) | BCP |
| -.M*AEPGGRGDYRKDGR.L | Putative uncharacterized protein LOC283932 | BCP |
| K.EFSHKIHLETMCEVK.K | Pyridine nucleotide-disulphide oxidoreductase domain 1 | BCP |
| R.KAPRNPAAEK.M | Rab effector MyRIP | BCP |
| R.TQAQVNSPIVTENKYIEVGEGPAALQGK.F | Rac GTPase activating protein | BCP |
| K.RPSGNLVSVLSGAEGSFVSSLVK.S | Regenerating islet-derived protein 3 alpha precursor | BCP |
| K.TIRQKFPLLTTRR.L | Regulatory factor X domain-containing protein 1 | BCP |
| K.AEDTAVYFCAR.D | Rheumatoid factor Vh I region precursor (Fragment) | BCP |
| K.DFLFGNIR.E | Rho family guanine-nucleotide exchange factor | BCP |
| R.DRGHDSEMIGDLQAR.I | Rho-associated protein kinase 1 | BCP |
| K.KNTGKEEQRARDLFFALWIPDLFM*K.R | Ribonucleoside-diphosphate reductase large subunit | BCP |
| R.NPANRLGAGPDGVEEIK.R | Ribosomal protein S6 kinase alpha-3 | BCP |
| R.EAAAM*GPTK.F | Ribosomal protein S6 kinase delta-1 | BCP |
| K.VSVFHVKLTDSALRAFESYR.A | RNA polymerase II elongation factor ELL | BCP |
| R.ALAVRGRDGYSGPPRREPLPPR.R | RNA-binding motif protein, X-linked-like-2 | BCP |
| K.RTTNEIKNLQYLPRTSEPREMLFEDRTR.A | round spermatid basic protein 1-like | BCP |
| R.DALKEKVIK.A | S-adenosylmethionine synthetase isoform type-2 | BCP |
| R.AVPSEFANGLSPTPVNGGALDLTSSHAEKIIK.E | Sal-like protein 1 | BCP |
| K.NPIDHVSFYCKTAPNR.A | SAM domain and HD domain-containing protein 1 | BCP |
| R.QIHPPKPGVYTETNRSAER.N | sciellin isoform b | BCP |
| R.HLTCLNATQPFWDSKEPVCIAACGGVIR.N | seizure related 6 homolog isoform 1 | BCP |
| R.GHAICVYHMSSIR.A | Semaphorin-3E precursor | BCP |
| R.FVYAGSNSGVVQAPLAFCGK.H | Semaphorin-4D precursor | BCP |
| R.GDQGGESSLSVSK.W | Semaphorin-7A precursor | BCP |
| R.LFLQQIAGAM*RLLHSK.G | Serine/threonine-protein kinase ULK1 | BCP |
| K.DEISVDSLDFSK.K | Serine/threonine-protein phosphatase 2A 55 kDa regulatory subunit B beta isoform | BCP |
| K.SLQGPQPPR.H | Serpin A11 precursor | BCP |
| R.FFGHGAEDSLADQAANEWGR.S | Serum amyloid A protein precursor | BCP |
| R.KRRRRRKRRRVSAR.V | SET domain containing 1B | BCP |
| R.NPPPPISLPPSRASRASPWK.V | Seven transmembrane helix receptor | BCP |
| -.M*AAAAAGPAASQR.F | SGT1, suppressor of G2 allele of SKP1 like 1 | BCP |
| R.TWSSPRDLTDAAIGPAYR.E | Sialidase-2 | BCP |
| K.LTCQVEHDGQPAVSK.S | signal-regulatory protein alpha precursor | BCP |
| R.GEQAAKMPGRCVGPSRSGR.G | similar to alpha 1 type I collagen preproprotein | BCP |
| K.EKHLFHENCM*LQEEIALLR.L | Similar to Ankyrin repeat domain-containing protein 26. Isoform 2 | BCP |
| R.AFACREEGSQLASERLKRATRKGTVLQPVLRR.K | similar to BAH domain and coiled-coil containing 1 | BCP |
| R.MACVFVPRGGYLDPVGISLLGELIWVLHR.C | Similar to Basic proline-rich protein precursor [Contains: Proline-rich peptide SP-A (PRP-SP-A); Proline-rich peptide SP-B (PRP-SP-B); Parotid hormone (PH-Ab)]. Isoform 2 | BCP |
| -.MGCGLLTDLR.D | similar to C05G5.5 | BCP |
| -.M*EEKREAVEGRR.E | Similar to Discs large homolog 5 | BCP |
| R.LAALPWQRAYTGRPGGRGAGCELETPR.E | similar to forkhead-associated (FHA) phosphopeptide binding domain 1 isoform 8 | BCP |
| K.YLGINSDELVVGCSDAIGPK.E | similar to FRG1 protein | BCP |
| K.RATLKPGCDCSK.G | Similar to GluR-delta2 philic-protein | BCP |
| -.M*DTAKSKSESPKELVQLKK.L | Similar to Heterogeneous nuclear ribonucleoprotein A1 | BCP |
| K.KEEEENEEDEEDEEEEK.D | similar to High mobility group protein B1 | BCP |
| K.RPPSGFFLFCSEFCPKSKSTNPGIPIGDVAKKLGEMWK.N | similar to high-mobility group box 3 | BCP |
| R.TM*FNLEQLEELEKVFAK.Q | similar to notochord | BCP |
| R.RKRISVM*EDQM*NEM*KQEEK.F | similar to paraspeckle protein 1 isoform 1 | BCP |
| K.DGRKM*YLDPEAPR.I | similar to Platelet basic protein precursor | BCP |
| R.KGWCGHGHGTRDSPPR.R | Similar to Positive cofactor 2 glutamine/Q-rich-associated protein | BCP |
| K.KKERGHGYNSSAGAWQAM*KNGGKNK.N | Similar to Proline-rich nuclear receptor coactivator 2 | BCP |
| K.KLVSQTPFDVADEGLVEHLEMLQK.K | similar to protein phosphatase 1, regulatory (inhibitor) subunit 12B isoform b; myosin phosphatase regulatory subunit; myosin phosphatase, target subunit 2 | BCP |
| R.RM*TILRR.L | similar to ribosomal protein L19 | BCP |
| K.EM*GIPDVHIDTRINKALWAK.G | similar to ribosomal protein L31 | BCP |
| R.SRRRGRGRGRGARGGKAEDK.E | similar to ribosomal protein S2 isoform 1 | BCP |
| R.NSRVQVGCRELRSTKYISDGQCTSISPLK.E | Similar to Sclerostin domain-containing protein 1 precursor | BCP |
| R.NESPLAVGVITVTGVIRKRSDVGASKR.F | similar to solute carrier family 35, member F4 | BCP |
| R.HKGKETAAMR.A | similar to sorting nexin 25 | BCP |
| R.RARRLVNR.L | similar to spermatogenesis and centriole associated 1 | BCP |
| R.LSGDRKHGDTPSPLSGDRTHRDPLSPLIGDRK.H | similar to TBP-associated factor 15 isoform 1 | BCP |
| R.LSGDRKHGDAPSPLSGDHQLLCPWIGTHWVE.- | similar to TBP-associated factor 15 isoform 1 | BCP |
| R.YPVNSVNILK.A | Similar to t-complex 1; T-complex locus TCP-1; t-complex 1 | BCP |
| K.RIPKASGATILSTLANLK.G | Similar to t-complex 1; T-complex locus TCP-1; t-complex 1 | BCP |
| R.FSPSQPKLPISSGAGKSRLANSNEGISSHK.V | similar to Temporarily Assigned Gene name family member | BCP |
| R.LVDAEAHGPWIAKVQVLEGAVLLMQRTQVSWAR.G | similar to titin isoform N2-A | BCP |
| R.EGSNWVLGLSAKEG.- | Similar to Vitamin K-dependent protein Z precursor. Isoform 2 | BCP |
| K.VTCANCKKPLQKGQTAYQR.K | Similar to Zinc finger MYM-type protein 5 | BCP |
| K.GFRSSLHLNKHLRKNTLEKPYGCK.E | similar to zinc finger protein 114 | BCP |
| R.SKRQEGVDLTGIQMQCHR.L | SLIT and NTRK-like protein 3 precursor | BCP |
| K.EKDLLHVDTRKR.L | SLIT and NTRK-like protein 4 precursor | BCP |
| R.GNRARGVSRSPPKKKNKASGRRSKSPR.S | Smad nuclear-interacting protein 1 | BCP |
| R.NPLIAGK.- | Small nuclear ribonucleoprotein Sm D2 | BCP |
| K.RDSDAGSSTPTTSTR.S | SMARCA4 isoform 2 | BCP |
| R.EQQEELMRDLAGSKGTKVK.D | snRNA-activating protein complex subunit 4 | BCP |
| K.QLLGENYRNNR.K | Sodium channel protein type 10 subunit alpha | BCP |
| K.KICPLYWYK.F | Sodium channel protein type 7 subunit alpha | BCP |
| K.RSEHREPR.L | Sodium/hydrogen exchanger 3 | BCP |
| K.FYEYMLKGEISSHELNNVQDNEMLR.K | solute carrier family 9 (sodium/hydrogen exchanger), isoform 6 isoform a | BCP |
| K.QKEM*ESLKKLLSEK.Q | sorting nexin 16 isoform b | BCP |
| R.AIQECRSPVPPPAADPPEAR.T | SPEG complex locus | BCP |
| R.INATGQGVTIFYVDR.L | sperm adhesion molecule 1 isoform 1 | BCP |
| R.LDTM*AETNSISLNGPLR.T | Sperm-associated antigen 5 | BCP |
| K.RVLKGLVDKGSAGSFTLGKKQASKSK.L | Spermatid-specific linker histone H1-like protein | BCP |
| K.GIKTPEIVAPQSAHAAFNKAASYFGMKIVR.V | Sphingosine-1-phosphate lyase 1 | BCP |
| K.KFGHIPVYTLGFESPQR.V | Spire homolog 2 (Fragment) | BCP |
| R.DTPGHGSGWAETPRTDRGGDSIGETPTPGASKR.K | Splicing factor 3B subunit 1 | BCP |
| R.SRNRGPPPSWGR.R | Splicing factor, arginine/serine-rich 3 | BCP |
| K.LGTCVSCSLVYWSR.C | Stabilin-2 precursor | BCP |
| K.NLKLIEEKLGMTPIRKCNDSLR.H | structural maintenance of chromosomes flexible hinge domain containing 1 | BCP |
| K.LRGMVFGAPVPK.Q | structural maintenance of chromosomes flexible hinge domain containing 1 | BCP |
| K.ADDLGKGGNEESTKTGNAGSR.L | Superoxide dismutase | BCP |
| K.LGQGVNHAADQAGKEVEK.L | suprabasin | BCP |
| K.ELQNAHNGVNQASK.E | suprabasin | BCP |
| R.LLVPQAQPPM*PAQRRGLKRR.K | SWI/SNF-related matrix-associated actin-dependent regulator of chromatin d2 isoform 2 | BCP |
| R.TGMIESNRSIKTKRKKSFRLSRKFPFYK.S | synapse-associated protein 102 isoform b | BCP |
| K.EAKSKCK.S | synaptopodin 2 | BCP |
| R.VALVVHPGTARLGSPDEEFFHKVRTIR.Q | Syntaxin-4 | BCP |
| K.RDVGKVLMKLRKPRITATIWSSGK.I | TATA box-binding protein-like protein 1 | BCP |
| -.M*TTLSPENSLSARQSASFILVK.R | TBC1 domain family member 21 | BCP |
| R.EGTIGDMAILGITESFQVKR.Q | T-complex protein 1 subunit beta | BCP |
| R.VDCDQHSDIAQR.Y | Thioredoxin domain-containing protein 4 precursor | BCP |
| K.TKQDFIDLIEVIYR.G | Thioredoxin-like protein 4B | BCP |
| R.CGPLIDLCKGPHVRHTGK.I | threonyl-tRNA synthetase-like 2 | BCP |
| K.IKEKGSFSDTGLGDGKMKSDSFAPKTDSEKPFRGSQSPKRYK.L | Thyroid hormone receptor-associated protein 3 | BCP |
| K.MEVFKILNKKTKKGQPNLNVQMEYLLQK.I | Thyroid transcription factor 1-associated protein 26 | BCP |
| R.YTTVPSVRPTALATKK.L | TL132 protein | BCP |
| R.LTVKPNIQNPDPAVYQLRDSKSSDK.S | TRA@ protein | BCP |
| K.GLKKNDSKSTGKNLDSVQK.L | Transcription elongation factor B polypeptide 3 | BCP |
| K.IKVEPVALAPSPVIPRLTLR.V | Transcription initiation factor TFIID subunit 3 | BCP |
| R.M*LTATESR.L | Transmembrane protein 79 | BCP |
| R.DRKFREEEQQVRRQERER.K | Trichohyalin | BCP |
| K.TEGDAKGDKAKVKDESQRRSAR.L | TRMT5 protein | BCP |
| R.KNIDALSGM*EGR.K | Troponin I, cardiac muscle | BCP |
| R.LVESDHQGLHRIHVDGSSGR.L | TTC31 protein | BCP |
| K.EVAPPAVLENIQSNCHGPISM*DFAISKLK.K | Tyrosine-protein kinase JAK2 | BCP |
| R.M*PMIWLDLK.E | Tyrosine-protein phosphatase non-receptor type 23 | BCP |
| K.APAEKVGGVLCTEEKR.N | Ubinuclein | BCP |
| R.NLLLVRPLGPQASIR.L | UDP-GlcNAc:betaGal beta-1,3-N-acetylglucosaminyltransferase 8 | BCP |
| R.VAPFPFEDVYTGLCIR.A | UDP-GlcNAc:betaGal beta-1,3-N-acetylglucosaminyltransferase 8 | BCP |
| K.VVAFCDVDENK.I | UDP-GlcNAc:betaGal beta-1,3-N-acetylglucosaminyltransferase-like protein 1 | BCP |
| R.ADRDPSNM*YTKYYIHRIPR.S | UDP-N-acetylglucosamine transferase subunit ALG14 homolog | BCP |
| R.VAGITDSHMLSLPHLR.S | Uncharacterized protein AGBL2 | BCP |
| K.MRCGESGAGPDPR.S | Uncharacterized protein C10orf103 | BCP |
| R.ATIKGQIQLLQPPATSPLLQGSK.E | Uncharacterized protein C17orf46 | BCP |
| K.YSAYPETKPSAKVLVSSQVESNVR.T | Uncharacterized protein C17orf47 | BCP |
| R.KHAGPIVSVWHRELRKAKSNR.K | Uncharacterized protein C20orf77 | BCP |
| K.GSEEPLQSK.E | Uncharacterized protein C21orf13 | BCP |
| R.ICTIAKHLEAMLITR.L | Uncharacterized protein C7orf38 | BCP |
| R.KMIPIWK.Q | Uncharacterized protein C8orf32 | BCP |
| K.VQTEKASIM*FM*QSIDSVVEFCNEK.T | Uncharacterized protein CLCA4 | BCP |
| K.LQLGLQFLPGKTVVHLGSR.R | Uncharacterized protein COL27A1 | BCP |
| K.TDTMRKYITPYGSQGTVPK.M | Uncharacterized protein ENSP00000339388 (Fragment) | BCP |
| K.RPLDDGVGNQLGALVHQR.T | Uncharacterized protein FUBP3 | BCP |
| R.GFKNSSLPSHEPRGMSQR.N | Uncharacterized protein KCNIP2 | BCP |
| R.EKM*ERGNPKVSMNGSIR.N | Uncharacterized protein KIAA0319 precursor | BCP |
| R.RKATEGSGSMRGGGGGNAREYK.I | Uncharacterized protein KIAA0776 | BCP |
| R.GCGPGAAAAEPGDPQPRRRR.R | Uncharacterized protein NPAS2 (Fragment) | BCP |
| R.VIRSRSQSMDAMGLSNK.K | Uncharacterized protein RAP1GAP | BCP |
| R.GLGHQVATDALVAMEKAM*KRDKIIVNDRQLACAR.I | UPF0027 protein C22orf28 | BCP |
| K.NNPLYGDLSLEEAMEERKK.N | UPF0258 protein KIAA1024-like | BCP |
| K.TASENTNK.G | Uveal autoantigen with coiled-coil domains and ankyrin repeats | BCP |
| R.KM*NARQSK.C | V(D)J recombination-activating protein 1 | BCP |
| R.NNNRPSGISER.F | V1-20 protein | BCP |
| K.DIRMAR.F | vacuolar protein sorting 13C protein isoform 2B | BCP |
| R.LHKQSSM*TVM*EAQESPLFNNVKLQR.K | Vacuolar protein-sorting-associated protein 25 | BCP |
| R.VALNSREPHMCLVR.D | WD repeat-containing protein 43 | BCP |
| R.SEEKDEDSERGDEDRERRYR.E | WD repeat-containing protein 60 | BCP |
| R.TNSLVFRELVEELHPLM*K.E | WUGSC:H_2G3A.1 protein | BCP |
| R.SLNTSPEPLKYSWELQTKK.H | XTP3TPA-transactivated protein 2 | BCP |
| R.GRGSRGGSRGRGM*GRGSR.G | Zinc finger CCCH domain-containing protein C19orf7 | BCP |
| R.LHERTHLAGRSQCFGR.R | Zinc finger protein 101 | BCP |
| R.RGNM*NVIDVGRLL.- | zinc finger protein 12 isoform a | BCP |
| K.DTLAQHQRVHTGERPYDCSECGK.A | Zinc finger protein 304 | BCP |
| R.QDIFIEETSQGMVKKESIK.D | Zinc finger protein 354C | BCP |
| K.IKAENPGGPPVLVVPYPILASGETAKEGK.T | Zinc finger protein 362 | BCP |
| K.DAASLLILSPQNLAVSFQPPVVGIPALSLRRRGERGGGK.H | zinc finger protein 37b (KOX 21) isoform 2 | BCP |
| K.SVM*M*EKGLDWEGRSSTEKNYKCK.E | Zinc finger protein 485 | BCP |
| K.NKIQAEPSRCLK.D | zinc finger protein 518B | BCP |
| K.LILRRHKKCGHDNLQLK.K | Zinc finger protein 66 | BCP |
| -.MEIPAPEPEKTALSSQDPALSLK.E | zinc finger protein 74 | BCP |
| R.RLYICAGCGRDFK.L | Zinc finger protein 771 | BCP |
| K.DFSVILKSNHISMTSKVSGCGNQRYKR.Y | Zinc finger protein 804B | BCP |
| R.TGSEGLALPRVPR.F | Zinc finger protein GLI3 | BCP |
| -.MAAVVENVVK.L | Zinc finger protein ubi-d4 | BCP |
| K.FKAQETRMVALCTCK.A | Zinc finger, CDGSH-type domain containing protein | BCP |
| K.TLDPKDWKNQDHYAVLGLGHVRYK.A | ZRF1 protein | BCP |
| R.TIAVLLDDILQR.L | 13 kDa protein | BCP & HP |
| R.DNSKNTLYLQMNSLR.A | 13 kDa protein | BCP & HP |
| K.NTLYLQMNSLR.A | 13 kDa protein | BCP & HP |
| R.AEDTAVYYCAR.R | 13 kDa protein | BCP & HP |
| C.EVQLVESGGGLIQPGGSLR.L | 13 kDa protein | BCP & HP |
| K.NVTELNEPLSNEER.N | 14-3-3 protein gamma | BCP & HP |
| R.YDDM*AAAM*K.A | 14-3-3 protein gamma | BCP & HP |
| R.YDDMAAAM*K.A | 14-3-3 protein gamma | BCP & HP |
| K.AVTEQGAELSNEER.N | 14-3-3 protein theta | BCP & HP |
| K.EM*QPTHPIR.L | 14-3-3 protein theta | BCP & HP |
| K.EMQPTHPIR.L | 14-3-3 protein theta | BCP & HP |
| K.YYQENFCEQICSK.Q | 15 kDa protein | BCP & HP |
| R.QIVVDKYYQENFCEQICSK.Q | 15 kDa protein | BCP & HP |
| R.CPINCLLGDFGPWSDCDPCIEK.Q | 15 kDa protein | BCP & HP |
| R.SVLRPSQFGGQPCTAPLVAFQPCIPSK.L | 15 kDa protein | BCP & HP |
| K.DAQLSAPTK.Q | 184 kDa protein | BCP & HP |
| K.QLYPASAFPEDFSILTTVK.A | 184 kDa protein | BCP & HP |
| K.QKSKKLLNIKK.E | 194 kDa protein | BCP & HP |
| K.EKKGKGKPQEDELKDSLADDDSSSTTTETSNPDTEPLLK.E | 194 kDa protein | BCP & HP |
| K.NLASRPYTFHSHGITYYK.E | 20 kDa protein | BCP & HP |
| K.LISVDTGFQNK.W | 20 kDa protein | BCP & HP |
| K.KALYLQYTDETFR.T | 20 kDa protein | BCP & HP |
| K.HYYIGIIETTWDYASDHGEKK.L | 20 kDa protein | BCP & HP |
| K.HYYIGIIETTWDYASDHGEK.K | 20 kDa protein | BCP & HP |
| K.ALYLQYTDETFR.T | 20 kDa protein | BCP & HP |
| K.AETGDKVYVHLK.N | 20 kDa protein | BCP & HP |
| K.PVWLGFLGPIIK.A | 20 kDa protein | BCP & HP |
| K.VYVHLK.N | 20 kDa protein | BCP & HP |
| K.WYSEDVTISRPNDQEEITAQILTK.K | 20 kDa protein | BCP & HP |
| R.TTIEKPVWLGFLGPIIK.A | 20 kDa protein | BCP & HP |
| L.YLQYTDETFR.T | 20 kDa protein | BCP & HP |
| R.NDLSPTTVMSEGAR.N | 22 kDa protein | BCP & HP |
| R.NDLSPTTVM*SEGAR.N | 22 kDa protein | BCP & HP |
| R.CLTTDEYDGHSTYPSHQYQ.- | 22 kDa protein | BCP & HP |
| K.LKRLSYRHGLLGVDKLFSYAITEWLNDIK.K | 253 kDa protein | BCP & HP |
| R.RAEILSEM*R.A | 2'-5'-oligoadenylate synthetase 3 | BCP & HP |
| K.TETITGFQVDAVPANGQTPIQR.T | 263 kDa protein | BCP & HP |
| K.WCGTTQNYDADQKFGFCPMAAHEEICTTNEGVM*YR.I | 263 kDa protein | BCP & HP |
| K.DTLTSRPAQGVVTTLENVSPPR.R | 263 kDa protein | BCP & HP |
| K.CFDHAAGTSYVVGETWEKPYQGWMMVDCTCLGEGSGR.I | 263 kDa protein | BCP & HP |
| K.DNRGNLLQCICTGNGR.G | 263 kDa protein | BCP & HP |
| K.DSM*IWDCTCIGAGR.G | 263 kDa protein | BCP & HP |
| K.DSMIWDCTCIGAGR.G | 263 kDa protein | BCP & HP |
| K.EYLGAICSCTCFGGQR.G | 263 kDa protein | BCP & HP |
| K.TPFVTHPGYDTGNGIQLPGTSGQQPSVGQQM*IFEEHGFR.R | 263 kDa protein | BCP & HP |
| K.GEWTCKPIAEK.C | 263 kDa protein | BCP & HP |
| K.GEFKCDPHEATCYDDGKTYHVGEQWQK.E | 263 kDa protein | BCP & HP |
| K.GEFKCDPHEATCYDDGK.T | 263 kDa protein | BCP & HP |
| K.VTIMWTPPESAVTGYR.V | 263 kDa protein | BCP & HP |
| K.FTQVTPTSLSAQWTPPNVQLTGYR.V | 263 kDa protein | BCP & HP |
| K.FGFCPMAAHEEICTTNEGVMYR.I | 263 kDa protein | BCP & HP |
| K.YEVSVYALK.D | 263 kDa protein | BCP & HP |
| K.FGFCPMAAHEEICTTNEGVM*YR.I | 263 kDa protein | BCP & HP |
| K.IGEKWDR.Q | 263 kDa protein | BCP & HP |
| K.FGFCPM*AAHEEICTTNEGVM*YR.I | 263 kDa protein | BCP & HP |
| K.IYLYTLNDNAR.S | 263 kDa protein | BCP & HP |
| K.EINLAPDSSSVVVSGLMVATK.Y | 263 kDa protein | BCP & HP |
| K.EINLAPDSSSVVVSGLM*VATK.Y | 263 kDa protein | BCP & HP |
| K.WDRQGENGQMMSCTCLGNGK.G | 263 kDa protein | BCP & HP |
| K.DTLTSRPAQGVVTTLENVSPPRR.A | 263 kDa protein | BCP & HP |
| K.CDPVDQCQDSETGTFYQIGDSWEK.Y | 263 kDa protein | BCP & HP |
| K.CDPHEATCYDDGKTYHVGEQWQK.E | 263 kDa protein | BCP & HP |
| K.CDPHEATCYDDGK.T | 263 kDa protein | BCP & HP |
| I.PVNLPGEHGQR.L | 263 kDa protein | BCP & HP |
| K.WCGTTQNYDADQKFGFCPMAAHEEICTTNEGVMYR.I | 263 kDa protein | BCP & HP |
| K.FGFCPM*AAHEEICTTNEGVMYR.I | 263 kDa protein | BCP & HP |
| K.TPFVTHPGYDTGNGIQLPGTSGQQPSVGQQMIFEEHGFRR.T | 263 kDa protein | BCP & HP |
| K.WCGTTQNYDADQK.F | 263 kDa protein | BCP & HP |
| K.VVTPLSPPTNLHLEANPDTGVLTVSWER.S | 263 kDa protein | BCP & HP |
| K.VTIMWTPPESAVTGYRVDVIPVNLPGEHGQR.L | 263 kDa protein | BCP & HP |
| K.WLPSSSPVTGYR.V | 263 kDa protein | BCP & HP |
| K.YEKPGSPPR.E | 263 kDa protein | BCP & HP |
| K.VTIM*WTPPESAVTGYRVDVIPVNLPGEHGQR.L | 263 kDa protein | BCP & HP |
| K.VTIM*WTPPESAVTGYR.V | 263 kDa protein | BCP & HP |
| K.VREEVVTVGNSVNEGLNQPTDDSCFDPYTVSHYAVGDEWER.M | 263 kDa protein | BCP & HP |
| K.VFAVSHGR.E | 263 kDa protein | BCP & HP |
| K.HYQINQQWER.T | 263 kDa protein | BCP & HP |
| K.TYHVGEQWQK.E | 263 kDa protein | BCP & HP |
| K.WCGTTQNYDADQKFGFCPM*AAHEEICTTNEGVMYR.I | 263 kDa protein | BCP & HP |
| K.TPFVTHPGYDTGNGIQLPGTSGQQPSVGQQMIFEEHGFR.R | 263 kDa protein | BCP & HP |
| K.TPFVTHPGYDTGNGIQLPGTSGQQPSVGQQM*IFEEHGFRR.T | 263 kDa protein | BCP & HP |
| K.TGPMKEINLAPDSSSVVVSGLMVATK.Y | 263 kDa protein | BCP & HP |
| K.STATISGLKPGVDYTITVYAVTGR.G | 263 kDa protein | BCP & HP |
| K.QMLCTCLGNGVSCQETAVTQTYGGNSNGEPCVLPFTYNGR.T | 263 kDa protein | BCP & HP |
| K.QM*LCTCLGNGVSCQETAVTQTYGGNSNGEPCVLPFTYNGR.T | 263 kDa protein | BCP & HP |
| K.NNQKSEPLIGR.K | 263 kDa protein | BCP & HP |
| K.LLCQCLGFGSGHFRCDSSR.W | 263 kDa protein | BCP & HP |
| K.LLCQCLGFGSGHFR.C | 263 kDa protein | BCP & HP |
| K.LGVRPSQGGEAPR.E | 263 kDa protein | BCP & HP |
| K.TYHVGEQWQKEYLGAICSCTCFGGQR.G | 263 kDa protein | BCP & HP |
| R.QYNVGPSVSK.Y | 263 kDa protein | BCP & HP |
| R.RPGGEPSPEGTTGQSYNQYSQR.Y | 263 kDa protein | BCP & HP |
| R.RPHETGGYM*LECVCLGNGK.G | 263 kDa protein | BCP & HP |
| R.QGENGQMMSCTCLGNGKGEFK.C | 263 kDa protein | BCP & HP |
| R.RPHETGGYMLECVCLGNGK.G | 263 kDa protein | BCP & HP |
| R.QYNVGPSVSKYPLR.N | 263 kDa protein | BCP & HP |
| R.SDTVPSPR.D | 263 kDa protein | BCP & HP |
| R.SSPVVIDASTAIDAPSNLR.F | 263 kDa protein | BCP & HP |
| R.SYTITGLQPGTDYK.I | 263 kDa protein | BCP & HP |
| R.TEIDKPSQM*QVTDVQDNSISVK.W | 263 kDa protein | BCP & HP |
| R.QGENGQMMSCTCLGNGK.G | 263 kDa protein | BCP & HP |
| R.AAVYQPQPHPQPPPYGHCVTDSGVVYSVGMQWLK.T | 263 kDa protein | BCP & HP |
| R.SYTITGLQPGTDYKIYLYTLNDNAR.S | 263 kDa protein | BCP & HP |
| R.STTPDITGYR.I | 263 kDa protein | BCP & HP |
| R.QGENGQMM*SCTCLGNGKGEFK.C | 263 kDa protein | BCP & HP |
| R.QGENGQMM*SCTCLGNGK.G | 263 kDa protein | BCP & HP |
| K.YTGNTYR.V | 263 kDa protein | BCP & HP |
| M.AAHEEICTTNEGVM*YR.I | 263 kDa protein | BCP & HP |
| M.M*VDCTCLGEGSGR.I | 263 kDa protein | BCP & HP |
| M.MVDCTCLGEGSGR.I | 263 kDa protein | BCP & HP |
| P.GGEPSPEGTTGQSYNQYSQR.Y | 263 kDa protein | BCP & HP |
| P.HETGGYMLECVCLGNGK.G | 263 kDa protein | BCP & HP |
| Q.PQSPVAVSQSKPGCYDNGK.H | 263 kDa protein | BCP & HP |
| R.AAVYQPQPHPQPPPYGHCVTDSGVVYSVGM*QWLK.T | 263 kDa protein | BCP & HP |
| R.AQITGYR.L | 263 kDa protein | BCP & HP |
| R.TEIDKPSQMQVTDVQDNSISVK.W | 263 kDa protein | BCP & HP |
| S.PPTNLHLEANPDTGVLTVSWER.S | 263 kDa protein | BCP & HP |
| Q.TAVPPPTDLRFFSDITANSFTVHWIAPR.A | 263 kDa protein | BCP & HP |
| R.QGENGQM*MSCTCLGNGK.G | 263 kDa protein | BCP & HP |
| R.WSRPQAPITGYR.I | 263 kDa protein | BCP & HP |
| R.YQCYCYGR.G | 263 kDa protein | BCP & HP |
| R.EESPLLIGQQSTVSDVPR.D | 263 kDa protein | BCP & HP |
| V.TGLSPGVTYYFK.V | 263 kDa protein | BCP & HP |
| R.CDNCRRPGGEPSPEGTTGQSYNQYSQR.Y | 263 kDa protein | BCP & HP |
| R.WKEATIPGHLNSYTIK.G | 263 kDa protein | BCP & HP |
| R.WKCDPVDQCQDSETGTFYQIGDSWEK.Y | 263 kDa protein | BCP & HP |
| R.WCHDNGVNYK.I | 263 kDa protein | BCP & HP |
| R.VTDATETTITISWR.T | 263 kDa protein | BCP & HP |
| R.VPGTSTSATLTGLTR.G | 263 kDa protein | BCP & HP |
| R.VGDTYERPKDSMIWDCTCIGAGR.G | 263 kDa protein | BCP & HP |
| R.VGDTYERPKDSM*IWDCTCIGAGR.G | 263 kDa protein | BCP & HP |
| Y.AVEENQESTPVVIQQETTGTPR.S | 263 kDa protein | BCP & HP |
| R.VEYELSEEGDEPQYLDLPSTATSVNIPDLLPGRK.Y | 263 kDa protein | BCP & HP |
| R.TFYSCTTEGR.Q | 263 kDa protein | BCP & HP |
| K.YEVSVYALKDTLTSRPAQGVVTTLENVSPPRR.A | 263 kDa protein | BCP & HP |
| R.VDVIPVNLPGEHGQR.L | 263 kDa protein | BCP & HP |
| R.TYLGNALVCTCYGGSR.G | 263 kDa protein | BCP & HP |
| R.TNTNVNCPIECFMPLDVQADREDSRE.- | 263 kDa protein | BCP & HP |
| R.TNTNVNCPIECFMPLDVQADR.E | 263 kDa protein | BCP & HP |
| R.TNTNVNCPIECFMPLDVQA.D | 263 kDa protein | BCP & HP |
| R.TNTNVNCPIECFMPLDVQ.A | 263 kDa protein | BCP & HP |
| R.TNTNVNCPIECFM*PLDVQADREDSRE.- | 263 kDa protein | BCP & HP |
| R.TNTNVNCPIECFM*PLDVQADR.E | 263 kDa protein | BCP & HP |
| R.TNTNVNCPIECFM*PLDVQA.D | 263 kDa protein | BCP & HP |
| R.TKTETITGFQVDAVPANGQTPIQR.T | 263 kDa protein | BCP & HP |
| R.TFYSCTTEGRQDGHLWCSTTSNYEQDQK.Y | 263 kDa protein | BCP & HP |
| R.VGDTYERPK.D | 263 kDa protein | BCP & HP |
| R.CTCVGNGRGEWTCIAYSQLR.D | 263 kDa protein | BCP & HP |
| R.IGDQWDKQHDMGHM*M*R.C | 263 kDa protein | BCP & HP |
| R.IGDQWDKQHDMGHM*MR.C | 263 kDa protein | BCP & HP |
| R.IGDQWDKQHDMGHMM*R.C | 263 kDa protein | BCP & HP |
| R.IGDTWSK.K | 263 kDa protein | BCP & HP |
| R.ISCTIANR.C | 263 kDa protein | BCP & HP |
| R.ITGYIIKYEKPGSPPR.E | 263 kDa protein | BCP & HP |
| R.ITYGETGGNSPVQEFTVPGSK.S | 263 kDa protein | BCP & HP |
| R.KKTDELPQLVTLPHPNLHGPEILDVPSTVQK.T | 263 kDa protein | BCP & HP |
| R.LTVGLTRR.G | 263 kDa protein | BCP & HP |
| R.MSESGFK.L | 263 kDa protein | BCP & HP |
| R.QGENGQM*M*SCTCLGNGK.G | 263 kDa protein | BCP & HP |
| R.IGDQWDKQHDM*GHMMR.C | 263 kDa protein | BCP & HP |
| R.QDGHLWCSTTSNYEQDQK.Y | 263 kDa protein | BCP & HP |
| R.IGDQWDKQHDMGHMMR.C | 263 kDa protein | BCP & HP |
| R.QAQQMVQPQSPVAVSQSKPGCYDNGKHYQINQQWER.T | 263 kDa protein | BCP & HP |
| R.DNM*KWCGTTQNYDADQK.F | 263 kDa protein | BCP & HP |
| R.QAQQMVQPQSPVAVSQSKPGCYDNGK.H | 263 kDa protein | BCP & HP |
| R.QAQQM*VQPQSPVAVSQSKPGCYDNGK.H | 263 kDa protein | BCP & HP |
| R.PRPGVTEATITGLEPGTEYTIYVIALK.N | 263 kDa protein | BCP & HP |
| K.YEVSVYALKDTLTSRPAQGVVTTLENVSPPR.R | 263 kDa protein | BCP & HP |
| R.PGGEPSPEGTTGQSYNQYSQR.Y | 263 kDa protein | BCP & HP |
| R.NTFAEVTGLSPGVTYYFK.V | 263 kDa protein | BCP & HP |
| R.NSITLTNLTPGTEYVVSIVALNGREESPLLIGQQSTVSDVPR.D | 263 kDa protein | BCP & HP |
| R.NSITLTNLTPGTEYVVSIVALNGR.E | 263 kDa protein | BCP & HP |
| R.NLQPASEYTVSLVAIKGNQESPK.A | 263 kDa protein | BCP & HP |
| R.NLQPASEYTVSLVAIK.G | 263 kDa protein | BCP & HP |
| R.QDGHLWCSTTSNYEQDQKYSFCTDHTVLVQTR.G | 263 kDa protein | BCP & HP |
| R.ESKPLTAQQTTK.L | 263 kDa protein | BCP & HP |
| R.DAPIVNK.V | 263 kDa protein | BCP & HP |
| R.DAPIVNKVVTPLSPPTNLHLEANPDTGVLTVSWER.S | 263 kDa protein | BCP & HP |
| R.DGQERDAPIVNK.V | 263 kDa protein | BCP & HP |
| R.DGQERDAPIVNKVVTPLSPPTNLHLEANPDTGVLTVSWER.S | 263 kDa protein | BCP & HP |
| R.DLEVVAATPTSLLISWDAPAVTVR.Y | 263 kDa protein | BCP & HP |
| R.DLQFVEVTDVK.V | 263 kDa protein | BCP & HP |
| K.YSFCTDHTVLVQTR.G | 263 kDa protein | BCP & HP |
| R.DNMKWCGTTQNYDADQK.F | 263 kDa protein | BCP & HP |
| R.IGDTWSKK.D | 263 kDa protein | BCP & HP |
| R.EEVVTVGNSVNEGLNQPTDDSCFDPYTVSHYAVGDEWER.M | 263 kDa protein | BCP & HP |
| R.IGDQWDKQHDM*GHM*M*R.C | 263 kDa protein | BCP & HP |
| R.EVTSDSGSIVVSGLTPGVEYVYTIQVLR.D | 263 kDa protein | BCP & HP |
| R.EVVPRPRPGVTEATITGLEPGTEYTIYVIALK.N | 263 kDa protein | BCP & HP |
| R.FFSDITANSFTVHWIAPR.A | 263 kDa protein | BCP & HP |
| R.HEEGHMLNCTCFGQGR.G | 263 kDa protein | BCP & HP |
| R.IGDQWDK.Q | 263 kDa protein | BCP & HP |
| R.QGENGQM*M*SCTCLGNGKGEFK.C | 263 kDa protein | BCP & HP |
| R.HTSVQTTSSGSGPFTDVR.A | 263 kDa protein | BCP & HP |
| R.FLATTPNSLLVSWQPPR.A | 263 kDa protein | BCP & HP |
| R.HEEGHM*LNCTCFGQGR.G | 263 kDa protein | BCP & HP |
| R.GRISCTIANR.C | 263 kDa protein | BCP & HP |
| R.GDSPASSKPISINYR.T | 263 kDa protein | BCP & HP |
| R.GNLLQCICTGNGR.G | 263 kDa protein | BCP & HP |
| R.GFNCESKPEAEETCFDKYTGNTYR.V | 263 kDa protein | BCP & HP |
| R.GFNCESKPEAEETCFDK.Y | 263 kDa protein | BCP & HP |
| R.GEWTCIAYSQLR.D | 263 kDa protein | BCP & HP |
| R.GNLLQCICTGNGRGEWK.C | 263 kDa protein | BCP & HP |
| R.YQDTNMQGVVYELNSYIEQ.R | 3',5'-cyclic nucleotide phosphodiesterase 10A2 | BCP & HP |
| R.QSFASDSSSK.S | 30 kDa protein | BCP & HP |
| K.TVMIDVCTTCR.C | 309 kDa protein | BCP & HP |
| K.AFVVDMMER.L | 309 kDa protein | BCP & HP |
| K.TYGLCGICDENGANDFMLR.D | 309 kDa protein | BCP & HP |
| K.AHLLSLVDVMQR.E | 309 kDa protein | BCP & HP |
| K.VCGLCGNFDGIQNNDLTSSNLQVEEDPVDFGNSWK.V | 309 kDa protein | BCP & HP |
| K.VIVIPVGIGPHANLK.Q | 309 kDa protein | BCP & HP |
| K.YAGSQVASTSEVLK.Y | 309 kDa protein | BCP & HP |
| K.YLFPGECQYVLVQDYCGSNPGTFR.I | 309 kDa protein | BCP & HP |
| R.YDVCSCSDGRECLCGALASYAAACAGR.G | 309 kDa protein | BCP & HP |
| R.CLPSACEVVTGSPR.G | 309 kDa protein | BCP & HP |
| K.SEVEVDIHYCQGK.C | 309 kDa protein | BCP & HP |
| R.CLPTACTIQLR.G | 309 kDa protein | BCP & HP |
| R.CMVQVGVISGFK.L | 309 kDa protein | BCP & HP |
| R.VAQCSQKPCEDSCR.S | 309 kDa protein | BCP & HP |
| R.VAVVEYHDGSHAYIGLKDR.K | 309 kDa protein | BCP & HP |
| R.VKEEVFIQQR.N | 309 kDa protein | BCP & HP |
| K.AMYSIDINDVQDQCSCCSPTR.T | 309 kDa protein | BCP & HP |
| R.VTVFPIGIGDRYDAAQLR.I | 309 kDa protein | BCP & HP |
| K.YTLFQIFSK.I | 309 kDa protein | BCP & HP |
| K.LTGSCSYVLFQNK.E | 309 kDa protein | BCP & HP |
| K.EQDLEVILHNGACSPGAR.Q | 309 kDa protein | BCP & HP |
| K.GLWEQCQLLK.S | 309 kDa protein | BCP & HP |
| K.IGCNTCVCQDR.K | 309 kDa protein | BCP & HP |
| K.ILDELLQTCVDPEDCPVCEVAGR.R | 309 kDa protein | BCP & HP |
| K.ILDELLQTCVDPEDCPVCEVAGRR.F | 309 kDa protein | BCP & HP |
| K.IPGTCCDTCEEPECNDITAR.L | 309 kDa protein | BCP & HP |
| K.LHGDCQDLQK.Q | 309 kDa protein | BCP & HP |
| K.TLCECAGGLECACPALLEYAR.T | 309 kDa protein | BCP & HP |
| K.LSGEAYGFVAR.I | 309 kDa protein | BCP & HP |
| K.SVGSQWASPENPCLINECVR.V | 309 kDa protein | BCP & HP |
| K.QHSDPCALNPR.M | 309 kDa protein | BCP & HP |
| K.QTM*VDSSCR.I | 309 kDa protein | BCP & HP |
| K.QTMVDSSCR.I | 309 kDa protein | BCP & HP |
| K.RDETLQDGCDTHFCK.V | 309 kDa protein | BCP & HP |
| K.RLPGDIQVVPIGVGPNANVQELER.I | 309 kDa protein | BCP & HP |
| K.RPGDVWTLPDQCHTVTCQPDGQTLLK.S | 309 kDa protein | BCP & HP |
| K.RYPPGTSLSR.D | 309 kDa protein | BCP & HP |
| R.YDVCSCSDGR.E | 309 kDa protein | BCP & HP |
| K.LKHGAGVAMDGQDVQLPLLK.G | 309 kDa protein | BCP & HP |
| R.SLSCRPPMVK.L | 309 kDa protein | BCP & HP |
| R.VSM*PYASK.G | 309 kDa protein | BCP & HP |
| R.KVPLDSSPATCHNNIMK.Q | 309 kDa protein | BCP & HP |
| R.LLDLVFLLDGSSR.L | 309 kDa protein | BCP & HP |
| R.LPGDIQVVPIGVGPNANVQELER.I | 309 kDa protein | BCP & HP |
| R.LRQNADQCCPEYECVCDPVSCDLPPVPHCER.G | 309 kDa protein | BCP & HP |
| R.LSEAEFEVLK.A | 309 kDa protein | BCP & HP |
| R.LTQVSVLQYGSITTIDVPWNVVPEK.A | 309 kDa protein | BCP & HP |
| R.NLRENGYECEWR.Y | 309 kDa protein | BCP & HP |
| R.KTTCNPCPLGYKEENNTGECCGR.C | 309 kDa protein | BCP & HP |
| R.SKEFMEEVIQR.M | 309 kDa protein | BCP & HP |
| R.IQHTVTASVR.L | 309 kDa protein | BCP & HP |
| R.SNRVTVFPIGIGDRYDAAQLR.I | 309 kDa protein | BCP & HP |
| R.STIYPVGQFWEEGCDVCTCTDM*EDAVM*GLR.V | 309 kDa protein | BCP & HP |
| R.STIYPVGQFWEEGCDVCTCTDMEDAVMGLR.V | 309 kDa protein | BCP & HP |
| R.TATLCPQSCEER.N | 309 kDa protein | BCP & HP |
| R.TCAQEGMVLYGWTDHSACSPVCPAGMEYR.Q | 309 kDa protein | BCP & HP |
| R.TCQSLHINEM*CQER.C | 309 kDa protein | BCP & HP |
| R.TCQSLHINEMCQER.C | 309 kDa protein | BCP & HP |
| R.TNGVCVDWR.T | 309 kDa protein | BCP & HP |
| R.TPDFCAMSCPPSLVYNHCEHGCPR.H | 309 kDa protein | BCP & HP |
| R.SGFTYVLHEGECCGR.C | 309 kDa protein | BCP & HP |
| R.GLRPSCPNSQSPVK.V | 309 kDa protein | BCP & HP |
| R.CPCFHQGKEYAPGETVK.I | 309 kDa protein | BCP & HP |
| R.CVALER.C | 309 kDa protein | BCP & HP |
| R.CVDGCSCPEGQLLDEGLCVESTECPCVHSGK.R | 309 kDa protein | BCP & HP |
| R.DCNTCICR.N | 309 kDa protein | BCP & HP |
| R.DGTVTTDWK.T | 309 kDa protein | BCP & HP |
| R.EAPDLVLQR.C | 309 kDa protein | BCP & HP |
| R.EGGPSQIGDALGFAVR.Y | 309 kDa protein | BCP & HP |
| R.KVPLDSSPATCHNNIM*K.Q | 309 kDa protein | BCP & HP |
| R.FSEEACAVLTSPTFEACHR.A | 309 kDa protein | BCP & HP |
| R.YLSDHSFLVSQGDREQAPNLVYMVTGNPASDEIK.R | 309 kDa protein | BCP & HP |
| R.GLRPSCPNSQSPVKVEETCGCR.W | 309 kDa protein | BCP & HP |
| R.HIVTFDGQNFK.L | 309 kDa protein | BCP & HP |
| R.IEDLPTMVTLGNSFLHK.L | 309 kDa protein | BCP & HP |
| R.IGWPNAPILIQDFETLPR.E | 309 kDa protein | BCP & HP |
| R.ILAGPAGDSNVVK.L | 309 kDa protein | BCP & HP |
| R.ILTSDVFQDCNK.L | 309 kDa protein | BCP & HP |
| R.FNHLGHIFTFTPQNNEFQLQLSPK.T | 309 kDa protein | BCP & HP |
| K.GQSEDPGSLLSLFR.R | 4F2 cell-surface antigen heavy chain | BCP & HP |
| R.IGDLQAFQGHGAGNLAGLK.G | 4F2 cell-surface antigen heavy chain | BCP & HP |
| R.LLTSFLPAQLLR.L | 4F2 cell-surface antigen heavy chain | BCP & HP |
| K.DDVAQTDLLQIDPNFGSKEDFDSLLQSAK.K | 4F2 cell-surface antigen heavy chain | BCP & HP |
| R.CIFLMKHGERVGWPVGVSMPR.G | 62 kDa protein | BCP & HP |
| R.FQGTSYDSCTTEGR.T | 72 kDa type IV collagenase precursor | BCP & HP |
| K.FFGLPQTGDLDQNTIETMR.K | 72 kDa type IV collagenase precursor | BCP & HP |
| R.DKPMGPLLVATFWPELPEKIDAVYEAPQEEK.A | 72 kDa type IV collagenase precursor | BCP & HP |
| R.VKYGNADGEYCKFPFLFNGK.E | 72 kDa type IV collagenase precursor | BCP & HP |
| R.CGNPDVANYNFFPR.K | 72 kDa type IV collagenase precursor | BCP & HP |
| R.AFQVWSDVTPLR.F | 72 kDa type IV collagenase precursor | BCP & HP |
| K.YGFCPHEALFTMGGNAEGQPCKFPFR.F | 72 kDa type IV collagenase precursor | BCP & HP |
| K.YGFCPETAMSTVGGNSEGAPCVFPFTFLGNKYESCTSAGR.S | 72 kDa type IV collagenase precursor | BCP & HP |
| K.TYIFAGDKFWR.Y | 72 kDa type IV collagenase precursor | BCP & HP |
| K.TDKELAVQYLNTFYGCPK.E | 72 kDa type IV collagenase precursor | BCP & HP |
| R.IIGYTPDLDPETVDDAFAR.A | 72 kDa type IV collagenase precursor | BCP & HP |
| K.SSTGPGEQLR.N | 80 kDa protein | BCP & HP |
| R.VPNSDQKDSDGDGIGDACDNCPQK.S | 80 kDa protein | BCP & HP |
| R.TGLPSVRPLLHCAPGFCFPGVACIQTESGAR.C | 80 kDa protein | BCP & HP |
| R.SCVCAVGWAGNGILCGR.D | 80 kDa protein | BCP & HP |
| R.NALWHTGDTESQVR.L | 80 kDa protein | BCP & HP |
| R.GSFQCGPCQPGFVGDQASGCQR.G | 80 kDa protein | BCP & HP |
| R.FYEGPELVADSNVVLDTTMR.G | 80 kDa protein | BCP & HP |
| R.FCPDGSPSECHEHADCVLER.D | 80 kDa protein | BCP & HP |
| R.ELQETNAALQDVR.D | 80 kDa protein | BCP & HP |
| R.DTDLDGFPDEK.L | 80 kDa protein | BCP & HP |
| R.DNCPTVPNSAQEDSDHDGQGDACDDDDDNDGVPDSR.D | 80 kDa protein | BCP & HP |
| R.AVAEPGIQLK.A | 80 kDa protein | BCP & HP |
| R.CEACPPGYSGPTHQGVGLAFAK.A | 80 kDa protein | BCP & HP |
| K.SNPDQADVDHDFVGDACDSDQDQDGDGHQDSR.D | 80 kDa protein | BCP & HP |
| K.QVCTDINECETGQHNCVPNSVCINTR.G | 80 kDa protein | BCP & HP |
| K.QMEQTYWQANPFR.A | 80 kDa protein | BCP & HP |
| K.NTVMECDACGMQQSVR.T | 80 kDa protein | BCP & HP |
| K.NTVMECDACGM*QQSVR.T | 80 kDa protein | BCP & HP |
| K.NTVM*ECDACGMQQSVR.T | 80 kDa protein | BCP & HP |
| K.NTVM*ECDACGM*QQSVR.T | 80 kDa protein | BCP & HP |
| K.DSDGDGIGDACDNCPQK.S | 80 kDa protein | BCP & HP |
| K.DNCVTVPNSGQEDVDR.D | 80 kDa protein | BCP & HP |
| K.DNCPLVR.N | 80 kDa protein | BCP & HP |
| R.CINTSPGFR.C | 80 kDa protein | BCP & HP |
| R.AFQTVVLDPEGDAQIDPNWVVLNQGR.E | 80 kDa protein | BCP & HP |
| K.YFFKDQVLVSCDTGYK.V | 92 kDa protein | BCP & HP |
| R.SLPTCLPVCGLPK.F | 92 kDa protein | BCP & HP |
| R.LRSDENEQHLGVK.H | 92 kDa protein | BCP & HP |
| R.FPETLMEIEIPIVDHSTCQK.A | 92 kDa protein | BCP & HP |
| R.ETTDTEQTPGQEVVLSPGSFM*SITFR.S | 92 kDa protein | BCP & HP |
| R.DMICAGEKEGGKDACAGDSGGPMVTLNR.E | 92 kDa protein | BCP & HP |
| R.DMICAGEK.E | 92 kDa protein | BCP & HP |
| R.APGELEHGLITFSTR.N | 92 kDa protein | BCP & HP |
| R.TCRVECSDNLFTQR.T | 92 kDa protein | BCP & HP |
| K.YSCQEPYYK.M | 92 kDa protein | BCP & HP |
| R.ETTDTEQTPGQEVVLSPGSFMSITFR.S | 92 kDa protein | BCP & HP |
| K.VLGPFCGEKAPEPISTQSHSVLILFHSDNSGENR.G | 92 kDa protein | BCP & HP |
| K.VETEDQVLATFCGR.E | 92 kDa protein | BCP & HP |
| K.SDFSNEER.F | 92 kDa protein | BCP & HP |
| K.EGGKDACAGDSGGPMVTLNR.E | 92 kDa protein | BCP & HP |
| K.DQVLVSCDTGYK.V | 92 kDa protein | BCP & HP |
| K.DGTWSNKIPTCK.K | 92 kDa protein | BCP & HP |
| K.DACAGDSGGPMVTLNR.E | 92 kDa protein | BCP & HP |
| K.DACAGDSGGPM*VTLNR.E | 92 kDa protein | BCP & HP |
| K.APEPISTQSHSVLILFHSDNSGENR.G | 92 kDa protein | BCP & HP |
| R.AAGNECPELQPPVHGK.I | 92 kDa protein | BCP & HP |
| R.VECSDNLFTQR.T | 92 kDa protein | BCP & HP |
| R.TGVITSPDFPNPYPK.S | 92 kDa protein | BCP & HP |
| R.DSDLLSPSDFK.I | 92 kDa protein | BCP & HP |
| R.M*YSVNGYTFGSLPGLSMCAEDRVK.W | 97 kDa protein | BCP & HP |
| R.KAEEEHLGILGPQLHADVGDKVK.I | 97 kDa protein | BCP & HP |
| R.KERGPEEEHLGILGPVIWAEVGDTIR.V | 97 kDa protein | BCP & HP |
| R.M*FTTAPDQVDK.E | 97 kDa protein | BCP & HP |
| R.M*FTTAPDQVDKEDEDFQESNK.M | 97 kDa protein | BCP & HP |
| R.M*YSVNGYTFGSLPGLSM*CAEDR.V | 97 kDa protein | BCP & HP |
| R.MYSVNGYTFGSLPGLSMCAEDRVK.W | 97 kDa protein | BCP & HP |
| R.M*YSVNGYTFGSLPGLSMCAEDR.V | 97 kDa protein | BCP & HP |
| R.IDTINLFPATLFDAYMVAQNPGEWM*LSCQNLNHLK.A | 97 kDa protein | BCP & HP |
| R.MFTTAPDQVDK.E | 97 kDa protein | BCP & HP |
| R.MFTTAPDQVDKEDEDFQESNK.M | 97 kDa protein | BCP & HP |
| R.MFTTAPDQVDKEDEDFQESNKMHSMNGFMYGNQPGLTMCK.G | 97 kDa protein | BCP & HP |
| R.MYSVNGYTFGSLPGLSM*CAEDR.V | 97 kDa protein | BCP & HP |
| R.MYSVNGYTFGSLPGLSM*CAEDRVK.W | 97 kDa protein | BCP & HP |
| K.EVGPTNADPVCLAK.M | 97 kDa protein | BCP & HP |
| R.M*YSVNGYTFGSLPGLSM*CAEDRVK.W | 97 kDa protein | BCP & HP |
| R.GPEEEHLGILGPVIWAEVGDTIR.V | 97 kDa protein | BCP & HP |
| R.DTANLFPQTSLTLHM*WPDTEGTFNVECLTTDHYTGGM*K.Q | 97 kDa protein | BCP & HP |
| R.DTANLFPQTSLTLHM*WPDTEGTFNVECLTTDHYTGGMK.Q | 97 kDa protein | BCP & HP |
| R.DTANLFPQTSLTLHMWPDTEGTFNVECLTTDHYTGGM*K.Q | 97 kDa protein | BCP & HP |
| R.DTANLFPQTSLTLHMWPDTEGTFNVECLTTDHYTGGMK.Q | 97 kDa protein | BCP & HP |
| R.EYTDASFTNR.K | 97 kDa protein | BCP & HP |
| R.EYTDASFTNRK.E | 97 kDa protein | BCP & HP |
| R.KAEEEHLGILGPQLHADVGDK.V | 97 kDa protein | BCP & HP |
| R.FNKNNEGTYYSPNYNPQSR.S | 97 kDa protein | BCP & HP |
| R.IDTINLFPATLFDAYMVAQNPGEWMLSCQNLNHLK.A | 97 kDa protein | BCP & HP |
| R.GVYSSDVFDIFPGTYQTLEM*FPR.T | 97 kDa protein | BCP & HP |
| R.GVYSSDVFDIFPGTYQTLEMFPR.T | 97 kDa protein | BCP & HP |
| R.HYYIAAEEIIWNYAPSGIDIFTK.E | 97 kDa protein | BCP & HP |
| R.IDTINLFPATLFDAYM*VAQNPGEWM*LSCQNLNHLK.A | 97 kDa protein | BCP & HP |
| R.IDTINLFPATLFDAYM*VAQNPGEWMLSCQNLNHLK.A | 97 kDa protein | BCP & HP |
| R.PYSIHAHGVQTESSTVTPTLPGETLTYVWK.I | 97 kDa protein | BCP & HP |
| R.FNKNNEGTYYSPNYNPQS.R | 97 kDa protein | BCP & HP |
| K.HRGVYSSDVFDIFPGTYQTLEMFPR.T | 97 kDa protein | BCP & HP |
| V.AQNPGEWMLSCQNLNHLK.A | 97 kDa protein | BCP & HP |
| V.GPTNADPVCLAK.M | 97 kDa protein | BCP & HP |
| V.PPSASHVAPTETFTYEWTVPK.E | 97 kDa protein | BCP & HP |
| W.AYYSTVDQVK.D | 97 kDa protein | BCP & HP |
| Y.LFPTVFDENESLLLEDNIR.M | 97 kDa protein | BCP & HP |
| R.MYSVNGYTFGSLPGLSMCAEDR.V | 97 kDa protein | BCP & HP |
| Y.SIHAHGVQTESSTVTPTLPGETLTYVWK.I | 97 kDa protein | BCP & HP |
| R.TYYIAAVEVEWDYSPQR.E | 97 kDa protein | BCP & HP |
| K.ICKKGSLHANGRQKDVDKEFYLFPTVFDENESLLLEDNIR.M | 97 kDa protein | BCP & HP |
| K.LVYREYTDASFTNR.K | 97 kDa protein | BCP & HP |
| K.LVYREYTDASFTNRK.E | 97 kDa protein | BCP & HP |
| K.M*HAINGR.M | 97 kDa protein | BCP & HP |
| K.M*HSM*NGFMYGNQPGLTM*CK.G | 97 kDa protein | BCP & HP |
| K.M*HSM*NGFM*YGNQPGLTMCK.G | 97 kDa protein | BCP & HP |
| Y.PLSIEPIGVR.F | 97 kDa protein | BCP & HP |
| R.RQSEDSTFYLGER.T | 97 kDa protein | BCP & HP |
| R.QKDVDKEFYLFPTVFDENESLLLEDNIR.M | 97 kDa protein | BCP & HP |
| R.QSEDSTFYLGER.T | 97 kDa protein | BCP & HP |
| R.QYTDSTFR.V | 97 kDa protein | BCP & HP |
| R.QYTDSTFRVPVER.K | 97 kDa protein | BCP & HP |
| R.QYTDSTFRVPVERK.A | 97 kDa protein | BCP & HP |
| R.RDTANLFPQTSLTLHM*WPDTEGTFNVECLTTDHYTGGMK.Q | 97 kDa protein | BCP & HP |
| T.TAPDQVDKEDEDFQESNK.M | 97 kDa protein | BCP & HP |
| R.RDTANLFPQTSLTLHMWPDTEGTFNVECLTTDHYTGGMK.Q | 97 kDa protein | BCP & HP |
| R.VTFHNKGAYPLSIEPIGVR.F | 97 kDa protein | BCP & HP |
| R.SGAGTEDSACIPWAYYSTVDQVK.D | 97 kDa protein | BCP & HP |
| R.SGAGTEDSACIPWAYYSTVDQVKDLYSGLIGPLIVCR.R | 97 kDa protein | BCP & HP |
| R.SVPPSASHVAPTETFTYEWTVPK.E | 97 kDa protein | BCP & HP |
| R.SVPPSASHVAPTETFTYEWTVPKEVGPTNADPVCLAK.M | 97 kDa protein | BCP & HP |
| R.TPGIWLLHCHVTDHIHAGM*ETTYTVLQNEDTK.S | 97 kDa protein | BCP & HP |
| K.WYLFGMGNEVDVHAAFFHGQALTNK.N | 97 kDa protein | BCP & HP |
| R.RDTANLFPQTSLTLHMWPDTEGTFNVECLTTDHYTGGM*K.Q | 97 kDa protein | BCP & HP |
| K.DDEEFIESNK.M | 97 kDa protein | BCP & HP |
| K.YTVNQCR.R | 97 kDa protein | BCP & HP |
| K.M*HSMNGFM*YGNQPGLTM*CK.G | 97 kDa protein | BCP & HP |
| N.KNNEGTYYSPNYNPQSR.S | 97 kDa protein | BCP & HP |
| K.HRGVYSSDVFDIFPGTYQTLEM*FPR.T | 97 kDa protein | BCP & HP |
| K.GDSVVWYLFSAGNEADVHGIYFSGNTYLWR.G | 97 kDa protein | BCP & HP |
| K.GAYPLSIEPIGVR.F | 97 kDa protein | BCP & HP |
| K.ERGPEEEHLGILGPVIWAEVGDTIR.V | 97 kDa protein | BCP & HP |
| K.EFYLFPTVFDENESLLLEDNIR.M | 97 kDa protein | BCP & HP |
| K.DVDKEFYLFPTVFDENESLLLEDNIR.M | 97 kDa protein | BCP & HP |
| K.DNEDFQESNRM*YSVNGYTFGSLPGLSMCAEDR.V | 97 kDa protein | BCP & HP |
| K.DNEDFQESNR.M | 97 kDa protein | BCP & HP |
| K.DLYSGLIGPLIVCR.R | 97 kDa protein | BCP & HP |
| K.M*HSMNGFMYGNQPGLTMCK.G | 97 kDa protein | BCP & HP |
| K.DIFTGLIGPM*K.I | 97 kDa protein | BCP & HP |
| K.M*HSMNGFM*YGNQPGLTMCK.G | 97 kDa protein | BCP & HP |
| K.AGLQAFFQVQECNK.S | 97 kDa protein | BCP & HP |
| K.AEEEHLGILGPQLHADVGDKVK.I | 97 kDa protein | BCP & HP |
| K.AEEEHLGILGPQLHADVGDK.V | 97 kDa protein | BCP & HP |
| I.PWAYYSTVDQVK.D | 97 kDa protein | BCP & HP |
| I.FPGTYQTLEMFPR.T | 97 kDa protein | BCP & HP |
| H.SMNGFMYGNQPGLTMCK.G | 97 kDa protein | BCP & HP |
| H.IHAGMETTYTVLQNEDTK.S | 97 kDa protein | BCP & HP |
| F.PGTYQTLEMFPR.T | 97 kDa protein | BCP & HP |
| F.PATLFDAYMVAQNPGEWMLSCQNLNHLK.A | 97 kDa protein | BCP & HP |
| F.PATLFDAYM*VAQNPGEWMLSCQNLNHLK.A | 97 kDa protein | BCP & HP |
| D.PTKDIFTGLIGPMK.I | 97 kDa protein | BCP & HP |
| D.LYSGLIGPLIVCR.R | 97 kDa protein | BCP & HP |
| A.PDQVDKEDEDFQESNK.M | 97 kDa protein | BCP & HP |
| K.DIFTGLIGPMK.I | 97 kDa protein | BCP & HP |
| K.TYSDHPEKVNKDDEEFIESNKM*HAINGR.M | 97 kDa protein | BCP & HP |
| K.M*YYSAVEPTKDIFTGLIGPM*K.I | 97 kDa protein | BCP & HP |
| K.QKYTVNQCR.R | 97 kDa protein | BCP & HP |
| K.M*HSM*NGFMYGNQPGLTMCK.G | 97 kDa protein | BCP & HP |
| K.TYCSEPEKVDKDNEDFQESNR.M | 97 kDa protein | BCP & HP |
| K.TYSDHPEK.V | 97 kDa protein | BCP & HP |
| K.NMATRPYSIHAHGVQTESSTVTPTLPGETLTYVWK.I | 97 kDa protein | BCP & HP |
| K.TYSDHPEKVNKDDEEFIESNK.M | 97 kDa protein | BCP & HP |
| K.NNEGTYYSPNYNPQSR.S | 97 kDa protein | BCP & HP |
| K.TYSDHPEKVNKDDEEFIESNKMHAINGR.M | 97 kDa protein | BCP & HP |
| K.VDKDNEDFQESNR.M | 97 kDa protein | BCP & HP |
| K.VNKDDEEFIESNK.M | 97 kDa protein | BCP & HP |
| K.VNKDDEEFIESNKM*HAINGR.M | 97 kDa protein | BCP & HP |
| K.VNKDDEEFIESNKMHAINGR.M | 97 kDa protein | BCP & HP |
| K.WYLFGM*GNEVDVHAAFFHGQALTNK.N | 97 kDa protein | BCP & HP |
| K.TYSDHPEKVNK.D | 97 kDa protein | BCP & HP |
| K.MHSM*NGFMYGNQPGLTMCK.G | 97 kDa protein | BCP & HP |
| K.M*YYSAVEPTKDIFTGLIGPMK.I | 97 kDa protein | BCP & HP |
| K.MHAINGR.M | 97 kDa protein | BCP & HP |
| K.MHSM*NGFM*YGNQPGLTM*CK.G | 97 kDa protein | BCP & HP |
| K.TYCSEPEKVDK.D | 97 kDa protein | BCP & HP |
| K.MHSM*NGFMYGNQPGLTM*CK.G | 97 kDa protein | BCP & HP |
| K.MHSMNGFM*YGNQPGLTM*CK.G | 97 kDa protein | BCP & HP |
| K.MHSMNGFM*YGNQPGLTMCK.G | 97 kDa protein | BCP & HP |
| K.MHSMNGFMYGNQPGLTM*CK.G | 97 kDa protein | BCP & HP |
| K.MHSMNGFMYGNQPGLTMCK.G | 97 kDa protein | BCP & HP |
| K.MYYSAVEPTKDIFTGLIGPM*K.I | 97 kDa protein | BCP & HP |
| K.MYYSAVEPTKDIFTGLIGPMK.I | 97 kDa protein | BCP & HP |
| K.NM*ATRPYSIHAHGVQTESSTVTPTLPGETLTYVWK.I | 97 kDa protein | BCP & HP |
| K.MHSM*NGFM*YGNQPGLTMCK.G | 97 kDa protein | BCP & HP |
| K.GSPVNSLFVAPAVTPVK.S | Acid sphingomyelinase-like phosphodiesterase 3a precursor | BCP & HP |
| K.VYNAVANLWKPWLDEEAISTLR.K | Acid sphingomyelinase-like phosphodiesterase 3a precursor | BCP & HP |
| R.DLTDYLM*K.I | Actin, aortic smooth muscle | BCP & HP |
| R.AVFPSIVGRPR.H | Actin, aortic smooth muscle | BCP & HP |
| R.SYELPDGQVITIGNER.F | Actin, aortic smooth muscle | BCP & HP |
| R.MQKEITALAPSTMK.I | Actin, aortic smooth muscle | BCP & HP |
| R.M*QKEITALAPSTMK.I | Actin, aortic smooth muscle | BCP & HP |
| R.HQGVMVGMGQK.D | Actin, aortic smooth muscle | BCP & HP |
| R.HQGVMVGM*GQK.D | Actin, aortic smooth muscle | BCP & HP |
| R.HQGVM*VGMGQK.D | Actin, aortic smooth muscle | BCP & HP |
| R.HQGVM*VGM*GQK.D | Actin, aortic smooth muscle | BCP & HP |
| K.YPIEHGIITNWDDMEK.I | Actin, aortic smooth muscle | BCP & HP |
| R.DLTDYLMK.I | Actin, aortic smooth muscle | BCP & HP |
| K.AGFAGDDAPR.A | Actin, aortic smooth muscle | BCP & HP |
| K.YPIEHGIITNWDDM*EK.I | Actin, aortic smooth muscle | BCP & HP |
| K.IKIIAPPER.K | Actin, aortic smooth muscle | BCP & HP |
| K.IIAPPERK.Y | Actin, aortic smooth muscle | BCP & HP |
| K.EITALAPSTMK.I | Actin, aortic smooth muscle | BCP & HP |
| K.EITALAPSTM*K.I | Actin, aortic smooth muscle | BCP & HP |
| K.DSYVGDEAQSKR.G | Actin, aortic smooth muscle | BCP & HP |
| K.DSYVGDEAQSK.R | Actin, aortic smooth muscle | BCP & HP |
| K.EMNDAAM*FYTNR.V | Adenylyl cyclase-associated protein 1 | BCP & HP |
| R.ALLVTASQCQQPAENK.L | Adenylyl cyclase-associated protein 1 | BCP & HP |
| K.KEPAVLELEGKK.W | Adenylyl cyclase-associated protein 1 | BCP & HP |
| K.AGAAPYVQAFDSLLAGPVAEYLK.I | Adenylyl cyclase-associated protein 1 | BCP & HP |
| K.GDIGETGVPGAEGPR.G | Adiponectin precursor | BCP & HP |
| R.SAFSVGLETYVTIPNMPIR.F | Adiponectin precursor | BCP & HP |
| K.IFYNQQNHYDGSTGK.F | Adiponectin precursor | BCP & HP |
| R.NGLYADNDNDSTFTGFLLYHDTN.- | Adiponectin precursor | BCP & HP |
| K.HVCGALLK.F | Afamin precursor | BCP & HP |
| K.LVKDM*VEYKDR.C | Afamin precursor | BCP & HP |
| K.FTDSENVCQERDADPDTFFAK.F | Afamin precursor | BCP & HP |
| K.FTFEYSR.R | Afamin precursor | BCP & HP |
| K.LVKDMVEYK.D | Afamin precursor | BCP & HP |
| K.IAPQLSTEELVSLGEK.M | Afamin precursor | BCP & HP |
| K.ICAM*EGLPQK.H | Afamin precursor | BCP & HP |
| K.ICAMEGLPQK.H | Afamin precursor | BCP & HP |
| K.LKHELTDEELQSLFTNFANVVDKCCK.A | Afamin precursor | BCP & HP |
| K.KSDVGFLPPFPTLDPEEK.C | Afamin precursor | BCP & HP |
| K.HELTDEELQSLFTNFANVVDK.C | Afamin precursor | BCP & HP |
| K.AESPEVCFNEESPK.I | Afamin precursor | BCP & HP |
| K.IEFKELISLVEDVSSNYDGCCEGDVVQCIR.D | Afamin precursor | BCP & HP |
| K.CCKAESPEVCFNEESPKIGN.- | Afamin precursor | BCP & HP |
| K.HFQNLGK.D | Afamin precursor | BCP & HP |
| K.HFQNLGKDGLK.Y | Afamin precursor | BCP & HP |
| K.LVKDMVEYKDR.C | Afamin precursor | BCP & HP |
| K.LPNNVLQEK.I | Afamin precursor | BCP & HP |
| C.CEEQNKVNCLQTR.A | Afamin precursor | BCP & HP |
| K.ADKTYVPPPFSQDLFTFHADMCQSQNEELQR.K | Afamin precursor | BCP & HP |
| K.AFSSYQK.H | Afamin precursor | BCP & HP |
| K.CCKAESPEVCFNEESPK.I | Afamin precursor | BCP & HP |
| K.FTDSENVCQER.D | Afamin precursor | BCP & HP |
| K.CQAYESNR.E | Afamin precursor | BCP & HP |
| K.CQAYESNRESLLNHFLYEVAR.R | Afamin precursor | BCP & HP |
| K.DGLKYHYLIR.L | Afamin precursor | BCP & HP |
| K.DM*VEYKDR.C | Afamin precursor | BCP & HP |
| K.DMVEYK.D | Afamin precursor | BCP & HP |
| K.DMVEYKDR.C | Afamin precursor | BCP & HP |
| K.ELISLVEDVSSNYDGCCEGDVVQCIR.D | Afamin precursor | BCP & HP |
| K.AESPEVCFNEESPKIGN.- | Afamin precursor | BCP & HP |
| R.NCCNTENPPGCYR.Y | Afamin precursor | BCP & HP |
| R.ESLLNHFLYEVAR.R | Afamin precursor | BCP & HP |
| R.ESLLNHFLYEVARR.N | Afamin precursor | BCP & HP |
| R.FLVNLVK.L | Afamin precursor | BCP & HP |
| R.GQCIINSNK.D | Afamin precursor | BCP & HP |
| R.GQCIINSNKDDRPK.D | Afamin precursor | BCP & HP |
| R.GQCIINSNKDDRPKDLSLR.E | Afamin precursor | BCP & HP |
| R.HPDLSIPELLR.I | Afamin precursor | BCP & HP |
| R.IVQIYKDLLR.N | Afamin precursor | BCP & HP |
| R.EGKFTDSENVCQER.D | Afamin precursor | BCP & HP |
| R.LCFFYNKK.S | Afamin precursor | BCP & HP |
| V.PPPFSQDLFTFHADMCQSQNEELQR.K | Afamin precursor | BCP & HP |
| R.NPFVFAPTLLTVAVHFEEVAK.S | Afamin precursor | BCP & HP |
| R.RHPDLSIPELLR.I | Afamin precursor | BCP & HP |
| R.RLCFFYNKK.S | Afamin precursor | BCP & HP |
| R.RPCFESLK.A | Afamin precursor | BCP & HP |
| R.RPCFESLKADK.T | Afamin precursor | BCP & HP |
| R.TINPAVDHCCK.T | Afamin precursor | BCP & HP |
| V.SSNYDGCCEGDVVQCIR.D | Afamin precursor | BCP & HP |
| K.LKHELTDEELQSLFTNFANVVDK.C | Afamin precursor | BCP & HP |
| R.LCFFYNK.K | Afamin precursor | BCP & HP |
| K.VM*NHICSK.Q | Afamin precursor | BCP & HP |
| K.SCCEEQNKVNCLQTR.A | Afamin precursor | BCP & HP |
| K.MVQQECK.H | Afamin precursor | BCP & HP |
| K.TDRFLVNLVK.L | Afamin precursor | BCP & HP |
| R.DTSKVMNHICSK.Q | Afamin precursor | BCP & HP |
| K.TYVPPPFSQDLFTFHADMCQSQNEELQR.K | Afamin precursor | BCP & HP |
| K.SDVGFLPPFPTLDPEEK.C | Afamin precursor | BCP & HP |
| K.VM*NHICSKQDSISSK.I | Afamin precursor | BCP & HP |
| K.VMNHICSK.Q | Afamin precursor | BCP & HP |
| K.VNCLQTR.A | Afamin precursor | BCP & HP |
| L.PECSKLPNNVLQEK.I | Afamin precursor | BCP & HP |
| P.CFESLKADK.T | Afamin precursor | BCP & HP |
| R.CM*ADKTLPECSK.L | Afamin precursor | BCP & HP |
| R.CM*ADKTLPECSKLPNNVLQEK.I | Afamin precursor | BCP & HP |
| R.CMADKTLPECSK.L | Afamin precursor | BCP & HP |
| R.CMADKTLPECSKLPNNVLQEK.I | Afamin precursor | BCP & HP |
| R.DADPDTFFAK.F | Afamin precursor | BCP & HP |
| K.YHYLIR.L | Afamin precursor | BCP & HP |
| K.TLPECSKLPNNVLQEK.I | Afamin precursor | BCP & HP |
| R.DTSKVM*NHICSK.Q | Afamin precursor | BCP & HP |
| R.TPCVGDKDSSPGVR.T | aggrecan isoform 2 precursor | BCP & HP |
| K.ANEGTVGVSAATER.S | Alkaline phosphatase, tissue-nonspecific isozyme precursor | BCP & HP |
| K.LLTPITTLTSEQIQK.L | alpha 3 type VI collagen isoform 3 precursor | BCP & HP |
| R.LLPSFVSSENAFYLSPDIR.K | alpha 3 type VI collagen isoform 3 precursor | BCP & HP |
| K.ISLSPEYVFSVSTFR.E | alpha 3 type VI collagen isoform 3 precursor | BCP & HP |
| R.ALGSAIEYTIENVFESAPNPR.D | alpha 3 type VI collagen isoform 3 precursor | BCP & HP |
| R.VGVVQFSNDVFPEFYLK.T | alpha 3 type VI collagen isoform 3 precursor | BCP & HP |
| K.VCAPVLAKPGVISVM*GT.- | alpha 3 type VI collagen isoform 3 precursor | BCP & HP |
| K.IGDLHPQIVNLLK.S | alpha 3 type VI collagen isoform 3 precursor | BCP & HP |
| K.NADPAELEQIVLSPAFILAAESLPK.I | alpha 3 type VI collagen isoform 3 precursor | BCP & HP |
| R.GM*TQLQGTR.A | alpha 3 type VI collagen isoform 3 precursor | BCP & HP |
| R.VAVVQYSDR.T | alpha 3 type VI collagen isoform 3 precursor | BCP & HP |
| R.TLSGTPEESKR.D | alpha 3 type VI collagen isoform 3 precursor | BCP & HP |
| R.SSDRVDGPASNLK.Q | alpha 3 type VI collagen isoform 3 precursor | BCP & HP |
| R.SGDDVRNPSVVVKR.G | alpha 3 type VI collagen isoform 3 precursor | BCP & HP |
| R.QLTLLGGPTPNTGAALEFVLR.N | alpha 3 type VI collagen isoform 3 precursor | BCP & HP |
| R.LQPVLQPLPSPGVGGK.R | alpha 3 type VI collagen isoform 3 precursor | BCP & HP |
| R.AAPLQGMLPGLLAPLR.T | alpha 3 type VI collagen isoform 3 precursor | BCP & HP |
| R.FDEHQSKPEILNLVKR.M | alpha 3 type VI collagen isoform 3 precursor | BCP & HP |
| K.IIDELNVKPEGTR.I | alpha 3 type VI collagen isoform 3 precursor | BCP & HP |
| R.KQCDWFQGDQPTK.N | alpha 3 type VI collagen isoform 3 precursor | BCP & HP |
| R.KMKPLDGSALYTGSALDFVR.N | alpha 3 type VI collagen isoform 3 precursor | BCP & HP |
| R.ITEGVPQLLIVLTADR.S | alpha 3 type VI collagen isoform 3 precursor | BCP & HP |
| K.VCAPVLAKPGVISVMGT.- | alpha 3 type VI collagen isoform 3 precursor | BCP & HP |
| R.NILVSSAGSR.I | alpha 3 type VI collagen isoform 3 precursor | BCP & HP |
| R.DVVFLIDGSQSAGPEFQYVR.T | alpha 3 type VI collagen isoform 3 precursor | BCP & HP |
| R.LVDYLDVGFDTTR.V | alpha 3 type VI collagen isoform 3 precursor | BCP & HP |
| R.VVESLDVGQDR.V | alpha 3 type VI collagen isoform 3 precursor | BCP & HP |
| R.LRGGSPLNTGK.A | alpha 3 type VI collagen isoform 3 precursor | BCP & HP |
| R.FDEHQSKPEILNLVK.R | alpha 3 type VI collagen isoform 3 precursor | BCP & HP |
| R.QINVGNALEYVSR.N | alpha 3 type VI collagen isoform 3 precursor | BCP & HP |
| K.NWGLSVYADKPETTKEQLGEFYEALDCLR.I | Alpha-1-acid glycoprotein 1 precursor | BCP & HP |
| K.WFYIASAFR.N | Alpha-1-acid glycoprotein 1 precursor | BCP & HP |
| K.TYMLAFDVNDEKNWGLSVYADKPETTK.E | Alpha-1-acid glycoprotein 1 precursor | BCP & HP |
| R.YVGGQEHFAHLLILR.D | Alpha-1-acid glycoprotein 1 precursor | BCP & HP |
| K.SDVVYTDWK.K | Alpha-1-acid glycoprotein 1 precursor | BCP & HP |
| K.EQLGEFYEALDCLR.I | Alpha-1-acid glycoprotein 1 precursor | BCP & HP |
| K.SDVVYTDWKK.D | Alpha-1-acid glycoprotein 1 precursor | BCP & HP |
| K.TLMFGSYLDDEKNWGLSFYADKPETTK.E | Alpha-1-acid glycoprotein 2 precursor | BCP & HP |
| K.EQLGEFYEALDCLCIPR.S | Alpha-1-acid glycoprotein 2 precursor | BCP & HP |
| K.WFYIASAFR.N | Alpha-1-acid glycoprotein 2 precursor | BCP & HP |
| R.EHVAHLLFLR.D | Alpha-1-acid glycoprotein 2 precursor | BCP & HP |
| R.SDVM*YTDWKK.D | Alpha-1-acid glycoprotein 2 precursor | BCP & HP |
| R.SDVMYTDWK.K | Alpha-1-acid glycoprotein 2 precursor | BCP & HP |
| R.SDVMYTDWKK.D | Alpha-1-acid glycoprotein 2 precursor | BCP & HP |
| R.SDVMYTDWKKDK.C | Alpha-1-acid glycoprotein 2 precursor | BCP & HP |
| K.DLDSQTMMVLVNYIFFK.A | Alpha-1-antichymotrypsin precursor | BCP & HP |
| K.EQLSLLDR.F | Alpha-1-antichymotrypsin precursor | BCP & HP |
| K.EQLSLLDRFTEDAK.R | Alpha-1-antichymotrypsin precursor | BCP & HP |
| K.ITDLIKDLDSQTM*M*VLVNYIFFK.A | Alpha-1-antichymotrypsin precursor | BCP & HP |
| K.EQLSLLDRFTEDAKR.L | Alpha-1-antichymotrypsin precursor | BCP & HP |
| K.DLDSQTMM*VLVNYIFFK.A | Alpha-1-antichymotrypsin precursor | BCP & HP |
| K.DLDSQTM*MVLVNYIFFK.A | Alpha-1-antichymotrypsin precursor | BCP & HP |
| K.ITDLIKDLDSQTMM*VLVNYIFFK.A | Alpha-1-antichymotrypsin precursor | BCP & HP |
| K.AVLDVFEEGTEASAATAVK.I | Alpha-1-antichymotrypsin precursor | BCP & HP |
| K.AKWEMPFDPQDTHQSR.F | Alpha-1-antichymotrypsin precursor | BCP & HP |
| K.AKWEM*PFDPQDTHQSR.F | Alpha-1-antichymotrypsin precursor | BCP & HP |
| K.DLDSQTM*M*VLVNYIFFK.A | Alpha-1-antichymotrypsin precursor | BCP & HP |
| K.ITDLIKDLDSQTM*MVLVNYIFFK.A | Alpha-1-antichymotrypsin precursor | BCP & HP |
| R.GTHVDLGLASANVDFAFSLYK.Q | Alpha-1-antichymotrypsin precursor | BCP & HP |
| R.GLASANVDFAFSLYK.H | Alpha-1-antichymotrypsin precursor | BCP & HP |
| R.GKITDLIKDLDSQTM*M*VLVNYIFFK.A | Alpha-1-antichymotrypsin precursor | BCP & HP |
| R.DEELSCTVVELK.Y | Alpha-1-antichymotrypsin precursor | BCP & HP |
| R.FNRPFLMIIVPTDTQNIFFMSK.V | Alpha-1-antichymotrypsin precursor | BCP & HP |
| R.FNRPFLMIIVPTDTQNIFFM*SK.V | Alpha-1-antichymotrypsin precursor | BCP & HP |
| R.FNRPFLM*IIVPTDTQNIFFMSK.V | Alpha-1-antichymotrypsin precursor | BCP & HP |
| K.ADLSGITGAR.N | Alpha-1-antichymotrypsin precursor | BCP & HP |
| K.QLVLKAPDK.N | Alpha-1-antichymotrypsin precursor | BCP & HP |
| R.DYNLNDILLQLGIEEAFTSK.A | Alpha-1-antichymotrypsin precursor | BCP & HP |
| R.EIGELYLPK.F | Alpha-1-antichymotrypsin precursor | BCP & HP |
| R.FTEDAKR.L | Alpha-1-antichymotrypsin precursor | BCP & HP |
| K.WEM*PFDPQDTHQSR.F | Alpha-1-antichymotrypsin precursor | BCP & HP |
| Y.FRDEELSCTVVELK.Y | Alpha-1-antichymotrypsin precursor | BCP & HP |
| T.DFQDSAAAK.K | Alpha-1-antichymotrypsin precursor | BCP & HP |
| R.WRDSLEFR.E | Alpha-1-antichymotrypsin precursor | BCP & HP |
| R.TLNQSSDELQLSMGNAMFVK.E | Alpha-1-antichymotrypsin precursor | BCP & HP |
| R.TLNQSSDELQLSM*GNAMFVK.E | Alpha-1-antichymotrypsin precursor | BCP & HP |
| R.NLAVSQVVHKAVLDVFEEGTEASAATAVK.I | Alpha-1-antichymotrypsin precursor | BCP & HP |
| R.NLAVSQVVHK.A | Alpha-1-antichymotrypsin precursor | BCP & HP |
| R.LYGSEAFATDFQDSAAAKK.L | Alpha-1-antichymotrypsin precursor | BCP & HP |
| R.LYGSEAFATDFQDSAAAK.K | Alpha-1-antichymotrypsin precursor | BCP & HP |
| P.NSPLDEENLTQENQDRGTHVDLGLASANVDFAFSLYK.Q | Alpha-1-antichymotrypsin precursor | BCP & HP |
| P.NSPLDEENLTQENQDR.G | Alpha-1-antichymotrypsin precursor | BCP & HP |
| P.LDEENLTQENQDR.G | Alpha-1-antichymotrypsin precursor | BCP & HP |
| N.SPLDEENLTQENQDR.G | Alpha-1-antichymotrypsin precursor | BCP & HP |
| K.MEEVEAMLLPETLK.R | Alpha-1-antichymotrypsin precursor | BCP & HP |
| K.MEEVEAM*LLPETLKR.W | Alpha-1-antichymotrypsin precursor | BCP & HP |
| F.LMIIVPTDTQNIFFMSK.V | Alpha-1-antichymotrypsin precursor | BCP & HP |
| C.HPNSPLDEENLTQENQDR.G | Alpha-1-antichymotrypsin precursor | BCP & HP |
| K.ITDLIKDLDSQTMMVLVNYIFFK.A | Alpha-1-antichymotrypsin precursor | BCP & HP |
| K.ITLLSALVETR.T | Alpha-1-antichymotrypsin precursor | BCP & HP |
| K.KLINDYVK.N | Alpha-1-antichymotrypsin precursor | BCP & HP |
| K.YTGNASALFILPDQDKMEEVEAMLLPETLKR.W | Alpha-1-antichymotrypsin precursor | BCP & HP |
| K.M*EEVEAMLLPETLKR.W | Alpha-1-antichymotrypsin precursor | BCP & HP |
| K.WEMPFDPQDTHQSR.F | Alpha-1-antichymotrypsin precursor | BCP & HP |
| R.FNRPFLM*IIVPTDTQNIFFM*SK.V | Alpha-1-antichymotrypsin precursor | BCP & HP |
| K.MEEVEAMLLPETLKR.W | Alpha-1-antichymotrypsin precursor | BCP & HP |
| K.RLYGSEAFATDFQDSAAAK.K | Alpha-1-antichymotrypsin precursor | BCP & HP |
| K.RLYGSEAFATDFQDSAAAKK.L | Alpha-1-antichymotrypsin precursor | BCP & HP |
| H.PNSPLDEENLTQENQDR.G | Alpha-1-antichymotrypsin precursor | BCP & HP |
| K.LINDYVK.N | Alpha-1-antichymotrypsin precursor | BCP & HP |
| L.PAPWLSM*APVSWITPGLK.T | Alpha-1B-glycoprotein precursor | BCP & HP |
| K.LLELTGPK.S | Alpha-1B-glycoprotein precursor | BCP & HP |
| R.CLAPLEGAR.F | Alpha-1B-glycoprotein precursor | BCP & HP |
| R.CEGPIPDVTFELLREGETK.A | Alpha-1B-glycoprotein precursor | BCP & HP |
| R.ATWSGAVLAGR.D | Alpha-1B-glycoprotein precursor | BCP & HP |
| N.GWSGDSAPVELILSDETLPAPEFSPEPESGR.A | Alpha-1B-glycoprotein precursor | BCP & HP |
| R.FALVREDR.G | Alpha-1B-glycoprotein precursor | BCP & HP |
| L.PAPWLSMAPVSWITPGLK.T | Alpha-1B-glycoprotein precursor | BCP & HP |
| R.GEKELLVPR.S | Alpha-1B-glycoprotein precursor | BCP & HP |
| L.PAPEFSPEPESGR.A | Alpha-1B-glycoprotein precursor | BCP & HP |
| K.VTLTCVAPLSGVDFQLRR.G | Alpha-1B-glycoprotein precursor | BCP & HP |
| K.VTLTCVAPLSGVDFQLR.R | Alpha-1B-glycoprotein precursor | BCP & HP |
| K.SLPAPWLSMAPVSWITPGLK.T | Alpha-1B-glycoprotein precursor | BCP & HP |
| K.SLPAPWLSM*APVSWITPGLK.T | Alpha-1B-glycoprotein precursor | BCP & HP |
| K.NGVAQEPVHLDSPAIKHQFLLTGDTQGR.Y | Alpha-1B-glycoprotein precursor | BCP & HP |
| K.NGVAQEPVHLDSPAIK.H | Alpha-1B-glycoprotein precursor | BCP & HP |
| N.AVALGDGGHYTCR.Y | Alpha-1B-glycoprotein precursor | BCP & HP |
| R.SSTSPDRIFFHLNAVALGDGGHYTCR.Y | Alpha-1B-glycoprotein precursor | BCP & HP |
| T.CVAPLSGVDFQLR.R | Alpha-1B-glycoprotein precursor | BCP & HP |
| S.MAPVSWITPGLK.T | Alpha-1B-glycoprotein precursor | BCP & HP |
| R.YRSWVPHTFESELSDPVELLVAES.- | Alpha-1B-glycoprotein precursor | BCP & HP |
| R.YRLHDNQNGWSGDSAPVELILSDETLPAPEFSPEPESGR.A | Alpha-1B-glycoprotein precursor | BCP & HP |
| R.TPGAAANLELIFVGPQHAGNYR.C | Alpha-1B-glycoprotein precursor | BCP & HP |
| R.TDGEGALSEPSATVTIEELAAPPPPVLMHHGESSQVLHPGNK.V | Alpha-1B-glycoprotein precursor | BCP & HP |
| R.CRSGLSTGWTQLSK.L | Alpha-1B-glycoprotein precursor | BCP & HP |
| R.SWVPHTFESELSDPVELLVAES.- | Alpha-1B-glycoprotein precursor | BCP & HP |
| P.GAAANLELIFVGPQHAGNYR.C | Alpha-1B-glycoprotein precursor | BCP & HP |
| R.SGLSTGWTQLSK.L | Alpha-1B-glycoprotein precursor | BCP & HP |
| R.RGEKELLVPR.S | Alpha-1B-glycoprotein precursor | BCP & HP |
| R.LHDNQNGWSGDSAPVELILSDETLPAPEFSPEPESGR.A | Alpha-1B-glycoprotein precursor | BCP & HP |
| R.LETPDFQLFKNGVAQEPVHLDSPAIK.H | Alpha-1B-glycoprotein precursor | BCP & HP |
| R.LETPDFQLFK.N | Alpha-1B-glycoprotein precursor | BCP & HP |
| R.LELHVDGPPPRPQLR.A | Alpha-1B-glycoprotein precursor | BCP & HP |
| R.IFFHLNAVALGDGGHYTCR.Y | Alpha-1B-glycoprotein precursor | BCP & HP |
| R.TDGEGALSEPSATVTIEELAAPPPPVLM*HHGESSQVLHPGNK.V | Alpha-1B-glycoprotein precursor | BCP & HP |
| K.HQFLLTGDTQGR.Y | Alpha-1B-glycoprotein precursor | BCP & HP |
| R.CEGPIPDVTFELLR.E | Alpha-1B-glycoprotein precursor | BCP & HP |
| A.PLSGVDFQLR.R | Alpha-1B-glycoprotein precursor | BCP & HP |
| A.PPPPVLMHHGESSQVLHPGNK.V | Alpha-1B-glycoprotein precursor | BCP & HP |
| A.PVSWITPGLK.T | Alpha-1B-glycoprotein precursor | BCP & HP |
| A.PWLSMAPVSWITPGLK.T | Alpha-1B-glycoprotein precursor | BCP & HP |
| E.PVHLDSPAIK.H | Alpha-1B-glycoprotein precursor | BCP & HP |
| I.FVGPQHAGNYR.C | Alpha-1B-glycoprotein precursor | BCP & HP |
| K.AYLEEECPATLR.K | alpha-2-glycoprotein 1, zinc | BCP & HP |
| K.WEAEPVYVQR.A | alpha-2-glycoprotein 1, zinc | BCP & HP |
| R.AKAYLEEECPATLRK.Y | alpha-2-glycoprotein 1, zinc | BCP & HP |
| K.AYLEEECPATLRK.Y | alpha-2-glycoprotein 1, zinc | BCP & HP |
| R.QVEGMEDWKQDSQLQK.A | alpha-2-glycoprotein 1, zinc | BCP & HP |
| R.YSLTYIYTGLSK.H | alpha-2-glycoprotein 1, zinc | BCP & HP |
| R.QVEGM*EDWKQDSQLQK.A | alpha-2-glycoprotein 1, zinc | BCP & HP |
| K.QKWEAEPVYVQR.A | alpha-2-glycoprotein 1, zinc | BCP & HP |
| K.SQPMGLWRQVEGMEDWKQDSQLQKAREDIFMETLK.D | alpha-2-glycoprotein 1, zinc | BCP & HP |
| K.YYYDGKDYIEFNKEIPAWVPFDPAAQITK.Q | alpha-2-glycoprotein 1, zinc | BCP & HP |
| R.AGEVQEPELR.G | alpha-2-glycoprotein 1, zinc | BCP & HP |
| R.QDPPSVVVTSHQAPGEK.K | alpha-2-glycoprotein 1, zinc | BCP & HP |
| R.AKAYLEEECPATLR.K | alpha-2-glycoprotein 1, zinc | BCP & HP |
| K.NILDRQDPPSVVVTSHQAPGEKK.K | alpha-2-glycoprotein 1, zinc | BCP & HP |
| K.NILDRQDPPSVVVTSHQAPGEK.K | alpha-2-glycoprotein 1, zinc | BCP & HP |
| K.HVEDVPAFQALGSLNDLQFFR.Y | alpha-2-glycoprotein 1, zinc | BCP & HP |
| K.EIPAWVPFDPAAQITK.Q | alpha-2-glycoprotein 1, zinc | BCP & HP |
| K.CLAYDFYPGK.I | alpha-2-glycoprotein 1, zinc | BCP & HP |
| K.SQPMGLWR.Q | alpha-2-glycoprotein 1, zinc | BCP & HP |
| V.VQPSVGAAAGPVVPPCPGR.I | Alpha-2-HS-glycoprotein precursor | BCP & HP |
| V.PLPPSTYVEFTVSGTDCVAK.E | Alpha-2-HS-glycoprotein precursor | BCP & HP |
| V.GAAAGPVVPPCPGR.I | Alpha-2-HS-glycoprotein precursor | BCP & HP |
| R.HTFM*GVVSLGSPSGEVSHPR.K | Alpha-2-HS-glycoprotein precursor | BCP & HP |
| R.HTFMGVVSLGSPSGEVSHP.R | Alpha-2-HS-glycoprotein precursor | BCP & HP |
| R.HTFMGVVSLGSPSGEVSHPR.K | Alpha-2-HS-glycoprotein precursor | BCP & HP |
| R.QLKEHAVEGDCDFQLLK.L | Alpha-2-HS-glycoprotein precursor | BCP & HP |
| R.QLKEHAVEGDCDFQLLKLDGK.F | Alpha-2-HS-glycoprotein precursor | BCP & HP |
| K.EATEAAKCNLLAEK.Q | Alpha-2-HS-glycoprotein precursor | BCP & HP |
| R.QPNCDDPETEEAALVAIDYINQNLPWGYKHTLNQIDEVK.V | Alpha-2-HS-glycoprotein precursor | BCP & HP |
| E.IDTLETTCHVLDPTPVAR.C | Alpha-2-HS-glycoprotein precursor | BCP & HP |
| P.HGPGLIYRQPNCDDPETEEAALVAIDYINQNLPWGYK.H | Alpha-2-HS-glycoprotein precursor | BCP & HP |
| R.QPNCDDPETEEAALVAIDYINQNLPWGYK.H | Alpha-2-HS-glycoprotein precursor | BCP & HP |
| K.EHAVEGDCDFQLLKLDGK.F | Alpha-2-HS-glycoprotein precursor | BCP & HP |
| K.EHAVEGDCDFQLLK.L | Alpha-2-HS-glycoprotein precursor | BCP & HP |
| A.IDYINQNLPWGYK.H | Alpha-2-HS-glycoprotein precursor | BCP & HP |
| D.PETEEAALVAIDYINQNLPWGYK.H | Alpha-2-HS-glycoprotein precursor | BCP & HP |
| K.QYGFCK.A | Alpha-2-HS-glycoprotein precursor | BCP & HP |
| G.AAAGPVVPPCPGR.I | Alpha-2-HS-glycoprotein precursor | BCP & HP |
| R.AQLVPLPPSTYVEFTVSGTDCVAKEATEAAK.C | Alpha-2-HS-glycoprotein precursor | BCP & HP |
| I.DYINQNLPWGYK.H | Alpha-2-HS-glycoprotein precursor | BCP & HP |
| P.NCDDPETEEAALVAIDYINQNLPWGYK.H | Alpha-2-HS-glycoprotein precursor | BCP & HP |
| K.AALAAFNAQNNGSNFQLEEISR.A | Alpha-2-HS-glycoprotein precursor | BCP & HP |
| K.CDSSPDSAEDVR.K | Alpha-2-HS-glycoprotein precursor | BCP & HP |
| K.CDSSPDSAEDVRK.V | Alpha-2-HS-glycoprotein precursor | BCP & HP |
| K.CNLLAEKQYGFCK.A | Alpha-2-HS-glycoprotein precursor | BCP & HP |
| R.AQLVPLPPSTYVEFTVSGTDCVAK.E | Alpha-2-HS-glycoprotein precursor | BCP & HP |
| D.DPETEEAALVAIDYINQNLPWGYK.H | Alpha-2-HS-glycoprotein precursor | BCP & HP |
| V.AIDYINQNLPWGYK.H | Alpha-2-HS-glycoprotein precursor | BCP & HP |
| S.APHGPGLIYRQPNCDDPETEEAALVAIDYINQNLPWGYK.H | Alpha-2-HS-glycoprotein precursor | BCP & HP |
| L.PPSTYVEFTVSGTDCVAK.E | Alpha-2-HS-glycoprotein precursor | BCP & HP |
| N.CDDPETEEAALVAIDYINQNLPWGYK.H | Alpha-2-HS-glycoprotein precursor | BCP & HP |
| S.PSGEVSHPR.K | Alpha-2-HS-glycoprotein precursor | BCP & HP |
| K.HTLNQIDEVK.V | Alpha-2-HS-glycoprotein precursor | BCP & HP |
| K.HTLNQIDEVKVWPQQPSGELFEIEIDTLETTCHVLDPTPVAR.C | Alpha-2-HS-glycoprotein precursor | BCP & HP |
| K.LDGKFSVVYAK.C | Alpha-2-HS-glycoprotein precursor | BCP & HP |
| R.TVVQPSVGAAAGPVVPPCPGR.I | Alpha-2-HS-glycoprotein precursor | BCP & HP |
| K.FSVVYAK.C | Alpha-2-HS-glycoprotein precursor | BCP & HP |
| T.VVQPSVGAAAGPVVPPCPGR.I | Alpha-2-HS-glycoprotein precursor | BCP & HP |
| K.VWPQQPSGELFEIEIDTLETTCHVLDPTPVAR.C | Alpha-2-HS-glycoprotein precursor | BCP & HP |
| Q.PSVGAAAGPVVPPCPGR.I | Alpha-2-HS-glycoprotein precursor | BCP & HP |
| K.YDVENCLANK.V | Alpha-2-macroglobulin precursor | BCP & HP |
| K.LSFVKVDSHFR.Q | Alpha-2-macroglobulin precursor | BCP & HP |
| K.MVSGFIPLKPTVK.M | Alpha-2-macroglobulin precursor | BCP & HP |
| K.MCPQLQQYEMHGPEGLR.V | Alpha-2-macroglobulin precursor | BCP & HP |
| K.MCPQLQQYEM*HGPEGLR.V | Alpha-2-macroglobulin precursor | BCP & HP |
| K.M*VSGFIPLKPTVK.M | Alpha-2-macroglobulin precursor | BCP & HP |
| K.M*CPQLQQYEMHGPEGLR.V | Alpha-2-macroglobulin precursor | BCP & HP |
| K.M*CPQLQQYEM*HGPEGLR.V | Alpha-2-macroglobulin precursor | BCP & HP |
| K.GVPIPNK.L | Alpha-2-macroglobulin precursor | BCP & HP |
| K.LSFYYLIM*AK.G | Alpha-2-macroglobulin precursor | BCP & HP |
| K.PQYMVLVPSLLHTETTEK.G | Alpha-2-macroglobulin precursor | BCP & HP |
| K.LPPNVVEESAR.A | Alpha-2-macroglobulin precursor | BCP & HP |
| K.LHTEAQIQEEGTVVELTGR.Q | Alpha-2-macroglobulin precursor | BCP & HP |
| K.KLSFYYLIMAK.G | Alpha-2-macroglobulin precursor | BCP & HP |
| K.KLSFYYLIM*AK.G | Alpha-2-macroglobulin precursor | BCP & HP |
| K.HYDGSYSTFGER.Y | Alpha-2-macroglobulin precursor | BCP & HP |
| K.VDLSFSPSQSLPASHAHLR.V | Alpha-2-macroglobulin precursor | BCP & HP |
| K.LSFYYLIMAK.G | Alpha-2-macroglobulin precursor | BCP & HP |
| K.SFVHLEPM*SHELPCGHTQTVQAHYILNGGTLLGLK.K | Alpha-2-macroglobulin precursor | BCP & HP |
| K.YNILPEKEEFPFALGVQTLPQTCDEPK.A | Alpha-2-macroglobulin precursor | BCP & HP |
| K.SSSNEEVMFLTVQVK.G | Alpha-2-macroglobulin precursor | BCP & HP |
| K.SSSNEEVM*FLTVQVK.G | Alpha-2-macroglobulin precursor | BCP & HP |
| K.SLNEEAVKK.D | Alpha-2-macroglobulin precursor | BCP & HP |
| K.SKAIGYLNTGYQR.Q | Alpha-2-macroglobulin precursor | BCP & HP |
| K.SIYKPGQTVK.F | Alpha-2-macroglobulin precursor | BCP & HP |
| K.NEDSLVFVQTDK.S | Alpha-2-macroglobulin precursor | BCP & HP |
| K.SFVHLEPMSHELPCGHTQTVQAHYILNGGTLLGLK.K | Alpha-2-macroglobulin precursor | BCP & HP |
| K.NEDSLVFVQTDKSIYKPGQTVK.F | Alpha-2-macroglobulin precursor | BCP & HP |
| K.SDIAPVAR.L | Alpha-2-macroglobulin precursor | BCP & HP |
| K.QQNAQGGFSSTQDTVVALHALSKYGAATFTR.T | Alpha-2-macroglobulin precursor | BCP & HP |
| K.QQNAQGGFSSTQDTVVALHALSK.Y | Alpha-2-macroglobulin precursor | BCP & HP |
| K.QFSFPLSSEPFQGSYK.V | Alpha-2-macroglobulin precursor | BCP & HP |
| K.QEDMKGHFSISIPVKSDIAPVAR.L | Alpha-2-macroglobulin precursor | BCP & HP |
| K.QEDM*KGHFSISIPVKSDIAPVAR.L | Alpha-2-macroglobulin precursor | BCP & HP |
| K.GPTQEFKK.R | Alpha-2-macroglobulin precursor | BCP & HP |
| K.SGGRTEHPFTVEEFVLPK.F | Alpha-2-macroglobulin precursor | BCP & HP |
| G.KPQYMVLVPSLLHTETTEK.G | Alpha-2-macroglobulin precursor | BCP & HP |
| K.DLTGFPGPLNDQDDEDCINR.H | Alpha-2-macroglobulin precursor | BCP & HP |
| K.ATVLNYLPK.C | Alpha-2-macroglobulin precursor | BCP & HP |
| K.ALLAYAFALAGNQDKRK.E | Alpha-2-macroglobulin precursor | BCP & HP |
| K.ALLAYAFALAGNQDKR.K | Alpha-2-macroglobulin precursor | BCP & HP |
| K.ALLAYAFALAGNQDK.R | Alpha-2-macroglobulin precursor | BCP & HP |
| K.AIGYLNTGYQR.Q | Alpha-2-macroglobulin precursor | BCP & HP |
| K.GVPIPNKVIFIR.G | Alpha-2-macroglobulin precursor | BCP & HP |
| K.AAQVTIQSSGTFSSK.F | Alpha-2-macroglobulin precursor | BCP & HP |
| K.DMYSFLEDM*GLK.A | Alpha-2-macroglobulin precursor | BCP & HP |
| F.TDLEAENDVLHCVAFAVPK.S | Alpha-2-macroglobulin precursor | BCP & HP |
| F.PLSSEPFQGSYK.V | Alpha-2-macroglobulin precursor | BCP & HP |
| E.FAIAEYNAPCSK.D | Alpha-2-macroglobulin precursor | BCP & HP |
| C.PQLQQYEMHGPEGLR.V | Alpha-2-macroglobulin precursor | BCP & HP |
| A.SVSGKPQYMVLVPSLLHTETTEK.G | Alpha-2-macroglobulin precursor | BCP & HP |
| A.SVSGKPQYM*VLVPSLLHTETTEK.G | Alpha-2-macroglobulin precursor | BCP & HP |
| K.AGAFCLSEDAGLGISSTASLR.A | Alpha-2-macroglobulin precursor | BCP & HP |
| K.ETTFNSLLCPSGGEVSEELSLKLPPNVVEESAR.A | Alpha-2-macroglobulin precursor | BCP & HP |
| K.GHFSISIPVKSDIAPVAR.L | Alpha-2-macroglobulin precursor | BCP & HP |
| K.GHFSISIPVK.S | Alpha-2-macroglobulin precursor | BCP & HP |
| K.FSGQLNSHGCFYQQVK.T | Alpha-2-macroglobulin precursor | BCP & HP |
| K.FRVVSMDENFHPLNELIPLVYIQDPK.G | Alpha-2-macroglobulin precursor | BCP & HP |
| K.FRVVSM*DENFHPLNELIPLVYIQDPK.G | Alpha-2-macroglobulin precursor | BCP & HP |
| K.FQVDNNNRLLLQQVSLPELPGEYSMK.V | Alpha-2-macroglobulin precursor | BCP & HP |
| K.DM*YSFLEDM*GLK.A | Alpha-2-macroglobulin precursor | BCP & HP |
| K.FEVQVTVPK.I | Alpha-2-macroglobulin precursor | BCP & HP |
| K.DM*YSFLEDMGLK.A | Alpha-2-macroglobulin precursor | BCP & HP |
| K.ETTFNSLLCPSGGEVSEELSLK.L | Alpha-2-macroglobulin precursor | BCP & HP |
| K.EQAPHCICANGR.Q | Alpha-2-macroglobulin precursor | BCP & HP |
| K.DTVIKPLLVEPEGLEKETTFNSLLCPSGGEVSEELSLK.L | Alpha-2-macroglobulin precursor | BCP & HP |
| K.DTVIKPLLVEPEGLEK.E | Alpha-2-macroglobulin precursor | BCP & HP |
| K.DNSVHWERPQKPK.A | Alpha-2-macroglobulin precursor | BCP & HP |
| K.DMYSFLEDMGLK.A | Alpha-2-macroglobulin precursor | BCP & HP |
| K.VSNQTLSLFFTVLQDVPVR.D | Alpha-2-macroglobulin precursor | BCP & HP |
| K.FQVDNNNR.L | Alpha-2-macroglobulin precursor | BCP & HP |
| R.SASNMAIVDVKMVSGFIPLKPTVK.M | Alpha-2-macroglobulin precursor | BCP & HP |
| R.TGKAAQVTIQSSGTFSSK.F | Alpha-2-macroglobulin precursor | BCP & HP |
| R.TEVSSNHVLIYLDK.V | Alpha-2-macroglobulin precursor | BCP & HP |
| R.TEHPFTVEEFVLPKFEVQVTVPK.I | Alpha-2-macroglobulin precursor | BCP & HP |
| R.TEHPFTVEEFVLPK.F | Alpha-2-macroglobulin precursor | BCP & HP |
| R.SSGSLLNNAIK.G | Alpha-2-macroglobulin precursor | BCP & HP |
| R.SPCYGYQWVSEEHEEAHHTAYLVFSPSK.S | Alpha-2-macroglobulin precursor | BCP & HP |
| R.NALFCLESAWK.T | Alpha-2-macroglobulin precursor | BCP & HP |
| R.SLFTDLEAENDVLHCVAF.A | Alpha-2-macroglobulin precursor | BCP & HP |
| R.TGTHGLLVKQEDMK.G | Alpha-2-macroglobulin precursor | BCP & HP |
| R.SASNMAIVDVK.M | Alpha-2-macroglobulin precursor | BCP & HP |
| R.SASNM*AIVDVK.M | Alpha-2-macroglobulin precursor | BCP & HP |
| R.QTVSWAVTPK.S | Alpha-2-macroglobulin precursor | BCP & HP |
| R.QLNYKHYDGSYSTFGER.Y | Alpha-2-macroglobulin precursor | BCP & HP |
| R.QGIPFFGQVR.L | Alpha-2-macroglobulin precursor | BCP & HP |
| K.TAQEGDHGSHVYTK.A | Alpha-2-macroglobulin precursor | BCP & HP |
| R.SLFTDLEAENDVLHCVAFAVPK.S | Alpha-2-macroglobulin precursor | BCP & HP |
| R.VTAAPQSVCALR.A | Alpha-2-macroglobulin precursor | BCP & HP |
| V.SGKPQYMVLVPSLLHTETTEK.G | Alpha-2-macroglobulin precursor | BCP & HP |
| V.PSLLHTETTEK.G | Alpha-2-macroglobulin precursor | BCP & HP |
| T.PVSSTNEKDMYSFLEDMGLK.A | Alpha-2-macroglobulin precursor | BCP & HP |
| S.FSPSQSLPASHAHLR.V | Alpha-2-macroglobulin precursor | BCP & HP |
| R.YGAATFTR.T | Alpha-2-macroglobulin precursor | BCP & HP |
| R.VVSMDENFHPLNELIPLVYIQDPKGNR.I | Alpha-2-macroglobulin precursor | BCP & HP |
| R.TGTHGLLVK.Q | Alpha-2-macroglobulin precursor | BCP & HP |
| R.VVSM*DENFHPLNELIPLVYIQDPK.G | Alpha-2-macroglobulin precursor | BCP & HP |
| R.TGTHGLLVKQEDM*K.G | Alpha-2-macroglobulin precursor | BCP & HP |
| R.VSVQLEASPAFLAVPVEKEQAPHCICANGR.Q | Alpha-2-macroglobulin precursor | BCP & HP |
| R.VSVQLEASPAFLAVPVEK.E | Alpha-2-macroglobulin precursor | BCP & HP |
| R.VGFYESDVMGR.G | Alpha-2-macroglobulin precursor | BCP & HP |
| R.VGFYESDVM*GR.G | Alpha-2-macroglobulin precursor | BCP & HP |
| R.TTVMVKNEDSLVFVQTDK.S | Alpha-2-macroglobulin precursor | BCP & HP |
| R.TTVMVK.N | Alpha-2-macroglobulin precursor | BCP & HP |
| R.LVHVEEPHTETVRK.Y | Alpha-2-macroglobulin precursor | BCP & HP |
| R.VVSMDENFHPLNELIPLVYIQDPK.G | Alpha-2-macroglobulin precursor | BCP & HP |
| L.PTGDVIGDSAK.Y | Alpha-2-macroglobulin precursor | BCP & HP |
| R.NQGNTWLTAFVLK.T | Alpha-2-macroglobulin precursor | BCP & HP |
| R.AVDQSVLLMKPDAELSASSVYNLLPEK.D | Alpha-2-macroglobulin precursor | BCP & HP |
| R.AFQPFFVELTMPYSVIRGEAFTLK.A | Alpha-2-macroglobulin precursor | BCP & HP |
| R.AFQPFFVELTMPYSVIR.G | Alpha-2-macroglobulin precursor | BCP & HP |
| R.AFQPFFVELTM*PYSVIRGEAFTLK.A | Alpha-2-macroglobulin precursor | BCP & HP |
| R.AFQPFFVELTM*PYSVIR.G | Alpha-2-macroglobulin precursor | BCP & HP |
| R.GEAFTLK.A | Alpha-2-macroglobulin precursor | BCP & HP |
| L.VPSLLHTETTEK.G | Alpha-2-macroglobulin precursor | BCP & HP |
| R.GEAFTLKATVLNYLPK.C | Alpha-2-macroglobulin precursor | BCP & HP |
| K.YSDASDCHGEDSQAFCEK.F | Alpha-2-macroglobulin precursor | BCP & HP |
| K.YDVENCLANKVDLSFSPSQSLPASHAHLR.V | Alpha-2-macroglobulin precursor | BCP & HP |
| K.VYDYYETDEFAIAEYNAPCSKDLGNA.- | Alpha-2-macroglobulin precursor | BCP & HP |
| K.VYDYYETDEFAIAEYNAPCSK.D | Alpha-2-macroglobulin precursor | BCP & HP |
| K.VTGEGCVYLQTSLK.Y | Alpha-2-macroglobulin precursor | BCP & HP |
| K.VSNQTLSLFFTVLQDVPVRDLKPAIVK.V | Alpha-2-macroglobulin precursor | BCP & HP |
| Q.PAPTSEDLTSATNIVK.W | Alpha-2-macroglobulin precursor | BCP & HP |
| R.KDTVIKPLLVEPEGLEKETTFNSLLCPSGGEVSEELSLK.L | Alpha-2-macroglobulin precursor | BCP & HP |
| R.LVHVEEPHTETVR.K | Alpha-2-macroglobulin precursor | BCP & HP |
| R.LVDGKGVPIPNKVIFIR.G | Alpha-2-macroglobulin precursor | BCP & HP |
| R.LLLQQVSLPELPGEYSMK.V | Alpha-2-macroglobulin precursor | BCP & HP |
| R.LLLQQVSLPELPGEYSM*K.V | Alpha-2-macroglobulin precursor | BCP & HP |
| R.LLIYAVLPTGDVIGDSAKYDVENCLANK.V | Alpha-2-macroglobulin precursor | BCP & HP |
| R.AYIFIDEAHITQALIWLSQR.Q | Alpha-2-macroglobulin precursor | BCP & HP |
| R.KYSDASDCHGEDSQAFCEK.F | Alpha-2-macroglobulin precursor | BCP & HP |
| Y.VLLAYLTAQPAPTSEDLTSATNIVK.W | Alpha-2-macroglobulin precursor | BCP & HP |
| R.KDTVIKPLLVEPEGLEK.E | Alpha-2-macroglobulin precursor | BCP & HP |
| R.IAQWQSFQLEGGLKQFSFPLSSEPFQGSYK.V | Alpha-2-macroglobulin precursor | BCP & HP |
| R.IAQWQSFQLEGGLK.Q | Alpha-2-macroglobulin precursor | BCP & HP |
| R.HNVYINGITYTPVSSTNEKDMYSFLEDMGLK.A | Alpha-2-macroglobulin precursor | BCP & HP |
| R.HNVYINGITYTPVSSTNEKDMYSFLEDM*GLK.A | Alpha-2-macroglobulin precursor | BCP & HP |
| R.HNVYINGITYTPVSSTNEKDM*YSFLEDMGLK.A | Alpha-2-macroglobulin precursor | BCP & HP |
| R.HNVYINGITYTPVSSTNEK.D | Alpha-2-macroglobulin precursor | BCP & HP |
| R.LLIYAVLPTGDVIGDSAK.Y | Alpha-2-macroglobulin precursor | BCP & HP |
| R.KAGTQIENIEEDFRDGLK.L | Alpha-actinin-1 | BCP & HP |
| R.VGWEQLLTTIAR.T | Alpha-actinin-1 | BCP & HP |
| R.RDQALTEEHAR.Q | Alpha-actinin-1 | BCP & HP |
| R.LAILGIHNEVSK.I | Alpha-actinin-1 | BCP & HP |
| R.ETADTDTADQVMASFK.I | Alpha-actinin-1 | BCP & HP |
| K.ICDQWDNLGALTQK.R | Alpha-actinin-1 | BCP & HP |
| K.DDPLTNLNTAFDVAEK.Y | Alpha-actinin-1 | BCP & HP |
| R.SGWAIDPFGHSPTMAYLLNR.A | Alpha-mannosidase 2 | BCP & HP |
| K.FSSPTLELQGEFSPLQSSLPCDIHLVNLR.T | Alpha-mannosidase 2 | BCP & HP |
| K.IQFGTLSDFFDALDKADETQR.D | Alpha-mannosidase 2 | BCP & HP |
| R.FYTDLNGYQIQPR.M | Alpha-mannosidase 2 | BCP & HP |
| R.DFCGCHVAWSGSQLR.L | Alpha-N-acetylglucosaminidase precursor | BCP & HP |
| R.AAAVSEAEADFYEQNSR.Y | Alpha-N-acetylglucosaminidase precursor | BCP & HP |
| R.ALAAKPGLDTYSLGGGGAAR.V | Alpha-N-acetylglucosaminidase precursor | BCP & HP |
| R.FLLGSWLEQAR.A | Alpha-N-acetylglucosaminidase precursor | BCP & HP |
| R.GSTGVAAAAGLHR.Y | Alpha-N-acetylglucosaminidase precursor | BCP & HP |
| R.KDPVPDLAAWVTSFAAR.R | Alpha-N-acetylglucosaminidase precursor | BCP & HP |
| R.YQLTLWGPEGNILDYANK.Q | Alpha-N-acetylglucosaminidase precursor | BCP & HP |
| R.AGGVLAYELLPALDEVLASDSR.F | Alpha-N-acetylglucosaminidase precursor | BCP & HP |
| R.VVAQGVGIPEDSIFTMADRGECVPGEQEPEPILIPRV.R | AMBP protein precursor | BCP & HP |
| R.EYCGVPGDGDEELLR.F | AMBP protein precursor | BCP & HP |
| R.GECVPGEQEPEPILIPR.V | AMBP protein precursor | BCP & HP |
| R.KGVCEETSGAYEK.T | AMBP protein precursor | BCP & HP |
| R.KGVCEETSGAYEKTDTDGKFLYHK.S | AMBP protein precursor | BCP & HP |
| R.M*TVSTLVLGEGATEAEISM*TSTR.W | AMBP protein precursor | BCP & HP |
| R.MTVSTLVLGEGATEAEISM*TSTR.W | AMBP protein precursor | BCP & HP |
| R.MTVSTLVLGEGATEAEISMTSTR.W | AMBP protein precursor | BCP & HP |
| R.TVAACNLPIVR.G | AMBP protein precursor | BCP & HP |
| R.VVAQGVGIPEDSIFTM*ADR.G | AMBP protein precursor | BCP & HP |
| R.VVAQGVGIPEDSIFTM*ADRGECVPGEQEPEPILIPR.V | AMBP protein precursor | BCP & HP |
| R.VVAQGVGIPEDSIFTMADRGECVPGEQEPEPILIPR.V | AMBP protein precursor | BCP & HP |
| R.GECVPGEQEPEPILIPRV.R | AMBP protein precursor | BCP & HP |
| R.VVAQGVGIPEDSIFTMADR.G | AMBP protein precursor | BCP & HP |
| K.FYSEKECR.E | AMBP protein precursor | BCP & HP |
| K.KEDSCQLGYSAGPCM*GM*TSR.Y | AMBP protein precursor | BCP & HP |
| K.GVCEETSGAYEKTDTDGKFLYHK.S | AMBP protein precursor | BCP & HP |
| K.GVCEETSGAYEKTDTDGK.F | AMBP protein precursor | BCP & HP |
| K.GVCEETSGAYEK.T | AMBP protein precursor | BCP & HP |
| R.KGVCEETSGAYEKTDTDGK.F | AMBP protein precursor | BCP & HP |
| K.KEDSCQLGYSAGPCM*GMTSR.Y | AMBP protein precursor | BCP & HP |
| K.GKCVLFPYGGCQGNGNK.F | AMBP protein precursor | BCP & HP |
| K.EDSCQLGYSAGPCMGMTSR.Y | AMBP protein precursor | BCP & HP |
| K.EDSCQLGYSAGPCMGM*TSR.Y | AMBP protein precursor | BCP & HP |
| K.EDSCQLGYSAGPCM*GM*TSR.Y | AMBP protein precursor | BCP & HP |
| K.ECLQTCR.T | AMBP protein precursor | BCP & HP |
| K.CVLFPYGGCQGNGNKFYSEKECR.E | AMBP protein precursor | BCP & HP |
| K.CVLFPYGGCQGNGNKFYSEK.E | AMBP protein precursor | BCP & HP |
| K.CVLFPYGGCQGNGNK.F | AMBP protein precursor | BCP & HP |
| K.GKCVLFPYGGCQGNGNKFYSEK.E | AMBP protein precursor | BCP & HP |
| K.KEDSCQLGYSAGPCMGMTSR.Y | AMBP protein precursor | BCP & HP |
| K.TDTDGKFLYHK.S | AMBP protein precursor | BCP & HP |
| K.WYNLAIGSTCPWLK.K | AMBP protein precursor | BCP & HP |
| K.WYNLAIGSTCPWLKK.I | AMBP protein precursor | BCP & HP |
| N.GNNFVTEKECLQTCR.T | AMBP protein precursor | BCP & HP |
| R.AFIQLWAFDAVK.G | AMBP protein precursor | BCP & HP |
| R.ETLLQDFR.V | AMBP protein precursor | BCP & HP |
| K.EDSCQLGYSAGPCM*GMTSR.Y | AMBP protein precursor | BCP & HP |
| K.KEDSCQLGYSAGPCMGM*TSR.Y | AMBP protein precursor | BCP & HP |
| R.SEYM*EGNVR.K | Aminopeptidase N | BCP & HP |
| R.RFSTEYELQQLEQFKK.D | Aminopeptidase N | BCP & HP |
| R.QYMPWEAALSSLSYFK.L | Aminopeptidase N | BCP & HP |
| R.MLSSFLSEDVFK.Q | Aminopeptidase N | BCP & HP |
| R.YLSYTLNPDLIR.K | Aminopeptidase N | BCP & HP |
| R.KVVATTQMQAADAR.K | Aminopeptidase N | BCP & HP |
| R.VTLRPYLTPNDR.G | Aminopeptidase N | BCP & HP |
| R.VMAVDALASSHPLSTPASEINTPAQISELFDAISYSK.G | Aminopeptidase N | BCP & HP |
| R.KVVATTQM*QAADAR.K | Aminopeptidase N | BCP & HP |
| R.KSFPCFDEPAMK.A | Aminopeptidase N | BCP & HP |
| R.GVGGSQPPDIDKTELVEPTEYLVVHLK.G | Aminopeptidase N | BCP & HP |
| R.DHSAIPVINR.A | Aminopeptidase N | BCP & HP |
| K.VVATTQMQAADAR.K | Aminopeptidase N | BCP & HP |
| K.VVATTQM*QAADAR.K | Aminopeptidase N | BCP & HP |
| K.QWMENPNNNPIHPNLR.S | Aminopeptidase N | BCP & HP |
| K.DNEETGFGSGTR.A | Aminopeptidase N | BCP & HP |
| K.DLTALSNMLPK.G | Aminopeptidase N | BCP & HP |
| T.TPSASATTNPASATTLDQSK.A | Aminopeptidase N | BCP & HP |
| K.EVVLQWFTENSK.- | Aminopeptidase N | BCP & HP |
| K.MLIHPTDSESFE.- | Angiopoietin-related protein 3 precursor | BCP & HP |
| R.NVKHDGIPAECTTIYNR.G | Angiopoietin-related protein 3 precursor | BCP & HP |
| F.AVYDQSATALHFLGR.V | Angiotensinogen precursor | BCP & HP |
| R.AAM*VGM*LANFLGFR.I | Angiotensinogen precursor | BCP & HP |
| H.YASDLDKVEGLTFQQNSLNWMK.K | Angiotensinogen precursor | BCP & HP |
| K.VEGLTFQQNSLNWMK.K | Angiotensinogen precursor | BCP & HP |
| R.AAM*VGMLANFLGFR.I | Angiotensinogen precursor | BCP & HP |
| R.AAMVGM*LANFLGFR.I | Angiotensinogen precursor | BCP & HP |
| R.AAMVGMLANFLGFR.I | Angiotensinogen precursor | BCP & HP |
| R.ADSQAQLLLSTVVGVFTAPGLHLK.Q | Angiotensinogen precursor | BCP & HP |
| R.FM*QAVTGWK.T | Angiotensinogen precursor | BCP & HP |
| R.FMQAVTGWK.T | Angiotensinogen precursor | BCP & HP |
| R.SLDFTELDVAAEK.I | Angiotensinogen precursor | BCP & HP |
| R.LDAHKVLSALQAVQGLLVAQGR.A | Angiotensinogen precursor | BCP & HP |
| R.VGEVLNSIFFELEADER.E | Angiotensinogen precursor | BCP & HP |
| R.VANPLSTA.- | Angiotensinogen precursor | BCP & HP |
| R.TIHLTM*PQLVLQGSYDLQDLLAQAELPAILHTELNLQK.L | Angiotensinogen precursor | BCP & HP |
| K.ALQDQLVLVAAK.L | Angiotensinogen precursor | BCP & HP |
| K.VLSALQAVQGLLVAQGR.A | Angiotensinogen precursor | BCP & HP |
| K.ALQDQLVLVAAKLDTEDKLR.A | Angiotensinogen precursor | BCP & HP |
| R.SLDFTELDVAAEKIDR.F | Angiotensinogen precursor | BCP & HP |
| K.VEGLTFQQNSLNWM*K.K | Angiotensinogen precursor | BCP & HP |
| R.SLDFTELDVAAEKIDRFMQAVTGWK.T | Angiotensinogen precursor | BCP & HP |
| K.TSPVDEKALQDQLVLVAAK.L | Angiotensinogen precursor | BCP & HP |
| K.TGCSLMGASVDSTLAFNTYVHFQGK.M | Angiotensinogen precursor | BCP & HP |
| K.TGCSLM*GASVDSTLAFNTYVHFQGK.M | Angiotensinogen precursor | BCP & HP |
| K.QPFVQGLALYTPVVLPR.S | Angiotensinogen precursor | BCP & HP |
| K.LDTEDKLR.A | Angiotensinogen precursor | BCP & HP |
| K.IDRFMQAVTGWK.T | Angiotensinogen precursor | BCP & HP |
| K.IDRFM*QAVTGWK.T | Angiotensinogen precursor | BCP & HP |
| K.DPTFIPAPIQAK.T | Angiotensinogen precursor | BCP & HP |
| K.ANAGKPKDPTFIPAPIQAK.T | Angiotensinogen precursor | BCP & HP |
| M.PQLVLQGSYDLQDLLAQAELPAILHTELNLQK.L | Angiotensinogen precursor | BCP & HP |
| -.DIQM*TQSPSSLPASVGDR.V | Anti-mucin1 light chain variable region (Fragment) | BCP & HP |
| R.VAEGTQVLELPFK.G | Antithrombin III variant | BCP & HP |
| K.ELFYKADGESCSASMMYQEGK.F | Antithrombin III variant | BCP & HP |
| R.VAEGTQVLELPFKGDDITMVLILPKPEK.S | Antithrombin III variant | BCP & HP |
| K.SKFSPENTR.K | Antithrombin III variant | BCP & HP |
| K.NDNDNIFLSPLSISTAFAMTK.L | Antithrombin III variant | BCP & HP |
| K.NDNDNIFLSPLSISTAFAM*TK.L | Antithrombin III variant | BCP & HP |
| K.LVSANRLFGDK.S | Antithrombin III variant | BCP & HP |
| K.LQPLDFKENAEQSR.A | Antithrombin III variant | BCP & HP |
| K.LQPLDFK.E | Antithrombin III variant | BCP & HP |
| K.LPGIVAEGRDDLYVSDAFHK.A | Antithrombin III variant | BCP & HP |
| K.LPGIVAEGR.D | Antithrombin III variant | BCP & HP |
| K.GDDITMVLILPKPEK.S | Antithrombin III variant | BCP & HP |
| K.GDDITM*VLILPKPEK.S | Antithrombin III variant | BCP & HP |
| K.EQLQDMGLVDLFSPEK.S | Antithrombin III variant | BCP & HP |
| K.SKLPGIVAEGRDDLYVSDAFHK.A | Antithrombin III variant | BCP & HP |
| K.ELFYKADGESCSASMMYQEGKFR.Y | Antithrombin III variant | BCP & HP |
| K.TSDQIHFFFAK.L | Antithrombin III variant | BCP & HP |
| K.ELFYKADGESCSASMM*YQEGK.F | Antithrombin III variant | BCP & HP |
| K.ELFYKADGESCSASM*MYQEGK.F | Antithrombin III variant | BCP & HP |
| K.ELFYKADGESCSASM*M*YQEGK.F | Antithrombin III variant | BCP & HP |
| K.AFLEVNEEGSEAAASTAVVIAGR.S | Antithrombin III variant | BCP & HP |
| K.ADGESCSASMMYQEGKFR.Y | Antithrombin III variant | BCP & HP |
| K.ADGESCSASMMYQEGK.F | Antithrombin III variant | BCP & HP |
| A.EGTQVLELPFKGDDITMVLILPKPEK.S | Antithrombin III variant | BCP & HP |
| C.HGSPVDICTAKPR.D | Antithrombin III variant | BCP & HP |
| E.LPFKGDDITMVLILPKPEK.S | Antithrombin III variant | BCP & HP |
| G.SPVDICTAKPR.D | Antithrombin III variant | BCP & HP |
| K.ADGESCSASM*M*YQEGK.F | Antithrombin III variant | BCP & HP |
| K.ADGESCSASM*MYQEGK.F | Antithrombin III variant | BCP & HP |
| K.EQLQDM*GLVDLFSPEK.S | Antithrombin III variant | BCP & HP |
| R.FRIEDGFSLK.E | Antithrombin III variant | BCP & HP |
| V.AEGTQVLELPFKGDDITMVLILPKPEK.S | Antithrombin III variant | BCP & HP |
| R.VAEGTQVLELPFKGDDITM*VLILPKPEK.S | Antithrombin III variant | BCP & HP |
| K.ADGESCSASMM*YQEGK.F | Antithrombin III variant | BCP & HP |
| R.SLNPNRVTFK.A | Antithrombin III variant | BCP & HP |
| R.RVAEGTQVLELPFKGDDITMVLILPKPEK.S | Antithrombin III variant | BCP & HP |
| R.RVAEGTQVLELPFKGDDITM*VLILPKPEK.S | Antithrombin III variant | BCP & HP |
| R.RVAEGTQVLELPFK.G | Antithrombin III variant | BCP & HP |
| R.ITDVIPSEAINELTVLVLVNTIYFK.G | Antithrombin III variant | BCP & HP |
| R.IEDGFSLKEQLQDMGLVDLFSPEK.S | Antithrombin III variant | BCP & HP |
| R.IEDGFSLKEQLQDM*GLVDLFSPEK.S | Antithrombin III variant | BCP & HP |
| R.IEDGFSLK.E | Antithrombin III variant | BCP & HP |
| K.SKLPGIVAEGR.D | Antithrombin III variant | BCP & HP |
| R.FRIEDGFSLKEQLQDM*GLVDLFSPEK.S | Antithrombin III variant | BCP & HP |
| R.EVPLNTIIFM*GR.V | Antithrombin III variant | BCP & HP |
| K.VEKELTPEVLQEWLDELEEM*M*LVVHM*PR.F | Antithrombin III variant | BCP & HP |
| Q.VLELPFKGDDITMVLILPKPEK.S | Antithrombin III variant | BCP & HP |
| R.DDLYVSDAFHK.A | Antithrombin III variant | BCP & HP |
| R.DIPM*NPM*CIYR.S | Antithrombin III variant | BCP & HP |
| R.DIPM*NPMCIYR.S | Antithrombin III variant | BCP & HP |
| R.FRIEDGFSLKEQLQDMGLVDLFSPEK.S | Antithrombin III variant | BCP & HP |
| R.DIPMNPMCIYR.S | Antithrombin III variant | BCP & HP |
| V.PLNTIIFMGR.V | Antithrombin III variant | BCP & HP |
| R.EVPLNTIIFMGR.V | Antithrombin III variant | BCP & HP |
| R.EVPLNTIIFMGRVANPCVK.- | Antithrombin III variant | BCP & HP |
| R.FATTFYQHLADSK.N | Antithrombin III variant | BCP & HP |
| R.FATTFYQHLADSKNDNDNIFLSPLSISTAFAM*TK.L | Antithrombin III variant | BCP & HP |
| R.FATTFYQHLADSKNDNDNIFLSPLSISTAFAMTK.L | Antithrombin III variant | BCP & HP |
| R.DIPMNPM*CIYR.S | Antithrombin III variant | BCP & HP |
| P.EQSHVVQDCYHGDGQSYR.G | Apolipoprotein | BCP & HP |
| R.RIPLYYPNAGLTR.N | Apolipoprotein | BCP & HP |
| R.TCQSWSSM*TPHR.H | Apolipoprotein | BCP & HP |
| R.TECYITGWGETQGTFGTGLLK.E | Apolipoprotein | BCP & HP |
| R.TPAYYPNAGLIK.N | Apolipoprotein | BCP & HP |
| R.TPENYPNAGLTENYCR.N | Apolipoprotein | BCP & HP |
| R.NPDPVAAPYCYTR.D | Apolipoprotein | BCP & HP |
| R.TPENYPNDGLTM*NYCR.N | Apolipoprotein | BCP & HP |
| R.TPENYPNDGLTMNYCR.N | Apolipoprotein | BCP & HP |
| R.TPEYYPNAGLIM*NYCR.N | Apolipoprotein | BCP & HP |
| R.TPEYYPNAGLIMNYCRNPDAVAAPYCYTR.D | Apolipoprotein | BCP & HP |
| R.TTENYPNAGLIM*NYCR.N | Apolipoprotein | BCP & HP |
| R.TTENYPNAGLIMNYCR.N | Apolipoprotein | BCP & HP |
| R.TTEYYPNGGLTR.N | Apolipoprotein | BCP & HP |
| R.TPENYPNAGLTR.N | Apolipoprotein | BCP & HP |
| K.CQSWSSM*TPHR.H | Apolipoprotein | BCP & HP |
| K.VMPACLPSPDYMVTAR.T | Apolipoprotein | BCP & HP |
| R.TPEYYPNAGLIMNYCR.N | Apolipoprotein | BCP & HP |
| R.NPDGDINGPWCYTMNPR.K | Apolipoprotein | BCP & HP |
| K.VM*PACLPSPDYMVTAR.T | Apolipoprotein | BCP & HP |
| K.CQSWSSMTPHR.H | Apolipoprotein | BCP & HP |
| R.GISSTTVTGR.T | Apolipoprotein | BCP & HP |
| R.GSFSTTVTGR.T | Apolipoprotein | BCP & HP |
| R.GTFSTTVTGR.T | Apolipoprotein | BCP & HP |
| R.GTYSTTVTGR.T | Apolipoprotein | BCP & HP |
| R.HSTFIPGTNK.W | Apolipoprotein | BCP & HP |
| R.KLFDYCDIPLCASSSFDCGKPQVEPK.K | Apolipoprotein | BCP & HP |
| R.NPDAVAAPYCYTR.D | Apolipoprotein | BCP & HP |
| K.KATTVTGTPCQEWAAQEPHR.H | Apolipoprotein | BCP & HP |
| K.KWQEEM*ELYR.Q | Apolipoprotein A-I precursor | BCP & HP |
| R.LEALKENGGAR.L | Apolipoprotein A-I precursor | BCP & HP |
| R.EQLGPVTQEFWDNLEKETEGLRQEMSK.D | Apolipoprotein A-I precursor | BCP & HP |
| R.DYVSQFEGSALGK.Q | Apolipoprotein A-I precursor | BCP & HP |
| R.AHVDALR.T | Apolipoprotein A-I precursor | BCP & HP |
| K.LREQLGPVTQEFWDNLEK.E | Apolipoprotein A-I precursor | BCP & HP |
| K.WQEEMELYR.Q | Apolipoprotein A-I precursor | BCP & HP |
| R.THLAPYSDELR.Q | Apolipoprotein A-I precursor | BCP & HP |
| K.DSGRDYVSQFEGSALGK.Q | Apolipoprotein A-I precursor | BCP & HP |
| K.DLATVYVDVLKDSGR.D | Apolipoprotein A-I precursor | BCP & HP |
| K.DLATVYVDVLK.D | Apolipoprotein A-I precursor | BCP & HP |
| K.ATEHLSTLSEK.A | Apolipoprotein A-I precursor | BCP & HP |
| K.AKVQPYLDDFQKK.W | Apolipoprotein A-I precursor | BCP & HP |
| K.AKPALEDLRQGLLPVLESFK.V | Apolipoprotein A-I precursor | BCP & HP |
| K.LLDNWDSVTSTFSK.L | Apolipoprotein A-I precursor | BCP & HP |
| R.QKVEPLRAELQEGAR.Q | Apolipoprotein A-I precursor | BCP & HP |
| R.THLAPYSDELRQR.L | Apolipoprotein A-I precursor | BCP & HP |
| R.VKDLATVYVDVLK.D | Apolipoprotein A-I precursor | BCP & HP |
| R.VKDLATVYVDVLKDSGR.D | Apolipoprotein A-I precursor | BCP & HP |
| R.VKDLATVYVDVLKDSGRDYVSQFEGSALGK.Q | Apolipoprotein A-I precursor | BCP & HP |
| K.VSFLSALEEYTKK.L | Apolipoprotein A-I precursor | BCP & HP |
| K.AKPALEDLR.Q | Apolipoprotein A-I precursor | BCP & HP |
| K.VSFLSALEEYTK.K | Apolipoprotein A-I precursor | BCP & HP |
| K.VQPYLDDFQKK.W | Apolipoprotein A-I precursor | BCP & HP |
| K.VQPYLDDFQK.K | Apolipoprotein A-I precursor | BCP & HP |
| K.VEPLRAELQEGAR.Q | Apolipoprotein A-I precursor | BCP & HP |
| K.ETEGLRQEMSKDLEEVKAK.V | Apolipoprotein A-I precursor | BCP & HP |
| K.LREQLGPVTQEFWDNLEKETEGLRQEMSK.D | Apolipoprotein A-I precursor | BCP & HP |
| K.LREQLGPVTQEFWDNLEKETEGLR.Q | Apolipoprotein A-I precursor | BCP & HP |
| R.QGLLPVLESFK.V | Apolipoprotein A-I precursor | BCP & HP |
| K.KWQEEMELYR.Q | Apolipoprotein A-I precursor | BCP & HP |
| K.EPCVESLVSQYFQTVTDYGKDLMEK.V | Apolipoprotein A-II precursor | BCP & HP |
| K.SKEQLTPLIK.K | Apolipoprotein A-II precursor | BCP & HP |
| K.KAGTELVNFLSYFVELGTQPATQ.- | Apolipoprotein A-II precursor | BCP & HP |
| R.QAKEPCVESLVSQYFQTVTDYGKDLMEK.V | Apolipoprotein A-II precursor | BCP & HP |
| K.EQLTPLIKK.A | Apolipoprotein A-II precursor | BCP & HP |
| K.SKEQLTPLIKK.A | Apolipoprotein A-II precursor | BCP & HP |
| K.SPELQAEAK.S | Apolipoprotein A-II precursor | BCP & HP |
| K.VKSPELQAEAK.S | Apolipoprotein A-II precursor | BCP & HP |
| K.EPCVESLVSQYFQTVTDYGKDLM*EK.V | Apolipoprotein A-II precursor | BCP & HP |
| K.EPCVESLVSQYFQTVTDYGK.D | Apolipoprotein A-II precursor | BCP & HP |
| V.SQYFQTVTDYGKDLMEK.V | Apolipoprotein A-II precursor | BCP & HP |
| L.VSQYFQTVTDYGKDLMEK.V | Apolipoprotein A-II precursor | BCP & HP |
| K.SELTQQLNALFQDKLGEVNTYAGDLQKK.L | apolipoprotein A-IV precursor | BCP & HP |
| R.LLPHANEVSQK.I | apolipoprotein A-IV precursor | BCP & HP |
| K.LNHQLEGLTFQM*K.K | apolipoprotein A-IV precursor | BCP & HP |
| K.LNHQLEGLTFQMK.K | apolipoprotein A-IV precursor | BCP & HP |
| K.LVPFATELHER.L | apolipoprotein A-IV precursor | BCP & HP |
| K.NAEELKAR.I | apolipoprotein A-IV precursor | BCP & HP |
| K.SELTQQLNALFQDKLGEVNTYAGDLQK.K | apolipoprotein A-IV precursor | BCP & HP |
| K.SELTQQLNALFQDK.L | apolipoprotein A-IV precursor | BCP & HP |
| R.LTPYADEFK.V | apolipoprotein A-IV precursor | BCP & HP |
| R.LTPYADEFKVK.I | apolipoprotein A-IV precursor | BCP & HP |
| R.QLTPYAQR.M | apolipoprotein A-IV precursor | BCP & HP |
| R.RQLTPYAQR.M | apolipoprotein A-IV precursor | BCP & HP |
| K.LKEEIGKELEELRAR.L | apolipoprotein A-IV precursor | BCP & HP |
| K.LKEEIGKELEELR.A | apolipoprotein A-IV precursor | BCP & HP |
| K.LGPHAGDVEGHLSFLEKDLRDK.V | apolipoprotein A-IV precursor | BCP & HP |
| K.SLAELGGHLDQQVEEFR.R | apolipoprotein A-IV precursor | BCP & HP |
| K.LGPHAGDVEGHLSFLEK.D | apolipoprotein A-IV precursor | BCP & HP |
| R.DKVNSFFSTFKEK.E | apolipoprotein A-IV precursor | BCP & HP |
| K.LGPHAGDVEGHLSFLEKDLR.D | apolipoprotein A-IV precursor | BCP & HP |
| R.DKVNSFFSTFK.E | apolipoprotein A-IV precursor | BCP & HP |
| R.SLAPYAQDTQEKLNHQLEGLTFQMKK.N | apolipoprotein A-IV precursor | BCP & HP |
| K.LGEVNTYAGDLQKK.L | apolipoprotein A-IV precursor | BCP & HP |
| R.LAPLAEDVR.G | apolipoprotein A-IV precursor | BCP & HP |
| R.LAKDSEKLKEEIGKELEELR.A | apolipoprotein A-IV precursor | BCP & HP |
| R.ISASAEELRQR.L | apolipoprotein A-IV precursor | BCP & HP |
| R.ISASAEELR.Q | apolipoprotein A-IV precursor | BCP & HP |
| R.GNTEGLQK.S | apolipoprotein A-IV precursor | BCP & HP |
| R.GNLRGNTEGLQK.S | apolipoprotein A-IV precursor | BCP & HP |
| R.AEVSADQVATVMWDYFSQLSNNAK.E | apolipoprotein A-IV precursor | BCP & HP |
| R.ENADSLQASLRPHADELK.A | apolipoprotein A-IV precursor | BCP & HP |
| K.SLAELGGHLDQQVEEFRR.R | apolipoprotein A-IV precursor | BCP & HP |
| R.LEPYADQLR.T | apolipoprotein A-IV precursor | BCP & HP |
| N.ALFQDKLGEVNTYAGDLQK.K | apolipoprotein A-IV precursor | BCP & HP |
| L.PHANEVSQK.I | apolipoprotein A-IV precursor | BCP & HP |
| K.VNSFFSTFKEK.E | apolipoprotein A-IV precursor | BCP & HP |
| K.VNSFFSTFK.E | apolipoprotein A-IV precursor | BCP & HP |
| K.VKIDQTVEELRR.S | apolipoprotein A-IV precursor | BCP & HP |
| K.TLSLPELEQQQEQQQEQQQEQVQMLAPLES.- | apolipoprotein A-IV precursor | BCP & HP |
| K.TLSLPELEQQQEQQQEQQQEQVQM*LAPLES.- | apolipoprotein A-IV precursor | BCP & HP |
| R.ENADSLQASLRPHADELKAK.I | apolipoprotein A-IV precursor | BCP & HP |
| K.LGEVNTYAGDLQK.K | apolipoprotein A-IV precursor | BCP & HP |
| K.ESQDKTLSLPELEQQQEQQQEQQQEQVQMLAPLES.- | apolipoprotein A-IV precursor | BCP & HP |
| K.ESQDKTLSLPELEQQQEQQQEQQQEQVQM*LAPLES.- | apolipoprotein A-IV precursor | BCP & HP |
| K.EKESQDKTLSLPELEQQQEQQQEQQQEQVQMLAPLES.- | apolipoprotein A-IV precursor | BCP & HP |
| K.EKESQDKTLSLPELEQQQEQQQEQQQEQVQM*LAPLES.- | apolipoprotein A-IV precursor | BCP & HP |
| K.EAVEHLQK.S | apolipoprotein A-IV precursor | BCP & HP |
| K.DSEKLKEEIGKELEELRAR.L | apolipoprotein A-IV precursor | BCP & HP |
| K.DSEKLKEEIGKELEELR.A | apolipoprotein A-IV precursor | BCP & HP |
| K.DSEKLKEEIGK.E | apolipoprotein A-IV precursor | BCP & HP |
| K.IDQNVEELK.G | apolipoprotein A-IV precursor | BCP & HP |
| K.DLRDKVNSFFSTFK.E | apolipoprotein A-IV precursor | BCP & HP |
| K.ALVQQMEQLR.Q | apolipoprotein A-IV precursor | BCP & HP |
| K.ALVQQM*EQLR.Q | apolipoprotein A-IV precursor | BCP & HP |
| R.SLAPYAQDTQEKLNHQLEGLTFQM*KK.N | apolipoprotein A-IV precursor | BCP & HP |
| K.AKIDQNVEELK.G | apolipoprotein A-IV precursor | BCP & HP |
| G.ARAEVSADQVATVMWDYFSQLSNNAK.E | apolipoprotein A-IV precursor | BCP & HP |
| A.RAEVSADQVATVMWDYFSQLSNNAK.E | apolipoprotein A-IV precursor | BCP & HP |
| A.PYAQDTQEKLNHQLEGLTFQMK.K | apolipoprotein A-IV precursor | BCP & HP |
| A.EVSADQVATVMWDYFSQLSNNAK.E | apolipoprotein A-IV precursor | BCP & HP |
| A.EVSADQVATVM*WDYFSQLSNNAK.E | apolipoprotein A-IV precursor | BCP & HP |
| K.DLRDKVNSFFSTFKEK.E | apolipoprotein A-IV precursor | BCP & HP |
| R.SLAPYAQDTQEK.L | apolipoprotein A-IV precursor | BCP & HP |
| K.AKIDQNVEELKGR.L | apolipoprotein A-IV precursor | BCP & HP |
| K.IDQNVEELKGR.L | apolipoprotein A-IV precursor | BCP & HP |
| R.RSLAPYAQDTQEKLNHQLEGLTFQM*K.K | apolipoprotein A-IV precursor | BCP & HP |
| R.RVEPYGENFNK.A | apolipoprotein A-IV precursor | BCP & HP |
| R.SLAPYAQDTQEKLNHQLEGLTFQM*K.K | apolipoprotein A-IV precursor | BCP & HP |
| R.SLAPYAQDTQEKLNHQLEGLTFQMK.K | apolipoprotein A-IV precursor | BCP & HP |
| R.RSLAPYAQDTQEK.L | apolipoprotein A-IV precursor | BCP & HP |
| R.TQVSTQAEQLR.R | apolipoprotein A-IV precursor | BCP & HP |
| R.TQVSTQAEQLRR.Q | apolipoprotein A-IV precursor | BCP & HP |
| K.IDQTVEELRR.S | apolipoprotein A-IV precursor | BCP & HP |
| R.VLRENADSLQASLR.P | apolipoprotein A-IV precursor | BCP & HP |
| R.VLRENADSLQASLRPHADELK.A | apolipoprotein A-IV precursor | BCP & HP |
| R.VLRENADSLQASLRPHADELKAK.I | apolipoprotein A-IV precursor | BCP & HP |
| W.DYFSQLSNNAK.E | apolipoprotein A-IV precursor | BCP & HP |
| K.KLVPFATELHER.L | apolipoprotein A-IV precursor | BCP & HP |
| K.IGDNLRELQQR.L | apolipoprotein A-IV precursor | BCP & HP |
| R.VEPYGENFNK.A | apolipoprotein A-IV precursor | BCP & HP |
| R.RSLAPYAQDTQEKLNHQLEGLTFQMK.K | apolipoprotein A-IV precursor | BCP & HP |
| K.IDQTVEELR.R | apolipoprotein A-IV precursor | BCP & HP |
| R.ENADSLQASLRPHADELKAK.I | Apolipoprotein A-IV precursor | BCP & HP |
| K.LKEEIGKELEELR.A | Apolipoprotein A-IV precursor | BCP & HP |
| K.IDQTVEELR.R | Apolipoprotein A-IV precursor | BCP & HP |
| K.IDQTVEELRR.S | Apolipoprotein A-IV precursor | BCP & HP |
| K.IGDNLRELQQR.L | Apolipoprotein A-IV precursor | BCP & HP |
| K.KLVPFATELHER.L | Apolipoprotein A-IV precursor | BCP & HP |
| K.LGEVNTYAGDLQK.K | Apolipoprotein A-IV precursor | BCP & HP |
| K.LGEVNTYAGDLQKK.L | Apolipoprotein A-IV precursor | BCP & HP |
| K.LGPHAGDVEGHLSFLEK.D | Apolipoprotein A-IV precursor | BCP & HP |
| A.EVSADQVATVM*WDYFSQLSNNAK.E | Apolipoprotein A-IV precursor | BCP & HP |
| K.LGPHAGDVEGHLSFLEKDLRDK.V | Apolipoprotein A-IV precursor | BCP & HP |
| K.ESQDKTLSLPELEQQQEQQQEQQQEQVQMLAPLES.- | Apolipoprotein A-IV precursor | BCP & HP |
| K.LKEEIGKELEELRAR.L | Apolipoprotein A-IV precursor | BCP & HP |
| K.LNHQLEGLTFQM*K.K | Apolipoprotein A-IV precursor | BCP & HP |
| K.LNHQLEGLTFQMK.K | Apolipoprotein A-IV precursor | BCP & HP |
| K.LVPFATELHER.L | Apolipoprotein A-IV precursor | BCP & HP |
| K.NAEELKAR.I | Apolipoprotein A-IV precursor | BCP & HP |
| K.SELTQQLNALFQDK.L | Apolipoprotein A-IV precursor | BCP & HP |
| K.SELTQQLNALFQDKLGEVNTYAGDLQK.K | Apolipoprotein A-IV precursor | BCP & HP |
| K.LGPHAGDVEGHLSFLEKDLR.D | Apolipoprotein A-IV precursor | BCP & HP |
| K.DLRDKVNSFFSTFKEK.E | Apolipoprotein A-IV precursor | BCP & HP |
| A.EVSADQVATVMWDYFSQLSNNAK.E | Apolipoprotein A-IV precursor | BCP & HP |
| A.PYAQDTQEKLNHQLEGLTFQMK.K | Apolipoprotein A-IV precursor | BCP & HP |
| A.RAEVSADQVATVMWDYFSQLSNNAK.E | Apolipoprotein A-IV precursor | BCP & HP |
| G.ARAEVSADQVATVMWDYFSQLSNNAK.E | Apolipoprotein A-IV precursor | BCP & HP |
| K.AKIDQNVEELK.G | Apolipoprotein A-IV precursor | BCP & HP |
| K.AKIDQNVEELKGR.L | Apolipoprotein A-IV precursor | BCP & HP |
| K.ALVQQM*EQLR.Q | Apolipoprotein A-IV precursor | BCP & HP |
| K.IDQNVEELKGR.L | Apolipoprotein A-IV precursor | BCP & HP |
| K.DLRDKVNSFFSTFK.E | Apolipoprotein A-IV precursor | BCP & HP |
| K.IDQNVEELK.G | Apolipoprotein A-IV precursor | BCP & HP |
| K.DSEKLKEEIGK.E | Apolipoprotein A-IV precursor | BCP & HP |
| K.DSEKLKEEIGKELEELR.A | Apolipoprotein A-IV precursor | BCP & HP |
| K.DSEKLKEEIGKELEELRAR.L | Apolipoprotein A-IV precursor | BCP & HP |
| K.EAVEHLQK.S | Apolipoprotein A-IV precursor | BCP & HP |
| K.EKESQDKTLSLPELEQQQEQQQEQQQEQVQM*LAPLES.- | Apolipoprotein A-IV precursor | BCP & HP |
| K.EKESQDKTLSLPELEQQQEQQQEQQQEQVQMLAPLES.- | Apolipoprotein A-IV precursor | BCP & HP |
| K.ESQDKTLSLPELEQQQEQQQEQQQEQVQM*LAPLES.- | Apolipoprotein A-IV precursor | BCP & HP |
| K.SLAELGGHLDQQVEEFRR.R | Apolipoprotein A-IV precursor | BCP & HP |
| K.ALVQQMEQLR.Q | Apolipoprotein A-IV precursor | BCP & HP |
| R.SLAPYAQDTQEKLNHQLEGLTFQMK.K | Apolipoprotein A-IV precursor | BCP & HP |
| K.SELTQQLNALFQDKLGEVNTYAGDLQKK.L | Apolipoprotein A-IV precursor | BCP & HP |
| R.RQLTPYAQR.M | Apolipoprotein A-IV precursor | BCP & HP |
| R.RSLAPYAQDTQEK.L | Apolipoprotein A-IV precursor | BCP & HP |
| R.RSLAPYAQDTQEKLNHQLEGLTFQM*K.K | Apolipoprotein A-IV precursor | BCP & HP |
| R.RSLAPYAQDTQEKLNHQLEGLTFQMK.K | Apolipoprotein A-IV precursor | BCP & HP |
| R.RVEPYGENFNK.A | Apolipoprotein A-IV precursor | BCP & HP |
| R.SLAPYAQDTQEK.L | Apolipoprotein A-IV precursor | BCP & HP |
| R.LTPYADEFKVK.I | Apolipoprotein A-IV precursor | BCP & HP |
| R.SLAPYAQDTQEKLNHQLEGLTFQM*KK.N | Apolipoprotein A-IV precursor | BCP & HP |
| R.LTPYADEFK.V | Apolipoprotein A-IV precursor | BCP & HP |
| R.SLAPYAQDTQEKLNHQLEGLTFQMKK.N | Apolipoprotein A-IV precursor | BCP & HP |
| R.TQVNTQAEQLR.R | Apolipoprotein A-IV precursor | BCP & HP |
| R.TQVNTQAEQLRR.Q | Apolipoprotein A-IV precursor | BCP & HP |
| R.VEPYGENFNK.A | Apolipoprotein A-IV precursor | BCP & HP |
| R.VLRENADSLQASLR.P | Apolipoprotein A-IV precursor | BCP & HP |
| R.VLRENADSLQASLRPHADELK.A | Apolipoprotein A-IV precursor | BCP & HP |
| R.VLRENADSLQASLRPHADELKAK.I | Apolipoprotein A-IV precursor | BCP & HP |
| W.DYFSQLSNNAK.E | Apolipoprotein A-IV precursor | BCP & HP |
| R.SLAPYAQDTQEKLNHQLEGLTFQM*K.K | Apolipoprotein A-IV precursor | BCP & HP |
| R.ENADSLQASLRPHADELK.A | Apolipoprotein A-IV precursor | BCP & HP |
| L.RTQVNTQAEQLR.R | Apolipoprotein A-IV precursor | BCP & HP |
| K.TLSLPELEQQQEQQQEQQQEQVQM*LAPLES.- | Apolipoprotein A-IV precursor | BCP & HP |
| K.TLSLPELEQQQEQQQEQQQEQVQMLAPLES.- | Apolipoprotein A-IV precursor | BCP & HP |
| K.VKIDQTVEELRR.S | Apolipoprotein A-IV precursor | BCP & HP |
| K.VNSFFSTFK.E | Apolipoprotein A-IV precursor | BCP & HP |
| K.VNSFFSTFKEK.E | Apolipoprotein A-IV precursor | BCP & HP |
| L.PHANEVSQK.I | Apolipoprotein A-IV precursor | BCP & HP |
| R.QLTPYAQR.M | Apolipoprotein A-IV precursor | BCP & HP |
| R.DKVNSFFSTFK.E | Apolipoprotein A-IV precursor | BCP & HP |
| K.SLAELGGHLDQQVEEFR.R | Apolipoprotein A-IV precursor | BCP & HP |
| R.GNLRGNTEGLQK.S | Apolipoprotein A-IV precursor | BCP & HP |
| R.GNTEGLQK.S | Apolipoprotein A-IV precursor | BCP & HP |
| R.ISASAEELR.Q | Apolipoprotein A-IV precursor | BCP & HP |
| R.ISASAEELRQR.L | Apolipoprotein A-IV precursor | BCP & HP |
| R.LAKDSEKLKEEIGKELEELR.A | Apolipoprotein A-IV precursor | BCP & HP |
| R.LAPLAEDVR.G | Apolipoprotein A-IV precursor | BCP & HP |
| R.LEPYADQLR.T | Apolipoprotein A-IV precursor | BCP & HP |
| R.LLPHANEVSQK.I | Apolipoprotein A-IV precursor | BCP & HP |
| N.ALFQDKLGEVNTYAGDLQK.K | Apolipoprotein A-IV precursor | BCP & HP |
| R.AEVSADQVATVMWDYFSQLSNNAK.E | Apolipoprotein A-IV precursor | BCP & HP |
| R.DKVNSFFSTFKEK.E | Apolipoprotein A-IV precursor | BCP & HP |
| R.NIQEYLSILTDPDGK.G | Apolipoprotein B-100 precursor | BCP & HP |
| R.NIQEYLSILTDPDGKGK.E | Apolipoprotein B-100 precursor | BCP & HP |
| R.SEILAHWSPAK.L | Apolipoprotein B-100 precursor | BCP & HP |
| R.NIQEYLSILTDPDGKGKEK.I | Apolipoprotein B-100 precursor | BCP & HP |
| R.NNALDFVTK.S | Apolipoprotein B-100 precursor | BCP & HP |
| R.QSWSVCK.Q | Apolipoprotein B-100 precursor | BCP & HP |
| R.PLSTLISSSQSCQYTLDAK.R | Apolipoprotein B-100 precursor | BCP & HP |
| R.RNLQNNAEWVYQGAIR.Q | Apolipoprotein B-100 precursor | BCP & HP |
| R.LLDHRVPETDM*TFR.H | Apolipoprotein B-100 precursor | BCP & HP |
| R.QIDDIDVR.F | Apolipoprotein B-100 precursor | BCP & HP |
| R.NLQNNAEWVYQGAIR.Q | Apolipoprotein B-100 precursor | BCP & HP |
| R.MNFKQELNGNTK.S | Apolipoprotein B-100 precursor | BCP & HP |
| R.M*NFKQELNGNTK.S | Apolipoprotein B-100 precursor | BCP & HP |
| R.LTLDIQNKK.I | Apolipoprotein B-100 precursor | BCP & HP |
| R.LPYTIITTPPLKDFSLWEK.T | Apolipoprotein B-100 precursor | BCP & HP |
| R.LPYTIITTPPLK.D | Apolipoprotein B-100 precursor | BCP & HP |
| R.LNGESNLR.F | Apolipoprotein B-100 precursor | BCP & HP |
| R.LELELRPTGEIEQYSVSATYELQREDR.A | Apolipoprotein B-100 precursor | BCP & HP |
| R.LELELRPTGEIEQYSVSATYELQR.E | Apolipoprotein B-100 precursor | BCP & HP |
| R.LAAYLMLMR.S | Apolipoprotein B-100 precursor | BCP & HP |
| R.SEYQADYESLR.F | Apolipoprotein B-100 precursor | BCP & HP |
| R.VPSYTLILPSLELPVLHVPR.N | Apolipoprotein B-100 precursor | BCP & HP |
| R.LAAYLM*LMR.S | Apolipoprotein B-100 precursor | BCP & HP |
| R.LAAYLMLM*R.S | Apolipoprotein B-100 precursor | BCP & HP |
| R.LNTDIAGLASAIDMSTNYNSDSLHFSNVFR.S | Apolipoprotein B-100 precursor | BCP & HP |
| R.TSSFALNLPTLPEVK.F | Apolipoprotein B-100 precursor | BCP & HP |
| R.KYTYNYEAESSSGVPGTADSR.S | Apolipoprotein B-100 precursor | BCP & HP |
| R.ESDEETQIKVNWEEEAASGLLTSLKDNVPK.A | Apolipoprotein B-100 precursor | BCP & HP |
| V.PEQTIEIPSIK.F | Apolipoprotein B-100 precursor | BCP & HP |
| R.YEDGTLSLTSTSDLQSGIIK.N | Apolipoprotein B-100 precursor | BCP & HP |
| R.VSTAFVYTKNPNGYSFSIPVK.V | Apolipoprotein B-100 precursor | BCP & HP |
| R.VSTAFVYTK.N | Apolipoprotein B-100 precursor | BCP & HP |
| R.VRESDEETQIKVNWEEEAASGLLTSLKDNVPK.A | Apolipoprotein B-100 precursor | BCP & HP |
| R.VRESDEETQIK.V | Apolipoprotein B-100 precursor | BCP & HP |
| R.VPETDMTFR.H | Apolipoprotein B-100 precursor | BCP & HP |
| R.VIGNMGQTMEQLTPELK.S | Apolipoprotein B-100 precursor | BCP & HP |
| R.VIGNM*GQTMEQLTPELK.S | Apolipoprotein B-100 precursor | BCP & HP |
| R.VNDESTEGKTSYR.L | Apolipoprotein B-100 precursor | BCP & HP |
| R.TSSFALNLPTLPEVKFPEVDVLTK.Y | Apolipoprotein B-100 precursor | BCP & HP |
| R.SGVQM*NTNFFHESGLEAHVALK.A | Apolipoprotein B-100 precursor | BCP & HP |
| R.TPALHFK.S | Apolipoprotein B-100 precursor | BCP & HP |
| R.TLQGIPQMIGEVIRK.G | Apolipoprotein B-100 precursor | BCP & HP |
| R.TLQGIPQMIGEVIR.K | Apolipoprotein B-100 precursor | BCP & HP |
| R.TLQGIPQM*IGEVIR.K | Apolipoprotein B-100 precursor | BCP & HP |
| R.TLADLTLLDSPIKVPLLLSEPINIIDALEMR.D | Apolipoprotein B-100 precursor | BCP & HP |
| R.TLADLTLLDSPIKVPLLLSEPINIIDALEM*R.D | Apolipoprotein B-100 precursor | BCP & HP |
| R.TGISPLALIK.G | Apolipoprotein B-100 precursor | BCP & HP |
| R.TEHGSEMLFFGNAIEGK.S | Apolipoprotein B-100 precursor | BCP & HP |
| R.SPSQADINKIVQILPWEQNEQVK.N | Apolipoprotein B-100 precursor | BCP & HP |
| R.SPSQADINK.I | Apolipoprotein B-100 precursor | BCP & HP |
| R.SGVQMNTNFFHESGLEAHVALK.A | Apolipoprotein B-100 precursor | BCP & HP |
| R.VIGNM*GQTM*EQLTPELK.S | Apolipoprotein B-100 precursor | BCP & HP |
| K.IEGNLIFDPNNYLPK.E | Apolipoprotein B-100 precursor | BCP & HP |
| K.LATALSLSNK.F | Apolipoprotein B-100 precursor | BCP & HP |
| K.LALWGEHTGQLYSK.F | Apolipoprotein B-100 precursor | BCP & HP |
| K.KMTSNFPVDLSDYPK.S | Apolipoprotein B-100 precursor | BCP & HP |
| K.KMGLAFESTK.S | Apolipoprotein B-100 precursor | BCP & HP |
| K.KM*GLAFESTK.S | Apolipoprotein B-100 precursor | BCP & HP |
| K.KLTISEQNIQR.A | Apolipoprotein B-100 precursor | BCP & HP |
| K.KIISDYHQQFR.Y | Apolipoprotein B-100 precursor | BCP & HP |
| K.IVSLIKNLLVALK.D | Apolipoprotein B-100 precursor | BCP & HP |
| K.MGLAFESTK.S | Apolipoprotein B-100 precursor | BCP & HP |
| K.INNQLTLDSNTK.Y | Apolipoprotein B-100 precursor | BCP & HP |
| K.LDVTTSIGR.R | Apolipoprotein B-100 precursor | BCP & HP |
| K.IEDGTLASK.T | Apolipoprotein B-100 precursor | BCP & HP |
| K.IDDIWNLEVK.E | Apolipoprotein B-100 precursor | BCP & HP |
| K.IAIANIIDEIIEK.L | Apolipoprotein B-100 precursor | BCP & HP |
| K.IAELSATAQEIIKSQAIATK.K | Apolipoprotein B-100 precursor | BCP & HP |
| K.IAELSATAQEIIK.S | Apolipoprotein B-100 precursor | BCP & HP |
| K.IADFELPTIIVPEQTIEIPSIK.F | Apolipoprotein B-100 precursor | BCP & HP |
| K.HVAEAICK.E | Apolipoprotein B-100 precursor | BCP & HP |
| K.HINIDQFVR.K | Apolipoprotein B-100 precursor | BCP & HP |
| K.GNVATEISTERDLGQCDR.F | Apolipoprotein B-100 precursor | BCP & HP |
| K.IVQILPWEQNEQVK.N | Apolipoprotein B-100 precursor | BCP & HP |
| K.LNDLNSVLVMPTFHVPFTDLQVPSCK.L | Apolipoprotein B-100 precursor | BCP & HP |
| R.EYSGTIASEANTYLNSK.S | Apolipoprotein B-100 precursor | BCP & HP |
| K.M*TSNFPVDLSDYPK.S | Apolipoprotein B-100 precursor | BCP & HP |
| K.M*DM*TFSK.Q | Apolipoprotein B-100 precursor | BCP & HP |
| K.LTISEQNIQR.A | Apolipoprotein B-100 precursor | BCP & HP |
| K.LSNDM*MGSYAEM*K.F | Apolipoprotein B-100 precursor | BCP & HP |
| K.LSNDM*M*GSYAEM*K.F | Apolipoprotein B-100 precursor | BCP & HP |
| K.LSLESLTSYFSIESSTKGDVK.G | Apolipoprotein B-100 precursor | BCP & HP |
| K.LSLESLTSYFSIESSTK.G | Apolipoprotein B-100 precursor | BCP & HP |
| K.LRTSSFALNLPTLPEVKFPEVDVLTK.Y | Apolipoprotein B-100 precursor | BCP & HP |
| K.LDFREIQIYKK.L | Apolipoprotein B-100 precursor | BCP & HP |
| K.LPQQANDYLNSFNWER.Q | Apolipoprotein B-100 precursor | BCP & HP |
| K.LDNIYSSDKFYK.Q | Apolipoprotein B-100 precursor | BCP & HP |
| K.LNDLNSVLVM*PTFHVPFTDLQVPSCK.L | Apolipoprotein B-100 precursor | BCP & HP |
| K.LLSGGNTLHLVSTTKTEVIPPLIENR.Q | Apolipoprotein B-100 precursor | BCP & HP |
| K.LLSGGNTLHLVSTTK.T | Apolipoprotein B-100 precursor | BCP & HP |
| K.LKTQFNNNEYSQDLDAYNTKDK.I | Apolipoprotein B-100 precursor | BCP & HP |
| K.LIVAMSSWLQK.A | Apolipoprotein B-100 precursor | BCP & HP |
| K.LGNNPVSK.G | Apolipoprotein B-100 precursor | BCP & HP |
| K.LEIQSQVDSQHVGHSVLTAK.G | Apolipoprotein B-100 precursor | BCP & HP |
| K.LEDTPKINSR.F | Apolipoprotein B-100 precursor | BCP & HP |
| K.GMTRPLSTLISSSQSCQYTLDAK.R | Apolipoprotein B-100 precursor | BCP & HP |
| K.LRTSSFALNLPTLPEVK.F | Apolipoprotein B-100 precursor | BCP & HP |
| K.ALVEQGFTVPEIK.T | Apolipoprotein B-100 precursor | BCP & HP |
| K.GNVATEISTER.D | Apolipoprotein B-100 precursor | BCP & HP |
| K.CVQSTKPSLM*IQK.A | Apolipoprotein B-100 precursor | BCP & HP |
| K.CSLLVLENELNAELGLSGASMK.L | Apolipoprotein B-100 precursor | BCP & HP |
| K.AVSMPSFSILGSDVR.V | Apolipoprotein B-100 precursor | BCP & HP |
| K.AVSM*PSFSILGSDVR.V | Apolipoprotein B-100 precursor | BCP & HP |
| K.ATLELSPWQMSALVQVHASQPSSFHDFPDLGQEVALNANTK.N | Apolipoprotein B-100 precursor | BCP & HP |
| K.ATGVLYDYVNKYHWEHTGLTLR.E | Apolipoprotein B-100 precursor | BCP & HP |
| K.ATGVLYDYVNK.Y | Apolipoprotein B-100 precursor | BCP & HP |
| K.ATFQTPDFIVPLTDLR.I | Apolipoprotein B-100 precursor | BCP & HP |
| K.DDKHEQDM*VNGIMLSVEK.L | Apolipoprotein B-100 precursor | BCP & HP |
| K.ALYWVNGQVPDGVSK.V | Apolipoprotein B-100 precursor | BCP & HP |
| K.DDKHEQDMVNGIMLSVEK.L | Apolipoprotein B-100 precursor | BCP & HP |
| K.AHLDIAGSLEGHLR.F | Apolipoprotein B-100 precursor | BCP & HP |
| K.AGHIAWTSSGK.G | Apolipoprotein B-100 precursor | BCP & HP |
| K.AEPLAFTFSHDYK.G | Apolipoprotein B-100 precursor | BCP & HP |
| K.AEKSHDELPR.T | Apolipoprotein B-100 precursor | BCP & HP |
| K.ADYVETVLDSTCSSTVQFLEYELNVLGTHK.I | Apolipoprotein B-100 precursor | BCP & HP |
| K.AASGTTGTYQEWKDK.A | Apolipoprotein B-100 precursor | BCP & HP |
| K.AASGTTGTYQEWK.D | Apolipoprotein B-100 precursor | BCP & HP |
| A.EEEMLENVSLVCPK.D | Apolipoprotein B-100 precursor | BCP & HP |
| A.EEEM*LENVSLVCPK.D | Apolipoprotein B-100 precursor | BCP & HP |
| K.AQNLYQELLTQEGQASFQGLKDNVFDGLVR.V | Apolipoprotein B-100 precursor | BCP & HP |
| K.FPEVDVLTK.Y | Apolipoprotein B-100 precursor | BCP & HP |
| K.MTSNFPVDLSDYPK.S | Apolipoprotein B-100 precursor | BCP & HP |
| K.GM*TRPLSTLISSSQSCQYTLDAKR.K | Apolipoprotein B-100 precursor | BCP & HP |
| K.GM*TRPLSTLISSSQSCQYTLDAK.R | Apolipoprotein B-100 precursor | BCP & HP |
| K.GISTSAASPAVGTVGMDMDEDDDFSKWNFYYSPQSSPDKK.L | Apolipoprotein B-100 precursor | BCP & HP |
| K.GISTSAASPAVGTVGMDMDEDDDFSK.W | Apolipoprotein B-100 precursor | BCP & HP |
| K.GISTSAASPAVGTVGMDM*DEDDDFSK.W | Apolipoprotein B-100 precursor | BCP & HP |
| K.GISTSAASPAVGTVGM*DMDEDDDFSK.W | Apolipoprotein B-100 precursor | BCP & HP |
| K.GISTSAASPAVGTVGM*DM*DEDDDFSK.W | Apolipoprotein B-100 precursor | BCP & HP |
| K.GFEPTLEALFGK.Q | Apolipoprotein B-100 precursor | BCP & HP |
| K.CVQSTKPSLMIQK.A | Apolipoprotein B-100 precursor | BCP & HP |
| K.FSVPAGIVIPSFQALTAR.F | Apolipoprotein B-100 precursor | BCP & HP |
| K.GMTRPLSTLISSSQSCQYTLDAKR.K | Apolipoprotein B-100 precursor | BCP & HP |
| K.EVYGFNPEGK.A | Apolipoprotein B-100 precursor | BCP & HP |
| K.ESQLPTVMDFRK.F | Apolipoprotein B-100 precursor | BCP & HP |
| K.ESQLPTVMDFR.K | Apolipoprotein B-100 precursor | BCP & HP |
| K.ESQLPTVM*DFR.K | Apolipoprotein B-100 precursor | BCP & HP |
| K.ENFAGEATLQR.I | Apolipoprotein B-100 precursor | BCP & HP |
| K.EFNLQNMGLPDFHIPENLFLK.S | Apolipoprotein B-100 precursor | BCP & HP |
| K.EALKESQLPTVMDFRK.F | Apolipoprotein B-100 precursor | BCP & HP |
| K.DKDQEVLLQTFLDDASPGDKR.L | Apolipoprotein B-100 precursor | BCP & HP |
| K.DKAQNLYQELLTQEGQASFQGLKDNVFDGLVR.V | Apolipoprotein B-100 precursor | BCP & HP |
| K.FVTQAEGAK.Q | Apolipoprotein B-100 precursor | BCP & HP |
| K.WNFYYSPQSSPDKK.L | Apolipoprotein B-100 precursor | BCP & HP |
| N.NKYGMVAQVTQTLK.L | Apolipoprotein B-100 precursor | BCP & HP |
| N.NKYGM*VAQVTQTLK.L | Apolipoprotein B-100 precursor | BCP & HP |
| K.YTYNYEAESSSGVPGTADSR.S | Apolipoprotein B-100 precursor | BCP & HP |
| K.YSQPEDSLIPFFEITVPESQLTVSQFTLPK.S | Apolipoprotein B-100 precursor | BCP & HP |
| K.YNALDLTNNGK.L | Apolipoprotein B-100 precursor | BCP & HP |
| K.YHWEHTGLTLR.E | Apolipoprotein B-100 precursor | BCP & HP |
| K.YGMVAQVTQTLKLEDTPK.I | Apolipoprotein B-100 precursor | BCP & HP |
| K.YGMVAQVTQTLK.L | Apolipoprotein B-100 precursor | BCP & HP |
| K.MDM*TFSK.Q | Apolipoprotein B-100 precursor | BCP & HP |
| K.YDKNQDVHSINLPFFETLQEYFER.N | Apolipoprotein B-100 precursor | BCP & HP |
| R.ANLFNKLVTELR.G | Apolipoprotein B-100 precursor | BCP & HP |
| K.WNFYYSPQSSPDK.K | Apolipoprotein B-100 precursor | BCP & HP |
| K.VSALLTPAEQTGTWK.L | Apolipoprotein B-100 precursor | BCP & HP |
| K.VQGVEFSHR.L | Apolipoprotein B-100 precursor | BCP & HP |
| K.VPLLLSEPINIIDALEMR.D | Apolipoprotein B-100 precursor | BCP & HP |
| K.VPLLLSEPINIIDALEM*R.D | Apolipoprotein B-100 precursor | BCP & HP |
| K.VNWEEEAASGLLTSLKDNVPK.A | Apolipoprotein B-100 precursor | BCP & HP |
| K.VNWEEEAASGLLTSLK.D | Apolipoprotein B-100 precursor | BCP & HP |
| K.VLVDHFGYTKDDKHEQDMVNGIMLSVEK.L | Apolipoprotein B-100 precursor | BCP & HP |
| K.VLVDHFGYTKDDKHEQDM*VNGIMLSVEK.L | Apolipoprotein B-100 precursor | BCP & HP |
| K.YGM*VAQVTQTLK.L | Apolipoprotein B-100 precursor | BCP & HP |
| R.FLKNIILPVYDK.S | Apolipoprotein B-100 precursor | BCP & HP |
| R.KGNVATEISTER.D | Apolipoprotein B-100 precursor | BCP & HP |
| R.IYSLWEHSTK.N | Apolipoprotein B-100 precursor | BCP & HP |
| R.ITENDIQIALDDAK.I | Apolipoprotein B-100 precursor | BCP & HP |
| R.INCKVELEVPQLCSFILK.T | Apolipoprotein B-100 precursor | BCP & HP |
| R.ILGEELGFASLHDLQLLGK.L | Apolipoprotein B-100 precursor | BCP & HP |
| R.IHSGSFQSQVELSNDQEK.A | Apolipoprotein B-100 precursor | BCP & HP |
| R.IGQDGISTSATTNLK.C | Apolipoprotein B-100 precursor | BCP & HP |
| R.HSITNPLAVLCEFISQSIK.S | Apolipoprotein B-100 precursor | BCP & HP |
| R.HIQNIDIQHLAGK.L | Apolipoprotein B-100 precursor | BCP & HP |
| N.NKYGMVAQVTQTLKLEDTPK.I | Apolipoprotein B-100 precursor | BCP & HP |
| R.FSDEGTHESQISFTIEGPLTSFGLSNK.I | Apolipoprotein B-100 precursor | BCP & HP |
| R.AALGKLPQQANDYLNSFNWER.Q | Apolipoprotein B-100 precursor | BCP & HP |
| R.FFGEGTKK.M | Apolipoprotein B-100 precursor | BCP & HP |
| R.FFGEGTK.K | Apolipoprotein B-100 precursor | BCP & HP |
| R.EVGTVLSQVYSK.V | Apolipoprotein B-100 precursor | BCP & HP |
| R.EIFNMAR.D | Apolipoprotein B-100 precursor | BCP & HP |
| R.EFQVPTFTIPK.L | Apolipoprotein B-100 precursor | BCP & HP |
| R.DLKVEDIPLAR.I | Apolipoprotein B-100 precursor | BCP & HP |
| R.DAVEKPQEFTIVAFVK.Y | Apolipoprotein B-100 precursor | BCP & HP |
| R.ATLYALSHAVNNYHK.T | Apolipoprotein B-100 precursor | BCP & HP |
| K.TTLTAFGFASADLIEIGLEGK.G | Apolipoprotein B-100 precursor | BCP & HP |
| R.GIISALLVPPETEEAK.Q | Apolipoprotein B-100 precursor | BCP & HP |
| K.NRNNALDFVTK.S | Apolipoprotein B-100 precursor | BCP & HP |
| K.VLVDHFGYTK.D | Apolipoprotein B-100 precursor | BCP & HP |
| K.QVFLYPEKDEPTYILNIKR.G | Apolipoprotein B-100 precursor | BCP & HP |
| K.QVFLYPEKDEPTYILNIK.R | Apolipoprotein B-100 precursor | BCP & HP |
| K.QTVNLQLQPYSLVTTLNSDLKYNALDLTNNGK.L | Apolipoprotein B-100 precursor | BCP & HP |
| K.QTVNLQLQPYSLVTTLNSDLK.Y | Apolipoprotein B-100 precursor | BCP & HP |
| K.QGFFPDSVNK.A | Apolipoprotein B-100 precursor | BCP & HP |
| K.NTASLKYENYELTLK.S | Apolipoprotein B-100 precursor | BCP & HP |
| K.NSLKIEIPLPFGGK.S | Apolipoprotein B-100 precursor | BCP & HP |
| K.NSLFFSAQPFEITASTNNEGNLK.V | Apolipoprotein B-100 precursor | BCP & HP |
| K.SHDELPR.T | Apolipoprotein B-100 precursor | BCP & HP |
| K.NSEEFAAAM*SR.Y | Apolipoprotein B-100 precursor | BCP & HP |
| K.SISAALEHK.V | Apolipoprotein B-100 precursor | BCP & HP |
| K.NQDVHSINLPFFETLQEYFER.N | Apolipoprotein B-100 precursor | BCP & HP |
| K.NPNGYSFSIPVK.V | Apolipoprotein B-100 precursor | BCP & HP |
| K.NKADYVETVLDSTCSSTVQFLEYELNVLGTHKIEDGTLASK.T | Apolipoprotein B-100 precursor | BCP & HP |
| K.NKADYVETVLDSTCSSTVQFLEYELNVLGTHK.I | Apolipoprotein B-100 precursor | BCP & HP |
| K.NIILPVYDKSLWDFLK.L | Apolipoprotein B-100 precursor | BCP & HP |
| K.NIILPVYDK.S | Apolipoprotein B-100 precursor | BCP & HP |
| K.NHLQLEGLFFTNGEHTSK.A | Apolipoprotein B-100 precursor | BCP & HP |
| K.NFVASHIANILNSEELDIQDLKK.L | Apolipoprotein B-100 precursor | BCP & HP |
| K.NFVASHIANILNSEELDIQDLK.K | Apolipoprotein B-100 precursor | BCP & HP |
| K.NSEEFAAAMSR.Y | Apolipoprotein B-100 precursor | BCP & HP |
| K.SVGFHLPSR.E | Apolipoprotein B-100 precursor | BCP & HP |
| R.KGNVATEISTERDLGQCDR.F | Apolipoprotein B-100 precursor | BCP & HP |
| K.TSQCTLKEVYGFNPEGK.A | Apolipoprotein B-100 precursor | BCP & HP |
| K.TQFNNNEYSQDLDAYNTK.D | Apolipoprotein B-100 precursor | BCP & HP |
| K.TNPTGTQELLDIANYLM*EQIQDDCTGDEDYTYLILR.V | Apolipoprotein B-100 precursor | BCP & HP |
| K.TKNSEEFAAAMSR.Y | Apolipoprotein B-100 precursor | BCP & HP |
| K.TKNSEEFAAAM*SR.Y | Apolipoprotein B-100 precursor | BCP & HP |
| K.TGLKEFLK.T | Apolipoprotein B-100 precursor | BCP & HP |
| K.TEVIPPLIENR.Q | Apolipoprotein B-100 precursor | BCP & HP |
| K.SVSLPSLDPASAK.I | Apolipoprotein B-100 precursor | BCP & HP |
| K.RGIISALLVPPETEEAK.Q | Apolipoprotein B-100 precursor | BCP & HP |
| K.SVSDGIAALDLNAVANK.I | Apolipoprotein B-100 precursor | BCP & HP |
| K.VELEVPQLCSFILK.T | Apolipoprotein B-100 precursor | BCP & HP |
| K.STSPPKQAEAVLK.T | Apolipoprotein B-100 precursor | BCP & HP |
| K.SPAFTDLHLR.Y | Apolipoprotein B-100 precursor | BCP & HP |
| K.SNTVASLHTEKNTLELSNGVIVK.I | Apolipoprotein B-100 precursor | BCP & HP |
| K.SNTVASLHTEK.N | Apolipoprotein B-100 precursor | BCP & HP |
| K.SLWDFLK.L | Apolipoprotein B-100 precursor | BCP & HP |
| K.SLHMYANR.L | Apolipoprotein B-100 precursor | BCP & HP |
| K.SKPTVSSSMEFK.Y | Apolipoprotein B-100 precursor | BCP & HP |
| K.SKPTVSSSM*EFK.Y | Apolipoprotein B-100 precursor | BCP & HP |
| K.SKEVPEAR.A | Apolipoprotein B-100 precursor | BCP & HP |
| K.SVSDGIAALDLNAVANKIADFELPTIIVPEQTIEIPSIK.F | Apolipoprotein B-100 precursor | BCP & HP |
| G.TPDVSSALDK.L | Apolipoprotein C-I precursor | BCP & HP |
| K.LKEFGNTLEDKAR.E | Apolipoprotein C-I precursor | BCP & HP |
| P.DVSSALDKLKEFGNTLEDKAR.E | Apolipoprotein C-I precursor | BCP & HP |
| P.DVSSALDKLKEFGNTLEDK.A | Apolipoprotein C-I precursor | BCP & HP |
| K.MREWFSETFQK.V | Apolipoprotein C-I precursor | BCP & HP |
| K.M*REWFSETFQK.V | Apolipoprotein C-I precursor | BCP & HP |
| R.IKQSELSAK.M | Apolipoprotein C-I precursor | BCP & HP |
| K.LKEFGNTLEDK.A | Apolipoprotein C-I precursor | BCP & HP |
| K.EFGNTLEDKAR.E | Apolipoprotein C-I precursor | BCP & HP |
| K.EFGNTLEDK.A | Apolipoprotein C-I precursor | BCP & HP |
| G.TPDVSSALDKLKEFGNTLEDKAR.E | Apolipoprotein C-I precursor | BCP & HP |
| G.TPDVSSALDKLK.E | Apolipoprotein C-I precursor | BCP & HP |
| G.TPDVSSALDKLKEFGNTLEDK.A | Apolipoprotein C-I precursor | BCP & HP |
| K.TYLPAVDEKLR.D | Apolipoprotein C-II precursor | BCP & HP |
| G.TQQPQQDEM*PSPTFLTQVK.E | Apolipoprotein C-II precursor | BCP & HP |
| G.TQQPQQDEMPSPTFLTQVK.E | Apolipoprotein C-II precursor | BCP & HP |
| K.ESLSSYWESAK.T | Apolipoprotein C-II precursor | BCP & HP |
| K.STAAM*STYTGIFTDQVLSVLK.G | Apolipoprotein C-II precursor | BCP & HP |
| K.STAAM*STYTGIFTDQVLSVLKGEE.- | Apolipoprotein C-II precursor | BCP & HP |
| K.STAAMSTYTGIFTDQVLSVLK.G | Apolipoprotein C-II precursor | BCP & HP |
| K.STAAMSTYTGIFTDQVLSVLKGEE.- | Apolipoprotein C-II precursor | BCP & HP |
| K.TYLPAVDEK.L | Apolipoprotein C-II precursor | BCP & HP |
| K.TYLPAVDEKLRDLYSK.S | Apolipoprotein C-II precursor | BCP & HP |
| K.TAAQNLYEK.T | Apolipoprotein C-II precursor | BCP & HP |
| R.GWVTDGFSSLKDYWSTVKDK.F | Apolipoprotein C-III precursor | BCP & HP |
| R.GWVTDGFSSLKDYWSTVK.D | Apolipoprotein C-III precursor | BCP & HP |
| R.GWVTDGFSSLK.D | Apolipoprotein C-III precursor | BCP & HP |
| K.TAKDALSSVQESQVAQQAR.G | Apolipoprotein C-III precursor | BCP & HP |
| K.DYWSTVK.D | Apolipoprotein C-III precursor | BCP & HP |
| K.DKFSEFWDLDPEVRPTSAVAA.- | Apolipoprotein C-III precursor | BCP & HP |
| K.DALSSVQESQVAQQAR.G | Apolipoprotein C-III precursor | BCP & HP |
| A.SEAEDASLLSFMQGYMK.H | Apolipoprotein C-III precursor | BCP & HP |
| A.SEAEDASLLSFMQGYM*K.H | Apolipoprotein C-III precursor | BCP & HP |
| A.SEAEDASLLSFM*QGYMK.H | Apolipoprotein C-III precursor | BCP & HP |
| R.MKELLETVVNR.T | Apolipoprotein C-IV precursor | BCP & HP |
| R.GFMQTYYDDHLR.D | Apolipoprotein C-IV precursor | BCP & HP |
| R.M*KELLETVVNR.T | Apolipoprotein C-IV precursor | BCP & HP |
| R.GFMQTYYDDHLRDLGPLTK.A | Apolipoprotein C-IV precursor | BCP & HP |
| K.AWFLESK.D | Apolipoprotein C-IV precursor | BCP & HP |
| R.DLGPLTK.A | Apolipoprotein C-IV precursor | BCP & HP |
| K.CPNPPVQENFDVNKYLGR.W | Apolipoprotein D precursor | BCP & HP |
| K.KM*TVTDQVNCPK.L | Apolipoprotein D precursor | BCP & HP |
| K.KMTVTDQVNCPK.L | Apolipoprotein D precursor | BCP & HP |
| K.M*TVTDQVNCPK.L | Apolipoprotein D precursor | BCP & HP |
| K.MTVTDQVNCPK.L | Apolipoprotein D precursor | BCP & HP |
| R.WYEIEKIPTTFENGR.C | Apolipoprotein D precursor | BCP & HP |
| K.NILTSNNIDVKK.M | Apolipoprotein D precursor | BCP & HP |
| R.ERLGPLVEQGR.V | Apolipoprotein E precursor | BCP & HP |
| R.ALM*DETMK.E | Apolipoprotein E precursor | BCP & HP |
| A.KVEQAVETEPEPELR.Q | Apolipoprotein E precursor | BCP & HP |
| K.LEEQAQQIR.L | Apolipoprotein E precursor | BCP & HP |
| K.SELEEQLTPVAEETR.A | Apolipoprotein E precursor | BCP & HP |
| K.SWFEPLVEDM*QR.Q | Apolipoprotein E precursor | BCP & HP |
| K.SWFEPLVEDMQR.Q | Apolipoprotein E precursor | BCP & HP |
| K.VQAAVGTSAAPVPSDNH.- | Apolipoprotein E precursor | BCP & HP |
| R.AATVGSLAGQPLQER.A | Apolipoprotein E precursor | BCP & HP |
| R.GEVQAMLGQSTEELR.V | Apolipoprotein E precursor | BCP & HP |
| R.ALM*DETM*K.E | Apolipoprotein E precursor | BCP & HP |
| R.GEVQAMLGQSTEELRVR.L | Apolipoprotein E precursor | BCP & HP |
| R.ALMDETM*K.E | Apolipoprotein E precursor | BCP & HP |
| R.ALMDETMKELK.A | Apolipoprotein E precursor | BCP & HP |
| R.ARM*EEMGSR.T | Apolipoprotein E precursor | BCP & HP |
| R.ARMEEMGSR.T | Apolipoprotein E precursor | BCP & HP |
| R.DRLDEVKEQVAEVR.A | Apolipoprotein E precursor | BCP & HP |
| K.AYKSELEEQLTPVAEETR.A | Apolipoprotein E precursor | BCP & HP |
| R.GEVQAM*LGQSTEELR.V | Apolipoprotein E precursor | BCP & HP |
| R.AKLEEQAQQIR.L | Apolipoprotein E precursor | BCP & HP |
| R.LGADMEDVCGR.L | Apolipoprotein E precursor | BCP & HP |
| R.WVQTLSEQVQEELLSSQVTQELR.A | Apolipoprotein E precursor | BCP & HP |
| R.WELALGR.F | Apolipoprotein E precursor | BCP & HP |
| R.TRDRLDEVKEQVAEVR.A | Apolipoprotein E precursor | BCP & HP |
| R.LSKELQAAQAR.L | Apolipoprotein E precursor | BCP & HP |
| R.LQAEAFQAR.L | Apolipoprotein E precursor | BCP & HP |
| R.LLRDADDLQKR.L | Apolipoprotein E precursor | BCP & HP |
| R.LKSWFEPLVEDMQR.Q | Apolipoprotein E precursor | BCP & HP |
| R.LGPLVEQGR.V | Apolipoprotein E precursor | BCP & HP |
| R.LGADM*EDVCGR.L | Apolipoprotein E precursor | BCP & HP |
| R.LDEVKEQVAEVR.A | Apolipoprotein E precursor | BCP & HP |
| R.LAVYQAGAR.E | Apolipoprotein E precursor | BCP & HP |
| R.LKSWFEPLVEDM*QR.Q | Apolipoprotein E precursor | BCP & HP |
| R.SGVQQLIQYYQDQK.D | apolipoprotein F precursor | BCP & HP |
| K.SYDLDPGAGSLEI.- | apolipoprotein F precursor | BCP & HP |
| R.MKDGLCVPR.K | Apolipoprotein M | BCP & HP |
| K.WIYHLTEGSTDLR.T | Apolipoprotein M | BCP & HP |
| K.TELFSSSCPGGIMLNETGQGYQR.F | Apolipoprotein M | BCP & HP |
| K.SLTSCLDSK.A | Apolipoprotein M | BCP & HP |
| K.DGRSNVRMEHIADK.R | ATP-sensitive inward rectifier potassium channel 10 | BCP & HP |
| K.RAETIRFSQHAVVASHNGKPCLMIRVANMRK.S | ATP-sensitive inward rectifier potassium channel 10 | BCP & HP |
| K.NGERIEKVEHSDLSFSK.D | B2M protein | BCP & HP |
| K.SNFLNCYVSGFHPSDIEVDLLK.N | B2M protein | BCP & HP |
| K.SNFLNCYVSGFHPSDIEVDLLKNGER.I | B2M protein | BCP & HP |
| K.VEHSDLSFSK.D | B2M protein | BCP & HP |
| R.IEKVEHSDLSFSK.D | B2M protein | BCP & HP |
| R.VNHVTLSQPK.I | B2M protein | BCP & HP |
| R.CQVSGSPPHYFYWSR.E | Basement membrane-specific heparan sulfate proteoglycan core protein precursor | BCP & HP |
| R.SPGPNVAVNAK.G | Basement membrane-specific heparan sulfate proteoglycan core protein precursor | BCP & HP |
| R.VSGISM*DVAVPEETGQDPALEVEQCSCPPGYR.G | Basement membrane-specific heparan sulfate proteoglycan core protein precursor | BCP & HP |
| R.TPSGLYLGTCER.C | Basement membrane-specific heparan sulfate proteoglycan core protein precursor | BCP & HP |
| R.SQSVRPGADVTFICTAK.S | Basement membrane-specific heparan sulfate proteoglycan core protein precursor | BCP & HP |
| R.EHLLMALADLDELLIR.A | Basement membrane-specific heparan sulfate proteoglycan core protein precursor | BCP & HP |
| R.IAHVELADAGQYR.C | Basement membrane-specific heparan sulfate proteoglycan core protein precursor | BCP & HP |
| K.ESDQGAYTCEAM*NAR.G | Basement membrane-specific heparan sulfate proteoglycan core protein precursor | BCP & HP |
| K.GSVYIGGAPDVATLTGGR.F | Basement membrane-specific heparan sulfate proteoglycan core protein precursor | BCP & HP |
| K.SKSPAYTLVWTR.L | Basement membrane-specific heparan sulfate proteoglycan core protein precursor | BCP & HP |
| R.AMDFNGILTIR.N | Basement membrane-specific heparan sulfate proteoglycan core protein precursor | BCP & HP |
| R.AQAGANTRPCPS.- | Basement membrane-specific heparan sulfate proteoglycan core protein precursor | BCP & HP |
| R.ASYAQQPAESR.V | Basement membrane-specific heparan sulfate proteoglycan core protein precursor | BCP & HP |
| R.CEQCQPGYYGDAQR.G | Basement membrane-specific heparan sulfate proteoglycan core protein precursor | BCP & HP |
| R.CESCAPGYEGNPIQPGGK.C | Basement membrane-specific heparan sulfate proteoglycan core protein precursor | BCP & HP |
| R.CPPGYIGLSCQDCAPGYTR.T | Basement membrane-specific heparan sulfate proteoglycan core protein precursor | BCP & HP |
| R.DSQVPGPIGCNCDPQGSVSSQCDAAGQCQCK.A | Basement membrane-specific heparan sulfate proteoglycan core protein precursor | BCP & HP |
| R.EDGRPVPSGTQQR.H | Basement membrane-specific heparan sulfate proteoglycan core protein precursor | BCP & HP |
| R.RGSIQVDGEELVSGR.S | Basement membrane-specific heparan sulfate proteoglycan core protein precursor | BCP & HP |
| R.FLGDKVTSYGGELR.F | Basement membrane-specific heparan sulfate proteoglycan core protein precursor | BCP & HP |
| R.GHTPTQPGALNQR.Q | Basement membrane-specific heparan sulfate proteoglycan core protein precursor | BCP & HP |
| R.GPSCQDCDTGYTR.T | Basement membrane-specific heparan sulfate proteoglycan core protein precursor | BCP & HP |
| R.HQGSELHFPSVQPSDAGVYICTCR.N | Basement membrane-specific heparan sulfate proteoglycan core protein precursor | BCP & HP |
| R.IESSSPTVVEGQTLDLNCVVAR.Q | Basement membrane-specific heparan sulfate proteoglycan core protein precursor | BCP & HP |
| R.LLSGPYFWSLPSR.F | Basement membrane-specific heparan sulfate proteoglycan core protein precursor | BCP & HP |
| R.LPAVEPTDQAQYLCR.A | Basement membrane-specific heparan sulfate proteoglycan core protein precursor | BCP & HP |
| R.LPQVSPADSGEYVCR.V | Basement membrane-specific heparan sulfate proteoglycan core protein precursor | BCP & HP |
| R.LYQASPADSGEYVCR.V | Basement membrane-specific heparan sulfate proteoglycan core protein precursor | BCP & HP |
| R.RCESCAPGYEGNPIQPGGK.C | Basement membrane-specific heparan sulfate proteoglycan core protein precursor | BCP & HP |
| R.CSATGSPTPTLEWTGGPGGQLPAK.A | Basement membrane-specific heparan sulfate proteoglycan core protein precursor | BCP & HP |
| R.HLISTHFAPGDFQGFALVNPQR.N | Basement membrane-specific heparan sulfate proteoglycan core protein precursor | BCP & HP |
| K.FICPLTGLWPINTLK.C | Beta-2-glycoprotein 1 precursor | BCP & HP |
| K.CPFPSRPDNGFVNYPAKPTLYYK.D | Beta-2-glycoprotein 1 precursor | BCP & HP |
| K.CPFPSRPDNGFVNYPAKPTLYYKDK.A | Beta-2-glycoprotein 1 precursor | BCP & HP |
| K.CSYTEDAQCIDGTIEVPK.C | Beta-2-glycoprotein 1 precursor | BCP & HP |
| K.CTEEGKWSPELPVCAPII.C | Beta-2-glycoprotein 1 precursor | BCP & HP |
| K.CTEEGKWSPELPVCAPIICPPPSIPTFATLR.V | Beta-2-glycoprotein 1 precursor | BCP & HP |
| K.DKATFGCHDGYSLDGPEEIECTK.L | Beta-2-glycoprotein 1 precursor | BCP & HP |
| K.FICPLTGLWPINT.L | Beta-2-glycoprotein 1 precursor | BCP & HP |
| K.FKNGM*LHGDK.V | Beta-2-glycoprotein 1 precursor | BCP & HP |
| K.FKNGM*LHGDKVSFFCK.N | Beta-2-glycoprotein 1 precursor | BCP & HP |
| K.FKNGMLHGDK.V | Beta-2-glycoprotein 1 precursor | BCP & HP |
| K.FKNGMLHGDKVSFFCK.N | Beta-2-glycoprotein 1 precursor | BCP & HP |
| K.EHSSLAFWK.T | Beta-2-glycoprotein 1 precursor | BCP & HP |
| K.KATVVYQGER.V | Beta-2-glycoprotein 1 precursor | BCP & HP |
| K.ATFGCHDGYSLDGPEEIECTK.L | Beta-2-glycoprotein 1 precursor | BCP & HP |
| K.CPFPSRPDNGFVNYPAKPTLY.Y | Beta-2-glycoprotein 1 precursor | BCP & HP |
| K.ASCKVPVKK.A | Beta-2-glycoprotein 1 precursor | BCP & HP |
| K.ASCKVPVK.K | Beta-2-glycoprotein 1 precursor | BCP & HP |
| G.RTCPKPDDLPFSTVVPLK.T | Beta-2-glycoprotein 1 precursor | BCP & HP |
| F.YEPGEEITYSCKPGYVSR.G | Beta-2-glycoprotein 1 precursor | BCP & HP |
| F.SCNTGFYLNGADSAK.C | Beta-2-glycoprotein 1 precursor | BCP & HP |
| E.PGEEITYSCKPGYVSR.G | Beta-2-glycoprotein 1 precursor | BCP & HP |
| C.PPPSIPTFATLR.V | Beta-2-glycoprotein 1 precursor | BCP & HP |
| C.PLTGLWPINTLK.C | Beta-2-glycoprotein 1 precursor | BCP & HP |
| C.PFAGILENGAVR.Y | Beta-2-glycoprotein 1 precursor | BCP & HP |
| A.GRTCPKPDDLPFSTVVPLKTFYEPGEEITYSCKPGYVSR.G | Beta-2-glycoprotein 1 precursor | BCP & HP |
| A.GRTCPKPDDLPFSTVVPLK.T | Beta-2-glycoprotein 1 precursor | BCP & HP |
| K.CFKEHSSLAFWK.T | Beta-2-glycoprotein 1 precursor | BCP & HP |
| K.KCSYTEDAQCIDGTIEVPK.C | Beta-2-glycoprotein 1 precursor | BCP & HP |
| P.FPSRPDNGFVNYPAKPTLYYKDK.A | Beta-2-glycoprotein 1 precursor | BCP & HP |
| R.VCPFAGILENGAVR.Y | Beta-2-glycoprotein 1 precursor | BCP & HP |
| P.SRPDNGFVNYPAKPTLYYK.D | Beta-2-glycoprotein 1 precursor | BCP & HP |
| R.EVKCPFPSRPDNGFVNYPAKPTLYYK.D | Beta-2-glycoprotein 1 precursor | BCP & HP |
| R.EVKCPFPSRPDNGFVNYPAKPTLYYKDK.A | Beta-2-glycoprotein 1 precursor | BCP & HP |
| R.KFICPLTGLWPINTLK.C | Beta-2-glycoprotein 1 precursor | BCP & HP |
| R.TCPKPDDLPFSTVVPLK.T | Beta-2-glycoprotein 1 precursor | BCP & HP |
| R.TCPKPDDLPFSTVVPLKTFYEPGEEITYSCK.P | Beta-2-glycoprotein 1 precursor | BCP & HP |
| R.TCPKPDDLPFSTVVPLKTFYEPGEEITYSCKPGYVSR.G | Beta-2-glycoprotein 1 precursor | BCP & HP |
| K.ATVVYQGER.V | Beta-2-glycoprotein 1 precursor | BCP & HP |
| R.YTTFEYPNTISFSCNTGFYLNGADSAK.C | Beta-2-glycoprotein 1 precursor | BCP & HP |
| K.TFYEPGEEITYSCKPGYVSR.G | Beta-2-glycoprotein 1 precursor | BCP & HP |
| K.LGNWSAMPSCK.A | Beta-2-glycoprotein 1 precursor | BCP & HP |
| K.NGM*LHGDKVSFFCK.N | Beta-2-glycoprotein 1 precursor | BCP & HP |
| K.NGMLHGDK.V | Beta-2-glycoprotein 1 precursor | BCP & HP |
| K.NGMLHGDKVSFFCK.N | Beta-2-glycoprotein 1 precursor | BCP & HP |
| K.TFYEPGEEITYSCKPGYVS.R | Beta-2-glycoprotein 1 precursor | BCP & HP |
| R.VCPFAGILENGAVRY.T | Beta-2-glycoprotein 1 precursor | BCP & HP |
| K.VSFFCK.N | Beta-2-glycoprotein 1 precursor | BCP & HP |
| K.WSPELPVCAPIICPPPSIPTFATLR.V | Beta-2-glycoprotein 1 precursor | BCP & HP |
| P.FPSRPDNGFVNYPAKPTLYYK.D | Beta-2-glycoprotein 1 precursor | BCP & HP |
| K.TDASDVKPC.- | Beta-2-glycoprotein 1 precursor | BCP & HP |
| R.VASVDMGPQQLPDGQSLPIPPVILAELGSDPTK.G | Beta-Ala-His dipeptidase precursor | BCP & HP |
| R.M*M*AVAADTLQR.L | Beta-Ala-His dipeptidase precursor | BCP & HP |
| R.ALEQDLPVNIK.F | Beta-Ala-His dipeptidase precursor | BCP & HP |
| R.DGSTIPIAK.M | Beta-Ala-His dipeptidase precursor | BCP & HP |
| L.PDGQSLPIPPVILAELGSDPTK.G | Beta-Ala-His dipeptidase precursor | BCP & HP |
| R.GNSYFMVEVK.C | Beta-Ala-His dipeptidase precursor | BCP & HP |
| K.VFQYIDLHQDEFVQTLK.E | Beta-Ala-His dipeptidase precursor | BCP & HP |
| R.HLEDVFSKR.N | Beta-Ala-His dipeptidase precursor | BCP & HP |
| R.KPAITYGTR.G | Beta-Ala-His dipeptidase precursor | BCP & HP |
| R.MM*AVAADTLQR.L | Beta-Ala-His dipeptidase precursor | BCP & HP |
| R.MMAVAADTLQR.L | Beta-Ala-His dipeptidase precursor | BCP & HP |
| R.TVFGTEPDM*IR.D | Beta-Ala-His dipeptidase precursor | BCP & HP |
| R.TVFGTEPDM*IRDGSTIPIAK.M | Beta-Ala-His dipeptidase precursor | BCP & HP |
| R.TVFGTEPDMIR.D | Beta-Ala-His dipeptidase precursor | BCP & HP |
| R.VASVDM*GPQQLPDGQSLPIPPVILAELGSDPTK.G | Beta-Ala-His dipeptidase precursor | BCP & HP |
| R.WNYIEGTK.L | Beta-Ala-His dipeptidase precursor | BCP & HP |
| R.YPSLSIHGIEGAFDEPGTK.T | Beta-Ala-His dipeptidase precursor | BCP & HP |
| R.GATDNKGPVLAWINAVSAFR.A | Beta-Ala-His dipeptidase precursor | BCP & HP |
| K.SVVLIPLGAVDDGEHSQNEK.I | Beta-Ala-His dipeptidase precursor | BCP & HP |
| R.TVFGTEPDMIRDGSTIPIAK.M | Beta-Ala-His dipeptidase precursor | BCP & HP |
| K.MFQEIVHK.S | Beta-Ala-His dipeptidase precursor | BCP & HP |
| K.MVVSMTLGLHPWIANIDDTQYLAAK.R | Beta-Ala-His dipeptidase precursor | BCP & HP |
| K.M*FQEIVHK.S | Beta-Ala-His dipeptidase precursor | BCP & HP |
| K.FIIEGMEEAGSVALEELVEKEKDR.F | Beta-Ala-His dipeptidase precursor | BCP & HP |
| K.EWVAIESDSVQPVPR.F | Beta-Ala-His dipeptidase precursor | BCP & HP |
| K.AIHLDLEEYR.N | Beta-Ala-His dipeptidase precursor | BCP & HP |
| R.HLEDVFSK.R | Beta-Ala-His dipeptidase precursor | BCP & HP |
| R.MSQEPPQEMAK.A | Beta-type platelet-derived growth factor receptor precursor | BCP & HP |
| R.LVEPVTDFLLDMPYHIR.S | Beta-type platelet-derived growth factor receptor precursor | BCP & HP |
| K.KGDVALPVPYDHQR.G | Beta-type platelet-derived growth factor receptor precursor | BCP & HP |
| K.ALFVSEEEK.K | B-factor, properdin | BCP & HP |
| K.DNEQHVFK.V | B-factor, properdin | BCP & HP |
| K.LKYGQTIRPICLPCTEGTTR.A | B-factor, properdin | BCP & HP |
| K.DMENLEDVFYQMIDESQSLSLCGM*VWEHR.K | B-factor, properdin | BCP & HP |
| E.YVCPSGFYPYPVQTR.T | B-factor, properdin | BCP & HP |
| K.ALFVSEEEKK.L | B-factor, properdin | BCP & HP |
| A.RPQGSCSLEGVEIK.G | B-factor, properdin | BCP & HP |
| I.PEFYDYDVALIK.L | B-factor, properdin | BCP & HP |
| E.DSVTYHCSR.G | B-factor, properdin | BCP & HP |
| D.PNTCRGDSGGPLIVHKR.S | B-factor, properdin | BCP & HP |
| K.DMENLEDVFYQMIDESQSLSLCGMVWEHR.K | B-factor, properdin | BCP & HP |
| C.PSGFYPYPVQTR.T | B-factor, properdin | BCP & HP |
| K.DISEVVTPR.F | B-factor, properdin | BCP & HP |
| R.FLCTGGVSPYADPNTCRGDSGGP.L | B-factor, properdin | BCP & HP |
| R.HVIILMTDGLHNMGGDPITVIDEIR.D | B-factor, properdin | BCP & HP |
| R.DFHINLFQVLPWLK.E | B-factor, properdin | BCP & HP |
| R.DLEIEVVLFHPNYNINGK.K | B-factor, properdin | BCP & HP |
| R.DLEIEVVLFHPNYNINGKE.E | B-factor, properdin | BCP & HP |
| R.DLLYIGK.D | B-factor, properdin | BCP & HP |
| R.DLLYIGKDR.K | B-factor, properdin | BCP & HP |
| R.DAQYAPGYDKVK.D | B-factor, properdin | BCP & HP |
| R.FLCTGGVSPYADPNTCR.G | B-factor, properdin | BCP & HP |
| R.DAQYAPGYDK.V | B-factor, properdin | BCP & HP |
| R.FLCTGGVSPYADPNTCRGDSGGPLIVHK.R | B-factor, properdin | BCP & HP |
| R.FLCTGGVSPYADPNTCRGDSGGPLIVHKR.S | B-factor, properdin | BCP & HP |
| R.GDSGGPLIVHK.R | B-factor, properdin | BCP & HP |
| R.GDSGGPLIVHKR.S | B-factor, properdin | BCP & HP |
| R.HVIILM*TDGLHNMGGDPITVIDEIR.D | B-factor, properdin | BCP & HP |
| R.HVIILMTDGLHNM*GGDPITVIDEIR.D | B-factor, properdin | BCP & HP |
| R.EDYLDVYVFGVGPLVNQVNINALASKK.D | B-factor, properdin | BCP & HP |
| R.WSGQTAICDNGAGYCSNPGIPIGTR.K | B-factor, properdin | BCP & HP |
| K.KDNEQHVFK.V | B-factor, properdin | BCP & HP |
| K.EAGIPEFYDYDVALIK.L | B-factor, properdin | BCP & HP |
| W.SLARPQGSCSLEGVEIK.G | B-factor, properdin | BCP & HP |
| V.CPSGFYPYPVQTR.T | B-factor, properdin | BCP & HP |
| T.TPWSLARPQGSCSLEGVEIKGGSFR.L | B-factor, properdin | BCP & HP |
| T.TPWSLARPQGSCSLEGVEIK.G | B-factor, properdin | BCP & HP |
| R.DAQYAPGYDKVKDISEVVTPR.F | B-factor, properdin | BCP & HP |
| R.WSGQTAICDNGAGYCSNPGIPIGTRK.V | B-factor, properdin | BCP & HP |
| R.FIQVGVISWGVVDVCK.N | B-factor, properdin | BCP & HP |
| R.TCRSTGSWSTLK.T | B-factor, properdin | BCP & HP |
| R.STGSWSTLK.T | B-factor, properdin | BCP & HP |
| R.PQGSCSLEGVEIK.G | B-factor, properdin | BCP & HP |
| R.PHDFENGEYWPR.S | B-factor, properdin | BCP & HP |
| R.ALRLPPTTTCQQQK.E | B-factor, properdin | BCP & HP |
| R.ALRLPPTTTCQQQKEELLPAQDIK.A | B-factor, properdin | BCP & HP |
| R.YGLVTYATYPK.I | B-factor, properdin | BCP & HP |
| K.GSCERDAQYAPGYDKVKDISEVVTPR.F | B-factor, properdin | BCP & HP |
| K.QLNEINYEDHK.L | B-factor, properdin | BCP & HP |
| K.NPREDYLDVYVFGVGPLVNQVNINALASKK.D | B-factor, properdin | BCP & HP |
| K.NPREDYLDVYVFGVGPLVNQVNINALASK.K | B-factor, properdin | BCP & HP |
| K.LQDEDLGFL.- | B-factor, properdin | BCP & HP |
| R.LPPTTTCQQQKEELLPAQDIK.A | B-factor, properdin | BCP & HP |
| K.KGSCERDAQYAPGYDK.V | B-factor, properdin | BCP & HP |
| K.QLNEINYEDHKLK.S | B-factor, properdin | BCP & HP |
| R.KGTDYHKQPWQAK.I | B-factor, properdin | BCP & HP |
| K.GTDYHKQPWQAK.I | B-factor, properdin | BCP & HP |
| R.EDYLDVYVFGVGPLVNQVNINALASK.K | B-factor, properdin | BCP & HP |
| K.GSCERDAQYAPGYDK.V | B-factor, properdin | BCP & HP |
| K.GHESCMGAVVSEYFVLTAAHCFTVDDKEHSIK.V | B-factor, properdin | BCP & HP |
| K.EVYIKNGDKK.G | B-factor, properdin | BCP & HP |
| K.EKLQDEDLGFL.- | B-factor, properdin | BCP & HP |
| K.EELLPAQDIK.A | B-factor, properdin | BCP & HP |
| K.EEAGIPEFYDYDVALIK.L | B-factor, properdin | BCP & HP |
| K.KCLVNLIEK.V | B-factor, properdin | BCP & HP |
| R.LLQEGQALEYVCPSGFYPYPVQTR.T | B-factor, properdin | BCP & HP |
| R.KNPREDYLDVYVFGVGPLVNQVNINALASK.K | B-factor, properdin | BCP & HP |
| R.KNPREDYLDVYVFGVGPLVNQVNINALASKK.D | B-factor, properdin | BCP & HP |
| K.GSCERDAQYAPGYDKVK.D | B-factor, properdin | BCP & HP |
| K.RDLEIEVVLFHPNYNINGK.K | B-factor, properdin | BCP & HP |
| R.LEDSVTYHCSR.G | B-factor, properdin | BCP & HP |
| R.LPPTTTCQQQK.E | B-factor, properdin | BCP & HP |
| R.AIHCPRPHDFENGEYWPR.S | B-factor, properdin | BCP & HP |
| P.WSLARPQGSCSLEGVEIK.G | B-factor, properdin | BCP & HP |
| K.VSEADSSNADWVTK.Q | B-factor, properdin | BCP & HP |
| K.YGQTIRPICLPCTEGTTR.A | B-factor, properdin | BCP & HP |
| K.VASYGVKPR.Y | B-factor, properdin | BCP & HP |
| K.VSVGGEKR.D | B-factor, properdin | BCP & HP |
| K.VGSQYRLEDSVTYHCSR.G | B-factor, properdin | BCP & HP |
| K.VKDISEVVTPR.F | B-factor, properdin | BCP & HP |
| R.KVGSQYR.L | B-factor, properdin | BCP & HP |
| R.QEALELMNQNLDIYEQQVMTAAQK.D | biotinidase precursor | BCP & HP |
| R.LSCMAIR.G | biotinidase precursor | BCP & HP |
| R.GDMFLVANLGTK.E | biotinidase precursor | BCP & HP |
| R.LSSGLVTAALYGR.L | biotinidase precursor | BCP & HP |
| K.HNLYFEAAFDVPLK.V | biotinidase precursor | BCP & HP |
| K.SRLSSGLVTAALYGR.L | biotinidase precursor | BCP & HP |
| K.SHLIIAQVAK.N | biotinidase precursor | BCP & HP |
| K.ILSGDPYCEKDAQEVHCDEATK.W | biotinidase precursor | BCP & HP |
| R.TSIYPFLDFM*PSPQVVR.W | biotinidase precursor | BCP & HP |
| K.ILSGDPYCEK.D | biotinidase precursor | BCP & HP |
| R.TSIYPFLDFMPSPQVVR.W | biotinidase precursor | BCP & HP |
| K.HVVYPTAWMNQLPLLAAIEIQK.A | biotinidase precursor | BCP & HP |
| K.EGYLHVCSNGLCCYLLYERPTLSK.E | biotinidase precursor | BCP & HP |
| R.GGHCVALCTR.G | Bone marrow proteoglycan precursor | BCP & HP |
| K.ETPAATEAPSSTPK.A | Brain acid soluble protein 1 | BCP & HP |
| V.PGAPFLLQALVR.E | C4B1 | BCP & HP |
| R.YVSHFETEGPHVLLYFDSVPTSR.E | C4B1 | BCP & HP |
| T.PGKPYILTVPGHLDEMQLDIQAR.Y | C4B1 | BCP & HP |
| V.PGHLDEMQLDIQAR.Y | C4B1 | BCP & HP |
| V.PVGLVQPASATLYDYYNPER.R | C4B1 | BCP & HP |
| Y.ILTVPGHLDEMQLDIQAR.Y | C4B1 | BCP & HP |
| K.TEQWSTLPPETK.D | C4B1 | BCP & HP |
| R.YRVFALDQK.M | C4B1 | BCP & HP |
| K.M*RPSTDTITVMVENSHGLR.V | C4B1 | BCP & HP |
| K.LHLETDSLALVALGALDTALYAAGSK.S | C4B1 | BCP & HP |
| K.SRLLATLCSAEVCQCAEGK.C | C4B1 | BCP & HP |
| K.SHKPLNMGK.V | C4B1 | BCP & HP |
| K.SHALQLNNR.Q | C4B1 | BCP & HP |
| K.SCGLHQLLR.G | C4B1 | BCP & HP |
| K.RCCQDGVTR.L | C4B1 | BCP & HP |
| K.QRVEASISK.A | C4B1 | BCP & HP |
| K.PVQGVAYVR.F | C4B1 | BCP & HP |
| K.VDFTLSSER.D | C4B1 | BCP & HP |
| K.MRPSTDTITVM*VENSHGLR.V | C4B1 | BCP & HP |
| K.VDFTLSSERDFALLSLQVPLK.D | C4B1 | BCP & HP |
| K.M*RPSTDTITVM*VENSHGLR.V | C4B1 | BCP & HP |
| K.LVNGQSHISLSKAEFQDALEKLNMGITDLQGLR.L | C4B1 | BCP & HP |
| K.LVNGQSHISLSKAEFQDALEKLNM*GITDLQGLR.L | C4B1 | BCP & HP |
| K.LVNGQSHISLSKAEFQDALEK.L | C4B1 | BCP & HP |
| K.LVNGQSHISLSK.A | C4B1 | BCP & HP |
| K.LTSLSDRYVSHFETEGPHVLLYFDSVPTSR.E | C4B1 | BCP & HP |
| K.LQETSNWLLSQQQADGSFQDLSPVIHR.S | C4B1 | BCP & HP |
| K.LNMGITDLQGLR.L | C4B1 | BCP & HP |
| K.TKGLCVATPVQLR.V | C4B1 | BCP & HP |
| K.MRPSTDTITVMVENSHGLR.V | C4B1 | BCP & HP |
| K.VLSLAQEQVGGSPEKLQETSNWLLSQQQADGSFQDLSPVIHR.S | C4B1 | BCP & HP |
| Q.PASATLYDYYNPER.R | C4B1 | BCP & HP |
| P.EVQLVAHSPWLK.D | C4B1 | BCP & HP |
| M.PSSIFQDDFVIPDISEPGTWK.I | C4B1 | BCP & HP |
| L.SLAQEQVGGSPEK.L | C4B1 | BCP & HP |
| L.PRGCGEQTMIYLAPTLAASR.Y | C4B1 | BCP & HP |
| L.LSQQQADGSFQDLSPVIHR.S | C4B1 | BCP & HP |
| L.CSAEVCQCAEGK.C | C4B1 | BCP & HP |
| L.AQEQVGGSPEK.L | C4B1 | BCP & HP |
| K.TEQWSTLPPETKDHAVDLIQK.G | C4B1 | BCP & HP |
| K.VVEEQESR.V | C4B1 | BCP & HP |
| K.LGQYASPTAKR.C | C4B1 | BCP & HP |
| K.VLSLAQEQVGGSPEK.L | C4B1 | BCP & HP |
| K.VLQIEKEGAIHREELVYELNPLDHR.G | C4B1 | BCP & HP |
| K.VLQIEKEGAIHR.E | C4B1 | BCP & HP |
| K.VGLSGMAIADVTLLSGFHALRADLEK.L | C4B1 | BCP & HP |
| K.VGLSGMAIADVTLLSGFHALR.A | C4B1 | BCP & HP |
| K.VGLSGM*AIADVTLLSGFHALR.A | C4B1 | BCP & HP |
| K.VFEAMNSYDLGCGPGGGDSALQVFQAAGLAFSDGDQWTLSR.K | C4B1 | BCP & HP |
| K.VFEAM*NSYDLGCGPGGGDSALQVFQAAGLAFSDGDQWTLSR.K | C4B1 | BCP & HP |
| K.VDFTLSSERDFALLSLQVPLKDAK.S | C4B1 | BCP & HP |
| K.YVLPNFEVK.I | C4B1 | BCP & HP |
| I.PDGDFNSYVR.V | C4B1 | BCP & HP |
| K.LNM*GITDLQGLR.L | C4B1 | BCP & HP |
| K.AEM*ADQAAAWLTR.Q | C4B1 | BCP & HP |
| K.AEFQDALEKLNMGITDLQGLR.L | C4B1 | BCP & HP |
| K.AEFQDALEKLNM*GITDLQGLR.L | C4B1 | BCP & HP |
| K.AEFQDALEK.L | C4B1 | BCP & HP |
| K.ADGSYAAWLSR.D | C4B1 | BCP & HP |
| I.PQTISELQLSVSAGSPHPAIAR.L | C4B1 | BCP & HP |
| I.PIIIPQTISELQLSVSAGSPHPAIAR.L | C4B1 | BCP & HP |
| K.ASAGLLGAHAAAITAYALTLTK.A | C4B1 | BCP & HP |
| I.PDISEPGTWK.I | C4B1 | BCP & HP |
| K.ASAGLLGAHAAAITAYALTLTKAPADLR.G | C4B1 | BCP & HP |
| I.ALDALSAYWIASHTTEER.G | C4B1 | BCP & HP |
| E.VPVGLVQPASATLYDYYNPER.R | C4B1 | BCP & HP |
| E.QTMIYLAPTLAASR.Y | C4B1 | BCP & HP |
| E.FQDALEKLNMGITDLQGLR.L | C4B1 | BCP & HP |
| D.SLALVALGALDTALYAAGSK.S | C4B1 | BCP & HP |
| D.PLDTLGSEGALSPGGVASLLR.L | C4B1 | BCP & HP |
| D.CREPFLSCCQFAESLR.K | C4B1 | BCP & HP |
| A.SATLYDYYNPER.R | C4B1 | BCP & HP |
| A.LDALSAYWIASHTTEER.G | C4B1 | BCP & HP |
| I.PGNSDPNM*IPDGDFNSYVR.V | C4B1 | BCP & HP |
| K.GLCVATPVQLR.V | C4B1 | BCP & HP |
| K.LGQYASPTAK.R | C4B1 | BCP & HP |
| K.LELSVDGAK.Q | C4B1 | BCP & HP |
| K.KYVLPNFEVK.I | C4B1 | BCP & HP |
| K.KEVYMPSSIFQDDFVIPDISEPGTWK.I | C4B1 | BCP & HP |
| K.KEVYM*PSSIFQDDFVIPDISEPGTWK.I | C4B1 | BCP & HP |
| K.ITQVLHFTKDVK.A | C4B1 | BCP & HP |
| K.ITQVLHFTK.D | C4B1 | BCP & HP |
| K.ITPGKPYILTVPGHLDEMQLDIQAR.Y | C4B1 | BCP & HP |
| K.AEMADQAAAWLTR.Q | C4B1 | BCP & HP |
| K.GSVFLRNPSR.N | C4B1 | BCP & HP |
| R.ADLEKLTSLSDRYVSHFETEGPHVLLYFDSVPTSR.E | C4B1 | BCP & HP |
| K.FACYYPR.V | C4B1 | BCP & HP |
| K.EVYMPSSIFQDDFVIPDISEPGTWK.I | C4B1 | BCP & HP |
| K.EVYM*PSSIFQDDFVIPDISEPGTWK.I | C4B1 | BCP & HP |
| K.EGAIHREELVYELNPLDHRG.R | C4B1 | BCP & HP |
| K.EGAIHREELVYELNPLDHR.G | C4B1 | BCP & HP |
| K.DHAVDLIQKGYMR.I | C4B1 | BCP & HP |
| K.DHAVDLIQK.G | C4B1 | BCP & HP |
| K.DDPDAPLQPVTPLQLFEGR.R | C4B1 | BCP & HP |
| K.ASSFLGEK.A | C4B1 | BCP & HP |
| K.ITPGKPYILTVPGHLDEM*QLDIQAR.Y | C4B1 | BCP & HP |
| R.MKFACYYPR.V | C4B1 | BCP & HP |
| R.AACAQLNDFLQEYGTQGCQV.- | C4B1 | BCP & HP |
| R.QRAACAQLNDFLQEYGTQGCQV.- | C4B1 | BCP & HP |
| R.QIRGLEEELQFSLGSK.I | C4B1 | BCP & HP |
| R.QGSFQGGFRSTQDTVIALDALSAYWIASHTTEER.G | C4B1 | BCP & HP |
| R.QGSFQGGFR.S | C4B1 | BCP & HP |
| R.PVAFSVVPTAATAVSLK.V | C4B1 | BCP & HP |
| R.NPSRNNVPCSPK.V | C4B1 | BCP & HP |
| R.NGKVGLSGMAIADVTLLSGFHALR.A | C4B1 | BCP & HP |
| R.RFEQLELRPVLYNYLDK.N | C4B1 | BCP & HP |
| R.NGESVKLHLETDSLALVALGALDTALYAAGSK.S | C4B1 | BCP & HP |
| R.RGHLFLQTDQPIYNPGQR.V | C4B1 | BCP & HP |
| R.M*KFACYYPR.V | C4B1 | BCP & HP |
| R.LTVAAPPSGGPGFLSIERPDSRPPR.V | C4B1 | BCP & HP |
| R.LRLEPGKEYLIMGLDGATYDLEGHPQYLLDSNSWIEEMPSER.L | C4B1 | BCP & HP |
| R.LRLEPGKEYLIM*GLDGATYDLEGHPQYLLDSNSWIEEMPSER.L | C4B1 | BCP & HP |
| R.LLLFSPSVVHLGVPLSVGVQLQDVPR.G | C4B1 | BCP & HP |
| R.LLATLCSAEVCQCAEGKCPR.Q | C4B1 | BCP & HP |
| R.LLATLCSAEVCQCAEGK.C | C4B1 | BCP & HP |
| R.LEPGKEYLIMGLDGATYDLEGHPQYLLDSNSWIEEMPSER.L | C4B1 | BCP & HP |
| R.KKEVYMPSSIFQDDFVIPDISEPGTWK.I | C4B1 | BCP & HP |
| R.NGFKSHALQLNNR.Q | C4B1 | BCP & HP |
| R.VDVQAGACEGK.L | C4B1 | BCP & HP |
| R.YLDKTEQWSTLPPETK.D | C4B1 | BCP & HP |
| R.YIYGKPVQGVAYVR.F | C4B1 | BCP & HP |
| R.VTASDPLDTLGSEGALSPGGVASLLR.L | C4B1 | BCP & HP |
| R.VQQPDCREPFLSCCQFAESLRK.K | C4B1 | BCP & HP |
| R.VQQPDCREPFLSCCQFAESLR.K | C4B1 | BCP & HP |
| R.VHYTVCIWR.N | C4B1 | BCP & HP |
| R.VGDTLNLNLR.A | C4B1 | BCP & HP |
| R.VFALDQK.M | C4B1 | BCP & HP |
| R.RCSVFYGAPSK.S | C4B1 | BCP & HP |
| R.VDVQAGACEGKLELSVDGAK.Q | C4B1 | BCP & HP |
| R.HLVPGAPFLLQALVR.E | C4B1 | BCP & HP |
| R.TYNVLDMK.N | C4B1 | BCP & HP |
| R.TYNVLDM*K.N | C4B1 | BCP & HP |
| R.TTNIQGINLLFSSR.R | C4B1 | BCP & HP |
| R.TLEIPGNSDPNMIPDGDFNSYVR.V | C4B1 | BCP & HP |
| R.TLEIPGNSDPNM*IPDGDFNSYVR.V | C4B1 | BCP & HP |
| R.STQDTVIALDALSAYWIASHTTEER.G | C4B1 | BCP & HP |
| R.SMQGGLVGNDETVALTAFVTIALHHGLAVFQDEGAEPLK.Q | C4B1 | BCP & HP |
| R.SM*QGGLVGNDETVALTAFVTIALHHGLAVFQDEGAEPLK.Q | C4B1 | BCP & HP |
| R.SFFPENWLWR.V | C4B1 | BCP & HP |
| R.VEYGFQVK.V | C4B1 | BCP & HP |
| R.DFALLSLQVPLK.D | C4B1 | BCP & HP |
| R.EPFLSCCQFAESLRK.K | C4B1 | BCP & HP |
| R.EPFLSCCQFAESLR.K | C4B1 | BCP & HP |
| R.EMSGSPASGIPVK.V | C4B1 | BCP & HP |
| R.EM*SGSPASGIPVK.V | C4B1 | BCP & HP |
| R.EELVYELNPLDHR.G | C4B1 | BCP & HP |
| R.ECVGFEAVQEVPVGLVQPASATLYDYYNPERR.C | C4B1 | BCP & HP |
| R.ECVGFEAVQEVPVGLVQPASATLYDYYNPER.R | C4B1 | BCP & HP |
| R.EAPKVVEEQESR.V | C4B1 | BCP & HP |
| R.KKEVYM*PSSIFQDDFVIPDISEPGTWK.I | C4B1 | BCP & HP |
| R.DFALLSLQVPLKDAK.S | C4B1 | BCP & HP |
| R.FGLLDEDGKKTFFR.G | C4B1 | BCP & HP |
| R.CSVFYGAPSKSR.L | C4B1 | BCP & HP |
| R.CSVFYGAPSK.S | C4B1 | BCP & HP |
| R.CCQDGVTRLPMMR.S | C4B1 | BCP & HP |
| R.CCQDGVTRLPM*M*R.S | C4B1 | BCP & HP |
| R.CCQDGVTR.L | C4B1 | BCP & HP |
| R.AVGSGATFSHYYYMILSR.G | C4B1 | BCP & HP |
| R.AVGSGATFSHYYYM*ILSR.G | C4B1 | BCP & HP |
| R.ALEILQEEDLIDEDDIPVR.S | C4B1 | BCP & HP |
| R.YLDKTEQWSTLPPETKDHAVDLIQK.G | C4B1 | BCP & HP |
| R.DKGQAGLQR.A | C4B1 | BCP & HP |
| R.GPEVQLVAHSPWLK.D | C4B1 | BCP & HP |
| R.ADLEKLTSLSDR.Y | C4B1 | BCP & HP |
| R.GSSTWLTAFVLK.V | C4B1 | BCP & HP |
| R.GSFEFPVGDAVSK.V | C4B1 | BCP & HP |
| R.GRTLEIPGNSDPNMIPDGDFNSYVR.V | C4B1 | BCP & HP |
| R.GRTLEIPGNSDPNM*IPDGDFNSYVR.V | C4B1 | BCP & HP |
| R.GQIVFMNREPKR.T | C4B1 | BCP & HP |
| R.GQIVFMNREPK.R | C4B1 | BCP & HP |
| R.GQIVFMNR.E | C4B1 | BCP & HP |
| R.GQIVFM*NREPK.R | C4B1 | BCP & HP |
| R.FGLLDEDGK.K | C4B1 | BCP & HP |
| R.GPEVQLVAHSPWLKDSLSR.T | C4B1 | BCP & HP |
| R.FGLLDEDGKK.T | C4B1 | BCP & HP |
| R.GLQDEDGYR.M | C4B1 | BCP & HP |
| R.GLESQTK.L | C4B1 | BCP & HP |
| R.GLEEELQFSLGSK.I | C4B1 | BCP & HP |
| R.GHLFLQTDQPIYNPGQR.V | C4B1 | BCP & HP |
| R.GCGEQTMIYLAPTLAASR.Y | C4B1 | BCP & HP |
| R.GCGEQTM*IYLAPTLAASRYLDKTEQWSTLPPETK.D | C4B1 | BCP & HP |
| R.GCGEQTM*IYLAPTLAASR.Y | C4B1 | BCP & HP |
| R.FGLLDEDGKKTFFRGLESQTK.L | C4B1 | BCP & HP |
| R.KADGSYAAWLSR.D | C4B1 | BCP & HP |
| R.GQIVFM*NR.E | C4B1 | BCP & HP |
| L.GNCGPPPTLSFAAPMDITLTETR.F | C4b-binding protein alpha chain precursor | BCP & HP |
| P.DVSHGEMVSGFGPIYNYKDTIVFK.C | C4b-binding protein alpha chain precursor | BCP & HP |
| R.FKTGTTLK.Y | C4b-binding protein alpha chain precursor | BCP & HP |
| R.CHPGYKPTTDEPTTVICQK.N | C4b-binding protein alpha chain precursor | BCP & HP |
| P.DVSHGEMVSGFGPIYNYK.D | C4b-binding protein alpha chain precursor | BCP & HP |
| K.YTCLPGYVR.S | C4b-binding protein alpha chain precursor | BCP & HP |
| K.WNPSPPACEPNSCINLPDIPHASWETYPRPTKEDVYVVGTVLR.Y | C4b-binding protein alpha chain precursor | BCP & HP |
| K.RLMQCLPNPEDVK.M | C4b-binding protein alpha chain precursor | BCP & HP |
| K.QSSSYSFFKEEIIYECDKGYILVGQAK.L | C4b-binding protein alpha chain precursor | BCP & HP |
| R.FSAICQGDGTWSPR.T | C4b-binding protein alpha chain precursor | BCP & HP |
| K.LSCSYSHWSAPAPQCK.A | C4b-binding protein alpha chain precursor | BCP & HP |
| K.LSLEIEQLELQR.D | C4b-binding protein alpha chain precursor | BCP & HP |
| K.LNNGEITQHR.K | C4b-binding protein alpha chain precursor | BCP & HP |
| K.EEIIYECDKGYILVGQAK.L | C4b-binding protein alpha chain precursor | BCP & HP |
| K.EDVYVVGTVLR.Y | C4b-binding protein alpha chain precursor | BCP & HP |
| K.CKPPPDIR.N | C4b-binding protein alpha chain precursor | BCP & HP |
| K.CEWETPEGCEQVLTGKR.L | C4b-binding protein alpha chain precursor | BCP & HP |
| G.NCGPPPTLSFAAPMDITLTETR.F | C4b-binding protein alpha chain precursor | BCP & HP |
| G.NCGPPPTLSFAAPM*DITLTETR.F | C4b-binding protein alpha chain precursor | BCP & HP |
| K.MALEVYK.L | C4b-binding protein alpha chain precursor | BCP & HP |
| R.SHSTQTLTCNSDGEWVYNTFCIYK.R | C4b-binding protein alpha chain precursor | BCP & HP |
| R.TWYPEVPKCEWETPEGCEQVLTGKR.L | C4b-binding protein alpha chain precursor | BCP & HP |
| R.TWYPEVPKCEWETPEGCEQVLTGK.R | C4b-binding protein alpha chain precursor | BCP & HP |
| K.LNNGEITQHRK.S | C4b-binding protein alpha chain precursor | BCP & HP |
| R.WTPYQGCEALCCPEPK.L | C4b-binding protein alpha chain precursor | BCP & HP |
| R.GSSVIHCDADSK.W | C4b-binding protein alpha chain precursor | BCP & HP |
| R.SHSTQTLTCNSDGEWVYNTFCIYKR.C | C4b-binding protein alpha chain precursor | BCP & HP |
| R.TWYPEVPK.C | C4b-binding protein alpha chain precursor | BCP & HP |
| R.LMQCLPNPEDVK.M | C4b-binding protein alpha chain precursor | BCP & HP |
| R.LM*QCLPNPEDVK.M | C4b-binding protein alpha chain precursor | BCP & HP |
| R.KPDVSHGEMVSGFGPIYNYKDTIVFK.C | C4b-binding protein alpha chain precursor | BCP & HP |
| R.KPDVSHGEMVSGFGPIYNYK.D | C4b-binding protein alpha chain precursor | BCP & HP |
| R.KPDVSHGEM*VSGFGPIYNYKDTIVFK.C | C4b-binding protein alpha chain precursor | BCP & HP |
| R.GVGWSHPLPQCEIVK.C | C4b-binding protein alpha chain precursor | BCP & HP |
| R.KPELVNGR.L | C4b-binding protein alpha chain precursor | BCP & HP |
| R.HSGEENFYAYGFSVTYSCDPR.F | C4b-binding protein alpha chain precursor | BCP & HP |
| R.TPSCGDICNFPPK.I | C4b-binding protein alpha chain precursor | BCP & HP |
| R.KPDVSHGEM*VSGFGPIYNYK.D | C4b-binding protein alpha chain precursor | BCP & HP |
| K.QSNNKYAASSYLSLTPEQWK.S | C7 protein | BCP & HP |
| A.ASSYLSLTPEQWK.S | C7 protein | BCP & HP |
| K.VGVETTKPSK.Q | C7 protein | BCP & HP |
| K.YAASSYLSLTPEQWK.S | C7 protein | BCP & HP |
| F.PPSSEELQANK.A | C7 protein | BCP & HP |
| K.AAPSVTLFPPSSEELQANK.A | C7 protein | BCP & HP |
| K.ADGSPVKVGVETTKPSK.Q | C7 protein | BCP & HP |
| R.SYSCRVTHEGSTVEK.T | C7 protein | BCP & HP |
| R.SIVVSPILIPENQR.Q | Cadherin-13 precursor | BCP & HP |
| R.INENTGSVSVTR.T | Cadherin-13 precursor | BCP & HP |
| K.TLEGPVPLEVIVIDQNDNRPIFR.E | Cadherin-13 precursor | BCP & HP |
| R.TLDREVIAVYQLFVETTDVNGK.T | Cadherin-13 precursor | BCP & HP |
| K.YLLKGEYVGK.V | Cadherin-5 precursor | BCP & HP |
| K.EYFAIDNSGR.I | Cadherin-5 precursor | BCP & HP |
| K.ELDSTGTPTGK.E | Cadherin-5 precursor | BCP & HP |
| K.GKEYFAIDNSGR.I | Cadherin-5 precursor | BCP & HP |
| K.KPLIGTVLAMDPDAAR.H | Cadherin-5 precursor | BCP & HP |
| K.VHDVNDNWPVFTHR.L | Cadherin-5 precursor | BCP & HP |
| K.YGQFDREHTK.V | Cadherin-5 precursor | BCP & HP |
| R.TSDKGQFFR.V | Cadherin-5 precursor | BCP & HP |
| R.YM*SPPAGNR.A | Cadherin-5 precursor | BCP & HP |
| K.VHFLPVVISDNGMPSR.T | Cadherin-5 precursor | BCP & HP |
| K.YTFVVPEDTR.V | Cadherin-5 precursor | BCP & HP |
| K.EQFLDGDGWTSR.W | Calreticulin precursor | BCP & HP |
| K.FYGDEEKDKGLQTSQDAR.F | Calreticulin precursor | BCP & HP |
| L.TADSDVDEFLDKFLSAGVK.Q | Carbohydrate sulfotransferase 12 | BCP & HP |
| K.LYPIANGNNQSPVDIK.T | Carbonic anhydrase 1 | BCP & HP |
| R.SLLSNVEGDNAVPM*QHNNRPTQPLK.G | Carbonic anhydrase 1 | BCP & HP |
| K.YSSLAEAASKADGLAVIGVLMK.V | Carbonic anhydrase 1 | BCP & HP |
| K.YSSLAEAASK.A | Carbonic anhydrase 1 | BCP & HP |
| K.ESISVSSEQLAQFR.S | Carbonic anhydrase 1 | BCP & HP |
| K.TSETKHDTSLKPISVSYNPATAK.E | Carbonic anhydrase 1 | BCP & HP |
| K.ADGLAVIGVLM*K.V | Carbonic anhydrase 1 | BCP & HP |
| K.ADGLAVIGVLMK.V | Carbonic anhydrase 1 | BCP & HP |
| R.SLLSNVEGDNAVPMQHNNRPTQPLK.G | Carbonic anhydrase 1 | BCP & HP |
| K.HDTSLKPISVSYNPATAK.E | Carbonic anhydrase 1 | BCP & HP |
| K.VLDALQAIK.T | Carbonic anhydrase 1 | BCP & HP |
| K.EIINVGHSFHVNFEDNDNR.S | Carbonic anhydrase 1 | BCP & HP |
| K.AVQQPDGLAVLGIFLK.V | Carbonic anhydrase 2 | BCP & HP |
| K.VGSAKPGLQK.V | Carbonic anhydrase 2 | BCP & HP |
| K.SADFTNFDPR.G | Carbonic anhydrase 2 | BCP & HP |
| R.KLNFNGEGEPEELMVDNWRPAQPLK.N | Carbonic anhydrase 2 | BCP & HP |
| K.YAAELHLVHWNTK.Y | Carbonic anhydrase 2 | BCP & HP |
| K.VVDVLDSIK.T | Carbonic anhydrase 2 | BCP & HP |
| R.LLLPGIYTVSATAPGYDPETVTVTVGPAEPTLVNFHLKR.S | Carboxypeptidase N catalytic chain precursor | BCP & HP |
| K.RSIPQVSPVR.R | Carboxypeptidase N catalytic chain precursor | BCP & HP |
| R.EWLGNREALIQFLEQVHQGIK.G | Carboxypeptidase N catalytic chain precursor | BCP & HP |
| R.IVQLIQDTR.I | Carboxypeptidase N catalytic chain precursor | BCP & HP |
| R.NFPDLNTYIYYNEK.Y | Carboxypeptidase N catalytic chain precursor | BCP & HP |
| R.IHILPSMNPDGYEVAAAQGPNKPGYLVGR.N | Carboxypeptidase N catalytic chain precursor | BCP & HP |
| R.IHILPSM*NPDGYEVAAAQGPNKPGYLVGR.N | Carboxypeptidase N catalytic chain precursor | BCP & HP |
| R.ELM*LQLSEFLCEEFR.N | Carboxypeptidase N catalytic chain precursor | BCP & HP |
| R.EALIQFLEQVHQGIK.G | Carboxypeptidase N catalytic chain precursor | BCP & HP |
| K.YVGNMHGNEALGR.E | Carboxypeptidase N catalytic chain precursor | BCP & HP |
| K.VQNECPGITR.V | Carboxypeptidase N catalytic chain precursor | BCP & HP |
| K.GMVLDENYNNLANAVISVSGINHDVTSGDHGDYFR.L | Carboxypeptidase N catalytic chain precursor | BCP & HP |
| R.HLYVLEFSDHPGIHEPLEPEVK.Y | Carboxypeptidase N catalytic chain precursor | BCP & HP |
| R.NNANGVDLNR.N | Carboxypeptidase N catalytic chain precursor | BCP & HP |
| K.YVGNM*HGNEALGR.E | Carboxypeptidase N catalytic chain precursor | BCP & HP |
| R.RTASTPTPDDKLFQK.L | Carboxypeptidase N catalytic chain precursor | BCP & HP |
| R.TASTPTPDDKLFQK.L | Carboxypeptidase N catalytic chain precursor | BCP & HP |
| R.TLYKVQNECPGITR.V | Carboxypeptidase N catalytic chain precursor | BCP & HP |
| R.YDDLVR.T | Carboxypeptidase N catalytic chain precursor | BCP & HP |
| R.ELMLQLSEFLCEEFR.N | Carboxypeptidase N catalytic chain precursor | BCP & HP |
| R.AYRSERFLPSEQIQGVVISVINLEPRTGFLSNPR.A | Cartilage intermediate layer protein 1 precursor | BCP & HP |
| R.DAVQNCCGISK.T | Cartilage intermediate layer protein 1 precursor | BCP & HP |
| R.TTDWTPAGSTGQVVHGSPR.E | Cartilage intermediate layer protein 1 precursor | BCP & HP |
| R.SKDVAVIAESIR.M | cat eye syndrome critical region protein 1 isoform b | BCP & HP |
| K.DIPIEVCPISNQVLK.L | cat eye syndrome critical region protein 1 isoform b | BCP & HP |
| R.RFNTANDDNVTQVR.A | Catalase | BCP & HP |
| R.LFAYPDTHR.H | Catalase | BCP & HP |
| R.FSTVAGESGSADTVRDPR.G | Catalase | BCP & HP |
| R.FNTANDDNVTQVR.A | Catalase | BCP & HP |
| R.AFYVNVLNEEQR.K | Catalase | BCP & HP |
| R.AAQKADVLTTGAGNPVGDKLNVITVGPR.G | Catalase | BCP & HP |
| K.LVNANGEAVYCK.F | Catalase | BCP & HP |
| K.NFTEVHPDYGSHIQALLDKYNAEKPK.N | Catalase | BCP & HP |
| K.ADVLTTGAGNPVGDKLNVITVGPR.G | Catalase | BCP & HP |
| K.AIGAVPLIQGEYMIPCEK.V | Cathepsin D precursor | BCP & HP |
| K.FDGILGM*AYPR.I | Cathepsin D precursor | BCP & HP |
| K.FDGILGMAYPR.I | Cathepsin D precursor | BCP & HP |
| R.DPDAQPGGELM*LGGTDSK.Y | Cathepsin D precursor | BCP & HP |
| R.DPDAQPGGELMLGGTDSK.Y | Cathepsin D precursor | BCP & HP |
| R.TMSEVGGSVEDLIAK.G | Cathepsin D precursor | BCP & HP |
| R.ISVNNVLPVFDNLMQQK.L | Cathepsin D precursor | BCP & HP |
| K.MLSLAEQQLVDCAQDFNNHGCQGGLPSQAFEYILYNK.G | Cathepsin H precursor | BCP & HP |
| K.GIMGEDTYPYQGK.D | Cathepsin H precursor | BCP & HP |
| K.GIM*GEDTYPYQGK.D | Cathepsin H precursor | BCP & HP |
| K.YQGSCGACWAFSAVGALEAQLK.L | Cathepsin S precursor | BCP & HP |
| K.GIDSDASYPYK.A | Cathepsin S precursor | BCP & HP |
| R.IVTSTYKDGK.G | Cathepsin Z precursor | BCP & HP |
| R.STYPRPHEYLSPADLPK.S | Cathepsin Z precursor | BCP & HP |
| R.VGDYGSLSGR.E | Cathepsin Z precursor | BCP & HP |
| K.LSGAYLVDDSDPDTSLFINVCR.D | Cation-independent mannose-6-phosphate receptor precursor | BCP & HP |
| K.KYDFYINVCGPVSVSPCQPDSGACQVAK.S | Cation-independent mannose-6-phosphate receptor precursor | BCP & HP |
| R.WYTSYACPEEPLECVVTDPSTLEQYDLSSLAK.S | Cation-independent mannose-6-phosphate receptor precursor | BCP & HP |
| R.KPWTAVDTSVDGR.K | Cation-independent mannose-6-phosphate receptor precursor | BCP & HP |
| R.HAEPEQNWEAVDGSQTETEKK.H | Cation-independent mannose-6-phosphate receptor precursor | BCP & HP |
| R.GCPEDAAVCAVDK.N | Cation-independent mannose-6-phosphate receptor precursor | BCP & HP |
| K.LSSDVCPTSDK.S | Cation-independent mannose-6-phosphate receptor precursor | BCP & HP |
| R.YSDNWEAITGTGDPEHYLINVCK.S | Cation-independent mannose-6-phosphate receptor precursor | BCP & HP |
| R.FVCNDDVYSGPLK.F | Cation-independent mannose-6-phosphate receptor precursor | BCP & HP |
| R.KPIWLSQMSCSGR.E | CD5 antigen-like precursor | BCP & HP |
| K.GVWGSVCDDNWGEKEDQVVCK.Q | CD5 antigen-like precursor | BCP & HP |
| K.HQNQWYTVCQTGWSLR.A | CD5 antigen-like precursor | BCP & HP |
| R.ELGCGAASGTPSGILYEPPAEKEQK.V | CD5 antigen-like precursor | BCP & HP |
| K.NTCNHDEDTWVECEDPFDLR.L | CD5 antigen-like precursor | BCP & HP |
| R.KPIWLSQM*SCSGR.E | CD5 antigen-like precursor | BCP & HP |
| R.EATLQDCPSGPWGK.N | CD5 antigen-like precursor | BCP & HP |
| R.LVGGDNLCSGR.L | CD5 antigen-like precursor | BCP & HP |
| K.CYGPGVGR.I | CD5 antigen-like precursor | BCP & HP |
| K.GQWGTVCDDGWDIKDVAVLCR.E | CD5 antigen-like precursor | BCP & HP |
| R.CSGEEQSLEQCQHR.F | CD5 antigen-like precursor | BCP & HP |
| R.ELGCGAASGTPSGILYEPPAEK.E | CD5 antigen-like precursor | BCP & HP |
| K.AGLQVYNK.C | CD59 glycoprotein precursor | BCP & HP |
| R.LRENELTYYCCKK.D | CD59 glycoprotein precursor | BCP & HP |
| S.LQCYNCPNPTADCK.T | CD59 glycoprotein precursor | BCP & HP |
| K.LGNFPWQAFTSIHGR.G | CDNA FLJ14022 fis, clone HEMBA1003538, weakly similar to COMPLEMENT C1R COMPONENT | BCP & HP |
| K.VQNHCQEPYYQAAAAGALTCATPGTWK.D | CDNA FLJ14022 fis, clone HEMBA1003538, weakly similar to COMPLEMENT C1R COMPONENT | BCP & HP |
| R.GGGALLGDR.W | CDNA FLJ14022 fis, clone HEMBA1003538, weakly similar to COMPLEMENT C1R COMPONENT | BCP & HP |
| R.GGGALLGDRWILTAAHTVYPK.D | CDNA FLJ14022 fis, clone HEMBA1003538, weakly similar to COMPLEMENT C1R COMPONENT | BCP & HP |
| R.GSEAINAPGDNPAK.V | CDNA FLJ14022 fis, clone HEMBA1003538, weakly similar to COMPLEMENT C1R COMPONENT | BCP & HP |
| R.VVVHPDYR.Q | CDNA FLJ14022 fis, clone HEMBA1003538, weakly similar to COMPLEMENT C1R COMPONENT | BCP & HP |
| R.WILTAAHTVYPK.D | CDNA FLJ14022 fis, clone HEMBA1003538, weakly similar to COMPLEMENT C1R COMPONENT | BCP & HP |
| K.YSRLPVAPR.E | CDNA FLJ14022 fis, clone HEMBA1003538, weakly similar to COMPLEMENT C1R COMPONENT | BCP & HP |
| G.SVLLAQELPQQLTSPGYPEPYGK.G | CDNA FLJ14022 fis, clone HEMBA1003538, weakly similar to COMPLEMENT C1R COMPONENT | BCP & HP |
| G.SVLLAQELPQQLTSPGYPEPYGKGQESSTDIKAPEGFAVR.L | CDNA FLJ14022 fis, clone HEMBA1003538, weakly similar to COMPLEMENT C1R COMPONENT | BCP & HP |
| K.GQESSTDIKAPEGFAVR.L | CDNA FLJ14022 fis, clone HEMBA1003538, weakly similar to COMPLEMENT C1R COMPONENT | BCP & HP |
| R.EACNAWLQK.R | CDNA FLJ14022 fis, clone HEMBA1003538, weakly similar to COMPLEMENT C1R COMPONENT | BCP & HP |
| K.YLTWASR.Q | CDNA FLJ14473 fis, clone MAMMA1001080, highly similar to Homo sapiens SNC73 protein (SNC73) mRNA | BCP & HP |
| K.GDTFSCMVGHEALPLAFTQK.T | CDNA FLJ14473 fis, clone MAMMA1001080, highly similar to Homo sapiens SNC73 protein (SNC73) mRNA | BCP & HP |
| K.KGDTFSCM*VGHEALPLAFTQK.T | CDNA FLJ14473 fis, clone MAMMA1001080, highly similar to Homo sapiens SNC73 protein (SNC73) mRNA | BCP & HP |
| K.KGDTFSCMVGHEALPLAFTQK.T | CDNA FLJ14473 fis, clone MAMMA1001080, highly similar to Homo sapiens SNC73 protein (SNC73) mRNA | BCP & HP |
| K.NSLYLQMNSLR.A | CDNA FLJ14473 fis, clone MAMMA1001080, highly similar to Homo sapiens SNC73 protein (SNC73) mRNA | BCP & HP |
| K.SAVQGPPER.D | CDNA FLJ14473 fis, clone MAMMA1001080, highly similar to Homo sapiens SNC73 protein (SNC73) mRNA | BCP & HP |
| K.SAVQGPPERDLCGCYSVSSVLPGCAEPWNHGK.T | CDNA FLJ14473 fis, clone MAMMA1001080, highly similar to Homo sapiens SNC73 protein (SNC73) mRNA | BCP & HP |
| R.WLQGSQELPREK.Y | CDNA FLJ14473 fis, clone MAMMA1001080, highly similar to Homo sapiens SNC73 protein (SNC73) mRNA | BCP & HP |
| K.SGNTFRPEVHLLPPPSEELALNELVTLTCLAR.G | CDNA FLJ14473 fis, clone MAMMA1001080, highly similar to Homo sapiens SNC73 protein (SNC73) mRNA | BCP & HP |
| K.TFTCTAAYPESK.T | CDNA FLJ14473 fis, clone MAMMA1001080, highly similar to Homo sapiens SNC73 protein (SNC73) mRNA | BCP & HP |
| K.GDTFSCM*VGHEALPLAFTQK.T | CDNA FLJ14473 fis, clone MAMMA1001080, highly similar to Homo sapiens SNC73 protein (SNC73) mRNA | BCP & HP |
| K.VFPLSLCSTQPDGNVVIACLVQGFFPQEPLSVTWSESGQGVTAR.N | CDNA FLJ14473 fis, clone MAMMA1001080, highly similar to Homo sapiens SNC73 protein (SNC73) mRNA | BCP & HP |
| H.LLPPPSEELALNELVTLTCLAR.G | CDNA FLJ14473 fis, clone MAMMA1001080, highly similar to Homo sapiens SNC73 protein (SNC73) mRNA | BCP & HP |
| Q.GTLVTVSSASPTSPK.V | CDNA FLJ14473 fis, clone MAMMA1001080, highly similar to Homo sapiens SNC73 protein (SNC73) mRNA | BCP & HP |
| R.DASGVTFTWTPSSGK.S | CDNA FLJ14473 fis, clone MAMMA1001080, highly similar to Homo sapiens SNC73 protein (SNC73) mRNA | BCP & HP |
| R.DLCGCYSVSSVLPGCAEPWNHGK.T | CDNA FLJ14473 fis, clone MAMMA1001080, highly similar to Homo sapiens SNC73 protein (SNC73) mRNA | BCP & HP |
| R.DLCGCYSVSSVLPGCAEPWNHGKTFTCTAAYPESK.T | CDNA FLJ14473 fis, clone MAMMA1001080, highly similar to Homo sapiens SNC73 protein (SNC73) mRNA | BCP & HP |
| R.EKYLTWASR.Q | CDNA FLJ14473 fis, clone MAMMA1001080, highly similar to Homo sapiens SNC73 protein (SNC73) mRNA | BCP & HP |
| R.GFSPKDVLVR.W | CDNA FLJ14473 fis, clone MAMMA1001080, highly similar to Homo sapiens SNC73 protein (SNC73) mRNA | BCP & HP |
| R.NFPPSQDASGDLYTTSSQLTLPATQCLAGK.S | CDNA FLJ14473 fis, clone MAMMA1001080, highly similar to Homo sapiens SNC73 protein (SNC73) mRNA | BCP & HP |
| R.QEPSQGTTTFAVTSILR.V | CDNA FLJ14473 fis, clone MAMMA1001080, highly similar to Homo sapiens SNC73 protein (SNC73) mRNA | BCP & HP |
| R.VAAEDWK.K | CDNA FLJ14473 fis, clone MAMMA1001080, highly similar to Homo sapiens SNC73 protein (SNC73) mRNA | BCP & HP |
| R.VDDTAVYYCAR.D | CDNA FLJ14473 fis, clone MAMMA1001080, highly similar to Homo sapiens SNC73 protein (SNC73) mRNA | BCP & HP |
| R.WLQGSQELPR.E | CDNA FLJ14473 fis, clone MAMMA1001080, highly similar to Homo sapiens SNC73 protein (SNC73) mRNA | BCP & HP |
| K.TPLTATLSK.S | CDNA FLJ14473 fis, clone MAMMA1001080, highly similar to Homo sapiens SNC73 protein (SNC73) mRNA | BCP & HP |
| K.SIIHIHLLLR.H | CDNA FLJ37035 fis, clone BRACE2011545 | BCP & HP |
| R.AAWLMVMGTRTRR.A | CDNA FLJ41116 fis, clone BRACE1000572 | BCP & HP |
| R.TLRAARLMM*MGTRTRR.A | CDNA FLJ41116 fis, clone BRACE1000572 | BCP & HP |
| R.DASGATFTWTPSSGK.S | CDNA FLJ41981 fis, clone SMINT2011888, highly similar to Protein Tro alpha1 H,myeloma | BCP & HP |
| H.LLPPPSEELALNELVTLTCLAR.G | CDNA FLJ41981 fis, clone SMINT2011888, highly similar to Protein Tro alpha1 H,myeloma | BCP & HP |
| K.GDTFSCM*VGHEALPLAFTQK.T | CDNA FLJ41981 fis, clone SMINT2011888, highly similar to Protein Tro alpha1 H,myeloma | BCP & HP |
| K.GDTFSCMVGHEALPLAFTQK.T | CDNA FLJ41981 fis, clone SMINT2011888, highly similar to Protein Tro alpha1 H,myeloma | BCP & HP |
| K.KGDTFSCM*VGHEALPLAFTQK.T | CDNA FLJ41981 fis, clone SMINT2011888, highly similar to Protein Tro alpha1 H,myeloma | BCP & HP |
| K.KGDTFSCMVGHEALPLAFTQK.T | CDNA FLJ41981 fis, clone SMINT2011888, highly similar to Protein Tro alpha1 H,myeloma | BCP & HP |
| K.SAVQGPPER.D | CDNA FLJ41981 fis, clone SMINT2011888, highly similar to Protein Tro alpha1 H,myeloma | BCP & HP |
| K.SGNTFRPEVHLLPPPSEELALNELVTLTCLAR.G | CDNA FLJ41981 fis, clone SMINT2011888, highly similar to Protein Tro alpha1 H,myeloma | BCP & HP |
| K.HYTNPSQDVTVPCPVPPPPPCCHPR.L | CDNA FLJ41981 fis, clone SMINT2011888, highly similar to Protein Tro alpha1 H,myeloma | BCP & HP |
| R.AEDTAVYYCAR.R | CDNA FLJ41981 fis, clone SMINT2011888, highly similar to Protein Tro alpha1 H,myeloma | BCP & HP |
| R.WLQGSQELPREK.Y | CDNA FLJ41981 fis, clone SMINT2011888, highly similar to Protein Tro alpha1 H,myeloma | BCP & HP |
| R.DLCGCYSVSSVLPGCAQPWNHGETFTCTAAHPELK.T | CDNA FLJ41981 fis, clone SMINT2011888, highly similar to Protein Tro alpha1 H,myeloma | BCP & HP |
| R.EKYLTWASR.Q | CDNA FLJ41981 fis, clone SMINT2011888, highly similar to Protein Tro alpha1 H,myeloma | BCP & HP |
| R.GFSPKDVLVR.W | CDNA FLJ41981 fis, clone SMINT2011888, highly similar to Protein Tro alpha1 H,myeloma | BCP & HP |
| R.GTQVTVSSASPTSPK.V | CDNA FLJ41981 fis, clone SMINT2011888, highly similar to Protein Tro alpha1 H,myeloma | BCP & HP |
| R.NFPPSQDASGDLYTTSSQLTLPATQCPDGK.S | CDNA FLJ41981 fis, clone SMINT2011888, highly similar to Protein Tro alpha1 H,myeloma | BCP & HP |
| R.QEPSQGTTTFAVTSILR.V | CDNA FLJ41981 fis, clone SMINT2011888, highly similar to Protein Tro alpha1 H,myeloma | BCP & HP |
| R.VAAEDWK.K | CDNA FLJ41981 fis, clone SMINT2011888, highly similar to Protein Tro alpha1 H,myeloma | BCP & HP |
| R.WLQGSQELPR.E | CDNA FLJ41981 fis, clone SMINT2011888, highly similar to Protein Tro alpha1 H,myeloma | BCP & HP |
| K.YLTWASR.Q | CDNA FLJ41981 fis, clone SMINT2011888, highly similar to Protein Tro alpha1 H,myeloma | BCP & HP |
| K.YYYVCQYCPAGNWANR.L | cDNA FLJ75207 | BCP & HP |
| R.LYVPYEQGAPCASCPDNCDDGLCTNGCKYEDLYSNCK.S | cDNA FLJ75207 | BCP & HP |
| R.EIVNKHNELR.R | cDNA FLJ75207 | BCP & HP |
| K.YEDLYSNCK.S | cDNA FLJ75207 | BCP & HP |
| K.SLKLTLTCK.H | cDNA FLJ75207 | BCP & HP |
| K.MEWNKEAAANAQK.W | cDNA FLJ75207 | BCP & HP |
| K.M*EWNKEAAANAQK.W | cDNA FLJ75207 | BCP & HP |
| A.NEDKDPAFTALLTTQTQVQR.E | cDNA FLJ75207 | BCP & HP |
| R.LYVPYEQGAPCASCPDNCDDGLCTNGCK.Y | cDNA FLJ75207 | BCP & HP |
| K.AVQKSTSENQTEWNAR.D | Centromere protein J | BCP & HP |
| K.NYLPMQGNPPRRSKSAPPRDLGNLDK.G | Centromere protein J | BCP & HP |
| K.CLVVCDSNPATDSK.G | Cerebellin-4 precursor | BCP & HP |
| K.VNKDDEEFIESNKM*HAINGR.M | Ceruloplasmin precursor | BCP & HP |
| L.LATEEQSPGEGDGNCVTR.I | Ceruloplasmin precursor | BCP & HP |
| K.QKYTVNQCR.R | Ceruloplasmin precursor | BCP & HP |
| K.TYCSEPEKVDK.D | Ceruloplasmin precursor | BCP & HP |
| K.TYSDHPEK.V | Ceruloplasmin precursor | BCP & HP |
| K.TYSDHPEKVNKDDEEFIESNK.M | Ceruloplasmin precursor | BCP & HP |
| K.TYSDHPEKVNKDDEEFIESNKM*HAINGR.M | Ceruloplasmin precursor | BCP & HP |
| K.TYSDHPEKVNKDDEEFIESNKMHAINGR.M | Ceruloplasmin precursor | BCP & HP |
| K.NNEGTYYSPNYNPQSR.S | Ceruloplasmin precursor | BCP & HP |
| K.VNKDDEEFIESNK.M | Ceruloplasmin precursor | BCP & HP |
| K.NMATRPYSIHAHGVQTESSTVTPTLPGETLTYVWK.I | Ceruloplasmin precursor | BCP & HP |
| K.VNKDDEEFIESNKMHAINGR.M | Ceruloplasmin precursor | BCP & HP |
| K.VYPGEQYTYM*LLATEEQSPGEGDGNCVTR.I | Ceruloplasmin precursor | BCP & HP |
| K.VYPGEQYTYMLLATEEQSPGEGDGNCVTR.I | Ceruloplasmin precursor | BCP & HP |
| K.VYVHLK.N | Ceruloplasmin precursor | BCP & HP |
| K.WYLFGM*GNEVDVHAAFFHGQALTNK.N | Ceruloplasmin precursor | BCP & HP |
| K.WYLFGMGNEVDVHAAFFHGQALTNK.N | Ceruloplasmin precursor | BCP & HP |
| K.YTVNQCR.R | Ceruloplasmin precursor | BCP & HP |
| K.M*HSMNGFMYGNQPGLTMCK.G | Ceruloplasmin precursor | BCP & HP |
| K.VDKDNEDFQESNR.M | Ceruloplasmin precursor | BCP & HP |
| K.MHSMNGFM*YGNQPGLTMCK.G | Ceruloplasmin precursor | BCP & HP |
| K.M*YYSAVDPTK.D | Ceruloplasmin precursor | BCP & HP |
| K.M*YYSAVDPTKDIFTGLIGPM*K.I | Ceruloplasmin precursor | BCP & HP |
| K.M*YYSAVDPTKDIFTGLIGPMK.I | Ceruloplasmin precursor | BCP & HP |
| K.MHAINGR.M | Ceruloplasmin precursor | BCP & HP |
| K.MHSM*NGFM*YGNQPGLTM*CK.G | Ceruloplasmin precursor | BCP & HP |
| K.MHSM*NGFM*YGNQPGLTMCK.G | Ceruloplasmin precursor | BCP & HP |
| K.MHSM*NGFMYGNQPGLTM*CK.G | Ceruloplasmin precursor | BCP & HP |
| K.PVWLGFLGPIIK.A | Ceruloplasmin precursor | BCP & HP |
| K.MHSMNGFM*YGNQPGLTM*CK.G | Ceruloplasmin precursor | BCP & HP |
| L.YLQYTDETFR.T | Ceruloplasmin precursor | BCP & HP |
| K.MHSMNGFMYGNQPGLTM*CK.G | Ceruloplasmin precursor | BCP & HP |
| K.MHSMNGFMYGNQPGLTMCK.G | Ceruloplasmin precursor | BCP & HP |
| K.MYYSAVDPTK.D | Ceruloplasmin precursor | BCP & HP |
| K.MYYSAVDPTKDIFTGLIGPM*K.I | Ceruloplasmin precursor | BCP & HP |
| K.MYYSAVDPTKDIFTGLIGPMK.I | Ceruloplasmin precursor | BCP & HP |
| K.MYYSAVDPTKDIFTGLIGPMKICK.K | Ceruloplasmin precursor | BCP & HP |
| K.NLASRPYTFHSHGITYYK.E | Ceruloplasmin precursor | BCP & HP |
| K.NM*ATRPYSIHAHGVQTESSTVTPTLPGETLTYVWK.I | Ceruloplasmin precursor | BCP & HP |
| K.MHSM*NGFMYGNQPGLTMCK.G | Ceruloplasmin precursor | BCP & HP |
| R.M*YSVNGYTFGSLPGLSM*CAEDRVK.W | Ceruloplasmin precursor | BCP & HP |
| L.ATEEQSPGEGDGNCVTR.I | Ceruloplasmin precursor | BCP & HP |
| R.IYHSHIDAPK.D | Ceruloplasmin precursor | BCP & HP |
| R.IYHSHIDAPKDIASGLIGPLIICK.K | Ceruloplasmin precursor | BCP & HP |
| R.KAEEEHLGILGPQLHADVGDK.V | Ceruloplasmin precursor | BCP & HP |
| R.KAEEEHLGILGPQLHADVGDKVK.I | Ceruloplasmin precursor | BCP & HP |
| R.KERGPEEEHLGILGPVIWAEVGDTIR.V | Ceruloplasmin precursor | BCP & HP |
| R.M*FTTAPDQVDK.E | Ceruloplasmin precursor | BCP & HP |
| R.IDTINLFPATLFDAYMVAQNPGEWM*LSCQNLNHLK.A | Ceruloplasmin precursor | BCP & HP |
| R.M*YSVNGYTFGSLPGLSM*CAEDR.V | Ceruloplasmin precursor | BCP & HP |
| R.IDTINLFPATLFDAYM*VAQNPGEWMLSCQNLNHLK.A | Ceruloplasmin precursor | BCP & HP |
| R.M*YSVNGYTFGSLPGLSMCAEDR.V | Ceruloplasmin precursor | BCP & HP |
| R.M*YSVNGYTFGSLPGLSMCAEDRVK.W | Ceruloplasmin precursor | BCP & HP |
| R.MFTTAPDQVDK.E | Ceruloplasmin precursor | BCP & HP |
| R.MFTTAPDQVDKEDEDFQESNK.M | Ceruloplasmin precursor | BCP & HP |
| R.MFTTAPDQVDKEDEDFQESNKMHSMNGFMYGNQPGLTMCK.G | Ceruloplasmin precursor | BCP & HP |
| R.MYSVNGYTFGSLPGLSM*CAEDR.V | Ceruloplasmin precursor | BCP & HP |
| Y.SIHAHGVQTESSTVTPTLPGETLTYVWK.I | Ceruloplasmin precursor | BCP & HP |
| Y.YSAVDPTKDIFTGLIGPMK.I | Ceruloplasmin precursor | BCP & HP |
| R.M*FTTAPDQVDKEDEDFQESNK.M | Ceruloplasmin precursor | BCP & HP |
| R.EYTDASFTNR.K | Ceruloplasmin precursor | BCP & HP |
| M.LLATEEQSPGEGDGNCVTR.I | Ceruloplasmin precursor | BCP & HP |
| N.KNNEGTYYSPNYNPQSR.S | Ceruloplasmin precursor | BCP & HP |
| R.ADDKVYPGEQYTY.M | Ceruloplasmin precursor | BCP & HP |
| R.ADDKVYPGEQYTYM*LLATEEQSPGEGDGNCVTR.I | Ceruloplasmin precursor | BCP & HP |
| R.ADDKVYPGEQYTYMLLATEEQSPGEGDGNCVTR.I | Ceruloplasmin precursor | BCP & HP |
| R.DTANLFPQTSLTLHM*WPDTEGTFNVECLTTDHYTGGM*K.Q | Ceruloplasmin precursor | BCP & HP |
| R.DTANLFPQTSLTLHM*WPDTEGTFNVECLTTDHYTGGMK.Q | Ceruloplasmin precursor | BCP & HP |
| R.IDTINLFPATLFDAYMVAQNPGEWMLSCQNLNHLK.A | Ceruloplasmin precursor | BCP & HP |
| R.DTANLFPQTSLTLHMWPDTEGTFNVECLTTDHYTGGMK.Q | Ceruloplasmin precursor | BCP & HP |
| K.TYCSEPEKVDKDNEDFQESNR.M | Ceruloplasmin precursor | BCP & HP |
| R.EYTDASFTNRK.E | Ceruloplasmin precursor | BCP & HP |
| R.FNKNNEGTYYSPNYNPQS.R | Ceruloplasmin precursor | BCP & HP |
| R.FNKNNEGTYYSPNYNPQSR.S | Ceruloplasmin precursor | BCP & HP |
| R.GPEEEHLGILGPVIWAEVGDTIR.V | Ceruloplasmin precursor | BCP & HP |
| R.GVYSSDVFDIFPGTYQTLEM*FPR.T | Ceruloplasmin precursor | BCP & HP |
| R.GVYSSDVFDIFPGTYQTLEMFPR.T | Ceruloplasmin precursor | BCP & HP |
| R.HYYIAAEEIIWNYAPSGIDIFTK.E | Ceruloplasmin precursor | BCP & HP |
| R.IDTINLFPATLFDAYM*VAQNPGEWM*LSCQNLNHLK.A | Ceruloplasmin precursor | BCP & HP |
| R.DTANLFPQTSLTLHMWPDTEGTFNVECLTTDHYTGGM*K.Q | Ceruloplasmin precursor | BCP & HP |
| A.KMYYSAVDPTKDIFTGLIGPMK.I | Ceruloplasmin precursor | BCP & HP |
| R.QYTDSTFRVPVERK.A | Ceruloplasmin precursor | BCP & HP |
| R.QYTDSTFRVPVER.K | Ceruloplasmin precursor | BCP & HP |
| R.QYTDSTFR.V | Ceruloplasmin precursor | BCP & HP |
| R.QSEDSTFYLGER.T | Ceruloplasmin precursor | BCP & HP |
| R.QKDVDKEFYLFPTVFDENESLLLEDNIR.M | Ceruloplasmin precursor | BCP & HP |
| R.PYSIHAHGVQTESSTVTPTLPGETLTYVWK.I | Ceruloplasmin precursor | BCP & HP |
| R.MYSVNGYTFGSLPGLSMCAEDRVK.W | Ceruloplasmin precursor | BCP & HP |
| I.FPGTYQTLEMFPR.T | Ceruloplasmin precursor | BCP & HP |
| R.MYSVNGYTFGSLPGLSM*CAEDRVK.W | Ceruloplasmin precursor | BCP & HP |
| R.RDTANLFPQTSLTLHMWPDTEGTFNVECLTTDHYTGGMK.Q | Ceruloplasmin precursor | BCP & HP |
| A.PDQVDKEDEDFQESNK.M | Ceruloplasmin precursor | BCP & HP |
| D.LYSGLIGPLIVCR.R | Ceruloplasmin precursor | BCP & HP |
| D.PTKDIFTGLIGPMK.I | Ceruloplasmin precursor | BCP & HP |
| F.PATLFDAYM*VAQNPGEWMLSCQNLNHLK.A | Ceruloplasmin precursor | BCP & HP |
| F.PATLFDAYMVAQNPGEWMLSCQNLNHLK.A | Ceruloplasmin precursor | BCP & HP |
| F.PGTYQTLEMFPR.T | Ceruloplasmin precursor | BCP & HP |
| H.IHAGMETTYTVLQNEDTK.S | Ceruloplasmin precursor | BCP & HP |
| H.SMNGFMYGNQPGLTMCK.G | Ceruloplasmin precursor | BCP & HP |
| R.MYSVNGYTFGSLPGLSMCAEDR.V | Ceruloplasmin precursor | BCP & HP |
| R.TYYIAAVEVEWDYSPQR.E | Ceruloplasmin precursor | BCP & HP |
| Y.PLSIEPIGVR.F | Ceruloplasmin precursor | BCP & HP |
| Y.MLLATEEQSPGEGDGNCVTR.I | Ceruloplasmin precursor | BCP & HP |
| Y.M*LLATEEQSPGEGDGNCVTR.I | Ceruloplasmin precursor | BCP & HP |
| Y.LFPTVFDENESLLLEDNIR.M | Ceruloplasmin precursor | BCP & HP |
| W.AYYSTVDQVK.D | Ceruloplasmin precursor | BCP & HP |
| V.PPSASHVAPTETFTYEWTVPK.E | Ceruloplasmin precursor | BCP & HP |
| V.GPTNADPVCLAK.M | Ceruloplasmin precursor | BCP & HP |
| V.AQNPGEWMLSCQNLNHLK.A | Ceruloplasmin precursor | BCP & HP |
| R.RDTANLFPQTSLTLHM*WPDTEGTFNVECLTTDHYTGGMK.Q | Ceruloplasmin precursor | BCP & HP |
| R.VTFHNKGAYPLSIEPIGVR.F | Ceruloplasmin precursor | BCP & HP |
| R.RDTANLFPQTSLTLHMWPDTEGTFNVECLTTDHYTGGM*K.Q | Ceruloplasmin precursor | BCP & HP |
| R.TTIEKPVWLGFLGPIIK.A | Ceruloplasmin precursor | BCP & HP |
| R.TPGIWLLHCHVTDHIHAGM*ETTYTVLQNEDTK.S | Ceruloplasmin precursor | BCP & HP |
| R.SVPPSASHVAPTETFTYEWTVPKEVGPTNADPVCLAK.M | Ceruloplasmin precursor | BCP & HP |
| K.TYSDHPEKVNK.D | Ceruloplasmin precursor | BCP & HP |
| R.SGAGTEDSACIPWAYYSTVDQVKDLYSGLIGPLIVCR.R | Ceruloplasmin precursor | BCP & HP |
| K.M*HSMNGFM*YGNQPGLTMCK.G | Ceruloplasmin precursor | BCP & HP |
| R.RQSEDSTFYLGER.T | Ceruloplasmin precursor | BCP & HP |
| R.SGAGTEDSACIPWAYYSTVDQVK.D | Ceruloplasmin precursor | BCP & HP |
| T.TAPDQVDKEDEDFQESNK.M | Ceruloplasmin precursor | BCP & HP |
| K.LVYREYTDASFTNR.K | Ceruloplasmin precursor | BCP & HP |
| K.GAYPLSIEPIGVR.F | Ceruloplasmin precursor | BCP & HP |
| K.GDSVVWYLFSAGNEADVHGIYFSGNTYLWR.G | Ceruloplasmin precursor | BCP & HP |
| K.HRGVYSSDVFDIFPGTYQTLEM*FPR.T | Ceruloplasmin precursor | BCP & HP |
| K.HRGVYSSDVFDIFPGTYQTLEMFPR.T | Ceruloplasmin precursor | BCP & HP |
| K.HYYIGIIETTWDYASDHGEK.K | Ceruloplasmin precursor | BCP & HP |
| K.HYYIGIIETTWDYASDHGEKK.L | Ceruloplasmin precursor | BCP & HP |
| K.ICKKGSLHANGRQKDVDKEFYLFPTVFDENESLLLEDNIR.M | Ceruloplasmin precursor | BCP & HP |
| K.KALYLQYTDETFR.T | Ceruloplasmin precursor | BCP & HP |
| K.EVGPTNADPVCLAK.M | Ceruloplasmin precursor | BCP & HP |
| K.LISVDTEHSNIYLQNGPDR.I | Ceruloplasmin precursor | BCP & HP |
| K.KLISVDTEHSNIYLQNGPDRIGR.L | Ceruloplasmin precursor | BCP & HP |
| K.LVYREYTDASFTNRK.E | Ceruloplasmin precursor | BCP & HP |
| K.M*HAINGR.M | Ceruloplasmin precursor | BCP & HP |
| K.M*HSM*NGFM*YGNQPGLTMCK.G | Ceruloplasmin precursor | BCP & HP |
| K.M*HSM*NGFMYGNQPGLTM*CK.G | Ceruloplasmin precursor | BCP & HP |
| I.PWAYYSTVDQVK.D | Ceruloplasmin precursor | BCP & HP |
| R.SVPPSASHVAPTETFTYEWTVPK.E | Ceruloplasmin precursor | BCP & HP |
| K.M*HSMNGFM*YGNQPGLTM*CK.G | Ceruloplasmin precursor | BCP & HP |
| K.M*HSM*NGFMYGNQPGLTMCK.G | Ceruloplasmin precursor | BCP & HP |
| K.KLISVDTEHSNIYLQNGPDR.I | Ceruloplasmin precursor | BCP & HP |
| K.DIASGLIGPLIICK.K | Ceruloplasmin precursor | BCP & HP |
| I.SVDTEHSNIYLQNGPDR.I | Ceruloplasmin precursor | BCP & HP |
| K.AEEEHLGILGPQLHADVGDKVK.I | Ceruloplasmin precursor | BCP & HP |
| K.AETGDKVYVHLK.N | Ceruloplasmin precursor | BCP & HP |
| K.LISVDTEHSNIYLQNGPDRIGR.L | Ceruloplasmin precursor | BCP & HP |
| K.ERGPEEEHLGILGPVIWAEVGDTIR.V | Ceruloplasmin precursor | BCP & HP |
| K.AGLQAFFQVQECNK.S | Ceruloplasmin precursor | BCP & HP |
| K.AEEEHLGILGPQLHADVGDK.V | Ceruloplasmin precursor | BCP & HP |
| K.DDEEFIESNK.M | Ceruloplasmin precursor | BCP & HP |
| K.DIASGLIGPLIICKK.D | Ceruloplasmin precursor | BCP & HP |
| K.DIFTGLIGPM*K.I | Ceruloplasmin precursor | BCP & HP |
| K.DNEDFQESNRM*YSVNGYTFGSLPGLSMCAEDR.V | Ceruloplasmin precursor | BCP & HP |
| K.DIFTGLIGPMK.I | Ceruloplasmin precursor | BCP & HP |
| K.EFYLFPTVFDENESLLLEDNIR.M | Ceruloplasmin precursor | BCP & HP |
| K.DLYSGLIGPLIVCR.R | Ceruloplasmin precursor | BCP & HP |
| K.DNEDFQESNR.M | Ceruloplasmin precursor | BCP & HP |
| K.DVDKEFYLFPTVFDENESLLLEDNIR.M | Ceruloplasmin precursor | BCP & HP |
| K.DSLDKEKEK.H | Ceruloplasmin precursor | BCP & HP |
| K.ALYLQYTDETFR.T | Ceruloplasmin precursor | BCP & HP |
| K.TMFICK.S | CFI protein | BCP & HP |
| K.TM*FICK.S | CFI protein | BCP & HP |
| K.LVDQDKTMFICK.S | CFI protein | BCP & HP |
| K.LVDQDKTM*FICK.S | CFI protein | BCP & HP |
| K.SLECLHPGTK.F | CFI protein | BCP & HP |
| K.VFCQPWQR.C | CFI protein | BCP & HP |
| K.VTYTSQEDLVEK.K | CFI protein | BCP & HP |
| K.YTHLSCDK.V | CFI protein | BCP & HP |
| K.YTHLSCDKVFCQPWQR.C | CFI protein | BCP & HP |
| R.CIEGTCVCK.L | CFI protein | BCP & HP |
| R.CIEGTCVCKLPYQCPK.N | CFI protein | BCP & HP |
| R.EANVACLDLGFQQGADTQR.R | CFI protein | BCP & HP |
| R.EANVACLDLGFQQGADTQRR.F | CFI protein | BCP & HP |
| R.TM*GYQDFADVVCYTQK.A | CFI protein | BCP & HP |
| K.VTYTSQEDLVEKK.C | CFI protein | BCP & HP |
| R.TM*GYQDFADVVCYTQKADSPMDDFFQCVNGK.Y | CFI protein | BCP & HP |
| R.GLETSLAECTFTK.R | CFI protein | BCP & HP |
| R.GLETSLAECTFTKR.R | CFI protein | BCP & HP |
| R.RSFPTYCQQK.S | CFI protein | BCP & HP |
| R.TMGYQDFADVVCYTQK.A | CFI protein | BCP & HP |
| R.SFPTYCQQK.S | CFI protein | BCP & HP |
| K.KYTHLSCDK.V | CFI protein | BCP & HP |
| R.TMGYQDFADVVCYTQKADSPMDDFFQCVNGK.Y | CFI protein | BCP & HP |
| C.KVTYTSQEDLVEK.K | CFI protein | BCP & HP |
| C.KVTYTSQEDLVEKK.C | CFI protein | BCP & HP |
| K.ACDGINDCGDQSDELCCK.A | CFI protein | BCP & HP |
| K.ADSPM*DDFFQCVNGK.Y | CFI protein | BCP & HP |
| K.ADSPMDDFFQCVNGK.Y | CFI protein | BCP & HP |
| K.HGNTDSEGIVEVK.L | CFI protein | BCP & HP |
| R.RTMGYQDFADVVCYTQK.A | CFI protein | BCP & HP |
| K.NQFNDYTSKK.E | Cholinesterase precursor | BCP & HP |
| K.TQILVGVNKDEGTAFLVYGAPGFSK.D | Cholinesterase precursor | BCP & HP |
| K.ESILFHYTDWVDDQRPENYR.E | Cholinesterase precursor | BCP & HP |
| T.EDDIIIATK.N | Cholinesterase precursor | BCP & HP |
| R.WNNYMMDWK.N | Cholinesterase precursor | BCP & HP |
| R.FWTSFFPK.V | Cholinesterase precursor | BCP & HP |
| K.NQFNDYTSK.K | Cholinesterase precursor | BCP & HP |
| K.NIAAFGGNPK.S | Cholinesterase precursor | BCP & HP |
| K.VLEMTGNIDEAEWEWK.A | Cholinesterase precursor | BCP & HP |
| K.YLTLNTESTR.I | Cholinesterase precursor | BCP & HP |
| R.AILQSGSFNAPWAVTSLYEAR.N | Cholinesterase precursor | BCP & HP |
| R.EALGDVVGDYNFICPALEFTK.K | Cholinesterase precursor | BCP & HP |
| K.DNNSIITR.K | Cholinesterase precursor | BCP & HP |
| K.FSEWGNNAFFYYFEHR.S | Cholinesterase precursor | BCP & HP |
| K.IFFPGVSEFGK.E | Cholinesterase precursor | BCP & HP |
| K.KTQILVGVNKDEGTAFLVYGAPGFSK.D | Cholinesterase precursor | BCP & HP |
| R.EALGDVVGDYNFICPALEFTKK.F | Cholinesterase precursor | BCP & HP |
| K.VLEM*TGNIDEAEWEWK.A | Cholinesterase precursor | BCP & HP |
| K.ASRPDATCASPAK.F | Chondroadherin precursor | BCP & HP |
| R.SGSGLVGR.Q | Clusterin precursor | BCP & HP |
| R.VTTVASHTSDSDVPSGVTEVVVK.L | Clusterin precursor | BCP & HP |
| R.IDSLLENDRQQTHMLDVMQDHFSR.A | Clusterin precursor | BCP & HP |
| R.KTLLSNLEEAK.K | Clusterin precursor | BCP & HP |
| R.KTLLSNLEEAKK.K | Clusterin precursor | BCP & HP |
| R.LANLTQGEDQYYLR.V | Clusterin precursor | BCP & HP |
| R.QQTHM*LDVM*QDHFSR.A | Clusterin precursor | BCP & HP |
| R.QQTHMLDVMQDHFSR.A | Clusterin precursor | BCP & HP |
| R.QQTHMLDVM*QDHFSR.A | Clusterin precursor | BCP & HP |
| R.IDSLLENDR.Q | Clusterin precursor | BCP & HP |
| R.FFTREPQDTYHYLPFSLPHR.R | Clusterin precursor | BCP & HP |
| R.IDSLLENDRQQTHMLDVM*QDHFSR.A | Clusterin precursor | BCP & HP |
| K.LFDSDPITVTVPVEVSRK.N | Clusterin precursor | BCP & HP |
| L.GDQTVSDNELQEMSNQGSK.Y | Clusterin precursor | BCP & HP |
| L.GDQTVSDNELQEM*SNQGSK.Y | Clusterin precursor | BCP & HP |
| K.YVNKEIQNAVNGVK.Q | Clusterin precursor | BCP & HP |
| G.DQTVSDNELQEMSNQGSK.Y | Clusterin precursor | BCP & HP |
| K.TLLSNLEEAKK.K | Clusterin precursor | BCP & HP |
| K.TLLSNLEEAK.K | Clusterin precursor | BCP & HP |
| R.ASSIIDELFQDRFFTR.E | Clusterin precursor | BCP & HP |
| K.TLIEKTNEER.K | Clusterin precursor | BCP & HP |
| R.EILSVDCSTNNPSQAK.L | Clusterin precursor | BCP & HP |
| K.LFDSDPITVTVPVEVSR.K | Clusterin precursor | BCP & HP |
| K.FMETVAEK.A | Clusterin precursor | BCP & HP |
| R.RELDESLQVAER.L | Clusterin precursor | BCP & HP |
| K.FM*ETVAEK.A | Clusterin precursor | BCP & HP |
| K.EIQNAVNGVK.Q | Clusterin precursor | BCP & HP |
| K.CREILSVDCSTNNPSQAK.L | Clusterin precursor | BCP & HP |
| R.EPQDTYHYLPFSLPHR.R | Clusterin precursor | BCP & HP |
| K.TLIEKTNEERK.T | Clusterin precursor | BCP & HP |
| R.ELDESLQVAER.L | Clusterin precursor | BCP & HP |
| R.QQTHM*LDVMQDHFSR.A | Clusterin precursor | BCP & HP |
| G.DQTVSDNELQEM*SNQGSK.Y | Clusterin precursor | BCP & HP |
| R.ASSIIDELFQDR.F | Clusterin precursor | BCP & HP |
| K.NCELDVTCNIK.N | Coagulation factor IX precursor | BCP & HP |
| K.FGSGYVSGWGR.V | Coagulation factor IX precursor | BCP & HP |
| R.SALVLQYLR.V | Coagulation factor IX precursor | BCP & HP |
| R.VPLVDR.A | Coagulation factor IX precursor | BCP & HP |
| R.VSVSQTSK.L | Coagulation factor IX precursor | BCP & HP |
| R.VVGGEDAKPGQFPWQVVLNGK.V | Coagulation factor IX precursor | BCP & HP |
| R.YVNWIKEK.T | Coagulation factor IX precursor | BCP & HP |
| K.ITVVAGEHNIEETEHTEQKR.N | Coagulation factor IX precursor | BCP & HP |
| K.ITVVAGEHNIEETEHTEQK.R | Coagulation factor IX precursor | BCP & HP |
| K.NSADNKVVCSCTEGYR.L | Coagulation factor IX precursor | BCP & HP |
| K.FTIYNNMFCAGFHEGGR.D | Coagulation factor IX precursor | BCP & HP |
| K.SCEPAVPFPCGR.V | Coagulation factor IX precursor | BCP & HP |
| R.DSCQGDSGGPHVTEVEGTSFLTGIISWGEECAMK.G | Coagulation factor IX precursor | BCP & HP |
| K.YNHDIALLELDEPLVLNSYVTPICIADKEYTNIFLK.F | Coagulation factor IX precursor | BCP & HP |
| K.WIVTAAHCVETGVK.I | Coagulation factor IX precursor | BCP & HP |
| K.VVCSCTEGYR.L | Coagulation factor IX precursor | BCP & HP |
| K.VDAFCGGSIVNEK.W | Coagulation factor IX precursor | BCP & HP |
| R.IIPHHNYNAAINK.Y | Coagulation factor IX precursor | BCP & HP |
| K.GKYGIYTK.V | Coagulation factor IX precursor | BCP & HP |
| R.SQHLDNFSNQIGK.H | Coagulation factor V | BCP & HP |
| R.AGMQTPFLIM*DR.D | Coagulation factor V | BCP & HP |
| R.AGM*QTPFLIM*DR.D | Coagulation factor V | BCP & HP |
| R.AEVDDVIQVR.F | Coagulation factor V | BCP & HP |
| K.SHEFHAINGMIYSLPGLK.M | Coagulation factor V | BCP & HP |
| K.WNILEFDEPTENDAQCLTRPYYSDVDIMR.D | Coagulation factor V | BCP & HP |
| K.WIISSLTPK.H | Coagulation factor V | BCP & HP |
| K.VMYTQYEDESFTK.H | Coagulation factor V | BCP & HP |
| R.AWAYYSAVNPEKDIHSGLIGPLLICQK.G | Coagulation factor V | BCP & HP |
| K.VM*YTQYEDESFTK.H | Coagulation factor V | BCP & HP |
| K.SYTIHYSEQGVEWKPYR.L | Coagulation factor V | BCP & HP |
| R.AWGESTPLANKPGK.Q | Coagulation factor V | BCP & HP |
| R.DIASGLIGLLLICK.S | Coagulation factor V | BCP & HP |
| R.ETDIEDSDDIPEDTTYK.K | Coagulation factor V | BCP & HP |
| R.ETDIEDSDDIPEDTTYKK.V | Coagulation factor V | BCP & HP |
| R.GEYEEHLGILGPIIR.A | Coagulation factor V | BCP & HP |
| R.KYLDSTFTK.R | Coagulation factor V | BCP & HP |
| R.LELFGCDIY.- | Coagulation factor V | BCP & HP |
| R.LELQGCEVNGCSTPLGMENGKIENK.Q | Coagulation factor V | BCP & HP |
| R.MRPWKDPPSDLLLLK.Q | Coagulation factor V | BCP & HP |
| R.NVMYFNGNSDASTIKENQFDPPIVAR.Y | Coagulation factor V | BCP & HP |
| R.SEAYNTFSER.R | Coagulation factor V | BCP & HP |
| K.FTVNNLAEPQKAPSHQQATTAGSPLR.H | Coagulation factor V | BCP & HP |
| K.QHQLGVWPLLPGSFK.T | Coagulation factor V | BCP & HP |
| R.HLSQDTGSPSGMRPWEDLPSQDTGSPSR.M | Coagulation factor V | BCP & HP |
| K.ASKPGWWLLNTEVGENQR.A | Coagulation factor V | BCP & HP |
| R.SSSPELSEMLEYDR.S | Coagulation factor V | BCP & HP |
| K.ADKPLSIHPQGIR.Y | Coagulation factor V | BCP & HP |
| K.MDDAVAPGR.E | Coagulation factor V | BCP & HP |
| K.LAAEFASKPWIQVDMQK.E | Coagulation factor V | BCP & HP |
| K.HTVNPNMKEDGILGPIIR.A | Coagulation factor V | BCP & HP |
| K.DSNM*PMDM*R.E | Coagulation factor V | BCP & HP |
| K.DSNM*PMDMR.E | Coagulation factor V | BCP & HP |
| K.EFNPLVIVGLSK.D | Coagulation factor V | BCP & HP |
| K.LSEGASYLDHTFPAEKMDDAVAPGR.E | Coagulation factor V | BCP & HP |
| K.ASEFLGYWEPR.L | Coagulation factor V | BCP & HP |
| K.M*DDAVAPGR.E | Coagulation factor V | BCP & HP |
| K.EKPQSTISGLLGPTLYAEVGDIIK.V | Coagulation factor V | BCP & HP |
| K.LSEGASYLDHTFPAEK.M | Coagulation factor V | BCP & HP |
| K.KITAIITQGCK.S | Coagulation factor V | BCP & HP |
| K.ITAIITQGCK.S | Coagulation factor V | BCP & HP |
| K.HTVNPNM*KEDGILGPIIR.A | Coagulation factor V | BCP & HP |
| K.FCENPDEVKR.D | Coagulation factor V | BCP & HP |
| K.EVIITGIQTQGAK.H | Coagulation factor V | BCP & HP |
| R.AWGESTPLANKPGK.Q | Coagulation factor V precursor | BCP & HP |
| K.SHEFHAINGMIYSLPGLK.M | Coagulation factor V precursor | BCP & HP |
| K.VMYTQYEDESFTK.H | Coagulation factor V precursor | BCP & HP |
| K.WIISSLTPK.H | Coagulation factor V precursor | BCP & HP |
| K.WNILEFDEPTENDAQCLTRPYYSDVDIMR.D | Coagulation factor V precursor | BCP & HP |
| R.AEVDDVIQVR.F | Coagulation factor V precursor | BCP & HP |
| K.SYTIHYSEQGVEWKPYR.L | Coagulation factor V precursor | BCP & HP |
| R.DIASGLIGLLLICK.S | Coagulation factor V precursor | BCP & HP |
| K.VM*YTQYEDESFTK.H | Coagulation factor V precursor | BCP & HP |
| R.AWAYYSAVNPEKDIHSGLIGPLLICQK.G | Coagulation factor V precursor | BCP & HP |
| R.AGMQTPFLIM*DR.D | Coagulation factor V precursor | BCP & HP |
| R.AGM*QTPFLIM*DR.D | Coagulation factor V precursor | BCP & HP |
| K.EVIITGIQTQGAK.H | Coagulation factor V precursor | BCP & HP |
| K.MDDAVAPGR.E | Coagulation factor V precursor | BCP & HP |
| K.KITAIITQGCK.S | Coagulation factor V precursor | BCP & HP |
| K.ITAIITQGCK.S | Coagulation factor V precursor | BCP & HP |
| K.HTVNPNMKEDGILGPIIR.A | Coagulation factor V precursor | BCP & HP |
| K.HTVNPNM*KEDGILGPIIR.A | Coagulation factor V precursor | BCP & HP |
| K.LSEGASYLDHTFPAEK.M | Coagulation factor V precursor | BCP & HP |
| K.FCENPDEVKR.D | Coagulation factor V precursor | BCP & HP |
| K.LSEGASYLDHTFPAEKMDDAVAPGR.E | Coagulation factor V precursor | BCP & HP |
| K.EKPQSTISGLLGPTLYAEVGDIIK.V | Coagulation factor V precursor | BCP & HP |
| K.EFNPLVIVGLSK.D | Coagulation factor V precursor | BCP & HP |
| K.DSNMPVDMR.E | Coagulation factor V precursor | BCP & HP |
| K.ASKPGWWLLNTEVGENQR.A | Coagulation factor V precursor | BCP & HP |
| K.ASEFLGYWEPR.L | Coagulation factor V precursor | BCP & HP |
| K.ADKPLSIHPQGIR.Y | Coagulation factor V precursor | BCP & HP |
| K.FTVNNLAEPQKAPSHQQATTAGSPLR.H | Coagulation factor V precursor | BCP & HP |
| R.NVMYFNGNSDASTIKENQFDPPIVAR.Y | Coagulation factor V precursor | BCP & HP |
| R.ETDIEDSDDIPEDTTYKK.V | Coagulation factor V precursor | BCP & HP |
| R.GEYEEHLGILGPIIR.A | Coagulation factor V precursor | BCP & HP |
| R.HLSQDTGSPSGMRPWEDLPSQDTGSPSR.M | Coagulation factor V precursor | BCP & HP |
| R.KYLDSTFTK.R | Coagulation factor V precursor | BCP & HP |
| R.LELFGCDIY.- | Coagulation factor V precursor | BCP & HP |
| K.LAAEFASKPWIQVDMQK.E | Coagulation factor V precursor | BCP & HP |
| R.MRPWKDPPSDLLLLK.Q | Coagulation factor V precursor | BCP & HP |
| K.QHQLGVWPLLPGSFK.T | Coagulation factor V precursor | BCP & HP |
| R.SEAYNTFSER.R | Coagulation factor V precursor | BCP & HP |
| R.SQHLDNFSNQIGK.H | Coagulation factor V precursor | BCP & HP |
| R.ETDIEDSDDIPEDTTYK.K | Coagulation factor V precursor | BCP & HP |
| K.M*DDAVAPGR.E | Coagulation factor V precursor | BCP & HP |
| R.SSSPELSEMLEYDR.S | Coagulation factor V precursor | BCP & HP |
| R.LELQGCEVNGCSTPLGMENGKIENK.Q | Coagulation factor V precursor | BCP & HP |
| K.VDSCPEEPQLR.M | Coagulation factor VIII precursor | BCP & HP |
| K.NSLNSGQGPSPK.Q | Coagulation factor VIII precursor | BCP & HP |
| K.ASEGAEYDDQTSQR.E | Coagulation factor VIII precursor | BCP & HP |
| K.HLKDFPILPGEIFK.Y | Coagulation factor VIII precursor | BCP & HP |
| K.YKDGDQCETSPCQNQGK.C | Coagulation factor X precursor | BCP & HP |
| R.DWAESTLM*TQK.T | Coagulation factor X precursor | BCP & HP |
| R.VGDRNTEQEEGGEAVHEVEVVIK.H | Coagulation factor X precursor | BCP & HP |
| R.NTEQEEGGEAVHEVEVVIK.H | Coagulation factor X precursor | BCP & HP |
| R.MNVAPACLPERDWAESTLMTQK.T | Coagulation factor X precursor | BCP & HP |
| R.MNVAPACLPERDWAESTLM*TQK.T | Coagulation factor X precursor | BCP & HP |
| R.M*NVAPACLPERDWAESTLMTQK.T | Coagulation factor X precursor | BCP & HP |
| R.KLCSLDNGDCDQFCHEEQNSVVCSCAR.G | Coagulation factor X precursor | BCP & HP |
| R.GYTLADNGK.A | Coagulation factor X precursor | BCP & HP |
| R.FTKETYDFDIAVLR.L | Coagulation factor X precursor | BCP & HP |
| K.QEDACQGDSGGPHVTR.F | Coagulation factor X precursor | BCP & HP |
| R.DWAESTLMTQK.T | Coagulation factor X precursor | BCP & HP |
| K.ACIPTGPYPCGK.Q | Coagulation factor X precursor | BCP & HP |
| K.TGIVSGFGR.T | Coagulation factor X precursor | BCP & HP |
| K.NCELFTR.K | Coagulation factor X precursor | BCP & HP |
| K.MLEVPYVDR.N | Coagulation factor X precursor | BCP & HP |
| K.LSSSFIITQNMFCAGYDTKQEDACQGDSGGPHVTR.F | Coagulation factor X precursor | BCP & HP |
| K.LSSSFIITQNMFCAGYDTK.Q | Coagulation factor X precursor | BCP & HP |
| K.GKYGIYTK.V | Coagulation factor X precursor | BCP & HP |
| K.ETYDFDIAVLR.L | Coagulation factor X precursor | BCP & HP |
| K.DGDQCETSPCQNQGK.C | Coagulation factor X precursor | BCP & HP |
| K.CKDGLGEYTCTCLEGFEGKNCELFTR.K | Coagulation factor X precursor | BCP & HP |
| K.CKDGLGEYTCTCLEGFEGK.N | Coagulation factor X precursor | BCP & HP |
| R.FKDTYFVTGIVSWGEGCAR.K | Coagulation factor X precursor | BCP & HP |
| R.PAPEDLTVVLGQER.R | Coagulation factor XII precursor | BCP & HP |
| R.TEQAAVAR.C | Coagulation factor XII precursor | BCP & HP |
| R.NKPGVYTDVAYYLAWIR.E | Coagulation factor XII precursor | BCP & HP |
| R.NPDNDIRPWCFVLNR.D | Coagulation factor XII precursor | BCP & HP |
| R.NHSCEPCQTLAVR.S | Coagulation factor XII precursor | BCP & HP |
| R.WGYCLEPK.K | Coagulation factor XII precursor | BCP & HP |
| R.NGPLSCGQR.L | Coagulation factor XII precursor | BCP & HP |
| R.PGPQPWCATTPNFDQDQR.W | Coagulation factor XII precursor | BCP & HP |
| R.PSETTLCQVAGWGHQFEGAEEYASFLQEAQVPFLSLER.C | Coagulation factor XII precursor | BCP & HP |
| R.TTLSGAPCQPWASEATYR.N | Coagulation factor XII precursor | BCP & HP |
| K.AEEHTVVLTVTGEPCHFPFQYHR.Q | Coagulation factor XII precursor | BCP & HP |
| R.RLTLQGIISWGSGCGDR.N | Coagulation factor XII precursor | BCP & HP |
| R.CSAPDVHGSSILPGMLCAGFLEGGTDACQGDSGGPLVCEDQAAER.R | Coagulation factor XII precursor | BCP & HP |
| R.NWGLGGHAFCR.N | Coagulation factor XII precursor | BCP & HP |
| R.LCHCPVGYTGPFCDVDTK.A | Coagulation factor XII precursor | BCP & HP |
| R.CSAPDVHGSSILPGM*LCAGFLEGGTDACQGDSGGPLVCEDQAAER.R | Coagulation factor XII precursor | BCP & HP |
| R.CLEVEGHR.L | Coagulation factor XII precursor | BCP & HP |
| Q.PWASEATYR.N | Coagulation factor XII precursor | BCP & HP |
| K.REQPPSLTR.N | Coagulation factor XII precursor | BCP & HP |
| K.NEIWYR.T | Coagulation factor XII precursor | BCP & HP |
| K.GRPGPQPWCATTPNFDQDQR.W | Coagulation factor XII precursor | BCP & HP |
| K.CFEPQLLR.F | Coagulation factor XII precursor | BCP & HP |
| R.EQPPSLTRNGPLSCGQR.L | Coagulation factor XII precursor | BCP & HP |
| R.TNPCLHGGR.C | Coagulation factor XII precursor | BCP & HP |
| R.LHEAFSPVSYQHDLALLR.L | Coagulation factor XII precursor | BCP & HP |
| R.LQEDADGSCALLSPYVQPVCLPSGAAR.P | Coagulation factor XII precursor | BCP & HP |
| R.VVGGLVALR.G | Coagulation factor XII precursor | BCP & HP |
| R.LTLQGIISWGSGCGDR.N | Coagulation factor XII precursor | BCP & HP |
| K.EKCFEPQLLR.F | Coagulation factor XII precursor | BCP & HP |
| K.KPLNTEGVM*K.S | Coagulation factor XIII A chain precursor | BCP & HP |
| R.VGSAM*VNAK.D | Coagulation factor XIII A chain precursor | BCP & HP |
| R.VGSAMVNAK.D | Coagulation factor XIII A chain precursor | BCP & HP |
| K.QKSTVLTIPEIIIK.V | Coagulation factor XIII A chain precursor | BCP & HP |
| K.QIGGDGMMDITDTYK.F | Coagulation factor XIII A chain precursor | BCP & HP |
| K.QIGGDGM*M*DITDTYK.F | Coagulation factor XIII A chain precursor | BCP & HP |
| R.VEYVIGRYPQENK.G | Coagulation factor XIII A chain precursor | BCP & HP |
| K.LIASM*SSDSLR.H | Coagulation factor XIII A chain precursor | BCP & HP |
| K.KDGTHVVENVDATHIGK.L | Coagulation factor XIII A chain precursor | BCP & HP |
| K.KETFDVTLEPLSFKK.E | Coagulation factor XIII A chain precursor | BCP & HP |
| K.GTYIPVPIVSELQSGK.W | Coagulation factor XIII A chain precursor | BCP & HP |
| K.FQEGQEEERLALETALMYGAK.K | Coagulation factor XIII A chain precursor | BCP & HP |
| K.DGTHVVENVDATHIGK.L | Coagulation factor XIII A chain precursor | BCP & HP |
| K.STVLTIPEIIIK.V | Coagulation factor XIII A chain precursor | BCP & HP |
| K.LIASMSSDSLR.H | Coagulation factor XIII A chain precursor | BCP & HP |
| R.GVNLQEFLNVTSVHLFK.E | Coagulation factor XIII A chain precursor | BCP & HP |
| R.SWSYGQFEDGILDTCLYVMDR.A | Coagulation factor XIII A chain precursor | BCP & HP |
| R.CGPASVQAIK.H | Coagulation factor XIII A chain precursor | BCP & HP |
| R.EIRPNSTVQWEEVCRPWVSGHR.K | Coagulation factor XIII A chain precursor | BCP & HP |
| R.AVPPNNSNAAEDDLPTVELQGVVPR.G | Coagulation factor XIII A chain precursor | BCP & HP |
| R.GNPIKVSR.V | Coagulation factor XIII A chain precursor | BCP & HP |
| R.HVYGELDVQIQR.R | Coagulation factor XIII A chain precursor | BCP & HP |
| R.INETRDVLAK.Q | Coagulation factor XIII A chain precursor | BCP & HP |
| R.IVTNYFSAHDNDANLQMDIFLEEDGNVNSK.L | Coagulation factor XIII A chain precursor | BCP & HP |
| R.KLIASM*SSDSLR.H | Coagulation factor XIII A chain precursor | BCP & HP |
| R.NVWVHLDGPGVTRPMK.K | Coagulation factor XIII A chain precursor | BCP & HP |
| R.M*YVAVWTPYGVLR.T | Coagulation factor XIII A chain precursor | BCP & HP |
| R.AQM*DLSGR.G | Coagulation factor XIII A chain precursor | BCP & HP |
| R.MYVAVWTPYGVLR.T | Coagulation factor XIII A chain precursor | BCP & HP |
| R.KLIASMSSDSLR.H | Coagulation factor XIII A chain precursor | BCP & HP |
| R.RAVPPNNSNAAEDDLPTVELQGVVPR.G | Coagulation factor XIII A chain precursor | BCP & HP |
| A.EEKPCGFPHVENGR.I | Coagulation factor XIII B chain precursor | BCP & HP |
| K.QGYDLSPLTPLSELSVQCNR.G | Coagulation factor XIII B chain precursor | BCP & HP |
| K.KTEEVECLTYGWSLTPK.C | Coagulation factor XIII B chain precursor | BCP & HP |
| K.KCTKPDLSNGYISDVK.L | Coagulation factor XIII B chain precursor | BCP & HP |
| K.IYYNGDKVTYACK.S | Coagulation factor XIII B chain precursor | BCP & HP |
| K.IQTHSTTYR.H | Coagulation factor XIII B chain precursor | BCP & HP |
| K.GMCTSPPLIK.H | Coagulation factor XIII B chain precursor | BCP & HP |
| K.DKVQYECATGYYTAGGKK.T | Coagulation factor XIII B chain precursor | BCP & HP |
| K.CTKPDLSNGYISDVK.L | Coagulation factor XIII B chain precursor | BCP & HP |
| K.TEEVECLTYGWSLTPK.C | Coagulation factor XIII B chain precursor | BCP & HP |
| K.QGYDLSPLTPLSELSVQCNRGEVKYPLCTR.K | Coagulation factor XIII B chain precursor | BCP & HP |
| K.DKVQYECATGYYTAGGK.K | Coagulation factor XIII B chain precursor | BCP & HP |
| R.QSTLSYQEPLRT.- | Coagulation factor XIII B chain precursor | BCP & HP |
| R.EAYCLDGMWTTPPLCLEPCTLSFTEMEK.N | Coagulation factor XIII B chain precursor | BCP & HP |
| R.CPPPPLPINSK.I | Coagulation factor XIII B chain precursor | BCP & HP |
| R.CNEYYLLR.G | Coagulation factor XIII B chain precursor | BCP & HP |
| R.CFDHHFLEGSR.E | Coagulation factor XIII B chain precursor | BCP & HP |
| R.CEDGKWTEPPKCIEGQEK.V | Coagulation factor XIII B chain precursor | BCP & HP |
| R.CEDGKWTEPPK.C | Coagulation factor XIII B chain precursor | BCP & HP |
| K.WTEPPKCIEGQEK.V | Coagulation factor XIII B chain precursor | BCP & HP |
| K.SFYFPMSIDKK.L | Coagulation factor XIII B chain precursor | BCP & HP |
| R.GDTYPAELYITGSILR.M | Coagulation factor XIII B chain precursor | BCP & HP |
| K.SFYFPMSIDK.K | Coagulation factor XIII B chain precursor | BCP & HP |
| R.GEVKYPLCTR.K | Coagulation factor XIII B chain precursor | BCP & HP |
| R.IAQYYYTFK.S | Coagulation factor XIII B chain precursor | BCP & HP |
| R.EAYCLDGMWTTPPLCLEPCTLSFTEMEKNNLLLK.W | Coagulation factor XIII B chain precursor | BCP & HP |
| R.NRCPPPPLPINSK.I | Coagulation factor XIII B chain precursor | BCP & HP |
| K.WSSPPVCLEPCTVNVDYMNR.N | Coagulation factor XIII B chain precursor | BCP & HP |
| R.YGCASGYK.T | Coagulation factor XIII B chain precursor | BCP & HP |
| K.WSSPPVCLEPCTVNVDYM*NR.N | Coagulation factor XIII B chain precursor | BCP & HP |
| K.VQYECATGYYTAGGKK.T | Coagulation factor XIII B chain precursor | BCP & HP |
| K.VQYECATGYYTAGGK.K | Coagulation factor XIII B chain precursor | BCP & HP |
| K.VLHGDLIDFVCK.Q | Coagulation factor XIII B chain precursor | BCP & HP |
| K.VKDKVQYECATGYYTAGGK.K | Coagulation factor XIII B chain precursor | BCP & HP |
| K.VACEEPPFIENGAANLHSK.I | Coagulation factor XIII B chain precursor | BCP & HP |
| K.TTGGKDEEVVQCLSDGWSSQPTCRK.E | Coagulation factor XIII B chain precursor | BCP & HP |
| K.TTGGKDEEVVQCLSDGWSSQPTCR.K | Coagulation factor XIII B chain precursor | BCP & HP |
| K.SGYLLHGSNEITCNR.G | Coagulation factor XIII B chain precursor | BCP & HP |
| R.LIENGYFHPVK.Q | Coagulation factor XIII B chain precursor | BCP & HP |
| R.YALYDATYETK.E | Cofilin-1 | BCP & HP |
| K.M*LPDKDCR.Y | Cofilin-1 | BCP & HP |
| K.KEDLVFIFWAPESAPLK.S | Cofilin-1 | BCP & HP |
| K.MLPDKDCR.Y | Cofilin-1 | BCP & HP |
| K.LTGIKHELQANCYEEVKDR.C | Cofilin-1 | BCP & HP |
| R.ETVDENGR.L | Coiled-coil domain-containing protein 13 | BCP & HP |
| R.DRLTEFVTVLQK.R | Coiled-coil domain-containing protein 13 | BCP & HP |
| R.VAVVQYSGTGQQRPER.A | Collagen alpha-1(VI) chain precursor | BCP & HP |
| R.DAEEAISQTIDTIVDM*IK.N | Collagen alpha-1(VI) chain precursor | BCP & HP |
| K.TAEYDVAYGESHLFR.V | Collagen alpha-1(VI) chain precursor | BCP & HP |
| R.LLLFSDGNSQGATPAAIEK.A | Collagen alpha-1(VI) chain precursor | BCP & HP |
| R.DAEEAISQTIDTIVDMIK.N | Collagen alpha-1(VI) chain precursor | BCP & HP |
| K.KGHIYQGSEADSVFSGFLIFPSA.- | Complement C1q subcomponent subunit A precursor | BCP & HP |
| K.DQPRPAFSAIR.R | Complement C1q subcomponent subunit A precursor | BCP & HP |
| R.SLGFCDTTNK.G | Complement C1q subcomponent subunit A precursor | BCP & HP |
| K.DQPRPAFSAIRR.N | Complement C1q subcomponent subunit A precursor | BCP & HP |
| K.GHIYQGSEADSVFSGFLIFPSA.- | Complement C1q subcomponent subunit A precursor | BCP & HP |
| K.GLFQVVSGGM*VLQLQQGDQVWVEKDPK.K | Complement C1q subcomponent subunit A precursor | BCP & HP |
| K.GLFQVVSGGMVLQLQQGDQVWVEKDPK.K | Complement C1q subcomponent subunit A precursor | BCP & HP |
| R.RSLGFCDTTNK.G | Complement C1q subcomponent subunit A precursor | BCP & HP |
| K.FQSVFTVTR.Q | Complement C1q subcomponent subunit C precursor | BCP & HP |
| K.NGPM*GPPGM*PGVPGPM*GIPGEPGEEGR.Y | Complement C1q subcomponent subunit C precursor | BCP & HP |
| K.TNQVNSGGVLLR.L | Complement C1q subcomponent subunit C precursor | BCP & HP |
| K.QKFQSVFTVTR.Q | Complement C1q subcomponent subunit C precursor | BCP & HP |
| K.VVTFCGHTSK.T | Complement C1q subcomponent subunit C precursor | BCP & HP |
| R.FNAVLTNPQGDYDTSTGK.F | Complement C1q subcomponent subunit C precursor | BCP & HP |
| R.QTHQPPAPNSLIR.F | Complement C1q subcomponent subunit C precursor | BCP & HP |
| R.FNAVLTNPQGDYDTSTGKFTCK.V | Complement C1q subcomponent subunit C precursor | BCP & HP |
| R.CLPVCGKPVNPVEQR.Q | Complement C1r subcomponent precursor | BCP & HP |
| R.ESEQGVYTCTAQGIWKNEQKGEK.I | Complement C1r subcomponent precursor | BCP & HP |
| K.QDACQGDSGGVFAVRDPNTDR.W | Complement C1r subcomponent precursor | BCP & HP |
| K.QGYQLIEGNQVLHSFTAVCQDDGTWHR.A | Complement C1r subcomponent precursor | BCP & HP |
| K.QDACQGDSGGVFAVR.D | Complement C1r subcomponent precursor | BCP & HP |
| K.QRPPDLDTSSNAVDLLFFTDESGDSR.G | Complement C1r subcomponent precursor | BCP & HP |
| R.FCGQLGSPLGNPPGK.K | Complement C1r subcomponent precursor | BCP & HP |
| K.VLNYVDWIKK.E | Complement C1r subcomponent precursor | BCP & HP |
| E.PSEGCFYDYVK.I | Complement C1r subcomponent precursor | BCP & HP |
| K.FLEPFDIDDHQQVHCPYDQLQIYANGK.N | Complement C1r subcomponent precursor | BCP & HP |
| R.HSCQAECSSELYTEASGYISSLEYPR.S | Complement C1r subcomponent precursor | BCP & HP |
| K.GFLAYYQAVDLDECASR.S | Complement C1r subcomponent precursor | BCP & HP |
| K.LFGEVTSPLFPKPYPNNFETTTVITVPTGYR.V | Complement C1r subcomponent precursor | BCP & HP |
| K.LVFQQFDLEPSEGCFYDYVK.I | Complement C1r subcomponent precursor | BCP & HP |
| K.M*GNFPWQVFTNIHGR.G | Complement C1r subcomponent precursor | BCP & HP |
| R.ESEQGVYTCTAQGIWK.N | Complement C1r subcomponent precursor | BCP & HP |
| K.VLNYVDWIK.K | Complement C1r subcomponent precursor | BCP & HP |
| R.ESEQGVYTCTAQGIWKNEQK.G | Complement C1r subcomponent precursor | BCP & HP |
| K.NIGEFCGK.Q | Complement C1r subcomponent precursor | BCP & HP |
| K.NRM*DVFSQNMFCAGHPSLK.Q | Complement C1r subcomponent precursor | BCP & HP |
| K.NRMDVFSQNM*FCAGHPSLK.Q | Complement C1r subcomponent precursor | BCP & HP |
| K.NRMDVFSQNMFCAGHPSLK.Q | Complement C1r subcomponent precursor | BCP & HP |
| K.NRMDVFSQNMFCAGHPSLKQDACQGDSGGVFAVR.D | Complement C1r subcomponent precursor | BCP & HP |
| K.VLNYVDWIKKEMEEED.- | Complement C1r subcomponent precursor | BCP & HP |
| K.PYPNNFETTTVITVPTGYR.V | Complement C1r subcomponent precursor | BCP & HP |
| R.DYFIATCK.Q | Complement C1r subcomponent precursor | BCP & HP |
| K.MGNFPWQVFTNIHGR.G | Complement C1r subcomponent precursor | BCP & HP |
| R.WVATGIVSWGIGCSR.G | Complement C1r subcomponent precursor | BCP & HP |
| R.GGGALLGDRWILTAAHTLYPK.E | Complement C1r subcomponent precursor | BCP & HP |
| R.FCGQLGSPLGNPPGKK.E | Complement C1r subcomponent precursor | BCP & HP |
| R.YTTTMGVNTYK.A | Complement C1r subcomponent precursor | BCP & HP |
| R.YTTTM*GVNTYK.A | Complement C1r subcomponent precursor | BCP & HP |
| R.YTTEIIK.C | Complement C1r subcomponent precursor | BCP & HP |
| R.WILTAAHTLYPK.E | Complement C1r subcomponent precursor | BCP & HP |
| R.VSVHPDYR.Q | Complement C1r subcomponent precursor | BCP & HP |
| R.VKLVFQQFDLEPSEGCFYDYVK.I | Complement C1r subcomponent precursor | BCP & HP |
| R.MDVFSQNMFCAGHPSLKQDACQGDSGGVFAVR.D | Complement C1r subcomponent precursor | BCP & HP |
| R.MDVFSQNMFCAGHPSLK.Q | Complement C1r subcomponent precursor | BCP & HP |
| K.TLDEFTIIQNLQPQYQFR.D | Complement C1r subcomponent precursor | BCP & HP |
| R.FCGQLGSPLGNPPGKKEFMSQGNK.M | Complement C1r subcomponent precursor | BCP & HP |
| R.YTTEIIKCPQPK.T | Complement C1r subcomponent precursor | BCP & HP |
| R.MDVFSQNM*FCAGHPSLK.Q | Complement C1r subcomponent precursor | BCP & HP |
| R.GGGALLGDR.W | Complement C1r subcomponent precursor | BCP & HP |
| R.FVRLPVANPQACENWLR.G | Complement C1r subcomponent precursor | BCP & HP |
| R.GYGFYTK.V | Complement C1r subcomponent precursor | BCP & HP |
| R.IQYYCHEPYYK.M | Complement C1r subcomponent precursor | BCP & HP |
| R.LPVANPQACENWLR.G | Complement C1r subcomponent precursor | BCP & HP |
| R.M*DVFSQNMFCAGHPSLK.Q | Complement C1r subcomponent precursor | BCP & HP |
| K.RQRPEVFSDNMFCVGDETQR.H | Complement C1r-like protein | BCP & HP |
| K.GQESSTDIKAPEGFAVR.L | Complement C1r-like protein | BCP & HP |
| R.GGGALLGDRWILTAAHTVYPK.D | Complement C1r-like protein | BCP & HP |
| R.GSEAINAPGDNPAK.V | Complement C1r-like protein | BCP & HP |
| R.GGGALLGDR.W | Complement C1r-like protein | BCP & HP |
| R.QRPEVFSDNM*FCVGDETQR.H | Complement C1r-like protein | BCP & HP |
| R.QRPEVFSDNMFCVGDETQR.H | Complement C1r-like protein | BCP & HP |
| R.VVVHPDYR.Q | Complement C1r-like protein | BCP & HP |
| G.SVLLAQELPQQLTSPGYPEPYGKGQESSTDIKAPEGFAVR.L | Complement C1r-like protein | BCP & HP |
| K.VQNHCQEPYYQAAAAGALTCATPGTWK.D | Complement C1r-like protein | BCP & HP |
| K.LGNFPWQAFTSIHGR.G | Complement C1r-like protein | BCP & HP |
| R.EACNAWLQK.R | Complement C1r-like protein | BCP & HP |
| R.WILTAAHTVYPK.D | Complement C1r-like protein | BCP & HP |
| K.YSRLPVAPR.E | Complement C1r-like protein | BCP & HP |
| K.VLSYVDWIK.G | Complement C1r-like protein | BCP & HP |
| G.SVLLAQELPQQLTSPGYPEPYGK.G | Complement C1r-like protein | BCP & HP |
| K.LKCQPVDCGIPESIENGKVEDPESTLFGSVIR.Y | Complement C1s subcomponent precursor | BCP & HP |
| K.M*GPTVSPICLPGTSSDYNLM*DGDLGLISGWGR.T | Complement C1s subcomponent precursor | BCP & HP |
| K.M*GPTVSPICLPGTSSDYNLMDGDLGLISGWGR.T | Complement C1s subcomponent precursor | BCP & HP |
| K.M*LTPEHVFIHPGWK.L | Complement C1s subcomponent precursor | BCP & HP |
| K.GMDSCKGDSGGAFAVQDPNDKTK.F | Complement C1s subcomponent precursor | BCP & HP |
| K.MGPTVSPICLPGTSSDYNLMDGDLGLISGWGR.T | Complement C1s subcomponent precursor | BCP & HP |
| K.MLTPEHVFIHPGWK.L | Complement C1s subcomponent precursor | BCP & HP |
| K.MGPTVSPICLPGTSSDYNLM*DGDLGLISGWGR.T | Complement C1s subcomponent precursor | BCP & HP |
| K.GMDSCKGDSGGAFAVQDPNDK.T | Complement C1s subcomponent precursor | BCP & HP |
| K.GM*DSCKGDSGGAFAVQDPNDKTK.F | Complement C1s subcomponent precursor | BCP & HP |
| K.GDSGGAFAVQDPNDK.T | Complement C1s subcomponent precursor | BCP & HP |
| K.FYAAGLVSWGPQCGTYGLYTR.V | Complement C1s subcomponent precursor | BCP & HP |
| K.EVKVEKPTADAEAYVFTPNMICAGGEK.G | Complement C1s subcomponent precursor | BCP & HP |
| K.EVKVEKPTADAEAYVFTPNM*ICAGGEK.G | Complement C1s subcomponent precursor | BCP & HP |
| K.EDTPNSVWEPAK.A | Complement C1s subcomponent precursor | BCP & HP |
| K.NYVDWIMK.T | Complement C1s subcomponent precursor | BCP & HP |
| R.QFGPYCGHGFPGPLNIETK.S | Complement C1s subcomponent precursor | BCP & HP |
| K.GDSGGAFAVQDPNDKTK.F | Complement C1s subcomponent precursor | BCP & HP |
| R.EPTM*YVGSTSVQTSR.L | Complement C1s subcomponent precursor | BCP & HP |
| R.VKNYVDWIM*K.T | Complement C1s subcomponent precursor | BCP & HP |
| R.VGATSFYSTCQSNGKWSNSK.L | Complement C1s subcomponent precursor | BCP & HP |
| R.VGATSFYSTCQSNGK.W | Complement C1s subcomponent precursor | BCP & HP |
| R.TNFDNDIALVR.L | Complement C1s subcomponent precursor | BCP & HP |
| R.SSNNPHSPIVEEFQVPYNKLQVIFK.S | Complement C1s subcomponent precursor | BCP & HP |
| R.SSNNPHSPIVEEFQVPYNK.L | Complement C1s subcomponent precursor | BCP & HP |
| R.REDFDVEAADSAGNCLDSLVFVAGDR.Q | Complement C1s subcomponent precursor | BCP & HP |
| A.EPTM*YGEILSPNYPQAYPSEVEK.S | Complement C1s subcomponent precursor | BCP & HP |
| R.EPTMYVGSTSVQTSR.L | Complement C1s subcomponent precursor | BCP & HP |
| K.CVPVCGVPREPFEEKQR.I | Complement C1s subcomponent precursor | BCP & HP |
| K.SDFSNEER.F | Complement C1s subcomponent precursor | BCP & HP |
| R.EDFDVEAADSAGNCLDSLVFVAGDR.Q | Complement C1s subcomponent precursor | BCP & HP |
| R.DVVQITCLDGFEVVEGR.V | Complement C1s subcomponent precursor | BCP & HP |
| R.CEYQIR.L | Complement C1s subcomponent precursor | BCP & HP |
| K.VEKPTADAEAYVFTPNMICAGGEK.G | Complement C1s subcomponent precursor | BCP & HP |
| K.VEKPTADAEAYVFTPNM*ICAGGEK.G | Complement C1s subcomponent precursor | BCP & HP |
| K.TMQENSTPRED.- | Complement C1s subcomponent precursor | BCP & HP |
| K.TM*QENSTPRED.- | Complement C1s subcomponent precursor | BCP & HP |
| K.SNALDIIFQTDLTGQKK.G | Complement C1s subcomponent precursor | BCP & HP |
| K.SNALDIIFQTDLTGQK.K | Complement C1s subcomponent precursor | BCP & HP |
| R.IIGGSDADIK.N | Complement C1s subcomponent precursor | BCP & HP |
| R.YHGDPM*PCPKEDTPNSVWEPAK.A | Complement C1s subcomponent precursor | BCP & HP |
| K.CVPVCGVPREPFEEK.Q | Complement C1s subcomponent precursor | BCP & HP |
| C.LPGTSSDYNLMDGDLGLISGWGR.T | Complement C1s subcomponent precursor | BCP & HP |
| R.YHGDPMPCPK.E | Complement C1s subcomponent precursor | BCP & HP |
| R.YHGDPM*PCPK.E | Complement C1s subcomponent precursor | BCP & HP |
| R.VKNYVDWIMK.T | Complement C1s subcomponent precursor | BCP & HP |
| K.CQPVDCGIPESIENGKVEDPESTLFGSVIR.Y | Complement C1s subcomponent precursor | BCP & HP |
| I.PESIENGKVEDPESTLFGSVIR.Y | Complement C1s subcomponent precursor | BCP & HP |
| K.CKEVKVEKPTADAEAYVFTPNMICAGGEK.G | Complement C1s subcomponent precursor | BCP & HP |
| A.EPTMYGEILSPNYPQAYPSEVEK.S | Complement C1s subcomponent precursor | BCP & HP |
| R.YHGDPMPCPKEDTPNSVWEPAK.A | Complement C1s subcomponent precursor | BCP & HP |
| K.CVPVCGVPR.E | Complement C1s subcomponent precursor | BCP & HP |
| P.APVSFENGIYTPR.L | Complement C2 precursor (Fragment) | BCP & HP |
| K.VLMSVLNDNSR.D | Complement C2 precursor (Fragment) | BCP & HP |
| K.LDVDWRELNELGSK.K | Complement C2 precursor (Fragment) | BCP & HP |
| K.VLM*SVLNDNSR.D | Complement C2 precursor (Fragment) | BCP & HP |
| K.AVISPGFDVFAKK.N | Complement C2 precursor (Fragment) | BCP & HP |
| K.ESASLM*VDR.I | Complement C2 precursor (Fragment) | BCP & HP |
| K.KNQGILEFYGDDIALLK.L | Complement C2 precursor (Fragment) | BCP & HP |
| R.FFQVGLVSWGLYNPCLGSADK.N | Complement C2 precursor (Fragment) | BCP & HP |
| K.MSTHARPICLPCTMEANLALR.R | Complement C2 precursor (Fragment) | BCP & HP |
| K.ALHQVFEHMLDVSK.L | Complement C2 precursor (Fragment) | BCP & HP |
| K.ALHQVFEHM*LDVSK.L | Complement C2 precursor (Fragment) | BCP & HP |
| K.M*GVEWTSCAEVVSQEK.T | Complement C2 precursor (Fragment) | BCP & HP |
| K.M*STHARPICLPCTM*EANLALR.R | Complement C2 precursor (Fragment) | BCP & HP |
| K.M*STHARPICLPCTMEANLALR.R | Complement C2 precursor (Fragment) | BCP & HP |
| K.VPPPRDFHINLFR.M | Complement C2 precursor (Fragment) | BCP & HP |
| K.MSTHARPICLPCTM*EANLALR.R | Complement C2 precursor (Fragment) | BCP & HP |
| K.AVISPGFDVFAK.K | Complement C2 precursor (Fragment) | BCP & HP |
| K.NQGILEFYGDDIALLK.L | Complement C2 precursor (Fragment) | BCP & HP |
| K.RNDYLDIYAIGVGK.L | Complement C2 precursor (Fragment) | BCP & HP |
| K.SQWGKEFLIEK.A | Complement C2 precursor (Fragment) | BCP & HP |
| K.SSGQWQTPGATR.S | Complement C2 precursor (Fragment) | BCP & HP |
| K.TAVDHIR.E | Complement C2 precursor (Fragment) | BCP & HP |
| K.TAVDHIREILNINQK.R | Complement C2 precursor (Fragment) | BCP & HP |
| K.MGVEWTSCAEVVSQEK.T | Complement C2 precursor (Fragment) | BCP & HP |
| R.QCRPNGM*WDGETAVCDNGAGHCPNPGISLGAVR.T | Complement C2 precursor (Fragment) | BCP & HP |
| R.HAIILLTDGKSNMGGSPK.T | Complement C2 precursor (Fragment) | BCP & HP |
| R.LLGM*ETM*AWQEIR.H | Complement C2 precursor (Fragment) | BCP & HP |
| R.LLGM*ETMAWQEIR.H | Complement C2 precursor (Fragment) | BCP & HP |
| R.LLGMETM*AWQEIR.H | Complement C2 precursor (Fragment) | BCP & HP |
| R.LLGMETMAWQEIR.H | Complement C2 precursor (Fragment) | BCP & HP |
| R.HAIILLTDGKSNM*GGSPK.T | Complement C2 precursor (Fragment) | BCP & HP |
| R.PICLPCTM*EANLALR.R | Complement C2 precursor (Fragment) | BCP & HP |
| R.PICLPCTMEANLALR.R | Complement C2 precursor (Fragment) | BCP & HP |
| R.EVVTDQFLCSGTQEDESPCK.G | Complement C2 precursor (Fragment) | BCP & HP |
| R.TPWHVTIKPK.S | Complement C2 precursor (Fragment) | BCP & HP |
| R.QPYSYDFPEDVAPALGTSFSHMLGATNPTQK.T | Complement C2 precursor (Fragment) | BCP & HP |
| R.QPYSYDFPEDVAPALGTSFSHM*LGATNPTQK.T | Complement C2 precursor (Fragment) | BCP & HP |
| R.QHLGDVLNFLPL.- | Complement C2 precursor (Fragment) | BCP & HP |
| R.QCRPNGMWDGETAVCDNGAGHCPNPGISLGAVR.T | Complement C2 precursor (Fragment) | BCP & HP |
| R.NDYLDIYAIGVGK.L | Complement C2 precursor (Fragment) | BCP & HP |
| R.EILNINQK.R | Complement C2 precursor (Fragment) | BCP & HP |
| R.CPAPVSFENGIYTPR.L | Complement C2 precursor (Fragment) | BCP & HP |
| R.DGNDHSLWR.V | Complement C2 precursor (Fragment) | BCP & HP |
| R.DFHINLFR.M | Complement C2 precursor (Fragment) | BCP & HP |
| R.CSSNLVLTGSSERECQGNGVWSGTEPICR.Q | Complement C2 precursor (Fragment) | BCP & HP |
| R.DHENELLNK.Q | Complement C2 precursor (Fragment) | BCP & HP |
| R.HAIILLTDGK.S | Complement C2 precursor (Fragment) | BCP & HP |
| R.CSSNLVLTGSSER.E | Complement C2 precursor (Fragment) | BCP & HP |
| R.DM*TEVISSLENANYK.D | Complement C2 precursor (Fragment) | BCP & HP |
| R.EILNINQKR.N | Complement C2 precursor (Fragment) | BCP & HP |
| R.ELNELGSKKDGER.H | Complement C2 precursor (Fragment) | BCP & HP |
| R.EVVTDQFLCSGTQEDESPCKGESGGAVFLER.R | Complement C2 precursor (Fragment) | BCP & HP |
| R.FFQVGLVSWGLYNPCLGSADKNSR.K | Complement C2 precursor (Fragment) | BCP & HP |
| R.GALISDQWVLTAAHCFR.D | Complement C2 precursor (Fragment) | BCP & HP |
| R.HAFILQDTK.A | Complement C2 precursor (Fragment) | BCP & HP |
| R.ECQGNGVWSGTEPICR.Q | Complement C2 precursor (Fragment) | BCP & HP |
| R.SGIPIVTSPYQIHFTK.T | Complement C3 precursor (Fragment) | BCP & HP |
| R.VVLVAVDK.G | Complement C3 precursor (Fragment) | BCP & HP |
| R.VPVAVQGEDTVQSLTQGDGVAK.L | Complement C3 precursor (Fragment) | BCP & HP |
| R.VELLHNPAFCSLATTK.R | Complement C3 precursor (Fragment) | BCP & HP |
| R.TVMVNIENPEGIPVKQDSLSSQNQLGVLPLSWDIPELVNMGQWK.I | Complement C3 precursor (Fragment) | BCP & HP |
| R.ILLQGTPVAQM*TEDAVDAER.L | Complement C3 precursor (Fragment) | BCP & HP |
| R.TMQALPYSTVGNSNNYLHLSVLR.T | Complement C3 precursor (Fragment) | BCP & HP |
| R.TKKQELSEAEQATR.T | Complement C3 precursor (Fragment) | BCP & HP |
| R.TELRPGETLNVNFLLR.M | Complement C3 precursor (Fragment) | BCP & HP |
| R.ILLQGTPVAQMTEDAVDAER.L | Complement C3 precursor (Fragment) | BCP & HP |
| R.SYTVAIAGYALAQMGR.L | Complement C3 precursor (Fragment) | BCP & HP |
| R.CAEENCFIQK.S | Complement C3 precursor (Fragment) | BCP & HP |
| R.SNLDEDIIAEENIVSR.S | Complement C3 precursor (Fragment) | BCP & HP |
| R.YYTYLIMNK.G | Complement C3 precursor (Fragment) | BCP & HP |
| R.SEFPESWLWNVEDLKEPPKNGISTK.L | Complement C3 precursor (Fragment) | BCP & HP |
| R.SEETKENEGFTVTAEGK.G | Complement C3 precursor (Fragment) | BCP & HP |
| R.QVREPGQDLVVLPLSITTDFIPSFR.L | Complement C3 precursor (Fragment) | BCP & HP |
| R.QPSSAFAAFVKR.A | Complement C3 precursor (Fragment) | BCP & HP |
| R.QPSSAFAAFVK.R | Complement C3 precursor (Fragment) | BCP & HP |
| R.NNNEKDMALTAFVLISLQEAKDICEEQVNSLPGSITK.A | Complement C3 precursor (Fragment) | BCP & HP |
| R.LVAYYTLIGASGQR.E | Complement C3 precursor (Fragment) | BCP & HP |
| R.IPIEDGSGEVVLSR.K | Complement C3 precursor (Fragment) | BCP & HP |
| R.LPYSVVRNEQVEIR.A | Complement C3 precursor (Fragment) | BCP & HP |
| R.LESEETMVLEAHDAQGDVPVTVTVHDFPGKK.L | Complement C3 precursor (Fragment) | BCP & HP |
| R.LESEETMVLEAHDAQGDVPVTVTVHDFPGK.K | Complement C3 precursor (Fragment) | BCP & HP |
| R.SVQLTEKR.M | Complement C3 precursor (Fragment) | BCP & HP |
| K.AAVYHHFISDGVR.K | Complement C3 precursor (Fragment) | BCP & HP |
| K.EYVLPSFEVIVEPTEK.F | Complement C3 precursor (Fragment) | BCP & HP |
| K.EDIPPADLSDQVPDTESETR.I | Complement C3 precursor (Fragment) | BCP & HP |
| K.DTWVEHWPEEDECQDEENQK.Q | Complement C3 precursor (Fragment) | BCP & HP |
| R.FISLGEACKK.V | Complement C3 precursor (Fragment) | BCP & HP |
| K.DSCVGSLVVK.S | Complement C3 precursor (Fragment) | BCP & HP |
| K.DICEEQVNSLPGSITK.A | Complement C3 precursor (Fragment) | BCP & HP |
| K.DAPDHQELNLDVSLQLPSR.S | Complement C3 precursor (Fragment) | BCP & HP |
| K.AKDQLTCNKFDLK.V | Complement C3 precursor (Fragment) | BCP & HP |
| K.AGDFLEANYMNLQR.S | Complement C3 precursor (Fragment) | BCP & HP |
| K.AGDFLEANYM*NLQR.S | Complement C3 precursor (Fragment) | BCP & HP |
| K.ADIGCTPGSGKDYAGVFSDAGLTFTSSSGQQTAQR.A | Complement C3 precursor (Fragment) | BCP & HP |
| R.YISKYELDKAFSDR.N | Complement C3 precursor (Fragment) | BCP & HP |
| K.ACEPGVDYVYK.T | Complement C3 precursor (Fragment) | BCP & HP |
| R.YYGGGYGSTQATFMVFQALAQYQK.D | Complement C3 precursor (Fragment) | BCP & HP |
| G.SPMYSIITPNILR.L | Complement C3 precursor (Fragment) | BCP & HP |
| R.EPGQDLVVLPLSITTDFIPSFR.L | Complement C3 precursor (Fragment) | BCP & HP |
| R.EVVADSVWVDVK.D | Complement C3 precursor (Fragment) | BCP & HP |
| R.HQQTVTIPPK.S | Complement C3 precursor (Fragment) | BCP & HP |
| R.KCCEDGMRENPMR.F | Complement C3 precursor (Fragment) | BCP & HP |
| R.FISLGEACK.K | Complement C3 precursor (Fragment) | BCP & HP |
| K.LCRDELCR.C | Complement C3 precursor (Fragment) | BCP & HP |
| R.IHWESASLLR.S | Complement C3 precursor (Fragment) | BCP & HP |
| R.FYHPEKEDGK.L | Complement C3 precursor (Fragment) | BCP & HP |
| R.EGVQKEDIPPADLSDQVPDTESETR.I | Complement C3 precursor (Fragment) | BCP & HP |
| R.TM*QALPYSTVGNSNNYLHLSVLR.T | Complement C3 precursor (Fragment) | BCP & HP |
| K.ADIGCTPGSGK.D | Complement C3 precursor (Fragment) | BCP & HP |
| K.FYYIYNEK.G | Complement C3 precursor (Fragment) | BCP & HP |
| K.SDDKVTLEERLDK.A | Complement C3 precursor (Fragment) | BCP & HP |
| K.RIPIEDGSGEVVLSRK.V | Complement C3 precursor (Fragment) | BCP & HP |
| K.RIPIEDGSGEVVLSR.K | Complement C3 precursor (Fragment) | BCP & HP |
| K.RAPSTWLTAYVVK.V | Complement C3 precursor (Fragment) | BCP & HP |
| R.AYYENSPQQVFSTEFEVK.E | Complement C3 precursor (Fragment) | BCP & HP |
| K.QKPDGVFQEDAPVIHQEMIGGLR.N | Complement C3 precursor (Fragment) | BCP & HP |
| R.TVMVNIENPEGIPVK.Q | Complement C3 precursor (Fragment) | BCP & HP |
| R.LDKACEPGVDYVYK.T | Complement C3 precursor (Fragment) | BCP & HP |
| K.QELSEAEQATR.T | Complement C3 precursor (Fragment) | BCP & HP |
| R.KVLLDGVQNPR.A | Complement C3 precursor (Fragment) | BCP & HP |
| K.QDSLSSQNQLGVLPLSWDIPELVNMGQWK.I | Complement C3 precursor (Fragment) | BCP & HP |
| K.NTMILEICTR.Y | Complement C3 precursor (Fragment) | BCP & HP |
| K.SGQSEDRQPVPGQQM*TLK.I | Complement C3 precursor (Fragment) | BCP & HP |
| K.LMNIFLK.D | Complement C3 precursor (Fragment) | BCP & HP |
| K.QLYNVEATSYALLALLQLK.D | Complement C3 precursor (Fragment) | BCP & HP |
| K.GLEVTITAR.F | Complement C3 precursor (Fragment) | BCP & HP |
| K.GQGTLSVVTM*YHAK.A | Complement C3 precursor (Fragment) | BCP & HP |
| K.GQGTLSVVTMYHAK.A | Complement C3 precursor (Fragment) | BCP & HP |
| K.GYTQQLAFR.Q | Complement C3 precursor (Fragment) | BCP & HP |
| K.GYTQQLAFRQPSSAFAAFVK.R | Complement C3 precursor (Fragment) | BCP & HP |
| K.HLIVTPSGCGEQNMIGMTPTVIAVHYLDETEQWEK.F | Complement C3 precursor (Fragment) | BCP & HP |
| K.IWDVVEK.A | Complement C3 precursor (Fragment) | BCP & HP |
| K.KGYTQQLAFR.Q | Complement C3 precursor (Fragment) | BCP & HP |
| K.KQELSEAEQATR.T | Complement C3 precursor (Fragment) | BCP & HP |
| K.KVEGTAFVIFGIQDGEQR.I | Complement C3 precursor (Fragment) | BCP & HP |
| K.KVFLDCCNYITELR.R | Complement C3 precursor (Fragment) | BCP & HP |
| K.KVFLDCCNYITELRR.Q | Complement C3 precursor (Fragment) | BCP & HP |
| K.LSINTHPSQKPLSITVR.T | Complement C3 precursor (Fragment) | BCP & HP |
| K.VTIKPAPETEK.R | Complement C3 precursor (Fragment) | BCP & HP |
| K.SGQSEDRQPVPGQQMTLK.I | Complement C3 precursor (Fragment) | BCP & HP |
| K.VEGTAFVIFGIQDGEQR.I | Complement C3 precursor (Fragment) | BCP & HP |
| K.VFLDCCNYITELR.R | Complement C3 precursor (Fragment) | BCP & HP |
| K.VHQYFNVELIQPGAVK.V | Complement C3 precursor (Fragment) | BCP & HP |
| K.VLLDGVQNPR.A | Complement C3 precursor (Fragment) | BCP & HP |
| K.VQLSNDFDEYIM*AIEQTIK.S | Complement C3 precursor (Fragment) | BCP & HP |
| K.VQLSNDFDEYIMAIEQTIK.S | Complement C3 precursor (Fragment) | BCP & HP |
| K.VRVELLHNPAFCSLATTK.R | Complement C3 precursor (Fragment) | BCP & HP |
| R.KCCEDGMRENPM*R.F | Complement C3 precursor (Fragment) | BCP & HP |
| K.TIYTPGSTVLYR.I | Complement C3 precursor (Fragment) | BCP & HP |
| K.TGLQEVEVK.A | Complement C3 precursor (Fragment) | BCP & HP |
| K.VTIKPAPETEKRPQDAK.N | Complement C3 precursor (Fragment) | BCP & HP |
| K.VYAYYNLEESCTR.F | Complement C3 precursor (Fragment) | BCP & HP |
| K.SSLSVPYVIVPLK.T | Complement C3 precursor (Fragment) | BCP & HP |
| R.ASHLGLAR.S | Complement C3 precursor (Fragment) | BCP & HP |
| K.VSHSEDDCLAFK.V | Complement C3 precursor (Fragment) | BCP & HP |
| K.SGSDEVQVGQQR.T | Complement C3 precursor (Fragment) | BCP & HP |
| K.YFKPGM*PFDLMVFVTNPDGSPAYR.V | Complement C3 precursor (Fragment) | BCP & HP |
| R.AELQCPQPAAR.R | Complement C3 precursor (Fragment) | BCP & HP |
| R.APSTWLTAYVVK.V | Complement C3 precursor (Fragment) | BCP & HP |
| P.IEDGSGEVVLSR.K | Complement C3 precursor (Fragment) | BCP & HP |
| K.SSLSVPYVIVPLKTGLQEVEVK.A | Complement C3 precursor (Fragment) | BCP & HP |
| K.YFKPGMPFDLMVFVTNPDGSPAYR.V | Complement C3 precursor (Fragment) | BCP & HP |
| K.YFKPGMPFDLM*VFVTNPDGSPAYR.V | Complement C3 precursor (Fragment) | BCP & HP |
| K.SLYVSATVILHSGSDMVQAER.S | Complement C3 precursor (Fragment) | BCP & HP |
| R.EPFLSCCQFAESLRK.K | Complement C4-A precursor | BCP & HP |
| R.GCGEQTM*IYLAPTLAASRYLDKTEQWSTLPPETK.D | Complement C4-A precursor | BCP & HP |
| R.GCGEQTM*IYLAPTLAASR.Y | Complement C4-A precursor | BCP & HP |
| R.FGLLDEDGKKTFFRGLESQTK.L | Complement C4-A precursor | BCP & HP |
| R.FGLLDEDGKKTFFR.G | Complement C4-A precursor | BCP & HP |
| R.EM*SGSPASGIPVK.V | Complement C4-A precursor | BCP & HP |
| R.EPFLSCCQFAESLR.K | Complement C4-A precursor | BCP & HP |
| R.FGLLDEDGK.K | Complement C4-A precursor | BCP & HP |
| R.GCGEQTMIYLAPTLAASR.Y | Complement C4-A precursor | BCP & HP |
| R.DKGQAGLQR.A | Complement C4-A precursor | BCP & HP |
| R.EMSGSPASGIPVK.V | Complement C4-A precursor | BCP & HP |
| R.FGLLDEDGKK.T | Complement C4-A precursor | BCP & HP |
| R.GHLFLQTDQPIYNPGQR.V | Complement C4-A precursor | BCP & HP |
| R.ECVGFEAVQEVPVGLVQPASATLYDYYNPERR.C | Complement C4-A precursor | BCP & HP |
| R.GLEEELQFSLGSK.I | Complement C4-A precursor | BCP & HP |
| R.ECVGFEAVQEVPVGLVQPASATLYDYYNPER.R | Complement C4-A precursor | BCP & HP |
| R.DSSTWLTAFVLK.V | Complement C4-A precursor | BCP & HP |
| R.DFALLSLQVPLKDAK.S | Complement C4-A precursor | BCP & HP |
| R.DFALLSLQVPLK.D | Complement C4-A precursor | BCP & HP |
| R.CSVFYGAPSKSR.L | Complement C4-A precursor | BCP & HP |
| R.CSVFYGAPSK.S | Complement C4-A precursor | BCP & HP |
| R.EELVYELNPLDHR.G | Complement C4-A precursor | BCP & HP |
| R.GQIVFM*NREPK.R | Complement C4-A precursor | BCP & HP |
| R.EAPKVVEEQESR.V | Complement C4-A precursor | BCP & HP |
| R.GPEVQLVAHSPWLK.D | Complement C4-A precursor | BCP & HP |
| R.NGKVGLSGMAIADVTLLSGFHALR.A | Complement C4-A precursor | BCP & HP |
| R.GRTLEIPGNSDPNM*IPDGDFNSYVR.V | Complement C4-A precursor | BCP & HP |
| R.GQIVFMNREPKR.T | Complement C4-A precursor | BCP & HP |
| R.GQIVFMNREPK.R | Complement C4-A precursor | BCP & HP |
| R.GQIVFMNR.E | Complement C4-A precursor | BCP & HP |
| R.QIRGLEEELQFSLGSK.I | Complement C4-A precursor | BCP & HP |
| R.CCQDGVTRLPMMR.S | Complement C4-A precursor | BCP & HP |
| R.MKFACYYPR.V | Complement C4-A precursor | BCP & HP |
| R.GLQDEDGYR.M | Complement C4-A precursor | BCP & HP |
| R.GLESQTK.L | Complement C4-A precursor | BCP & HP |
| R.NPSRNNVPCSPK.V | Complement C4-A precursor | BCP & HP |
| R.PVAFSVVPTAAAAVSLK.V | Complement C4-A precursor | BCP & HP |
| R.QGSFQGGFR.S | Complement C4-A precursor | BCP & HP |
| R.GPEVQLVAHSPWLKDSLSR.T | Complement C4-A precursor | BCP & HP |
| R.GQIVFM*NR.E | Complement C4-A precursor | BCP & HP |
| R.LLATLCSAEVCQCAEGKCPR.Q | Complement C4-A precursor | BCP & HP |
| R.GRTLEIPGNSDPNMIPDGDFNSYVR.V | Complement C4-A precursor | BCP & HP |
| R.GSFEFPVGDAVSK.V | Complement C4-A precursor | BCP & HP |
| R.HLVPGAPFLLQALVR.E | Complement C4-A precursor | BCP & HP |
| R.KADGSYAAWLSR.D | Complement C4-A precursor | BCP & HP |
| R.KKEVYM*PSSIFQDDFVIPDISEPGTWK.I | Complement C4-A precursor | BCP & HP |
| R.KKEVYMPSSIFQDDFVIPDISEPGTWK.I | Complement C4-A precursor | BCP & HP |
| R.NGFKSHALQLNNR.Q | Complement C4-A precursor | BCP & HP |
| R.LLATLCSAEVCQCAEGK.C | Complement C4-A precursor | BCP & HP |
| R.NGESVKLHLETDSLALVALGALDTALYAAGSK.S | Complement C4-A precursor | BCP & HP |
| R.LLLFSPSVVHLGVPLSVGVQLQDVPR.G | Complement C4-A precursor | BCP & HP |
| R.LRLEPGKEYLIM*GLDGATYDLEGHPQYLLDSNSWIEEMPSER.L | Complement C4-A precursor | BCP & HP |
| R.LRLEPGKEYLIMGLDGATYDLEGHPQYLLDSNSWIEEMPSER.L | Complement C4-A precursor | BCP & HP |
| R.LTVAAPPSGGPGFLSIERPDSRPPR.V | Complement C4-A precursor | BCP & HP |
| R.M*KFACYYPR.V | Complement C4-A precursor | BCP & HP |
| R.QRAACAQLNDFLQEYGTQGCQV.- | Complement C4-A precursor | BCP & HP |
| R.LEPGKEYLIMGLDGATYDLEGHPQYLLDSNSWIEEMPSER.L | Complement C4-A precursor | BCP & HP |
| R.YLDKTEQWSTLPPETKDHAVDLIQK.G | Complement C4-A precursor | BCP & HP |
| R.VGDTLNLNLR.A | Complement C4-A precursor | BCP & HP |
| R.VHYTVCIWR.N | Complement C4-A precursor | BCP & HP |
| R.VQQPDCREPFLSCCQFAESLR.K | Complement C4-A precursor | BCP & HP |
| R.VQQPDCREPFLSCCQFAESLRK.K | Complement C4-A precursor | BCP & HP |
| R.VTASDPLDTLGSEGALSPGGVASLLR.L | Complement C4-A precursor | BCP & HP |
| R.QGSFQGGFRSTQDTVIALDALSAYWIASHTTEER.G | Complement C4-A precursor | BCP & HP |
| R.YLDKTEQWSTLPPETK.D | Complement C4-A precursor | BCP & HP |
| R.VEASISKANSFLGEK.A | Complement C4-A precursor | BCP & HP |
| R.YRVFALDQK.M | Complement C4-A precursor | BCP & HP |
| R.YVSHFETEGPHVLLYFDSVPTSR.E | Complement C4-A precursor | BCP & HP |
| T.PGKPYILTVPGHLDEMQLDIQAR.Y | Complement C4-A precursor | BCP & HP |
| V.PGAPFLLQALVR.E | Complement C4-A precursor | BCP & HP |
| V.PGHLDEMQLDIQAR.Y | Complement C4-A precursor | BCP & HP |
| V.PVGLVQPASATLYDYYNPER.R | Complement C4-A precursor | BCP & HP |
| R.YIYGKPVQGVAYVR.F | Complement C4-A precursor | BCP & HP |
| R.TLEIPGNSDPNMIPDGDFNSYVR.V | Complement C4-A precursor | BCP & HP |
| R.RCSVFYGAPSK.S | Complement C4-A precursor | BCP & HP |
| R.RFEQLELRPVLYNYLDK.N | Complement C4-A precursor | BCP & HP |
| R.RGHLFLQTDQPIYNPGQR.V | Complement C4-A precursor | BCP & HP |
| R.SFFPENWLWR.V | Complement C4-A precursor | BCP & HP |
| R.SM*QGGLVGNDETVALTAFVTIALHHGLAVFQDEGAEPLK.Q | Complement C4-A precursor | BCP & HP |
| R.SMQGGLVGNDETVALTAFVTIALHHGLAVFQDEGAEPLK.Q | Complement C4-A precursor | BCP & HP |
| R.VFALDQK.M | Complement C4-A precursor | BCP & HP |
| R.TLEIPGNSDPNM*IPDGDFNSYVR.V | Complement C4-A precursor | BCP & HP |
| R.VEYGFQVK.V | Complement C4-A precursor | BCP & HP |
| R.TTNIQGINLLFSSR.R | Complement C4-A precursor | BCP & HP |
| R.TYNVLDM*K.N | Complement C4-A precursor | BCP & HP |
| R.TYNVLDMK.N | Complement C4-A precursor | BCP & HP |
| R.VDVQAGACEGK.L | Complement C4-A precursor | BCP & HP |
| R.VDVQAGACEGKLELSVDGAK.Q | Complement C4-A precursor | BCP & HP |
| Y.ILTVPGHLDEMQLDIQAR.Y | Complement C4-A precursor | BCP & HP |
| R.STQDTVIALDALSAYWIASHTTEER.G | Complement C4-A precursor | BCP & HP |
| K.KEVYM*PSSIFQDDFVIPDISEPGTWK.I | Complement C4-A precursor | BCP & HP |
| K.EGAIHREELVYELNPLDHR.G | Complement C4-A precursor | BCP & HP |
| K.EGAIHREELVYELNPLDHRG.R | Complement C4-A precursor | BCP & HP |
| K.EVYM*PSSIFQDDFVIPDISEPGTWK.I | Complement C4-A precursor | BCP & HP |
| K.EVYMPSSIFQDDFVIPDISEPGTWK.I | Complement C4-A precursor | BCP & HP |
| K.FACYYPR.V | Complement C4-A precursor | BCP & HP |
| K.GLCVATPVQLR.V | Complement C4-A precursor | BCP & HP |
| K.GSVFLRNPSR.N | Complement C4-A precursor | BCP & HP |
| K.ITPGKPYILTVPGHLDEM*QLDIQAR.Y | Complement C4-A precursor | BCP & HP |
| K.ITPGKPYILTVPGHLDEMQLDIQAR.Y | Complement C4-A precursor | BCP & HP |
| K.LVNGQSHISLSK.A | Complement C4-A precursor | BCP & HP |
| K.ITQVLHFTKDVK.A | Complement C4-A precursor | BCP & HP |
| K.DDPDAPLQPVTPLQLFEGR.R | Complement C4-A precursor | BCP & HP |
| K.KEVYMPSSIFQDDFVIPDISEPGTWK.I | Complement C4-A precursor | BCP & HP |
| K.KYVLPNFEVK.I | Complement C4-A precursor | BCP & HP |
| K.LELSVDGAK.Q | Complement C4-A precursor | BCP & HP |
| K.LGQYASPTAKR.C | Complement C4-A precursor | BCP & HP |
| K.LHLETDSLALVALGALDTALYAAGSK.S | Complement C4-A precursor | BCP & HP |
| K.LGQYASPTAK.R | Complement C4-A precursor | BCP & HP |
| K.LNMGITDLQGLR.L | Complement C4-A precursor | BCP & HP |
| R.CCQDGVTRLPM*M*R.S | Complement C4-A precursor | BCP & HP |
| K.LTSLSDRYVSHFETEGPHVLLYFDSVPTSR.E | Complement C4-A precursor | BCP & HP |
| K.ITQVLHFTK.D | Complement C4-A precursor | BCP & HP |
| I.PGNSDPNM*IPDGDFNSYVR.V | Complement C4-A precursor | BCP & HP |
| A.LDALSAYWIASHTTEER.G | Complement C4-A precursor | BCP & HP |
| A.SATLYDYYNPER.R | Complement C4-A precursor | BCP & HP |
| D.CREPFLSCCQFAESLR.K | Complement C4-A precursor | BCP & HP |
| D.PLDTLGSEGALSPGGVASLLR.L | Complement C4-A precursor | BCP & HP |
| D.SLALVALGALDTALYAAGSK.S | Complement C4-A precursor | BCP & HP |
| E.FQDALEKLNMGITDLQGLR.L | Complement C4-A precursor | BCP & HP |
| E.QTMIYLAPTLAASR.Y | Complement C4-A precursor | BCP & HP |
| E.VPVGLVQPASATLYDYYNPER.R | Complement C4-A precursor | BCP & HP |
| I.ALDALSAYWIASHTTEER.G | Complement C4-A precursor | BCP & HP |
| K.DHAVDLIQKGYMR.I | Complement C4-A precursor | BCP & HP |
| I.PDISEPGTWK.I | Complement C4-A precursor | BCP & HP |
| K.DHAVDLIQK.G | Complement C4-A precursor | BCP & HP |
| I.PIIIPQTISELQLSVSAGSPHPAIAR.L | Complement C4-A precursor | BCP & HP |
| I.PQTISELQLSVSAGSPHPAIAR.L | Complement C4-A precursor | BCP & HP |
| K.ADGSYAAWLSR.D | Complement C4-A precursor | BCP & HP |
| K.AEFQDALEK.L | Complement C4-A precursor | BCP & HP |
| K.AEFQDALEKLNM*GITDLQGLR.L | Complement C4-A precursor | BCP & HP |
| K.AEFQDALEKLNMGITDLQGLR.L | Complement C4-A precursor | BCP & HP |
| K.AEMADQASAWLTR.Q | Complement C4-A precursor | BCP & HP |
| K.ANSFLGEK.A | Complement C4-A precursor | BCP & HP |
| K.APVDLLGVAHNNLMAMAQETGDNLYWGSVTGSQSNAVSPTPAPR.N | Complement C4-A precursor | BCP & HP |
| K.LQETSNWLLSQQQADGSFQDPCPVLDR.S | Complement C4-A precursor | BCP & HP |
| I.PDGDFNSYVR.V | Complement C4-A precursor | BCP & HP |
| L.SQQQADGSFQDPCPVLDR.S | Complement C4-A precursor | BCP & HP |
| K.VGLSGMAIADVTLLSGFHALRADLEK.L | Complement C4-A precursor | BCP & HP |
| K.LVNGQSHISLSKAEFQDALEK.L | Complement C4-A precursor | BCP & HP |
| K.VLQIEKEGAIHREELVYELNPLDHR.G | Complement C4-A precursor | BCP & HP |
| K.LNM*GITDLQGLR.L | Complement C4-A precursor | BCP & HP |
| K.VLSLAQEQVGGSPEKLQETSNWLLSQQQADGSFQDPCPVLDR.S | Complement C4-A precursor | BCP & HP |
| K.VVEEQESR.V | Complement C4-A precursor | BCP & HP |
| K.YVLPNFEVK.I | Complement C4-A precursor | BCP & HP |
| L.AQEQVGGSPEK.L | Complement C4-A precursor | BCP & HP |
| L.CSAEVCQCAEGK.C | Complement C4-A precursor | BCP & HP |
| L.LSQQQADGSFQDPCPVLDR.S | Complement C4-A precursor | BCP & HP |
| K.VGLSGMAIADVTLLSGFHALR.A | Complement C4-A precursor | BCP & HP |
| L.SLAQEQVGGSPEK.L | Complement C4-A precursor | BCP & HP |
| K.VLQIEKEGAIHR.E | Complement C4-A precursor | BCP & HP |
| M.PSSIFQDDFVIPDISEPGTWK.I | Complement C4-A precursor | BCP & HP |
| P.EVQLVAHSPWLK.D | Complement C4-A precursor | BCP & HP |
| Q.PASATLYDYYNPER.R | Complement C4-A precursor | BCP & HP |
| R.AACAQLNDFLQEYGTQGCQV.- | Complement C4-A precursor | BCP & HP |
| R.ADLEKLTSLSDR.Y | Complement C4-A precursor | BCP & HP |
| R.ADLEKLTSLSDRYVSHFETEGPHVLLYFDSVPTSR.E | Complement C4-A precursor | BCP & HP |
| R.ALEILQEEDLIDEDDIPVR.S | Complement C4-A precursor | BCP & HP |
| R.AVGSGATFSHYYYM*ILSR.G | Complement C4-A precursor | BCP & HP |
| R.AVGSGATFSHYYYMILSR.G | Complement C4-A precursor | BCP & HP |
| R.CCQDGVTR.L | Complement C4-A precursor | BCP & HP |
| L.PRGCGEQTMIYLAPTLAASR.Y | Complement C4-A precursor | BCP & HP |
| K.SCGLHQLLR.G | Complement C4-A precursor | BCP & HP |
| K.LVNGQSHISLSKAEFQDALEKLNM*GITDLQGLR.L | Complement C4-A precursor | BCP & HP |
| K.LVNGQSHISLSKAEFQDALEKLNMGITDLQGLR.L | Complement C4-A precursor | BCP & HP |
| K.M*RPSTDTITVM*VENSHGLR.V | Complement C4-A precursor | BCP & HP |
| K.M*RPSTDTITVMVENSHGLR.V | Complement C4-A precursor | BCP & HP |
| K.MRPSTDTITVM*VENSHGLR.V | Complement C4-A precursor | BCP & HP |
| K.MRPSTDTITVMVENSHGLR.V | Complement C4-A precursor | BCP & HP |
| K.PVQGVAYVR.F | Complement C4-A precursor | BCP & HP |
| K.VLSLAQEQVGGSPEK.L | Complement C4-A precursor | BCP & HP |
| K.RCCQDGVTR.L | Complement C4-A precursor | BCP & HP |
| K.QRVEASISK.A | Complement C4-A precursor | BCP & HP |
| K.SHALQLNNR.Q | Complement C4-A precursor | BCP & HP |
| K.SHKPLNMGK.V | Complement C4-A precursor | BCP & HP |
| K.SRLLATLCSAEVCQCAEGK.C | Complement C4-A precursor | BCP & HP |
| K.TEQWSTLPPETK.D | Complement C4-A precursor | BCP & HP |
| K.TEQWSTLPPETKDHAVDLIQK.G | Complement C4-A precursor | BCP & HP |
| K.TKGLCVATPVQLR.V | Complement C4-A precursor | BCP & HP |
| K.VDFTLSSER.D | Complement C4-A precursor | BCP & HP |
| K.VDFTLSSERDFALLSLQVPLK.D | Complement C4-A precursor | BCP & HP |
| K.VDFTLSSERDFALLSLQVPLKDAK.S | Complement C4-A precursor | BCP & HP |
| K.VFEAM*NSYDLGCGPGGGDSALQVFQAAGLAFSDGDQWTLSR.K | Complement C4-A precursor | BCP & HP |
| K.VFEAMNSYDLGCGPGGGDSALQVFQAAGLAFSDGDQWTLSR.K | Complement C4-A precursor | BCP & HP |
| K.VGLSGM*AIADVTLLSGFHALR.A | Complement C4-A precursor | BCP & HP |
| K.YKATLLDIYKTGEAVAEKDSEITFIK.K | Complement C5 precursor | BCP & HP |
| K.TSTSEEVCSFYLK.I | Complement C5 precursor | BCP & HP |
| R.EKFSDASYQSINIPVTQNM*VPSSR.L | Complement C5 precursor | BCP & HP |
| K.VQVKDSLDQLVGGVPVTLNAQTIDVNQETSDLDPSK.S | Complement C5 precursor | BCP & HP |
| K.SPYIDKITHYNYLILSK.G | Complement C5 precursor | BCP & HP |
| K.VSITSITVENVFVK.Y | Complement C5 precursor | BCP & HP |
| K.VTCTNAELVK.G | Complement C5 precursor | BCP & HP |
| K.VFKDVFLEMNIPYSVVR.G | Complement C5 precursor | BCP & HP |
| K.YKATLLDIYK.T | Complement C5 precursor | BCP & HP |
| K.VFKDVFLEM*NIPYSVVR.G | Complement C5 precursor | BCP & HP |
| K.YKEDFSTTGTAYFEVK.E | Complement C5 precursor | BCP & HP |
| K.YNFSFR.Y | Complement C5 precursor | BCP & HP |
| K.YVEQNQNSICNSLLWLVENYQLDNGSFK.E | Complement C5 precursor | BCP & HP |
| K.YVEQNQNSICNSLLWLVENYQLDNGSFKENSQYQPIK.L | Complement C5 precursor | BCP & HP |
| K.YVLSPYK.L | Complement C5 precursor | BCP & HP |
| R.EALVKGNPPIYR.F | Complement C5 precursor | BCP & HP |
| R.EESSSGSSHAVMDISLPTGISANEEDLK.A | Complement C5 precursor | BCP & HP |
| K.VTCTNAELVKGR.Q | Complement C5 precursor | BCP & HP |
| K.LQGTLPVEAR.E | Complement C5 precursor | BCP & HP |
| K.QTACKPEIAYAYK.V | Complement C5 precursor | BCP & HP |
| K.QLQFALPDSLTTWEIQGVGISNTGICVADTVK.A | Complement C5 precursor | BCP & HP |
| K.QLPGGQNPVSYVYLEVVSK.H | Complement C5 precursor | BCP & HP |
| K.QCTMFYSTSNIK.I | Complement C5 precursor | BCP & HP |
| K.QCTM*FYSTSNIK.I | Complement C5 precursor | BCP & HP |
| K.NFKNFEITIK.A | Complement C5 precursor | BCP & HP |
| K.VFKDVFLEMNIPYSVVRGEQIQLK.G | Complement C5 precursor | BCP & HP |
| K.M*SAVEGICTSESPVIDHQGTK.S | Complement C5 precursor | BCP & HP |
| K.RM*PITYDNGFLFIHTDKPVYTPDQSVK.V | Complement C5 precursor | BCP & HP |
| K.TLLPVSKPEIR.S | Complement C5 precursor | BCP & HP |
| K.TGEAVAEKDSEITFIK.K | Complement C5 precursor | BCP & HP |
| K.TGEAVAEKDSEITFIKK.V | Complement C5 precursor | BCP & HP |
| K.RMPITYDNGFLFIHTDKPVYTPDQSVK.V | Complement C5 precursor | BCP & HP |
| K.TDAPDLPEENQAR.E | Complement C5 precursor | BCP & HP |
| K.SDLGCGAGGGLNNANVFHLAGLTFLTNANADDSQENDEPCK.E | Complement C5 precursor | BCP & HP |
| K.LNLVATPLFLKPGIPYPIKVQVK.D | Complement C5 precursor | BCP & HP |
| K.MSAVEGICTSESPVIDHQGTK.S | Complement C5 precursor | BCP & HP |
| R.TSGMQFCVK.M | Complement C5 precursor | BCP & HP |
| K.EFPYRIPLDLVPK.T | Complement C5 precursor | BCP & HP |
| R.YIYPLDSLTWIEYWPR.D | Complement C5 precursor | BCP & HP |
| R.YGMWTIK.A | Complement C5 precursor | BCP & HP |
| R.YGM*WTIK.A | Complement C5 precursor | BCP & HP |
| R.YGGGFYSTQDTINAIEGLTEYSLLVK.Q | Complement C5 precursor | BCP & HP |
| R.VYSLNDDLKPAKR.E | Complement C5 precursor | BCP & HP |
| R.VYSLNDDLKPAK.R | Complement C5 precursor | BCP & HP |
| R.M*VETTAYALLTSLNLKDINYVNPVIK.W | Complement C5 precursor | BCP & HP |
| R.VDDGVASFVLNLPSGVTVLEFNVK.T | Complement C5 precursor | BCP & HP |
| R.M*VETTAYALLTSLNLK.D | Complement C5 precursor | BCP & HP |
| R.TSGM*QFCVK.M | Complement C5 precursor | BCP & HP |
| R.SYFPESWLWEVHLVPR.R | Complement C5 precursor | BCP & HP |
| R.SIVSALKR.E | Complement C5 precursor | BCP & HP |
| R.QYLIMGK.E | Complement C5 precursor | BCP & HP |
| R.NADYSYSVWK.G | Complement C5 precursor | BCP & HP |
| R.MVETTAYALLTSLNLKDINYVNPVIK.W | Complement C5 precursor | BCP & HP |
| R.MVETTAYALLTSLNLK.D | Complement C5 precursor | BCP & HP |
| R.VLGQVNKYVEQNQNSICNSLLWLVENYQLDNGSFK.E | Complement C5 precursor | BCP & HP |
| R.IPLDLVPKTEIKR.I | Complement C5 precursor | BCP & HP |
| R.ENSLYLTAFTVIGIR.K | Complement C5 precursor | BCP & HP |
| R.ENSLYLTAFTVIGIRK.A | Complement C5 precursor | BCP & HP |
| R.ESYSGVTLDPR.G | Complement C5 precursor | BCP & HP |
| R.ETVLTFIDPEGSEVDM*VEEIDHIGIISFPDFK.I | Complement C5 precursor | BCP & HP |
| R.ETVLTFIDPEGSEVDMVEEIDHIGIISFPDFK.I | Complement C5 precursor | BCP & HP |
| R.ETVLTFIDPEGSEVDMVEEIDHIGIISFPDFKIPSNPR.Y | Complement C5 precursor | BCP & HP |
| R.GYGNSDYK.R | Complement C5 precursor | BCP & HP |
| K.LNLVATPLFLKPGIPYPIK.V | Complement C5 precursor | BCP & HP |
| R.IPLDLVPK.T | Complement C5 precursor | BCP & HP |
| R.MPITYDNGFLFIHTDKPVYTPDQSVK.V | Complement C5 precursor | BCP & HP |
| R.IVACASYKPSR.E | Complement C5 precursor | BCP & HP |
| R.KAFDICPLVK.I | Complement C5 precursor | BCP & HP |
| R.KEFPYRIPLDLVPK.T | Complement C5 precursor | BCP & HP |
| R.KQTACKPEIAYAYK.V | Complement C5 precursor | BCP & HP |
| R.LSM*DIDVSYK.H | Complement C5 precursor | BCP & HP |
| R.LSMDIDVSYK.H | Complement C5 precursor | BCP & HP |
| R.M*PITYDNGFLFIHTDKPVYTPDQSVK.V | Complement C5 precursor | BCP & HP |
| R.EKFSDASYQSINIPVTQNMVPSSR.L | Complement C5 precursor | BCP & HP |
| R.GYGNSDYKR.I | Complement C5 precursor | BCP & HP |
| K.ATLLDIYKTGEAVAEKDSEITFIK.K | Complement C5 precursor | BCP & HP |
| K.DSSVPNTGTARMVETTAYALLTSLNLKDINYVNPVIK.W | Complement C5 precursor | BCP & HP |
| K.DSSVPNTGTAR.M | Complement C5 precursor | BCP & HP |
| K.DSLDQLVGGVPVTLNAQTIDVNQETSDLDPSKSVTR.V | Complement C5 precursor | BCP & HP |
| K.DSLDQLVGGVPVTLNAQTIDVNQETSDLDPSK.S | Complement C5 precursor | BCP & HP |
| K.DSEITFIKK.V | Complement C5 precursor | BCP & HP |
| K.DNLQHKDSSVPNTGTAR.M | Complement C5 precursor | BCP & HP |
| K.DINYVNPVIK.W | Complement C5 precursor | BCP & HP |
| K.DGHVILQLNSIPSSDFLCVR.F | Complement C5 precursor | BCP & HP |
| K.CVEADCGQMQEELDLTISAETRK.Q | Complement C5 precursor | BCP & HP |
| K.CVEADCGQMQEELDLTISAETR.K | Complement C5 precursor | BCP & HP |
| K.DVFLEM*NIPYSVVR.G | Complement C5 precursor | BCP & HP |
| K.CCYDGACVNNDETCEQR.A | Complement C5 precursor | BCP & HP |
| K.ATLLDIYKTGEAVAEKDSEITFIKK.V | Complement C5 precursor | BCP & HP |
| K.ALVEGVDQLFTDYQIK.D | Complement C5 precursor | BCP & HP |
| K.ALLVGEHLNIIVTPK.S | Complement C5 precursor | BCP & HP |
| K.AKVFKDVFLEMNIPYSVVR.G | Complement C5 precursor | BCP & HP |
| K.AKVFKDVFLEM*NIPYSVVR.G | Complement C5 precursor | BCP & HP |
| K.AFTECCVVASQLR.A | Complement C5 precursor | BCP & HP |
| K.AFDICPLVK.I | Complement C5 precursor | BCP & HP |
| K.ADNFLLENTLPAQSTFTLAISAYALSLGDKTHPQFR.S | Complement C5 precursor | BCP & HP |
| K.ADNFLLENTLPAQSTFTLAISAYALSLGDK.T | Complement C5 precursor | BCP & HP |
| I.PVTQNMVPSSR.L | Complement C5 precursor | BCP & HP |
| K.EGM*LSIMSYR.N | Complement C5 precursor | BCP & HP |
| K.LKEGMLSIMSYR.N | Complement C5 precursor | BCP & HP |
| K.CVEADCGQM*QEELDLTISAETR.K | Complement C5 precursor | BCP & HP |
| K.IQKVCEGAACK.C | Complement C5 precursor | BCP & HP |
| K.LKEGMLSIM*SYR.N | Complement C5 precursor | BCP & HP |
| K.LKEGM*LSIMSYR.N | Complement C5 precursor | BCP & HP |
| K.ATLLDIYK.T | Complement C5 precursor | BCP & HP |
| K.DVFLEMNIPYSVVR.G | Complement C5 precursor | BCP & HP |
| K.LKEGM*LSIM*SYR.N | Complement C5 precursor | BCP & HP |
| K.KVTCTNAELVK.G | Complement C5 precursor | BCP & HP |
| K.KIEEIAAK.Y | Complement C5 precursor | BCP & HP |
| K.ITHYNYLILSK.G | Complement C5 precursor | BCP & HP |
| K.IDTQDIEASHYR.G | Complement C5 precursor | BCP & HP |
| K.GTVYNYR.T | Complement C5 precursor | BCP & HP |
| K.GRQYLIMGK.E | Complement C5 precursor | BCP & HP |
| K.GLLVGEILSAVLSQEGINILTHLPK.G | Complement C5 precursor | BCP & HP |
| K.FQNSAILTIQPK.Q | Complement C5 precursor | BCP & HP |
| K.EGMLSIMSYR.N | Complement C5 precursor | BCP & HP |
| K.ELSYYSLEDLNNK.Y | Complement C5 precursor | BCP & HP |
| K.KCCYDGACVNNDETCEQR.A | Complement C5 precursor | BCP & HP |
| K.ENSQYQPIKLQGTLPVEAR.E | Complement C5 precursor | BCP & HP |
| K.EGM*LSIM*SYR.N | Complement C5 precursor | BCP & HP |
| K.EYVLPHFSVSIEPEYNFIGYK.N | Complement C5 precursor | BCP & HP |
| K.ENSQYQPIK.L | Complement C5 precursor | BCP & HP |
| K.FSDASYQSINIPVTQNM*VPSSR.L | Complement C5 precursor | BCP & HP |
| K.FSDASYQSINIPVTQNMVPSSR.L | Complement C5 precursor | BCP & HP |
| K.FSYSSGHVHLSSENK.F | Complement C5 precursor | BCP & HP |
| K.GALHNYK.M | Complement C5 precursor | BCP & HP |
| K.GGSASTWLTAFALR.V | Complement C5 precursor | BCP & HP |
| R.GNLCVNLMR.G | complement component 1, q subcomponent, B chain precursor | BCP & HP |
| R.GNLCVNLM*R.G | complement component 1, q subcomponent, B chain precursor | BCP & HP |
| R.FDHVITNMNNNYEPR.S | complement component 1, q subcomponent, B chain precursor | BCP & HP |
| K.VVTFCDYAYNTFQVTTGGMVLK.L | complement component 1, q subcomponent, B chain precursor | BCP & HP |
| R.FDHVITNM*NNNYEPR.S | complement component 1, q subcomponent, B chain precursor | BCP & HP |
| R.DQTIRFDHVITNMNNNYEPR.S | complement component 1, q subcomponent, B chain precursor | BCP & HP |
| R.DQTIRFDHVITNM*NNNYEPR.S | complement component 1, q subcomponent, B chain precursor | BCP & HP |
| K.LEQGENVFLQATDK.N | complement component 1, q subcomponent, B chain precursor | BCP & HP |
| K.VVTFCDYAYNTFQVTTGGM*VLK.L | complement component 1, q subcomponent, B chain precursor | BCP & HP |
| K.VPGLYYFTYHASSR.G | complement component 1, q subcomponent, B chain precursor | BCP & HP |
| K.LEQGENVFLQATDKNSLLGM*EGANSIFSGFLLFPDM*EA.- | complement component 1, q subcomponent, B chain precursor | BCP & HP |
| R.GGRSEYGAALAWEK.G | Complement component 6 precursor | BCP & HP |
| K.LKGHCQLGQK.Q | Complement component 6 precursor | BCP & HP |
| K.LSEKHEGSFIQGAEK.S | Complement component 6 precursor | BCP & HP |
| K.MEILHPGK.C | Complement component 6 precursor | BCP & HP |
| K.NSGLTEEEAKHCVR.I | Complement component 6 precursor | BCP & HP |
| K.QSGSECICMSPEEDCSHHSEDLCVFDTDSNDYFTSPACK.F | Complement component 6 precursor | BCP & HP |
| K.RSENINHNSAFK.Q | Complement component 6 precursor | BCP & HP |
| K.YNPIPSVQLMGNGFHFLAGEPR.G | Complement component 6 precursor | BCP & HP |
| K.TFSEWLESVK.E | Complement component 6 precursor | BCP & HP |
| K.TFSEWLESVKENPAVIDFELAPIVDLVR.N | Complement component 6 precursor | BCP & HP |
| K.TLNICEVGTIR.C | Complement component 6 precursor | BCP & HP |
| K.DLHLSDVFLK.A | Complement component 6 precursor | BCP & HP |
| R.GGRSEYGAALAWEKGSSGLEEK.T | Complement component 6 precursor | BCP & HP |
| K.YYQENFCEQICSK.Q | Complement component 6 precursor | BCP & HP |
| R.CLPDGTWR.Q | Complement component 6 precursor | BCP & HP |
| R.CPINCLLGDFGPWSDCDPCIEK.Q | Complement component 6 precursor | BCP & HP |
| R.ECNNPAPQR.G | Complement component 6 precursor | BCP & HP |
| R.GEVLDNSFTGGICK.T | Complement component 6 precursor | BCP & HP |
| K.LECNGENDCGDNSDERDCGR.T | Complement component 6 precursor | BCP & HP |
| K.TDFYKDLTSLGHNENQQGSFSSQGGSSFSVPIFYSSK.R | Complement component 6 precursor | BCP & HP |
| K.ESCGYDTCYDWEK.C | Complement component 6 precursor | BCP & HP |
| R.SEYGAALAWEKGSSGLEEK.T | Complement component 6 precursor | BCP & HP |
| R.IFDDFGTHYFTSGSLGGVYDLLYQFSSEELK.N | Complement component 6 precursor | BCP & HP |
| K.AKDLHLSDVFLK.A | Complement component 6 precursor | BCP & HP |
| K.ALNHLPLEYNSALYSR.I | Complement component 6 precursor | BCP & HP |
| K.ALQEYAAK.F | Complement component 6 precursor | BCP & HP |
| K.CLNNQQLHFLHIGSCQDGR.Q | Complement component 6 precursor | BCP & HP |
| K.CVCLLPPQCFK.G | Complement component 6 precursor | BCP & HP |
| K.DLTSLGHNENQQGSFSSQGGSSFSVPIFYSSKR.S | Complement component 6 precursor | BCP & HP |
| K.ENPAVIDFELAPIVDLVR.N | Complement component 6 precursor | BCP & HP |
| K.LCKIEEADCKNK.F | Complement component 6 precursor | BCP & HP |
| K.FDPCQCAPCPNNGRPTLSGTECLCVCQSGTYGENCEK.Q | Complement component 6 precursor | BCP & HP |
| K.GFVVAGPSR.Y | Complement component 6 precursor | BCP & HP |
| K.GGNQLYCVK.M | Complement component 6 precursor | BCP & HP |
| K.HEGSFIQGAEK.S | Complement component 6 precursor | BCP & HP |
| K.IEEADCKNKFR.C | Complement component 6 precursor | BCP & HP |
| K.KDSSFIR.I | Complement component 6 precursor | BCP & HP |
| K.KESCGYDTCYDWEK.C | Complement component 6 precursor | BCP & HP |
| K.LCKIEEADCK.N | Complement component 6 precursor | BCP & HP |
| K.DLTSLGHNENQQGSFSSQGGSSFSVPIFYSSK.R | Complement component 6 precursor | BCP & HP |
| R.SVLRPSQFGGQPCTEPLVAFQPCIPSKLCK.I | Complement component 6 precursor | BCP & HP |
| R.IFDDFGTHYFTSGSLGGVYDLLYQFSSEELKNSGLTEEEAK.H | Complement component 6 precursor | BCP & HP |
| R.KYNPIPSVQLMGNGFHFLAGEPR.G | Complement component 6 precursor | BCP & HP |
| R.SENINHNSAFK.Q | Complement component 6 precursor | BCP & HP |
| R.QGDVECQRTECIKPVVQEVLTITPFQR.L | Complement component 6 precursor | BCP & HP |
| R.QIVVDKYYQENFCEQICSK.Q | Complement component 6 precursor | BCP & HP |
| R.QLEWGLER.T | Complement component 6 precursor | BCP & HP |
| R.KMEILHPGK.C | Complement component 6 precursor | BCP & HP |
| R.SVLRPSQFGGQPCTEPLVAFQPCIPSK.L | Complement component 6 precursor | BCP & HP |
| R.KYNPIPSVQLM*GNGFHFLAGEPR.G | Complement component 6 precursor | BCP & HP |
| R.TECIKPVVQEVLTITPFQR.L | Complement component 6 precursor | BCP & HP |
| R.TRECNNPAPQR.G | Complement component 6 precursor | BCP & HP |
| R.TSNPYRVPANLENVGFEVQTAEDDLK.T | Complement component 6 precursor | BCP & HP |
| R.TSNPYRVPANLENVGFEVQTAEDDLKTDFYK.D | Complement component 6 precursor | BCP & HP |
| R.VPANLENVGFEVQTAEDDLKTDFYK.D | Complement component 6 precursor | BCP & HP |
| R.YTCQGNSWTPPISNSLTCEK.D | Complement component 6 precursor | BCP & HP |
| R.YTCQGNSWTPPISNSLTCEKDTLTK.L | Complement component 6 precursor | BCP & HP |
| R.SEYGAALAWEK.G | Complement component 6 precursor | BCP & HP |
| R.IGESIELTCPK.G | Complement component 6 precursor | BCP & HP |
| R.KALQEYAAK.F | Complement component 6 precursor | BCP & HP |
| R.KLECNGENDCGDNSDER.D | Complement component 6 precursor | BCP & HP |
| R.KLECNGENDCGDNSDERDCGR.T | Complement component 6 precursor | BCP & HP |
| R.NIPCAVTKR.N | Complement component 6 precursor | BCP & HP |
| K.WSEGPCGSPGSPGSNIEGFVCK.F | Complement component C1q receptor precursor | BCP & HP |
| K.LSAAEAQNHCNQNGGNLATVK.S | Complement component C1q receptor precursor | BCP & HP |
| R.LLDDLVTCASR.N | Complement component C1q receptor precursor | BCP & HP |
| K.QNDFNSVEEKK.C | Complement component C7 precursor | BCP & HP |
| K.M*HVLHCQGR.N | Complement component C7 precursor | BCP & HP |
| K.LKQNDFNSVEEKK.C | Complement component C7 precursor | BCP & HP |
| K.M*PYECGPSLDVCAQDER.S | Complement component C7 precursor | BCP & HP |
| K.MHVLHCQGR.N | Complement component C7 precursor | BCP & HP |
| K.MPYECGPSLDVCAQDER.S | Complement component C7 precursor | BCP & HP |
| K.LKQNDFNSVEEK.K | Complement component C7 precursor | BCP & HP |
| K.QKLTPLYELVK.E | Complement component C7 precursor | BCP & HP |
| K.EQTMSECEAGALR.C | Complement component C7 precursor | BCP & HP |
| K.RILPLTVCK.M | Complement component C7 precursor | BCP & HP |
| K.SFGGQCR.K | Complement component C7 precursor | BCP & HP |
| K.SLVCNGDSDCDEDSADEDR.C | Complement component C7 precursor | BCP & HP |
| K.NVVYTCNEGYSLIGNPVAR.C | Complement component C7 precursor | BCP & HP |
| K.ELENALK.N | Complement component C7 precursor | BCP & HP |
| A.SSPVNCQWDFYAPWSECNGCTK.T | Complement component C7 precursor | BCP & HP |
| I.DQYGTHYLQSGSLGGEYR.V | Complement component C7 precursor | BCP & HP |
| K.AASGTQNNVLR.G | Complement component C7 precursor | BCP & HP |
| K.AASGTQNNVLRGEPFIR.G | Complement component C7 precursor | BCP & HP |
| K.ACGACPLWGK.C | Complement component C7 precursor | BCP & HP |
| K.ACGACPLWGKCDAESSK.C | Complement component C7 precursor | BCP & HP |
| K.EVPCASVKK.L | Complement component C7 precursor | BCP & HP |
| K.DGFVQDEGTMFPVGK.N | Complement component C7 precursor | BCP & HP |
| K.IACVLPVLMDGIQSHPQKPFYTVGEK.V | Complement component C7 precursor | BCP & HP |
| K.ELSHLPSLYDYSAYR.R | Complement component C7 precursor | BCP & HP |
| K.ELSHLPSLYDYSAYRR.L | Complement component C7 precursor | BCP & HP |
| K.EQTM*SECEAGALR.C | Complement component C7 precursor | BCP & HP |
| K.VFSGDGKDFYR.L | Complement component C7 precursor | BCP & HP |
| K.EVPCASVK.K | Complement component C7 precursor | BCP & HP |
| K.FSSHGCKELENALK.A | Complement component C7 precursor | BCP & HP |
| K.IACVLPVLM*DGIQSHPQKPFYTVGEK.V | Complement component C7 precursor | BCP & HP |
| K.DGFVQDEGTM*FPVGK.N | Complement component C7 precursor | BCP & HP |
| R.SYTSHTNEIHK.G | Complement component C7 precursor | BCP & HP |
| K.SSGWHFVVK.F | Complement component C7 precursor | BCP & HP |
| R.RPSCDIDKPPPNIELTGNGYNELTGQFR.N | Complement component C7 precursor | BCP & HP |
| K.SLVCNGDSDCDEDSADEDRCEDSER.R | Complement component C7 precursor | BCP & HP |
| R.RYSAWAESVTNLPQVIK.Q | Complement component C7 precursor | BCP & HP |
| R.SCVGETTESTQCEDEELEHLR.L | Complement component C7 precursor | BCP & HP |
| R.SRECNNPPPSGGGR.S | Complement component C7 precursor | BCP & HP |
| R.LSGNVLSYTFQVK.I | Complement component C7 precursor | BCP & HP |
| R.SVAVYGQYGGQPCVGNAFETQSCEPTRGCPTEEGCGER.F | Complement component C7 precursor | BCP & HP |
| R.RLIDQYGTHYLQSGSLGGEYR.V | Complement component C7 precursor | BCP & HP |
| R.SYTSHTNEIHKGK.S | Complement component C7 precursor | BCP & HP |
| R.VLFYVDSEK.L | Complement component C7 precursor | BCP & HP |
| R.VLFYVDSEKLK.Q | Complement component C7 precursor | BCP & HP |
| R.VLFYVDSEKLKQNDFNSVEEK.K | Complement component C7 precursor | BCP & HP |
| R.WLVGEM*HCQK.I | Complement component C7 precursor | BCP & HP |
| R.WLVGEMHCQK.I | Complement component C7 precursor | BCP & HP |
| R.YSAWAESVTNLPQVIK.Q | Complement component C7 precursor | BCP & HP |
| R.SVAVYGQYGGQPCVGNAFETQSCEPTR.G | Complement component C7 precursor | BCP & HP |
| R.DSCTLPASAEK.A | Complement component C7 precursor | BCP & HP |
| K.VTVSCSGGM*SLEGPSAFLCGSSLK.W | Complement component C7 precursor | BCP & HP |
| K.VTVSCSGGMSLEGPSAFLCGSSLK.W | Complement component C7 precursor | BCP & HP |
| R.CFSGQCISK.S | Complement component C7 precursor | BCP & HP |
| R.RSVAVYGQYGGQPCVGNAFETQSCEPTR.G | Complement component C7 precursor | BCP & HP |
| R.CVQKENPLTQAVPK.C | Complement component C7 precursor | BCP & HP |
| R.LLEPHCFPLSLVPTEFCPSPPALKDGFVQDEGTMFPVGK.N | Complement component C7 precursor | BCP & HP |
| R.GCPTEEGCGER.F | Complement component C7 precursor | BCP & HP |
| R.GGGAGFISGLSYLELDNPAGNK.R | Complement component C7 precursor | BCP & HP |
| R.LLEPHCFPLSLVPTEFCPSPPALKDGFVQDEGTM*FPVGK.N | Complement component C7 precursor | BCP & HP |
| R.GGGAGFISGLSYLELDNPAGNKRR.Y | Complement component C7 precursor | BCP & HP |
| R.GQSISVTSIRPCAAETQ.- | Complement component C7 precursor | BCP & HP |
| R.ILPLTVCK.M | Complement component C7 precursor | BCP & HP |
| R.KVFSGDGKDFYR.L | Complement component C7 precursor | BCP & HP |
| R.LIDQYGTHYLQSGSLGGEYR.V | Complement component C7 precursor | BCP & HP |
| R.LLEPHCFPLSLVPTEFCPSPPALK.D | Complement component C7 precursor | BCP & HP |
| R.GGGAGFISGLSYLELDNPAGNKR.R | Complement component C7 precursor | BCP & HP |
| R.CRGQSISVTSIRPCAAETQ.- | Complement component C7 precursor | BCP & HP |
| R.HLVCNGDQDCLDGSDEDDCEDVRAIDEDCSQYEPIPGSQK.A | Complement component C8 alpha chain precursor | BCP & HP |
| R.AIDEDCSQYEPIPGSQK.A | Complement component C8 alpha chain precursor | BCP & HP |
| R.ALDQYLM*EFNACR.C | Complement component C8 alpha chain precursor | BCP & HP |
| R.ALDQYLMEFNACR.C | Complement component C8 alpha chain precursor | BCP & HP |
| R.CGPCFNNGVPILEGTSCR.C | Complement component C8 alpha chain precursor | BCP & HP |
| R.DITTCFGGSLGIQYEDK.I | Complement component C8 alpha chain precursor | BCP & HP |
| R.ECDNPAPQNGGASCPGR.K | Complement component C8 alpha chain precursor | BCP & HP |
| R.ECDNPAPQNGGASCPGRK.V | Complement component C8 alpha chain precursor | BCP & HP |
| R.HTSLGPLEAK.R | Complement component C8 alpha chain precursor | BCP & HP |
| R.HLVCNGDQDCLDGSDEDDCEDVR.A | Complement component C8 alpha chain precursor | BCP & HP |
| K.YNPVVIDFEMQPIHEVLR.H | Complement component C8 alpha chain precursor | BCP & HP |
| K.MRKDDIMLDEGM*LQSLMELPDQYNYGMYAK.F | Complement component C8 alpha chain precursor | BCP & HP |
| R.KAMAVEDIISR.V | Complement component C8 alpha chain precursor | BCP & HP |
| R.KAM*AVEDIISR.V | Complement component C8 alpha chain precursor | BCP & HP |
| R.ELRYDSTCER.L | Complement component C8 alpha chain precursor | BCP & HP |
| K.YNPVVIDFEM*QPIHEVLR.H | Complement component C8 alpha chain precursor | BCP & HP |
| K.YHFEALADTGISSEFYDNANDLLSK.V | Complement component C8 alpha chain precursor | BCP & HP |
| K.TERARKAMAVEDIISR.V | Complement component C8 alpha chain precursor | BCP & HP |
| K.RQNLRRALDQYLM*EFNACR.C | Complement component C8 alpha chain precursor | BCP & HP |
| K.RHLVCNGDQDCLDGSDEDDCEDVR.A | Complement component C8 alpha chain precursor | BCP & HP |
| K.INVGGGLSGDHCKK.F | Complement component C8 alpha chain precursor | BCP & HP |
| K.MRKDDIMLDEGMLQSLM*ELPDQYNYGMYAK.F | Complement component C8 alpha chain precursor | BCP & HP |
| K.MRKDDIMLDEGM*LQSLMELPDQYNYGM*YAK.F | Complement component C8 alpha chain precursor | BCP & HP |
| K.MESLGITSR.D | Complement component C8 alpha chain precursor | BCP & HP |
| K.M*RKDDIMLDEGMLQSLMELPDQYNYGMYAK.F | Complement component C8 alpha chain precursor | BCP & HP |
| K.M*ESLGITSR.D | Complement component C8 alpha chain precursor | BCP & HP |
| K.AALGYNILTQEDAQSVYDASYYGGQCETVYNGEWR.E | Complement component C8 alpha chain precursor | BCP & HP |
| R.KDDIM*LDEGMLQSLMELPDQYNYGM*YAK.F | Complement component C8 alpha chain precursor | BCP & HP |
| G.SLGAACEQTQTEGAK.A | Complement component C8 alpha chain precursor | BCP & HP |
| K.MRKDDIMLDEGMLQSLMELPDQYNYGMYAK.F | Complement component C8 alpha chain precursor | BCP & HP |
| K.AKM*ESLGITSR.D | Complement component C8 alpha chain precursor | BCP & HP |
| R.KDDIMLDEGMLQSLMELPDQYNYGMYAK.F | Complement component C8 alpha chain precursor | BCP & HP |
| R.KDDIMLDEGM*LQSLMELPDQYNYGMYAK.F | Complement component C8 alpha chain precursor | BCP & HP |
| R.KDDIMLDEGMLQSLMELPDQYNYGM*YAK.F | Complement component C8 alpha chain precursor | BCP & HP |
| R.LGSLGAACEQTQTEGAK.A | Complement component C8 alpha chain precursor | BCP & HP |
| K.AKMESLGITSR.D | Complement component C8 alpha chain precursor | BCP & HP |
| K.INVGGGLSGDHCK.K | Complement component C8 alpha chain precursor | BCP & HP |
| K.AM*AVEDIISR.V | Complement component C8 alpha chain precursor | BCP & HP |
| K.AMAVEDIISR.V | Complement component C8 alpha chain precursor | BCP & HP |
| K.FGGGKTER.A | Complement component C8 alpha chain precursor | BCP & HP |
| K.FGGTICSGDIWDQASCSSSTTCVR.Q | Complement component C8 alpha chain precursor | BCP & HP |
| K.FGGTICSGDIWDQASCSSSTTCVRQ.A | Complement component C8 alpha chain precursor | BCP & HP |
| K.FINDYGTHYITSGSM*GGIYEYILVIDK.A | Complement component C8 alpha chain precursor | BCP & HP |
| R.RALDQYLM*EFNACR.C | Complement component C8 alpha chain precursor | BCP & HP |
| R.SLLQPNKFGGTICSGDIWDQASCSSSTTCVRQ.A | Complement component C8 alpha chain precursor | BCP & HP |
| R.SLLQPNKFGGTICSGDIWDQASCSSSTTCVR.Q | Complement component C8 alpha chain precursor | BCP & HP |
| R.SLKYNPVVIDFEMQPIHEVLR.H | Complement component C8 alpha chain precursor | BCP & HP |
| R.RRECDNPAPQNGGASCPGR.K | Complement component C8 alpha chain precursor | BCP & HP |
| R.RECDNPAPQNGGASCPGRK.V | Complement component C8 alpha chain precursor | BCP & HP |
| R.RECDNPAPQNGGASCPGR.K | Complement component C8 alpha chain precursor | BCP & HP |
| R.RALDQYLMEFNACR.C | Complement component C8 alpha chain precursor | BCP & HP |
| R.QAQCGQDFQCKETGR.C | Complement component C8 alpha chain precursor | BCP & HP |
| K.FINDYGTHYITSGSMGGIYEYILVIDK.A | Complement component C8 alpha chain precursor | BCP & HP |
| R.LYYGDDEK.Y | Complement component C8 alpha chain precursor | BCP & HP |
| R.LYYGDDEKYFR.K | Complement component C8 alpha chain precursor | BCP & HP |
| R.QAQCGQDFQCK.E | Complement component C8 alpha chain precursor | BCP & HP |
| K.SGFSFGFK.I | Complement component C8 beta chain precursor | BCP & HP |
| R.GDYTLNNVHACAK.N | Complement component C8 beta chain precursor | BCP & HP |
| R.FRKPYNVESYTPQTQGK.Y | Complement component C8 beta chain precursor | BCP & HP |
| R.DTMVEDLVVLVR.G | Complement component C8 beta chain precursor | BCP & HP |
| R.DTM*VEDLVVLVR.G | Complement component C8 beta chain precursor | BCP & HP |
| R.DFGTHYITEAVLGGIYEYTLVMNK.E | Complement component C8 beta chain precursor | BCP & HP |
| R.DFGTHYITEAVLGGIYEYTLVM*NK.E | Complement component C8 beta chain precursor | BCP & HP |
| R.CEGFVCAQTGR.C | Complement component C8 beta chain precursor | BCP & HP |
| R.CDCICPVGSQGLACEVSYRK.N | Complement component C8 beta chain precursor | BCP & HP |
| R.CDCICPVGSQGLACEVSYR.K | Complement component C8 beta chain precursor | BCP & HP |
| R.GGASEHITTLAYQELPTADLM*QEWGDAVQYNPAIIK.V | Complement component C8 beta chain precursor | BCP & HP |
| K.VEPLYELVTATDFAYSSTVR.Q | Complement component C8 beta chain precursor | BCP & HP |
| R.KPYNVESYTPQTQGK.Y | Complement component C8 beta chain precursor | BCP & HP |
| K.RLPLEYSYGEYRDLFR.D | Complement component C8 beta chain precursor | BCP & HP |
| K.RLPLEYSYGEYR.D | Complement component C8 beta chain precursor | BCP & HP |
| K.QALEEFQKEVSSCHCAPCQGNGVPVLK.G | Complement component C8 beta chain precursor | BCP & HP |
| K.QALEEFQK.E | Complement component C8 beta chain precursor | BCP & HP |
| K.IPGIFELGISSQSDRGK.H | Complement component C8 beta chain precursor | BCP & HP |
| K.IPGIFELGISSQSDR.G | Complement component C8 beta chain precursor | BCP & HP |
| K.IGGAIEEVYVSLGVSVGK.C | Complement component C8 beta chain precursor | BCP & HP |
| K.VKVEPLYELVTATDFAYSSTVR.Q | Complement component C8 beta chain precursor | BCP & HP |
| R.RLLCNGDNDCGDQSDEANCR.R | Complement component C8 beta chain precursor | BCP & HP |
| R.YYAGGCSPHYILNTR.F | Complement component C8 beta chain precursor | BCP & HP |
| K.CQHEM*DQYWGIGSLASGINLFTNSFEGPVLDHR.Y | Complement component C8 beta chain precursor | BCP & HP |
| K.CQHEMDQYWGIGSLASGINLFTNSFEGPVLDHR.Y | Complement component C8 beta chain precursor | BCP & HP |
| K.EAMERGDYTLNNVHACAK.N | Complement component C8 beta chain precursor | BCP & HP |
| R.YAYLLQPSQFHGEPCNFSDKEVEDCVTNRPCR.S | Complement component C8 beta chain precursor | BCP & HP |
| R.VKRLPLEYSYGEYR.D | Complement component C8 beta chain precursor | BCP & HP |
| R.SLMLHYEFLQR.V | Complement component C8 beta chain precursor | BCP & HP |
| R.SLM*LHYEFLQR.V | Complement component C8 beta chain precursor | BCP & HP |
| R.GGASEHITTLAYQELPTADLMQEWGDAVQYNPAIIK.V | Complement component C8 beta chain precursor | BCP & HP |
| R.RLLCNGDNDCGDQSDEANCRR.I | Complement component C8 beta chain precursor | BCP & HP |
| K.GSRCDCICPVGSQGLACEVSYRK.N | Complement component C8 beta chain precursor | BCP & HP |
| R.QRQCNNPPPQNGGSPCSGPASETLDCS.- | Complement component C8 beta chain precursor | BCP & HP |
| R.QCNNPPPQNGGSPCSGPASETLDCS.- | Complement component C8 beta chain precursor | BCP & HP |
| R.LPLEYSYGEYR.D | Complement component C8 beta chain precursor | BCP & HP |
| R.LLCNGDNDCGDQSDEANCRR.I | Complement component C8 beta chain precursor | BCP & HP |
| R.LLCNGDNDCGDQSDEANCR.R | Complement component C8 beta chain precursor | BCP & HP |
| R.KPYNVESYTPQTQGKYEFILK.E | Complement component C8 beta chain precursor | BCP & HP |
| K.EVSSCHCAPCQGNGVPVLK.G | Complement component C8 beta chain precursor | BCP & HP |
| R.GILNEIKDR.N | Complement component C8 beta chain precursor | BCP & HP |
| R.SDLEVAHYK.L | Complement component C8 beta chain precursor | BCP & HP |
| K.EAM*ERGDYTLNNVHACAK.N | Complement component C8 beta chain precursor | BCP & HP |
| R.AEATTLHVAPQGTAMAVSTFRK.L | Complement component C8 gamma chain precursor | BCP & HP |
| K.LDGICWQVR.Q | Complement component C8 gamma chain precursor | BCP & HP |
| K.YGFCEAADQFHVLDEV.R | Complement component C8 gamma chain precursor | BCP & HP |
| K.YGFCEAADQFHVLDEVR.R | Complement component C8 gamma chain precursor | BCP & HP |
| K.YGFCEAADQFHVLDEVRR.- | Complement component C8 gamma chain precursor | BCP & HP |
| L.PVSDSVLSGFEQR.V | Complement component C8 gamma chain precursor | BCP & HP |
| R.AEATTLHVAPQGTAM*AVSTFR.K | Complement component C8 gamma chain precursor | BCP & HP |
| K.ANFDAQQFAGTWLLVAVGSACR.F | Complement component C8 gamma chain precursor | BCP & HP |
| R.AEATTLHVAPQGTAMAVSTFR.K | Complement component C8 gamma chain precursor | BCP & HP |
| R.FLQEQGHRAEATTLHVAPQGTAM*AVSTFR.K | Complement component C8 gamma chain precursor | BCP & HP |
| R.FLQEQGHRAEATTLHVAPQGTAMAVSTFR.K | Complement component C8 gamma chain precursor | BCP & HP |
| R.FLQEQGHR.A | Complement component C8 gamma chain precursor | BCP & HP |
| R.KLDGICWQVR.Q | Complement component C8 gamma chain precursor | BCP & HP |
| R.QLYGDTGVLGR.F | Complement component C8 gamma chain precursor | BCP & HP |
| R.RPASPISTIQPK.A | Complement component C8 gamma chain precursor | BCP & HP |
| R.SLPVSDSVLSGFEQR.V | Complement component C8 gamma chain precursor | BCP & HP |
| R.VQEAHLTEDQIFYFPK.Y | Complement component C8 gamma chain precursor | BCP & HP |
| R.AEATTLHVAPQGTAM*AVSTFRK.L | Complement component C8 gamma chain precursor | BCP & HP |
| R.NRDVVLTTTFVDDIK.A | Complement component C9 precursor | BCP & HP |
| T.PFDNEFYNGLCNR.D | Complement component C9 precursor | BCP & HP |
| R.CNGDNDCGDFSDEDDCESEPRPPCRDR.V | Complement component C9 precursor | BCP & HP |
| R.CTDAVGDRR.Q | Complement component C9 precursor | BCP & HP |
| R.DGNTLTYYR.R | Complement component C9 precursor | BCP & HP |
| R.DRDGNTLTYYR.R | Complement component C9 precursor | BCP & HP |
| R.DRVVEESELAR.T | Complement component C9 precursor | BCP & HP |
| R.DVVLTTTFVDDIK.A | Complement component C9 precursor | BCP & HP |
| R.GTVIDVTDFVNWASSINDAPVLISQK.L | Complement component C9 precursor | BCP & HP |
| R.KCHTCQNGGTVILM*DGK.C | Complement component C9 precursor | BCP & HP |
| R.AVNITSENLIDDVVSLIR.G | Complement component C9 precursor | BCP & HP |
| R.LRCNGDNDCGDFSDEDDCESEPRPPCRDR.V | Complement component C9 precursor | BCP & HP |
| R.AIEDYINEFSVRK.C | Complement component C9 precursor | BCP & HP |
| R.QCVPTEPCEDAEDDCGNDFQCSTGR.C | Complement component C9 precursor | BCP & HP |
| R.RPWNVASLIYETK.G | Complement component C9 precursor | BCP & HP |
| R.RQCVPTEPCEDAEDDCGNDFQCSTGR.C | Complement component C9 precursor | BCP & HP |
| R.SIEVFGQFNGK.R | Complement component C9 precursor | BCP & HP |
| R.SIEVFGQFNGKR.C | Complement component C9 precursor | BCP & HP |
| R.TAGYGINILGM*DPLSTPFDNEFYNGLCNR.D | Complement component C9 precursor | BCP & HP |
| R.TAGYGINILGMDPLSTPFDNEFYNGLCNR.D | Complement component C9 precursor | BCP & HP |
| R.TAGYGINILGMDPLSTPFDNEFYNGLCNRDR.D | Complement component C9 precursor | BCP & HP |
| R.TEHYEEQIEAFK.S | Complement component C9 precursor | BCP & HP |
| R.VVEESELAR.T | Complement component C9 precursor | BCP & HP |
| R.KYAFELK.E | Complement component C9 precursor | BCP & HP |
| K.ISEGLPALEFPNE.K | Complement component C9 precursor | BCP & HP |
| D.AEDDCGNDFQCSTGR.C | Complement component C9 precursor | BCP & HP |
| D.PLSTPFDNEFYNGLCNR.D | Complement component C9 precursor | BCP & HP |
| E.PCEDAEDDCGNDFQCSTGR.C | Complement component C9 precursor | BCP & HP |
| G.MDPLSTPFDNEFYNGLCNR.D | Complement component C9 precursor | BCP & HP |
| K.AEQCCEETASSISLHGK.G | Complement component C9 precursor | BCP & HP |
| K.ALPTTYEK.G | Complement component C9 precursor | BCP & HP |
| K.CHTCQNGGTVILMDGK.C | Complement component C9 precursor | BCP & HP |
| K.CLCACPFK.F | Complement component C9 precursor | BCP & HP |
| K.CLCACPFKFEGIACEISK.Q | Complement component C9 precursor | BCP & HP |
| K.FEGIACEISK.Q | Complement component C9 precursor | BCP & HP |
| R.CNGDNDCGDFSDEDDCESEPRPPCR.D | Complement component C9 precursor | BCP & HP |
| K.FTPTETNKAEQCCEETASSISLHGK.G | Complement component C9 precursor | BCP & HP |
| R.LRCNGDNDCGDFSDEDDCESEPRPPCR.D | Complement component C9 precursor | BCP & HP |
| K.ISEGLPALEFPNEK.- | Complement component C9 precursor | BCP & HP |
| K.LSPIYNLVPVK.M | Complement component C9 precursor | BCP & HP |
| K.NFRTEHYEEQIEAFK.S | Complement component C9 precursor | BCP & HP |
| K.QKISEGLPALEFPNEK.- | Complement component C9 precursor | BCP & HP |
| K.TSNFNAAISLK.F | Complement component C9 precursor | BCP & HP |
| K.YAFELKEK.L | Complement component C9 precursor | BCP & HP |
| K.YAFELKEKLLRGTVIDVTDFVNWASSINDAPVLISQK.L | Complement component C9 precursor | BCP & HP |
| M.DPLSTPFDNEFYNGLCNR.D | Complement component C9 precursor | BCP & HP |
| P.WNVASLIYETK.G | Complement component C9 precursor | BCP & HP |
| R.AIEDYINEFSVR.K | Complement component C9 precursor | BCP & HP |
| K.FTPTETNK.A | Complement component C9 precursor | BCP & HP |
| R.KCHTCQNGGTVILMDGK.C | Complement component C9 precursor | BCP & HP |
| R.LMCAESNR.R | Complement factor D preproprotein | BCP & HP |
| K.VQVLLGAHSLSQPEPSKR.L | Complement factor D preproprotein | BCP & HP |
| R.RPDSLQHVLLPVLDR.A | Complement factor D preproprotein | BCP & HP |
| R.DSCKGDSGGPLVCGGVLEGVVTSGSR.V | Complement factor D preproprotein | BCP & HP |
| R.AVPHPDSQPDTIDHDLLLLQLSEK.A | Complement factor D preproprotein | BCP & HP |
| R.VDRDVAPGTLCDVAGWGIVNHAGR.R | Complement factor D preproprotein | BCP & HP |
| K.CGPPPPIDNGDITSFPLSVYAPASSVEYQCQNLYQLEGNKR.I | complement factor H-related 1 | BCP & HP |
| R.TTCWDGKLEYPTCAK.R | complement factor H-related 1 | BCP & HP |
| R.QMSKYPSGER.V | complement factor H-related 1 | BCP & HP |
| K.CLHPCVISR.E | complement factor H-related 1 | BCP & HP |
| R.NGQWSEPPK.C | complement factor H-related 1 | BCP & HP |
| R.TTCWDGKLEYPTCA.K | complement factor H-related 1 | BCP & HP |
| K.INHGILYDEEK.Y | complement factor H-related 1 | BCP & HP |
| K.YKPFSQVPTGEVFYYSCEYNFVSPSK.S | complement factor H-related 1 | BCP & HP |
| R.EIM*ENYNIALR.W | complement factor H-related 1 | BCP & HP |
| R.EIMENYNIALR.W | complement factor H-related 1 | BCP & HP |
| R.ITCTEEGWSPTPK.C | complement factor H-related 1 | BCP & HP |
| R.TGESAEFVCK.R | complement factor H-related 1 | BCP & HP |
| R.LQNNENNISCVER.G | complement factor H-related 1 | BCP & HP |
| K.CGPPPPIDNGDITSFPLSVYAPASSVEYQCQNLYQLEGNK.R | complement factor H-related 1 | BCP & HP |
| R.NGQWSEPPKCLHPCVISR.E | complement factor H-related 1 | BCP & HP |
| R.QM*SKYPSGER.V | complement factor H-related 1 | BCP & HP |
| R.STDTSCVNPPTVQNAHILSR.Q | complement factor H-related 1 | BCP & HP |
| R.TGESAEFVCKR.G | complement factor H-related 1 | BCP & HP |
| A.PASSVEYQCQNLYQLEGNK.R | complement factor H-related 1 | BCP & HP |
| A.PASSVEYQCQNLYQLEGNKR.I | complement factor H-related 1 | BCP & HP |
| F.PLSVYAPASSVEYQCQNLYQLEGNKR.I | complement factor H-related 1 | BCP & HP |
| R.LCFFPFVENGHSESSGQTHLEGDTVQIICNTGYR.L | complement factor H-related 1 | BCP & HP |
| K.INHGILYDEEKYKPFSQVPTGEVFYYSCEYNFVSPSK.S | complement factor H-related 1 | BCP & HP |
| R.LQNNENNISCVER.G | Complement factor H-related protein 1 precursor | BCP & HP |
| K.CGPPPPIDNGDITSFPLSVYAPASSVEYQCQNLYQLEGNK.R | Complement factor H-related protein 1 precursor | BCP & HP |
| K.CGPPPPIDNGDITSFPLSVYAPASSVEYQCQNLYQLEGNKR.I | Complement factor H-related protein 1 precursor | BCP & HP |
| K.CLHPCVISR.E | Complement factor H-related protein 1 precursor | BCP & HP |
| K.INHGILYDEEK.Y | Complement factor H-related protein 1 precursor | BCP & HP |
| K.INHGILYDEEKYKPFSQVPTGEVFYYSCEYNFVSPSK.S | Complement factor H-related protein 1 precursor | BCP & HP |
| K.YKPFSQVPTGEVFYYSCEYNFVSPSK.S | Complement factor H-related protein 1 precursor | BCP & HP |
| N.PPTVQNAYIVSR.Q | Complement factor H-related protein 1 precursor | BCP & HP |
| P.PTVQNAYIVSR.Q | Complement factor H-related protein 1 precursor | BCP & HP |
| R.EIM*ENYNIALR.W | Complement factor H-related protein 1 precursor | BCP & HP |
| R.EIMENYNIALR.W | Complement factor H-related protein 1 precursor | BCP & HP |
| F.PLSVYAPASSVEYQCQNLYQLEGNKR.I | Complement factor H-related protein 1 precursor | BCP & HP |
| R.LCFFPFVENGHSESSGQTHLEGDTVQIICNTGYR.L | Complement factor H-related protein 1 precursor | BCP & HP |
| R.NGQWSEPPKCLHPCVISR.E | Complement factor H-related protein 1 precursor | BCP & HP |
| R.NGQWSEPPK.C | Complement factor H-related protein 1 precursor | BCP & HP |
| R.QM*SKYPSGER.V | Complement factor H-related protein 1 precursor | BCP & HP |
| R.TGESAEFVCK.R | Complement factor H-related protein 1 precursor | BCP & HP |
| A.PASSVEYQCQNLYQLEGNKR.I | Complement factor H-related protein 1 precursor | BCP & HP |
| R.STDTSCVNPPTVQNAYIVSR.Q | Complement factor H-related protein 1 precursor | BCP & HP |
| V.NPPTVQNAYIVSR.Q | Complement factor H-related protein 1 precursor | BCP & HP |
| T.DTSCVNPPTVQNAYIVSR.Q | Complement factor H-related protein 1 precursor | BCP & HP |
| R.TTCWDGKLEYPTCAK.R | Complement factor H-related protein 1 precursor | BCP & HP |
| R.TTCWDGKLEYPTCA.K | Complement factor H-related protein 1 precursor | BCP & HP |
| R.TGESAEFVCKR.G | Complement factor H-related protein 1 precursor | BCP & HP |
| R.ITCTEEGWSPTPK.C | Complement factor H-related protein 1 precursor | BCP & HP |
| R.QMSKYPSGER.V | Complement factor H-related protein 1 precursor | BCP & HP |
| A.PASSVEYQCQNLYQLEGNK.R | Complement factor H-related protein 1 precursor | BCP & HP |
| K.AQTTVTCTEKGWSPTPR.C | Complement factor H-related protein 3 precursor | BCP & HP |
| K.CYFPYLENGYNQNYGR.K | Complement factor H-related protein 3 precursor | BCP & HP |
| K.CYFPYLENGYNQNYGRK.F | Complement factor H-related protein 3 precursor | BCP & HP |
| R.KCYFPYLENGYNQNYGR.K | Complement factor H-related protein 3 precursor | BCP & HP |
| R.KCYFPYLENGYNQNYGRK.F | Complement factor H-related protein 3 precursor | BCP & HP |
| R.RPYFPVAVGK.Y | Complement factor H-related protein 3 precursor | BCP & HP |
| K.YTCM*AQTVVDSASK.E | Contactin-2 precursor | BCP & HP |
| K.YTCMAQTVVDSASKEATVLVRGPPGPPGGVVVR.D | Contactin-2 precursor | BCP & HP |
| R.AAPEASGTPSSDAVSR.L | Coronin-1A | BCP & HP |
| R.DGGLICTSCR.D | Coronin-1A | BCP & HP |
| K.ADQCYEDVR.V | Coronin-1A | BCP & HP |
| K.GTWTQPFDLASTR.E | Corticosteroid-binding globulin precursor | BCP & HP |
| R.QINSYVK.N | Corticosteroid-binding globulin precursor | BCP & HP |
| K.AVLQLNEEGVDTAGSTGVTLNLTSKPIILR.F | Corticosteroid-binding globulin precursor | BCP & HP |
| R.ITQDAQLK.S | Corticosteroid-binding globulin precursor | BCP & HP |
| K.HLVALSPK.K | Corticosteroid-binding globulin precursor | BCP & HP |
| R.WSAGLTSSQVDLYIPK.V | Corticosteroid-binding globulin precursor | BCP & HP |
| K.HYYESEVLAMNFQDWATASR.Q | Corticosteroid-binding globulin precursor | BCP & HP |
| R.SETEIHQGFQHLHQLFAK.S | Corticosteroid-binding globulin precursor | BCP & HP |
| R.GLASANVDFAFSLYK.H | Corticosteroid-binding globulin precursor | BCP & HP |
| R.ALDFAVGEYNK.A | Cystatin-C precursor | BCP & HP |
| R.LVGGPM*DASVEEEGVRR.A | Cystatin-C precursor | BCP & HP |
| K.QIVAGVNYFLDVELGR.T | Cystatin-C precursor | BCP & HP |
| R.LVGGPMDASVEEEGVR.R | Cystatin-C precursor | BCP & HP |
| R.RALDFAVGEYNK.A | Cystatin-C precursor | BCP & HP |
| K.TQPNLDNCPFHDQPHLK.R | Cystatin-C precursor | BCP & HP |
| K.ASNDMYHSR.A | Cystatin-C precursor | BCP & HP |
| K.AFCSFQIYAVPWQGTMTLSK.S | Cystatin-C precursor | BCP & HP |
| R.LVGGPMDASVEEEGVRR.A | Cystatin-C precursor | BCP & HP |
| R.DLSPDDPQVQK.A | Cystatin-M precursor | BCP & HP |
| R.VTGDHVDLTTCPLAAGAQQEK.L | Cystatin-M precursor | BCP & HP |
| K.AQSQLVAGIK.Y | Cystatin-M precursor | BCP & HP |
| K.TGPNLHGLFGRK.T | Cytochrome c | BCP & HP |
| K.TGPNLHGLFGR.K | Cytochrome c | BCP & HP |
| K.LDKIKECSEKVELIHGKKAGLAADKKEFKPLPGR.T | Cytoskeleton-associated protein 5 | BCP & HP |
| R.LACMEEFQKAVELM*DR.T | Cytoskeleton-associated protein 5 | BCP & HP |
| R.GNNVEKPLELR.I | Desmoglein-2 precursor | BCP & HP |
| K.IARQESTSVLLQQSEK.K | Desmoglein-2 precursor | BCP & HP |
| K.VKVKNVKEGIHFKSSVISIYVSESM*DR.S | Desmoglein-2 precursor | BCP & HP |
| K.IHSDLAEER.G | Desmoglein-2 precursor | BCP & HP |
| K.GNVVDWQNDFWNIPNLALK.A | Developmentally regulated G-protein-coupled receptor beta 1 | BCP & HP |
| K.VILPQTSDAYQVSVAK.S | Developmentally regulated G-protein-coupled receptor beta 1 | BCP & HP |
| R.NYETVPCDSTISK.V | Developmentally regulated G-protein-coupled receptor beta 1 | BCP & HP |
| R.LLIQAEQTSDGPNPCDMVK.Q | Dihydropyridine receptor alpha 2 subunit | BCP & HP |
| R.VLLDAGFTNELVQNYWSK.Q | Dihydropyridine receptor alpha 2 subunit | BCP & HP |
| R.KTPNNPSCNADLINR.V | Dihydropyridine receptor alpha 2 subunit | BCP & HP |
| R.IKPVFIEDANFGR.Q | Dihydropyridine receptor alpha 2 subunit | BCP & HP |
| K.SWVDKMQEDLVTLAK.T | Dihydropyridine receptor alpha 2 subunit | BCP & HP |
| K.SYDYQSVCEPGAAPK.Q | Dihydropyridine receptor alpha 2 subunit | BCP & HP |
| R.VVLKGDVSLKDIIDPAFR.A | Di-N-acetylchitobiase precursor | BCP & HP |
| R.GIGMWNANCLDYSGDAVAK.Q | Di-N-acetylchitobiase precursor | BCP & HP |
| R.APYYNYKDPAGHFHQVWYDNPQSISLK.A | Di-N-acetylchitobiase precursor | BCP & HP |
| K.TQYM*DGINIDIEQEVNCLSPEYDALTALVK.E | Di-N-acetylchitobiase precursor | BCP & HP |
| K.SYDWSQITTVATFGK.Y | Di-N-acetylchitobiase precursor | BCP & HP |
| K.QQTEEMWEVLKPK.L | Di-N-acetylchitobiase precursor | BCP & HP |
| K.GDVSLKDIIDPAFR.A | Di-N-acetylchitobiase precursor | BCP & HP |
| K.ATYIQNYR.L | Di-N-acetylchitobiase precursor | BCP & HP |
| K.FLWECVTSRRIPGSHGCIM*ADEM*GLGK.T | DNA repair and recombination protein RAD54-like | BCP & HP |
| R.DEVLQAAWDAASTAITFVFHQR.S | DNA repair and recombination protein RAD54-like | BCP & HP |
| K.VISTLEEPTPQCPTSQGR.S | Dopamine beta-hydroxylase | BCP & HP |
| K.GNEALVHHMEVFQCAPEMDSVPHFSGPCDSK.M | Dopamine beta-hydroxylase | BCP & HP |
| R.FNNEDVCTCPQASVSQQFTSVPWNSFNR.D | Dopamine beta-hydroxylase | BCP & HP |
| K.DYLIEDGTVHLVYGILEEPFR.S | Dopamine beta-hydroxylase | BCP & HP |
| K.GQIHLDPQQDYQLLQVQR.T | Dopamine beta-hydroxylase | BCP & HP |
| K.SAVDAGFLQK.Y | Dopamine beta-hydroxylase | BCP & HP |
| K.YFHLINR.F | Dopamine beta-hydroxylase | BCP & HP |
| R.FQGEWNLQPLPK.V | Dopamine beta-hydroxylase | BCP & HP |
| R.KVVTVLVR.D | Dopamine beta-hydroxylase | BCP & HP |
| R.SPAGPTVVSIGGGK.G | Dopamine beta-hydroxylase | BCP & HP |
| R.SPAGPTVVSIGGGKG.- | Dopamine beta-hydroxylase | BCP & HP |
| R.TPEGLTLLFK.R | Dopamine beta-hydroxylase | BCP & HP |
| R.VQLLKPNIPEPELPSDACTMEVQAPNIQIPSQETTYWCYIK.E | Dopamine beta-hydroxylase | BCP & HP |
| R.HVLAAWALGAK.A | Dopamine beta-hydroxylase | BCP & HP |
| K.AFYYPEEAGLAFGGPGSSR.Y | Dopamine beta-hydroxylase | BCP & HP |
| K.IGDKEVEYHPK.F | dynein, axonemal, heavy chain 17 | BCP & HP |
| K.IMTICTIDVHARDVVAKMIVESSQAFTWQAQLRHRWDEEKR.H | dynein, axonemal, heavy chain 17 | BCP & HP |
| K.QISQLNVLITLLIGNLNAGDR.M | dynein, axonemal, heavy chain 17 | BCP & HP |
| R.SESNSSITTTQPTIAKGR.T | Dystonin | BCP & HP |
| K.LQKAQEESSAM*M*QWLQKM*NK.T | Dystonin | BCP & HP |
| K.LGCSLNQNSVPDIHGVEAPAR.E | Dystroglycan precursor | BCP & HP |
| R.VTIPTDLIASSGDIIK.V | Dystroglycan precursor | BCP & HP |
| R.TASPDPGEVVSSACAADEPVTVLTVILDADLTK.M | Dystroglycan precursor | BCP & HP |
| K.KVVENGALLSWK.L | Dystroglycan precursor | BCP & HP |
| K.TTPPMLDSNGSFFLYSK.L | ELK2, member of ETS oncogene family, pseudogene 1 | BCP & HP |
| K.GFYPSDIAVEWESNGQPENNYK.T | ELK2, member of ETS oncogene family, pseudogene 1 | BCP & HP |
| K.WYVDGVEVHNAK.T | ELK2, member of ETS oncogene family, pseudogene 1 | BCP & HP |
| R.TPEVTCVVVDVSHED.P | ELK2, member of ETS oncogene family, pseudogene 1 | BCP & HP |
| K.FNWYVDGVEVHNAK.T | ELK2, member of ETS oncogene family, pseudogene 1 | BCP & HP |
| K.DISTNYYASQK.K | Endoplasmin precursor | BCP & HP |
| K.GVVDSDDLPLNVSR.E | Endoplasmin precursor | BCP & HP |
| K.LGVIEDHSNR.T | Endoplasmin precursor | BCP & HP |
| A.DDEVDVDGTVEEDLGK.S | Endoplasmin precursor | BCP & HP |
| K.TVWDWELMNDIKPIWQRPSK.E | Endoplasmin precursor | BCP & HP |
| R.EAVEKEFEPLLNWMK.D | Endoplasmin precursor | BCP & HP |
| R.RVFITDDFHDMMPK.Y | Endoplasmin precursor | BCP & HP |
| R.FQSSHHPTDITSLDQYVER.M | Endoplasmin precursor | BCP & HP |
| R.LISLTDENALSGNEELTVK.I | Endoplasmin precursor | BCP & HP |
| R.LHMLQISYFR.D | Endothelial protein C receptor precursor | BCP & HP |
| R.APGEGPQVACTGPPSAPR.N | Ephrin type-A receptor 1 precursor | BCP & HP |
| K.IDTIAADESFTQVDIGDR.I | Ephrin type-A receptor 4 precursor | BCP & HP |
| K.EVNVSPCPTQPCQLSK.G | Epididymal secretory protein E1 precursor | BCP & HP |
| K.AVVHGILMGVPVPFPIPEPDGCK.S | Epididymal secretory protein E1 precursor | BCP & HP |
| K.VFYSITGQGADTPPVGVFIIER.E | Epithelial cadherin precursor | BCP & HP |
| K.GQVPENEANVVITTLK.V | Epithelial cadherin precursor | BCP & HP |
| R.DWVIPPISCPENEKGPFPK.N | Epithelial cadherin precursor | BCP & HP |
| R.YSNIMISQFGFPYANYK.K | EXTL2 protein (Fragment) | BCP & HP |
| K.LVNIYDSMPLR.Y | EXTL2 protein (Fragment) | BCP & HP |
| R.FSCFQEEAPQPHYQLR.A | Extracellular matrix protein 1 precursor | BCP & HP |
| R.FCEAEFSVK.T | Extracellular matrix protein 1 precursor | BCP & HP |
| K.AWEDTLDKYCDR.E | Extracellular matrix protein 1 precursor | BCP & HP |
| K.AWEDTLDKYCDREYAVK.T | Extracellular matrix protein 1 precursor | BCP & HP |
| K.ELPSLQHPNEQK.E | Extracellular matrix protein 1 precursor | BCP & HP |
| K.EVGPPLPQEAVPLQK.E | Extracellular matrix protein 1 precursor | BCP & HP |
| K.LLPAQLPAEK.E | Extracellular matrix protein 1 precursor | BCP & HP |
| K.LLPAQLPAEKEVGPPLPQEAVPLQK.E | Extracellular matrix protein 1 precursor | BCP & HP |
| K.LVWEEAMSR.F | Extracellular matrix protein 1 precursor | BCP & HP |
| K.YCDREYAVK.T | Extracellular matrix protein 1 precursor | BCP & HP |
| R.ACPSHQPDISSGLELPFPPGVPTLDNIK.N | Extracellular matrix protein 1 precursor | BCP & HP |
| R.APYPNYDRDILTIDIGR.V | Extracellular matrix protein 1 precursor | BCP & HP |
| R.CCDLPFPEQACCAEEEKLTFINDLCGPR.R | Extracellular matrix protein 1 precursor | BCP & HP |
| R.LDGFPPGRPSPDNLNQICLPNR.Q | Extracellular matrix protein 1 precursor | BCP & HP |
| R.ELLALIQLER.E | Extracellular matrix protein 1 precursor | BCP & HP |
| R.QLRPEHFQEVGYAAPPSPPLSR.S | Extracellular matrix protein 1 precursor | BCP & HP |
| R.VTPNLM*GHLCGNQR.V | Extracellular matrix protein 1 precursor | BCP & HP |
| R.SQGGWGHRLDGFPPGRPSPDNLNQICLPNR.Q | Extracellular matrix protein 1 precursor | BCP & HP |
| R.SLPMDHPDSSQHGPPFEGQSQVQPPPSQEATPLQQEK.L | Extracellular matrix protein 1 precursor | BCP & HP |
| R.SLPM*DHPDSSQHGPPFEGQSQVQPPPSQEATPLQQEK.L | Extracellular matrix protein 1 precursor | BCP & HP |
| R.DPALCCYLSPGDEQVNCFNINYLR.N | Extracellular matrix protein 1 precursor | BCP & HP |
| R.RAPYPNYDRDILTIDIGR.V | Extracellular matrix protein 1 precursor | BCP & HP |
| R.VTPNLMGHLCGNQR.V | Extracellular matrix protein 1 precursor | BCP & HP |
| R.QHVVYGPWNLPQSSYSHLTR.Q | Extracellular matrix protein 1 precursor | BCP & HP |
| R.QGETLNFLEIGYSR.C | Extracellular matrix protein 1 precursor | BCP & HP |
| R.NVALVSGDTENAK.G | Extracellular matrix protein 1 precursor | BCP & HP |
| R.NLPATDPLQR.E | Extracellular matrix protein 1 precursor | BCP & HP |
| R.NIWRDPALCCYLSPGDEQVNCFNINYLR.N | Extracellular matrix protein 1 precursor | BCP & HP |
| R.HPPSPTRDECFAR.R | Extracellular matrix protein 1 precursor | BCP & HP |
| R.RNIWRDPALCCYLSPGDEQVNCFNINYLR.N | Extracellular matrix protein 1 precursor | BCP & HP |
| R.AGLAASLAGPHSIVGR.A | Extracellular superoxide dismutase [Cu-Zn] precursor | BCP & HP |
| R.AVVVHAGEDDLGRGGNQASVENGNAGR.R | Extracellular superoxide dismutase [Cu-Zn] precursor | BCP & HP |
| R.AVVVHAGEDDLGR.G | Extracellular superoxide dismutase [Cu-Zn] precursor | BCP & HP |
| R.LACCVVGVCGPGLWER.Q | Extracellular superoxide dismutase [Cu-Zn] precursor | BCP & HP |
| K.VTEIWQEVMQR.R | Extracellular superoxide dismutase [Cu-Zn] precursor | BCP & HP |
| K.GTAVTVSSASTK.- | F5-20 (Fragment) | BCP & HP |
| R.ADDTAVYYCAR.A | F5-20 (Fragment) | BCP & HP |
| R.HNAEIAAFHLDR.I | family with sequence similarity 20, member C | BCP & HP |
| K.AAENPDWPHAGAEGAEFLSPGEAAVDSYPNWLK.F | family with sequence similarity 20, member C | BCP & HP |
| R.HNPAIEALLHDLSSQR.I | family with sequence similarity 20, member C | BCP & HP |
| K.VTYLQNGK.D | Fc-gamma receptor IIIb | BCP & HP |
| K.VTYLQNGKDR.K | Fc-gamma receptor IIIb | BCP & HP |
| K.ATLKDSGSYFCR.G | Fc-gamma receptor IIIb | BCP & HP |
| R.WVFKEEDPIHLR.C | Fc-gamma receptor IIIb | BCP & HP |
| K.DSGSYFCR.G | Fc-gamma receptor IIIb | BCP & HP |
| K.AVVFLEPQWYSVLEK.D | Fc-gamma receptor IIIb | BCP & HP |
| K.AVVFLEPQWYSVLEKDSVTLK.C | Fc-gamma receptor IIIb | BCP & HP |
| R.IFFESVYGQCK.A | Fetuin-B precursor | BCP & HP |
| K.LVVLPFPK.E | Fetuin-B precursor | BCP & HP |
| R.KDGYVLR.L | Fetuin-B precursor | BCP & HP |
| K.IYM*TCPDCPSSIPTDSSNHQVLEAATESLAK.Y | Fetuin-B precursor | BCP & HP |
| K.KAWQDCGMR.I | Fetuin-B precursor | BCP & HP |
| A.M*SPPQLALNPSALLSR.G | Fetuin-B precursor | BCP & HP |
| A.MSPPQLALNPSALLSR.G | Fetuin-B precursor | BCP & HP |
| R.VNDAQEYR.R | Fetuin-B precursor | BCP & HP |
| R.GGLGSLFYLTLDVLETDCHVLR.K | Fetuin-B precursor | BCP & HP |
| R.LNRVNDAQEYR.R | Fetuin-B precursor | BCP & HP |
| K.KIYMTCPDCPSSIPTDSSNHQVLEAATESLAK.Y | Fetuin-B precursor | BCP & HP |
| K.KKIYMTCPDCPSSIPTDSSNHQVLEAATESLAK.Y | Fetuin-B precursor | BCP & HP |
| K.IYMTCPDCPSSIPTDSSNHQVLEAATESLAK.Y | Fetuin-B precursor | BCP & HP |
| R.GSVQYLPDLDDKNSQEK.G | Fetuin-B precursor | BCP & HP |
| R.ASSQWVVGPSYFVEYLIK.E | Fetuin-B precursor | BCP & HP |
| K.AIFYM*NNPSR.V | Fetuin-B precursor | BCP & HP |
| K.YNNENTSKQYSLFK.V | Fetuin-B precursor | BCP & HP |
| K.SQASSCSLQSSDSVPVGLCK.G | Fetuin-B precursor | BCP & HP |
| K.AIFYMNNPSR.V | Fetuin-B precursor | BCP & HP |
| R.TAECPGPAQNASPLVLPP.- | Fetuin-B precursor | BCP & HP |
| K.NNKDSHSLTTNIM*EILR.G | FGA protein | BCP & HP |
| A.DSGEGDFLAEGGGVR.G | FGA protein | BCP & HP |
| D.SGEGDFLAEGGGVR.G | FGA protein | BCP & HP |
| K.DSHSLTTNIM*EILR.G | FGA protein | BCP & HP |
| K.DSHSLTTNIMEILR.G | FGA protein | BCP & HP |
| K.GLIDEVNQDFTNR.I | FGA protein | BCP & HP |
| K.GLIDEVNQDFTNRINK.L | FGA protein | BCP & HP |
| K.NNKDSHSLTTNIMEILR.G | FGA protein | BCP & HP |
| R.MKGLIDEVNQDFTNR.I | FGA protein | BCP & HP |
| K.LKNSLFEYQK.N | FGA protein | BCP & HP |
| T.ADSGEGDFLAEGGGVR.G | FGA protein | BCP & HP |
| R.MKGLIDEVNQDFTNRINK.L | FGA protein | BCP & HP |
| R.M*KGLIDEVNQDFTNRINK.L | FGA protein | BCP & HP |
| R.M*KGLIDEVNQDFTNR.I | FGA protein | BCP & HP |
| R.GDFSSANNRDNTYNR.V | FGA protein | BCP & HP |
| R.GDFSSANNR.D | FGA protein | BCP & HP |
| K.VIEKVTANNLLVAR.V | FGA protein | BCP & HP |
| K.NSLFEYQKNNKDSHSLTTNIMEILR.G | FGA protein | BCP & HP |
| K.NSLFEYQK.N | FGA protein | BCP & HP |
| S.GEGDFLAEGGGVR.G | FGA protein | BCP & HP |
| K.CQCPSGM*TLDATGR.I | Fibrillin-1 precursor | BCP & HP |
| K.ILCPGGEGFRPNPITVILEDIDECQELPGLCQGGK.C | Fibrillin-1 precursor | BCP & HP |
| K.CLCPEGFSLSSSGR.R | Fibrillin-1 precursor | BCP & HP |
| K.NCEDM*DECSIR.N | Fibrillin-1 precursor | BCP & HP |
| K.YQCACNPGYHSTPDR.L | Fibrillin-1 precursor | BCP & HP |
| R.CECEIGYELDR.S | Fibrillin-1 precursor | BCP & HP |
| R.GDNGDTACSNEIGVGVSK.A | Fibrillin-1 precursor | BCP & HP |
| K.CICNSGYEVDSTGK.N | Fibrillin-1 precursor | BCP & HP |
| R.IGQGHCVSGM*GM*GR.G | Fibrillin-1 precursor | BCP & HP |
| R.YYWGGQYTWDM*AK.H | Fibrinogen beta chain precursor | BCP & HP |
| R.TMTIHNGM*FFSTYDRDNDGWLTSDPR.K | Fibrinogen beta chain precursor | BCP & HP |
| R.TMTIHNGMFFSTYDRDNDGWLTSDPRK.Q | Fibrinogen beta chain precursor | BCP & HP |
| R.TMTIHNGM*FFSTYDRDNDGWLTSDPRK.Q | Fibrinogen beta chain precursor | BCP & HP |
| R.TMTIHNGMFFSTYDR.D | Fibrinogen beta chain precursor | BCP & HP |
| R.TMTIHNGM*FFSTYDR.D | Fibrinogen beta chain precursor | BCP & HP |
| R.TMTIHNGMFFSTYDRDNDGWLTSDPR.K | Fibrinogen beta chain precursor | BCP & HP |
| R.TPCTVSCNIPVVSGK.E | Fibrinogen beta chain precursor | BCP & HP |
| R.TPCTVSCNIPVVSGKECEEIIR.K | Fibrinogen beta chain precursor | BCP & HP |
| R.TPCTVSCNIPVVSGKECEEIIRK.G | Fibrinogen beta chain precursor | BCP & HP |
| R.VYCDMNTENGGWTVIQNR.Q | Fibrinogen beta chain precursor | BCP & HP |
| R.YYWGGQYTWDMAK.H | Fibrinogen beta chain precursor | BCP & HP |
| R.SKIQKLESDVSAQMEYCR.T | Fibrinogen beta chain precursor | BCP & HP |
| R.TM*TIHNGMFFSTYDRDNDGWLTSDPRK.Q | Fibrinogen beta chain precursor | BCP & HP |
| R.VYCDM*NTENGGWTVIQNR.Q | Fibrinogen beta chain precursor | BCP & HP |
| K.QVKDNENVVNEYSSELEK.H | Fibrinogen beta chain precursor | BCP & HP |
| K.KREEAPSLRPAPPPISGGGYR.A | Fibrinogen beta chain precursor | BCP & HP |
| K.LESDVSAQM*EYCR.T | Fibrinogen beta chain precursor | BCP & HP |
| K.LESDVSAQMEYCR.T | Fibrinogen beta chain precursor | BCP & HP |
| K.NYCGLPGEYWLGNDK.I | Fibrinogen beta chain precursor | BCP & HP |
| K.NYCGLPGEYWLGNDKISQLTR.M | Fibrinogen beta chain precursor | BCP & HP |
| K.QCSKEDGGGWWYNR.C | Fibrinogen beta chain precursor | BCP & HP |
| K.QGFGNVATNTDGK.N | Fibrinogen beta chain precursor | BCP & HP |
| Q.PDSSVKPYR.V | Fibrinogen beta chain precursor | BCP & HP |
| K.QGFGNVATNTDGKNYCGLPGEYWLGNDKISQLTR.M | Fibrinogen beta chain precursor | BCP & HP |
| K.HQLYIDETVNSNIPTNLR.V | Fibrinogen beta chain precursor | BCP & HP |
| K.QVKDNENVVNEYSSELEKHQLYIDETVNSNIPTNLR.V | Fibrinogen beta chain precursor | BCP & HP |
| K.REEAPSLRPAPPPISGGGYR.A | Fibrinogen beta chain precursor | BCP & HP |
| K.WDPYKQGFGNVATNTDGK.N | Fibrinogen beta chain precursor | BCP & HP |
| K.WDPYKQGFGNVATNTDGKNYCGLPGEYWLGNDK.I | Fibrinogen beta chain precursor | BCP & HP |
| K.WDPYKQGFGNVATNTDGKNYCGLPGEYWLGNDKISQLTR.M | Fibrinogen beta chain precursor | BCP & HP |
| K.YQISVNK.Y | Fibrinogen beta chain precursor | BCP & HP |
| K.YQISVNKYR.G | Fibrinogen beta chain precursor | BCP & HP |
| R.TM*TIHNGMFFSTYDR.D | Fibrinogen beta chain precursor | BCP & HP |
| K.QGFGNVATNTDGKNYCGLPGEYWLGNDK.I | Fibrinogen beta chain precursor | BCP & HP |
| K.ECEEIIRK.G | Fibrinogen beta chain precursor | BCP & HP |
| F.TVQNEANKYQISVNK.Y | Fibrinogen beta chain precursor | BCP & HP |
| G.VNDNEEGFFSAR.G | Fibrinogen beta chain precursor | BCP & HP |
| I.PVVSGKECEEIIR.K | Fibrinogen beta chain precursor | BCP & HP |
| I.PVVSGKECEEIIRK.G | Fibrinogen beta chain precursor | BCP & HP |
| K.AHYGGFTVQNEANK.Y | Fibrinogen beta chain precursor | BCP & HP |
| K.AHYGGFTVQNEANKYQISVNK.Y | Fibrinogen beta chain precursor | BCP & HP |
| K.APDAGGCLHADPDLGVLCPTGCQLQEALLQQERPIR.N | Fibrinogen beta chain precursor | BCP & HP |
| K.DNENVVNEYSSELEK.H | Fibrinogen beta chain precursor | BCP & HP |
| K.IQKLESDVSAQMEYCR.T | Fibrinogen beta chain precursor | BCP & HP |
| K.ECEEIIR.K | Fibrinogen beta chain precursor | BCP & HP |
| K.IQKLESDVSAQM*EYCR.T | Fibrinogen beta chain precursor | BCP & HP |
| K.EDGGGWWYNR.C | Fibrinogen beta chain precursor | BCP & HP |
| K.GGETSEM*YLIQPDSSVKPYR.V | Fibrinogen beta chain precursor | BCP & HP |
| K.GGETSEMYLIQPDSSVKPYR.V | Fibrinogen beta chain precursor | BCP & HP |
| K.GSWYSM*R.K | Fibrinogen beta chain precursor | BCP & HP |
| K.GSWYSMR.K | Fibrinogen beta chain precursor | BCP & HP |
| K.HGTDDGVVWM*NWK.G | Fibrinogen beta chain precursor | BCP & HP |
| K.HGTDDGVVWMNWK.G | Fibrinogen beta chain precursor | BCP & HP |
| R.DNDGWLTSDPR.K | Fibrinogen beta chain precursor | BCP & HP |
| K.DNENVVNEYSSELEKHQLYIDETVNSNIPTNLR.V | Fibrinogen beta chain precursor | BCP & HP |
| R.NSVDELNNNVEAVSQTSSSSFQYM*YLLK.D | Fibrinogen beta chain precursor | BCP & HP |
| R.M*GPTELLIEMEDWKGDK.V | Fibrinogen beta chain precursor | BCP & HP |
| R.TM*TIHNGMFFSTYDRDNDGWLTSDPR.K | Fibrinogen beta chain precursor | BCP & HP |
| R.MGPTELLIEM*EDWK.G | Fibrinogen beta chain precursor | BCP & HP |
| L.LIEMEDWKGDKVK.A | Fibrinogen beta chain precursor | BCP & HP |
| R.MGPTELLIEM*EDWKGDKVK.A | Fibrinogen beta chain precursor | BCP & HP |
| R.MGPTELLIEMEDWK.G | Fibrinogen beta chain precursor | BCP & HP |
| R.M*GPTELLIEMEDWK.G | Fibrinogen beta chain precursor | BCP & HP |
| R.MGPTELLIEMEDWKGDKVK.A | Fibrinogen beta chain precursor | BCP & HP |
| R.M*GPTELLIEMEDWKGDKVK.A | Fibrinogen beta chain precursor | BCP & HP |
| R.NSVDELNNNVEAVSQTSSSSFQYMYLLK.D | Fibrinogen beta chain precursor | BCP & HP |
| R.QDGSVDFGR.K | Fibrinogen beta chain precursor | BCP & HP |
| R.QDGSVDFGRK.W | Fibrinogen beta chain precursor | BCP & HP |
| R.QKQVKDNENVVNEYSSELEK.H | Fibrinogen beta chain precursor | BCP & HP |
| R.QKQVKDNENVVNEYSSELEKHQLYIDETVNSNIPTNLR.V | Fibrinogen beta chain precursor | BCP & HP |
| R.SKIQKLESDVSAQM*EYCR.T | Fibrinogen beta chain precursor | BCP & HP |
| R.TM*TIHNGM*FFSTYDR.D | Fibrinogen beta chain precursor | BCP & HP |
| R.MGPTELLIEMEDWKGDK.V | Fibrinogen beta chain precursor | BCP & HP |
| R.KGGETSEMYLIQPDSSVKPYR.V | Fibrinogen beta chain precursor | BCP & HP |
| R.DNDGWLTSDPRK.Q | Fibrinogen beta chain precursor | BCP & HP |
| R.EEAPSLRPAPPPISGGGYR.A | Fibrinogen beta chain precursor | BCP & HP |
| R.KAPDAGGCLHADPDLGVLCPTGCQLQEALLQQERPIR.N | Fibrinogen beta chain precursor | BCP & HP |
| R.MGPTELLIEM*EDWKGDK.V | Fibrinogen beta chain precursor | BCP & HP |
| R.KGGETSEM*YLIQPDSSVKPYR.V | Fibrinogen beta chain precursor | BCP & HP |
| R.M*GPTELLIEM*EDWK.G | Fibrinogen beta chain precursor | BCP & HP |
| R.KQCSKEDGGGWWYNR.C | Fibrinogen beta chain precursor | BCP & HP |
| R.KWDPYKQGFGNVATNTDGKNYCGLPGEYWLGNDKISQLTR.M | Fibrinogen beta chain precursor | BCP & HP |
| R.KWDPYKQGFGNVATNTDGKNYCGLPGEYWLGNDK.I | Fibrinogen beta chain precursor | BCP & HP |
| R.KWDPYK.Q | Fibrinogen beta chain precursor | BCP & HP |
| R.KWDPYKQGFGNVATNTDGK.N | Fibrinogen beta chain precursor | BCP & HP |
| R.CHSANLNGVYYSGPYTAK.T | Fibrinogen-like protein 1 precursor | BCP & HP |
| K.IKPLQSPAEFSVYCDMSDGGGWTVIQR.R | Fibrinogen-like protein 1 precursor | BCP & HP |
| K.NLHFLTTQEDYTLK.I | Fibrinogen-like protein 1 precursor | BCP & HP |
| R.IEEVFKEVQNLK.E | Fibroleukin precursor | BCP & HP |
| R.AKDVCPVR.L | Fibroleukin precursor | BCP & HP |
| R.DM*DVVSGR.S | Fibulin-5 precursor | BCP & HP |
| R.CM*CPAENPGCR.D | Fibulin-5 precursor | BCP & HP |
| K.VPVHDVTDASK.V | filamin A, alpha isoform 1 | BCP & HP |
| K.AFGPGLQGGSAGSPAR.F | filamin A, alpha isoform 1 | BCP & HP |
| R.ANLPQSFQVDTSK.A | filamin A, alpha isoform 1 | BCP & HP |
| R.ALTQTGGPHVK.A | filamin A, alpha isoform 1 | BCP & HP |
| K.ATCAPQHGAPGPGPADASK.V | filamin A, alpha isoform 1 | BCP & HP |
| K.DGSCGVAYVVQEPGDYEVSVK.F | filamin A, alpha isoform 1 | BCP & HP |
| K.DNGNGTYSCSYVPR.K | filamin A, alpha isoform 1 | BCP & HP |
| K.VGSAADIPINISETDLSLLTATVVPPSGR.E | filamin A, alpha isoform 1 | BCP & HP |
| K.VTAQGPGLEPSGNIANK.T | filamin A, alpha isoform 1 | BCP & HP |
| R.AGQSAAGAAPGGGVDTR.D | filamin A, alpha isoform 1 | BCP & HP |
| R.ALGALVDSCAPGLCPDWDSWDASKPVTNAR.E | filamin A, alpha isoform 1 | BCP & HP |
| R.EATTEFSVDAR.A | filamin A, alpha isoform 1 | BCP & HP |
| R.EGPYSISVLYGDEEVPR.S | filamin A, alpha isoform 1 | BCP & HP |
| K.GLVEPVDVVDNADGTQTVNYVPSR.E | filamin A, alpha isoform 1 | BCP & HP |
| R.SPYTVTVGQACNPSACR.A | filamin A, alpha isoform 1 | BCP & HP |
| R.VANPSGNLTETYVQDR.G | filamin A, alpha isoform 1 | BCP & HP |
| R.VQVQDNEGCPVEALVK.D | filamin A, alpha isoform 1 | BCP & HP |
| K.SKICANVFCGAGR.E | Follistatin-related protein 1 precursor | BCP & HP |
| K.GAQTQTEEEM*TR.Y | Follistatin-related protein 1 precursor | BCP & HP |
| K.CLNPSFNPPEKK.C | Follistatin-related protein 1 precursor | BCP & HP |
| K.CALEDETYADGAETEVDCNR.C | Follistatin-related protein 1 precursor | BCP & HP |
| K.GAQTQTEEEMTR.Y | Follistatin-related protein 1 precursor | BCP & HP |
| K.AAQEEYVKR.A | Fructose-bisphosphate aldolase A | BCP & HP |
| K.ELSDIAHR.I | Fructose-bisphosphate aldolase A | BCP & HP |
| K.GVVPLAGTNGETTTQGLDGLSER.C | Fructose-bisphosphate aldolase A | BCP & HP |
| K.IGEHTPSALAIMENANVLAR.Y | Fructose-bisphosphate aldolase A | BCP & HP |
| K.YTPSGQAGAAASESLFVSNHAY.- | Fructose-bisphosphate aldolase A | BCP & HP |
| M.PYQYPALTPEQK.K | Fructose-bisphosphate aldolase A | BCP & HP |
| R.YASICQQNGIVPIVEPEILPDGDHDLKR.C | Fructose-bisphosphate aldolase A | BCP & HP |
| K.GILAADESTGSIAK.R | Fructose-bisphosphate aldolase A | BCP & HP |
| K.KYTPEQVAMATVTALHR.T | Fructose-bisphosphate aldolase B | BCP & HP |
| K.AANKEATQEAFM*KR.A | Fructose-bisphosphate aldolase B | BCP & HP |
| K.AANKEATQEAFMK.R | Fructose-bisphosphate aldolase B | BCP & HP |
| K.AANKEATQEAFMKR.A | Fructose-bisphosphate aldolase B | BCP & HP |
| K.ELSEIAQSIVANGK.G | Fructose-bisphosphate aldolase B | BCP & HP |
| K.ETTIQGLDGLSER.C | Fructose-bisphosphate aldolase B | BCP & HP |
| K.GILAADESVGTMGNR.L | Fructose-bisphosphate aldolase B | BCP & HP |
| K.LDQGGAPLAGTNK.E | Fructose-bisphosphate aldolase B | BCP & HP |
| K.LDQGGAPLAGTNKETTIQGLDGLSER.C | Fructose-bisphosphate aldolase B | BCP & HP |
| R.ALQASALAAWGGK.A | Fructose-bisphosphate aldolase B | BCP & HP |
| R.IADQCPSSLAIQENANALAR.Y | Fructose-bisphosphate aldolase B | BCP & HP |
| K.GILAADESVGTM*GNR.L | Fructose-bisphosphate aldolase B | BCP & HP |
| K.GEGM*SQAATICK.S | Fumarylacetoacetase | BCP & HP |
| K.SFGTTVSPWVVPMDALMPFAVPNPK.Q | Fumarylacetoacetase | BCP & HP |
| R.VFLQNLLSVSQAR.L | Fumarylacetoacetase | BCP & HP |
| K.HQDVFNQPTLNSFMGLGQAAWK.E | Fumarylacetoacetase | BCP & HP |
| R.LADGGATNQGR.V | Galectin-3-binding protein precursor | BCP & HP |
| R.IYTSPTWSAFVTDSSWSAR.K | Galectin-3-binding protein precursor | BCP & HP |
| R.AAFGQGSGPIM*LDEVQCTGTEASLADCK.S | Galectin-3-binding protein precursor | BCP & HP |
| R.GQWGTVCDNLWDLTDASVVCR.A | Galectin-3-binding protein precursor | BCP & HP |
| R.ELSEALGQIFDSQR.G | Galectin-3-binding protein precursor | BCP & HP |
| R.STHTLDLSR.E | Galectin-3-binding protein precursor | BCP & HP |
| R.SDLAVPSELALLK.A | Galectin-3-binding protein precursor | BCP & HP |
| R.RIDITLSSVK.C | Galectin-3-binding protein precursor | BCP & HP |
| R.ASHEEVEGLVEK.I | Galectin-3-binding protein precursor | BCP & HP |
| R.AAFGQGSGPIMLDEVQCTGTEASLADCK.S | Galectin-3-binding protein precursor | BCP & HP |
| K.LASAYGAR.Q | Galectin-3-binding protein precursor | BCP & HP |
| K.SQLVYQSR.R | Galectin-3-binding protein precursor | BCP & HP |
| K.TLQALEFHTVPFQLLAR.Y | Galectin-3-binding protein precursor | BCP & HP |
| K.YSSDYFQAPSDYR.Y | Galectin-3-binding protein precursor | BCP & HP |
| R.KSQLVYQSR.R | Galectin-3-binding protein precursor | BCP & HP |
| K.HFVALSTNTTK.V | Glucose-6-phosphate isomerase | BCP & HP |
| K.ILLANFLAQTEALMR.G | Glucose-6-phosphate isomerase | BCP & HP |
| R.ASLIDDAFALAR.A | Glutamyl aminopeptidase | BCP & HP |
| R.PGGGFVPNFQLFEK.G | Glutathione peroxidase 3 precursor | BCP & HP |
| K.YVRPGGGFVPNFQLFEKGDVNGEKEQK.F | Glutathione peroxidase 3 precursor | BCP & HP |
| R.WNFEKFLVGPDGIPIMR.W | Glutathione peroxidase 3 precursor | BCP & HP |
| R.LFWEPMKVHDIR.W | Glutathione peroxidase 3 precursor | BCP & HP |
| R.LFWEPM*KVHDIR.W | Glutathione peroxidase 3 precursor | BCP & HP |
| K.YVRPGGGFVPNFQLFEKGDVNGEK.E | Glutathione peroxidase 3 precursor | BCP & HP |
| K.YVRPGGGFVPNFQLFEK.G | Glutathione peroxidase 3 precursor | BCP & HP |
| K.QEPGENSEILPTLK.Y | Glutathione peroxidase 3 precursor | BCP & HP |
| K.NSCPPTSELLGTSDR.L | Glutathione peroxidase 3 precursor | BCP & HP |
| K.MDILSYMR.R | Glutathione peroxidase 3 precursor | BCP & HP |
| K.MDILSYM*R.R | Glutathione peroxidase 3 precursor | BCP & HP |
| K.M*DILSYMR.R | Glutathione peroxidase 3 precursor | BCP & HP |
| K.FLVGPDGIPIM*R.W | Glutathione peroxidase 3 precursor | BCP & HP |
| K.FLVGPDGIPIMR.W | Glutathione peroxidase 3 precursor | BCP & HP |
| K.GSAPPGPVPEGSIR.I | Glutathione transferase omega-1 | BCP & HP |
| K.LNECVDHTPK.L | Glutathione transferase omega-1 | BCP & HP |
| R.GALQNIIPASTGAAK.A | Glyceraldehyde-3-phosphate dehydrogenase | BCP & HP |
| R.VPTANVSVVDLTCR.L | Glyceraldehyde-3-phosphate dehydrogenase | BCP & HP |
| K.IISNASCTTNCLAPLAK.V | Glyceraldehyde-3-phosphate dehydrogenase | BCP & HP |
| K.VIHDNFGIVEGLMTTVHAITATQK.T | Glyceraldehyde-3-phosphate dehydrogenase | BCP & HP |
| K.AVGDKLPECEADDGCPKPPEIAHGYVEHSVR.Y | Haptoglobin precursor | BCP & HP |
| K.SPVGVQPILNEHTFCAGMSK.Y | Haptoglobin precursor | BCP & HP |
| K.SPVGVQPILNEHTFCAGM*SK.Y | Haptoglobin precursor | BCP & HP |
| A.VDSGNDVTDIADDGCPKPPEIAHGYVEHSVR.Y | Haptoglobin precursor | BCP & HP |
| R.TEGDGVYTLNDKK.Q | Haptoglobin precursor | BCP & HP |
| K.SCAVAEYGVYVK.V | Haptoglobin precursor | BCP & HP |
| K.DIAPTLTLYVGKK.Q | Haptoglobin precursor | BCP & HP |
| K.DIAPTLTLYVGK.K | Haptoglobin precursor | BCP & HP |
| K.AVGDKLPECEAVCGKPKNPANPVQ.R | Haptoglobin precursor | BCP & HP |
| R.VMPICLPSKDYAEVGR.V | Haptoglobin precursor | BCP & HP |
| K.YVMLPVADQDQCIR.H | Haptoglobin precursor | BCP & HP |
| R.VGYVSGWGR.N | Haptoglobin precursor | BCP & HP |
| K.YQEDTCYGDAGSAFAVHDLEEDTWYATGILSFDK.S | Haptoglobin precursor | BCP & HP |
| R.LRTEGDGVYTLNDKK.Q | Haptoglobin precursor | BCP & HP |
| K.AVGDKLPECEAVCGKPK.N | Haptoglobin precursor | BCP & HP |
| R.ILGGHLDAK.G | Haptoglobin precursor | BCP & HP |
| R.HYEGSTVPEK.K | Haptoglobin precursor | BCP & HP |
| K.YVM*LPVADQDQCIR.H | Haptoglobin precursor | BCP & HP |
| K.NQVALNPQNTVFDAK.R | Heat shock 70 kDa protein 1 | BCP & HP |
| K.ITITNDKGR.L | Heat shock 70 kDa protein 1 | BCP & HP |
| G.YPITLFVEKECDK.E | Heat shock protein 90 kDa alpha class A member 2 (Fragment) | BCP & HP |
| K.AFMEALQAGADISMIGQFGVGFYSAYLVAEK.V | heat shock protein 90kDa alpha (cytosolic), class A member 1 isoform 1 | BCP & HP |
| K.DQVANSAFVER.L | heat shock protein 90kDa alpha (cytosolic), class A member 1 isoform 1 | BCP & HP |
| K.HSQFIGYPITLFVEKER.D | heat shock protein 90kDa alpha (cytosolic), class A member 1 isoform 1 | BCP & HP |
| K.NTLYLQMNSLR.A | Heavy chain Fab (Fragment) | BCP & HP |
| R.AEDTATYFCAK.D | Heavy chain Fab (Fragment) | BCP & HP |
| R.FFESFGDLSSPDAVM*GNPK.V | Hemoglobin delta-beta fusion protein | BCP & HP |
| K.KVLGAFSDGLAHLDNLK.G | Hemoglobin delta-beta fusion protein | BCP & HP |
| R.LLVVYPWTQR.F | Hemoglobin delta-beta fusion protein | BCP & HP |
| K.TYFPHFDLSHGSAQVK.G | Hemoglobin subunit alpha | BCP & HP |
| K.VGAHAGEYGAEALER.M | Hemoglobin subunit alpha | BCP & HP |
| K.VNVDEVGGEALGR.L | Hemoglobin subunit beta | BCP & HP |
| R.LLVVYPWTQR.F | Hemoglobin subunit beta | BCP & HP |
| R.FFESFGDLSTPDAVMGNPK.V | Hemoglobin subunit beta | BCP & HP |
| K.VVAGVANALAHK.Y | Hemoglobin subunit beta | BCP & HP |
| K.EFTPPVQAAYQK.V | Hemoglobin subunit beta | BCP & HP |
| K.KVLGAFSDGLAHLDNLK.G | Hemoglobin subunit beta | BCP & HP |
| H.LVLSALTSDNHGATYAFSGTHYWR.L | Hemopexin precursor | BCP & HP |
| D.NHGATYAFSGTHYWR.L | Hemopexin precursor | BCP & HP |
| K.SLGPNSCSANGPGLYLIHG.P | Hemopexin precursor | BCP & HP |
| A.HGNVAEGETKPDPDVTER.C | Hemopexin precursor | BCP & HP |
| C.SANGPGLYLIHGPNLYCYSDVEK.L | Hemopexin precursor | BCP & HP |
| F.PGIPSPLDAAVECHR.G | Hemopexin precursor | BCP & HP |
| F.PSPVDAAFR.Q | Hemopexin precursor | BCP & HP |
| G.NVAEGETKPDPDVTER.C | Hemopexin precursor | BCP & HP |
| G.PGLYLIHGPNLYCYSDVEKLNAAK.A | Hemopexin precursor | BCP & HP |
| G.PNLYCYSDVEKLNAAK.A | Hemopexin precursor | BCP & HP |
| H.GNVAEGETKPDPDVTER.C | Hemopexin precursor | BCP & HP |
| I.HGPNLYCYSDVEK.L | Hemopexin precursor | BCP & HP |
| I.PSPLDAAVECHR.G | Hemopexin precursor | BCP & HP |
| H.GIILDSVDAAFICPGSSR.L | Hemopexin precursor | BCP & HP |
| K.LLQDEFPGIPSPLDAAVECH.R | Hemopexin precursor | BCP & HP |
| K.SGAQATWTELPWPHEKVDGALCM*EK.S | Hemopexin precursor | BCP & HP |
| K.SGAQATWTELPWPHEK.V | Hemopexin precursor | BCP & HP |
| K.RLEKEVGTPHGIILDSVDAAFICPGSSR.L | Hemopexin precursor | BCP & HP |
| K.NFPSPVDAAFRQGHNSVFLIK.G | Hemopexin precursor | BCP & HP |
| K.NFPSPVDAAFR.Q | Hemopexin precursor | BCP & HP |
| K.LYLVQGTQVYVFLTK.G | Hemopexin precursor | BCP & HP |
| R.FDPVRGEVPPR.Y | Hemopexin precursor | BCP & HP |
| K.LLQDEFPGIPSPLDAAVECHR.G | Hemopexin precursor | BCP & HP |
| R.GECQAEGVLFFQGDREWFWDLATGTM*KER.S | Hemopexin precursor | BCP & HP |
| K.GGYTLVSGYPKR.L | Hemopexin precursor | BCP & HP |
| K.GDKVWVYPPEKK.E | Hemopexin precursor | BCP & HP |
| K.EVGTPHGIILDSVDAAFICPGSSRLHIMAGR.R | Hemopexin precursor | BCP & HP |
| K.EVGTPHGIILDSVDAAFICPGSSR.L | Hemopexin precursor | BCP & HP |
| K.ALPQPQNVTSLLGCTH.- | Hemopexin precursor | BCP & HP |
| R.GECQAEGVLFFQGDR.E | Hemopexin precursor | BCP & HP |
| R.FDPVRGEVPPRYPR.D | Hemopexin precursor | BCP & HP |
| K.LLQDEFPGIPSPLDAAVECHRGECQAEGVLFFQGDR.E | Hemopexin precursor | BCP & HP |
| R.QGHNSVFLIK.G | Hemopexin precursor | BCP & HP |
| W.PHEKVDGALCMEK.S | Hemopexin precursor | BCP & HP |
| T.SAHGNVAEGETKPDPDVTER.C | Hemopexin precursor | BCP & HP |
| S.AHGNVAEGETKPDPDVTER.C | Hemopexin precursor | BCP & HP |
| R.YYCFQGNQFLR.F | Hemopexin precursor | BCP & HP |
| R.WKNFPSPVDAAFRQGHNSVFLIK.G | Hemopexin precursor | BCP & HP |
| R.WKNFPSPVDAAFR.Q | Hemopexin precursor | BCP & HP |
| R.SWPAVGNCSSALR.W | Hemopexin precursor | BCP & HP |
| K.SGAQATWTELPWPHEKVDGALCMEK.S | Hemopexin precursor | BCP & HP |
| R.QGHNSVFLIKGDK.V | Hemopexin precursor | BCP & HP |
| R.GECQAEGVLFFQGDREWFWDLATGTM*K.E | Hemopexin precursor | BCP & HP |
| R.LWWLDLK.S | Hemopexin precursor | BCP & HP |
| R.LEKEVGTPHGIILDSVDAAFICPGSSR.L | Hemopexin precursor | BCP & HP |
| R.LDTSRDGWHSWPIAHQWPQGPSAVDAAFSWEEK.L | Hemopexin precursor | BCP & HP |
| R.GEVPPRYPR.D | Hemopexin precursor | BCP & HP |
| R.GECQAEGVLFFQGDREWFWDLATGTMKER.S | Hemopexin precursor | BCP & HP |
| R.GECQAEGVLFFQGDREWFWDLATGTMK.E | Hemopexin precursor | BCP & HP |
| K.GDKVWVYPPEK.K | Hemopexin precursor | BCP & HP |
| R.RLWWLDLK.S | Hemopexin precursor | BCP & HP |
| N.SCSANGPGLYLIHGPNLYCYSDVEK.L | Hemopexin precursor | BCP & HP |
| R.CSPHLVLSALTSDNHGATYAF.S | Hemopexin precursor | BCP & HP |
| R.CSDGWSFDATTLDDNGTMLFFK.G | Hemopexin precursor | BCP & HP |
| P.TSAHGNVAEGETKPDPDVTER.C | Hemopexin precursor | BCP & HP |
| P.PTSAHGNVAEGETKPDPDVTER.C | Hemopexin precursor | BCP & HP |
| R.EWFWDLATGTMKER.S | Hemopexin precursor | BCP & HP |
| P.GLYLIHGPNLYCYSDVEKLNAAK.A | Hemopexin precursor | BCP & HP |
| K.GGYTLVSGYPK.R | Hemopexin precursor | BCP & HP |
| R.CSPHLVLSALTSDNHGATYAFSGTHYWR.L | Hemopexin precursor | BCP & HP |
| K.SHKWDR.E | Hemopexin precursor | BCP & HP |
| P.LPPTSAHGNVAEGETKPDPDVTER.C | Hemopexin precursor | BCP & HP |
| N.HGATYAFSGTHYWR.L | Hemopexin precursor | BCP & HP |
| N.GPGLYLIHGPNLYCYSDVEK.L | Hemopexin precursor | BCP & HP |
| L.YCYSDVEKLNAAK.A | Hemopexin precursor | BCP & HP |
| L.TSDNHGATYAFSGTHYWR.L | Hemopexin precursor | BCP & HP |
| L.SALTSDNHGATYAFSGTHYWR.L | Hemopexin precursor | BCP & HP |
| L.PPTSAHGNVAEGETKPDPDVTER.C | Hemopexin precursor | BCP & HP |
| L.GPNSCSANGPGLYLIHGPNLYCYSDVEKLNAAK.A | Hemopexin precursor | BCP & HP |
| K.WDRELISER.W | Hemopexin precursor | BCP & HP |
| N.SCSANGPGLYLIHGPNLYCYSDVEKLNAAK.A | Hemopexin precursor | BCP & HP |
| K.SHKWDRELISER.W | Hemopexin precursor | BCP & HP |
| R.EWFWDLATGTMK.E | Hemopexin precursor | BCP & HP |
| P.GLYLIHGPNLYCYSDVEK.L | Hemopexin precursor | BCP & HP |
| R.EWFWDLATGTM*K.E | Hemopexin precursor | BCP & HP |
| R.DGWHSWPIAHQWPQGPSAVDAAFSWEEK.L | Hemopexin precursor | BCP & HP |
| K.SLGPNSCSANGPGLYLIHGPN.L | Hemopexin precursor | BCP & HP |
| K.SLGPNSCSANGPGLYLIHGPNLY.C | Hemopexin precursor | BCP & HP |
| K.SLGPNSCSANGPGLYLIHGPNLYCYSDVEK.L | Hemopexin precursor | BCP & HP |
| K.SLGPNSCSANGPGLYLIHGPNLYCYSDVEKLNAAK.A | Hemopexin precursor | BCP & HP |
| R.DYFM*PCPGR.G | Hemopexin precursor | BCP & HP |
| R.EWFWDLATGTM*KER.S | Hemopexin precursor | BCP & HP |
| K.VDGALCM*EK.S | Hemopexin precursor | BCP & HP |
| R.DVRDYFMPCPGR.G | Hemopexin precursor | BCP & HP |
| R.DYFMPCPGR.G | Hemopexin precursor | BCP & HP |
| R.ELISERWKNFPSPVDAAFR.Q | Hemopexin precursor | BCP & HP |
| K.VWVYPPEKK.E | Hemopexin precursor | BCP & HP |
| K.VDGALCMEK.S | Hemopexin precursor | BCP & HP |
| R.DVRDYFM*PCPGR.G | Hemopexin precursor | BCP & HP |
| K.SDACQGDSGGPLACEK.N | Hepatocyte growth factor activator precursor | BCP & HP |
| K.CFDETRYEYLEGGDR.W | Hepatocyte growth factor activator precursor | BCP & HP |
| K.CFDETRYEYLEGGDRWAR.V | Hepatocyte growth factor activator precursor | BCP & HP |
| K.CQIAGWGHLDENVSGYSSSLR.E | Hepatocyte growth factor activator precursor | BCP & HP |
| K.CSSPEVYGADISPNM*LCAGYFDCK.S | Hepatocyte growth factor activator precursor | BCP & HP |
| K.CSSPEVYGADISPNMLCAGYFDCK.S | Hepatocyte growth factor activator precursor | BCP & HP |
| R.VQLSPDLLATLPEPASPGR.Q | Hepatocyte growth factor activator precursor | BCP & HP |
| K.NGVAYLYGIISWGDGCGR.L | Hepatocyte growth factor activator precursor | BCP & HP |
| K.WCATTHNYDR.D | Hepatocyte growth factor activator precursor | BCP & HP |
| K.YIPYTLYSVFNPSDHDLVLIR.L | Hepatocyte growth factor activator precursor | BCP & HP |
| R.AFTGKDCGTEK.C | Hepatocyte growth factor activator precursor | BCP & HP |
| R.EALVPLVADHK.C | Hepatocyte growth factor activator precursor | BCP & HP |
| R.LCNIEPDER.C | Hepatocyte growth factor activator precursor | BCP & HP |
| R.LEACESLTR.V | Hepatocyte growth factor activator precursor | BCP & HP |
| R.MLHACTSEGSAHR.K | Hepatocyte growth factor activator precursor | BCP & HP |
| R.NPDNDERPWCYVVK.D | Hepatocyte growth factor activator precursor | BCP & HP |
| R.QGHVEQCECFGGR.T | Hepatocyte growth factor activator precursor | BCP & HP |
| R.SQFVQPICLPEPGSTFPAGHK.C | Hepatocyte growth factor activator precursor | BCP & HP |
| R.TTDVTQTFGIEK.Y | Hepatocyte growth factor activator precursor | BCP & HP |
| R.VANYVDWINDR.I | Hepatocyte growth factor activator precursor | BCP & HP |
| K.DSALSWEYCR.L | Hepatocyte growth factor activator precursor | BCP & HP |
| R.SRWPAVFTR.V | Hepatocyte growth factor-like protein precursor | BCP & HP |
| K.M*VCGPSGSQLVLLK.L | Hepatocyte growth factor-like protein precursor | BCP & HP |
| R.EFCDLPR.C | Hepatocyte growth factor-like protein precursor | BCP & HP |
| R.EAACVWCNGEEYR.G | Hepatocyte growth factor-like protein precursor | BCP & HP |
| R.GTMATTVGGLPCQAWSHK.F | Hepatocyte growth factor-like protein precursor | BCP & HP |
| R.NGLEENFCR.N | Hepatocyte growth factor-like protein precursor | BCP & HP |
| R.NGLEENFCRNPDGDPGGPWCYTTDPAVR.F | Hepatocyte growth factor-like protein precursor | BCP & HP |
| R.NPDGDPGGPWCYTTDPAVR.F | Hepatocyte growth factor-like protein precursor | BCP & HP |
| R.QCFSSCHMPLTGYEVWLGTLFQNPQHGEPSLQR.V | Hepatocyte growth factor-like protein precursor | BCP & HP |
| R.QEATTVSCFR.G | Hepatocyte growth factor-like protein precursor | BCP & HP |
| R.QGQHFCGGSLVK.E | Hepatocyte growth factor-like protein precursor | BCP & HP |
| R.RCADDQPPSILDPPDQVQFEK.C | Hepatocyte growth factor-like protein precursor | BCP & HP |
| K.FLDQGLDDNYCR.N | Hepatocyte growth factor-like protein precursor | BCP & HP |
| R.SPLNDFQVLR.G | Hepatocyte growth factor-like protein precursor | BCP & HP |
| K.EQWILTAR.Q | Hepatocyte growth factor-like protein precursor | BCP & HP |
| R.TCIMNNGVGYR.G | Hepatocyte growth factor-like protein precursor | BCP & HP |
| R.TPFDYCALR.R | Hepatocyte growth factor-like protein precursor | BCP & HP |
| R.VALICLPPEWYVVPPGTK.C | Hepatocyte growth factor-like protein precursor | BCP & HP |
| R.VVGGHPGNSPWTVSLR.N | Hepatocyte growth factor-like protein precursor | BCP & HP |
| R.WSAETPHKPQFTFTSEPHAQLEENFCR.N | Hepatocyte growth factor-like protein precursor | BCP & HP |
| R.CTDDVRPQDCYHGAGEQYR.G | Hepatocyte growth factor-like protein precursor | BCP & HP |
| R.CGSEAQPR.Q | Hepatocyte growth factor-like protein precursor | BCP & HP |
| R.CGPLMDCR.A | Hepatocyte growth factor-like protein precursor | BCP & HP |
| R.CADDQPPSILDPPDQVQFEK.C | Hepatocyte growth factor-like protein precursor | BCP & HP |
| R.AAFCYQIR.R | Hepatocyte growth factor-like protein precursor | BCP & HP |
| K.YACKDLR.E | Hepatocyte growth factor-like protein precursor | BCP & HP |
| K.MVCGPSGSQLVLLKLER.S | Hepatocyte growth factor-like protein precursor | BCP & HP |
| R.RCTDDVRPQDCYHGAGEQYR.G | Hepatocyte growth factor-like protein precursor | BCP & HP |
| R.GTELQHLLHAVVPGPWQEDVADAEECAGR.C | Hepatocyte growth factor-like protein precursor | BCP & HP |
| K.FPNDHKYTPTLR.N | Hepatocyte growth factor-like protein precursor | BCP & HP |
| R.ENFCRNPDGSEAPWCFTLRPGMR.A | Hepatocyte growth factor-like protein precursor | BCP & HP |
| R.FQSCGIK.S | Hepatocyte growth factor-like protein precursor | BCP & HP |
| R.FTPEKYACK.D | Hepatocyte growth factor-like protein precursor | BCP & HP |
| K.CEIAGWGETK.G | Hepatocyte growth factor-like protein precursor | BCP & HP |
| K.MVCGPSGSQLVLLK.L | Hepatocyte growth factor-like protein precursor | BCP & HP |
| R.QIGSVYRLPPLR.K | Histidine-rich glycoprotein precursor | BCP & HP |
| R.HPNVFGFCR.A | Histidine-rich glycoprotein precursor | BCP & HP |
| R.ADLFYDVEALDLESPK.N | Histidine-rich glycoprotein precursor | BCP & HP |
| R.DHSHGPPLPQGPPPLLPM*SCSSCQHATFGTNGAQR.H | Histidine-rich glycoprotein precursor | BCP & HP |
| R.DHSHGPPLPQGPPPLLPMSCSSCQHATFGTNGAQR.H | Histidine-rich glycoprotein precursor | BCP & HP |
| R.GGEGTGYFVDFSVR.N | Histidine-rich glycoprotein precursor | BCP & HP |
| R.DGYLFQLLR.I | Histidine-rich glycoprotein precursor | BCP & HP |
| R.IADAHLDR.V | Histidine-rich glycoprotein precursor | BCP & HP |
| R.KGEVLPLPEANFPSFPLPH.H | Histidine-rich glycoprotein precursor | BCP & HP |
| R.KGEVLPLPEANFPSFPLPHHK.H | Histidine-rich glycoprotein precursor | BCP & HP |
| P.SEIVIGQCK.V | Histidine-rich glycoprotein precursor | BCP & HP |
| R.KYWNDCEPPDSRRPSEIVIGQCK.V | Histidine-rich glycoprotein precursor | BCP & HP |
| R.RPSEIVIGQCK.V | Histidine-rich glycoprotein precursor | BCP & HP |
| R.VIDFNCTTSSVSSALANTK.D | Histidine-rich glycoprotein precursor | BCP & HP |
| R.VIDFNCTTSSVSSALANTKDSPVLIDFFEDTER.Y | Histidine-rich glycoprotein precursor | BCP & HP |
| R.VRGGEGTGYFVDFSVR.N | Histidine-rich glycoprotein precursor | BCP & HP |
| R.KYWNDCEPPDSR.R | Histidine-rich glycoprotein precursor | BCP & HP |
| K.YKEENDDFASFRVDR.I | Histidine-rich glycoprotein precursor | BCP & HP |
| K.YKEENDDFASFR.V | Histidine-rich glycoprotein precursor | BCP & HP |
| A.VSPTDCSAVEPEAEK.A | Histidine-rich glycoprotein precursor | BCP & HP |
| A.VSPTDCSAVEPEAEKALDLINK.R | Histidine-rich glycoprotein precursor | BCP & HP |
| F.YDVEALDLESPK.N | Histidine-rich glycoprotein precursor | BCP & HP |
| K.ALDLINKR.R | Histidine-rich glycoprotein precursor | BCP & HP |
| K.DSPVLIDFFEDTER.Y | Histidine-rich glycoprotein precursor | BCP & HP |
| K.SGFPQVSMFFTHTFPK.- | Histidine-rich glycoprotein precursor | BCP & HP |
| K.FKSGFPQVSMFFTHTFPK.- | Histidine-rich glycoprotein precursor | BCP & HP |
| N.TKDSPVLIDFFEDTER.Y | Histidine-rich glycoprotein precursor | BCP & HP |
| K.YWNDCEPPDSR.R | Histidine-rich glycoprotein precursor | BCP & HP |
| K.YWNDCEPPDSRRPSEIVIGQCK.V | Histidine-rich glycoprotein precursor | BCP & HP |
| K.GEVLPLPEANFPSFPLPHHK.H | Histidine-rich glycoprotein precursor | BCP & HP |
| K.HPLKPDNQPFPQSVSESCPGK.F | Histidine-rich glycoprotein precursor | BCP & HP |
| K.SGFPQVSM*FFTHTFPK.- | Histidine-rich glycoprotein precursor | BCP & HP |
| S.PTDCSAVEPEAEK.A | Histidine-rich glycoprotein precursor | BCP & HP |
| M.SCSSCQHATFGTNGAQR.H | Histidine-rich glycoprotein precursor | BCP & HP |
| K.DSPVLIDFFEDTERYR.K | Histidine-rich glycoprotein precursor | BCP & HP |
| R.RDGYLFQLLR.I | Histidine-rich glycoprotein precursor | BCP & HP |
| K.KAKKPAAATVTKKVAKSPKKAKVAKPK.K | Histone H1.2 | BCP & HP |
| K.GTLVQTKGTGASGSFK.L | Histone H1.2 | BCP & HP |
| R.YFSAAVSRPGRGEPR.F | HLA class I histocompatibility antigen, alpha chain G precursor | BCP & HP |
| R.FDSDAASPR.E | HLA class I histocompatibility antigen, B-15 alpha chain precursor | BCP & HP |
| R.DGEDQTQDTELVETRPAGDR.T | HLA class I histocompatibility antigen, B-15 alpha chain precursor | BCP & HP |
| R.MAPRAPWIEQEGPEYWDRETQISKTNTQTYRESLR.N | HLA class I histocompatibility antigen, B-15 alpha chain precursor | BCP & HP |
| R.YLENGKETLQR.A | HLA class I histocompatibility antigen, B-15 alpha chain precursor | BCP & HP |
| R.FSGSGSGTDFTLTISR.L | HRV Fab N27-VL (Fragment) | BCP & HP |
| R.ATGIPDRFSGSGSGTDFTLTISR.L | HRV Fab N27-VL (Fragment) | BCP & HP |
| R.LLIYGASSR.A | HRV Fab N27-VL (Fragment) | BCP & HP |
| R.ASQSVSSSYLAWYQQKPGQAPR.L | HRV Fab N27-VL (Fragment) | BCP & HP |
| A.ELTLTQSPGTLSLSPGER.A | HRV Fab N27-VL (Fragment) | BCP & HP |
| K.NGRVEIIANDQGNR.I | HSPA5 protein | BCP & HP |
| R.TWNDPSVQQDIK.F | HSPA5 protein | BCP & HP |
| R.NELESYAYSLK.N | HSPA5 protein | BCP & HP |
| R.ITPSYVAFTPEGER.L | HSPA5 protein | BCP & HP |
| R.IINEPTAAAIAYGLDK.R | HSPA5 protein | BCP & HP |
| R.AKFEELNMDLFR.S | HSPA5 protein | BCP & HP |
| K.VYEGERPLTK.D | HSPA5 protein | BCP & HP |
| K.VTHAVVTVPAYFNDAQR.Q | HSPA5 protein | BCP & HP |
| K.TKPYIQVDIGGGQTK.T | HSPA5 protein | BCP & HP |
| K.SQIFSTASDNQPTVTIK.V | HSPA5 protein | BCP & HP |
| K.KTKPYIQVDIGGGQTKTFAPEEISAMVLTK.M | HSPA5 protein | BCP & HP |
| K.KKELEEIVQPIISK.L | HSPA5 protein | BCP & HP |
| K.EFFNGKEPSR.G | HSPA5 protein | BCP & HP |
| K.DNHLLGTFDLTGIPPAPR.G | HSPA5 protein | BCP & HP |
| K.DAGTIAGLNVMR.I | HSPA5 protein | BCP & HP |
| K.TFAPEEISAMVLTK.M | HSPA5 protein | BCP & HP |
| R.GSDVASASFPTSGM*LLWCSGASR.V | HSPC109 | BCP & HP |
| K.VVLGDQDLKKEEFHEQSFRVEK.I | Hyaluronan-binding protein 2 precursor | BCP & HP |
| K.GKFCEIGSDDCYVGDGYSYR.G | Hyaluronan-binding protein 2 precursor | BCP & HP |
| K.FTCACPDQFKGK.F | Hyaluronan-binding protein 2 precursor | BCP & HP |
| K.FCEIGSDDCYVGDGYSYR.G | Hyaluronan-binding protein 2 precursor | BCP & HP |
| K.ATIKSESGF.- | Hyaluronan-binding protein 2 precursor | BCP & HP |
| R.SKFTCACPDQFK.G | Hyaluronan-binding protein 2 precursor | BCP & HP |
| K.VVLGDQDLKKEEFHEQSFR.V | Hyaluronan-binding protein 2 precursor | BCP & HP |
| R.NPDADEKPWCFIK.V | Hyaluronan-binding protein 2 precursor | BCP & HP |
| K.TVCLPDGSFPSGSECHISGWGVTETGK.G | Hyaluronan-binding protein 2 precursor | BCP & HP |
| R.GQCLITQSPPYYR.C | Hyaluronan-binding protein 2 precursor | BCP & HP |
| R.QLYDHMIDDSMICAGNLQKPGQDTCQGDSGGPLTCEK.D | Hyaluronan-binding protein 2 precursor | BCP & HP |
| K.VQNTCKDNPCGR.G | Hyaluronan-binding protein 2 precursor | BCP & HP |
| K.VKLIANTLCNSR.Q | Hyaluronan-binding protein 2 precursor | BCP & HP |
| R.SKFTCACPDQFKGK.F | Hyaluronan-binding protein 2 precursor | BCP & HP |
| K.HPYTGPSCSQVVPVCRPNPCQNGATCSR.H | Hyaluronan-binding protein 2 precursor | BCP & HP |
| K.LIANTLCNSR.Q | Hyaluronan-binding protein 2 precursor | BCP & HP |
| K.RPGVYTQVTK.F | Hyaluronan-binding protein 2 precursor | BCP & HP |
| K.PGQDTCQGDSGGPLTCEK.D | Hyaluronan-binding protein 2 precursor | BCP & HP |
| K.LKPVDGHCALESK.Y | Hyaluronan-binding protein 2 precursor | BCP & HP |
| K.YSHYNERDEIPHNDIALLK.L | Hyaluronan-binding protein 2 precursor | BCP & HP |
| K.TVCLPDGSFPSGSECHISGWGVTETGKGSR.Q | Hyaluronan-binding protein 2 precursor | BCP & HP |
| R.ELGCGGALAAPGGAR.F | Hypothetical LOC284297 | BCP & HP |
| R.LTQVVEQERQER.Q | Hypothetical LOC284297 | BCP & HP |
| R.LADGPHGCAGR.L | Hypothetical LOC284297 | BCP & HP |
| R.QALLLGLTQLVEAAR.G | Hypothetical LOC284297 | BCP & HP |
| R.ELGCGGPQQPDPAAGR.F | Hypothetical LOC284297 | BCP & HP |
| R.DVGGQLQR.L | Hypothetical LOC284297 | BCP & HP |
| K.LTKNMLCAGYK.N | hypothetical protein LOC203074 | BCP & HP |
| K.DLYANTVLSGGSTM*YPGIADR.M | hypothetical protein LOC345651 | BCP & HP |
| K.CDVDIRK.D | hypothetical protein LOC345651 | BCP & HP |
| K.IIAPPERK.Y | hypothetical protein LOC345651 | BCP & HP |
| K.IKIIAPPER.K | hypothetical protein LOC345651 | BCP & HP |
| R.DLTDYLM*K.I | hypothetical protein LOC345651 | BCP & HP |
| R.HQGVM*VGM*GQK.D | hypothetical protein LOC345651 | BCP & HP |
| R.HQGVM*VGMGQK.D | hypothetical protein LOC345651 | BCP & HP |
| R.HQGVMVGM*GQK.D | hypothetical protein LOC345651 | BCP & HP |
| R.HQGVMVGMGQK.D | hypothetical protein LOC345651 | BCP & HP |
| R.SYELPDGQVITIGNER.F | hypothetical protein LOC345651 | BCP & HP |
| R.DLTDYLMK.I | hypothetical protein LOC345651 | BCP & HP |
| R.EVQYLLNK.A | Hypoxia up-regulated protein 1 precursor | BCP & HP |
| K.LPATEKPVLLSK.D | Hypoxia up-regulated protein 1 precursor | BCP & HP |
| K.MMALDREVQYLLNK.A | Hypoxia up-regulated protein 1 precursor | BCP & HP |
| K.LSAASTWLEDEGVGATTVMLK.E | Hypoxia up-regulated protein 1 precursor | BCP & HP |
| R.LTISKDTSK.N | Ig heavy chain V-II region SESS precursor | BCP & HP |
| -.EVQLVETGGGLIQPGGSLR.L | Ig heavy chain V-III region BUT | BCP & HP |
| R.BTVYLQMBSLR.A | Ig heavy chain V-III region BUT | BCP & HP |
| R.AEDTAVYYCAR.R | Ig heavy chain V-III region BUT | BCP & HP |
| R.VEDTATYYCAR.V | Ig heavy chain V-III region GAR | BCP & HP |
| -.QVKLVQAGGGVVQPGR.S | Ig heavy chain V-III region HIL | BCP & HP |
| R.TEDTAVYYCAR.D | Ig heavy chain V-III region HIL | BCP & HP |
| -.DVQLVESGGGLVKPGGSLR.L | Ig heavy chain V-III region JON | BCP & HP |
| R.AEDTAVYYCAR.R | Ig heavy chain V-III region WEA | BCP & HP |
| K.NSLYLQMSSLR.A | Ig heavy chain V-III region WEA | BCP & HP |
| -.DIQLTQSPSSLSASVGDR.V | Ig kappa chain V-I region BAN | BCP & HP |
| -.DIQM*TQSPSTLSASVGDR.V | Ig kappa chain V-I region EU | BCP & HP |
| -.DIQMTQSPSTLSASVGDR.V | Ig kappa chain V-I region EU | BCP & HP |
| K.ASSLESGVPSR.F | Ig kappa chain V-I region EU | BCP & HP |
| -.DIQMTQSPSSLSVSVGDR.V | Ig kappa chain V-I region Lay | BCP & HP |
| -.DIQM*TQSPSSLSVSVGDR.V | Ig kappa chain V-I region Lay | BCP & HP |
| -.DVQM*TQSPSSLSASVGDR.V | Ig kappa chain V-I region Mev | BCP & HP |
| -.DVQMTQSPSSLSASVGDR.V | Ig kappa chain V-I region Mev | BCP & HP |
| -.DIQMTQSPSSLSATVGDR.V | Ig kappa chain V-I region Ni | BCP & HP |
| -.DIQM*TQSPSSLSATVGDR.V | Ig kappa chain V-I region Ni | BCP & HP |
| -.DIVLTQSPLSLPVTPGEPASISCR.S | Ig kappa chain V-II region MIL | BCP & HP |
| G.EIVLTQSPGTLSLSPGER.A | Ig kappa chain V-III region GOL | BCP & HP |
| R.ATGIPDRFSGSGSGTDFTLTISR.L | Ig kappa chain V-III region GOL | BCP & HP |
| R.FSGSGSGTDFTLTISR.L | Ig kappa chain V-III region GOL | BCP & HP |
| R.LEPEDFAVYYCQQYGSSPR.S | Ig kappa chain V-III region GOL | BCP & HP |
| -.DIVM*TQSPDSLVVSLGER.A | Ig kappa chain V-IV region STH (Fragment) | BCP & HP |
| K.SSQSVLYSSNNK.N | Ig kappa chain V-IV region STH (Fragment) | BCP & HP |
| R.FSGSNSGNTATLTISR.V | Ig lambda chain V-III region LOI | BCP & HP |
| -.YVLTQPPSVSVAPGETAR.L | Ig lambda chain V-III region LOI | BCP & HP |
| A.NFMLTQPHSVSESPGK.T | Ig lambda chain V-VI region EB4 precursor | BCP & HP |
| R.TVDKSTGKPTLYNVSLVMSDTAGTCY.- | Ig mu heavy chain disease protein | BCP & HP |
| R.ESATITCLVTGFSPADVFVQWM*QR.G | Ig mu heavy chain disease protein | BCP & HP |
| R.ESATITCLVTGFSPADVFVQWMQR.G | Ig mu heavy chain disease protein | BCP & HP |
| R.FTCTVTHTDLPSPLK.Q | Ig mu heavy chain disease protein | BCP & HP |
| R.FTCTVTHTDLPSPLKQTISRPK.G | Ig mu heavy chain disease protein | BCP & HP |
| R.GQPLSPEK.Y | Ig mu heavy chain disease protein | BCP & HP |
| R.GQPLSPEKYVTSAPM*PEPQAPGR.Y | Ig mu heavy chain disease protein | BCP & HP |
| R.GQPLSPEKYVTSAPMPEPQAPGR.Y | Ig mu heavy chain disease protein | BCP & HP |
| R.DGFFGNPR.K | Ig mu heavy chain disease protein | BCP & HP |
| R.TVDKSTGKPTLYNVSLVM*SDTAGTCY.- | Ig mu heavy chain disease protein | BCP & HP |
| K.YVTSAPMPEPQAPGR.Y | Ig mu heavy chain disease protein | BCP & HP |
| R.VFAIPPSFASIFLTK.S | Ig mu heavy chain disease protein | BCP & HP |
| R.YFAHSILTVSEEEWNTGETYTCVVAHEALPNR.V | Ig mu heavy chain disease protein | BCP & HP |
| R.YFAHSILTVSEEEWNTGETYTCVVAHEALPNRVTER.T | Ig mu heavy chain disease protein | BCP & HP |
| R.QIEVSWLR.E | Ig mu heavy chain disease protein | BCP & HP |
| K.LICQATGFSPR.Q | Ig mu heavy chain disease protein | BCP & HP |
| F.TCTVTHTDLPSPLK.Q | Ig mu heavy chain disease protein | BCP & HP |
| I.PPSFASIFLTK.S | Ig mu heavy chain disease protein | BCP & HP |
| K.ESDWLGQSMFTCR.V | Ig mu heavy chain disease protein | BCP & HP |
| R.EGKQVGSGVTTDEVEAEAKESGPTTYK.V | Ig mu heavy chain disease protein | BCP & HP |
| K.GVALHRPDVYLLPPAREQLNLR.E | Ig mu heavy chain disease protein | BCP & HP |
| K.LTCLVTDLTTYDSVTISWTR.Q | Ig mu heavy chain disease protein | BCP & HP |
| K.QVGSGVTTDEVEAEAK.E | Ig mu heavy chain disease protein | BCP & HP |
| K.VTSTLTIK.E | Ig mu heavy chain disease protein | BCP & HP |
| K.STGKPTLYNVSLVM*SDTAGTCY.- | Ig mu heavy chain disease protein | BCP & HP |
| K.STGKPTLYNVSLVMSDTAGTC.Y | Ig mu heavy chain disease protein | BCP & HP |
| K.STGKPTLYNVSLVMSDTAGTCY.- | Ig mu heavy chain disease protein | BCP & HP |
| K.VSVFVPPR.D | Ig mu heavy chain disease protein | BCP & HP |
| K.VSVFVPPRDGFFGNPR.K | Ig mu heavy chain disease protein | BCP & HP |
| K.SKLICQATGFSPR.Q | Ig mu heavy chain disease protein | BCP & HP |
| K.YVTSAPM*PEPQAPGR.Y | Ig mu heavy chain disease protein | BCP & HP |
| K.GVALHRPDVYLLPPAR.E | Ig mu heavy chain disease protein | BCP & HP |
| K.YQKEEFCGLLSSPTGPLSSCHK.L | IgGFc-binding protein precursor | BCP & HP |
| K.LTYNHGGITGSR.G | IgGFc-binding protein precursor | BCP & HP |
| K.NQNRGNPAVSYVR.V | IgGFc-binding protein precursor | BCP & HP |
| K.NTGREEFLTAFLQNYQLAYSK.A | IgGFc-binding protein precursor | BCP & HP |
| K.SVPGCEGVALVVAQTK.A | IgGFc-binding protein precursor | BCP & HP |
| K.VAVIVSNDHAGK.L | IgGFc-binding protein precursor | BCP & HP |
| K.VPSSYAEALCGLCGNFNGDPADDLALR.G | IgGFc-binding protein precursor | BCP & HP |
| K.VTASSPVAVLSGHSCAQK.H | IgGFc-binding protein precursor | BCP & HP |
| K.VTVNGVDMK.L | IgGFc-binding protein precursor | BCP & HP |
| K.LPVVLANGQIR.A | IgGFc-binding protein precursor | BCP & HP |
| K.ALASYVAACQAAGVVIEDWR.A | IgGFc-binding protein precursor | BCP & HP |
| R.APGWDPLCWDECR.G | IgGFc-binding protein precursor | BCP & HP |
| K.VTVNGVDM*K.L | IgGFc-binding protein precursor | BCP & HP |
| K.LDSLVAQQLQSK.N | IgGFc-binding protein precursor | BCP & HP |
| K.LCGACGNFDGDQTNDWHDSQEKPAMEK.W | IgGFc-binding protein precursor | BCP & HP |
| K.GMVCQEHSCKPGQVCQPSGGILSCVTK.D | IgGFc-binding protein precursor | BCP & HP |
| K.GCVLDVCMGGGDRDILCK.A | IgGFc-binding protein precursor | BCP & HP |
| K.GCVLDVCMGGGDHDILCK.A | IgGFc-binding protein precursor | BCP & HP |
| R.ASQHGSDVVIETDFGLR.V | IgGFc-binding protein precursor | BCP & HP |
| R.YDLAFVVASQATK.L | IgGFc-binding protein precursor | BCP & HP |
| K.DPCHGVTCRPQETCK.E | IgGFc-binding protein precursor | BCP & HP |
| K.AISGLTIDGHAVGAK.L | IgGFc-binding protein precursor | BCP & HP |
| K.AIGYATAADCGR.T | IgGFc-binding protein precursor | BCP & HP |
| K.AGCVAESTAVCR.A | IgGFc-binding protein precursor | BCP & HP |
| R.YYPLGQTFYPGPGCDSLCR.C | IgGFc-binding protein precursor | BCP & HP |
| R.LRVPAAYAGSLCGLCGNYNQDPADDLK.A | IgGFc-binding protein precursor | BCP & HP |
| K.FDFQGTCNYVLATTGCPGVSTQGLTPFTVTTK.N | IgGFc-binding protein precursor | BCP & HP |
| R.AYSHSVSLTR.G | IgGFc-binding protein precursor | BCP & HP |
| R.TVLSPVEPSCEGMQCAAGQR.C | IgGFc-binding protein precursor | BCP & HP |
| R.TVLSPVEPSCEGM*QCAAGQR.C | IgGFc-binding protein precursor | BCP & HP |
| R.TCQGSCAALSGLTGCTTR.C | IgGFc-binding protein precursor | BCP & HP |
| R.SRLPVSLSEGR.L | IgGFc-binding protein precursor | BCP & HP |
| R.SPANCPLSCPANSR.Y | IgGFc-binding protein precursor | BCP & HP |
| R.SLAAYTAACQAAGVAVKPWR.T | IgGFc-binding protein precursor | BCP & HP |
| R.RPDFCPFQCPAHSHYELCGDSCPGSCPSLSAPEGCESACR.E | IgGFc-binding protein precursor | BCP & HP |
| R.VAYDLVYYVR.V | IgGFc-binding protein precursor | BCP & HP |
| R.NPQGPFATCQAVLSPSEYFR.Q | IgGFc-binding protein precursor | BCP & HP |
| R.RVSYVGLVTVR.A | IgGFc-binding protein precursor | BCP & HP |
| R.YYPLGEVFYPGPECER.R | IgGFc-binding protein precursor | BCP & HP |
| R.NMVLQTTK.G | IgGFc-binding protein precursor | BCP & HP |
| R.NEVTYDPYLVLIPDVAAYCPAYVVK.S | IgGFc-binding protein precursor | BCP & HP |
| R.GSQAVSYTR.S | IgGFc-binding protein precursor | BCP & HP |
| R.GSQTVSYTR.A | IgGFc-binding protein precursor | BCP & HP |
| R.ISVAQGASK.A | IgGFc-binding protein precursor | BCP & HP |
| R.KFDFQGTCNYVLATTGCPGVSTQGLTPFTVTTK.N | IgGFc-binding protein precursor | BCP & HP |
| R.LRVPAAYAASLCGLCGNYNQDPADDLK.A | IgGFc-binding protein precursor | BCP & HP |
| R.QCVYDLCAQKGDK.A | IgGFc-binding protein precursor | BCP & HP |
| R.FAVLQENVAWGNGR.V | IgGFc-binding protein precursor | BCP & HP |
| R.CGPGGGSLVCTPASCGLGEVCGLLPSGQHGCQPVSTAECQAWGD.P | IgGFc-binding protein precursor | BCP & HP |
| R.CLANGGIHYITLDGR.V | IgGFc-binding protein precursor | BCP & HP |
| R.CLLPGQSGPLCDALATYAAACQAAGATVHPWR.S | IgGFc-binding protein precursor | BCP & HP |
| R.CPGLQNTIPWYR.V | IgGFc-binding protein precursor | BCP & HP |
| R.CSCSSSSGLTCQAAGCPPGR.V | IgGFc-binding protein precursor | BCP & HP |
| R.CSVQNGLLGCYPDR.F | IgGFc-binding protein precursor | BCP & HP |
| R.CTCNGATHQVTCR.D | IgGFc-binding protein precursor | BCP & HP |
| R.REYPGQVLVDDVLQYLPFQAADGQVQVFR.Q | IgGFc-binding protein precursor | BCP & HP |
| R.EYPGQVLVDDVLQYLPFQAADGQVQVFR.Q | IgGFc-binding protein precursor | BCP & HP |
| R.FQDQVCGLCGNYNGDPADDFLTPDGALAPDAVEFASSWK.L | IgGFc-binding protein precursor | BCP & HP |
| R.VVAEVQICHGK.T | IgGFc-binding protein precursor | BCP & HP |
| R.VLVENEHRGSQTVSYTR.A | IgGFc-binding protein precursor | BCP & HP |
| R.VNGVLTALPVSVADGR.I | IgGFc-binding protein precursor | BCP & HP |
| R.VPAAYAGSLCGLCGNYNQDPADDLK.A | IgGFc-binding protein precursor | BCP & HP |
| R.VTLQPYNVAQLQSSVDLSGSK.V | IgGFc-binding protein precursor | BCP & HP |
| R.GEVGFVLVDNQR.S | IgGFc-binding protein precursor | BCP & HP |
| R.GNPAVSYVR.V | IgGFc-binding protein precursor | BCP & HP |
| R.GATTSPGVYELSSR.C | IgGFc-binding protein precursor | BCP & HP |
| K.VFPLSLCSTQPDGNVVIACLVQGFFPQEPLSVTWSESGQGVTAR.N | IGHA1 protein | BCP & HP |
| K.TFTCTAAYPESK.T | IGHA1 protein | BCP & HP |
| R.GFSPKDVLVR.W | IGHA1 protein | BCP & HP |
| R.QEPSQGTTTFAVTSILR.V | IGHA1 protein | BCP & HP |
| R.EKYLTWASR.Q | IGHA1 protein | BCP & HP |
| K.TPLTATLSK.S | IGHA1 protein | BCP & HP |
| K.YLTWASR.Q | IGHA1 protein | BCP & HP |
| R.DASGVTFTWTPSSGK.S | IGHA1 protein | BCP & HP |
| R.SDDTAVYYCAR.R | IGHA1 protein | BCP & HP |
| R.VAAEDWK.K | IGHA1 protein | BCP & HP |
| K.SGNTFRPEVHLLPPPSEELALNELVTLTCLAR.G | IGHA1 protein | BCP & HP |
| R.WLQGSQELPREK.Y | IGHA1 protein | BCP & HP |
| K.SAVQGPPDR.D | IGHA1 protein | BCP & HP |
| R.WLQGSQELPR.E | IGHA1 protein | BCP & HP |
| H.LLPPPSEELALNELVTLTCLAR.G | IGHA1 protein | BCP & HP |
| K.KGDTFSCMVGHEALPLAFTQE.T | IGHA1 protein | BCP & HP |
| K.KGDTFSCM*VGHEALPLAFTQE.T | IGHA1 protein | BCP & HP |
| K.GTTVTVSSASPTSPK.V | IGHA1 protein | BCP & HP |
| R.NFPPSQDASGDLYTTSSQLTLPATQCLAGK.S | IGHA1 protein | BCP & HP |
| C.EVQLVESGGGLVQPGR.S | IGHD protein | BCP & HP |
| K.APDVFPIISGCR.H | IGHD protein | BCP & HP |
| K.CVVQHTASK.S | IGHD protein | BCP & HP |
| K.DAHLTWEVAGK.V | IGHD protein | BCP & HP |
| K.DAHLTWEVAGKVPTGGVEEGLLER.H | IGHD protein | BCP & HP |
| K.EIFRWPESPK.A | IGHD protein | BCP & HP |
| R.VPAPPSPQPATYTCVVSHEDSR.T | IGHD protein | BCP & HP |
| R.ETKTPECPSHTQPLGVYLLTPAVQDLWLR.D | IGHD protein | BCP & HP |
| K.NSLYLQMNSLR.A | IGHD protein | BCP & HP |
| R.LMALREPAAQAPVK.L | IGHD protein | BCP & HP |
| R.LM*ALREPAAQAPVK.L | IGHD protein | BCP & HP |
| K.VPTGGVEEGLLER.H | IGHD protein | BCP & HP |
| Q.GTTVTVSSAPTKAPDVFPIISGCR.H | IGHD protein | BCP & HP |
| R.AEDTALYYCAK.H | IGHD protein | BCP & HP |
| R.DKATFTCFVVGSDLK.D | IGHD protein | BCP & HP |
| R.DSYYM*TSSQLSTPLQQWR.Q | IGHD protein | BCP & HP |
| R.DSYYMTSSQLSTPLQQWR.Q | IGHD protein | BCP & HP |
| R.EPAAQAPVK.L | IGHD protein | BCP & HP |
| R.EVNTSGFAPARPPPQPGSTTFWAWSVLR.V | IGHD protein | BCP & HP |
| R.SLWNAGTSVTCTLNHPSLPPQR.L | IGHD protein | BCP & HP |
| K.TPECPSHTQPLGVYLLTPAVQDLWLR.D | IGHD protein | BCP & HP |
| K.YVTSAPM*PEPQAPGR.Y | IGHM protein | BCP & HP |
| R.DGFFGNPR.K | IGHM protein | BCP & HP |
| K.YAATSQVLLPSK.D | IGHM protein | BCP & HP |
| K.STGKPTLYNVSLVMSDTAGTCY.- | IGHM protein | BCP & HP |
| K.STGKPTLYNVSLVMSDTAGTC.Y | IGHM protein | BCP & HP |
| K.YAATSQVLLPSKDVM*QGTDEHVVCK.V | IGHM protein | BCP & HP |
| K.NVPLPVIAELPPKVSVFVPPR.D | IGHM protein | BCP & HP |
| K.EKNVPLPVIAELPPK.V | IGHM protein | BCP & HP |
| K.ESDWLSQSM*FTCR.V | IGHM protein | BCP & HP |
| K.ESDWLSQSMFTCR.V | IGHM protein | BCP & HP |
| K.GVALHRPDVYLLPPAR.E | IGHM protein | BCP & HP |
| K.GVALHRPDVYLLPPAREQLNLR.E | IGHM protein | BCP & HP |
| K.LICQATGFSPR.Q | IGHM protein | BCP & HP |
| K.VQHPNGNKEKNVPLPVIAELPPKVSVFVPPR.D | IGHM protein | BCP & HP |
| K.NVPLPVIAELPPK.V | IGHM protein | BCP & HP |
| K.VTSTLTIKESDWLSQSMFTCR.V | IGHM protein | BCP & HP |
| K.NVPLPVIAELPPKVSVFVPPRDGFFGNPR.K | IGHM protein | BCP & HP |
| K.QVGSGVTTDQVQAEAK.E | IGHM protein | BCP & HP |
| K.YAATSQVLLPSKDVMQGTDEHVVCK.V | IGHM protein | BCP & HP |
| K.QVGSGVTTDQVQAEAKESGPTTYK.V | IGHM protein | BCP & HP |
| K.VSVFVPPR.D | IGHM protein | BCP & HP |
| K.VSVFVPPRDGFFGNPR.K | IGHM protein | BCP & HP |
| K.VTSTLTIK.E | IGHM protein | BCP & HP |
| K.LTCLVTDLTTYDSVTISWTR.Q | IGHM protein | BCP & HP |
| R.EGKQVGSGVTTDQVQAEAK.E | IGHM protein | BCP & HP |
| K.YVTSAPMPEPQAPGR.Y | IGHM protein | BCP & HP |
| R.DTLYLQM*NSLR.A | IGHM protein | BCP & HP |
| K.DVMQGTDEHVVCK.V | IGHM protein | BCP & HP |
| R.ESATITCLVTGFSPADVFVQWM*QR.G | IGHM protein | BCP & HP |
| R.ESATITCLVTGFSPADVFVQWMQR.G | IGHM protein | BCP & HP |
| R.FTCTVTHTDLPSPLK.Q | IGHM protein | BCP & HP |
| R.FTCTVTHTDLPSPLKQTISRPK.G | IGHM protein | BCP & HP |
| R.GGKYAATSQVLLPSK.D | IGHM protein | BCP & HP |
| R.GGKYAATSQVLLPSKDVM*QGTDEHVVCK.V | IGHM protein | BCP & HP |
| R.GGKYAATSQVLLPSKDVMQGTDEHVVCK.V | IGHM protein | BCP & HP |
| R.GQPLSPEK.Y | IGHM protein | BCP & HP |
| R.GQPLSPEKYVTSAPM*PEPQAPGR.Y | IGHM protein | BCP & HP |
| R.GQPLSPEKYVTSAPMPEPQAPGR.Y | IGHM protein | BCP & HP |
| R.QIQVSWLR.E | IGHM protein | BCP & HP |
| I.PPSFASIFLTK.S | IGHM protein | BCP & HP |
| R.TVDKSTGKPTLYNVSLVM*SDTAGTCY.- | IGHM protein | BCP & HP |
| K.DVM*QGTDEHVVCK.V | IGHM protein | BCP & HP |
| G.SGVTTDQVQAEAK.E | IGHM protein | BCP & HP |
| F.TCTVTHTDLPSPLK.Q | IGHM protein | BCP & HP |
| C.EVQLLESGGGLVQPGGSLR.L | IGHM protein | BCP & HP |
| K.SKLICQATGFSPR.Q | IGHM protein | BCP & HP |
| V.PLPVIAELPPK.V | IGHM protein | BCP & HP |
| R.YFAHSILTVSEEEWNTGETYTCVVAHEALPNRVTER.T | IGHM protein | BCP & HP |
| R.YFAHSILTVSEEEWNTGETYTCVVAHEALPNR.V | IGHM protein | BCP & HP |
| R.VFAIPPSFASIFLTK.S | IGHM protein | BCP & HP |
| K.STGKPTLYNVSLVM*SDTAGTCY.- | IGHM protein | BCP & HP |
| R.TVDKSTGKPTLYNVSLVMSDTAGTCY.- | IGHM protein | BCP & HP |
| K.GFYPSDIAVEWESNGQPENNYK.T | IGHV4-31 protein | BCP & HP |
| K.FNWYVDGVEVHNAK.T | IGHV4-31 protein | BCP & HP |
| K.DTLMISR.T | IGHV4-31 protein | BCP & HP |
| K.DTLM*ISR.T | IGHV4-31 protein | BCP & HP |
| K.DSLYLQMNSLR.V | IGHV4-31 protein | BCP & HP |
| K.ALPAPIEK.T | IGHV4-31 protein | BCP & HP |
| K.GQPREPQVYTLPPSR.E | IGHV4-31 protein | BCP & HP |
| K.DSLYLQM*NSLR.V | IGHV4-31 protein | BCP & HP |
| R.EPQVYTLPPSR.E | IGHV4-31 protein | BCP & HP |
| K.GPSVFPLAPSSK.S | IGHV4-31 protein | BCP & HP |
| K.GPSVFPLAPSSKSTSGGTAALGCLVK.D | IGHV4-31 protein | BCP & HP |
| V.SVLTVLHQDWLNGK.E | IGHV4-31 protein | BCP & HP |
| T.PPVLDSDGSFFLYSK.L | IGHV4-31 protein | BCP & HP |
| R.VVSVLTVLHQDWLNGKEYK.C | IGHV4-31 protein | BCP & HP |
| R.VVSVLTVLHQDWLNGK.E | IGHV4-31 protein | BCP & HP |
| R.TPEVTCVVVDVSHED.P | IGHV4-31 protein | BCP & HP |
| R.STSGGTAALGCLVK.D | IGHV4-31 protein | BCP & HP |
| R.EPQVYTLPPSRDELTK.N | IGHV4-31 protein | BCP & HP |
| R.CPAPELLGGPSVFLFPPKPK.D | IGHV4-31 protein | BCP & HP |
| K.WYVDGVEVHNAK.T | IGHV4-31 protein | BCP & HP |
| K.TTPPVLDSDGSFFLYSK.L | IGHV4-31 protein | BCP & HP |
| K.THTCPPCPAPELLGGPSVFLFPPKPK.D | IGHV4-31 protein | BCP & HP |
| K.SCDKTHTCPPCPAPELLGGPSVFLFPPKPK.D | IGHV4-31 protein | BCP & HP |
| K.NQVSLTCLVK.G | IGHV4-31 protein | BCP & HP |
| R.ATGIPDRFSGSGSGTDFTLTISR.L | IGK@ protein | BCP & HP |
| R.LLM*FGSSSR.A | IGK@ protein | BCP & HP |
| R.FSGSGSGTDFTLTISR.L | IGK@ protein | BCP & HP |
| K.HKVYACEVTHQGLSSPVTK.S | IGK@ protein | BCP & HP |
| Y.ACEVTHQGLSSPVTK.S | IGK@ protein | BCP & HP |
| R.TVAAPSVFIFPPSDEQLKSGTASVVCLLNNFYPR.E | IGK@ protein | BCP & HP |
| R.TVAAPSVFIFPPSDEQLK.S | IGK@ protein | BCP & HP |
| A.PSVFIFPPSDEQLK.S | IGK@ protein | BCP & HP |
| E.IVLTQSPATLSLSPGER.A | IGK@ protein | BCP & HP |
| K.DSTYSLSSTLTLSK.A | IGK@ protein | BCP & HP |
| K.VYACEVTHQGLSSPVTK.S | IGK@ protein | BCP & HP |
| K.RTVAAPSVFIFPPSDEQLK.S | IGK@ protein | BCP & HP |
| K.SFNRGEC.- | IGK@ protein | BCP & HP |
| K.SGTASVVCLLNNFYPR.E | IGK@ protein | BCP & HP |
| K.VDNALQSGNSQESVTEQDSK.D | IGK@ protein | BCP & HP |
| K.VDNALQSGNSQESVTEQDSKDSTYSLSSTLTLSK.A | IGK@ protein | BCP & HP |
| K.VQWKVDNALQSGNSQESVTEQDSK.D | IGK@ protein | BCP & HP |
| K.VQWKVDNALQSGNSQESVTEQDSKDSTYSLSSTLTLSK.A | IGK@ protein | BCP & HP |
| G.EIVLTQSPATLSLSPGER.A | IGK@ protein | BCP & HP |
| A.PSVFIFPPSDEQLK.S | IGKC protein | BCP & HP |
| R.TVAAPSVFIFPPSDEQLKSGTASVVCLLNNFYPR.E | IGKC protein | BCP & HP |
| K.DSTYSLSSTLTLSK.A | IGKC protein | BCP & HP |
| K.SFNRGEC.- | IGKC protein | BCP & HP |
| Y.ACEVTHQGLSSPVTK.S | IGKC protein | BCP & HP |
| K.HKVYACEVTHQGLSSPVTK.S | IGKC protein | BCP & HP |
| R.TVAAPSVFIFPPSDEQLK.S | IGKC protein | BCP & HP |
| R.GTVAAPSVFIFPPSDEQLK.S | IGKC protein | BCP & HP |
| K.VYACEVTHQGLSSPVTK.S | IGKC protein | BCP & HP |
| K.VQWKVDNALQSGNSQESVTEQDSKDSTYSLSSTLTLSK.A | IGKC protein | BCP & HP |
| K.VQWKVDNALQSGNSQESVTEQDSK.D | IGKC protein | BCP & HP |
| K.VDNALQSGNSQESVTEQDSKDSTYSLSSTLTLSK.A | IGKC protein | BCP & HP |
| K.VDNALQSGNSQESVTEQDSK.D | IGKC protein | BCP & HP |
| K.SGTASVVCLLNNFYPR.E | IGKC protein | BCP & HP |
| R.LLIYGASSR.A | IGKC protein | BCP & HP |
| K.VQWKVDNALQSGNSQESVTEQDSKDSTYSLSSTLTLSK.A | IGKV2-24 protein | BCP & HP |
| R.FSGVPDRFSGSGAGTDFTLK.I | IGKV2-24 protein | BCP & HP |
| R.FSGSGAGTDFTLK.I | IGKV2-24 protein | BCP & HP |
| R.TVAAPSVFIFPPSDEQLKSGTASVVCLLNNFYPR.E | IGKV2-24 protein | BCP & HP |
| G.DIVMTQTPLSSPVTLGQPASISCR.S | IGKV2-24 protein | BCP & HP |
| G.DIVM*TQTPLSSPVTLGQPASISCR.S | IGKV2-24 protein | BCP & HP |
| K.DSTYSLSSTLTLSK.A | IGKV2-24 protein | BCP & HP |
| K.HKVYACEVTHQGLSSPVTK.S | IGKV2-24 protein | BCP & HP |
| K.RTVAAPSVFIFPPSDEQLK.S | IGKV2-24 protein | BCP & HP |
| K.SFNRGEC.- | IGKV2-24 protein | BCP & HP |
| K.SGTASVVCLLNNFYPR.E | IGKV2-24 protein | BCP & HP |
| R.TVAAPSVFIFPPSDEQLK.S | IGKV2-24 protein | BCP & HP |
| K.VDNALQSGNSQESVTEQDSKDSTYSLSSTLTLSK.A | IGKV2-24 protein | BCP & HP |
| K.VQWKVDNALQSGNSQESVTEQDSK.D | IGKV2-24 protein | BCP & HP |
| K.VYACEVTHQGLSSPVTK.S | IGKV2-24 protein | BCP & HP |
| A.PSVFIFPPSDEQLK.S | IGKV2-24 protein | BCP & HP |
| Y.ACEVTHQGLSSPVTK.S | IGKV2-24 protein | BCP & HP |
| K.VDNALQSGNSQESVTEQDSK.D | IGKV2-24 protein | BCP & HP |
| K.QSNNKYAASSYLSLTPEQWK.S | IGL@ protein | BCP & HP |
| A.ASSYLSLTPEQWK.S | IGL@ protein | BCP & HP |
| K.AAPSVTLFPPSSEELQANK.A | IGL@ protein | BCP & HP |
| K.ADSSPVKAGVETTTPSKQSNNK.Y | IGL@ protein | BCP & HP |
| K.AGVETTTPSK.Q | IGL@ protein | BCP & HP |
| K.AGVETTTPSKQSNNK.Y | IGL@ protein | BCP & HP |
| K.LTVLGQPKAAPSVTLFPPSSEELQANK.A | IGL@ protein | BCP & HP |
| F.PPSSEELQANK.A | IGL@ protein | BCP & HP |
| K.YAASSYLSLTPEQWK.S | IGL@ protein | BCP & HP |
| R.FSGSNSGNTATLTISR.V | IGL@ protein | BCP & HP |
| R.ITCGGNNIGSK.S | IGL@ protein | BCP & HP |
| R.SYSCQVTHEGSTVEK.T | IGL@ protein | BCP & HP |
| R.SYSCQVTHEGSTVEKTVAPTECS.- | IGL@ protein | BCP & HP |
| Y.SCQVTHEGSTVEK.T | IGL@ protein | BCP & HP |
| K.ATLVCLISDFYPGAVTVAWK.A | IGL@ protein | BCP & HP |
| K.ADSSPVKAGVETTTPSK.Q | IGL@ protein | BCP & HP |
| S.YVLTQPASVSVAPGQTAR.I | IGLC1 protein | BCP & HP |
| F.PPSSEELQANK.A | IGLC1 protein | BCP & HP |
| Y.SCQVTHEGSTVEK.T | IGLC1 protein | BCP & HP |
| R.SYSCQVTHEGSTVEKTVAPTECS.- | IGLC1 protein | BCP & HP |
| R.SYSCQVTHEGSTVEK.T | IGLC1 protein | BCP & HP |
| K.YAASSYLSLTPEQWK.S | IGLC1 protein | BCP & HP |
| K.VTVLGQPK.A | IGLC1 protein | BCP & HP |
| K.QSNNKYAASSYLSLTPEQWK.S | IGLC1 protein | BCP & HP |
| K.ATLVCLISDFYPGAVTVAWK.A | IGLC1 protein | BCP & HP |
| K.ANPTVTLFPPSSEELQANK.A | IGLC1 protein | BCP & HP |
| K.ADGSPVKAGVETTKPSK.Q | IGLC1 protein | BCP & HP |
| A.ASSYLSLTPEQWK.S | IGLC1 protein | BCP & HP |
| K.AGVETTKPSK.Q | IGLC1 protein | BCP & HP |
| K.AAPSVTLFPPSSEELQANK.A | IGLV2-14 protein | BCP & HP |
| K.AGVETTTPSKQSNNK.Y | IGLV2-14 protein | BCP & HP |
| K.LM*IYDVSNRPSGVSNR.F | IGLV2-14 protein | BCP & HP |
| K.LTVLGQPKAAPSVTLFPPSSEELQANK.A | IGLV2-14 protein | BCP & HP |
| K.QSNNKYAASSYLSLTPEQWK.S | IGLV2-14 protein | BCP & HP |
| F.PPSSEELQANK.A | IGLV2-14 protein | BCP & HP |
| K.YAASSYLSLTPEQWK.S | IGLV2-14 protein | BCP & HP |
| R.SYSCQVTHEGSTVEK.T | IGLV2-14 protein | BCP & HP |
| R.SYSCQVTHEGSTVEKTVAPTECS.- | IGLV2-14 protein | BCP & HP |
| K.ADSSPVKAGVETTTPSK.Q | IGLV2-14 protein | BCP & HP |
| K.ADSSPVKAGVETTTPSKQSNNK.Y | IGLV2-14 protein | BCP & HP |
| K.AGVETTTPSK.Q | IGLV2-14 protein | BCP & HP |
| Y.SCQVTHEGSTVEK.T | IGLV2-14 protein | BCP & HP |
| A.ASSYLSLTPEQWK.S | IGLV2-14 protein | BCP & HP |
| K.NTLYLQMNSLR.A | Immunglobulin heavy chain variable region (Fragment) | BCP & HP |
| R.DNSKNTLYLQMNSLR.A | Immunglobulin heavy chain variable region (Fragment) | BCP & HP |
| R.LEDTAVYYCAK.K | Immunglobulin heavy chain variable region (Fragment) | BCP & HP |
| K.NTLYLQMNSLR.A | Immunglobulin heavy chain variable region (Fragment) | BCP & HP |
| R.AEDAAVYYCAR.D | Immunglobulin heavy chain variable region (Fragment) | BCP & HP |
| R.DNSKNTLYLQMNSLR.A | Immunglobulin heavy chain variable region (Fragment) | BCP & HP |
| R.FVYHLSDLCKK.C | immunoglobulin J chain | BCP & HP |
| R.FVYHLSDLCK.K | immunoglobulin J chain | BCP & HP |
| K.MVETALTPDACYPD.- | immunoglobulin J chain | BCP & HP |
| K.M*VETALTPDACYPD.- | immunoglobulin J chain | BCP & HP |
| K.CYTAVVPLVYGGETK.M | immunoglobulin J chain | BCP & HP |
| K.CDPTEVELDNQIVTATQSNICDEDSATETCYTYDR.N | immunoglobulin J chain | BCP & HP |
| R.SSEDPNEDIVER.N | immunoglobulin J chain | BCP & HP |
| R.NKCYTAVVPLVYGGETK.M | immunoglobulin J chain | BCP & HP |
| R.ALPGTPVASSQPR.F | Immunoglobulin superfamily containing leucine-rich repeat protein precursor | BCP & HP |
| R.EVPLLQSLWLAHNEIR.T | Immunoglobulin superfamily containing leucine-rich repeat protein precursor | BCP & HP |
| R.TVAAGALASLSHLK.S | Immunoglobulin superfamily containing leucine-rich repeat protein precursor | BCP & HP |
| K.ANTAAGTTGGGSCCVPTAR.R | Inhibin beta C chain precursor | BCP & HP |
| R.AGGQCPACGGPTLELESQR.E | Inhibin beta C chain precursor | BCP & HP |
| K.ANNPWPASTSCCVPTAR.R | Inhibin beta E chain precursor | BCP & HP |
| K.RSIMMKQLQQQQPNDKTNIQGM*TGTPMVAASPER.H | Insulin gene enhancer protein ISL-1 | BCP & HP |
| R.GFYFNKPTGYGSSSR.R | Insulin-like growth factor IB precursor | BCP & HP |
| R.RLEMYCAPLKPAK.S | Insulin-like growth factor IB precursor | BCP & HP |
| R.RAPQTGIVDECCFR.S | Insulin-like growth factor IB precursor | BCP & HP |
| A.GPETLCGAELVDALQFVCGDR.G | Insulin-like growth factor IB precursor | BCP & HP |
| R.KGWPKTHPGGEQKEGTEASLQIRGKK.K | Insulin-like growth factor IB precursor | BCP & HP |
| R.LEMYCAPLKPAK.S | Insulin-like growth factor IB precursor | BCP & HP |
| R.EPGCGCCSVCAR.L | Insulin-like growth factor-binding protein 2 precursor | BCP & HP |
| R.TPCQQELDQVLER.I | Insulin-like growth factor-binding protein 2 precursor | BCP & HP |
| R.MPCAELVREPGCGCCSVCAR.L | Insulin-like growth factor-binding protein 2 precursor | BCP & HP |
| R.LEGEACGVYTPR.C | Insulin-like growth factor-binding protein 2 precursor | BCP & HP |
| R.LAACGPPPVAPPAAVAAVAGGAR.M | Insulin-like growth factor-binding protein 2 precursor | BCP & HP |
| R.CYPHPGSELPLQALVMGEGTCEK.R | Insulin-like growth factor-binding protein 2 precursor | BCP & HP |
| R.REM*EDTLNHLK.F | Insulin-like growth factor-binding protein 3 precursor | BCP & HP |
| K.YGQPLPGYTTK.G | Insulin-like growth factor-binding protein 3 precursor | BCP & HP |
| Y.KVDYESQSTDTQNFSSESKR.E | Insulin-like growth factor-binding protein 3 precursor | BCP & HP |
| R.SAGSVESPSVSSTHR.V | Insulin-like growth factor-binding protein 3 precursor | BCP & HP |
| K.RGFCWCVDKYGQPLPGYTTK.G | Insulin-like growth factor-binding protein 3 precursor | BCP & HP |
| K.FLNVLSPR.G | Insulin-like growth factor-binding protein 3 precursor | BCP & HP |
| R.ALAQCAPPPAVCAELVREPGCGCCLTCALSEGQPCGIYTER.C | Insulin-like growth factor-binding protein 3 precursor | BCP & HP |
| K.RETEYGPCR.R | Insulin-like growth factor-binding protein 3 precursor | BCP & HP |
| R.GFCWCVDKYGQPLPGYTTK.G | Insulin-like growth factor-binding protein 3 precursor | BCP & HP |
| R.ALAQCAPPPAVCAELVR.E | Insulin-like growth factor-binding protein 3 precursor | BCP & HP |
| R.EPGCGCCLTCALSEGQPCGIYTER.C | Insulin-like growth factor-binding protein 3 precursor | BCP & HP |
| R.CQPSPDEARPLQALLDGR.G | Insulin-like growth factor-binding protein 3 precursor | BCP & HP |
| R.EM*EDTLNHLK.F | Insulin-like growth factor-binding protein 3 precursor | BCP & HP |
| R.EMEDTLNHLK.F | Insulin-like growth factor-binding protein 3 precursor | BCP & HP |
| K.GKEDVHCYSMQS.K | Insulin-like growth factor-binding protein 3 precursor | BCP & HP |
| R.THEDLYIIPIPNCDR.N | Insulin-like growth factor-binding protein 4 precursor | BCP & HP |
| K.QCHPALDGQR.G | Insulin-like growth factor-binding protein 4 precursor | BCP & HP |
| R.EPGCGCCATCALGLGMPCGVYTPR.C | Insulin-like growth factor-binding protein 4 precursor | BCP & HP |
| R.CRPPVGCEELVREPGCGCCATCALGLGMPCGVYTPR.C | Insulin-like growth factor-binding protein 4 precursor | BCP & HP |
| R.EDARPVPQGSCQSELHR.A | Insulin-like growth factor-binding protein 4 precursor | BCP & HP |
| R.ISELKAEAVKK.D | Insulin-like growth factor-binding protein 5 precursor | BCP & HP |
| K.ALSMCPPSPLGCELVK.E | Insulin-like growth factor-binding protein 5 precursor | BCP & HP |
| R.HMEASLQELK.A | Insulin-like growth factor-binding protein 5 precursor | BCP & HP |
| R.ISELKAEAVK.K | Insulin-like growth factor-binding protein 5 precursor | BCP & HP |
| K.FVGGAENTAHPR.I | Insulin-like growth factor-binding protein 5 precursor | BCP & HP |
| R.RGPCWCVDR.M | Insulin-like growth factor-binding protein 6 precursor | BCP & HP |
| R.GAQTLYVPNCDHR.G | Insulin-like growth factor-binding protein 6 precursor | BCP & HP |
| R.HLDSVLQQLQTEVYR.G | Insulin-like growth factor-binding protein 6 precursor | BCP & HP |
| R.DACGCCPM*CAR.G | Insulin-like growth factor-binding protein 7 precursor | BCP & HP |
| K.EDAGEYECHASNSQGQASASAK.I | Insulin-like growth factor-binding protein 7 precursor | BCP & HP |
| R.DACGCCPMCAR.G | Insulin-like growth factor-binding protein 7 precursor | BCP & HP |
| K.AGAAAGGPGVSGVCVCK.S | Insulin-like growth factor-binding protein 7 precursor | BCP & HP |
| K.SRYPVCGSDGTTYPSGCQLR.A | Insulin-like growth factor-binding protein 7 precursor | BCP & HP |
| K.GKAGAAAGGPGVSGVCVCK.S | Insulin-like growth factor-binding protein 7 precursor | BCP & HP |
| R.DFALQNPSAVPR.F | Insulin-like growth factor-binding protein complex acid labile chain precursor | BCP & HP |
| A.GLLEDTFPGLLGLR.V | Insulin-like growth factor-binding protein complex acid labile chain precursor | BCP & HP |
| G.ADPGTPGEAEGPACPAACVCSYDDDADELSVFCSSR.N | Insulin-like growth factor-binding protein complex acid labile chain precursor | BCP & HP |
| K.ALRDFALQNPSAVPR.F | Insulin-like growth factor-binding protein complex acid labile chain precursor | BCP & HP |
| K.ANVFVQLPR.L | Insulin-like growth factor-binding protein complex acid labile chain precursor | BCP & HP |
| K.DLHFLEELQLGHNR.I | Insulin-like growth factor-binding protein complex acid labile chain precursor | BCP & HP |
| K.LEYLLLSR.N | Insulin-like growth factor-binding protein complex acid labile chain precursor | BCP & HP |
| K.LHSLHLEGSCLGR.I | Insulin-like growth factor-binding protein complex acid labile chain precursor | BCP & HP |
| K.LYLDRNLIAAVAPGAFLGLK.A | Insulin-like growth factor-binding protein complex acid labile chain precursor | BCP & HP |
| R.AFWLDVSHNRLEALPNSLLAPLGR.L | Insulin-like growth factor-binding protein complex acid labile chain precursor | BCP & HP |
| R.DLSEAHFAPC.- | Insulin-like growth factor-binding protein complex acid labile chain precursor | BCP & HP |
| R.ELVLAGNR.L | Insulin-like growth factor-binding protein complex acid labile chain precursor | BCP & HP |
| R.GLGSLRELVLAGNR.L | Insulin-like growth factor-binding protein complex acid labile chain precursor | BCP & HP |
| R.LAELPADALGPLQR.A | Insulin-like growth factor-binding protein complex acid labile chain precursor | BCP & HP |
| R.TFKDLHFLEELQLGHNR.I | Insulin-like growth factor-binding protein complex acid labile chain precursor | BCP & HP |
| R.LAYLQPALFSGLAELRELDLSR.N | Insulin-like growth factor-binding protein complex acid labile chain precursor | BCP & HP |
| R.AFWLDVSHNR.L | Insulin-like growth factor-binding protein complex acid labile chain precursor | BCP & HP |
| R.TFTPQPPGLER.L | Insulin-like growth factor-binding protein complex acid labile chain precursor | BCP & HP |
| R.LEALPNSLLAPLGR.L | Insulin-like growth factor-binding protein complex acid labile chain precursor | BCP & HP |
| R.WLDLSHNR.V | Insulin-like growth factor-binding protein complex acid labile chain precursor | BCP & HP |
| R.SLALGTFAHTPALASLGLSNNR.L | Insulin-like growth factor-binding protein complex acid labile chain precursor | BCP & HP |
| R.SFEGLGQLEVLTLDHNQLQEVK.A | Insulin-like growth factor-binding protein complex acid labile chain precursor | BCP & HP |
| R.NRLAELPADALGPLQR.A | Insulin-like growth factor-binding protein complex acid labile chain precursor | BCP & HP |
| R.LWLEGNPWDCGCPLK.A | Insulin-like growth factor-binding protein complex acid labile chain precursor | BCP & HP |
| R.LAYLQPALFSGLAELR.E | Insulin-like growth factor-binding protein complex acid labile chain precursor | BCP & HP |
| R.LSHNAIASLRPR.T | Insulin-like growth factor-binding protein complex acid labile chain precursor | BCP & HP |
| R.LQKLYLDR.N | Insulin-like growth factor-binding protein complex acid labile chain precursor | BCP & HP |
| R.LFQGLGKLEYLLLSR.N | Insulin-like growth factor-binding protein complex acid labile chain precursor | BCP & HP |
| R.NLIAAVAPGAFLGLK.A | Insulin-like growth factor-binding protein complex acid labile chain precursor | BCP & HP |
| R.VAGLLEDTFPGLLGLR.V | Insulin-like growth factor-binding protein complex acid labile chain precursor | BCP & HP |
| K.TRRTQKSSLFLPRVLDHR.D | INT-2 proto-oncogene protein precursor | BCP & HP |
| R.RTQKSSLFLPR.V | INT-2 proto-oncogene protein precursor | BCP & HP |
| I.GGLLLLALITAVLYKVGFFKR.Q | Integrin alpha-X precursor | BCP & HP |
| K.LKPEDITQIQPQQLVLR.L | integrin beta 1 isoform 1A precursor | BCP & HP |
| V.DLQYPQDAVLALTQNHHK.Q | Inter-alpha-trypsin inhibitor heavy chain H1 precursor | BCP & HP |
| K.RQAVDTAVDGVFIR.S | Inter-alpha-trypsin inhibitor heavy chain H1 precursor | BCP & HP |
| R.VQSWKGSLVQASEANLQAAQDFVR.G | Inter-alpha-trypsin inhibitor heavy chain H1 precursor | BCP & HP |
| Q.MSLDYGFVTPLTSM*SIR.G | Inter-alpha-trypsin inhibitor heavy chain H1 precursor | BCP & HP |
| R.FAHYVVTSQVVNTANEAREVAFDLEIPK.T | Inter-alpha-trypsin inhibitor heavy chain H1 precursor | BCP & HP |
| K.QYYEGSEIVVAGR.I | Inter-alpha-trypsin inhibitor heavy chain H1 precursor | BCP & HP |
| K.TAFISDFAVTADGNAFIGDIK.D | Inter-alpha-trypsin inhibitor heavy chain H1 precursor | BCP & HP |
| K.TAFISDFAVTADGNAFIGDIKDKVTAWK.Q | Inter-alpha-trypsin inhibitor heavy chain H1 precursor | BCP & HP |
| K.VTFQLTYEEVLK.R | Inter-alpha-trypsin inhibitor heavy chain H1 precursor | BCP & HP |
| K.VTFQLTYEEVLKR.N | Inter-alpha-trypsin inhibitor heavy chain H1 precursor | BCP & HP |
| K.VTYDVSR.D | Inter-alpha-trypsin inhibitor heavy chain H1 precursor | BCP & HP |
| K.VTYDVSRDK.I | Inter-alpha-trypsin inhibitor heavy chain H1 precursor | BCP & HP |
| K.QSSFKADVQAHGEGQEFSITCLVDEEEMKK.L | Inter-alpha-trypsin inhibitor heavy chain H1 precursor | BCP & HP |
| Q.M*SLDYGFVTPLTSMSIR.G | Inter-alpha-trypsin inhibitor heavy chain H1 precursor | BCP & HP |
| K.QLVHHFEIDVDIFEPQGISK.L | Inter-alpha-trypsin inhibitor heavy chain H1 precursor | BCP & HP |
| Q.MSLDYGFVTPLTSMSIR.G | Inter-alpha-trypsin inhibitor heavy chain H1 precursor | BCP & HP |
| Q.YPQDAVLALTQNHHK.Q | Inter-alpha-trypsin inhibitor heavy chain H1 precursor | BCP & HP |
| R.ANLSSQALQM*SLDYGFVTPLTSMSIR.G | Inter-alpha-trypsin inhibitor heavy chain H1 precursor | BCP & HP |
| R.ANLSSQALQMSLDYGFVTPLTSMSIR.G | Inter-alpha-trypsin inhibitor heavy chain H1 precursor | BCP & HP |
| R.DQAVLRQDGVVVTINK.K | Inter-alpha-trypsin inhibitor heavy chain H1 precursor | BCP & HP |
| R.ERGHM*LENHVER.L | Inter-alpha-trypsin inhibitor heavy chain H1 precursor | BCP & HP |
| R.ERGHMLENHVER.L | Inter-alpha-trypsin inhibitor heavy chain H1 precursor | BCP & HP |
| R.EVAFDLEIPK.T | Inter-alpha-trypsin inhibitor heavy chain H1 precursor | BCP & HP |
| K.TAFISDFAVTADGNAFIGDIKDK.V | Inter-alpha-trypsin inhibitor heavy chain H1 precursor | BCP & HP |
| Q.M*SLDYGFVTPLTSM*SIR.G | Inter-alpha-trypsin inhibitor heavy chain H1 precursor | BCP & HP |
| K.GHVLFRPTVSQQQSCPTCSTSLLNGHFK.V | Inter-alpha-trypsin inhibitor heavy chain H1 precursor | BCP & HP |
| A.HYVVTSQVVNTANEAR.E | Inter-alpha-trypsin inhibitor heavy chain H1 precursor | BCP & HP |
| H.VLFRPTVSQQQSCPTCSTSLLNGHFK.V | Inter-alpha-trypsin inhibitor heavy chain H1 precursor | BCP & HP |
| K.AAISGENAGLVR.A | Inter-alpha-trypsin inhibitor heavy chain H1 precursor | BCP & HP |
| K.ADVQAHGEGQEFSITCLVDEEEM*K.K | Inter-alpha-trypsin inhibitor heavy chain H1 precursor | BCP & HP |
| K.ADVQAHGEGQEFSITCLVDEEEM*KK.L | Inter-alpha-trypsin inhibitor heavy chain H1 precursor | BCP & HP |
| K.ADVQAHGEGQEFSITCLVDEEEMK.K | Inter-alpha-trypsin inhibitor heavy chain H1 precursor | BCP & HP |
| K.ADVQAHGEGQEFSITCLVDEEEMKK.L | Inter-alpha-trypsin inhibitor heavy chain H1 precursor | BCP & HP |
| K.DKVTAWK.Q | Inter-alpha-trypsin inhibitor heavy chain H1 precursor | BCP & HP |
| K.QYRKAAISGENAGLVR.A | Inter-alpha-trypsin inhibitor heavy chain H1 precursor | BCP & HP |
| K.ELAAQTIKK.S | Inter-alpha-trypsin inhibitor heavy chain H1 precursor | BCP & HP |
| R.FPLYNLGFGHNVDFNFLEVMSM*ENNGR.A | Inter-alpha-trypsin inhibitor heavy chain H1 precursor | BCP & HP |
| K.GSLVQASEANLQAAQDFVR.G | Inter-alpha-trypsin inhibitor heavy chain H1 precursor | BCP & HP |
| K.GSLVQASEANLQAAQDFVRGFSLDEATNLNGGLLR.G | Inter-alpha-trypsin inhibitor heavy chain H1 precursor | BCP & HP |
| K.ICDLLVANNHFAHFFAPQNLTNMNK.N | Inter-alpha-trypsin inhibitor heavy chain H1 precursor | BCP & HP |
| K.ILGDM*QPGDYFDLVLFGTR.V | Inter-alpha-trypsin inhibitor heavy chain H1 precursor | BCP & HP |
| K.ILGDMQPGDYFDLVLFGTR.V | Inter-alpha-trypsin inhibitor heavy chain H1 precursor | BCP & HP |
| K.LDAQASFLPK.E | Inter-alpha-trypsin inhibitor heavy chain H1 precursor | BCP & HP |
| K.NVVFVIDISGSM*R.G | Inter-alpha-trypsin inhibitor heavy chain H1 precursor | BCP & HP |
| K.NVVFVIDISGSMR.G | Inter-alpha-trypsin inhibitor heavy chain H1 precursor | BCP & HP |
| K.PLLVDVDLQYPQDAVLALTQNHHK.Q | Inter-alpha-trypsin inhibitor heavy chain H1 precursor | BCP & HP |
| K.ELAAQTIK.K | Inter-alpha-trypsin inhibitor heavy chain H1 precursor | BCP & HP |
| R.FAHYVVTSQVVNTANEAR.E | Inter-alpha-trypsin inhibitor heavy chain H1 precursor | BCP & HP |
| R.IYEDHDATQQLQGFYSQVAK.P | Inter-alpha-trypsin inhibitor heavy chain H1 precursor | BCP & HP |
| R.KAAISGENAGLVR.A | Inter-alpha-trypsin inhibitor heavy chain H1 precursor | BCP & HP |
| R.LWAYLTIQELLAK.R | Inter-alpha-trypsin inhibitor heavy chain H1 precursor | BCP & HP |
| R.LWAYLTIQELLAKR.M | Inter-alpha-trypsin inhibitor heavy chain H1 precursor | BCP & HP |
| R.LWAYLTIQELLAKRMKVDR.E | Inter-alpha-trypsin inhibitor heavy chain H1 precursor | BCP & HP |
| R.IADNKQSSFK.A | Inter-alpha-trypsin inhibitor heavy chain H1 precursor | BCP & HP |
| R.NHMQYEIVIK.V | Inter-alpha-trypsin inhibitor heavy chain H1 precursor | BCP & HP |
| R.NHM*QYEIVIK.V | Inter-alpha-trypsin inhibitor heavy chain H1 precursor | BCP & HP |
| R.QAVDTAVDGVFIR.S | Inter-alpha-trypsin inhibitor heavy chain H1 precursor | BCP & HP |
| R.QDGVVVTINK.K | Inter-alpha-trypsin inhibitor heavy chain H1 precursor | BCP & HP |
| R.RTFVLSALQPSPTHSSSNTQR.L | Inter-alpha-trypsin inhibitor heavy chain H1 precursor | BCP & HP |
| R.SPGQHDGTYFGR.L | Inter-alpha-trypsin inhibitor heavy chain H1 precursor | BCP & HP |
| R.TFVLSALQPSPTHSSSNTQR.L | Inter-alpha-trypsin inhibitor heavy chain H1 precursor | BCP & HP |
| R.TM*EQFTIHLTVNPQSK.V | Inter-alpha-trypsin inhibitor heavy chain H1 precursor | BCP & HP |
| R.TMEQFTIHLTVNPQSK.V | Inter-alpha-trypsin inhibitor heavy chain H1 precursor | BCP & HP |
| R.GIEILNQVQESLPELSNHASILIMLTDGDPTEGVTDR.S | Inter-alpha-trypsin inhibitor heavy chain H1 precursor | BCP & HP |
| R.GHM*LENHVER.L | Inter-alpha-trypsin inhibitor heavy chain H1 precursor | BCP & HP |
| R.PTVSQQQSCPTCSTSLLNGHFK.V | Inter-alpha-trypsin inhibitor heavy chain H1 precursor | BCP & HP |
| R.GFSLDEATNLNGGLLR.G | Inter-alpha-trypsin inhibitor heavy chain H1 precursor | BCP & HP |
| R.GRFPLYNLGFGHNVDFNFLEVMSMENNGR.A | Inter-alpha-trypsin inhibitor heavy chain H1 precursor | BCP & HP |
| R.FPLYNLGFGHNVDFNFLEVMSMENNGR.A | Inter-alpha-trypsin inhibitor heavy chain H1 precursor | BCP & HP |
| R.GIEILNQVQESLPELSNHASILIM*LTDGDPTEGVTDR.S | Inter-alpha-trypsin inhibitor heavy chain H1 precursor | BCP & HP |
| R.GM*ADQDGLKPTIDKPSEDSPPLEM*LGPR.R | Inter-alpha-trypsin inhibitor heavy chain H1 precursor | BCP & HP |
| R.GM*ADQDGLKPTIDKPSEDSPPLEMLGPR.R | Inter-alpha-trypsin inhibitor heavy chain H1 precursor | BCP & HP |
| R.GMADQDGLKPTIDKPSEDSPPLEM*LGPR.R | Inter-alpha-trypsin inhibitor heavy chain H1 precursor | BCP & HP |
| R.GMADQDGLKPTIDKPSEDSPPLEMLGPR.R | Inter-alpha-trypsin inhibitor heavy chain H1 precursor | BCP & HP |
| R.GMADQDGLKPTIDKPSEDSPPLEMLGPRR.T | Inter-alpha-trypsin inhibitor heavy chain H1 precursor | BCP & HP |
| R.GRFPLYNLGFGHNVDFNFLEVM*SM*ENNGR.A | Inter-alpha-trypsin inhibitor heavy chain H1 precursor | BCP & HP |
| R.GRFPLYNLGFGHNVDFNFLEVM*SMENNGR.A | Inter-alpha-trypsin inhibitor heavy chain H1 precursor | BCP & HP |
| R.GRFPLYNLGFGHNVDFNFLEVMSM*ENNGR.A | Inter-alpha-trypsin inhibitor heavy chain H1 precursor | BCP & HP |
| R.GHMLENHVER.L | Inter-alpha-trypsin inhibitor heavy chain H1 precursor | BCP & HP |
| K.RLSNENHGIAQR.I | Inter-alpha-trypsin inhibitor heavy chain H2 precursor | BCP & HP |
| K.LWAYLTINQLLAER.S | Inter-alpha-trypsin inhibitor heavy chain H2 precursor | BCP & HP |
| K.M*KQTVEAM*K.T | Inter-alpha-trypsin inhibitor heavy chain H2 precursor | BCP & HP |
| K.M*KQTVEAMK.T | Inter-alpha-trypsin inhibitor heavy chain H2 precursor | BCP & HP |
| K.MKQTVEAM*K.T | Inter-alpha-trypsin inhibitor heavy chain H2 precursor | BCP & HP |
| K.MKQTVEAMK.T | Inter-alpha-trypsin inhibitor heavy chain H2 precursor | BCP & HP |
| K.NILFVIDVSGSMWGVK.M | Inter-alpha-trypsin inhibitor heavy chain H2 precursor | BCP & HP |
| K.NVKENIQDNISLFSLGM*GFDVDYDFLK.R | Inter-alpha-trypsin inhibitor heavy chain H2 precursor | BCP & HP |
| K.TAGLVR.S | Inter-alpha-trypsin inhibitor heavy chain H2 precursor | BCP & HP |
| K.QTVEAMKTILDDLRAEDHFSVIDFNQNIR.T | Inter-alpha-trypsin inhibitor heavy chain H2 precursor | BCP & HP |
| K.LGSYEHR.I | Inter-alpha-trypsin inhibitor heavy chain H2 precursor | BCP & HP |
| K.TQVADAKR.Y | Inter-alpha-trypsin inhibitor heavy chain H2 precursor | BCP & HP |
| K.TILDDLRAEDHFSVIDFNQNIR.T | Inter-alpha-trypsin inhibitor heavy chain H2 precursor | BCP & HP |
| K.NVKENIQDNISLFSLGM*GFDVDYDFLKR.L | Inter-alpha-trypsin inhibitor heavy chain H2 precursor | BCP & HP |
| K.LDQIESVITATSANTQLVLETLAQMDDLQDFLSK.D | Inter-alpha-trypsin inhibitor heavy chain H2 precursor | BCP & HP |
| K.LDQIESVITATSANTQLVLETLAQM*DDLQDFLSK.D | Inter-alpha-trypsin inhibitor heavy chain H2 precursor | BCP & HP |
| K.KFYNQVSTPLLR.N | Inter-alpha-trypsin inhibitor heavy chain H2 precursor | BCP & HP |
| K.IQPSGGTNINEALLR.A | Inter-alpha-trypsin inhibitor heavy chain H2 precursor | BCP & HP |
| K.HLEVDVWVIEPQGLR.F | Inter-alpha-trypsin inhibitor heavy chain H2 precursor | BCP & HP |
| K.HADPDFTR.K | Inter-alpha-trypsin inhibitor heavy chain H2 precursor | BCP & HP |
| K.FYNQVSTPLLR.N | Inter-alpha-trypsin inhibitor heavy chain H2 precursor | BCP & HP |
| K.FDPAKLDQIESVITATSANTQLVLETLAQMDDLQDFLSKDK.H | Inter-alpha-trypsin inhibitor heavy chain H2 precursor | BCP & HP |
| K.FDPAKLDQIESVITATSANTQLVLETLAQMDDLQDFLSK.D | Inter-alpha-trypsin inhibitor heavy chain H2 precursor | BCP & HP |
| K.FDPAKLDQIESVITATSANTQLVLETLAQM*DDLQDFLSKDK.H | Inter-alpha-trypsin inhibitor heavy chain H2 precursor | BCP & HP |
| K.FDPAKLDQIESVITATSANTQLVLETLAQM*DDLQDFLSK.D | Inter-alpha-trypsin inhibitor heavy chain H2 precursor | BCP & HP |
| K.ENIQDNISLFSLGM*GFDVDYDFLKR.L | Inter-alpha-trypsin inhibitor heavy chain H2 precursor | BCP & HP |
| K.VQFELHYQEVK.W | Inter-alpha-trypsin inhibitor heavy chain H2 precursor | BCP & HP |
| R.SLPGESEEMM*EEVDQVTLYSYK.V | Inter-alpha-trypsin inhibitor heavy chain H2 precursor | BCP & HP |
| K.ENIQDNISLFSLGMGFDVDYDFLKR.L | Inter-alpha-trypsin inhibitor heavy chain H2 precursor | BCP & HP |
| R.KLWAYLTINQLLAER.S | Inter-alpha-trypsin inhibitor heavy chain H2 precursor | BCP & HP |
| K.VQSTITSR.M | Inter-alpha-trypsin inhibitor heavy chain H2 precursor | BCP & HP |
| R.SLPGESEEM*M*EEVDQVTLYSYK.V | Inter-alpha-trypsin inhibitor heavy chain H2 precursor | BCP & HP |
| K.DKHADPDFTR.K | Inter-alpha-trypsin inhibitor heavy chain H2 precursor | BCP & HP |
| R.SLAPTAAAK.R | Inter-alpha-trypsin inhibitor heavy chain H2 precursor | BCP & HP |
| R.SILQMSLDHHIVTPLTSLVIENEAGDER.M | Inter-alpha-trypsin inhibitor heavy chain H2 precursor | BCP & HP |
| R.SILQM*SLDHHIVTPLTSLVIENEAGDER.M | Inter-alpha-trypsin inhibitor heavy chain H2 precursor | BCP & HP |
| R.NVQFNYPHTSVTDVTQNNFHNYFGGSEIVVAGK.F | Inter-alpha-trypsin inhibitor heavy chain H2 precursor | BCP & HP |
| R.MLADAPPQDPSCCSGALYYGSK.V | Inter-alpha-trypsin inhibitor heavy chain H2 precursor | BCP & HP |
| R.MATTMIQSK.V | Inter-alpha-trypsin inhibitor heavy chain H2 precursor | BCP & HP |
| R.MATTM*IQSK.V | Inter-alpha-trypsin inhibitor heavy chain H2 precursor | BCP & HP |
| R.M*LADAPPQDPSCCSGALYYGSK.V | Inter-alpha-trypsin inhibitor heavy chain H2 precursor | BCP & HP |
| R.M*ATTMIQSK.V | Inter-alpha-trypsin inhibitor heavy chain H2 precursor | BCP & HP |
| R.SLAPTAAAKR.R | Inter-alpha-trypsin inhibitor heavy chain H2 precursor | BCP & HP |
| R.LSNENHGIAQR.I | Inter-alpha-trypsin inhibitor heavy chain H2 precursor | BCP & HP |
| K.VVNNSPQPQNVVFDVQIPK.G | Inter-alpha-trypsin inhibitor heavy chain H2 precursor | BCP & HP |
| R.KLGSYEHR.I | Inter-alpha-trypsin inhibitor heavy chain H2 precursor | BCP & HP |
| R.IYLQPGR.L | Inter-alpha-trypsin inhibitor heavy chain H2 precursor | BCP & HP |
| R.IYGNQDTSSQLKK.F | Inter-alpha-trypsin inhibitor heavy chain H2 precursor | BCP & HP |
| R.IYGNQDTSSQLK.K | Inter-alpha-trypsin inhibitor heavy chain H2 precursor | BCP & HP |
| R.FLHVPDTFEGHFDGVPVISK.G | Inter-alpha-trypsin inhibitor heavy chain H2 precursor | BCP & HP |
| R.ETAVDGELVVLYDVKREEK.A | Inter-alpha-trypsin inhibitor heavy chain H2 precursor | BCP & HP |
| R.ETAVDGELVVLYDVKR.E | Inter-alpha-trypsin inhibitor heavy chain H2 precursor | BCP & HP |
| R.ETAVDGELVVLYDVK.R | Inter-alpha-trypsin inhibitor heavy chain H2 precursor | BCP & HP |
| R.EEKAGELEVFNGYFVHFFAPDNLDPIPK.N | Inter-alpha-trypsin inhibitor heavy chain H2 precursor | BCP & HP |
| R.AIFILNEANNLGLLDPNSVSLIILVSDGDPTVGELK.L | Inter-alpha-trypsin inhibitor heavy chain H2 precursor | BCP & HP |
| R.AEDHFSVIDFNQNIR.T | Inter-alpha-trypsin inhibitor heavy chain H2 precursor | BCP & HP |
| Q.PSGGTNINEALLR.A | Inter-alpha-trypsin inhibitor heavy chain H2 precursor | BCP & HP |
| R.M*ATTM*IQSK.V | Inter-alpha-trypsin inhibitor heavy chain H2 precursor | BCP & HP |
| S.PQPQNVVFDVQIPK.G | Inter-alpha-trypsin inhibitor heavy chain H2 precursor | BCP & HP |
| R.SSALDM*ENFR.T | Inter-alpha-trypsin inhibitor heavy chain H2 precursor | BCP & HP |
| R.SSALDMENFR.T | Inter-alpha-trypsin inhibitor heavy chain H2 precursor | BCP & HP |
| R.SSALDMENFRTEVNVLPGAK.V | Inter-alpha-trypsin inhibitor heavy chain H2 precursor | BCP & HP |
| R.TEVNVLPGAK.V | Inter-alpha-trypsin inhibitor heavy chain H2 precursor | BCP & HP |
| M.SLDHHIVTPLTSLVIENEAGDER.M | Inter-alpha-trypsin inhibitor heavy chain H2 precursor | BCP & HP |
| R.SLPGESEEMMEEVDQVTLYSYK.V | Inter-alpha-trypsin inhibitor heavy chain H2 precursor | BCP & HP |
| R.TWRNDLISATK.T | Inter-alpha-trypsin inhibitor heavy chain H2 precursor | BCP & HP |
| K.AHVSFKPTVAQQR.I | Inter-alpha-trypsin inhibitor heavy chain H2 precursor | BCP & HP |
| A.DAPPQDPSCCSGALYYGSK.V | Inter-alpha-trypsin inhibitor heavy chain H2 precursor | BCP & HP |
| A.PPQDPSCCSGALYYGSK.V | Inter-alpha-trypsin inhibitor heavy chain H2 precursor | BCP & HP |
| D.APPQDPSCCSGALYYGSK.V | Inter-alpha-trypsin inhibitor heavy chain H2 precursor | BCP & HP |
| D.PSCCSGALYYGSK.V | Inter-alpha-trypsin inhibitor heavy chain H2 precursor | BCP & HP |
| K.AGELEVFNGYFVHFFAPDNLDPIPK.N | Inter-alpha-trypsin inhibitor heavy chain H2 precursor | BCP & HP |
| K.ASVSVTAEDEGTQR.L | Intercellular adhesion molecule 1 precursor | BCP & HP |
| K.CLKDGTFPLPIGESVTVTR.D | Intercellular adhesion molecule 1 precursor | BCP & HP |
| K.ELKREPAVGEPAEVTTTVLVR.R | Intercellular adhesion molecule 1 precursor | BCP & HP |
| K.VTLNGVPAQPLGPR.A | Intercellular adhesion molecule 1 precursor | BCP & HP |
| R.GGSVLVTCSTSCDQPK.L | Intercellular adhesion molecule 1 precursor | BCP & HP |
| R.DLEGTYLCR.A | Intercellular adhesion molecule 1 precursor | BCP & HP |
| R.QVILTLQPTLVAVGK.S | Intercellular adhesion molecule 2 precursor | BCP & HP |
| R.VPTVEPLDSLTLFLFR.G | Intercellular adhesion molecule 2 precursor | BCP & HP |
| R.EPAPAEALPQQYPEPAPAALCGPPPRAPSR.N | Interferon regulatory factor 2-binding protein 1 | BCP & HP |
| R.EFNSYGARR.G | Iron-responsive element-binding protein 2 | BCP & HP |
| K.EKSNFSLAILNVGAPAAGMNAAVRSAVR.T | Isoform 1 of 6-phosphofructokinase, liver type | BCP & HP |
| R.RLLPGPQENSVQSSACGR.Q | Isoform 1 of ADAMTS-13 precursor | BCP & HP |
| R.SSPGGASFYHWGAAVPHSQGDALCR.H | Isoform 1 of ADAMTS-13 precursor | BCP & HP |
| R.RQLLSLLSAGR.A | Isoform 1 of ADAMTS-13 precursor | BCP & HP |
| R.LLVPLLDGTECGVEK.W | Isoform 1 of ADAMTS-13 precursor | BCP & HP |
| R.LLPGPQENSVQSSACGR.Q | Isoform 1 of ADAMTS-13 precursor | BCP & HP |
| R.IWGPLQEDADIQVYR.R | Isoform 1 of ADAMTS-13 precursor | BCP & HP |
| R.EVCQAVPCPAR.W | Isoform 1 of ADAMTS-13 precursor | BCP & HP |
| R.AGAQQPAVALETCNPQPCPAR.W | Isoform 1 of ADAMTS-13 precursor | BCP & HP |
[truncated: 388,746 more chars]
