# Supplementary material for: Blood Peptidome-Degradome Profile of Breast Cancer
Source: PLoS One. 2010 Oct 18;5(10):e13133. doi: 10.1371/journal.pone.0013133 (PMC2956627; doi:10.1371/journal.pone.0013133)
Supplement: Table S2 — The peptidome peptides identified from the pooled breast cancer patients (BCP) and control healthy persons (HP) blood plasma samples. (0.95 MB DOC) [file pone.0013133.s002.doc]

Table S2. The peptidome peptides identified from the pooled breast cancer patients (BCP) and control healthy persons (HP) blood plasma samples.*

| **Peptidome peptide** | **Protein** | **Source** | **A_HP_** | **A_BCP_** |
| --- | --- | --- | --- | --- |
| P.NSPLDEENLTQENQDRGTHVDLGLASANVDFAFSLYK.Q | α-1-Antichymotrypsin | BCP | 4.37 | 5.85 |
| V.PTDTQNIFFMSKVTNPKQA.- | α-1-Antichymotrypsin | BCP&HP | 5.91 | 6.20 |
| R.FNRPFLMIIVPTDTQNIFFMSKVTNPKQA.- | α-1-Antichymotrypsin | BCP&HP | 6.00 | 6.26 |
| L.VETRTIVRFNRPFLMIIVPTDTQNIFFMSKVTNPKQA.- | α-1-Antichymotrypsin | BCP&HP | 6.04 | 5.56 |
| R.TIVRFNRPFLMIIVPTDTQNIFFMSKVTNPKQA.- | α-1-Antichymotrypsin | BCP&HP | 6.23 | 6.51 |
| V.ETRTIVRFNRPFLMIIVPTDTQNIFFMSKVTNPKQA.- | α-1-Antichymotrypsin | BCP&HP | 6.20 | 5.98 |
| S.ALVETRTIVRFNRPFLMIIVPTDTQNIFFMSKVTNPKQA.- | α-1-Antichymotrypsin | BCP&HP | 6.49 | 6.58 |
| A.LVETRTIVRFNRPFLMIIVPTDTQNIFFMSKVTNPKQA.- | α-1-Antichymotrypsin | BCP&HP | 7.00 | 7.15 |
| L.SALVETRTIVRFNRPFLMIIVPTDTQNIFFMSKVTNPKQA.- | α-1-Antichymotrypsin | BCP&HP | 7.93 | 8.40 |
| C.HPNSPLDEENLTQENQDRGTHVDLGLASANVDFAFSLYK.Q | α-1-Antichymotrypsin | BCP&HP | 5.74 | 6.67 |
| I.FFMSKVTNPKQA.- | α-1-Antichymotrypsin | BCP&HP | 5.88 | 6.43 |
| C.HPNSPLDEENLTQENQDR.G | α-1-Antichymotrypsin | HP | 6.91 | 4.13 |
| E.TRTIVRFNRPFLMIIVPTDTQNIFFMSKVTNPKQA.- | α-1-Antichymotrypsin | HP(BCP) | 5.56 | 5.49 |
| L.LSALVETRTIVRFNRPFLMIIVPTDTQNIFFMSKVTNPKQA.- | α-1-Antichymotrypsin | HP(BCP) | 4.96 | 5.45 |
| L.SALVETRTIVRFNRPFLM(−hydroxylation)IIVPTDTQNIFFMSKVTNP(−hydroxylation)KQA.- | α-1-Antichymotrypsin | HP(BCP) | 6.43 | 6.00 |
| K.AVLDVFEEGTEASAATAVKITLLSALVETRTIVRFNRPFLMIIVPTDTQNIFFMSKVTNPKQA.- | α-1-Antichymotrypsin | BCP | 4.44 | 6.26 |
| A.LVETRTIVRFNRPFLM(−hydroxylation)IIVPTDTQNIFFMSKVTNPKQA.- | α-1-Antichymotrypsin | BCP&HP | 6.43 | 6.15 |
| L.SALVETRTIVRFNRPFLM(−hydroxylation)IIVPTDTQNIFFMSKVTNPKQA.- | α-1-Antichymotrypsin | BCP&HP | 7.00 | 6.79 |
| L.SALVETRTIVRFNRPFLMIIVPTD(−dehydration)T.Q | α-1-Antichymotrypsin | BCP&HP | 6.28 | 5.40 |
| L.SALVETRTIVRFNRPFLMIIVPTDTQNIFFMSKVTNPK(−dehydration).Q | α-1-Antichymotrypsin | BCP&HP | 5.86 | 6.43 |
| L.SALVETRTIVRFNRPFLMIIVPTDTQNIFFMSKVTN(−dehydration).P | α-1-Antichymotrypsin | BCP&HP | 5.80 | 6.36 |
| L.SALVETRTIVRFNRPFLMIIVPTDTQ(−dehydrated).N | α-1-Antichymotrypsin | BCP&HP | 6.18 | 6.80 |
| L.SALVETRTIVRFNRPFLMIIVPTDTQN(−dehydration).I | α-1-Antichymotrypsin | BCP&HP | 6.40 | 6.99 |
| R.NLAVSQVVHKAVLDVFEEGTEASAATAVKITLLSALVETRTIVRFNRPFLMIIVPTDTQNIFFMSKVTNPKQA.- | α-1-Antichymotrypsin | HP | 6.57 | 4.62 |
| R.SWVPHTFESELSDPVELLVAES.- | α-1B-glycoprotein | HP | 5.38 | 4.40 |
| R.MSLSSFSVNRPFLFFIFEDTTGLPLFVGSVRNPNPSAPRELKEQQ.D | α-2-Antiplasmin | BCP | 4.26 | 6.60 |
| V.PPMEEDYPQFGSPK.- | α-2-Antiplasmin | BCP | 4.32 | 6.52 |
| R.GDKLFGPDLKLVPPMEEDYPQFGSPK.- | α-2-Antiplasmin | BCP | 4.33 | 7.40 |
| R.MSLSSFSVNRPFLFFIFEDTTGLPLFVGSVRNPNPSAPRELKEQQDSPGNKDFLQSLK.G | α-2-Antiplasmin | BCP | 4.36 | 6.49 |
| R.MSLSSFSVNRPFLFFIFEDTTGLPLFVGSVRNPNPSAPRELKEQQDSPGNKDFLQSLKGFPR.G | α-2-Antiplasmin | BCP | 4.35 | 6.40 |
| R.NPNPSAPRELKEQQDSPGNKDFLQSLKGFPR.G | α-2-Antiplasmin | BCP(HP) | 5.56 | 5.92 |
| R.NPNPSAPRELKEQQ.D | α-2-Antiplasmin | BCP(HP) | 4.52 | 6.28 |
| K.GFPRGDKLFGPDLKLVPPMEEDYPQFGSPK.- | α-2-Antiplasmin | BCP(HP) | 4.78 | 6.38 |
| R.GISEQSLVVSGVQHQSTLELSEVGVEAAAATSIAM(−hydroxylation)SR.M | α-2-Antiplasmin | HP(BCP) | 6.53 | 5.75 |
| K.GFPRGDKLFGPDLKLVPPM(−oxid)EEDYPQFGSPK.- | α-2-Antiplasmin | BCP&HP | 6.78 | 6.68 |
| R.M(−hydroxylation)SLSSFSVNRPFLFFIFEDTTGLPLFVGSVRNPNPSAPRELKEQQ.D | α-2-Antiplasmin | BCP&HP | 7.32 | 6.11 |
| R.M(−hydroxylation)SLSSFSVNRPFLFFIFEDTTGLPLFVGSVRNPNPSAPR(→K)ELKEQQ.D | α-2-Antiplasmin | HP | 6.82 | 4.37 |
| L.AAPPGHQLHRAHYDLRHTFMGVVSLGSPSGEVSHPRKT.R | α-2-HS-glycoprotein | BCP | 4.27 | 6.43 |
| G.VVSLGSPSGEVSHPRKT.R | α-2-HS-glycoprotein | BCP | 4.19 | 6.00 |
| L.AAPPGHQLHRAHYDLRHTFMGVVSLGSPSGEVSHPR.K | α-2-HS-glycoprotein | BCP | 4.22 | 5.93 |
| R.AHYDLRHTFMGVVSLGSPSGEVSHPRKT.R | α-2-HS-glycoprotein | BCP | 4.33 | 7.04 |
| R.AHYDLRHTFMGVVSLGSPSGEVSHPR.K | α-2-HS-glycoprotein | BCP | 4.46 | 7.04 |
| L.AAPPGHQLHRAHYDL.R | α-2-HS-glycoprotein | BCP | 4.51 | 5.81 |
| L.PPAGSPPDSHVLLAAPPGHQLHRAHYDLRHTFMGVVSLGSPSGEVSHPRKT.R | α-2-HS-glycoprotein | BCP | 4.69 | 5.66 |
| S.PSGEVSHPRKT.R | α-2-HS-glycoprotein | BCP&HP | 6.18 | 6.59 |
| S.PSGEVSHPR.K | α-2-HS-glycoprotein | BCP&HP | 6.41 | 6.23 |
| R.HTFMGVVSLGSPSGEVSHPR.K | α-2-HS-glycoprotein | BCP(HP) | 5.11 | 6.89 |
| R.HTFMGVVSLGSPSGEVSHPRKT.R | α-2-HS-glycoprotein | BCP(HP) | 5.52 | 7.04 |
| G.SPSGEVSHPR.K | α-2-HS-glycoprotein | HP(BCP) | 5.63 | 4.96 |
| L.GSPSGEVSHPR.K | α-2-HS-glycoprotein | HP(BCP) | 6.23 | 5.87 |
| R.HTFM(−hydroxylation)GVVSLGSPSGEVSHPR.K | α-2-HS-glycoprotein | HP(BCP) | 5.85 | 5.62 |
| R.AHYDLRHTFM(−hydroxylation)GVVSLGSPSGEVSHPRKT.R | α-2-HS-glycoprotein | HP(BCP) | 7.04 | 6.86 |
| R.AHYDLRHTFM(−hydroxylation)GVVSLGSPSGEVSHPR.K | α-2-HS-glycoprotein | BCP&HP | 7.04 | 6.67 |
| R.ADSQAQLLLSTVVGVFTAPGLHLKQPFVQGLALYTPVVLPR.S | Angiotensinogen | BCP | 4.48 | 6.26 |
| R.TIHLTMPQLVLQGSYDLQDLLAQAELPAILHTELNLQKLSNDRIR.V | Angiotensinogen | BCP | 4.80 | 6.93 |
| K.TSPVDEKALQDQLVLVAAKLDTEDKLR.A | Angiotensinogen | BCP&HP | 5.97 | 5.99 |
| K.TSPVDEKALQDQLVLVAAKLDTEDKL.R | Angiotensinogen | BCP(HP) | 5.36 | 5.95 |
| S.PVDEKALQDQLVLVAAKLDTEDKLR.A | Angiotensinogen | HP | 5.34 | 4.32 |
| A.VYDQSATALHFLGR.V | Angiotensinogen | HP | 5.32 | 4.28 |
| R.VGEVLNSIFFELEADEREPTESTQQLNKPEVLEVTLNRPFLFAVYDQSATALHFLGR.V | Angiotensinogen | HP | 6.34 | 4.21 |
| R.VGEVLNSIFFELEADEREPTESTQQLNKPEVLEVTLNRPFLFAVYDQSATALHFLGRVANPLSTA.- | Angiotensinogen | BCP(HP) | 6.26 | 6.28 |
| R.IEDGFSLKEQLQDMGLVDLFSPEKSKLPGIVAEGR.D | Antithrombin III | BCP | 4.29 | 5.74 |
| Q.PLDFKENAEQS.R | Antithrombin III | BCP | 4.18 | 5.00 |
| R.RVWELSK.A | Antithrombin III | BCP | 4.51 | 6.40 |
| R.RVAEGTQVLELPFKGDDITMVLILPKPEKSLAKVEKELTPEVLQEWLDELEEMMLVVHMPR.F | Antithrombin III | BCP | 4.53 | 6.32 |
| I.PSEAINELTVLVLVNTIYFKGLWKSKFSPENT.R | Antithrombin III | BCP | 4.75 | 6.63 |
| R.RVWELSKANS.R | Antithrombin III | BCP&HP | 5.30 | 6.48 |
| R.RVAEGTQVLELPFKGDDITMVLILPKPEKSLAK.V | Antithrombin III | BCP&HP | 6.04 | 6.89 |
| R.DDLYVSDAFHKAFLEVNEEGSEAAASTAVVIAGR.S | Antithrombin III | BCP&HP | 6.23 | 6.59 |
| I.PSEAINELTVLVLVNTIYFKGLWKSKFSPENTR.K | Antithrombin III | BCP&HP | 6.28 | 6.34 |
| R.SLNPNRVTFK.A | Antithrombin III | BCP(HP) | 4.98 | 6.43 |
| R.ITDVIPSEAINELTVLVLVNTIYFKGLWKSKFSPENT.R | Antithrombin III | BCP(HP) | 6.15 | 6.84 |
| K.AFLEVNEEGSEAAASTAVVIAGR.S | Antithrombin III | HP | 4.97 | 4.14 |
| Q.PLDFKENAEQSR.A | Antithrombin III | HP(BCP) | 4.91 | 5.20 |
| N.ETYQDISELVYGAKLQPLDFKENAEQSR.A | Antithrombin III | HP(BCP) | 5.23 | 4.92 |
| K.SKLPGIVAEGRDDLYVSDAFHKAFLEVNEEGSEAAASTAVVIAGR.S | Antithrombin III | HP(BCP) | 5.56 | 5.70 |
| R.RVWELSKANSRFATTFYQHLADSKNDNDNIFLSPL.S | Antithrombin III | HP(BCP) | 5.97 | 6.54 |
| R.ITDVIPSEAINELTVLVLVNTIYFKGLWKSKFSPENTR.K | Antithrombin III | HP(BCP) | 7.04 | 6.68 |
| R.RVWELSKANSR.F | Antithrombin III | HP(BCP) | 5.76 | 5.32 |
| R.RVAEGTQVLELPFKGDDITMVLILPKPEKSLAKVEKELTPEVLQEWLDELEEMMLVVHMPRFRIEDGFSLKEQLQDMGLVDLFSPEKSKLPGIVAEGR.D | Antithrombin III | BCP | 4.16 | 5.95 |
| R.KELFYKADGESC(−dehydro)SASMMYQEGKFR.Y; R.SLNPNRVTFKANRPFLVFIREVPLNTIIFMGRVANPC(−dehydro)VK.- | Antithrombin III | BCP | 4.51 | 7.18 |
| R.KELFYKADGESC(−dehydro)SASMMYQEGKFR.Y; R.SLNPNRVTFKANRPFLVFIREVPLNTIIFM(−hydroxylation)GRVANPC(−dehydro)VK.- | Antithrombin III | BCP | 4.71 | 6.58 |
| R.KELFYKADGESC(−dehydro)SASMMYQEGKFR.Y; K.ANRPFLVFIREVPLNTIIFMGRVANPC(−dehydro)VK.- | Antithrombin III | BCP | 4.36 | 6.20 |
| R.FRIEDGFSLKEQLQDMGLVDLFSPEKSKLPGIVAEGR.D | Antithrombin III | BCP&HP | 5.65 | 6.08 |
| R.KELFYKAD(−reduction)GESC(−dehydro)SASMMYQEGKFR.Y; L.NRVTFKANRPFLVFIREVPLNTIIFMGRVANPC(−dehydro)VK.- | Antithrombin III | BCP(HP) | 4.25 | 3.70 |
| R.KELFYKADGESC(−dehydro)SASMMYQEGKFR.Y;  R.VANPC(−dehydro)VK.- | Antithrombin III | BCP(HP) | 5.38 | 6.53 |
| R.LAEYHAKATEHLSTLSEKAKPALEDL.R | Apolipoprotein A-I | BCP | 4.20 | 5.51 |
| R.QKLHELQEKLSPLGEEMRDRA.R | Apolipoprotein A-I | BCP(HP) | 5.24 | 5.86 |
| R.QKLHELQEKLSPLGEEMR(−hydroxylation)DRA.R | Apolipoprotein A-I | BCP | 4.14 | 5.64 |
| R.LAPLAEDVRGNL.R | Apolipoprotein A-IV | BCP | 4.15 | 6.71 |
| R.VLRENADSLQASLRPHADELKAKIDQNVEELKGRLTPYADEFKVKIDQTVEEL.R | Apolipoprotein A-IV | BCP | 4.31 | 6.65 |
| R.SLAPYAQDTQEKLNHQLEGLTFQMKKNAEELKARISASAEELRQ.R | Apolipoprotein A-IV | BCP | 4.26 | 6.56 |
| R.RVEPYGENFNKALVQQMEQL.R | Apolipoprotein A-IV | BCP | 4.24 | 6.38 |
| R.LLPHANEVSQKIGDNLRELQQRLEPYADQL.R | Apolipoprotein A-IV | BCP | 4.26 | 6.30 |
| Y.AQDTQEKLNHQLEGLTFQMKKNAEELKARISASAEELR.Q | Apolipoprotein A-IV | BCP | 4.25 | 6.28 |
| Y.AQRMERVLRENADSLQASLRPHADELKAKIDQNVEELKGRLTPYADEFKVKIDQTVEELRR.S | Apolipoprotein A-IV | BCP | 4.30 | 6.26 |
| L.APYAQDTQEKLNHQLEGLTFQMKKNAEELKARISASAEELR.Q | Apolipoprotein A-IV | BCP | 4.32 | 6.26 |
| R.RSLAPYAQDTQEKLNHQLEGLTFQMKKNAEELKARISASAEELRQR.L | Apolipoprotein A-IV | BCP | 4.24 | 6.00 |
| R.SLAPYAQDTQEKLNHQLEGLTFQMKKNAEELKARISASAEEL.R | Apolipoprotein A-IV | BCP | 4.24 | 5.89 |
| Y.AQDTQEKLNHQLEGLTFQMKKNAEELKARISASAEEL.R | Apolipoprotein A-IV | BCP | 4.22 | 5.86 |
| R.ENADSLQASLRPHADELKAKIDQNVEELKG.R | Apolipoprotein A-IV | BCP | 4.23 | 5.81 |
| R.LLPHANEVSQKIGDNL.R | Apolipoprotein A-IV | BCP | 4.29 | 5.80 |
| Y.AQRMERVLRENADSLQASLRPHADELKAKIDQNVEELKGR.L | Apolipoprotein A-IV | BCP | 4.29 | 5.79 |
| R.ARLLPHANEVSQKIGDNLRELQQ.R | Apolipoprotein A-IV | BCP | 3.82 | 5.76 |
| R.MERVLRENADSLQASLRPHADELKAKIDQNVEELKGR.L | Apolipoprotein A-IV | BCP | 4.28 | 5.72 |
| A.PYAQDTQEKLNHQLEGLTFQMKK.N | Apolipoprotein A-IV | BCP | 4.30 | 5.72 |
| R.ENADSLQASLRPHADELKAKIDQNVEELKGRLTPYADEFK.V | Apolipoprotein A-IV | BCP | 4.22 | 5.70 |
| L.APYAQDTQEKLNHQLEGLTFQMK.K | Apolipoprotein A-IV | BCP | 4.30 | 5.57 |
| A.DEFKVKIDQTVEELR.R | Apolipoprotein A-IV | BCP | 4.30 | 5.36 |
| H.ADELKAKIDQNVEELKGR.L | Apolipoprotein A-IV | BCP | 4.08 | 5.32 |
| R.ARLLPHANEVSQKIGDNL.R | Apolipoprotein A-IV | BCP | 4.22 | 5.32 |
| R.ENADSLQASLRPH.A | Apolipoprotein A-IV | BCP | 4.24 | 5.30 |
| R.ARLLPHANEVSQKIGDNLR.E | Apolipoprotein A-IV | BCP | 4.16 | 5.28 |
| V.KIDQTVEELR.R | Apolipoprotein A-IV | BCP | 4.16 | 5.28 |
| Y.AQDTQEKLNHQLEGLTFQMKKNAEELKARISASAEELRQR.L | Apolipoprotein A-IV | BCP | 3.88 | 5.18 |
| H.ANEVSQKIGDNLR.E | Apolipoprotein A-IV | BCP | 4.16 | 5.04 |
| A.PYAQDTQEKLNHQLEGLTFQMKKNAEELKA.R | Apolipoprotein A-IV | BCP | 4.46 | 6.88 |
| L.APYAQDTQEKLNHQLEGLTFQMKKNAEELKA.R | Apolipoprotein A-IV | BCP | 4.48 | 6.56 |
| Y.AQRMERVLRENADSLQASLRPHADELKAKIDQNVEELKGRLTPYADEFKVKIDQTVEELR.R | Apolipoprotein A-IV | BCP | 4.38 | 6.40 |
| Y.AQDTQEKLNHQLEGLTFQMKKNAEELKA.R | Apolipoprotein A-IV | BCP | 4.44 | 6.36 |
| H.ADELKAKIDQNVEELKGRLTPYADEFKVKIDQTVEEL.R | Apolipoprotein A-IV | BCP | 4.42 | 6.34 |
| A.QDTQEKLNHQLEGLTFQMKKNAEELKAR.I | Apolipoprotein A-IV | BCP | 4.48 | 6.18 |
| P.YAQDTQEKLNHQLEGLTFQMKKNAEELKA.R | Apolipoprotein A-IV | BCP | 4.43 | 5.96 |
| R.LTPYADEFKVKIDQTVEEL.R | Apolipoprotein A-IV | BCP | 4.34 | 5.92 |
| Q.LEGLTFQMKKNAEELKAR.I | Apolipoprotein A-IV | BCP | 4.47 | 5.74 |
| Y.AQDTQEKLNHQLEGLTFQMKK.N | Apolipoprotein A-IV | BCP | 4.33 | 5.40 |
| R.RRVEPYGENFNKALVQQMEQ.L | Apolipoprotein A-IV | BCP | 4.34 | 5.32 |
| P.YADEFKVKIDQTVEELR.R | Apolipoprotein A-IV | BCP&HP | 5.26 | 5.79 |
| Y.ADEFKVKIDQTVEELR.R | Apolipoprotein A-IV | BCP&HP | 5.20 | 5.75 |
| R.LTPYADEFKVKIDQTVEELR.R | Apolipoprotein A-IV | BCP&HP | 5.23 | 5.67 |
| K.VKIDQTVEELR.R | Apolipoprotein A-IV | BCP&HP | 5.20 | 5.60 |
| R.GNTEGLQKSLAELGGHLDQQVEEF.R | Apolipoprotein A-IV | BCP&HP | 5.45 | 6.72 |
| A.PYAQDTQEKLNHQLEGLTFQMKKNAEELKARISASAEELR.Q | Apolipoprotein A-IV | BCP&HP | 5.59 | 6.61 |
| R.VLRENADSLQASLRPHADELKAKIDQNVEELKGRLTPY.A | Apolipoprotein A-IV | BCP&HP | 5.52 | 6.59 |
| R.RSLAPYAQDTQEKLNHQLEGLTFQMKKNAEELKARISASAEELR.Q | Apolipoprotein A-IV | BCP&HP | 5.73 | 6.18 |
| K.SLAELGGHLDQQVEEFRR.R | Apolipoprotein A-IV | BCP&HP | 5.53 | 6.11 |
| R.LAKDSEKLKEEIGKELEELR.A | Apolipoprotein A-IV | BCP&HP | 5.41 | 6.08 |
| L.QKSLAELGGHLDQQVEEFR.R | Apolipoprotein A-IV | BCP&HP | 5.48 | 5.97 |
| L.TPYADEFKVKIDQTVEELR.R | Apolipoprotein A-IV | BCP&HP | 5.40 | 5.85 |
| R.VLRENADSLQASLRPHADELKAKIDQNVEELK.G | Apolipoprotein A-IV | BCP&HP | 5.46 | 5.79 |
| E.GLQKSLAELGGHLDQQVEEFR.R | Apolipoprotein A-IV | BCP&HP | 5.53 | 5.79 |
| K.GRLTPYADEFKVKIDQTVEELR.R | Apolipoprotein A-IV | BCP&HP | 5.52 | 5.70 |
| L.APYAQDTQEKLNHQLEGLTFQMKKNAEELKAR.I | Apolipoprotein A-IV | BCP&HP | 5.83 | 6.79 |
| R.VLRENADSLQASLRPHADELKAKIDQNVEELKGRLTPYADEFK.V | Apolipoprotein A-IV | BCP&HP | 5.80 | 6.67 |
| H.ADELKAKIDQNVEELKGRLTPYADEFKVKIDQTVEELRR.S | Apolipoprotein A-IV | BCP&HP | 5.79 | 6.38 |
| K.AKIDQNVEELKGRLTPYADEFKVKIDQTVEELR.R | Apolipoprotein A-IV | BCP&HP | 5.79 | 6.15 |
| K.AKIDQNVEELKGRLTPYADEFKVKIDQTVEELRR.S | Apolipoprotein A-IV | BCP&HP | 5.86 | 6.15 |
| T.PYADEFKVKIDQTVEELRR.S | Apolipoprotein A-IV | BCP&HP | 5.76 | 6.11 |
| L.AELGGHLDQQVEEFRR.R | Apolipoprotein A-IV | BCP&HP | 5.83 | 6.04 |
| L.GGHLDQQVEEFRR.R | Apolipoprotein A-IV | BCP&HP | 5.80 | 6.00 |
| Q.KSLAELGGHLDQQVEEFR.R | Apolipoprotein A-IV | BCP&HP | 5.77 | 5.89 |
| N.TEGLQKSLAELGGHLDQQVEEFR.R | Apolipoprotein A-IV | BCP&HP | 5.88 | 5.77 |
| R.RVEPYGENFNKALVQQMEQLR.Q | Apolipoprotein A-IV | BCP&HP | 6.18 | 7.04 |
| R.RRVEPYGENFNKALVQQMEQLR.Q | Apolipoprotein A-IV | BCP&HP | 6.00 | 6.72 |
| H.ADELKAKIDQNVEELKGRLTPYADEFKVKIDQTVEELR.R | Apolipoprotein A-IV | BCP&HP | 6.04 | 6.71 |
| T.PYADEFKVKIDQTVEELR.R | Apolipoprotein A-IV | BCP&HP | 6.11 | 6.46 |
| R.QKLGPHAGDVEGHLSFLEKDLR.D | Apolipoprotein A-IV | BCP&HP | 6.11 | 6.34 |
| L.GGHLDQQVEEFR.R | Apolipoprotein A-IV | BCP&HP | 6.11 | 6.20 |
| G.HLDQQVEEFR.R | Apolipoprotein A-IV | BCP&HP | 5.97 | 6.15 |
| A.ELGGHLDQQVEEFR.R | Apolipoprotein A-IV | BCP&HP | 6.04 | 6.08 |
| R.GNTEGLQKSLAELGGHLDQQVEEFRRRVEPYGENFNKALVQQMEQLR.Q | Apolipoprotein A-IV | BCP&HP | 6.00 | 5.85 |
| A.PYAQDTQEKLNHQLEGLTFQMKKNAEELKAR.I | Apolipoprotein A-IV | BCP&HP | 6.20 | 7.11 |
| K.SLAELGGHLDQQVEEF.R | Apolipoprotein A-IV | BCP&HP | 6.28 | 7.08 |
| R.SLAPYAQDTQEKLNHQLEGLTFQMKKNAEELKARISASAEELRQR.L | Apolipoprotein A-IV | BCP&HP | 6.20 | 6.95 |
| E.LGGHLDQQVEEFR.R | Apolipoprotein A-IV | BCP&HP | 6.20 | 6.60 |
| R.ENADSLQASLRPHADELKAKIDQNVEELKGR.L | Apolipoprotein A-IV | BCP&HP | 6.23 | 6.60 |
| R.VLRENADSLQASLRPHADELKAKIDQNVEELKGRLTPYADEFKVKIDQTVEELR.R | Apolipoprotein A-IV | BCP&HP | 6.66 | 7.30 |
| R.VLRENADSLQASLRPHADELKAKIDQNVEELKGRLTPYADEFKVKIDQTVEELRR.S | Apolipoprotein A-IV | BCP&HP | 6.64 | 7.11 |
| R.VLRENADSLQASLRPHADELKAKIDQNVEELKGR.L | Apolipoprotein A-IV | BCP&HP | 6.38 | 7.08 |
| R.ENADSLQASLRPHADELKAKIDQNVEELKGRLTPYADEFKVKIDQTVEELRR.S | Apolipoprotein A-IV | BCP&HP | 6.63 | 6.95 |
| L.AELGGHLDQQVEEFR.R | Apolipoprotein A-IV | BCP&HP | 6.41 | 6.43 |
| R.TQVNTQAEQLR.R | Apolipoprotein A-IV | BCP&HP | 6.49 | 5.82 |
| R.SLAPYAQDTQEKLNHQLEGLTFQMKKNAEELKAR.I | Apolipoprotein A-IV | BCP&HP | 6.93 | 7.70 |
| R.SLAPYAQDTQEKLNHQLEGLTFQMKKNAEELKARISASAEELR.Q | Apolipoprotein A-IV | BCP&HP | 6.72 | 7.46 |
| R.LAPLAEDVRGNLR.G | Apolipoprotein A-IV | BCP&HP | 6.92 | 7.32 |
| R.ENADSLQASLRPHADELKAKIDQNVEELKGRLTPYADEFKVKIDQTVEELR.R | Apolipoprotein A-IV | BCP&HP | 6.85 | 7.28 |
| R.SLAPYAQDTQEKLNHQLEGLTFQMKKNAEELKARISASAEELRQRLAPLAEDVRGNLR.G | Apolipoprotein A-IV | BCP&HP | 6.72 | 7.04 |
| R.GNTEGLQKSLAELGGHLDQQVEEFRR.R | Apolipoprotein A-IV | BCP&HP | 6.72 | 6.84 |
| R.GNTEGLQKSLAELGGHLDQQVEEFR.R | Apolipoprotein A-IV | BCP&HP | 7.58 | 7.65 |
| K.SLAELGGHLDQQVEEFR.R | Apolipoprotein A-IV | BCP&HP | 7.28 | 7.53 |
| Q.DTQEKLNHQLEGLTFQMKKNAEELKAR.I | Apolipoprotein A-IV | BCP&HP | 4.93 | 6.00 |
| R.SLAPYAQDTQEKLNHQLEGLTFQMKKNAEELK.A | Apolipoprotein A-IV | BCP&HP | 4.72 | 5.84 |
| R.SLAPYAQDTQEKLNHQLEGLTFQMKK.N | Apolipoprotein A-IV | BCP&HP | 4.81 | 6.80 |
| R.SLAPYAQDTQEKLNHQLEGLTFQMK.K | Apolipoprotein A-IV | BCP&HP | 4.92 | 6.63 |
| A.PLAEDVRGNL.R | Apolipoprotein A-IV | BCP&HP | 5.28 | 6.45 |
| R.ARLLPHANEVSQKIGDNLRELQQRLEPYADQL.R | Apolipoprotein A-IV | BCP&HP | 4.95 | 6.40 |
| P.YAQDTQEKLNHQLEGLTFQMKKNAEELKAR.I | Apolipoprotein A-IV | BCP&HP | 5.04 | 6.18 |
| H.ANEVSQKIGDNLRELQQRLEPYADQLR.T | Apolipoprotein A-IV | BCP&HP | 5.28 | 5.69 |
| F.ATELHERLAKDSEKLKEEIGKELEELR.A | Apolipoprotein A-IV | BCP&HP | 4.81 | 5.62 |
| L.TPYADEFKVKIDQTVEELRR.S | Apolipoprotein A-IV | BCP&HP | 4.87 | 5.46 |
| R.VLRENADSLQASLRPHADELKAKIDQNVEELKG.R | Apolipoprotein A-IV | BCP&HP | 5.51 | 6.82 |
| R.ENADSLQASLRPHADELKAKIDQNVEELKGRLTPY.A | Apolipoprotein A-IV | BCP&HP | 5.65 | 6.32 |
| R.ISASAEELRQR.L | Apolipoprotein A-IV | BCP&HP | 5.57 | 6.20 |
| Y.AQDTQEKLNHQLEGLTFQMKKNAEELKAR.I | Apolipoprotein A-IV | BCP&HP | 5.58 | 6.11 |
| R.RVEPYGENFNK.A | Apolipoprotein A-IV | BCP&HP | 5.43 | 6.04 |
| L.GGHLDQQVEEF.R | Apolipoprotein A-IV | BCP&HP | 5.69 | 5.92 |
| E.LGGHLDQQVEEFRR.R | Apolipoprotein A-IV | BCP&HP | 5.59 | 5.75 |
| K.IDQNVEELKGRLTPYADEFKVKIDQTVEELRR.S | Apolipoprotein A-IV | BCP&HP | 5.41 | 5.57 |
| R.QRLAPLAEDVRGNL.R | Apolipoprotein A-IV | BCP&HP | 5.95 | 7.26 |
| R.RSLAPYAQDTQEKLNHQLEGLTFQMKKNAEELKAR.I | Apolipoprotein A-IV | BCP&HP | 5.90 | 6.04 |
| R.ISASAEELRQRLAPLAEDVRGNL.R | Apolipoprotein A-IV | BCP&HP | 6.30 | 7.11 |
| R.TQVNTQAEQLRR.Q | Apolipoprotein A-IV | BCP&HP | 6.20 | 7.04 |
| R.LLPHANEVSQKIGDNLRELQQRLEPYADQLR.T | Apolipoprotein A-IV | BCP&HP | 6.36 | 6.61 |
| R.RSLAPYAQDTQEKLNHQLEGLTFQMKKNAEELKA.R | Apolipoprotein A-IV | BCP&HP | 6.91 | 7.74 |
| H.ANEVSQKIGDNLRELQQRLEPYADQL.R | Apolipoprotein A-IV | BCP&HP | 4.51 | 6.23 |
| R.RRVEPYGENFNK.A | Apolipoprotein A-IV | BCP&HP | 4.87 | 6.68 |
| A.PYAQDTQEKLNHQLEGLTFQMKKNAEELKARISASAEELRQR.L | Apolipoprotein A-IV | BCP&HP | 5.36 | 6.34 |
| A.PYAQDTQEKLNHQLEGLTFQMK.K | Apolipoprotein A-IV | BCP&HP | 4.78 | 5.98 |
| K.SLAELGGHLDQQVEEFRRRVEPYGENFNKALVQQMEQL.R | Apolipoprotein A-IV | BCP&HP | 4.94 | 5.94 |
| L.APYAQDTQEKLNHQLEGLTFQMKK.N | Apolipoprotein A-IV | BCP&HP | 5.13 | 5.85 |
| R.ENADSLQASLRPHADELKAKIDQNVEELKGRLTPYADEFKVKIDQTVEEL.R | Apolipoprotein A-IV | BCP&HP | 5.55 | 6.91 |
| L.APYAQDTQEKLNHQLEGLTFQMKKNAEELKARISASAEELRQR.L | Apolipoprotein A-IV | BCP&HP | 5.45 | 6.00 |
| R.MERVLRENADSLQASLRPHADELKAKIDQNVEELKGRLTPYADEFKVKIDQTVEELR.R | Apolipoprotein A-IV | BCP&HP | 5.41 | 5.95 |
| R.SLAPYAQDTQEKLNHQLEGLTFQMKKNAEELKARISASAEELRQRLAPLAEDVRGNL.R | Apolipoprotein A-IV | BCP&HP | 5.93 | 7.15 |
| R.GNTEGLQK.S | Apolipoprotein A-IV | BCP&HP | 6.32 | 6.88 |
| R.MERVLRENADSLQASLRPHADELKAKIDQNVEELKGRLTPYADEFKVKIDQTVEELRR.S | Apolipoprotein A-IV | BCP&HP | 6.26 | 6.11 |
| R.ELQQRLEPYADQLR.T | Apolipoprotein A-IV | HP(BCP) | 5.88 | 6.46 |
| T.EGLQKSLAELGGHLDQQVEEFR.R | Apolipoprotein A-IV | HP(BCP) | 5.45 | 5.65 |
| R.ARLLPHANEVSQKIGDNLRELQQRLEPYADQLR.T | Apolipoprotein A-IV | HP(BCP) | 5.18 | 5.46 |
| R.LAPLAEDVR.G | Apolipoprotein A-IV | HP(BCP) | 7.52 | 5.18 |
| R.GNT(−loss of ammonia)EGLQKSLAELGGHLDQQVEEFRR.R | Apolipoprotein A-IV | HP(BCP) | 5.08 | 4.84 |
| R.LTPYADEFKVKIDQTVEELRR.S | Apolipoprotein A-IV | HP(BCP) | 6.04 | 6.20 |
| R.RVEPYGENFNKALVQQM(−dethiomethyl)EQLR.Q | Apolipoprotein A-IV | HP(BCP) | 6.00 | 5.26 |
| R.SLAPYAQDTQEKLNHQLEGLTFQM(−hydroxylation)KKNAEELKARISASAEELR.Q | Apolipoprotein A-IV | HP(BCP) | 6.28 | 5.71 |
| A.PYAQDTQEKLNHQLEGLTFQM(−hydroxylation)KKNAEELKAR.I | Apolipoprotein A-IV | HP(BCP) | 6.46 | 6.04 |
| R.RVEPYGENFNKALVQQM(−hydroxylation)EQLR.Q | Apolipoprotein A-IV | HP(BCP) | 7.23 | 6.71 |
| R.RRVEPYGENFNKALVQQM(-hydroxylation)EQL.R | Apolipoprotein A-IV | HP(BCP) | 7.24 | 6.57 |
| R.MERVLRENADSLQASLRPHADELKAKIDQNVEELKGRLTPYADEFKVKIDQTVEEL.R | Apolipoprotein A-IV | BCP | 4.14 | 5.91 |
| A.EVSADQVATVMWDYFSQLSNNAKEAVEHLQKSELTQQLNALFQDKLGEVNTYAGDLQKKLVPFATELHER.I | Apolipoprotein A-IV | BCP | 4.18 | 5.85 |
| R.QKLGPHAGDVEGHLSFLEKDL.R | Apolipoprotein A-IV | BCP | 4.28 | 5.58 |
| R.RVEPYGENFNKALVQ(−dehydration).Q | Apolipoprotein A-IV | BCP | 4.35 | 5.98 |
| R.RVEPYGENFNKALVQ(−dehydration)QME.Q | Apolipoprotein A-IV | BCP | 4.33 | 5.83 |
| S.LAELGGHLDQQVEEFR.R | Apolipoprotein A-IV | BCP&HP | 4.29 | 5.59 |
| R.RVEPYGENFNKAL(−dehydration).V | Apolipoprotein A-IV | BCP&HP | 5.73 | 6.15 |
| R.SLAPYAQDTQEKLNHQLEGLTFQMKKNAEELKA.R | Apolipoprotein A-IV | BCP&HP | 6.15 | 7.62 |
| R.RRVEPYGENFNKALVQQMEQL.R | Apolipoprotein A-IV | BCP&HP | 6.23 | 7.20 |
| R.SLAPYAQDTQEKLNHQLEGLTFQM(−hydroxylation)KKNAEELKAR.I | Apolipoprotein A-IV | BCP&HP | 7.23 | 6.74 |
| R.RRVEPYGENFNKALVQQMEQ(-loss of ammonia)L.R | Apolipoprotein A-IV | BCP(HP) | 4.37 | 3.70 |
| R.RVEPYGENFNKALV(−dehydration).Q | Apolipoprotein A-IV | BCP(HP) | 4.84 | 6.04 |
| A.PYAQDT(−loss of ammonia)QEKLNHQLEGLTFQMKKNAEELKAR.I | Apolipoprotein A-IV | BCP(HP) | 5.00 | 5.83 |
| R.E(−pyro-glu)NADSLQASLRPHADELKAKIDQNVEELKGR.l | Apolipoprotein A-IV | BCP(HP) | 5.11 | 5.76 |
| R.GNTEGLQKSLAELGGHLDQQ(−dehydration).V | Apolipoprotein A-IV | BCP(HP) | 5.48 | 6.65 |
| R.GNTEGLQKSLAELGGHLDQQ(−dehydration)V.E | Apolipoprotein A-IV | BCP(HP) | 5.41 | 6.54 |
| Q.VNTQAEQLRRQLTPY.A | Apolipoprotein A-IV | BCP(HP) | 5.45 | 5.69 |
| R.SLAPYAQDTQEKLNHQLEGLTFQM(−hydroxylation)KKNAEELKA.R | Apolipoprotein A-IV | BCP(HP) | 6.56 | 7.00 |
| R.SLAPYAQDTQEKLNHQLEGLTFQM(−hydroxylation)KK.N | Apolipoprotein A-IV | BCP(HP) | 4.31 | 3.70 |
| R.SLAPYAQDTQEKLNHQLEGLTFQM(−hydroxylation)KKNAEELKARISASAEELRQRLAPLAEDVRGNLR.G | Apolipoprotein A-IV | HP | 6.15 | 4.31 |
| R.ISASAEELR.Q | Apolipoprotein A-IV | HP | 7.49 | 3.85 |
| A.SEAEDASLLSFMQGYMKHATKTAKDALSSV(→M)QESQVAQQAR.G | Apolipoprotein C-III | HP(BCP) | 6.38 | 5.76 |
| N.LHQSNTSRAELLVTEAPSKPITVTV.E | Basement membrane-specific heparan sulfate proteoglycan core protein | BCP(HP) | 4.96 | 5.85 |
| A.IQRTPKIQVYSRHPAENGKSNFLNC(−dehydro)YVSGFHPSDIEVDLLKNGERIEKVEHSDLSFSKDWSFYLLYYTEFTPTEKDEYAC(−dehydro)RVNHVTLSQPKIVKWDRDI(→M−oxidation).- | β-Microglobulin | BCP&HP | 8.01 | 8.03 |
| A.IQRTPKIQVYSRHPAENGKSNFLNC(−dehydro)YVSGFHPSDIEVDLLKNGERIEKVEHSDLSFSKDWSFYLLYYTEFTPTEKDEYAC(−dehydro)RVNHVTLSQPKIVKWDRDI(→M).- | β-Microglobulin | BCP(HP) | 5.35 | 7.56 |
| R.VFDKDGNGYISAAELR.H | Calmodulin | HP | 5.34 | 4.21 |
| R.SLLSNVEGDNAVPMQHNNRPTQPLKGR.T | Carbonic anhydrase 1 | BCP | 4.18 | 5.34 |
| R.SLLSNVEGDNAVPMQHNNRPTQPLKG.R | Carbonic anhydrase 1 | BCP(HP) | 4.94 | 6.00 |
| R.SLLSNVEGDNAVPM(-hydroxylation)QHNNRPTQPLKG.R | Carbonic anhydrase 1 | BCP(HP) | 4.93 | 5.62 |
| R.SKSKDHEELSLVASEAVR.A | Carboxypeptidase B2 | BCP&HP | 5.36 | 5.93 |
| K.AVASFLRRNINQIKAYISMHSYSQHIVFPYSYTR.S | Carboxypeptidase B2 | BCP&HP | 5.32 | 5.57 |
| A.FQSGQVLAALPR.T | Carboxypeptidase B2 | BCP&HP | 5.82 | 5.91 |
| K.AVASFLRRNINQIKAYISMHSYSQHIVFPYSYTRSKSKDHEELSLVASEAVR.A | Carboxypeptidase B2 | BCP(HP) | 4.71 | 5.52 |
| R.SKSKDHEELSLVASEAV.R | Carboxypeptidase B2 | BCP(HP) | 4.54 | 5.80 |
| K.SKDHEELSLVASEAVR.A | Carboxypeptidase B2 | HP(BCP) | 5.20 | 5.23 |
| A.KEKHYYIGIIETTWDYASDHGEKKLISVDTEHSNIYLQNGPDRIGRLY.K | Ceruloplasmin | BCP | 4.25 | 5.85 |
| W.DYASDHGEKKLISVDTEHSNIYLQNGPDRIGR.L | Ceruloplasmin | BCP | 4.21 | 5.18 |
| R.QKDVDKEFYLFPTVFDENESLLLEDNI.R | Ceruloplasmin | BCP | 4.39 | 6.97 |
| K.KLVYREYTDASFTNRKERGPEEEHLGILGPVIWAEVGDTIRVTFHNKGAYPLSIEPIGVR.F | Ceruloplasmin | BCP | 4.46 | 6.26 |
| R.IGGSYKKLVYREYTDASFTNRKERGPEEEHLGILGPVIWAEVGDTIR.V | Ceruloplasmin | BCP | 4.36 | 5.74 |
| K.KLVYREYTDASFTN.R | Ceruloplasmin | BCP | 4.53 | 5.61 |
| A.KEKHYYIGIIETTWDYASDHGEKKLISVDTEHSNIYLQNGPDRIGRLYKKALYLQYTDET.F | Ceruloplasmin | BCP | 4.34 | 5.48 |
| R.IGGSYKKLVYREYTDASFTNR.K | Ceruloplasmin | BCP&HP | 5.41 | 5.87 |
| K.KALYLQYTDETFR.T | Ceruloplasmin | BCP&HP | 5.63 | 5.70 |
| L.SIEPIGVRFNKNNEGTYYSPNYNPQSR.S | Ceruloplasmin | BCP&HP | 5.45 | 5.66 |
| A.KEKHYYIGIIETTWDYASDHGEKK.L | Ceruloplasmin | BCP&HP | 5.98 | 5.99 |
| A.KEKHYYIGIIETTWDYASDHGEKKLISVDTEHSNIYLQNGPDRIGRLYK.K | Ceruloplasmin | BCP&HP | 6.28 | 6.80 |
| A.KEKHYYIGIIETTWDYASDHGEKKLISVDTEHSNIYLQNGPDRIGRLYKKALYLQYTDETFR.T | Ceruloplasmin | BCP&HP | 6.28 | 6.60 |
| R.IGGSYKKLVYREYTDASFTNRKERGPEEEHLGILGPVIWAEVGDTIRVTFHNKGAYPLSIEPIGVR.F | Ceruloplasmin | BCP&HP | 6.40 | 7.08 |
| A.KEKHYYIGIIETTWDYASDHGEKKLISVDTEHSNIYLQNGPDRIGR.L | Ceruloplasmin | BCP&HP | 6.38 | 6.98 |
| R.QKDVDKEFYLFPTVFDENESLLLEDNIR.M | Ceruloplasmin | BCP&HP | 6.86 | 7.32 |
| W.DYASDHGEKKLISVDTEHSNIYLQNGPDRIGRLYK.K | Ceruloplasmin | BCP(HP) | 4.72 | 5.32 |
| A.KEKHYYIGIIETTWDYASDHGEKKLISVDTEHSNIYLQNGPDR.I | Ceruloplasmin | BCP(HP) | 5.26 | 6.04 |
| R.FNKNNEGTYYSPNYNPQ.S | Ceruloplasmin | BCP(HP) | 5.11 | 5.88 |
| A.KEKHYYIGIIETTWDYASDHGEKKLISVDTEHSNIYLQNGPDRIGRLYKK.A | Ceruloplasmin | BCP(HP) | 5.34 | 5.81 |
| A.KEKHYYIGIIETTWDYASDHGEKKLISVDTEHSNIYLQNGPDRIG.R | Ceruloplasmin | BCP(HP) | 5.48 | 6.36 |
| A.KEKHYYIGIIETTWDYASDHGEKKLISVDTEHSNIYLQNGPDRIGRLYKKALYLQYTDETF.R | Ceruloplasmin | BCP(HP) | 5.40 | 6.18 |
| R.LYKKALYLQYTDETF.R | Ceruloplasmin | BCP(HP) | 4.40 | 5.59 |
| K.VFNPRRKLEFALLFLVFDENESWYLDDNIKTYSDHPEKVNKDDEEFIESNKMHAINGR.M | Ceruloplasmin | BCP(HP) | 5.51 | 6.20 |
| R.FNKNNEGTYYSPNYNPQSR.S | Ceruloplasmin | HP(BCP) | 5.26 | 5.79 |
| F.PTVFDENESLLLEDNIR.M | Ceruloplasmin | HP(BCP) | 5.15 | 5.66 |
| A.KEKHYYIGIIETTWDYASDHGEK.K | Ceruloplasmin | HP(BCP) | 5.36 | 5.20 |
| R.Q(-pyro-glu)KDVDKEFYLFPTVFDENESLLLEDNIR.M | Ceruloplasmin | HP(BCP) | 5.64 | 5.76 |
| R.SVPPSASHVAPTETFTYEWTVPKEVGPTNADPVC(−dehydro)LAKMYYSAVDPTKDIFTGLIGPM(−hydroxylation)KIC(−dehydro)KKGSLHANGRQKDVDKEFYLFPTVFDENESLLLEDNIR.M | Ceruloplasmin | HP(BCP) | 6.23 | 6.32 |
| R.GVYSSDVFDIFPGTYQTLEMFPRTPGIWLLHCHVTDHIHAGMETTYTVLQNEDTKSG.- | Ceruloplasmin | BCP | 4.08 | 5.83 |
| K.KLVYREYTDASFTN(→D)RKERGPEEEHLGILGPVIWAEVGDTIRVTFHNKGAYPLSIEPIGVR.F | Ceruloplasmin | BCP | 4.09 | 5.64 |
| A.KEKHYYIGIIETTWDYASDHGEKKLISVDTEHSNIYLQNGPDRIGRLYKKALYLQYTDETFRTTIEKPVWLGFLGPIIKAETGDKVYVHLKNLASRPY.T | Ceruloplasmin | BCP | 4.32 | 5.62 |
| R.IGGSYKKLVYREYTDASFTNRKERGPEEEHLGILGPVIWAEVGDTIRVTFHNKGAYPLSIEPIGVRFNKNNEGTYYSPNYNPQS.R | Ceruloplasmin | BCP | 4.60 | 7.20 |
| R.IGGSYKKLVYREYTDASFTNRKERGPEEEHLGILGPVIWAEVGDTIRVTFHNKGAYPLSIEPIGVRFNKNNEGTYYSPNYNPQSR.S | Ceruloplasmin | BCP&HP | 6.64 | 6.75 |
| V.PPSASHVAPTETFTYEWTVPKEVGPTNADPVC(−dehydro)LAKMYYSAVDPTKDIFTGLIGPMKIC(−dehydro)KKGSLHANGR.Q | Ceruloplasmin | BCP(HP) | 5.62 | 6.58 |
| V.P(−hydroxylation)PSASHVAPTETFTYEWTVPKEVGPTNADPVC(−dehydro)LAKMYYSAVDPTKDIFTGLIGPMKIC(−dehydro)KKGSLHANGR.Q | Ceruloplasmin | BCP(HP) | 5.68 | 6.15 |
| R.SVPPSASHVAPTETFTYEWTVPKEVGPTNADPVC(−dehydro)LAKMYYSAVDPTKDIFTGLIGPMKIC(−dehydro)KKGSLHANGRQKDVDKEFYLFPTVFDENESLLLEDNIR.M | Ceruloplasmin | BCP(HP) | 6.66 | 7.36 |
| K.AKSHAPEVITSSPL.K | Coagulation factor X | BCP(HP) | 5.04 | 5.53 |
| R.TINVPLRRDQTIRFDHVITNMNNNYEPR.S | Complement C1q, B chain | BCP(HP) | 5.11 | 5.82 |
| T.SFSHMLGATNPTQKTKESLG.R | Complement C2 | BCP(HP) | 5.62 | 5.97 |
| R.EGVQKEDIPPADLSDQVPDTESET.R | Complement C3 | BCP | 3.96 | 7.60 |
| G.SPMYSIITPNILRLESEETMVLEAHDAQGDVPVTVTVHDFPGKKLVLSSEK.T | Complement C3 | BCP | 4.24 | 6.04 |
| V.PVAVQGEDTVQSLTQGDGVAKLSINTHPSQKPLSITVR.T | Complement C3 | BCP | 4.32 | 6.00 |
| R.VPVAVQGEDTVQSLTQGDGVAKLSINTHPSQKPLSITVRTKKQELSEAEQAT.R | Complement C3 | BCP | 4.23 | 5.96 |
| L.TQGDGVAKLSINTHPSQKPLSITV.R | Complement C3 | BCP | 4.31 | 5.82 |
| N.PDGSPAYRVPVAVQGEDTVQSLTQGDGVAKLSINTHPSQKPLSITV.R | Complement C3 | BCP | 4.27 | 5.77 |
| V.AKLSINTHPSQKPLSITVR.T | Complement C3 | BCP | 4.32 | 5.73 |
| R.TKKQELSEAEQAT.R | Complement C3 | BCP | 3.72 | 5.72 |
| K.SGQSEDRQPVPGQQMTLKIEGDHGARVVLVAVDKGVFVLNKKNKLTQSK.I | Complement C3 | BCP | 4.14 | 5.70 |
| K.TGLQEVEVKAAVYHHFISDGVRKSLK.V | Complement C3 | BCP | 4.14 | 5.56 |
| R.TMQALPYSTVGNSNNYLHLSVL.R | Complement C3 | BCP | 4.28 | 5.40 |
| R.QGALELIKKGYTQQLAFRQPSSAFAAFVKR.A | Complement C3 | BCP | 4.12 | 5.20 |
| K.AAVYHHFISDGVRKSLKVVPEGIR.M | Complement C3 | BCP | 4.17 | 5.20 |
| K.AAVYHHFISDGVR.K | Complement C3 | BCP | 4.29 | 5.20 |
| T.PVAQMTEDAVDAERLK.H | Complement C3 | BCP | 4.41 | 6.11 |
| N.ILRLESEETMVLEAHDAQGDVPVTVTVHDFPGKKLVLSSEKTVLTPA.T | Complement C3 | BCP | 4.44 | 6.00 |
| V.PVAVQGEDTVQSLTQGDGVAKLSINTHPSQKPLSITV.R | Complement C3 | BCP | 4.41 | 5.99 |
| R.ILLQGTPVAQMTEDAVDAERLK.H | Complement C3 | BCP | 4.33 | 5.97 |
| G.SPMYSIITPNILRLESEETMVLEAHDAQGDVPVTVTVHDFPGKK.L | Complement C3 | BCP | 4.51 | 5.96 |
| R.VPVAVQGEDTVQSLTQGDGVAK.L | Complement C3 | BCP | 4.56 | 5.90 |
| V.SATVILHSGSDMVQAER.S | Complement C3 | BCP | 4.63 | 5.90 |
| Q.GDGVAKLSINTHPSQKPLSITV.R | Complement C3 | BCP | 4.33 | 5.86 |
| Q.GTPVAQMTEDAVDAERLK.H | Complement C3 | BCP | 4.44 | 5.85 |
| L.QGTPVAQMTEDAVDAERLK.H | Complement C3 | BCP | 4.45 | 5.83 |
| L.LQGTPVAQMTEDAVDAERLK.H | Complement C3 | BCP | 4.39 | 5.81 |
| G.SPMYSIITPNILRLESEETMVLEAHDAQGDVPVTVTVHDFPGK.K | Complement C3 | BCP | 4.42 | 5.80 |
| K.RQGALELIKKGYTQQLA.F | Complement C3 | BCP | 4.50 | 5.76 |
| T.PVAQMTEDAVDAERLKHLIVTPSG.C | Complement C3 | BCP | 4.42 | 5.65 |
| R.ILLQGTPVAQMTEDAVDAERL.K | Complement C3 | BCP | 4.37 | 5.32 |
| R.TVMVNIENPEGIPVKQDSLSSQNQLGVLPLSWDIPELVNMGQWKIR.A | Complement C3 | BCP | 4.36 | 7.04 |
| R.TELRPGETLNVNFLLRMDRAHEAKIRYYTYLIMNKGRLLKAGR.Q | Complement C3 | BCP | 4.48 | 6.76 |
| T.PGSTVLYRIFTVNHKLLPVGR.T | Complement C3 | BCP | 4.83 | 6.79 |
| R.KVLLDGVQNPR.A | Complement C3 | BCP&HP | 5.68 | 7.08 |
| R.KVLLDGVQNPRAEDLVGKSLYVSATVILHSGSDMVQAER.S | Complement C3 | BCP&HP | 5.51 | 6.64 |
| I.GMTPTVIAVHYLDETEQWEKFGLEKRQGALELIKKGYTQQLAFR.Q | Complement C3 | BCP&HP | 5.68 | 6.23 |
| T.PTVIAVHYLDETEQWEKFGLEKRQGALELIKKGYTQQLAFR.Q | Complement C3 | BCP&HP | 5.67 | 5.91 |
| N.PDGSPAYRVPVAVQGEDTVQSLTQGDGVAKLSINTHPSQKPLSITVR.T | Complement C3 | BCP&HP | 5.84 | 6.67 |
| R.IPIEDGSGEVVLSRKVLLDGVQNPR.A | Complement C3 | BCP&HP | 5.89 | 6.53 |
| R.VPVAVQGEDTVQSLTQGDGVAKLSINTHPSQKPLSITVRTKKQELSEAEQATR.T | Complement C3 | BCP&HP | 5.79 | 6.28 |
| R.VPVAVQGEDTVQSLTQGDGVAKLSINTHPSQKPLSITV.R | Complement C3 | BCP&HP | 6.11 | 7.67 |
| R.EGVQKEDIPPADLSDQVPDTESETR.I | Complement C3 | BCP&HP | 6.23 | 8.04 |
| R.TLDPERLGREGVQKEDIPPADLSDQVPDTESETR.I | Complement C3 | BCP&HP | 6.48 | 7.15 |
| R.IPIEDGSGEVVLSR.K | Complement C3 | BCP&HP | 6.38 | 6.94 |
| R.VPVAVQGEDTVQSLTQGDGVAKLSINTHPSQKPLSITVR.T | Complement C3 | BCP&HP | 6.92 | 7.72 |
| I.PPADLSDQVPDTESETR.I | Complement C3 | BCP&HP | 6.77 | 6.73 |
| M.TEDAVDAERLKHLIVTPSG.C | Complement C3 | BCP(HP) | 4.61 | 5.26 |
| R.IPIEDGSGEVVLSRKVLLDGVQNPRAEDLVGKSLYVSATVILHSGSDMVQAER.S | Complement C3 | BCP(HP) | 4.97 | 5.51 |
| K.TGLQEVEVKAAVYHHFISDGVRK.S | Complement C3 | BCP(HP) | 4.73 | 5.91 |
| R.AEDLVGKSLYVSATVILHSGSDMVQAER.S | Complement C3 | BCP(HP) | 5.04 | 7.08 |
| R.IPIEDGSGEVVLS.R | Complement C3 | BCP(HP) | 5.30 | 6.81 |
| R.TKKQELSEAEQATRTMQALPYSTVGNSNNYLHLSVLR.T | Complement C3 | BCP(HP) | 4.91 | 6.59 |
| R.VPVAVQGEDTVQSLTQGDGVAKLSINTHPSQKPL.S | Complement C3 | BCP(HP) | 5.20 | 6.52 |
| R.QGALELIKKGYTQQLAF.R | Complement C3 | BCP(HP) | 5.30 | 6.46 |
| L.PYSTVGNSNNYLHLSVLRTELRPGETLNVNFLLRMDRAHEAKIRYYTYLIMNKGRLLK.A | Complement C3 | BCP(HP) | 5.04 | 6.32 |
| L.PYSTVGNSNNYLHLSVLR.T | Complement C3 | BCP(HP) | 5.18 | 6.20 |
| G.SPMYSIITPNILR.L | Complement C3 | BCP(HP) | 5.26 | 6.04 |
| R.QGALELIKKGYTQQLAFR.Q | Complement C3 | BCP(HP) | 5.11 | 5.98 |
| T.QGDGVAKLSINTHPSQKPLSITVR.T | Complement C3 | BCP(HP) | 4.94 | 5.86 |
| I.PIEDGSGEVVLSRKVLLDGVQNPR.A | Complement C3 | BCP(HP) | 4.98 | 5.82 |
| Q.GDGVAKLSINTHPSQKPLSITVR.T | Complement C3 | BCP(HP) | 4.90 | 5.81 |
| K.LSINTHPSQKPLSITVR.T | Complement C3 | BCP(HP) | 4.75 | 5.76 |
| R.TLDPERLGREGVQKEDIPPADLSDQVPDTESET.R | Complement C3 | BCP(HP) | 5.49 | 7.30 |
| I.MNKGRLLKAGR.Q | Complement C3 | BCP(HP) | 5.67 | 6.63 |
| K.VLLDGVQNPR.A | Complement C3 | BCP(HP) | 5.52 | 6.62 |
| R.SGIPIVTSPYQIHFTKTPKYFKPGMPFDLMVFVTNPDGSPAYR.V | Complement C3 | BCP(HP) | 5.58 | 6.62 |
| V.QSLTQGDGVAKLSINTHPSQKPLSITVR.T | Complement C3 | BCP(HP) | 5.51 | 6.56 |
| Q.SLTQGDGVAKLSINTHPSQKPLSITVR.T | Complement C3 | BCP(HP) | 5.49 | 6.45 |
| L.TQGDGVAKLSINTHPSQKPLSITVR.T | Complement C3 | BCP(HP) | 5.52 | 6.40 |
| K.RIPIEDGSGEVVLSR.K | Complement C3 | BCP(HP) | 5.67 | 6.28 |
| R.TMQALPYSTVGNSNNYLHLSVLRTELRPGETLNVNFLLR.M | Complement C3 | BCP(HP) | 6.04 | 6.71 |
| R.EFKSEKGRNKFVTVQATFGTQVVEKVVLVSLQSGYLFIQTDKTIYTPGSTVLYRIFTVNHKLLPVGR.T | Complement C3 | BCP(HP) | 6.11 | 6.53 |
| R.ILLQGTPVAQMTEDAVDAERLKHLIVTPSG.C | Complement C3 | BCP(HP) | 6.40 | 6.64 |
| K.AAVYHHFISDGVRK.S | Complement C3 | BCP(HP) | 4.51 | 6.15 |
| K.LSINTHPSQKPLSITV.R | Complement C3 | BCP(HP) | 4.56 | 5.86 |
| R.QGALELIKKGYTQQLA.F | Complement C3 | BCP(HP) | 5.00 | 6.78 |
| K.IWDVVEKADIGC(-dehydro)TPGSGKDYAGVFSDAGLTFTSSSGQQTAQRAELQC(-dehydro)PQPAA.R | Complement C3 | BCP(HP) | 5.30 | 6.25 |
| Y.SIITPNILRLESEETMVLEAHDAQGDVPVTVTVHDFPGK.K | Complement C3 | BCP(HP) | 4.96 | 5.36 |
| G.TPVAQMTEDAVDAERLK.H | Complement C3 | BCP(HP) | 5.49 | 5.20 |
| R.TLDPERLGR.E | Complement C3 | BCP(HP) | 6.56 | 7.08 |
| R.TKKQELSEAEQATR.T | Complement C3 | HP | 6.92 | 4.18 |
| L.PYSTVGNSNNYLHLSVLRTELRPGETLNVNFLLRMDRAHEAKIRYYTYLIMNKGRLLKAGR.Q | Complement C3 | HP(BCP) | 6.45 | 7.57 |
| V.PDTESETR.I | Complement C3 | HP(BCP) | 6.84 | 6.78 |
| I.PIEDGSGEVVLSR.K | Complement C3 | HP(BCP) | 6.08 | 4.93 |
| R.TKKQELSEAEQATRTMQALPYSTVGNSNNYLHLSVLRTELRPGETLNVNFLLRMDRAHEAKIRYYTYLIMNKGRLLKAG.R | Complement C3 | BCP | 4.21 | 6.56 |
| L.PYSTVGNSNNYLHLSVLRTELRPGETLNVNFLLRMDRAHEAKIRYYTYLIMNKGRLLKAG.R | Complement C3 | BCP | 4.24 | 6.20 |
| R.TKKQELSEAEQATRTMQALPYSTVGNSNNYLHLSVLRTELRPGETLNVNFLLR.M | Complement C3 | BCP | 4.13 | 5.48 |
| R.ILLQGTPVAQMTEDAVDAERLKHLIVTPSG(-amidation).C | Complement C3 | BCP | 4.41 | 6.92 |
| R.AEDLVGKSLYVSATVILHSGSDMVQAERSGIPIVTSPYQIHFTKTPKYFKPGMPFDLMVFVTNPDGSPAYRVPVAVQGED(amidation)TVQSLTQGDGVAKLSINTHPSQKPLSITVR.T | Complement C3 | BCP | 4.59 | 6.68 |
| R.AEDLVGKSLYVSATVILHSGSDMVQAERSGIPIVTSPYQIHFTKTPKYFKPGMPFDLMVFVTNPDGSPAYRVPVAVQGEDTVQSLTQGDGVAKLSINTHPSQKPLSITVR.T | Complement C3 | BCP | 4.46 | 6.62 |
| R.AEDLVGKSLYVSATVILHSGSDMVQAERSGI(-dehydration).P | Complement C3 | BCP | 4.55 | 6.56 |
| R.TKKQELSEAEQAT(-dehydration)RTMQAL.P | Complement C3 | BCP | 4.64 | 6.54 |
| R.TVM(-hydroxylation)VNIENPEGIPVKQDSLSSQNQLGVLPLSWDIPELVNMGQWKIR.A | Complement C3 | BCP | 4.38 | 6.48 |
| R.KVLLDGVQNP(hydroxylation)RAEDLVGKSLYVSATVILHSGSDMVQAER.S | Complement C3 | BCP | 4.51 | 6.38 |
| R.TKKQELSEAEQATRTMQALPYSTVGNSNNYLHLSVLRTELRPGETLNVNFLLRMDRAHEAKIRYYTYLIMNKGRLLKAGR.Q | Complement C3 | BCP&HP | 6.54 | 7.53 |
| R.AEDLVGKSLYVSATVILHSGSDM(-hydroxylation)VQAER.S | Complement C3 | BCP&HP | 6.45 | 6.58 |
| M.TEDAVDAERLKHLIVTPSG(-amidation).C | Complement C3 | BCP(HP) | 4.79 | 5.52 |
| I.PPADLSDQVPDTESETR.I(unexplained modification on residues after D11 manifesting through loss of 34.9674) | Complement C3 | BCP(HP) | 6.50 | 6.92 |
| R.TMQALPYSTVGNSNNYLHLSVLRTELRPGETLNVNFLLRMDRAHEAKIRYYTYLIMNKGRLLKAG.R | Complement C3 | BCP(HP) | 4.93 | 6.56 |
| R.IPIEDGSGEVVLSRKVLLDGVQNP(→L)R.A | Complement C3 | BCP(HP) | 4.21 | 3.70 |
| R.TKKQELSEAEQATRTMQALPYSTVGNSNNYLHLSVLRTELRPGETLNVNFLLRMDRAHEAKIRYYTYLIMNKGRLLK.A | Complement C3 | BCP(HP) | 5.32 | 6.40 |
| A.LPYSTVGNSNNYLHLSVLRTELRPGETLNVNFLLRMDRAHEAKIRYYTYLIMNKGRLLKAGR.Q | Complement C3 | BCP(HP) | 5.00 | 6.30 |
| K.IWDVVEKADIGC(−dehydro)TPGSGKDYAGVFSDAGLTFTSSSGQQTAQR.A;  R:AELQC(-dehydro)PQPAA.R | Complement C3 | BCP(HP) | 5.22 | 6.04 |
| K.IEGDHGARVVLVAVDKGVFVLNKKNKLTQSKIWDVVEKADIGC(−dehydro)TPGSGKDYAGVFSDAGLTFTSSSGQQTAQRAELQC(−dehydro)PQPAA.R | Complement C3 | BCP(HP) | 5.26 | 5.86 |
| R.SGIPIVTSPYQIHFTKTPKYFKPGMPFDLM(−hydroxylation)VFVTNPDGSPAYRVPVAVQGEDTVQSLTQGDGVAKLSINTHPSQKPLSITVR.T | Complement C3 | BCP(HP) | 4.92 | 5.75 |
| Y.STVGNSNNYLHLSVLRTELRPGETLNVNFLLRMDRAHEAKIRYYTYLIMNKGRLLKAGR.Q | Complement C3 | BCP(HP) | 5.71 | 6.90 |
| T.VGNSNNYLHLSVLRTELRPGETLNVNFLLRMDRAHEAKIRYYTYLIMNKGRLLKAGR.Q | Complement C3 | BCP(HP) | 5.56 | 6.72 |
| L.PYSTVGNSNNYLHLSVLRTELRPGETLNVNFLLRMDRAHEAKIRYYTYLIM(−hydroxylation)NKGRLLKAGR.Q | Complement C3 | BCP(HP) | 5.51 | 6.36 |
| L.PYSTVGNSNNYLHLSVLRTELRPGETLNVNFLLRM(−hydroxylation)DRAHEAKIRYYTYLIMNKGRLLKAGR.Q | Complement C3 | BCP(HP) | 5.61 | 6.36 |
| K.SGQSEDRQPVPGQQMTLKIEGDHGARVVLVAVDKGVFVLNKKNKLTQSKIWDVVEKADIGC(−dehydro)TPGSGKDYAGVFSDAGLTFTSSSGQQTAQRAELQC(−dehydro)PQPAA.R | Complement C3 | BCP(HP) | 5.94 | 7.00 |
| R.KVLLDGVQNPRAEDLVGKSLYVSATVILHSGSDM(-hydroxylation)VQAER.S | Complement C3 | BCP(HP) | 5.83 | 5.61 |
| I.PPADLSDQVPDTESETRILLQGTPVAQ(-dehydration).M | Complement C3 | BCP(HP) | 6.20 | 7.18 |
| R.TKKQELSEAEQATRTMQALPYSTVGNSNNYLHLSVLRTELRPGETLNVNFLLRMDRAHEAKIRYYTYLIM(-hydroxylation)NKGRLLKAGR.Q | Complement C3 | BCP(HP) | 6.08 | 6.71 |
| R.TKKQELSEAEQATRTM(−hydroxylation)QALPYSTVGNSNNYLHLSVLRTELRPGETLNVNFLLRMDRAHEAKIRYYTYLIMNKGRLLKAGR.Q | Complement C3 | BCP(HP) | 6.08 | 6.71 |
| R.TM(−hydroxylation)QALPYSTVGNSNNYLHLSVLRTELRPGETLNVNFLLRMDRAHEAKIRYYTYLIMNKGRLLKAGR.Q | Complement C3 | BCP(HP) | 6.08 | 6.45 |
| R.ILLQGTPVAQM(-oxid)TEDAVDAERLKHLIVTPSG.C | Complement C3 | BCP(HP) | 5.99 | 6.15 |
| R.KVLLDGVQNPRAEDLVGKSLYVSATVILHSGSDMVQAERSGIPIVTSPYQIHFTKTPKYFKPGM(−hydroxylation)PFDLMVFVTNPDGSPAYRVPVAVQGEDTVQSLTQGDGVAKLSINTHPSQKPLSITVR.T | Complement C3 | BCP(HP) | 6.32 | 6.56 |
| R.SGIPIVTSPYQIHFTKTPKYFKPGMPFDLMVFVTNPDGSPAYRVPVAVQGEDTVQSLTQGDGVAKLSINTHPSQKPLSITVR.T | Complement C3 | BCP(HP) | 6.41 | 7.30 |
| R.TVMVNIENPEGIPVKQDSLSSQNQLGVLPLSWDIPELVNM(-hydroxylation)GQWKIR.A | Complement C3 | HP | 6.00 | 4.25 |
| R.TLEIPGNSDPNMIPDGDFNSYVR.V | Complement C4-A | BCP | 4.31 | 7.04 |
| R.GSFEFPVGDAVSKVLQIEKEGAIHREELVYELNPLDH.R | Complement C4-A | BCP | 4.28 | 6.41 |
| R.TTNIQGINLLFSSRRGHLFLQTDQPIYNPGQRVRYRVFALDQKMRPSTDTITVMVENSHGLR.V | Complement C4-A | BCP | 4.29 | 6.15 |
| R.GSFEFPVGDAVSKVLQIEKEGAIHREELVYELNPLDHRG.R | Complement C4-A | BCP | 4.13 | 5.81 |
| K.SHALQLNNRQIRGLEEELQFSLGSKINVKVGGNSKGTLK.V | Complement C4-A | BCP | 4.15 | 5.45 |
| R.VFALDQKMRPSTDTITVMVENSHGLR.V | Complement C4-A | BCP | 4.00 | 5.41 |
| K.VLQIEKEGAIHREELVYELNPLDH.R | Complement C4-A | BCP | 4.17 | 5.23 |
| I.PGNSDPNMIPDGDFNSYVR.V | Complement C4-A | BCP | 4.35 | 6.43 |
| R.AVGSGATFSHYYYMILSRGQIVFMNREPKRTLTSVSVFVDHHLAPSFYFVAFYYHGDHPVANSLR.V | Complement C4-A | BCP | 4.45 | 6.15 |
| R.NGFKSHALQLNNRQIRGLEEELQFSLGSKINVKVGGNSKGTLKVL.R | Complement C4-A | BCP | 4.34 | 5.99 |
| R.LLLFSPSVVHLGVPLSVGVQLQDVPR.G | Complement C4-A | BCP | 4.58 | 5.92 |
| D.PLDTLGSEGALSPGGVASLLR.L | Complement C4-A | BCP | 4.56 | 5.59 |
| K.SHALQLNNRQIRGLEEELQFSLGSKINVKVGGNSKGTLKVLR.T | Complement C4-A | BCP&HP | 5.40 | 6.04 |
| L.SPGGVASLLR.L | Complement C4-A | BCP&HP | 5.45 | 6.00 |
| Q.KPRLLLFSPSVVHLGVPLSVGVQLQDVPRGQVVKGSVFLR.N | Complement C4-A | BCP&HP | 5.74 | 6.00 |
| R.ALEILQEEDLIDEDDIPVR.S | Complement C4-A | BCP&HP | 5.85 | 6.90 |
| R.VTASDPLDTLGSEGALSPGGVASLLRLPR.G | Complement C4-A | BCP&HP | 5.76 | 6.67 |
| S.PGGVASLLR.L | Complement C4-A | BCP&HP | 5.90 | 6.40 |
| R.QIRGLEEELQFSLGSKINVKVGGNSKGTLKVLR.T | Complement C4-A | BCP&HP | 5.87 | 6.36 |
| R.GLEEELQFSLGSKINVKVGGNSKGTLKVLR.T | Complement C4-A | BCP&HP | 6.15 | 6.62 |
| R.NGFKSHALQLNNRQIRGLEEELQFSLGSKINVKVGGNSKGTLKVLR.T | Complement C4-A | BCP&HP | 6.30 | 6.69 |
| R.GSFEFPVGDAVSKVLQIEKEGAIHREELVYELNPLDHR.G | Complement C4-A | BCP&HP | 6.56 | 7.53 |
| R.VTASDPLDTLGSEGALSPGGVASLLR.L | Complement C4-A | BCP&HP | 6.38 | 6.68 |
| I.PDGDFNSYVR.V | Complement C4-A | BCP&HP | 7.08 | 7.08 |
| R.VFALDQKMRPSTDTITVMVENSHGL.R | Complement C4-A | BCP(HP) | 4.98 | 5.53 |
| R.QIRGLEEELQFSLGSKINVKVGGNSKGTLKVL.R | Complement C4-A | BCP(HP) | 4.70 | 5.73 |
| Q.KPRLLLFSPSVVHLGVPLSVGVQLQDVPRGQVVKGSVFL.R | Complement C4-A | BCP(HP) | 5.18 | 6.61 |
| Q.KPRLLLFSPSVVHLGVPLSVGVQLQDVPR.G | Complement C4-A | BCP(HP) | 5.73 | 6.76 |
| R.GLEEELQFSLGSKINVKVGGNSKGTLKVL.R | Complement C4-A | BCP(HP) | 5.69 | 6.72 |
| R.GLEEELQFSLGSKINVKVGGNSKGTLK.V | Complement C4-A | BCP(HP) | 5.51 | 6.49 |
| R.YLDKTEQWSTLPPETKDHAVDLIQK.G | Complement C4-A | BCP(HP) | 5.51 | 6.30 |
| R.VRYRVFALDQKMRPSTDTITVMVENSHGLR.V | Complement C4-A | BCP(HP) | 5.69 | 6.20 |
| R.GLEEELQFSLGSKINVK.V | Complement C4-A | BCP(HP) | 5.72 | 5.94 |
| E.FPVGDAVSKVLQIEKEGAIHREELVYELNPLDHR.G | Complement C4-A | BCP(HP) | 5.33 | 6.18 |
| L.PPETKDHAVDLIQK.G | Complement C4-A | BCP(HP) | 5.04 | 5.85 |
| F.PVGDAVSKVLQIEKEGAIHREELVYELNPLDHR.G | Complement C4-A | BCP(HP) | 5.78 | 6.67 |
| L.QFSLGSKINVKVGGNSKGTLKVLR.T | Complement C4-A | HP(BCP) | 5.36 | 5.53 |
| L.EEELQFSLGSKINVKVGGNSKGTLKVLR.T | Complement C4-A | HP(BCP) | 5.00 | 5.51 |
| R.NGESVKLHLETDSLALVALGALDTALYAAGSKSHKPLNMGKVFEAMNSYDLGC(−dehydro)GPGGGDSALQVFQAAGLAFSDGDQWTLSRKRLSC(−dehydro)PKEKTT.R | Complement C4-A | BCP | 4.26 | 6.26 |
| Q.FSLGSKINVKVGGNSKGTLKVLR.T | Complement C4-A | BCP | 4.18 | 5.32 |
| R.LTVAAPPSGGPGFLSIERPDSRPPRVGDTLNLNL.R | Complement C4-A | BCP | 4.51 | 6.43 |
| Q.KPRLLLFSPSVVHLGVPLSVGVQLQDVPRGQVVKGSVFLRNPSRNNVPC(−dehydro)SPKVDFTLSSERDFALLSLQVPLKDAKSC(−dehydro)GLHQLLRGPEVQLVAHSPWLKDSLSR.T | Complement C4-A | BCP&HP | 6.30 | 6.94 |
| R.GPEVQLVAHSPWLKDSLSRTTNIQGINLLFSSRRGHLFLQTDQPIYNPGQR.V | Complement C4-A | BCP(HP) | 4.65 | 5.75 |
| Q.KPRLLLFSPSVVHLGVPLSVGVQLQDVPRGQVVKGSVFLRNPSRNNVPC(−dehydro)SPKVDFTLSSERDFALLSLQVPLKDAKSC(−dehydro)GLHQLLR.G | Complement C4-A | BCP(HP) | 5.58 | 6.48 |
| R.STQDTVIALDALSAYWIASHTTEER(-dihydroxylation)GLNVTLSSTGRNG.F | Complement C4-A | BCP(HP) | 4.63 | 5.42 |
| R.SIVSALKREALVKGNPPIYRFWKDNLQHKDSSVPNTGTAR.M | Complement C5 | BCP(HP) | 5.04 | 5.76 |
| R.SLKYNPVVIDFEMQPIHEVLRHTSLGPLEAKRQNLR.R | Complement C8 α | BCP | 4.16 | 5.54 |
| R.SLKYNPVVIDFEMQPIHEVLR.H | Complement C8 α | BCP | 4.43 | 5.81 |
| R.SLKYNPVVIDFEMQPIHEVLRHTSLGPLEAKRQNLRR.A | Complement C8 α | BCP | 4.33 | 5.67 |
| R.SLKYNPVVIDFEM(-hydroxylation)QPIHEVLRHTSLGPLEAKRQNLR.R | Complement C8 α | HP(BCP) | 5.77 | 5.48 |
| R.SLKYNPVVIDFEMQPIHEVLRHTSLGPLEAKRQNL.R | Complement C8 α | BCP(HP) | 4.90 | 6.52 |
| R.RPASPISTIQPK.A | Complement C8 γ | BCP(HP) | 5.28 | 6.15 |
| R.RPWNVASLIYETKGEKNF.R | Complement C9 | BCP | 4.22 | 5.80 |
| R.RPWNVASLIYETKGEKNFR.T | Complement C9 | BCP&HP | 5.57 | 5.85 |
| R.TEHYEEQIEAFK.S | Complement C9 | BCP(HP) | 5.00 | 5.54 |
| A.LFVSEEEKKLT.R | Complement factor B | BCP | 4.29 | 5.40 |
| R.KEVYIKNGDKKGSCERDAQYAPGYDKVKDISEVVTPR.F | Complement factor B | BCP | 4.39 | 5.52 |
| K.ALFVSEEEKKLT.R | Complement factor B | BCP&HP | 5.69 | 6.36 |
| R.DAQYAPGYDKVKDISEVVTPR.F | Complement factor B | BCP(HP) | 5.41 | 5.61 |
| Y.APGYDKVKDISEVVTPR.F | Complement factor B | BCP(HP) | 5.11 | 5.58 |
| R.QKQVPAHARDFHINLFQVLPWLKEKLQDEDLGFL.- | Complement factor B | HP(BCP) | 6.93 | 7.08 |
| R.LPPT(-acetylhexosamine)TTC(-dehydro)QQQKEELLPAQDIKALFVSEEEKKLTRKEVYIKNGDKKGSC(-dehydro)ERDAQYAPGYDKVKDISEVVTPR.F | Complement factor B | BCP(HP) | 5.77 | 6.78 |
| R.GSQRRTC(-dehydro)QEGGSWSGTEPSC(-dehydro)QDSFMYDTPQEVAEAFLSSLTETIEGVDAEDGHGPGEQQKR.K | Complement factor B | BCP(HP) | 6.23 | 6.57 |
| A.NFSTLLMNLGPENC(-dehydro)ATLLLFVLLESKILLHSLRPAVLTGVAEAVVAMIFPFQWQC(-dehydro)PYIPLCP.L | DENN domain-containing protein 4C | BCP(HP) | 5.07 | 6.36 |
| R.SSDPAFRILEDGSIYTTHDLILSSERKSFSIFLSDGQR.R | Desmocollin-1 | BCP | 4.21 | 5.43 |
| .G.Q.RQLRPEHFQEVGYAA.P.P.S.P | Extracellular matrix protein | BCP(HP) | 5.52 | 5.54 |
| R.DINKDRKDGYVLR.L | Fetuin B | BCP | 3.96 | 6.46 |
| R.GSVQYLPDLDDKNSQEKGPQEAFPVHLDLTTNPQGETLDISFLFLEPMEEKLVVLPFPKEKAR.T | Fetuin B | BCP | 4.45 | 6.66 |
| R.GSVQYLPDLDDKNSQEKGPQEAFPVHLDLTTNPQGETLDISFLFLEPM(−hydroxylation)EEKLVVLPFPKEKAR.T | Fetuin B | BCP&HP | 6.68 | 6.38 |
| K.SYKMADEAGSEADHEGTHSTKRGHAKSRPV.R | Fibrinogen α chain | BCP | 3.91 | 6.26 |
| R.GDSTFESKSYKMADEAGSEADHEGTHSTK.R | Fibrinogen α chain | BCP | 3.74 | 5.91 |
| R.HRHPDEAAFFDTASTGKTFPGFFSPMLGEFVSETESRGSESGIFTNTKESSSHHPGIAEFPSR.G | Fibrinogen α chain | BCP | 4.32 | 5.83 |
| R.HRHPDEAAFFDTASTGKTFPGFFSPMLGEFVSETESRGSESGIFTNTKESSSHHPGIAEFPSRG.K | Fibrinogen α chain | BCP | 4.29 | 5.82 |
| R.GSAGHWTSESSVSGSTGQWHSESGSFRPD.S | Fibrinogen α chain | BCP | 4.22 | 5.48 |
| S.ESGIFTNTKESSSHHPGIAEFPSRGK.S | Fibrinogen α chain | BCP | 4.27 | 5.46 |
| R.GDSTFESKSYKMADEAGSEADHEGTHSTKRGHAK.S | Fibrinogen α chain | BCP | 4.26 | 5.36 |
| R.HRHPDEAAFFDTASTG.K | Fibrinogen α chain | BCP | 4.26 | 5.11 |
| R.EYHTEKLVTSKGDKEL.R | Fibrinogen α chain | BCP | 4.21 | 5.08 |
| R.HRHPDEAAFFDTASTGKTFPGFFSPMLGEFVSETESRGSESGIFTNTKESSSHHPGIAEFPSRGK.S | Fibrinogen α chain | BCP | 4.38 | 6.86 |
| R.HRHPDEAAFFDTASTGKTFPGFFSPMLGEFVSETESRGSESGIFTNTKESSSHHPGIAEFPS.R | Fibrinogen α chain | BCP | 4.37 | 6.45 |
| R.HRHPDEAAFFDTASTGKTFPGFFSPMLGEFVSETESR.G | Fibrinogen α chain | BCP | 4.33 | 6.04 |
| K.SSSYSKQFTSSTSYN.R | Fibrinogen α chain | BCP&HP | 5.34 | 5.67 |
| S.STSYNRGDSTFESKSYK.M | Fibrinogen α chain | BCP&HP | 5.20 | 5.26 |
| R.HRHPDEAAFFDTASTGK.T | Fibrinogen α chain | BCP&HP | 5.61 | 5.93 |
| H.TEKLVTSKGDKELR.T | Fibrinogen α chain | BCP&HP | 5.58 | 5.51 |
| R.GSESGIFTNTKESSSHHPGIAEFPSRG.K | Fibrinogen α chain | BCP&HP | 5.78 | 6.11 |
| S.GIFTNTKESSSHHPGIAEFPSRGK.S | Fibrinogen α chain | BCP&HP | 5.88 | 5.77 |
| R.EYHTEKLVTSKGDKELR.T | Fibrinogen α chain | BCP&HP | 6.15 | 7.91 |
| R.REYHTEKLVTSKGDKEL.R | Fibrinogen α chain | BCP&HP | 6.08 | 6.43 |
| K.SSSYSKQFTSSTSYNRGDSTFESKS.Y | Fibrinogen α chain | BCP&HP | 6.20 | 6.48 |
| K.SSSYSKQFTSSTSYNRGDSTFESKSYK.M | Fibrinogen α chain | BCP&HP | 6.34 | 6.30 |
| K.SSSYSKQFTSSTSYNRGDSTFESK.S | Fibrinogen α chain | BCP&HP | 6.66 | 6.85 |
| R.GSESGIFTNTKESSSHHPGIAEFPSRGK.S | Fibrinogen α chain | BCP&HP | 6.66 | 6.56 |
| K.SSSYSKQFTSSTSYNRGDSTFESKSY.K | Fibrinogen α chain | BCP&HP | 6.53 | 6.26 |
| R.GSESGIFTNTKESSSHHPGIAEFPSR.G | Fibrinogen α chain | BCP&HP | 6.45 | 6.15 |
| V.SETESRGSESGIFTNTKESSSHHPGIAEFPSRGK.S | Fibrinogen α chain | BCP&HP | 6.41 | 5.75 |
| R.REYHTEKLVTSKGDKELR.T | Fibrinogen α chain | BCP&HP | 7.00 | 6.75 |
| R.TGKEKVTSGSTTTTR.R | Fibrinogen α chain | BCP&HP | 6.80 | 6.72 |
| I.FTNTKESSSHHPGIAEFPSRGK.S | Fibrinogen α chain | BCP(HP) | 5.85 | 5.66 |
| R.GKSSSYSKQFTSSTSYN.R | Fibrinogen α chain | BCP(HP) | 5.38 | 6.79 |
| R.GSESGIFTNTKESSSHHPGIAEFPS.R | Fibrinogen α chain | BCP(HP) | 5.56 | 5.80 |
| K.SSSYSKQFTSSTSYNRGDSTFESKSYKMADEAGSEADHEGTHSTKR.G | Fibrinogen α chain | BCP(HP) | 4.60 | 5.98 |
| K.QFTSSTSYNRGDSTFESK.S | Fibrinogen α chain | BCP(HP) | 5.08 | 6.38 |
| P.GSGNARPNNPDWGTFEEVSGNVSPGTR.R | Fibrinogen α chain | BCP(HP) | 4.88 | 5.45 |
| K.SSSYSKQFTSSTSYNR.G | Fibrinogen α chain | BCP(HP) | 5.82 | 6.75 |
| F.TSSTSYNRGDSTFESKSYK.M | Fibrinogen α chain | HP | 4.93 | 4.40 |
| V.SETESRGSESGIFTNTKESSSHHPGIAEFPSRG.K | Fibrinogen α chain | HP | 5.53 | 4.44 |
| V.SETESRGSESGIFTNTKESSSHHPGIAEFPSR.G | Fibrinogen α chain | HP | 5.89 | 4.55 |
| R.MELERPGGNEITR.G | Fibrinogen α chain | HP | 5.93 | 3.89 |
| R.TGKEKVTSGSTTTT.R | Fibrinogen α chain | HP | 6.18 | 3.83 |
| F.VSETESRGSESGIFTNTKESSSHHPGIAEFPSRGK.S | Fibrinogen α chain | HP(BCP) | 5.73 | 5.46 |
| R.NPSSAGSWNSGSSGPGSTGNR.N | Fibrinogen α chain | HP(BCP) | 5.30 | 6.15 |
| S.TSYNRGDSTFESKSYK.M | Fibrinogen α chain | HP(BCP) | 5.04 | 5.11 |
| T.SYNRGDSTFESKSYK.M | Fibrinogen α chain | HP(BCP) | 5.04 | 5.04 |
| R.GKSSSYSKQFTSSTSYNRGDSTFESK.S | Fibrinogen α chain | HP(BCP) | 5.20 | 4.84 |
| E.TESRGSESGIFTNTKESSSHHPGIAEFPSRGK.S | Fibrinogen α chain | HP(BCP) | 5.67 | 4.93 |
| R.GKSSSYSKQFTSSTSYNRGDSTFESKSYK.M | Fibrinogen α chain | HP(BCP) | 5.88 | 5.53 |
| H.PGIAEFPSRGK.S | Fibrinogen α chain | HP(BCP) | 5.81 | 5.30 |
| E.SGIFTNTKESSSHHPGIAEFPSRGK.S | Fibrinogen α chain | HP(BCP) | 6.15 | 5.93 |
| D.SPGSGNARPNNPDWGTFEEVSGNVSPGTR.R | Fibrinogen α chain | HP(BCP) | 5.97 | 5.43 |
| R.SC(-dehydro)SKTVTKTVIGPDGHKEVTKEVVTSEDGSDC(-dehydro)PEAM(-hydroxylation)DLGTLSGIGTLDGFR.H | Fibrinogen α chain | HP(BCP) | 6.11 | 6.68 |
| R.HRHPDEAAFFDTASTGKT(-dehydration)FPGF.F | Fibrinogen α chain | HP(BCP) | 5.98 | 5.30 |
| R.HRHPDEAAFFDTASTGKTFPGFFSPM(-hydroxylation)LGEFVSETESRGSESGIFTNTKESSSHHPGIAEFPSR.G | Fibrinogen α chain | HP(BCP) | 6.63 | 5.91 |
| R.TGKEKVTSGSTTTTRRSC(-dehydro)SKTVTKTVIGPDGHKEVTKEVVTSEDGSDC(-dehydro)PEAMDLGTLSGIGTLDGFRHRHPDEAAFFDTASTGKTFPGFFSPM(-hydroxylation)LGEFVSETESRGSESGIFTNTKESSSHHPGIAEFPSRGK.S | Fibrinogen α chain | HP(BCP) | 6.58 | 5.11 |
| R.TGKEKVTSGSTTTTRRSCSKTVTKTVIGPDGHKEVTKEVVTSEDGSDCPEAM(-hydroxylation)DLGTLSGIGTLDGFR.H | Fibrinogen α chain | HP(BCP) | 7.15 | 6.44 |
| R.HRHPDEAAFFDTASTGKTFPGFFSPM(-hydroxylation)LGEFVSETESR.G | Fibrinogen α chain | HP(BCP) | 6.76 | 6.18 |
| K.SSSYSKQFTSSTSYNRGDSTFESKSYKM(−hydroxylation)ADEAGSEADHEGTHSTKR.G | Fibrinogen α chain | HP(BCP) | 7.04 | 6.15 |
| R.HRHPDEAAFFDTASTGKT(-dehydration)FPGFFS.P | Fibrinogen α chain | HP(BCP) | 6.26 | 5.64 |
| R.HRHPDE(→Q)AAFFDTASTGKTFPGFFSPMLGEFVSETESRGSESGIFTNTKESSSHHPGIAEFPSRGK.S | Fibrinogen α chain | BCP | 4.30 | 6.23 |
| R.TGKEKVTSGSTTTTRRSC(-dehydro)SKTVTKTVIGPDGHKEVTKEVVTSEDGSDC(-dehydro)PEAMDLGTLSGIGTLDGFRHRHPDEAAFFDTASTGKTFPGFFSPMLGEFVSETESRGSESGIFTNTKESSSHHPGIAEFPSRGK.S | Fibrinogen α chain | BCP | 4.00 | 5.72 |
| R.TGKEKVTSGSTTTTRRSC(-dehydro)SKTVTKTVIGPDGHKEVTKEVVTSEDGSDC(-dehydro)PEAMDLGTLSGIGTLDGF.R | Fibrinogen α chain | BCP | 4.51 | 6.70 |
| R.TGKEKVTSGSTTTTRRSC(-dehydro)SKTVTKTVIGPDGHKEVTKEVVTSEDGSDC(-dehydro)PEAMDLGTLSGIGTLDGFR.H | Fibrinogen α chain | BCP | 4.40 | 6.68 |
| R.RSC(-dehydro)SKTVTKTVIGPDGHKEVTKEVVTSEDGSDC(-dehydro)PEAM(-hydroxylation)DLGTLSGIGTLDGFR.H | Fibrinogen α chain | BCP&HP | 7.15 | 6.46 |
| R.TGKEKVTSGSTTTTRRSC(-dehydro)SKTVTKTVIGPDGHKEVTKEVVTSEDGSDC(-dehydro)PEAM(-hydroxylation)DLGTLSGIGTLDGFR.H | Fibrinogen α chain | BCP&HP | 7.20 | 6.40 |
| R.HRHPDEAAFFDTASTGKTFPGFFSPM(-hydroxylation)LGEFVSETESRGSESGIFTNTKESSSHHPGIAEFPSRGK.S | Fibrinogen α chain | BCP&HP | 6.69 | 6.26 |
| R.GSESGIFTNTKESSSHHPGIAE(-dehydration)F.P | Fibrinogen α chain | BCP(HP) | 5.24 | 4.88 |
| R.RSC(−dehydro)SKTVTKTVIGPDGHKEVTKEVVTSEDGSDC(−dehydro)PEAMDLGTLSGIGTLDGF.R | Fibrinogen α chain | BCP(HP) | 4.20 | 3.70 |
| R.REYHTEKLVTSKGDK(-H2O).E | Fibrinogen α chain | BCP(HP) | 5.26 | 5.73 |
| R.RSC(−dehydro)SKTVTKTVIGPDGHKEVTKEVVTSEDGSDC(−dehydro)PEAMDLGTLSGIGTLDGFR.H | Fibrinogen α chain | BCP(HP) | 5.55 | 6.56 |
| R.TGKEKVTSGSTTTTRRSC(−dehydro)SKTVTKTVIGPDGHKEVTKEVVTSEDGSDC(−dehydro)PEAM(-hydroxylation)DLGTLSGIGTLDGFRHRHPDEAAFFDTASTGK.T | Fibrinogen α chain | HP | 5.85 | 4.35 |
| R.RSC(−dehydro)SKTVTKTVIGPDGHKEVTKEVVTSEDGSDC(−dehydro)PEAMDLGTLSGIGTLDGFRHRHPDEAAFFDTASTGKTFPGFFSPM(−hydroxylation)LGEFVSETESRGSESGIFTNTKESSSHHPGIAEFPSRGK.S | Fibrinogen α chain | HP | 6.15 | 4.40 |
| R.TGKEKVTSGSTTTTRRSC(−dehydro)SKTVTKTVIGPDGHKEVTKEVVTSEDGSDC(−dehydro)PEAM(hydroxylation)DLGTLSGIGTLDGFRHRHPDEAAFFDTASTGKTFPGFFSPM(-hydroxylation)LGEFVSETESRGSESGIFTNTKESSSHHPGIAEFPSRGK.S | Fibrinogen α chain | HP | 6.45 | 5.44 |
| R.GHRPLDKKREEAPSL.R | Fibrinogen β chain | BCP&HP | 6.30 | 6.82 |
| R.GHRPLDKKREEAPSLRPAPPPISGGGY.R | Fibrinogen β chain | BCP&HP | 7.11 | 7.34 |
| R.GHRPLDKKREEAPSLRPAPPPISGGG.Y | Fibrinogen β chain | BCP(HP) | 5.99 | 6.72 |
| V.NDNEEGFFSAR.G | Fibrinogen β chain | HP | 5.58 | 4.28 |
| R.GHRPLDKKREEAPSLRPAPPPISGGGYR.A | Fibrinogen β chain | HP(BCP) | 6.91 | 5.27 |
| R.GHRPLDKKREEAPS(-dehydration)LRPAPP.P | Fibrinogen β chain | BCP&HP | 4.41 | 5.72 |
| S.Q(pyro-glus)GVNDNEEGFFSAR.G | Fibrinogen β chain | HP | 6.20 | 4.27 |
| K.ASTPNGYDNGIIWATWKTRWYSMKKTTMKIIPFNRLTIGEGQQHHLGGAKQAGDV.- | Fibrinogen γ chain | BCP(HP) | 4.84 | 5.89 |
| T.ADSGEGDFLAEGGGVR.G | Fibrinopeptide A | BCP(HP) | 4.19 | 6.23 |
| R.VPFDAATLHTSTAMAAQHGMDDDGTGQKQIWR.I | Gelsolin | BCP | 4.06 | 6.00 |
| R.KAALKTASDFITKMDYPKQTQVSVLPEGGETPLFK.Q | Gelsolin | BCP | 4.19 | 5.95 |
| R.ANSAGATRAVEVLPKAGALNSNDAFVLKTPSAAYLWVGTGASEAEKTGAQELLRVL.R | Gelsolin | BCP | 4.06 | 5.89 |
| R.VPFDAATLHTSTAMAAQHGMDDDGTGQKQIW.R | Gelsolin | BCP | 4.23 | 5.88 |
| L.WVGTGASEAEKTGAQELLR.V | Gelsolin | BCP | 4.29 | 5.63 |
| L.QVLGPKPALPAGTEDTAKEDAANR.K | Gelsolin | BCP | 4.23 | 5.23 |
| V.GTGASEAEKTGAQELLRVLR.A | Gelsolin | BCP | 4.35 | 6.08 |
| K.AGALNSNDAFVLKTPSAAYLWVGTGASEAEKTGAQELLR.V | Gelsolin | BCP | 4.37 | 5.82 |
| R.VVQGKEPAHLMSLFGGKPMIIYK.G | Gelsolin | BCP | 4.37 | 5.81 |
| R.VLRAQPVQVAEGSEPDGFWEALGGKAAYR.T | Gelsolin | BCP | 4.43 | 5.81 |
| R.AVQHREVQGFESATFLGYFKSGLKYKKGGVASGFKHVVPNEVVVQRLFQVKG.R | Gelsolin | BCP | 4.53 | 5.81 |
| K.TASDFITKMDYPKQTQVSVLPEGGETPLFKQFFK.N | Gelsolin | BCP | 4.51 | 5.76 |
| R.IEGSNKVPVDPATYGQFYGGDSYIILYNYR.H | Gelsolin | BCP&HP | 5.64 | 5.76 |
| R.RVVRATEVPVSWESFNNGDC(-dehydro)FILDLGNNIHQWC(-dehydro)GSNSNRYERLKATQVSKGIRDNERSGRAR.V | Gelsolin | BCP&HP | 5.46 | 5.48 |
| R.AVQHREVQGFESATFLGYFKSGLKYKKGGVASGFK.H | Gelsolin | BCP&HP | 5.92 | 6.45 |
| V.GTGASEAEKTGAQELLR.V | Gelsolin | BCP&HP | 5.82 | 6.20 |
| R.VVQGKEPAHLMSLFGGKPMIIYKGGTSREGGQTAPASTRLFQVR.A | Gelsolin | BCP&HP | 6.04 | 6.65 |
| K.NWRDPDQTDGLGLSYLSSHIANVER.V | Gelsolin | BCP&HP | 6.04 | 6.53 |
| R.AVQHREVQGFESATFLGYFKSGLKYKKGGVASGFKHVVPNEVVVQRLFQVKGR.R | Gelsolin | BCP&HP | 6.00 | 6.45 |
| V.VPNEVVVQRLFQVKGR.R | Gelsolin | BCP&HP | 6.04 | 6.34 |
| R.IEGSNKVPVDPATYGQFYGGDSYIILYNYRHGGRQGQIIYNWQGAQSTQDEVAASAILTAQLDEELGGTPVQSR.V | Gelsolin | BCP&HP | 6.04 | 5.86 |
| V.PNEVVVQRLFQVKGR.R | Gelsolin | BCP&HP | 6.26 | 6.67 |
| K.HVVPNEVVVQRLFQVKGR.R | Gelsolin | BCP&HP | 6.36 | 6.53 |
| R.ANSAGATRAVEVLPKAGALNSNDAFVLKTPSAAYLWVGTGASEAEKTGAQELLR.V | Gelsolin | BCP&HP | 6.53 | 6.93 |
| R.ANSAGATRAVEVLPKAGALNSNDAFVLKTPSAAYLWVGTGASEAEKTGAQELLRVLR.A | Gelsolin | BCP&HP | 6.38 | 6.88 |
| K.PMIIYKGGTSREGGQTAPASTRLFQVR.A | Gelsolin | BCP(HP) | 4.52 | 6.08 |
| K.QFFKNWRDPDQTDGLGLSYLSSHIANVER.V | Gelsolin | BCP(HP) | 4.41 | 5.62 |
| R.AQPVQVAEGSEPDGFWEALGGK.A | Gelsolin | BCP(HP) | 4.59 | 5.36 |
| R.VVQGKEPAHLMSLFGGKPMIIYKGGTSREGGQTAPASTRLFQV.R | Gelsolin | BCP(HP) | 5.30 | 7.41 |
| K.HVVPNEVVVQRLFQVKG.R | Gelsolin | BCP(HP) | 5.20 | 6.80 |
| L.SYLSSHIANVER.V | Gelsolin | BCP(HP) | 4.88 | 6.00 |
| R.AVQHREVQGFESATFLGYFKSGLKYKKGGVASGF.K | Gelsolin | BCP(HP) | 4.84 | 5.81 |
| K.TPSAAYLWVGTGASEAEKTGAQELLRVLR.A | Gelsolin | BCP(HP) | 5.04 | 5.72 |
| R.DPDQTDGLGLSYLSSHIANVER.V | Gelsolin | BCP(HP) | 5.08 | 5.64 |
| R.AVQHREVQGFESATFLGYFKSGLKYKKGGVASGFKHVVPNEVVVQRLFQVKGRRVVR.A | Gelsolin | BCP(HP) | 5.34 | 5.54 |
| H.VVPNEVVVQRLFQVKGR.R | Gelsolin | BCP(HP) | 5.70 | 6.08 |
| R.AVEVLPKAGALNSNDAFVLKTPSAAYLWVGTGASEAEKTGAQELLR.V | Gelsolin | BCP(HP) | 5.72 | 5.90 |
| R.VHVSEEGTEPEAMLQVLGPKPALPAGTEDTAKEDAANR.K | Gelsolin | BCP(HP) | 5.81 | 6.53 |
| R.ANSAGATRAVEVLPKAGALNSNDAFVLK.T | Gelsolin | BCP(HP) | 5.88 | 6.41 |
| R.ANSAGATRAVEVLPK.A | Gelsolin | BCP(HP) | 6.08 | 6.46 |
| A.ATASRGASQAGAPQGR.V | Gelsolin | BCP(HP) | 5.68 | 6.60 |
| Q.PVQVAEGSEPDGFWEALGGKAAYR.T | Gelsolin | HP | 5.32 | 4.33 |
| A.ATASRGASQAGAPQG.R | Gelsolin | HP | 6.00 | 4.04 |
| R.GASQAGAPQGR.V | Gelsolin | HP | 7.41 | 4.21 |
| K.AGALNSNDAFVLKTPSAAYLWVGTGASEAEKTGAQELLRVLR.A | Gelsolin | HP(BCP) | 5.72 | 6.45 |
| R.VVQGKEPAHLMSLFGGKPM(-hydroxylation)IIYKGGTSREGGQTAPASTRLFQV.R | Gelsolin | BCP | 4.33 | 6.20 |
| W.VGTGASEAEKTGAQELLR.V | Gelsolin | BCP&HP | 5.38 | 5.85 |
| R.RVVRATEVPVSWESFNNGDC(−dehydro)FILDLGNNIHQWC(−dehydro)GSNSNRYERLKATQVSKGIRDNER.S | Gelsolin | BCP&HP | 5.83 | 6.04 |
| R.RVVRATEVPVSWESFNNGDC(−dehydro)FILDLGNNIHQWC(−dehydro)GSNSNRYERLKATQVSKGIRDNERSGR.A | Gelsolin | BCP&HP | 5.94 | 5.94 |
| V.PVSWESFNNGDC(−dehydro)FILDLGNNIHQWC(−dehydro)GSNSNRYERLKATQVSKGIRDNER.S | Gelsolin | BCP(HP) | 4.84 | 5.67 |
| R.RVVRATEVPVSWESFNNGDC(−dehydro)FILDLGNNIHQWC(−dehydro)GSNSNRYERLK.A | Gelsolin | BCP(HP) | 5.64 | 5.73 |
| K.HVVPNEVVVQRLFQVKGRRVVRATEVPVSWESFNNGDC(−dehydro)FILDLGNNIHQWC(−dehydro)GSNSNRYERLKATQVSKGIRDNERSGRAR.V | Gelsolin | BCP(HP) | 5.41 | 5.72 |
| R.VHVSEEGTEPEAMLQVLGPKPALPAGTEDTAKEDAANRKLAKLYK.V | Gelsolin | BCP(HP) | 5.77 | 6.18 |
| R.ATEVPVSWESFNNGDC(-dehydro)FILDLGNNIHQWC(-dehydro)GSNSNRYERLKATQVSKGIRDNER.S | Gelsolin | BCP(HP) | 6.20 | 6.86 |
| K.TASDFITKM(-oxid)DYPKQTQVSVLPEGGETPLFKQFFK.N | Gelsolin | BCP(HP) | 6.20 | 6.00 |
| R.ANSAGATRAVEVLPKAGALNSNDAFVLKTPSAAYLWVGTGASEAEKTGAQELLRVLRAQPVQVAEGSEPDGFWEALGGKAAYR.T | Gelsolin | BCP(HP) | 6.04 | 6.08 |
| R.RVVRATEVPVSWESFN.N | Gelsolin | HP | 5.59 | 4.47 |
| G.VTVQDGNFSFSLESVKKLKDLQEPQEPR.V | Guanylin | BCP&HP | 5.57 | 5.78 |
| K.SHKWDRELISERWKNFPSPVDAAFR.Q | Hemopexin | BCP&HP | 5.78 | 5.66 |
| R.QGHNSVFLIKGDKVWVYPPEKKEKGYPK.L | Hemopexin | BCP&HP | 6.95 | 7.32 |
| P.PEKKEKGYPK.L | Hemopexin | BCP(HP) | 4.92 | 5.86 |
| F.PSPVDAAFRQGHNSVFLIKGDKVWVYPPEKKEKGYPK.L | Hemopexin | BCP(HP) | 5.48 | 5.97 |
| Y.PPEKKEKGYPK.L | Hemopexin | BCP(HP) | 5.91 | 6.41 |
| N.SVFLIKGDKVWVYPPEKKEKGYPK.L | Hemopexin | BCP(HP) | 5.88 | 6.32 |
| G.HNSVFLIKGDKVWVYPPEKKEKGYPK.L | Hemopexin | BCP(HP) | 5.18 | 5.99 |
| K.GDKVWVYPPEKKEKGYPK.L | Hemopexin | BCP(HP) | 4.94 | 5.83 |
| K.LLQDEFPGIPSPLDAAVEC(-dehydro)HRGEC(-dehydro)QAEGVLFFQGDREWFWDLATGTMKER.S | Hemopexin | BCP & HP | 5.76 | 6.65 |
| R.Q(-pyro-glu)GHNSVFLIKGDKVWVYPPEKKEKGYPK.L | Hemopexin | BCP&HP | 6.04 | 6.57 |
| R.QGHNSVFLIKGDKVWVYPPEKKEKGYPKLLQDEFPGIPSPLDAAVEC(−dehydro)HRGEC(−dehydro)QAEGVLFFQGDREWFWDLATGTMKER.S | Hemopexin | BCP(HP) | 5.65 | 6.41 |
| R.QGHNSVFLIKGD(-dehydration)KVWV.Y | Hemopexin | BCP(HP) | 4.97 | 4.27 |
| K.LLQDEFPGIPSPLDAAVEC(−dehydro)HRGEC(−dehydro)QAEGVLFFQGDREWFWDLATGTM(-hydroxylation)KER.S | Hemopexin | HP | 6.28 | 4.56 |
| R.SVPPSASHVAPTETFTYEWTVPKEVGPTNADPVCLAKMYYSAVEPTKDIFTGLIGPMKICKKGSLHANGRQKDVDKEFYLFPTVFDENESLLLEDNIR.M | Highly similar to ceruloplasmin | BCP(HP) | 6.45 | 6.63 |
| R.KQANKALEKYKEENDDFASFR.V | Histidine-rich glycoprotein | BCP&HP | 5.85 | 5.58 |
| R.GHVLAKELEAFR.E | Insulin-like growth factor II | HP(BCP) | 5.58 | 5.41 |
| R.VSDPKFHPLHSKIIIIKKGHAKDSQR.Y | Insulin-like growth factor-binding protein 3 | BCP | 4.20 | 5.34 |
| R.VSDPKFHPLHSKIIIIKKGHAKDSQRY.K | Insulin-like growth factor-binding protein 3 | BCP&HP | 5.70 | 5.49 |
| R.SAGSVESPSVSSTHR.V | Insulin-like growth factor-binding protein 3 | BCP(HP) | 4.61 | 5.68 |
| R.ISELKAEAVKKDRR.K | Insulin-like growth factor-binding protein 5 | BCP(HP) | 4.51 | 5.70 |
| R.AFWLDVSHNRLEALPNSLLAPLGRL.R | Insulin-like growth factor-binding protein complex acid labile chain | BCP | 4.31 | 5.89 |
| R.AFWLDVSHNRLEALPNSLLAPLGRLR.Y | Insulin-like growth factor-binding protein complex acid labile chain | BCP | 4.50 | 6.66 |
| K.ALRDFALQNPSAVPR.F | Insulin-like growth factor-binding protein complex acid labile chain | BCP | 4.60 | 5.43 |
| R.AFWLDVSHNRLEALPNSLLAPLGR.L | Insulin-like growth factor-binding protein complex acid labile chain | BCP&HP | 5.40 | 6.91 |
| Y.PQDAVLALTQNHHKQYYEGSEIVVAGR.I | ITI HC1 | BCP | 4.27 | 6.08 |
| R.AQRIYEDHDATQQLQGFYSQVAKPLLVDVDLQYPQDAVLALTQNHHKQYYEGSEIVVAG.R | ITI HC1 | BCP | 4.19 | 5.75 |
| R.IADNKQSSFK.A | ITI HC1 | BCP | 3.89 | 5.56 |
| Q.MSLDYGFVTPLTSMSIRGMADQDGLKPTIDKPSEDSPPLEMLGPR.R | ITI HC1 | BCP | 4.31 | 5.49 |
| R.NAIRGRFPLYNLGFGHNVDFNFLEVMSMENNGR.A | ITI HC1 | BCP | 4.18 | 5.38 |
| R.GFSLDEATNLNGGLLR.G | ITI HC1 | BCP | 4.30 | 5.30 |
| R.GMADQDGLKPTIDKPSEDSPPLEMLGPR.R | ITI HC1 | BCP | 4.57 | 7.45 |
| R.IYEDHDATQQLQGFYSQVAKPLLVDVDLQYPQDAVLALTQNHHKQYYEGSEIVVAG.R | ITI HC1 | BCP | 4.66 | 6.46 |
| R.IYEDHDATQQLQGFYSQVAKPLLVDVDLQYPQDAVLALTQNHHKQYYEGSEIVVAGR.I | ITI HC1 | BCP | 4.65 | 6.36 |
| R.KAAISGENAGLV.R | ITI HC1 | BCP | 4.37 | 6.18 |
| Q.MSLDYGFVTPLTSMSIR.G | ITI HC1 | BCP | 4.40 | 6.11 |
| S.PPLEMLGPR.R | ITI HC1 | BCP | 4.57 | 6.08 |
| R.SKSSEKRQAVDTAVDGVFI.R | ITI HC1 | BCP | 4.56 | 5.72 |
| R.SKSSEKRQAVDTAVDGVFIRSLKVNCKVTSRFAHYVVTSQVVNTANEAR.E | ITI HC1 | BCP | 4.41 | 5.67 |
| K.AAISGENAGLVR.A | ITI HC1 | BCP(HP) | 4.57 | 5.40 |
| R.RTFVLSALQPSPTHSSSNTQRLPDRVTGVDTD.P | ITI HC1 | BCP(HP) | 5.15 | 6.15 |
| R.SKSSEKRQAVDTAVDGVFIR.S | ITI HC1 | BCP(HP) | 5.08 | 5.74 |
| R.AQRIYEDHDATQQLQGFYSQVAKPLLVDVDLQYPQDAVLALTQNHHKQYYEGSEIVVAGR.I | ITI HC1 | BCP(HP) | 5.72 | 6.93 |
| R.KAAISGENAGLVR.A | ITI HC1 | BCP(HP) | 5.00 | 6.36 |
| R.EVAFDLEIPKTAFISDFAVTADGNAFIGDIKDKVTAWKQYR.K | ITI HC1 | BCP(HP) | 6.04 | 6.15 |
| Q.M(-oxid)SLDYGFVTPLTSMSIR.G | ITI HC1 | BCP | 4.30 | 5.49 |
| R.GM(-hydroxylation)ADQDGLKPTIDKPSEDSPPLEMLGPR.R | ITI HC1 | BCP(HP) | 5.46 | 7.45 |
| R.ITRSILQMSLDHHIVTPLTSLVIENEAGDER.M | ITI HC2 | BCP | 4.26 | 6.30 |
| R.MATTMIQSKVVNNSPQPQNVVFDVQIPK.G | ITI HC2 | BCP | 4.32 | 6.20 |
| K.RRITRSILQMSLDHHIVTPLTSLVIENEAGDER.M | ITI HC2 | BCP | 4.34 | 5.99 |
| K.VVNNSPQPQNVVFDVQIPK.G | ITI HC2 | BCP | 4.62 | 5.96 |
| R.NDLISATKTQVADAKR.Y | ITI HC2 | BCP | 4.45 | 5.61 |
| R.SILQMSLDHHIVTPLTSLVIENEAGDER.M | ITI HC2 | BCP&HP | 5.54 | 7.11 |
| R.IYGNQDTSSQLKKFYNQVSTPLLR.N | ITI HC2 | BCP&HP | 5.38 | 5.51 |
| G.FEIPINGLSEFVDYEDLVELAPGKFQLVAENR.R | ITI HC2 | BCP&HP | 6.34 | 6.28 |
| R.NDLISATKTQVADAK.R | ITI HC2 | BCP(HP) | 4.57 | 6.15 |
| R.AKGKTAGLVR.S | ITI HC2 | BCP(HP) | 5.62 | 5.67 |
| R.RITRSILQMSLDHHIVTPLTSLVIENEAGDER.M | ITI HC2 | BCP(HP) | 6.36 | 6.61 |
| G.FEIPIN(→D)GLSEFVDYEDLVELAPGKFQLVAENR.R | ITI HC2 | HP(BCP) | 5.79 | 5.51 |
| R.SSALDMENFRTEVNVLPGAKVQFELHYQEVKWRKLGSYEHRIYLQPGRLAKHLEVDVWVIEPQGLRFLHVPDTFEGHFDGVPVISKGQQKAHVSFKPTVAQQR.I | ITI HC2 | BCP | 4.33 | 6.18 |
| R.SILQM(-hydroxylation)SLDHHIVTPLTSLVIENEAGDER.M | ITI HC2 | BCP&HP | 5.83 | 6.32 |
| R.SILQMSLDHHIVTP(-hydroxylation)LTSLVIENEAGDER.M | ITI HC2 | BCP(HP) | 5.83 | 6.32 |
| R.GISMLNKAREEHRIPERSTSIVIMLTDGDANVGESRPEKIQENVR.N | ITI HC3 | BCP | 4.28 | 6.00 |
| R.NAIGGKFPLYNLGFGNNLNYNFLENMALENHGFA.R | ITI HC3 | BCP | 4.28 | 5.81 |
| R.NAIGGKFPLYNLGFGNNLNYNFLENMALENHGFAR.R | ITI HC3 | BCP | 4.21 | 5.49 |
| K.YYLQGAKIPKPEASFSPR.R | ITI HC4, isoform 1 | BCP&HP | 5.57 | 5.96 |
| I.PKPEASFSPR.R | ITI HC4, isoform 1 | BCP&HP | 5.51 | 5.80 |
| V.HSGSTFFKYYLQGAKIPKPEASFSPR.R | ITI HC4, isoform 1 | BCP&HP | 5.82 | 6.00 |
| R.NVHSGSTFFKYYLQGAKIPKPEASFSPR.R | ITI HC4, isoform 1 | BCP&HP | 7.00 | 7.20 |
| F.KYYLQGAKIPKPEASFSPR.R | ITI HC4, isoform 1 | BCP(HP) | 4.91 | 5.43 |
| Y.YLQGAKIPKPEASFSPR.R | ITI HC4, isoform 1 | BCP(HP) | 5.70 | 5.97 |
| H.SGSTFFKYYLQGAKIPKPEASFSPR.R | ITI HC4, isoform 1 | HP(BCP) | 5.67 | 5.97 |
| R.NRNVHSGSTFFKYYLQGAKIPKPEASFSPR.R | ITI HC4, isoform 1 | HP(BCP) | 5.73 | 5.76 |
| R.NVHSGSTFFKYYLQGAKIPKPGDK(−Aminoethylbenzenesulfonylation)VGGGGR(−deamidation)GD.- | ITI HC4, isoform 1 | HP(BCP) | 6.38 | 6.55 |
| R.NVHSGSTFFKYYLQGAKIPKPEASFS(-acetylhexosamine)PR.R | ITI HC4, isoform 1 | HP(BCP) | 6.85 | 7.11 |
| R.N(→D)VHSGSTFFKYYLQGAKIPKPEASFSPR.R | ITI HC4, isoform 1 | BCP&HP | 5.95 | 6.60 |
| R.NVHSGSTFFKYYLQGAKIPKPEASFS(→G)P(→H)RRGWN.R | ITI HC4, isoform 1 | BCP&HP | 6.99 | 7.18 |
| F.KYYLQGAKIPKPEASFS(→G)P(→H)RRGWN.R | ITI HC4, isoform 1 | BCP(HP) | 4.24 | 4.78 |
| R.NVHSGSTFFKYYLQGAKI(-dehydration).P | ITI HC4, isoform 1 | BCP(HP) | 5.82 | 6.08 |
| N.RN(→D-methylthio)VHSGSTFFKYYLQGAKIPKPEASFSPR.R | ITI HC4, isoform 1 | BCP(HP) | 6.85 | 7.11 |
| K.YYLQGAKIPKPEASFS(→G)P(→H)RRGWN.R | ITI HC4, isoform 1 | BCP(HP) | 4.19 | 5.87 |
| R.N(→D)VHSGSTFFKYYLQGAKIPKPEASFS(→G)P(→H)RRGWN.R | ITI HC4, isoform 1 | BCP(HP) | 4.96 | 6.31 |
| F.KYYLQGAKIPKPEASFS(-acetylhexosamine)PR.R | ITI HC4, isoform 1 | BCP(HP) | 5.49 | 5.96 |
| R.KIQQTREALIKILDDLSPRDQFNLIVFSTEATQWRPSLVPASAENVNKA.R | ITI HC4, isoform 2 | BCP | 4.32 | 5.80 |
| K.GSEMVVAGKLQDR.G | ITI HC4, isoform 2 | BCP | 4.09 | 5.67 |
| R.HRQGPVNLLSDPEQGVEVTGQYEREKAGFSWIEVTFKNPLVWVHASPEHVVVTRNRR.S | ITI HC4, isoform 2 | BCP | 4.11 | 5.64 |
| R.RIHEDSDSALQLQDFYQEVANPLLTAVTFEYPSNAVEEVTQNNF.R | ITI HC4, isoform 2 | BCP | 4.35 | 6.46 |
| R.HRQGPVNLLSDPEQGVEVTGQYEREKAGFSWIEVTFKNPLVWVHASPEHVVVT.R | ITI HC4, isoform 2 | BCP | 4.33 | 6.08 |
| R.HRQGPVNLLSDPEQGVEVTGQYEREKAGFSWIEVTFKNPLVWVHASPEHVVVTR.N | ITI HC4, isoform 2 | BCP&HP | 5.71 | 6.30 |
| R.KIQQTREALIKILDDLSPRDQFNLIVFSTEATQWRPSLVPASAENVNKAR.S | ITI HC4, isoform 2 | BCP&HP | 5.86 | 6.46 |
| R.RIHEDSDSALQLQDFYQEVANPLLTAVTFEYPSNAVEEVTQNNFR.L | ITI HC4, isoform 2 | BCP&HP | 5.85 | 5.62 |
| S.SRQLGLPGPPDVPDHAAYHPF.R | ITI HC4, isoform 2 | BCP&HP | 6.28 | 6.40 |
| R.NVHSAGAAGSR.M | ITI HC4, isoform 2 | BCP&HP | 6.49 | 7.86 |
| G.PPDVPDHAAYHPF.R | ITI HC4, isoform 2 | BCP&HP | 7.15 | 7.26 |
| V.PDHAAYHPF.R | ITI HC4, isoform 2 | BCP&HP | 7.08 | 7.23 |
| P.PDVPDHAAYHPF.R | ITI HC4, isoform 2 | BCP&HP | 6.91 | 7.11 |
| P.GPPDVPDHAAYHPF.R | ITI HC4, isoform 2 | BCP&HP | 6.88 | 6.94 |
| R.QLGLPGPPDVPDHAAYHPF.R | ITI HC4, isoform 2 | BCP&HP | 7.89 | 8.18 |
| L.PGPPDVPDHAAYHPF.R | ITI HC4, isoform 2 | BCP&HP | 7.34 | 7.40 |
| L.PGPPDVPDHAAYHPFR.R | ITI HC4, isoform 2 | BCP&HP | 7.41 | 5.76 |
| R.HRQGPVNLLSDPEQGVEVTGQYEREKAGFSWIEVTFKNPLVWVHASPEHVVVTRN.R | ITI HC4, isoform 2 | BCP(HP) | 4.43 | 5.85 |
| A.EKNGIDIYSLTVDSRVSSRFAHTVVTSRVVN.R | ITI HC4, isoform 2 | BCP(HP) | 4.52 | 5.65 |
| R.HRQGPVNLLSDPEQGVEVTGQYEREKAGFSWIEVTFKNPLVWVHASPEHVVVTRNR.R | ITI HC4, isoform 2 | BCP(HP) | 4.79 | 6.15 |
| A.EKNGIDIYSLTVDSR.V | ITI HC4, isoform 2 | BCP(HP) | 5.20 | 6.18 |
| L.PGPPDVPDHAAYHP.F | ITI HC4, isoform 2 | BCP(HP) | 5.26 | 5.94 |
| G.PPDVPDHAAYHP.F | ITI HC4, isoform 2 | BCP(HP) | 5.04 | 5.76 |
| L.SSRQLGLPGPPDVPDHAAYHPFR.R | ITI HC4, isoform 2 | HP | 5.54 | 4.28 |
| P.DVPDHAAYHPFR.R | ITI HC4, isoform 2 | HP | 6.00 | 4.55 |
| D.VPDHAAYHPFR.R | ITI HC4, isoform 2 | HP | 6.11 | 4.41 |
| M.NFRPGVLSSR.L | ITI HC4, isoform 2 | HP | 6.36 | 4.68 |
| R.RIHEDSDSALQLQDFYQEVANPLLTAVTFEYPSNAVEEVTQNNFRLLFK.G | ITI HC4, isoform 2 | HP | 6.26 | 4.31 |
| L.GLPGPPDVPDHAAYHPFR.R | ITI HC4, isoform 2 | HP | 6.43 | 4.37 |
| P.GPPDVPDHAAYHPFR.R | ITI HC4, isoform 2 | HP | 6.84 | 5.31 |
| S.RQLGLPGPPDVPDHAAYHPFR.R | ITI HC4, isoform 2 | HP(BCP) | 5.96 | 4.98 |
| S.SRQLGLPGPPDVPDHAAYHPFR.R | ITI HC4, isoform 2 | HP(BCP) | 6.04 | 4.74 |
| L.PGPPDVPDHAA.Y | ITI HC4, isoform 2 | HP(BCP) | 5.60 | 5.93 |
| N.VHSAGAAGSR.M | ITI HC4, isoform 2 | HP(BCP) | 6.04 | 6.29 |
| D.VPDHAAYHPF.R | ITI HC4, isoform 2 | HP(BCP) | 5.97 | 5.57 |
| R.PGVLSSRQLGLPGPPDVPDHAAYHPF.R | ITI HC4, isoform 2 | HP(BCP) | 6.32 | 6.45 |
| S.RQLGLPGPPDVPDHAAYHPF.R | ITI HC4, isoform 2 | HP(BCP) | 6.48 | 5.30 |
| P.PDVPDHAAYHPFR.R | ITI HC4, isoform 2 | HP(BCP) | 6.59 | 5.08 |
| R.VQGNDHSATRER.R | ITI HC4, isoform 2 | HP(BCP) | 6.49 | 4.53 |
| G.PPDVPDHAAYHPFR.R | ITI HC4, isoform 2 | HP(BCP) | 7.00 | 5.65 |
| R.QLGLPGPPDVPDHAAYHPFR.R | ITI HC4, isoform 2 | HP(BCP) | 7.00 | 5.48 |
| V.PDHAAYHPFR.R | ITI HC4, isoform 2 | HP(BCP) | 6.95 | 5.40 |
| R.PGVLSSRQLGLPGPPDVPDHAAYHPFR.R | ITI HC4, isoform 2 | HP(BCP) | 6.08 | 5.26 |
| R.NVHSAGAAGSRM(-hydroxylation)NFRPGVLSSRQLGLPGPPDVPDHAAYHPFR.R | ITI HC4, isoform 2 | HP(BCP) | 6.93 | 5.28 |
| R.NVHSAGAAGSRM(-hydroxylation)NFRPGVLSSRQLGLPGPPDVPDHAAYHPF.R | ITI HC4, isoform 2 | HP(BCP) | 7.49 | 7.36 |
| R.MNFRPGVLSSRL(→Q)LGLPGPPDVPDHAAYHPF.R | ITI HC4, isoform 2 | BCP | 4.31 | 7.23 |
| R.E(→Q)ALIKILDDLSPRDQFNLIVFSTEATQWRPSLVPASAENVNKAR.S | ITI HC4, isoform 2 | BCP | 4.28 | 5.62 |
| R.PGVLSSRL(→Q)LGLPGPPDVPDHAAYHPF.R | ITI HC4, isoform 2 | BCP | 4.45 | 6.51 |
| H.S(-acetylation)AGAAGSRMNFRPGVLSSRQLGLPGPPDVPD(→N)HAAYHPF.R | ITI HC4, isoform 2 | BCP | 4.47 | 6.48 |
| R.L(→Q)LGLPGPPDVPDHAAYHPF.R | ITI HC4, isoform 2 | BCP&HP | 4.55 | 8.18 |
| R.NVHSAGAAGSRM(-dethiomethyl)NFRPGVLSS.R | ITI HC4, isoform 2 | BCP&HP | 6.11 | 6.08 |
| R.Q(-pyro-glu)LGLPGPPDVPDHAAYHPF.R | ITI HC4, isoform 2 | BCP&HP | 7.23 | 7.53 |
| R.M(-hydroxylation)NFRPGVLSSRQLGLPGPPDVPDHAAYHPF.R | ITI HC4, isoform 2 | BCP&HP | 7.38 | 7.04 |
| R.NVHSAGAAGSRMNFRPGVLSSRQLGLPGPPDVPDHAAYHPF.R | ITI HC4, isoform 2 | BCP(HP) | 4.28 | 6.71 |
| R.MNFRPGVLSSRQLGLPGPPDVPDHAAYHPF.R | ITI HC4, isoform 2 | BCP(HP) | 4.75 | 7.23 |
| A.GAAGSRM(-oxid)NFRPGVLSSRQLGLPGPPDVPDHAAYHPF.R | ITI HC4, isoform 2 | BCP(HP) | 6.18 | 6.70 |
| R.QLGLP(-hydroxylation)GPPDVPDHAAYHPF.R | ITI HC4, isoform 2 | BCP(HP) | 6.23 | 6.90 |
| R.NVHSAGAAGSRMNFR(-hydroxylation)PGVLSS.R | ITI HC4, isoform 2 | BCP(HP) | 6.78 | 6.67 |
| H.S(-acetylation)AGAAGSRM(-oxid)NFRPGVLSSRQLGLPGPPDVPD(→N)HAAYHPF.R | ITI HC4, isoform 2 | BCP(HP) | 7.45 | 7.20 |
| R.NVHSAGAAGSRMNFRPGVLSS.R | ITI HC4, isoform 2 | BCP(HP) | 4.45 | 6.15 |
| R.TTPKDFYVDENTTVR.V | Kallistatin | BCP(HP) | 5.11 | 5.30 |
| R.GHGLGHGHEQQHGLGHGHKFKLDDDLEHQGGHVLDHGHKH.K | Kininogen-1 (HMW) | BCP&HP | 6.56 | 6.80 |
| R.GHGLGHGHEQQHGLGHGHKF.K | Kininogen-1 (HMW) | BCP&HP | 6.54 | 6.20 |
| R.RHDWGHEKQ.R | Kininogen-1 (HMW) | BCP&HP | 7.15 | 7.11 |
| R.GHGLGHGHEQQHGLGHGHKFKLDDDLEHQGGHVLDHGHKHKHGHGHGKHKNKG.K | Kininogen-1 (HMW) | BCP(HP) | 6.38 | 6.64 |
| R.RHDWGHEKQR.K | Kininogen-1 (HMW) | HP | 6.18 | 4.07 |
| R.KHNLGHGHKHERDQGHGHQR.G | Kininogen-1 (HMW) | HP | 6.46 | 4.70 |
| R.GHGLGHGHEQQHGLGHGHKFKLDDDLEHQGGHVLDHGHK.H | Kininogen-1 (HMW) | HP(BCP) | 5.57 | 5.49 |
| R.IGEIKEETTSHL.R | Kininogen-1 (LMW) | BCP&HP | 5.26 | 5.96 |
| R.IGEIKEETTSHLR.S | Kininogen-1 (LMW) | BCP&HP | 5.82 | 5.30 |
| L.PQPDLRYLFLNGNKLAR.V | Leucine-rich α-2-glycoprotein | BCP | 4.18 | 5.46 |
| K.ALGHLDLSGNRL.R | Leucine-rich α-2-glycoprotein | BCP | 4.26 | 5.20 |
| R.GPLQLERLHLEGNKLQVLGKDLLLPQPDLRYLFLNGNKLA.R | Leucine-rich α-2-glycoprotein | BCP&HP | 5.43 | 6.23 |
| R.GPLQLERLHLEGNKLQVLGKDLLLPQPDLR.Y | Leucine-rich α-2-glycoprotein | BCP&HP | 5.76 | 6.32 |
| R.GPLQLERLHLEGNKLQVLGKDLLLPQPDLRYLFLNGNKLAR.V | Leucine-rich α-2-glycoprotein | BCP&HP | 6.00 | 6.36 |
| R.TLDLGENQLETLPPDLLR.G | Leucine-rich α-2-glycoprotein | BCP&HP | 6.43 | 6.38 |
| R.GPLQLERLHLEGNKLQVLGKDLLLPQPDL.R | Leucine-rich α-2-glycoprotein | BCP(HP) | 4.54 | 6.18 |
| R.GPLQLERLHLEGNKLQVLGKDLLLPQPDLRYLFLNGNKLAR (aminoethylbenzenesulfonylation on H or K).V | Leucine-rich α-2-glycoprotein | BCP | 4.10 | 5.54 |
| K.SFRPFVPR.L | Lipopolysaccharide-binding protein | BCP | 4.03 | 5.53 |
| A.KETLQKLPEEIQRDILLEKKKVAQDQL.R | Mannosidase, α, class 1A, member 1 | BCP | 4.26 | 5.79 |
| R.EAKETLQKLPEEIQRDILLEKKKVAQDQLRDKAPF.R | Mannosidase, α, class 1A, member 1 | BCP | 4.34 | 6.36 |
| R.EAKETLQKLPEEIQRDILLEKKKVAQDQL.R | Mannosidase, α, class 1A, member 1 | BCP | 4.34 | 5.68 |
| R.EAKETLQKLPEEIQRDILLEKKKVAQDQLR.D | Mannosidase, α, class 1A, member 1 | BCP&HP | 6.30 | 6.26 |
| A.KETLQKLPEEIQRDILLEKKKVAQDQLR.D | Mannosidase, α, class 1A, member 1 | BCP&HP | 6.43 | 6.30 |
| A.KETLQKLPEEIQRDILLEKKKVAQDQLRDKAPF.R | Mannosidase, α, class 1A, member 1 | BCP(HP) | 6.30 | 6.63 |
| R.EAKETLQKLPEEIQRDILLEKKKVAQDQLRDKAPFR.G | Mannosidase, α, class 1A, member 1 | HP(BCP) | 6.11 | 5.49 |
| A.KETLQKLPEEIQRDILLEKKKVAQDQLRDKAPFR.G | Mannosidase, α, class 1A, member 1 | HP(BCP) | 6.38 | 5.68 |
| R.SMQRYHQDTQGWGDIGYSFVVGSDGYVYEGRGWHWVGAHTLGHNS.R | N-acetylmuramoyl-L-alanine amidase | BCP | 4.27 | 5.61 |
| R.RVINLPLDSMAAPWETGDTFPDVVAIAPDVRATSSPGL.R | N-acetylmuramoyl-L-alanine amidase | BCP | 4.56 | 6.28 |
| R.SMQRYHQDTQGWGDIGYSFVVGSDGYVYEGRGWHWVGAHTLGHNSR.G | N-acetylmuramoyl-L-alanine amidase | BCP&HP | 5.30 | 6.08 |
| R.TWPHFTATVKPRPA.R | N-acetylmuramoyl-L-alanine amidase | BCP&HP | 5.81 | 6.32 |
| R.EGKEYGVVLAPDGSTVAVEPLLAGLEAGLQGR.R | N-acetylmuramoyl-L-alanine amidase | BCP&HP | 6.28 | 6.32 |
| R.TPEPRPSLSHLLSQYYGAGVARDPGF.R | N-acetylmuramoyl-L-alanine amidase | BCP(HP) | 5.46 | 6.64 |
| A.PWETGDTFPDVVAIAPDVR.A | N-acetylmuramoyl-L-alanine amidase | HP(BCP) | 5.61 | 5.23 |
| R.TPEPRPSLSHLLSQYYGAGVARDPGFR.S | N-acetylmuramoyl-L-alanine amidase | BCP&HP | 5.54 | 5.53 |
| R.HDVREGKEYGVVLAPDGSTVAVEPLLAGLEAGLQGR.R | N-acetylmuramoyl-L-alanine amidase | BCP(HP) | 5.60 | 5.96 |
| R.SM(-oxid)QRYHQDTQGWGDIGYSFVVGSDGYVYEGRGWHWVGAHTLGHNSR.G | N-acetylmuramoyl-L-alanine amidase | BCP(HP) | 5.72 | 5.84 |
| R.TVRDTLPSC(-dehydro)AVRAGLLRPDYALLGHRQLVRTDC(-dehydro)PGDALFDLLRTWPHFTATVKPRPA.R | N-acetylmuramoyl-L-alanine amidase | BCP(HP) | 6.18 | 5.91 |
| R.RVINLPLDSM(-hydroxylation)AAPWETGDTFPDVVAIAPDVR.A | N-acetylmuramoyl-L-alanine amidase | HP | 6.66 | 6.02 |
| R.GLFIIDGKGVLRQITVNDLPVGR.S | Peroxiredoxin-2 | BCP(HP) | 4.76 | 5.56 |
| R.SSLGARLSGALHVYSLGSD.- | Phosphatidylinositol-glycan-specific phospholipase D | BCP(HP) | 4.97 | 6.04 |
| S.PPEEGSPDPDSTGALVEEEDPFFKVPVNKLAAAVSNFGYDL.Y | Pigment epithelium-derived factor | BCP | 4.32 | 6.28 |
| K.TVQAVLTVPKLKLSYEGEVTKSLQEMKLQSLFDSPDFSKITGKPIKLTQVEH.R | Pigment epithelium-derived factor | BCP | 4.23 | 6.18 |
| S.PPEEGSPDPDSTGALVEEEDPFFKVPVNKLAAAVSNFGYDLY.R | Pigment epithelium-derived factor | BCP | 4.17 | 6.04 |
| K.LKLSYEGEVTKSLQEMKLQSLFDSPDFSKITGKPIKLTQVEHR.A | Pigment epithelium-derived factor | BCP | 4.22 | 5.92 |
| R.IVFEKKLR.I | Pigment epithelium-derived factor | BCP | 4.09 | 5.87 |
| K.LKLSYEGEVTKSLQEMKLQSLFDSPDFSKITGKPIKLTQVEH.R | Pigment epithelium-derived factor | BCP | 4.31 | 5.85 |
| R.ALYYDLISSPDIHGTYKELLDTVTAPQKNL.K | Pigment epithelium-derived factor | BCP | 4.47 | 6.20 |
| K.SLQEMKLQSLFDSPDFSKITGKPIKLTQVEH.R | Pigment epithelium-derived factor | BCP | 4.38 | 6.11 |
| Y.DLISSPDIHGTYKELLDTVTAPQKNLK.S | Pigment epithelium-derived factor | BCP | 4.38 | 5.91 |
| I.SSPDIHGTYKELLDTVTAPQKNLK.S | Pigment epithelium-derived factor | BCP | 4.50 | 5.88 |
| I.SSPDIHGTYKELLDTVTAPQKNLKSASR.I | Pigment epithelium-derived factor | BCP | 4.37 | 5.79 |
| L.YYDLISSPDIHGTYKELLDTVTAPQKNLK.S | Pigment epithelium-derived factor | BCP | 4.33 | 5.67 |
| R.ALYYDLISSPDIHGTYKELLDTVTAPQK.N | Pigment epithelium-derived factor | BCP | 4.35 | 5.41 |
| R.TESIIHRALYYDLISSPDIHGTYKELLDTVTAPQKNLK.S | Pigment epithelium-derived factor | BCP&HP | 5.64 | 5.91 |
| R.VLTGNPRLDLQEINNWVQAQMKGK.L | Pigment epithelium-derived factor | BCP&HP | 5.41 | 5.45 |
| R.IKSSFVAPLEKSYGTRPRVLTGNPRLDLQEINNWVQAQMKGKLAR.S | Pigment epithelium-derived factor | BCP&HP | 5.79 | 5.92 |
| R.VLTGNPRLDLQEINNWVQAQMKGKLA.R | Pigment epithelium-derived factor | BCP&HP | 6.08 | 7.11 |
| R.ALYYDLISSPDIHGTYKELLDTVTAPQKNLK.S | Pigment epithelium-derived factor | BCP&HP | 6.11 | 6.79 |
| S.PPEEGSPDPDSTGALVEEEDPFFKVPVNKLAAAVSNFGYDLYR.V | Pigment epithelium-derived factor | BCP&HP | 6.18 | 6.68 |
| K.TVQAVLTVPKLKLSYEGEVTKSLQEMKLQSLFDSPDFSKITGKPIKLTQVEHR.A | Pigment epithelium-derived factor | BCP&HP | 6.04 | 6.40 |
| R.IVFEKKLRIKSSFVAPLEKSYGTRPR.V | Pigment epithelium-derived factor | BCP&HP | 6.11 | 6.08 |
| R.STKEIPDEISILLLGVAHFKGQWVTKFDSR.K | Pigment epithelium-derived factor | BCP&HP | 6.00 | 5.88 |
| R.STKEIPDEISILLLGVAHFKGQWVTKFDSRKTSLEDFYLDEER.T | Pigment epithelium-derived factor | BCP&HP | 6.30 | 6.73 |
| R.VLTGNPRLDLQEINNWVQAQMKGKLAR.S | Pigment epithelium-derived factor | BCP&HP | 6.34 | 6.51 |
| S.PPEEGSPDPDSTGALVEEEDPFFKVPVNKLAAAVSNFGYDLYRVR.S | Pigment epithelium-derived factor | BCP&HP | 6.20 | 6.30 |
| R.ALYYDLISSPDIHGTYKELLDTVTAPQKNLKSASR.I | Pigment epithelium-derived factor | BCP&HP | 5.74 | 4.29 |
| K.SLQEMKLQSLFDSPDFSKITGKPIKLTQVEHR.A | Pigment epithelium-derived factor | BCP(HP) | 4.99 | 6.26 |
| Y.YDLISSPDIHGTYKELLDTVTAPQKNLK.S | Pigment epithelium-derived factor | BCP(HP) | 4.83 | 5.95 |
| L.ISSPDIHGTYKELLDTVTAPQKNLK.S | Pigment epithelium-derived factor | BCP(HP) | 4.85 | 5.77 |
| V.APLEKSYGTRPR.V | Pigment epithelium-derived factor | BCP(HP) | 5.69 | 6.20 |
| A.PLEKSYGTRPR.V | Pigment epithelium-derived factor | BCP(HP) | 5.88 | 6.34 |
| K.SASRIVFEKKLRIKSSFVAPLEKSYGTRPR.V | Pigment epithelium-derived factor | BCP(HP) | 5.76 | 5.86 |
| R.IKSSFVAPLEKSYGTRPR.V | Pigment epithelium-derived factor | BCP(HP) | 6.08 | 6.92 |
| K.SSFVAPLEKSYGTRPR.V | Pigment epithelium-derived factor | BCP(HP) | 6.23 | 6.63 |
| R.IVFEKKLRIKSSFVAPLEKSYGTRPRVLTGNPRLDLQEINNWVQAQMKGKLAR.S | Pigment epithelium-derived factor | HP(BCP) | 5.45 | 5.38 |
| R.TESIIHRALYYDLISSPDIHGTYKELLDTVTAPQKNLKSASR.I | Pigment epithelium-derived factor | HP(BCP) | 5.26 | 5.36 |
| R.ALYYDLISSPDIHGTYKELLDTVTAPQKNLKSASRIVFEKKLR.I | Pigment epithelium-derived factor | HP(BCP) | 5.58 | 5.87 |
| V.LTGNPRLDLQEINNWVQAQMKGKLAR.S | Pigment epithelium-derived factor | HP(BCP) | 5.48 | 5.35 |
| L.TGNPRLDLQEINNWVQAQMKGKLAR.S | Pigment epithelium-derived factor | HP(BCP) | 5.91 | 5.49 |
| R.STKEIPDEISILLLGVAHFKGQWVTKFDSRKTSLEDFYLDEERTVR.V | Pigment epithelium-derived factor | HP(BCP) | 6.66 | 6.96 |
| C.Q(-pyro-glu)NPASPPEEGSPDPDSTGALVEEEDPFFKVPVNKLAAAVSNFGYDLYRVR.S | Pigment epithelium-derived factor | HP(BCP) | 7.18 | 7.15 |
| R.TESIIHRALYYDLISSPDIHGTYKELLDTVTAPQKNLKSASRIVFEKKLRIKSSFVAPLEKSYGTRPR.V | Pigment epithelium-derived factor | BCP(HP) | 5.28 | 5.57 |
| R.ALYYDLISSPDIHGTYKELLDTVTAPQKNLKSASRIVFEKKLRIKSSFVAPLEKSYGTRPR.V | Pigment epithelium-derived factor | BCP(HP) | 5.93 | 6.18 |
| R.VLTGNPRLDLQEINNWVQAQM(-hydroxylation)KGKLA.R | Pigment epithelium-derived factor | BCP(HP) | 5.76 | 6.15 |
| K.SLQEM(-hydroxylation)KLQSLFDSPDFSKITGKPIKLTQVEHR.A | Pigment epithelium-derived factor | BCP(HP) | 5.86 | 5.61 |
| R.TLLVFEVQQPFLFVLWDQQHKFPVFMGRVYDPRA.- | Plasma protease C1 inhibitor | BCP | 4.64 | 7.46 |
| D.PDLQVSAMQHQTVLELTETGVEAAAASAISVAR.T | Plasma protease C1 inhibitor | HP(BCP) | 5.40 | 5.66 |
| D.PDLQVSAM(-hydroxylation)QHQTVLELTETGVEAAAASAISVAR.T | Plasma protease C1 inhibitor | HP(BCP) | 5.95 | 5.03 |
| R.LNSQRLVFNRPFLMFIVDNNILFLGKVNRP.- | Plasma serine protease inhibitor | BCP&HP | 6.08 | 6.26 |
| R.SARLNSQRLVFNRPFLMFIVDNNILFLGKVNRP.- | Plasma serine protease inhibitor | BCP&HP | 7.32 | 7.30 |
| R.SARLNSQRLVFNRPFLM(-hydroxylation)FIVDNN][ILFLGKVNRP.- | Plasma serine protease inhibitor | HP(BCP) | 6.00 | 6.10 |
| Q.GASLFSVTKKQLGAGSIEEC(−dehydro)AAKC(−dehydro)EEDEEFTC(−dehydro)RAFQYHSKEQQC(−dehydro)VIMAENRKSSIIIR.M | Plasminogen | BCP | 4.25 | 5.66 |
| G.EPLDDYVNTQGASLFSVTKKQLGAGSIEEC(-dehydro)AAKC(-dehydro)EEDEEFTC(-dehydro)RAFQYHSKEQQC(-dehydro)VIM(-hydroxylation)AENR.K | Plasminogen | HP(BCP) | 6.18 | 5.67 |
| G.EPLDDYVNTQGASLFSVTKKQLGAGSIEEC(-dehydro)AAKC(-dehydro)EEDEEFTC(-dehydro)RAFQYHSKEQQC(-dehydro)VIM(-hydroxylation)AENRK.S | Plasminogen | HP(BCP) | 6.26 | 5.34 |
| G.EPLDDYVNTQGASLFSVTKKQLGAGSIEEC(-dehydro)AAKC(-dehydro)EEDEEFTC(-dehydro)RAFQYHSKEQQC(-dehydro)VIMAENRKSSIIIRMR.D | Plasminogen | BCP | 4.32 | 5.92 |
| G.EPLDDYVNTQGASLFSVTKKQLGAGSIEEC(-dehydro)AAKC(-dehydro)EEDEEFTC(-dehydro)RAFQYHSKEQQC(-dehydro)VIMAEN.R | Plasminogen | BCP | 4.29 | 5.58 |
| G.EPLDDYVNTQGASLFSVTKKQLGAGSIEEC(-dehydro)AAKC(-dehydro)EEDEEFTC(-dehydro)RAFQYHSKEQQC(-dehydro)VIMAENRKSSIIIRM.R | Plasminogen | BCP | 4.23 | 5.40 |
| P.LDDYVNTQGASLFSVTKKQLGAGSIEEC(-dehydro)AAKC(-dehydro)EEDEEFTC(-dehydro)RAFQYHSKEQQC(-dehydro)VIMAENRKSSIII.R | Plasminogen | BCP | 4.40 | 6.04 |
| G.EP(-hydroxylation)LDDYVNTQGASLFSVTKKQLGAGSIEEC(-dehydro)AAKC(-dehydro)EEDEEFTC(-dehydro)RAFQYHSKEQQC(-dehydro)VIMAENRKSSIIIRM.R | Plasminogen | BCP | 4.40 | 5.85 |
| G.EPLDDYVNTQGASLFSVTKKQLGAGSIEEC(-dehydro)AAKC(-dehydro)EEDEEFTC(-dehydro)RAFQYHSKEQQC(-dehydro)VIMAENRKSSIIIR.M | Plasminogen | BCP&HP | 5.73 | 6.62 |
| G.EPLDDYVNTQGASLFSVTKKQLGAGSIEEC(-dehydro)AAKC(-dehydro)EEDEEFTC(-dehydro)RAFQYHSKEQQC(-dehydro)VIMAENRK.S | Plasminogen | BCP(HP) | 5.82 | 6.41 |
| G.EPLDDYVNTQGASLFSVTKKQLGAGSIEEC(-dehydro)AAKC(-dehydro)EEDEEFTC(-dehydro)RAFQYHSKEQQC(-dehydro)VIM(-hydroxylation)AENRKSSIIIRMR.D | Plasminogen | BCP(HP) | 5.41 | 6.15 |
| G.EPLDDYVNTQGASLFSVTKKQLGAGSIEEC(-dehydro)AAKC(-dehydro)EEDEEFTC(-dehydro)RAFQYHSKEQQC(-dehydro)VIM(-hydroxylation)AENRKSSIIIR.M | Plasminogen | HP | 6.77 | 6.59 |
| R.IKKIVQKKLAGDESAD.- | Platelet basic protein | BCP | 4.38 | 5.91 |
| R.LWVMPNHQVLLGPEEDQDHIYHPQ.- | Proline-rich acidic protein 1 | BCP | 4.51 | 6.18 |
| A.APEAQVSVQPNFQQDKFLGR.W | Prostaglandin-H2 D-isomerase | HP(BCP) | 5.28 | 4.89 |
| R.IVEGSDAEIGMSPWQVMLFR.K | Prothrombin | BCP | 4.30 | 6.20 |
| R.YERNIEKISMLEKIYIHPR.Y | Prothrombin | BCP | 4.42 | 6.00 |
| R.IGKHSRTRYERNIEKISMLEKIYIHPR.Y | Prothrombin | BCP&HP | 5.81 | 6.51 |
| A.TSEYQTFFNPR.T | Prothrombin | BCP&HP | 6.15 | 6.40 |
| R.TATSEYQTFFNPR.T | Prothrombin | BCP&HP | 6.26 | 7.32 |
| T.SEYQTFFNPR.T | Prothrombin | BCP&HP | 6.49 | 6.82 |
| Q.DFNSAVQLVENFC(−dehydro)RNPDGDEEGVWC(−dehydro)YVAGKPGDFGYC(−dehydro)DLNYC(−dehydro)EEAVEEETGDGLDEDSDRAIEGR.T | Prothrombin | BCP(HP) | 4.41 | 5.22 |
| R.TRYERNIEKISMLEKIYIHPR.Y | Prothrombin | BCP(HP) | 4.88 | 6.78 |
| R.NIEKISMLEKIYIHPR.Y | Prothrombin | BCP(HP) | 5.25 | 6.04 |
| K.SLEDKTERELLESYIDGR.I | Prothrombin | HP(BCP) | 5.60 | 5.72 |
| R.TAT(-dehydration)SEYQTFFNPR.T | Prothrombin | HP(BCP) | 6.20 | 6.43 |
| R.SEGSSVNLSPPLEQC(−dehydro)VPDRGQQYQGRLAVTTHGLPC(−dehydro)LAWASAQAKALSKHQDFNSAVQLVENFC(−dehydro)RNPDGDEEGVWC(−dehydro)YVAGKPGDFGYC(−dehydro)DLNYC(-dehydro)EEAVEEETGDGLDEDSDRAIEGR.T | Prothrombin | HP(BCP) | 5.99 | 6.20 |
| R.SEGSSVNLSPPLEQC(−dehydro)VPDRGQQYQGRLAVTTHGLPC(−dehydro)LAWASAQAKALSKHQDFNSAVQLVENFC(−dehydro)RNPDGDEEGVWC(−dehydro)YVAGKPGDFGYC(−dehydro)DLNYC(−dehydro)EEAVEEETGDGLDEDSDRAIEGR.T (hydroxylationized) | Prothrombin | HP(BCP) | 5.99 | 6.00 |
| R.SEGSSVNLSPPLEQC(−dehydro)VPDRGQQYQGRLAVTTHGLPC(−dehydro)LAWASAQAKALSKHQDFNSAVQLVENFC(−dehydro)RNPDGDEEGVWC(−dehydro)YVAGKPGDFGYC(−dehydro)DLNYC(−dehydro)EEAVEEETGDGLDEDSDR.A | Prothrombin | BCP&HP | 7.62 | 8.04 |
| R.YNWRENLDRDIALMKLKKPVAFSDYIHPVC(-dehydro)LPDR.E;T.FFNPRTFGSGEADC(-dehydro)GLRPLFEKKSLEDKTERELLESYIDGRIVEGSDAE.I | Prothrombin | BCP(HP) | 5.75 | 5.86 |
| R.TRYERNIEKISM(-hydroxylation)LEKIYIHPR.Y | Prothrombin | BCP(HP) | 6.26 | 6.75 |
| R.SEGSSVNLSPPLEQC(−dehydro)VPDRGQQYQGRLAVTTHGLPC(−dehydro)LAWASAQAKALSKHQDFNSAVQLVENFC(−dehydro)RNPDGDEEGVWC(−dehydro)YVAGKPGDFGYC(−dehydro)DLNYC(-dehydro)EEAVEEETGDGLDEDSDRAIEGR.T_hydroxylation | Prothrombin | BCP(HP) | 6.34 | 6.48 |
| M.SIPPEVKFNKPFVFLMIEQNTKSPLFMGKVVNPTQK.- | Serine proteinase inhibitor, clade A, member 1 | BCP(HP) | 5.26 | 6.52 |
| I.PPEVKFNKPFVFLMIEQ.N | Serine proteinase inhibitor, clade A, member 1 | BCP(HP) | 4.43 | 3.70 |
| R.VTFKANRPFLVFIREVPLNTIIFMGR.V | Serpin peptidase inhibitor, clade C, member 1 | BCP | 4.36 | 6.20 |
| K.ANRPFLVFIREVPLNTIIFMGR.V | Serpin peptidase inhibitor, clade C, member 1 | BCP&HP | 5.83 | 6.88 |
| R.SLNPNRVTFKANRPFLVFIREVPLNTIIFMGR.V | Serpin peptidase inhibitor, clade C, member 1 | BCP&HP | 7.43 | 8.00 |
| L.NPNRVTFKANRPFLVFIREVPLNTIIFMGR.V | Serpin peptidase inhibitor, clade C, member 1 | BCP(HP) | 5.69 | 6.26 |
| N.PNRVTFKANRPFLVFIREVPLNTIIFMGR.V | Serpin peptidase inhibitor, clade C, member 1 | BCP(HP) | 5.70 | 6.11 |
| R.SLNPNRVTFKANRPFLVFIREVPLNTIIFMG.R | Serpin peptidase inhibitor, clade C, member 1 | BCP | 4.69 | 6.90 |
| R.EVLLPKFKLEKNYNLVESLKLMGIR.M | Serpin peptidase inhibitor, clade D | BCP | 4.26 | 5.40 |
| K.SRIQRLNILNAKFAFNLYR.V | Serpin peptidase inhibitor, clade D | BCP(HP) | 4.85 | 5.26 |
| R.SVNDLYIQKQFPILLDFKTKVREYYFAEAQIADFSDPAFISK.T | Serpin peptidase inhibitor, clade D | HP | 5.79 | 4.37 |
| K.FKLEKNYNLVESLKLMGIR.M | Serpin peptidase inhibitor, clade D | HP(BCP) | 5.53 | 5.32 |
| K.SFNPNSPGKILLMDLNEEDPTVLELGITGSKFDVSSFNPH.G | Serum paraoxonase/arylesterase 1 | BCP | 4.30 | 5.64 |
| R.SEETKENEGFTVTAEGKGQGTLSVVTMYHAK.A | Similar to C3 | BCP | 4.15 | 5.49 |
| R.SEETKENEGFTVTAEGK.G | Similar to C3 | BCP | 4.11 | 4.30 |
| R.SSKITHRIHWESAS.L | Similar to C3 | BCP&HP | 5.81 | 6.00 |
| S.SKITHRIHWESASLL.R | Similar to C3 | BCP&HP | 6.08 | 6.04 |
| R.SSKITHRIHWESASLLR.S | Similar to C3 | BCP&HP | 6.36 | 5.54 |
| R.SSKITHRIHWESASLL.R | Similar to C3 | BCP&HP | 6.80 | 6.32 |
| S.SKITHRIHWESAS.L | Similar to C3 | HP | 4.91 | 4.23 |
| R.IHWESASLLR.S | Similar to C3 | HP | 5.30 | 4.20 |
| T.HRIHWESASLLR.S | Similar to C3 | HP | 5.52 | 4.30 |
| K.ITHRIHWESASLLR.S | Similar to C3 | HP | 6.00 | 4.34 |
| I.THRIHWESASLLR.S | Similar to C3 | HP(BCP) | 6.04 | 5.00 |
| R.SSKITHRIHWESASL.L | Similar to C3 | HP(BCP) | 5.41 | 5.32 |
| R.AFAGPSQKPETIELR.T | Thrombospondin-4 | BCP | 3.84 | 5.04 |
| K.PDMAEIEKFDKSKLKKTETQEKNPLPSKETIEQEKQAGES.- | Thymosin β-4 | BCP | 4.23 | 6.08 |
| K.SKLKKTETQEKNPLPSKETIEQEKQAGES.- | Thymosin β-4 | HP(BCP) | 5.86 | 5.56 |
| M.S(−acetylation)DKPDM(−hydroxylation)AEIEKFDKSKLKKTETQEKNPLPSKETIEQEKQAGES.- | Thymosin β-4 | BCP&HP | 7.97 | 7.45 |
| M.S(-acetylation)DKPDMAEIEKFDKSKLKKTETQEKNPLPSKETIEQEKQAGES.- | Thymosin β-4 | BCP(HP) | 4.94 | 7.75 |
| L.PSKETIEQEKQAGES.- | Thymosin-like 3 | BCP | 4.37 | 5.71 |
| K.KTETQEKNPLPSKETIEQEKQAGES.- | Thymosin-like 3 | BCP&HP | 5.88 | 7.36 |
| N.PLPSKETIEQEKQAGES.- | Thymosin-like 3 | HP(BCP) | 5.76 | 5.11 |
| K.AVLHIGEKGTEAAAVPEVELSDQPENTFLHPIIQIDRSFMLLILERSTRSILFLGKVVNPTEA.- | Thyroxine-binding globulin | BCP | 4.44 | 6.26 |
| Y.SYSTTAVVTNPKE.- | Transthyretin | BCP | 4.23 | 5.41 |
| R.RYTIAALLSPYSYSTTAVVTNPKE.- | Transthyretin | BCP | 4.42 | 5.98 |
| R.KAADDTWEPFASGKTSESGELHGLTTEEEFVEGIYKVEIDTKSYWK.A | Transthyretin | BCP&HP | 5.69 | 5.70 |
| K.SYWKALGISPFHEHAEVVFTANDSGPRRYTIAALLSPYSYSTTAVVTNPKE.- | Transthyretin | BCP&HP | 5.88 | 5.96 |
| K.ALGISPFHEHAEVVFTANDSGPRRYTIAALLSPYSYSTTAVVTNPKE.- | Transthyretin | BCP&HP | 6.85 | 6.83 |
| R.KAADDTWEPFASGK.T | Transthyretin | BCP(HP) | 4.78 | 5.36 |
| Y.STTAVVTNPKE.- | Transthyretin | BCP(HP) | 5.57 | 7.20 |
| R.KAADDTWEPFASGKTSESGELHGLTTEEEFVEGIYKVEIDTK.S | Transthyretin | BCP(HP) | 5.46 | 6.11 |
| A.GPTGTGESKC(-cystylation)PLM(-hydroxylation)VKVLDAVRGSPAINVAVHVFR.K | Transthyretin | HP(BCP) | 7.11 | 6.53 |
| A.GPTGTGESKC(−cysteinylation)PLMVKVLDAVRGSPAINVAVHVFR→T)KAADDTWEPFASGKTSESGELHGLTTEEEFVEGIYKVEIDTKSYWKALGISPFHEHAEVVFTANDSGPRRYTIAALLSPYSYSTTAVVTNPKE.- | Transthyretin | HP(BCP) | 7.11 | 6.34 |
| A.GPTGTGESKC(-cystylation)PLMVKVLDAVRGSPAINVAVHVFR.K | Transthyretin | BCP&HP | 6.41 | 6.87 |
| A.GPTGTGESKC(−cysteinylation)PLMVKVLDAVRGSPAINVAVHVFRKAADDTWEPFASGKTSESGELHGLTTEEEFVEGIYKVEIDTKSYWKALGISPFHEHAEVVFTANDSGPRRYTIAALLSPYSYSTTAVVTNPKE.- | Transthyretin | BCP&HP | 8.26 | 8.48 |
| A.GPTGTG(→S)ES(→N)KC(−cysteinylation)PLMVKVLDAVRGSPAINVAVHVFRKAADDTWEPFASGKTSESGELHGLTTEEEFVEGIYKVEIDTKSYWKALGISPFHEHAEVVFTANDSGPRRYTIAALLSPYSYSTTAVVTNPKE.- | Transthyretin | BCP&HP | 7.57 | 7.64 |
| A.GPTGTGESKCPLMVKVLDAVRGSPAINVAVHVFRKAADDTWEPFASGKTSESGELHGLTTEEEFVEGIYKVEIDTKSYWKALGISPFHEHAEVVFTANDSGPRRYTIAALLSPYSYSTTAVVTNPKE.- | Transthyretin | BCP&HP | 7.59 | 7.57 |
| A.GPTGTGESKC(−cysteinylation)PLMVKVLDAVRGSPAINVAVHVFRKAADDTWEPFASGKTSESGELHGLTTEEEFVEGIYKVEIDTKSYWKALGISPFHEHAEVVFTANDSGPRRYTIAALLSPYSYSTTAVVTNPKE(-hydroxylation).- | Transthyretin | BCP&HP | 7.98 | 6.38 |
| TTR unresolved form 2 | Transthyretin | BCP(HP) | 6.90 | 7.01 |
| G.PTGTGESKCPLMVKVLDAVRGSPAINVAVHVFRKAADDTWEPFASGKTSESGELHGLTTEEEFVEGIYKVEIDTKSYWKALGISPFHEHAEVVFTANDSGPRRYTIAALLSPYSYSTTAVVTNPKE.-(unknown mod) | Transthyretin | BCP(HP) | 7.29 | 7.37 |
| TTR unresolved form 1 | Transthyretin | BCP(HP) | 7.93 | 6.65 |
| M(−hydroxylation)QIFVKTLTGKTITLEVEPSDTIENVKAKIQDKEGIPPDQQRLIFAGKQLEDGRTLSDYNIQKESTLHLVLRLRGG | Ubiquitin and ribosomal protein S27a | HP(BCP) | 5.93 | 4.80 |
| −.MQIFVKTLTGKTITLEVEPSDTIENVKAKIQDKEGIPPDQQRLIFAGKQLEDGRTLSDYNIQKESTLHLVLRLRGG.M | Ubiquitin and ribosomal protein S27a | BCP&HP | 6.48 | 6.52 |
| R.THLPEVFLSKVLEPTLK.S | Vitamin D-binding protein | BCP&HP | 5.64 | 6.04 |
| R.RTHLPEVFLSKVLEPTLK.S | Vitamin D-binding protein | BCP(HP) | 5.67 | 5.95 |
| M.APRPSLAKKQRF.R | Vitronectin | BCP | 4.08 | 6.46 |
| R.IYISGMAPRPSLAK.K | Vitronectin | BCP | 4.26 | 5.48 |
| R.IYISGMAPRPSLAKKQR.F | Vitronectin | BCP | 4.15 | 5.11 |
| R.IYISGMAPRPSLAKKQRF.R | Vitronectin | BCP&HP | 5.28 | 6.04 |
| S.GMAPRPSLAKKQRF.R | Vitronectin | BCP&HP | 5.54 | 6.20 |
| R.QPQFISRDWHGVPGQVDAAMAGR.I | Vitronectin | BCP&HP | 5.94 | 6.11 |
| R.TSAGTRQPQFISRDWHGVPGQVDAAMAGRIYISGMAPRPSLAKKQRF.R | Vitronectin | BCP&HP | 6.08 | 6.78 |
| I.YISGMAPRPSLAKKQRF.R | Vitronectin | BCP&HP | 5.99 | 6.70 |
| R.DWHGVPGQVDAAMAGR.I | Vitronectin | BCP&HP | 6.04 | 6.40 |
| V.PGQVDAAMAGRIYISGMAPRPSLAKKQRFR.H | Vitronectin | BCP&HP | 6.11 | 6.00 |
| R.TSAGTRQPQFISR.D | Vitronectin | BCP&HP | 6.23 | 7.08 |
| I.SGMAPRPSLAKKQRF.R | Vitronectin | BCP&HP | 6.20 | 6.87 |
| Y.ISGMAPRPSLAKKQRF.R | Vitronectin | BCP&HP | 6.20 | 6.78 |
| R.PSLAKKQRFR.H | Vitronectin | BCP&HP | 6.23 | 6.04 |
| V.PGQVDAAMAGR.I | Vitronectin | BCP&HP | 6.48 | 6.49 |
| R.TSAGTRQPQFISRDWHGVPGQVDAAMAG.R | Vitronectin | BCP&HP | 6.76 | 7.38 |
| R.TSAGTRQPQFISRDWHGVPGQVDAAMAGRIYISGMAPRPSLAKKQRFR.H | Vitronectin | BCP&HP | 6.90 | 6.98 |
| R.TSAGTRQPQFISRDWHGVPGQVDAAMAGR.I | Vitronectin | BCP&HP | 7.23 | 7.36 |
| R.IYISGMAPRPSLAKKQRFR.H | Vitronectin | BCP&HP | 7.20 | 7.20 |
| M.APRPSLAKKQRFR.H | Vitronectin | BCP&HP | 5.60 | 5.89 |
| R.DWHGVPGQVDAAMAGRIYISGMAPRPSLAKKQRF.R | Vitronectin | BCP(HP) | 4.85 | 6.28 |
| R.TSAGTRQPQFISRDWHGVPGQVDAAMAGRIYISGMAPRPSLAKKQR.F | Vitronectin | BCP(HP) | 5.14 | 5.78 |
| V.PGQVDAAMAGRIYISGMAPRPSLAKKQRF.R | Vitronectin | BCP(HP) | 5.04 | 5.51 |
| A.PRPSLAKKQR.F | Vitronectin | BCP(HP) | 5.21 | 5.18 |
| I.SGMAPRPSLAKKQR.F | Vitronectin | BCP(HP) | 5.46 | 6.18 |
| A.PRPSLAKKQRF.R | Vitronectin | BCP(HP) | 5.48 | 5.95 |
| R.DWHGVPGQVDAAMAGRIYISGMAPRPSLAKKQRFR.H | Vitronectin | BCP(HP) | 5.85 | 6.26 |
| I.YISGMAPRPSLAKKQR.F | Vitronectin | BCP(HP) | 6.11 | 6.53 |
| R.IYISGMAPRPSLAKKQ.R | Vitronectin | BCP(HP) | 4.56 | 5.98 |
| A.PRPSLAKKQRFR.H | Vitronectin | HP | 5.76 | 4.38 |
| Y.ISGMAPRPSLAKKQRFR.H | Vitronectin | HP(BCP) | 5.98 | 5.90 |
| H.GVPGQVDAAMAGRIYISGMAPRPSLAKKQRF.R | Vitronectin | HP(BCP) | 6.11 | 6.00 |
| R.TSAGTRQPQFISRDWHGVPGQVDAAM(-hydroxylation)AG.R | Vitronectin | HP(BCP) | 6.99 | 7.34 |
| V.PGQVDAAM(-hydroxylation)AGR.I | Vitronectin | BCP&HP | 6.43 | 5.89 |
| R.TSAGTRQPQFISRDWHGVPGQVDAAMAGRIYISGMAPRPSLAK.K | Vitronectin | BCP(HP) | 5.49 | 5.92 |
| R.TSAGTRQPQFISRDWHGVPGQ(-loss HCOOH).V | Vitronectin | BCP(HP) | 5.82 | 6.18 |
| R.TSAGTRQPQFISRDWHGV(-dehydration).P | Vitronectin | BCP(HP) | 6.68 | 6.88 |
| R.TSAGTRQPQFISRDWHGVPGQ(-pyro-glu).V | Vitronectin | BCP(HP) | 5.89 | 6.11 |
| R.SIAQYWLGC(-dehydro)PAPGHL.-; R.SIAQYWLGC(-dehydro)PAPGHL.- | Vitronectin | HP | 6.08 | 4.36 |

*BCP: the peptide is only identified in the BCP; HP: the peptide is only identified in the control HP; BCP(HP) or HP (BCP): the peptide is identified in the BCP or the HP and might exist in the HP or the BCP due to observation of the molecular masses; A_HP_ and A_BCP_: the abundance (log value) of the HP and the BCP peptide, respectively. The identification method is described in the Methods section, and identification details for each peptide are available in the supplementary identification datasets.
