# Supplementary material for: Blood Peptidome-Degradome Profile of Breast Cancer
Source: PLoS One. 2010 Oct 18;5(10):e13133. doi: 10.1371/journal.pone.0013133 (PMC2956627; doi:10.1371/journal.pone.0013133)
Supplement: Table S3 — The MMPs identified from the pooled breast cancer patients (BCP) and control healthy persons (HP) blood plasma proteomic samples. (0.06 MB DOC) [file pone.0013133.s003.doc]

Table S3. The MMPs identified from the pooled breast cancer patients (BCP) and control healthy persons (HP) blood plasma proteomic samples.

| Secreted MMPs | UniTP ^a^  (BCP) | UniTP  (HP) |  | Membrane-linked MMPs | UniTP  (BCP) | UniTP  (HP) |
| --- | --- | --- | --- | --- | --- | --- |
| Matrilysin (MMP-7) | -- | -- |  | MT1-MMP (MMP-14) | -- | -- |
| Matrilysin (MMP-26) | -- | -- |  | MT2-MMP (MMP-15) | -- | -- |
| Collagenase-1 (MMP-1) | -- | -- |  | MT3-MMP (MMP-16) | -- | -- |
| Collagenase-2 (MMP-8) | -- | -- |  | MT4-MMP (MMP-17) | -- | -- |
| Collagenase-3 (MMP-13) | -- | -- |  | MT5-MMP (MMP-24) | -- | -- |
| Stromelysin-1 (MMP-3) | -- | -- |  | MT6-MMP (MMP-25) | -- | -- |
| Stromelysin-2 (MMP-10) | -- | -- |  | CA-MMP (MMP-23) | -- | -- |
| Metalloelastase (MMP-12) | -- | -- |  |  |  |  |
| RASI-1 (MMP-9) | -- | -- |  |  |  |  |
| Enamelysin (MMP-20) | -- | -- |  |  |  |  |
| MMP-27 (MMP-22, C-MMP) | -- | -- |  |  |  |  |
| Stromelysin-3 (MMP-11) | -- | -- |  |  |  |  |
| MMP-21 (X-MMP) | -- | -- |  |  |  |  |
| Epilysin (MMP-28) | -- | -- |  |  |  |  |
| Gelatinase A (MMP-2) | 8 | 7 |  |  |  |  |
| Gelatinase B (MMP-9) | 1^b^ | 0 |  |  |  |  |

^a^UniTP: unique tryptic peptides identified from the proteomics analysis; --: Not detected.

^b^The spectrum was manually inspected, and the identification is suspicious.
