# Supplementary material for: Blood Peptidome-Degradome Profile of Breast Cancer
Source: PLoS One. 2010 Oct 18;5(10):e13133. doi: 10.1371/journal.pone.0013133 (PMC2956627; doi:10.1371/journal.pone.0013133)
Supplement: Figure S1 — The HC1-3 composed of protein complexes IαI, PαI and IαIL that function to stabilize the ECM. The red and blue numbers represent the different peptidome peptides observed for the BCP and the control HP, respectively. (0.06 MB DOC) [file pone.0013133.s004.doc]

Figure S1

The HC1-3 compose of protein complexes IαI, PαI and IαIL that function to stabilize the ECM. The red and blue numbers represent the different peptidome peptides observed for the BCP and the control HP, respectively.
