# Supplementary material for: Blood Peptidome-Degradome Profile of Breast Cancer
Source: PLoS One. 2010 Oct 18;5(10):e13133. doi: 10.1371/journal.pone.0013133 (PMC2956627; doi:10.1371/journal.pone.0013133)
Supplement: Figure S2 — The selective degradation of the BCP complement system convertases and MAC components would lead to the system dysfunction. The key convertases C3b, C4b, and factor B and MAC components C8 and C9 in the complement system that limits tumor growth are differentially degraded on their function domains for the BCP; the grey arrows represent the activation routes to be limited due to the excessive degradation [1]. The red and blue numbers represent the different peptides observed for the BCP and the control HP, respectively. 1 Markiewski MM, Lambris JD (2009) Is complement good or bad for cancer patients? A new perspective on an old dilemma. Trends Immun 30: 286–292. (0.23 MB DOC) [file pone.0013133.s005.doc]

Figure S2

The selective degradation of the BCP complement system convertases and MAC components would lead to the system dysfunction. The key convertases C3b, C4b, and factor B and MAC components C8 and C9 in the complement system that limits tumor growth [Markiewski MM, Lambris JD (2009) Is complement good or bad for cancer patients? A new perspective on an old dilemma. *Trends Immun* 30: 286-292.] are differentially degraded on their function domains for the BCP; the grey arrows represent the activation routes to be limited due to the excessive degradation. The red and blue numbers represent the different peptides observed for the BCP and the control HP, respectively.
