# Supplementary material for: Blood Peptidome-Degradome Profile of Breast Cancer
Source: PLoS One. 2010 Oct 18;5(10):e13133. doi: 10.1371/journal.pone.0013133 (PMC2956627; doi:10.1371/journal.pone.0013133)
Supplement: Figure S3 — The BCP and the control HP peptidome peptide molecular weight distributions. The BCP and HP peptidomes were isolated with use of size exclusion chromatography. (0.08 MB DOC) [file pone.0013133.s006.doc]

Figure S3

The BCP and the control HP peptidome peptide molecular weight distributions. The BCP and HP peptidomes were isolated with use of size exclusion chromatography.
